# Supplementary material for: Adipose tissue-secreted Spz5 promotes distal tumor progression via Toll-6-mediated Hh pathway activation in Drosophila
Source: EMBO J. 2025 Jun 23;44(15):4301–30. doi: 10.1038/s44318-025-00489-y (PMC12317064; doi:10.1038/s44318-025-00489-y)
Supplement: Supplementary file 1 — Table EV1 [file 44318_2025_489_MOESM1_ESM.pdf]

# Table EV1 Oligonucleotides and *Drosophila* strains used in this study.

## 1. Oligonucleotides used in this study.

| Oligonucleotides | Sequence | IDENTIFIER |
|---|---|---|
| *AP-2α-K34* to R-F | 5-AAACGCATAAACAGAGAGTTGGCCA | N/A |
| *AP-2α-K34* to R-R | 5-TGGCCAACTCTCTGTTTATGCGTTT | N/A |
| *AP-2α-K290* to R-F | 5-CGATTTTGAACAGAGCGCAAGAGCC | N/A |
| *AP-2α-K290* to R-R | 5-GGCTCTTGCGCTCTGTTCAAAATCG | N/A |
| *AP-2α-K857* to R-F | 5-CAGAAAGTGTTCAGAGCTGCACAG | N/A |
| *AP-2α-K857* to R-R | 5-CTGTGCAGCTCTGAACACTTTCTG | N/A |
| DsRNA for *mib1* F1 | 5-TAATACGACTCACTATAGGGAGAGGGTGTGGTAGTGAGCAGTG | N/A |
| DsRNA for *mib1* R1 | 5-TCTCCCTATAGTGAGTCGTATTAGGAGAATCTTCGGACGAGGG | N/A |
| DsRNA for *mib1* F2 | 5-TAATACGACTCACTATAGGGAGATGTGCTCCATCTGCTACCAC | N/A |
| DsRNA for *mib1* R2 | 5-TCTCCCTATAGTGAGTCGTATTACCTCTGGCGAGCACTTTCTT | N/A |
| qPCR for *rp49* F | 5-CCACCAGTCGGATCGATATGC | N/A |
| qPCR for *rp49* R | 5-CTCTTGAGAACGCAGGCGACC | N/A |
| qPCR for *mib1* F | 5-GGAGAATCTTCGGACGAGGG | N/A |
| qPCR for *mib1* R: | 5-GGGTGTGGTAGTGAGCAGTG | N/A |
| qPCR-*spz5* -F | 5-GGAAAGACGTACTGCGAGCA | N/A |
| qPCR-*spz5* -R | 5-AAATCTTCCCAGGTTTCGTCC | N/A |
| qPCR-*Socs36E* -F | 5-GCACAGAAGGCAGACC | N/A |
| qPCR-*Socs36E* -R | 5-ACGTAGGAGACCCGTAT | N/A |
| qPCR-*TotA* -F | 5-CTGCTCTTATGTAAGTAGTATCGAAT | N/A |
| qPCR-*TotA* -R | 5-CAACGATCCTCGCCTTTCGACC | N/A |
| qPCR-*TotM* -F | 5-TCGACAGCCTGGTCACTTTC | N/A |
| qPCR-*TotM* -R | 5-ACCAAGACCACACGAGCATT | N/A |
| qPCR-*Pvf1* -F | 5-AAGCCGGAACACCATTGAC | N/A |
| qPCR-*Pvf1* -R | 5-CATGATGCTGCGCTTAAAGT | N/A |
| qPCR-*Pvf2* -F | 5-CCTATGCCCCAAGCACTTCA | N/A |
| qPCR-*Pvf2* -R | 5-CCCTCAACGCCGTTTTTCAG | N/A |
| qPCR-*Pvf3* -F | 5-AGCCAAATTTGTGCCGCCAAG | N/A |
| qPCR-*Pvf3* -R | 5-CTGCGATGCTTACTGCTCTTCACG | N/A |
| qPCR-*upd1* -F | 5-GCACACTGATTTCGATACGG | N/A |
| qPCR-*upd1* -R | 5-CTGCCGTGGTGCTGTTTT | N/A |
| qPCR-*upd2* --F | 5-GGCTCTTCTGCTGATCCTTG | N/A |
| qPCR-*upd2* -R | 5-AAGACTTGGTACCGCCACA | N/A |
| qPCR-*upd3* -F | 5-GCGGGGAGGATGTACC | N/A |
| qPCR-*upd3* -R | 5-GTCTTCATGGAATGAGCC | N/A |
| qPCR-*ban* -F | 5-ATT TGA CTA CGA AAC CGG TTT TCG | N/A |
| qPCR-*ban* -R | 5-CAG CTT TCA AAA TGA TCT CAC TTG | N/A |
| qPCR-*CycE* -F | 5-GAC ATC AGC CCC ATC ACC | N/A |
| qPCR-CycE-R | 5-TGC CAA TCT GGG AGA ACG | N/A |
| qPCR-*Mer* -F | 5-CCA AAG ACA ACG TTC CAG TG | N/A |
| qPCR-*Mer* -R | 5-GGA GGT CCT GCG AGT AAA AG | N/A |
| qPCR-*Myc* -F: | 5-AGCCAGAGATCCGCAACATC | N/A |
| qPCR-*Myc* -R | 5-CGCGCTGTAGAGATTCGTAGAG | N/A |
| qPCR-*ex*- F | 5-AGAGTGCACGAAGAGTGAGC | N/A |
| qPCR-*ex*- R | 5-AATCGCGAGCCTGGTGATAG | N/A |
| qPCR-*fj* -F | 5-AGGGATGCGGAAGAATGCAA | N/A |
| qPCR-*fj* -R | 5-GGCTGAAAGGGCTGTGGTAT | N/A |
| qPCR-*Diap1* -F | 5-AAATGCTTTTTCTGCGGCGT | N/A |
| qPCR-*Diap1* -R | 5-CTCATCTCCAGCGTCGAGTC | N/A |
| qPCR-*cact* -F | 5-CTGCATCTTTCCTGCATCGC | N/A |
| qPCR-*cact* -R | 5-CTCAGGGAACTGACCGCTTT | N/A |
| spz5 Cut&Run pF1 | 5-ACGTGTTATATACATATGTA | N/A |
| spz5 Cut&Run pR1 | 5-CACTTAAGCCGCTCATTAAT | N/A |
| spz5 Cut&Run pF2 | 5-CGGCAGACTGATGAAGTCAC | N/A |
| spz5 Cut&Run pR2 | 5-AGAAAAGTACGGGAAAACCC | N/A |
| spz5 Cut&Run pF3 | 5-AGTGAGTAAATCATGTAACCATAC | N/A |
| spz5 Cut&Run pR3 | 5-ATAGACCGAAATATATCTAGTAAG | N/A |

# 2. *Drosophila* strains used in this study.

| REAGENT or RESOURCE | SOURCE | IDENTIFIER |
|---|---|---|
| Experimental Models: *Drosophila melanogaster* | | |
| Drosophila melanogaster: w$^{1118}$ | Bloomington Drosophila Stock Center | Cat# 5905 |
| Drosophila melanogaster: alphaTub84B-QF2 | Bloomington Drosophila Stock Center | Cat# 51958 |
| Drosophila melanogaster: alphaTub84B-QS | Bloomington Drosophila Stock Center | Cat# 30034 |
| Drosophila melanogaster: QUAS-mCD8-GFP | Bloomington Drosophila Stock Center | Cat# 30002 |
| Drosophila melanogaster: QUAS-mtdTomato-3xHA, M2ET-QF | Bloomington Drosophila Stock Center | Cat# 30043 |
| Drosophila melanogaster: Act5C>y+>Gal4 | Bloomington Drosophila Stock Center | Cat# 3953 |
| Drosophila melanogaster: UAS-Toll-6$^{RNAi\#1}$ | Bloomington Drosophila Stock Center | Cat# 64968 |
| Drosophila melanogaster: UAS-yki$^{S168A.GFP.HA}$ | Bloomington Drosophila Stock Center | Cat# 28816 |
| Drosophila melanogaster: UAS-yki$^{S111A.S168A.S250A.V5}$ | Bloomington Drosophila Stock Center | Cat# 28817 |
| Drosophila melanogaster: UAS-smo$^{RNAi}$ | Bloomington Drosophila Stock Center | Cat# 24472 |
| Drosophila melanogaster: UAS-smo$^{ACT.Flag}$ | Bloomington Drosophila Stock Center | Cat# 44621 |
| Drosophila melanogaster: UAS-spz5$^{RNAi\#1}$ | Bloomington Drosophila Stock Center | Cat# 67229 |
| Drosophila melanogaster: UAS-bsk$^{DN}$ | Bloomington Drosophila Stock Center | Cat# 6409 |
| Drosophila melanogaster: UAS-Stat92E$^{RNAi\#2}$ | Bloomington Drosophila Stock Center | Cat# 35600 |
| Drosophila melanogaster: UAS-hop | Bloomington Drosophila Stock Center | Cat# 79033 |
| Drosophila melanogaster: UAS-pvf1$^{RNAi}$ | Bloomington Drosophila Stock Center | Cat# 39038 |
| Drosophila melanogaster: UAS-pvf2$^{RNAi}$ | Bloomington Drosophila Stock Center | Cat# 61955 |
| Drosophila melanogaster: UAS-pvf3$^{RNAi}$ | Bloomington Drosophila Stock Center | Cat# 38962 |
| Drosophila melanogaster: UAS-pvr$^{DN}$ | Bloomington Drosophila Stock Center | Cat# 58430 |
| Drosophila melanogaster: TRE-DsRed | Bloomington Drosophila Stock Center | Cat# 59012 |
| Drosophila melanogaster: pnt-lacZ | Bloomington Drosophila Stock Center | Cat# 11724 |
| Drosophila melanogaster: spi-lacZ | Bloomington Drosophila Stock Center | Cat# 10462 |
| Drosophila melanogaster: AP-2α$^{40\text{-}31}$ | Bloomington Drosophila Stock Center | Cat# 64752 |
| Drosophila melanogaster: UAS-Toll-6$^{RNAi\#2}$ | Vienna Drosophila Resource Center | Cat# v27102 |
| Drosophila melanogaster: UAS-Toll-6$^{RNAi\#3}$ | Vienna Drosophila Resource Center | Cat# v27103 |
| Drosophila melanogaster: UAS-mib1$^{RNAi\#1}$ | Vienna Drosophila Resource Center | Cat# v27525 |
| Drosophila melanogaster: UAS-upd1$^{RNAi}$ | Vienna Drosophila Resource Center | Cat# v3282 |
| Drosophila melanogaster: UAS-upd2$^{RNAi}$ | Vienna Drosophila Resource Center | Cat# v330691 |
| Drosophila melanogaster: UAS-upd3$^{RNAi}$ | Vienna Drosophila Resource Center | Cat# v27134 |
| Drosophila melanogaster: UAS-hop$^{RNAi}$ | Vienna Drosophila Resource Center | Cat# v40037 |
| Drosophila melanogaster: UAS-arm$^{RNAi}$ | Vienna Drosophila Resource Center | Cat# v107344 |
| Drosophila melanogaster: UAS-spz5$^{RNAi\#2}$ | Vienna Drosophila Resource Center | Cat# v41295 |
| Drosophila melanogaster: UAS-ci$^{RNAi}$ | National Institute of Genetics (NIG) | Cat# 2125R-2 |
| Drosophila melanogaster: UAS-mib1$^{RNAi\#2}$ | Tsinghua Fly Center | Cat# TH201501168.S |
| Drosophila melanogaster: UAS-Stat92E$^{RNAi\#1}$ | Tsinghua Fly Center | Cat# THU4483 |
| Drosophila melanogaster: UAS-pvr$^{RNAi}$ | Tsinghua Fly Center | Cat# THU5196 |
| Drosophila melanogaster: UAS-Toll-6$^{ACT.Flag}$ | Gift from Tian Xu (Westlake University) | Original source: (Mishra-Gorur, et al., 2019, DMM) |
| Drosophila melanogaster: scrib$^{1}$ | Gift from Tian Xu (Westlake University) | Original source: (Bilder, et al., 2000, Science) |
| Drosophila melanogaster: UAS-dome$^{DN}$ | Gift from Tian Xu (Westlake University) | Original source: (Ming Wu, et al., 2010, Nature) |
| Drosophila melanogaster: UAS-dome | Gift from Tian Xu (Westlake University) | N/A |
| Drosophila melanogaster: UAS-sd$^{ACT}$ | Gift from Tian Xu (Westlake University) | N/A |
| Drosophila melanogaster: UAS-sd | Gift from Tian Xu (Westlake University) | N/A |
| Drosophila melanogaster: UAS-sd$^{RNAi}$ | Gift from Tian Xu (Westlake University) | N/A |
| Drosophila melanogaster: UAS-myr.RFP | Gift from Tian Xu (Westlake University) | N/A |
| Drosophila melanogaster: FRT 40A | Gift from Tian Xu (Westlake University) | N/A |
| Drosophila melanogaster: FRT 79E | Gift from Tian Xu (Westlake University) | N/A |
| Drosophila melanogaster: FRT 82B | Gift from Tian Xu (Westlake University) | N/A |
| Drosophila melanogaster: yw, ey-Flp1; Tub-Gal80, FRT 40A; Act5C>y+>Gal4, UAS-GFP | Gift from Tian Xu (Westlake University) | N/A |
| Drosophila melanogaster: yw, ey-Flp1; Act5C>y+>Gal4, UAS-GFP; Tub-Gal80, FRT 79E | Gift from Tian Xu (Westlake University) | N/A |
| Drosophila melanogaster: yw, ey-Flp1; Act5C>y+>Gal4, UAS-GFP; FRT 82B, Tub-Gal80 | Gift from Tian Xu (Westlake University) | N/A |
| Drosophila melanogaster: yw, ey-Flp1; Act5C>y+>Gal4, UAS-RFP; FRT 82B, Tub-Gal80 | Gift from Tian Xu (Westlake University) | N/A |
| Drosophila melanogaster: yw, Ubx-Flp1; Act5C>y+>Gal4, UAS-GFP; FRT 82B, Tub-Gal80 | Gift from Tian Xu (Westlake University) | N/A |
| Drosophila melanogaster: UAS-ci$^{ACT.HA}$ | Gift from Lei Xue (Tongji University) | Original source: (Yang Chen, et al., 1999, Development) |
| Drosophila melanogaster: 10xStat-GFP | Gift from Lei Xue (Tongji University) | N/A |
| Drosophila melanogaster: NRE-GFP | Gift from Lei Xue (Tongji University) | N/A |
| Drosophila melanogaster: UAS-ptc | Gift from Lei Xue (Tongji University) | N/A |
| Drosophila melanogaster: Cg-Gal4 | Gift from Lei Xue (Tongji University) | N/A |
| Drosophila melanogaster: r4-Gal4 | Gift from Lei Xue (Tongji University) | N/A |
| Drosophila melanogaster: He-Gal4 | Gift from Lei Xue (Tongji University) | N/A |
| Drosophila melanogaster: ap-Gal4 | Gift from Lei Xue (Tongji University) | N/A |
| Drosophila melanogaster: dpp-lacZ | Gift from Zizhang Zhou (Shandong Agricultural University) | N/A |
| Drosophila melanogaster: UAS-AP-2α$^{HA}$ | This Paper | N/A |
| Drosophila melanogaster: QUAS-yki$^{WT}$ | This Paper | N/A |
| Drosophila melanogaster: QUAS-yki$^{S111A.S168A.S250A}$ | This Paper | N/A |
