# Supplementary material for: Adipose tissue-secreted Spz5 promotes distal tumor progression via Toll-6-mediated Hh pathway activation in Drosophila
Source: EMBO J. 2025 Jun 23;44(15):4301–30. doi: 10.1038/s44318-025-00489-y (PMC12317064; doi:10.1038/s44318-025-00489-y)
Supplement: Supplementary file 2 — Table EV2 [file 44318_2025_489_MOESM2_ESM.pdf]

Table EV2 Detailed genotypes used in this study, related to all figures

The detailed genotypes of each experiment are described below

**Fig 1.**	
**Fig 1A**	y, w, eyFLP1/+; Tub-QF2, QUAS-GFP/+; FRT82B, Tub-QS/FRT82B
	y, w, eyFLP1/+; Tub-QF2, QUAS-GFP/+; FRT82B, Tub-QS/FRT82B, scrib^{1}
	y, w, eyFLP1/+; Tub-QF2, QUAS-GFP/+; FRT82B, Tub-QS/QUAS-yki^{WT}, FRT82B
	y, w, eyFLP1/+; Tub-QF2, QUAS-GFP/+; FRT82B, Tub-QS/QUAS-yki^{WT}, FRT82B, scrib^{1}
	y, w, eyFLP1/+; Tub-QF2, QUAS-GFP/+; FRT82B, Tub-QS/QUAS-yki$^{S111A.S168A.S250A}$, FRT82B
	y, w, eyFLP1/+; Tub-QF2, QUAS-GFP/+; FRT82B, Tub-QS/QUAS-yki$^{S111A.S168A.S250A}$, FRT82B, scrib^{1}
**Fig 1D**	y, w, eyFLP1/+; Tub-QF2, QUAS-GFP/+; FRT82B, Tub-QS/QUAS-yki$^{S111A.S168A.S250A}$, FRT82B
	y, w, eyFLP1/+; Tub-QF2, QUAS-GFP/+; FRT82B, Tub-QS/QUAS-yki$^{S111A.S168A.S250A}$, FRT82B, scrib^{1}
**Fig 1F**	y, w, eyFLP1/+; Tub-QF2, QUAS-GFP, Act5C>y^{+}>Gal4/+; FRT82B, Tub-Gal80, Tub-QS/FRT82B
	y, w, eyFLP1/+; Tub-QF2, QUAS-GFP, Act5C>y^{+}>Gal4/+; FRT82B, Tub-Gal80, Tub-QS/QUAS-yki$^{S111A.S168A.S250A}$, FRT82B, scrib^{1}
	y, w, eyFLP1/+; Tub-QF2, QUAS-GFP, Act5C>y^{+}>Gal4/UAS-Toll-6$^{RNAi\#1}$; FRT82B, Tub-Gal80, Tub-QS/QUAS-yki$^{S111A.S168A.S250A}$, FRT82B, UAS-Toll-6$^{RNAi\#2}$, UAS-Toll-6$^{RNAi\#3}$, scrib^{1}
**Fig 1J**	y, w, eyFLP1/+; Tub-QF2, QUAS-GFP, Cg-Gal4/UAS-RFP; FRT82B, Tub-QS/FRT82B
**Fig 1K**	y, w, eyFLP1/+; Tub-QF2, QUAS-GFP/UAS-RFP; r4-Gal4, FRT82B, Tub-QS/FRT82B
**Fig 1L**	y, w, eyFLP1/+; Tub-QF2, QUAS-GFP/UAS-RFP; He-Gal4, FRT82B, Tub-QS/FRT82B
**Fig 1M**	y, w, eyFLP1/+; Tub-QF2, QUAS-GFP, Cg-Gal4/+; FRT82B, Tub-QS/QUAS-yki$^{S111A.S168A.S250A}$, FRT82B, scrib^{1}
	y, w, eyFLP1/+; Tub-QF2, QUAS-GFP, Cg-Gal4/UAS-spz5$^{RNAi\#1}$; FRT82B, Tub-QS/QUAS-yki$^{S111A.S168A.S250A}$, FRT82B, scrib^{1}
	y, w, eyFLP1/+; Tub-QF2, QUAS-GFP, Cg-Gal4/UAS-spz5$^{RNAi\#2}$; FRT82B, Tub-QS/QUAS-yki$^{S111A.S168A.S250A}$, FRT82B, scrib^{1}
**Fig 2.**	
**Fig 2A**	y, w, eyFLP1/+; Act5C>y^{+}>Gal4, UAS-GFP/+; FRT82B, Tub-Gal80/FRT82B
	y, w, eyFLP1/+; Act5C>y^{+}>Gal4, UAS-GFP/+; FRT82B, Tub-Gal80/FRT82B, UAS-Toll-6^{ACT}
	y, w, eyFLP1/+; Act5C>y^{+}>Gal4, UAS-GFP/+; FRT82B, Tub-Gal80/UAS-yki$^{S168A.GFP}$, FRT82B
	y, w, eyFLP1/+; Act5C>y^{+}>Gal4, UAS-GFP/+; FRT82B, Tub-Gal80/UAS-yki$^{S168A.GFP}$, FRT82B, UAS-Toll-6^{ACT}
	y, w, eyFLP1/+; Act5C>y^{+}>Gal4, UAS-GFP/+; FRT82B, Tub-Gal80/UAS-yki$^{S111A.S168A.S250A}$, FRT82B
	y, w, eyFLP1/+; Act5C>y^{+}>Gal4, UAS-GFP/+; FRT82B, Tub-Gal80/UAS-yki$^{S111A.S168A.S250A}$, FRT82B, UAS-Toll-6^{ACT}
**Fig 2D**	y, w, eyFLP1/+; Act5C>y^{+}>Gal4, UAS-GFP/+; FRT82B, Tub-Gal80/FRT82B
	y, w, eyFLP1/+; Act5C>y^{+}>Gal4, UAS-GFP/+; FRT82B, Tub-Gal80/UAS-yki$^{S168A.GFP}$, FRT82B, UAS-Toll-6^{ACT}
**Fig 2F**	y, w, eyFLP1/+; Act5C>y^{+}>Gal4, UAS-GFP/+; FRT82B, Tub-Gal80/FRT82B
	y, w, eyFLP1/+; Act5C>y^{+}>Gal4, UAS-GFP/+; FRT82B, Tub-Gal80/FRT82B, UAS-Toll-6^{ACT}
	y, w, eyFLP1/+; Act5C>y^{+}>Gal4, UAS-GFP/+; FRT82B, Tub-Gal80/UAS-yki$^{S168A.GFP}$, FRT82B
	y, w, eyFLP1/+; Act5C>y^{+}>Gal4, UAS-GFP/+; FRT82B, Tub-Gal80/UAS-yki$^{S168A.GFP}$, FRT82B, UAS-Toll-6^{ACT}
**Fig 2G**	y, w, eyFLP1/+; Act5C>y^{+}>Gal4, UAS-GFP/+; FRT82B, Tub-Gal80/FRT82B
	y, w, eyFLP1/+; Act5C>y^{+}>Gal4, UAS-GFP/+; FRT82B, Tub-Gal80/FRT82B, UAS-Toll-6^{ACT}
	y, w, eyFLP1/+; Act5C>y^{+}>Gal4, UAS-GFP/+; FRT82B, Tub-Gal80/UAS-yki$^{S168A.GFP}$, FRT82B
	y, w, eyFLP1/+; Act5C>y^{+}>Gal4, UAS-GFP/+; FRT82B, Tub-Gal80/UAS-yki$^{S168A.GFP}$, FRT82B, UAS-Toll-6^{ACT}
**Fig 3.**	
**Fig 3A**	y, w, eyFLP1/+; Act5C>y^{+}>Gal4, UAS-GFP/+; FRT82B, Tub-Gal80/FRT82B
	y, w, eyFLP1/+; Act5C>y^{+}>Gal4, UAS-GFP/+; FRT82B, Tub-Gal80/FRT82B, UAS-Toll-6^{ACT}
**Fig 3B**	y, w, eyFLP1/+; Act5C>y^{+}>Gal4, UAS-GFP/+; FRT82B, Tub-Gal80/FRT82B
	y, w, eyFLP1/+; Act5C>y^{+}>Gal4, UAS-GFP/+; FRT82B, Tub-Gal80/FRT82B, UAS-Toll-6^{ACT}
**Fig 3C**	y, w, eyFLP1/+; Act5C>y^{+}>Gal4, UAS-GFP/dpp-lacZ; FRT82B, Tub-Gal80/FRT82B
	y, w, eyFLP1/+; Act5C>y^{+}>Gal4, UAS-GFP/dpp-lacZ; FRT82B, Tub-Gal80/FRT82B, UAS-Toll-6^{ACT}
**Fig 3D**	y, w, eyFLP1/+; Act5C>y^{+}>Gal4, UAS-GFP/+; FRT82B, Tub-Gal80/FRT82B
	y, w, eyFLP1/+; Act5C>y^{+}>Gal4, UAS-GFP/+; FRT82B, Tub-Gal80/UAS-yki$^{S168A.GFP}$, FRT82B
	y, w, eyFLP1/+; Act5C>y^{+}>Gal4, UAS-GFP/+; FRT82B, Tub-Gal80/UAS-yki$^{S168A.GFP}$, FRT82B, UAS-Toll-6^{ACT}
	y, w, eyFLP1/+; Act5C>y^{+}>Gal4, UAS-GFP/UAS-ci^{RNAi}; FRT82B, Tub-Gal80/UAS-yki$^{S168A.GFP}$, FRT82B, UAS-Toll-6^{ACT}
	y, w, eyFLP1/+; Act5C>y^{+}>Gal4, UAS-GFP/UAS-smo^{RNAi}; FRT82B, Tub-Gal80/UAS-yki$^{S168A.GFP}$, FRT82B, UAS-Toll-6^{ACT}
	y, w, eyFLP1/+; Act5C>y^{+}>Gal4, UAS-GFP/UAS-ptc; FRT82B, Tub-Gal80/UAS-yki$^{S168A.GFP}$, FRT82B, UAS-Toll-6^{ACT}
	y, w, eyFLP1/+; Act5C>y^{+}>Gal4, UAS-GFP/+; FRT82B, Tub-Gal80/UAS-yki$^{S168A.GFP}$, FRT82B, UAS-ci^{ACT}
	y, w, eyFLP1/+; Act5C>y^{+}>Gal4, UAS-GFP/UAS-smo^{ACT}; FRT82B, Tub-Gal80/UAS-yki$^{S168A.GFP}$, FRT82B
**Fig 3F**	y, w, eyFLP1/+; Act5C>y^{+}>Gal4, UAS-GFP/+; FRT82B, Tub-Gal80/FRT82B
	y, w, eyFLP1/+; Act5C>y^{+}>Gal4, UAS-GFP/+; FRT82B, Tub-Gal80/UAS-yki$^{S168A.GFP}$, FRT82B, UAS-ci^{ACT}
**Fig 3G**	y, w, eyFLP1/+; Tub-QF2, QUAS-GFP, Act5C>y+>Gal4/+; FRT82B, Tub-Gal80, Tub-QS/FRT82B
	y, w, eyFLP1/+; Tub-QF2, QUAS-GFP, Act5C>y+>Gal4/+; FRT82B, Tub-Gal80, Tub-QS/QUAS-yki$^{S111A.S168A.S250A}$, FRT82B, scrib^{1}
	y, w, eyFLP1/+; Tub-QF2, QUAS-GFP, Act5C>y+>Gal4/UAS-ci^{RNAi}; FRT82B, Tub-Gal80, Tub-QS/QUAS-yki$^{S111A.S168A.S250A}$, FRT82B, scrib^{1}
	y, w, eyFLP1/+; Tub-QF2, QUAS-GFP, Act5C>y+>Gal4/UAS-smo^{RNAi}; FRT82B, Tub-Gal80, Tub-QS/QUAS-yki$^{S111A.S168A.S250A}$, FRT82B, scrib^{1}
	y, w, eyFLP1/+; Tub-QF2, QUAS-GFP, Act5C>y+>Gal4/UAS-ptc; FRT82B, Tub-Gal80, Tub-QS/QUAS-yki$^{S111A.S168A.S250A}$, FRT82B, scrib^{1}
**Fig 4.**	
**Fig 4A**	y, w, eyFLP1/+; Act5C>y^{+}>Gal4, UAS-GFP/+; FRT82B, Tub-Gal80/FRT82B
	y, w, eyFLP1/+; Act5C>y^{+}>Gal4, UAS-GFP/+; FRT82B, Tub-Gal80/FRT82B, UAS-Toll-6^{ACT}
**Fig 4B**	y, w, eyFLP1/+; Act5C>y^{+}>Gal4, UAS-GFP/+; FRT82B, Tub-Gal80/FRT82B
	y, w, eyFLP1/+; Act5C>y^{+}>Gal4, UAS-GFP/+; FRT82B, Tub-Gal80/FRT82B, UAS-Toll-6^{ACT}
**Fig 4C**	y, w, eyFLP1/+; Act5C>y^{+}>Gal4, UAS-GFP/+; FRT82B, Tub-Gal80/UAS-AP-2α^{HA}, FRT82B, UAS-Toll-6$^{ACT.Flag}$
**Fig 4D**	y, w, eyFLP1/+; Act5C>y^{+}>Gal4, UAS-GFP/+; FRT82B, Tub-Gal80/FRT82B
	y, w, eyFLP1/+; Act5C>y^{+}>Gal4, UAS-GFP/+; FRT82B, Tub-Gal80/FRT82B, UAS-Toll-6^{ACT}
**Fig 4E**	y, w, eyFLP1/+; Act5C>y^{+}>Gal4, UAS-GFP/+; FRT82B, Tub-Gal80/FRT82B
	y, w, eyFLP1/+; Act5C>y^{+}>Gal4, UAS-GFP/+; FRT82B, Tub-Gal80/FRT82B, UAS-Toll-6^{ACT}
	y, w, eyFLP1/+; Act5C>y^{+}>Gal4, UAS-GFP/+; FRT82B, Tub-Gal80/UAS-AP-2α^{HA}, FRT82B, UAS-Toll-6^{ACT}
**Fig 4G**	y, w, eyFLP1/+; Act5C>y^{+}>Gal4, UAS-GFP/+; FRT82B, Tub-Gal80/FRT82B
	y, w, eyFLP1/+; Act5C>y^{+}>Gal4, UAS-GFP/+; FRT82B, Tub-Gal80/FRT82B, UAS-Toll-6^{ACT}
	y, w, eyFLP1/+; Act5C>y^{+}>Gal4, UAS-GFP/+; FRT82B, Tub-Gal80/UAS-AP-2α^{HA}, FRT82B, UAS-Toll-6^{ACT}
	y, w, eyFLP1/+; Tub-Gal80, FRT40A/AP-2α$^{40-31}$, FRT40A; Act5C>y^{+}>Gal4, UAS-GFP/+
**Fig 4H**	y, w, eyFLP1/+; Act5C>y^{+}>Gal4, UAS-GFP/+; FRT82B, Tub-Gal80/FRT82B
	y, w, eyFLP1/+; Act5C>y^{+}>Gal4, UAS-GFP/+; FRT82B, Tub-Gal80/UAS-yki$^{S168A.GFP}$, FRT82B
	y, w, eyFLP1/+; Act5C>y^{+}>Gal4, UAS-GFP/+; FRT82B, Tub-Gal80/UAS-yki$^{S168A.GFP}$, FRT82B, UAS-Toll-6^{ACT}
	y, w, eyFLP1/+; Act5C>y^{+}>Gal4, UAS-GFP/+; FRT82B, Tub-Gal80/UAS-AP-2α^{HA}, UAS-yki$^{S168A.GFP}$, FRT82B, UAS-Toll-6^{ACT}
	y, w, eyFLP1/+; Tub-Gal80, FRT40A/AP-2α$^{40-31}$, FRT40A; Act5C>y^{+}>Gal4, UAS-GFP/UAS-yki$^{S168A.GFP}$
**Fig 5.**	
**Fig 5E**	y, w, eyFLP1/+; Act5C>y^{+}>Gal4, UAS-GFP/+; FRT82B, Tub-Gal80/FRT82B
	y, w, eyFLP1/+; Act5C>y^{+}>Gal4, UAS-GFP/+; FRT82B, Tub-Gal80/FRT82B, UAS-Toll-6^{ACT}
	y, w, eyFLP1/+; Act5C>y^{+}>Gal4, UAS-GFP/UAS-mib1$^{RNAi\#1}$; FRT82B, Tub-Gal80/FRT82B, UAS-Toll-6^{ACT}
**Fig 5F**	y, w, eyFLP1/+; Act5C>y^{+}>Gal4, UAS-GFP/+; FRT82B, Tub-Gal80/FRT82B
	y, w, eyFLP1/+; Act5C>y^{+}>Gal4, UAS-GFP/+; FRT82B, Tub-Gal80/FRT82B, UAS-Toll-6^{ACT}
	y, w, eyFLP1/+; Act5C>y^{+}>Gal4, UAS-GFP/UAS-mib1$^{RNAi\#1}$; FRT82B, Tub-Gal80/FRT82B, UAS-Toll-6^{ACT}
**Fig 5G**	y, w, eyFLP1/+; Act5C>y^{+}>Gal4, UAS-GFP/+; FRT82B, Tub-Gal80/FRT82B
	y, w, eyFLP1/+; Act5C>y^{+}>Gal4, UAS-GFP/+; FRT82B, Tub-Gal80/UAS-yki$^{S168A.GFP}$, FRT82B
	y, w, eyFLP1/+; Act5C>y^{+}>Gal4, UAS-GFP/+; FRT82B, Tub-Gal80/UAS-yki$^{S168A.GFP}$, FRT82B, UAS-Toll-6^{ACT}
	y, w, eyFLP1/+; Act5C>y^{+}>Gal4, UAS-GFP/UAS-mib1$^{RNAi\#1}$; FRT82B, Tub-Gal80/UAS-yki$^{S168A.GFP}$, FRT82B, UAS-Toll-6^{ACT}
	y, w, eyFLP1/+; Act5C>y^{+}>Gal4, UAS-GFP/UAS-mib1$^{RNAi\#2}$; FRT82B, Tub-Gal80/UAS-yki$^{S168A.GFP}$, FRT82B, UAS-Toll-6^{ACT}
**Fig 6.**	
**Fig 6B**	y, w, eyFLP1/+; Tub-QF2, QUAS-GFP, Act5C>y+>Gal4/+; FRT82B, Tub-Gal80, Tub-QS/QUAS-yki$^{S111A.S168A.S250A}$, FRT82B, scrib^{1}
	y, w, eyFLP1/+; Tub-QF2, QUAS-GFP, Act5C>y+>Gal4/UAS-upd1^{RNAi}; FRT82B, Tub-Gal80, Tub-QS/QUAS-yki$^{S111A.S168A.S250A}$, FRT82B, scrib^{1}
	y, w, eyFLP1/+; Tub-QF2, QUAS-GFP, Act5C>y+>Gal4/UAS-upd2^{RNAi}; FRT82B, Tub-Gal80, Tub-QS/QUAS-yki$^{S111A.S168A.S250A}$, FRT82B, scrib^{1}
	y, w, eyFLP1/+; Tub-QF2, QUAS-GFP, Act5C>y+>Gal4/UAS-upd3^{RNAi}; FRT82B, Tub-Gal80, Tub-QS/QUAS-yki$^{S111A.S168A.S250A}$, FRT82B, scrib^{1}
**Fig 6F**	y, w, eyFLP1/+; M2ET-QF, QUAS-mtdTomato-3xHA/10xStat-GFP; FRT82B, Tub-QS/FRT82B
	y, w, eyFLP1/+; M2ET-QF, QUAS-mtdTomato-3xHA/10xStat-GFP; FRT82B, Tub-QS/QUAS-yki$^{S111A.S168A.S250A}$, FRT82B
	y, w, eyFLP1/+; M2ET-QF, QUAS-mtdTomato-3xHA/10xStat-GFP; FRT82B, Tub-QS/QUAS-yki$^{S111A.S168A.S250A}$, FRT82B, scrib^{1}
**Fig 6I**	y, w, eyFLP1/+; Tub-QF2, QUAS-GFP/+; r4-Gal4, FRT82B, Tub-QS/QUAS-yki$^{S111A.S168A.S250A}$, FRT82B, scrib^{1}
	y, w, eyFLP1/+; Tub-QF2, QUAS-GFP/UAS-dome^{DN}; r4-Gal4, FRT82B, Tub-QS/QUAS-yki$^{S111A.S168A.S250A}$, FRT82B, scrib^{1}

y, w, eyFLP1/+; Tub-QF2, QUAS-GFP/UAS-hop^{RNAi} ; r4-Gal4, FRT82B, Tub-QS/QUAS-yki$^{S111A.S168A.S250A}$, FRT82B, scrib^{1}
y, w, eyFLP1/+; Tub-QF2, QUAS-GFP/UAS-Stat92E$^{RNAi\#2}$; r4-Gal4, FRT82B, Tub-QS/QUAS-yki$^{S111A.S168A.S250A}$, FRT82B, scrib^{1}

Fig 6M +/+;r4-Gal4/+
+/UAS-hop;r4-Gal4/+
+/+;r4-Gal4/UAS-dome
+/UAS-sd^{RNAi} ;r4-Gal4/UAS-dome
+/UAS-sd^{ACT} ;r4-Gal4/+
+/UAS-sd;r4-Gal4/UAS-yki$^{S168A.GFP}$

Fig 6O y, w, eyFLP1/+; Tub-QF2, QUAS-GFP/+; r4-Gal4, FRT82B, Tub-QS/QUAS-yki$^{S111A.S168A.S250A}$, FRT82B, scrib^{1}
y, w, eyFLP1/+; Tub-QF2, QUAS-GFP/UAS-sd^{RNAi} ; r4-Gal4, FRT82B, Tub-QS/QUAS-yki$^{S111A.S168A.S250A}$, FRT82B, scrib^{1}

Fig S1.

Fig S1A y, w, eyFLP1/+; Tub-QF2, QUAS-GFP/+; FRT82B, Tub-QS/FRT82B
y, w, eyFLP1/+; Tub-QF2, QUAS-GFP/+; FRT82B, Tub-QS/QUAS-yki$^{S111A.S168A.S250A}$, FRT82B
y, w, eyFLP1/+; Tub-QF2, QUAS-GFP/+; FRT82B, Tub-QS/QUAS-yki$^{S111A.S168A.S250A}$, FRT82B, scrib^{1}

Fig S1B y, w, eyFLP1/+; Tub-QF2, QUAS-GFP, Act5C>y+>Gal4/UAS-RFP; FRT82B, Tub-Gal80, Tub-QS/FRT82B

Fig S1D y, w, eyFLP1/+; Tub-QF2, QUAS-GFP/+; r4-Gal4, FRT82B, Tub-QS/QUAS-yki$^{S111A.S168A.S250A}$, FRT82B, scrib^{1}
y, w, eyFLP1/+; Tub-QF2, QUAS-GFP/UAS-spz5$^{RNAi\#1}$; r4-Gal4, FRT82B, Tub-QS/QUAS-yki$^{S111A.S168A.S250A}$, FRT82B, scrib^{1}
y, w, eyFLP1/+; Tub-QF2, QUAS-GFP/UAS-spz5$^{RNAi\#2}$; r4-Gal4, FRT82B, Tub-QS/QUAS-yki$^{S111A.S168A.S250A}$, FRT82B, scrib^{1}

Fig S1G y, w, eyFLP1/+; Tub-QF2, QUAS-GFP/+; He-Gal4, FRT82B, Tub-QS/QUAS-yki$^{S111A.S168A.S250A}$, FRT82B, scrib^{1}
y, w, eyFLP1/+; Tub-QF2, QUAS-GFP/UAS-spz5$^{RNAi\#1}$; He-Gal4, FRT82B, Tub-QS/QUAS-yki$^{S111A.S168A.S250A}$, FRT82B, scrib^{1}
y, w, eyFLP1/+; Tub-QF2, QUAS-GFP/UAS-spz5$^{RNAi\#2}$; He-Gal4, FRT82B, Tub-QS/QUAS-yki$^{S111A.S168A.S250A}$, FRT82B, scrib^{1}

Fig S2.

Fig S2A y, w, eyFLP1/+; Tub-QF2, QUAS-GFP, Act5C>y+>Gal4/+; FRT82B, Tub-Gal80, Tub-QS/FRT82B
y, w, eyFLP1/+; Tub-QF2, QUAS-GFP, Act5C>y+>Gal4/+; FRT82B, Tub-Gal80, Tub-QS/QUAS-yki$^{S111A.S168A.S250A}$, FRT82B
y, w, eyFLP1/+; Tub-QF2, QUAS-GFP, Act5C>y+>Gal4/+; FRT82B, Tub-Gal80, Tub-QS/QUAS-yki$^{S111A.S168A.S250A}$, FRT82B, UAS-Toll-6^{ACT}

Fig S2E y, w, eyFLP1/+; Act5C>y^{+}>Gal4 , UAS-GFP/+; FRT82B, Tub-Gal80/UAS-yki$^{S168A.GFP}$, FRT82B
y, w, eyFLP1/+; Act5C>y^{+}>Gal4 , UAS-GFP/+; FRT82B, Tub-Gal80/UAS-yki$^{S168A.GFP}$, FRT82B, UAS-Toll-6^{ACT}

Fig S2G y, w, UbxFLP/+; Act5C>y^{+}>Gal4 , UAS-GFP/+; FRT82B, Tub-Gal80/FRT82B
y, w, UbxFLP/+; Act5C>y^{+}>Gal4 , UAS-GFP/+; FRT82B, Tub-Gal80/FRT82B, UAS-Toll-6^{ACT}
y, w, UbxFLP/+; Act5C>y^{+}>Gal4 , UAS-GFP/+; FRT82B, Tub-Gal80/UAS-yki$^{S168A.GFP}$, FRT82B
y, w, UbxFLP/+; Act5C>y^{+}>Gal4 , UAS-GFP/+; FRT82B, Tub-Gal80/UAS-yki$^{S168A.GFP}$, FRT82B, UAS-Toll-6^{ACT}

Fig S2J ap-Gal4, UAS-RFP/+; UAS-yki$^{S168A.GFP}$ /+
ap-Gal4, UAS-RFP/+; UAS-yki$^{S168A.GFP}$, UAS-Toll-6^{ACT} /+

Fig S2O y, w, eyFLP1/+; Act5C>y^{+}>Gal4 , UAS-GFP/+; +/+
y, w, eyFLP1/+; Act5C>y^{+}>Gal4 , UAS-GFP/+; UAS-Toll-6^{ACT} /UAS-Toll-6^{ACT}

Fig S2P y, w, eyFLP1/+; Act5C>y^{+}>Gal4, UAS-GFP/+; FRT82B, Tub-Gal80/FRT82B
y, w, eyFLP1/+; Act5C>y^{+}>Gal4, UAS-GFP/+; FRT82B, Tub-Gal80/FRT82B, UAS-Toll-6^{ACT}

Fig S2Q y, w, eyFLP1/+; Act5C>y^{+}>Gal4, UAS-GFP/+; FRT82B, Tub-Gal80/FRT82B
y, w, eyFLP1/+; Act5C>y^{+}>Gal4, UAS-GFP/+; FRT82B, Tub-Gal80/FRT82B, UAS-Toll-6^{ACT}

Fig S3.

Fig S3A-S3C y, w, eyFLP1/+; Act5C>y^{+}>Gal4 , UAS-GFP/+; FRT82B, Tub-Gal80/FRT82B, UAS-Toll-6^{ACT}

Fig S3D y, w, eyFLP1/+; Act5C>y^{+}>Gal4 , UAS-RFP/NRE-GFP; FRT82B, Tub-Gal80/FRT82B, UAS-Toll-6^{ACT}

Fig S3E y, w, eyFLP1/+; Act5C>y^{+}>Gal4 , UAS-GFP/+; FRT82B, Tub-Gal80/FRT82B
y, w, eyFLP1/+; Act5C>y^{+}>Gal4 , UAS-GFP/+; FRT82B, Tub-Gal80/FRT82B, UAS-Toll-6^{ACT}

Fig S3F y, w, eyFLP1/+; Act5C>y^{+}>Gal4 , UAS-GFP/spi-lacZ; FRT82B, Tub-Gal80/FRT82B
y, w, eyFLP1/+; Act5C>y^{+}>Gal4 , UAS-GFP/spi-lacZ; FRT82B, Tub-Gal80/FRT82B, UAS-Toll-6^{ACT}

Fig S3G y, w, eyFLP1/+; Act5C>y^{+}>Gal4 , UAS-GFP/+; FRT82B, Tub-Gal80/FRT82B
y, w, eyFLP1/+; Act5C>y^{+}>Gal4 , UAS-GFP/+; FRT82B, Tub-Gal80/FRT82B, UAS-Toll-6^{ACT}

Fig S3H y, w, eyFLP1/+; Act5C>y^{+}>Gal4 , UAS-GFP/+; Tub-Gal80, FRT79E/FRT79E, pnt-lacZ
y, w, eyFLP1/+; Act5C>y^{+}>Gal4 , UAS-GFP/+; Tub-Gal80, FRT79E/FRT79E, pnt-lacZ, UAS-Toll-6^{ACT}

Fig S3I y, w, eyFLP1/+; Act5C>y^{+}>Gal4 , UAS-GFP/+; FRT82B, Tub-Gal80/UAS-yki$^{S168A.GFP}$, FRT82B
y, w, eyFLP1/+; Act5C>y^{+}>Gal4 , UAS-GFP/+; FRT82B, Tub-Gal80/UAS-yki$^{S168A.GFP}$, FRT82B, UAS-Toll-6^{ACT}

Fig S3J y, w, eyFLP1/+; Act5C>y^{+}>Gal4 , UAS-GFP/+; FRT82B, Tub-Gal80/FRT82B
y, w, eyFLP1/+; Act5C>y^{+}>Gal4 , UAS-GFP/UAS-ci^{RNAi} ; FRT82B, Tub-Gal80/FRT82B
y, w, eyFLP1/+; Act5C>y^{+}>Gal4 , UAS-GFP/+; FRT82B, Tub-Gal80/FRT82B, UAS-ci^{ACT}

Fig S3K y, w, eyFLP1/+; Act5C>y^{+}>Gal4 , UAS-GFP/+; FRT82B, Tub-Gal80/FRT82B
y, w, eyFLP1/+; Act5C>y^{+}>Gal4 , UAS-GFP/UAS-smo^{RNAi} ; FRT82B, Tub-Gal80/FRT82B
y, w, eyFLP1/+; Act5C>y^{+}>Gal4 , UAS-GFP/UAS-smo^{ACT} ; FRT82B, Tub-Gal80/FRT82B

Fig S3L y, w, eyFLP1/+; Act5C>y^{+}>Gal4 , UAS-GFP/+; FRT82B, Tub-Gal80/FRT82B
y, w, eyFLP1/+; Act5C>y^{+}>Gal4 , UAS-GFP/UAS-ptc; FRT82B, Tub-Gal80/FRT82B

Fig S3N y, w, eyFLP1/+; Act5C>y^{+}>Gal4 , UAS-GFP/+; FRT82B, Tub-Gal80/FRT82B
y, w, eyFLP1/+; Act5C>y^{+}>Gal4 , UAS-GFP/+; FRT82B, Tub-Gal80/UAS-yki$^{S168A.GFP}$, FRT82B, UAS-Toll-6^{ACT}
y, w, eyFLP1/+; Act5C>y^{+}>Gal4 , UAS-GFP/UAS-ci^{RNAi} ; FRT82B, Tub-Gal80/UAS-yki$^{S168A.GFP}$, FRT82B, UAS-Toll-6^{ACT}
y, w, eyFLP1/+; Act5C>y^{+}>Gal4 , UAS-GFP/UAS-smo^{RNAi} ; FRT82B, Tub-Gal80/UAS-yki$^{S168A.GFP}$, FRT82B, UAS-Toll-6^{ACT}
y, w, eyFLP1/+; Act5C>y^{+}>Gal4 , UAS-GFP/UAS-ptc; FRT82B, Tub-Gal80/UAS-yki$^{S168A.GFP}$, FRT82B, UAS-Toll-6^{ACT}
y, w, eyFLP1/+; Act5C>y^{+}>Gal4 , UAS-GFP/+; FRT82B, Tub-Gal80/UAS-yki$^{S168A.GFP}$, FRT82B, UAS-ci^{ACT}
y, w, eyFLP1/+; Act5C>y^{+}>Gal4 , UAS-GFP/UAS-smo^{ACT} ; FRT82B, Tub-Gal80/UAS-yki$^{S168A.GFP}$, FRT82B

Fig S3Q y, w, eyFLP1/+; Act5C>y^{+}>Gal4 , UAS-RFP/+; FRT82B, Tub-Gal80/UAS-yki$^{S168A.GFP}$, FRT82B
y, w, eyFLP1/+; Act5C>y^{+}>Gal4 , UAS-RFP/+; FRT82B, Tub-Gal80/UAS-yki$^{S168A.GFP}$, FRT82B, UAS-ci^{ACT}

Fig S4.

Fig S4A y, w, eyFLP1/+; Act5C>y^{+}>Gal4 , UAS-GFP/+; FRT82B, Tub-Gal80/UAS-yki$^{S168A.GFP}$, FRT82B, UAS-Toll-6^{ACT}
y, w, eyFLP1/UAS-bsk^{DN} ; Act5C>y^{+}>Gal4 , UAS-GFP/+; FRT82B, Tub-Gal80/UAS-yki$^{S168A.GFP}$, FRT82B, UAS-Toll-6^{ACT}

Fig S4C y, w, eyFLP1/+; Act5C>y^{+}>Gal4 , UAS-GFP/+; FRT82B, Tub-Gal80/UAS-yki$^{S168A.GFP}$, FRT82B, UAS-Toll-6^{ACT}
y, w, eyFLP1/+; Act5C>y^{+}>Gal4 , UAS-GFP/UAS-ci^{RNAi} ; FRT82B, Tub-Gal80/UAS-yki$^{S168A.GFP}$, FRT82B, UAS-Toll-6^{ACT}

Fig S4E y, w, eyFLP1/+; Act5C>y^{+}>Gal4 , UAS-GFP/+; FRT82B, Tub-Gal80/FRT82B
y, w, eyFLP1/+; Act5C>y^{+}>Gal4 , UAS-GFP/+; FRT82B, Tub-Gal80/FRT82B, UAS-ci^{ACT}
y, w, eyFLP1/+; Act5C>y^{+}>Gal4 , UAS-GFP/+; FRT82B, Tub-Gal80/FRT82B, UAS-Toll-6^{ACT}
y, w, eyFLP1/+; Act5C>y^{+}>Gal4 , UAS-GFP/UAS-ci^{RNAi} ; FRT82B, Tub-Gal80/FRT82B, UAS-Toll-6^{ACT}

Fig S4G y, w, eyFLP1/+; Act5C>y^{+}>Gal4 , UAS-GFP/TRE-DsRed; FRT82B, Tub-Gal80/FRT82B, UAS-Toll-6^{ACT}
y, w, eyFLP1/+; Act5C>y^{+}>Gal4 , UAS-GFP/TRE-DsRed, UAS-ci^{RNAi} ; FRT82B, Tub-Gal80/FRT82B, UAS-Toll-6^{ACT}

Fig S4I y, w, eyFLP1/+; Act5C>y^{+}>Gal4 , UAS-GFP/+; FRT82B, Tub-Gal80/UAS-yki$^{S168A.GFP}$, FRT82B, UAS-Toll-6^{ACT}
y, w, eyFLP1/UAS-bsk^{DN} ; Act5C>y^{+}>Gal4 , UAS-GFP/+; FRT82B, Tub-Gal80/UAS-yki$^{S168A.GFP}$, FRT82B, UAS-Toll-6^{ACT}
y, w, eyFLP1/+; Act5C>y^{+}>Gal4 , UAS-GFP/+; FRT82B, Tub-Gal80/UAS-yki$^{S168A.GFP}$, FRT82B, UAS-ci^{ACT}
y, w, eyFLP1/UAS-bsk^{DN} ; Act5C>y^{+}>Gal4 , UAS-GFP/+; FRT82B, Tub-Gal80/UAS-yki$^{S168A.GFP}$, FRT82B, UAS-ci^{ACT}
y, w, eyFLP1/+; Tub-QF2, QUAS-GFP, Act5C>y+>Gal4/+; FRT82B, Tub-QS, Tub-Gal80/QUAS-yki$^{S111A.S168A.S250A}$, FRT82B, scrib^{1}
y, w, eyFLP1/UAS-bsk^{DN} ; Tub-QF2, QUAS-GFP, Act5C>y+>Gal4/+; FRT82B, Tub-QS, Tub-Gal80/QUAS-yki$^{S111A.S168A.S250A}$, FRT82B, scrib^{1}

Fig S5.

Fig S5A y, w, eyFLP1/+; Tub-Gal80, FRT40A/FRT40A; Act5C>y^{+}>Gal4 , UAS-GFP/+
y, w, eyFLP1/+; Tub-Gal80, FRT40A/FRT40A; Act5C>y^{+}>Gal4 , UAS-GFP/UAS-AP-2α^{HA}

Fig S5B y, w, eyFLP1/+; Act5C>y^{+}>Gal4 , UAS-GFP/+; FRT82B, Tub-Gal80/UAS-AP-2α^{HA} , FRT82B, UAS-Toll-6$^{ACT.Flag}$

Fig S5C y, w, eyFLP1/+; Tub-Gal80, FRT40A/FRT40A; Act5C>y^{+}>Gal4 , UAS-GFP/+
y, w, eyFLP1/+; Tub-Gal80, FRT40A/AP-2α$^{40\text{-}31}$, FRT40A; Act5C>y^{+}>Gal4 , UAS-GFP/+
y, w, eyFLP1/+; Tub-Gal80, FRT40A/FRT40A; Act5C>y^{+}>Gal4 , UAS-GFP/UAS-AP-2α^{HA}

Fig S5F y, w, eyFLP1/+; Tub-Gal80, FRT40A/FRT40A; Act5C>y^{+}>Gal4, UAS-GFP/+
y, w, eyFLP1/+; Tub-Gal80, FRT40A/AP-2α$^{40\text{-}31}$, FRT40A; Act5C>y^{+}>Gal4, UAS-GFP/+

Fig S6.

Fig S6G y, w, eyFLP1/+; Tub-QF2, QUAS-GFP, Act5C>y+>Gal4/+; FRT82B, Tub-Gal80, Tub-QS/FRT82B
y, w, eyFLP1/+; Tub-QF2, QUAS-GFP, Act5C>y+>Gal4/+; FRT82B, Tub-Gal80, Tub-QS/QUAS-yki$^{S111A.S168A.S250A}$, FRT82B, scrib^{1}
y, w, eyFLP1/+; Tub-QF2, QUAS-GFP, Act5C>y+>Gal4/UAS-mib1$^{RNAi\#1}$; FRT82B, Tub-Gal80, Tub-QS/QUAS-yki$^{S111A.S168A.S250A}$, FRT82B, scrib^{1}

y, w, eyFLP1/+; Tub-QF2, QUAS-GFP, Act5C>y+>Gal4/UAS-mib1 $^{RNAi\#2}$ *; FRT82B, Tub-Gal80, Tub-QS/QUAS-yki* $^{S111A.S168A.S250A}$ *, FRT82B, scrib* ^{1}

Fig S7.

Fig S7C

y, w, eyFLP1/+; Tub-QF2, QUAS-GFP, Act5C>y+>Gal4/+; FRT82B, Tub-QS, Tub-Gal80/QUAS-yki $^{S111A.S168A.S250A}$ *, FRT82B, scrib* ^{1}

y, w, eyFLP1/+; Tub-QF2, QUAS-GFP, Act5C>y+>Gal4/UAS-pvf1 ^{RNAi} *; FRT82B, Tub-QS, Tub-Gal80/QUAS-yki* $^{S111A.S168A.S250A}$ *, FRT82B, scrib* ^{1}

y, w, eyFLP1/+; Tub-QF2, QUAS-GFP, Act5C>y+>Gal4/UAS-pvf2 ^{RNAi} *; FRT82B, Tub-QS, Tub-Gal80/QUAS-yki* $^{S111A.S168A.S250A}$ *, FRT82B, scrib* ^{1}

y, w, eyFLP1/+; Tub-QF2, QUAS-GFP, Act5C>y+>Gal4/UAS-pvf3 ^{RNAi} *; FRT82B, Tub-QS, Tub-Gal80/QUAS-yki* $^{S111A.S168A.S250A}$ *, FRT82B, scrib* ^{1}

Fig S7F

y, w, eyFLP1/+; Tub-QF2, QUAS-GFP, Act5C>y+>Gal4/+; FRT82B, Tub-QS, Tub-Gal80/FRT82B

y, w, eyFLP1/+; Tub-QF2, QUAS-GFP, Act5C>y+>Gal4/+; FRT82B, Tub-QS, Tub-Gal80/QUAS-yki $^{S111A.S168A.S250A}$ *, FRT82B*

y, w, eyFLP1/+; Tub-QF2, QUAS-GFP, Act5C>y+>Gal4/+; FRT82B, Tub-QS, Tub-Gal80/QUAS-yki $^{S111A.S168A.S250A}$ *, FRT82B, scrib* ^{1}

y, w, eyFLP1/+; Tub-QF2, QUAS-GFP, Act5C>y+>Gal4/UAS-upd1 ^{RNAi} *; FRT82B, Tub-QS, Tub-Gal80/QUAS-yki* $^{S111A.S168A.S250A}$ *, FRT82B, scrib* ^{1}

y, w, eyFLP1/+; Tub-QF2, QUAS-GFP, Act5C>y+>Gal4/UAS-upd2 ^{RNAi} *; FRT82B, Tub-QS, Tub-Gal80/QUAS-yki* $^{S111A.S168A.S250A}$ *, FRT82B, scrib* ^{1}

y, w, eyFLP1/+; Tub-QF2, QUAS-GFP, Act5C>y+>Gal4/UAS-upd3 ^{RNAi} *; FRT82B, Tub-QS, Tub-Gal80/QUAS-yki* $^{S111A.S168A.S250A}$ *, FRT82B, scrib* ^{1}

Fig S7I

y, w, eyFLP1/+; Tub-QF2, QUAS-GFP, Act5C>y+>Gal4/+; FRT82B, Tub-QS, Tub-Gal80/QUAS-yki $^{S111A.S168A.S250A}$ *, FRT82B, scrib* ^{1}

y, w, eyFLP1/+; Tub-QF2, QUAS-GFP, Act5C>y+>Gal4/UAS-dome ^{DN} *; FRT82B, Tub-QS, Tub-Gal80/QUAS-yki* $^{S111A.S168A.S250A}$ *, FRT82B, scrib* ^{1}

Fig S7N

y, w, eyFLP1/+; Tub-QF2, QUAS-GFP/+; r4-Gal4, FRT82B, Tub-QS/QUAS-yki $^{S111A.S168A.S250A}$ *, FRT82B, scrib* ^{1}

y, w, eyFLP1/+; Tub-QF2, QUAS-GFP/UAS-pvr ^{DN} *; r4-Gal4, FRT82B, Tub-QS/QUAS-yki* $^{S111A.S168A.S250A}$ *, FRT82B, scrib* ^{1}

y, w, eyFLP1/+; Tub-QF2, QUAS-GFP/UAS-pvr ^{RNAi} *; r4-Gal4, FRT82B, Tub-QS/QUAS-yki* $^{S111A.S168A.S250A}$ *, FRT82B, scrib* ^{1}
