# Supplementary figures and images for: Adipose tissue-secreted Spz5 promotes distal tumor progression via Toll-6-mediated Hh pathway activation in Drosophila (part 1 of 5)
Source: EMBO J. 2025 Jun 23;44(15):4301–30. doi: 10.1038/s44318-025-00489-y (PMC12317064; doi:10.1038/s44318-025-00489-y)

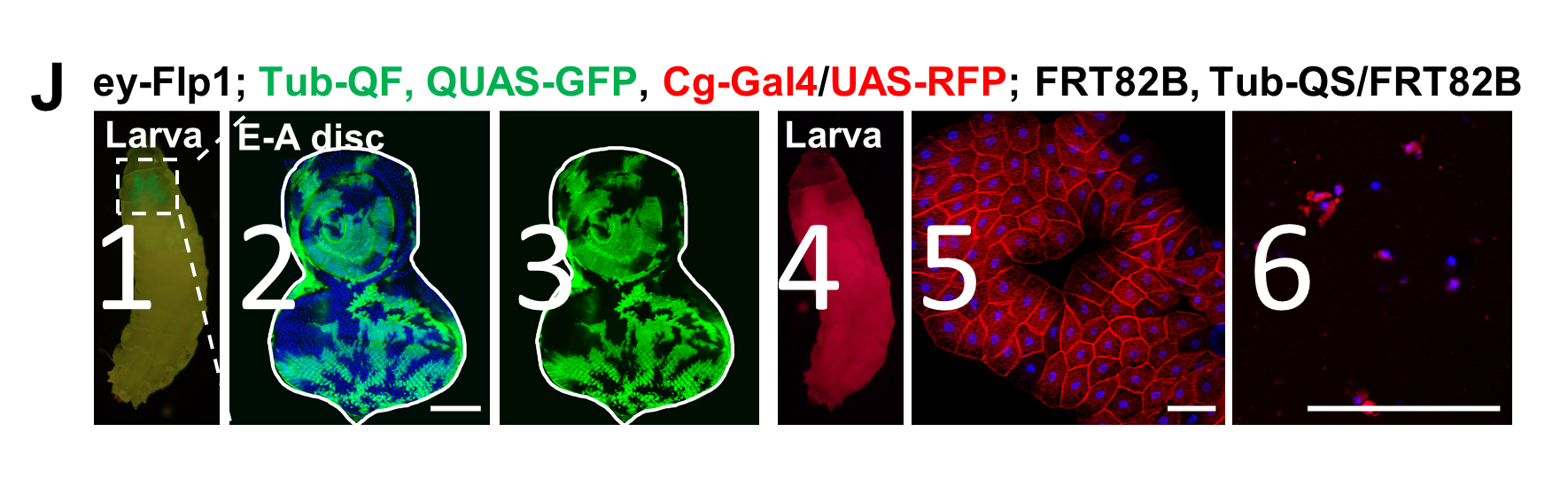

Supplement: Supplementary file 5 — Source data Fig. 1 [file 44318_2025_489_MOESM5_ESM.zip › Figure 1J/0 paper Figure 1J with provided image sequence.tif]

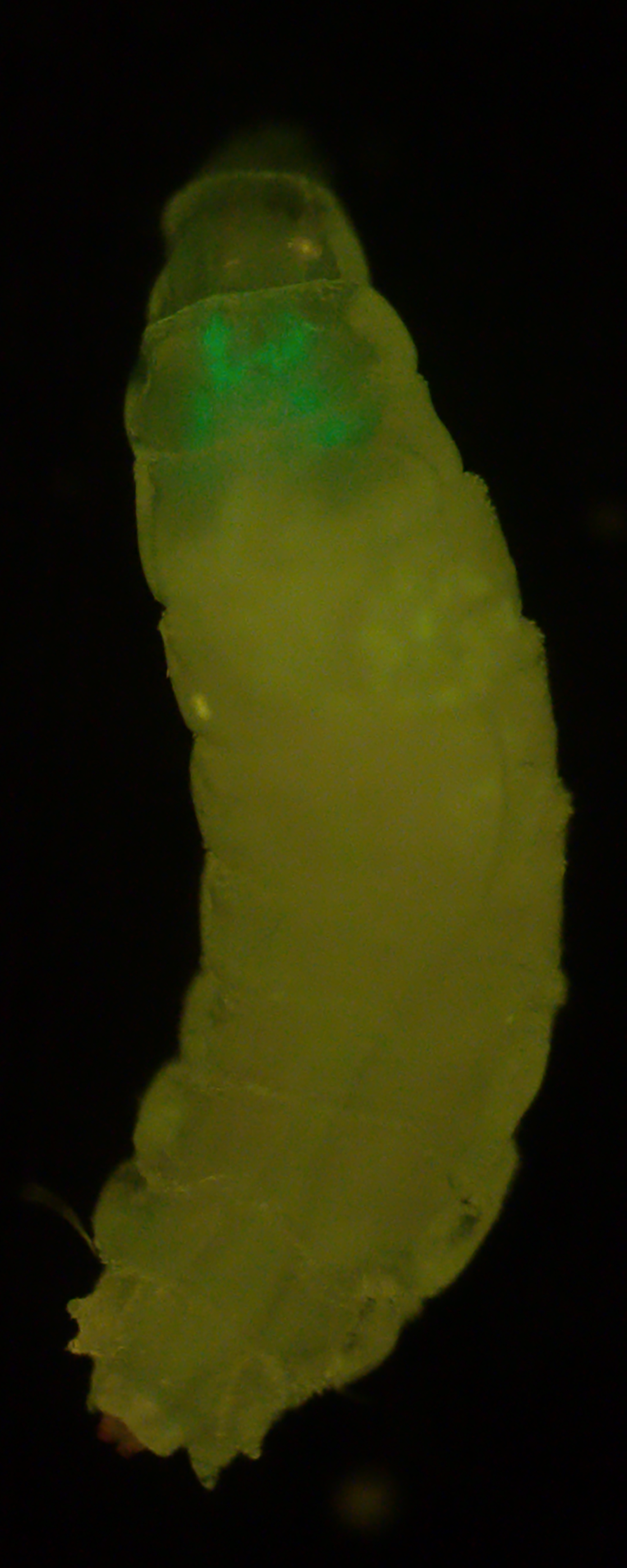

Supplement: Supplementary file 5 — Source data Fig. 1 [file 44318_2025_489_MOESM5_ESM.zip › Figure 1J/1-1 rotated and cut image.tif]

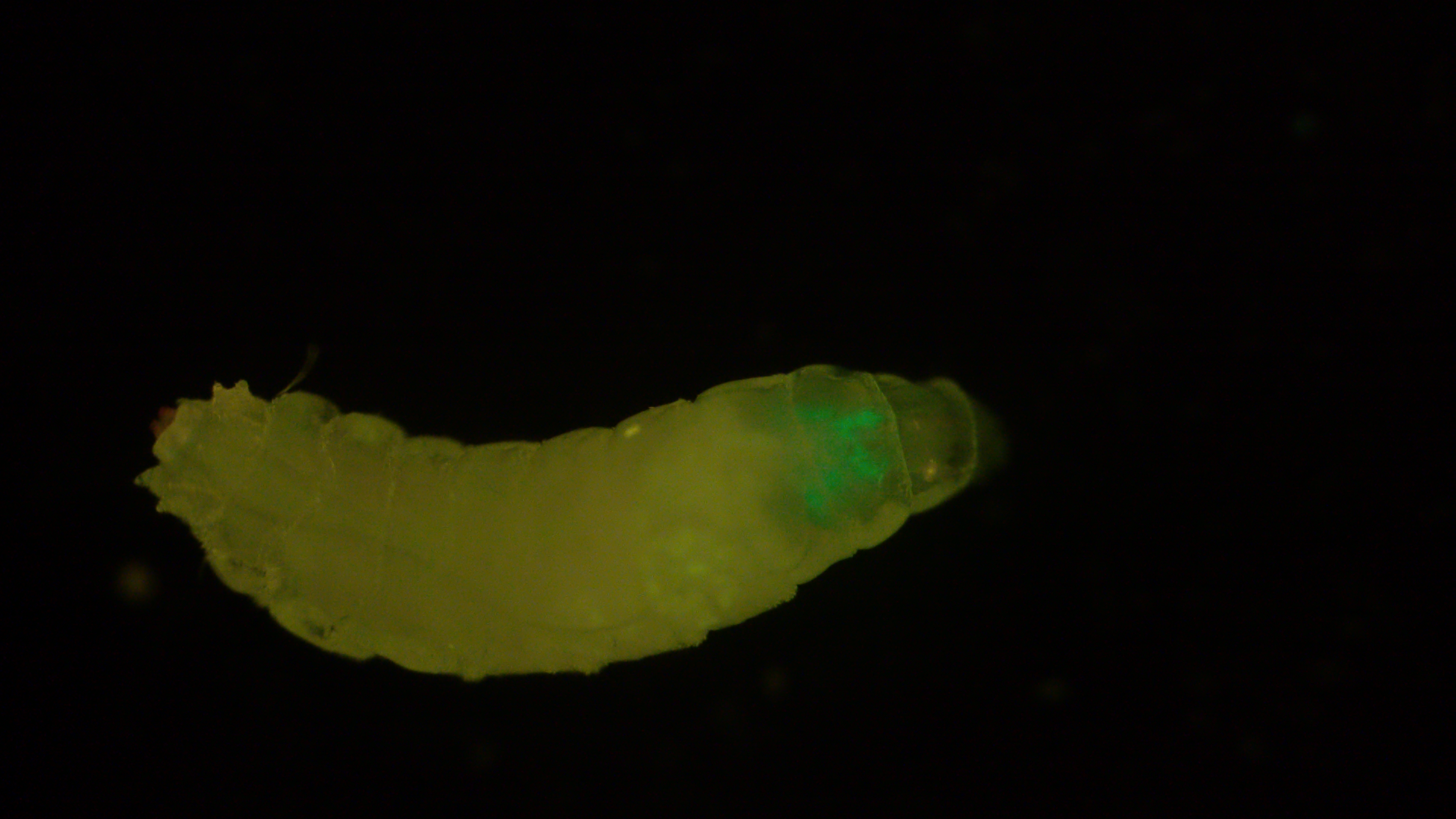

Supplement: Supplementary file 5 — Source data Fig. 1 [file 44318_2025_489_MOESM5_ESM.zip › Figure 1J/1-2 original image.tif]

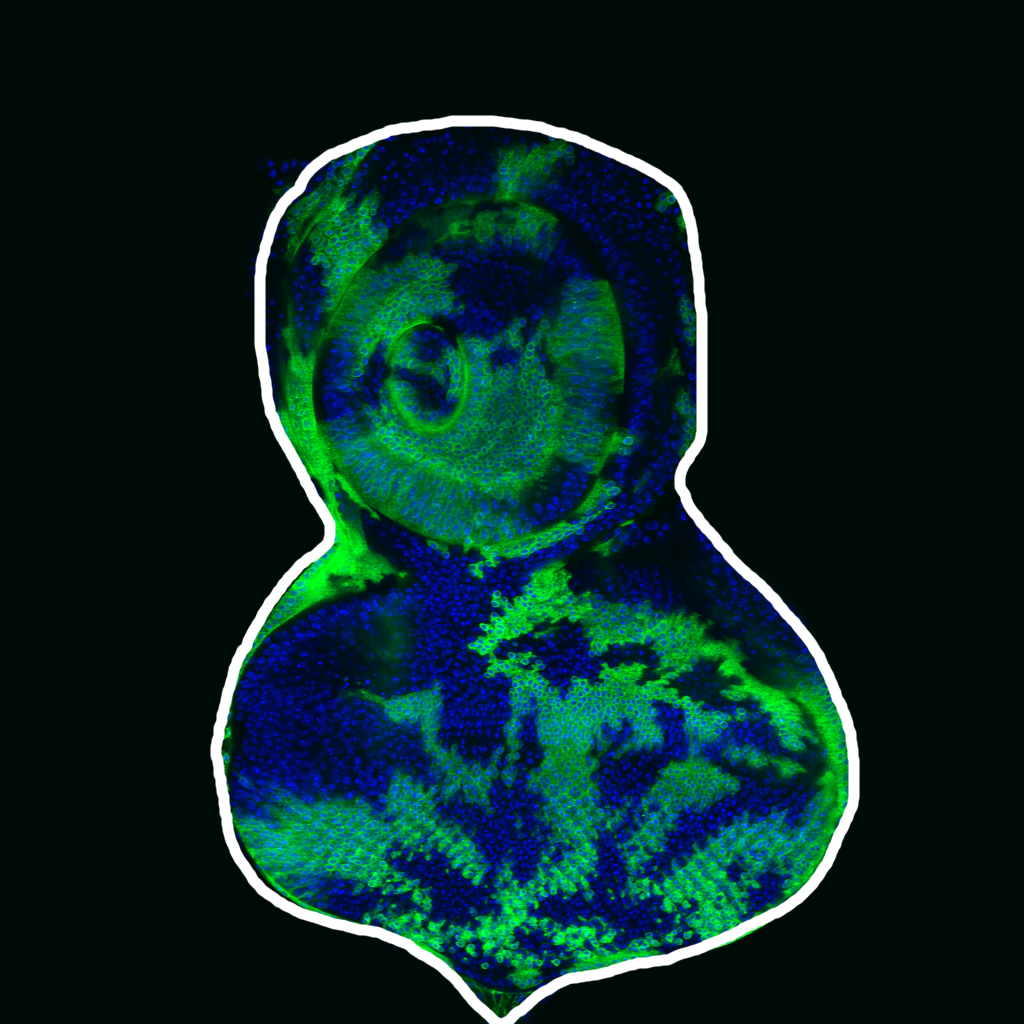

Supplement: Supplementary file 5 — Source data Fig. 1 [file 44318_2025_489_MOESM5_ESM.zip › Figure 1J/2-1 rotated and cut image with border line.tif]

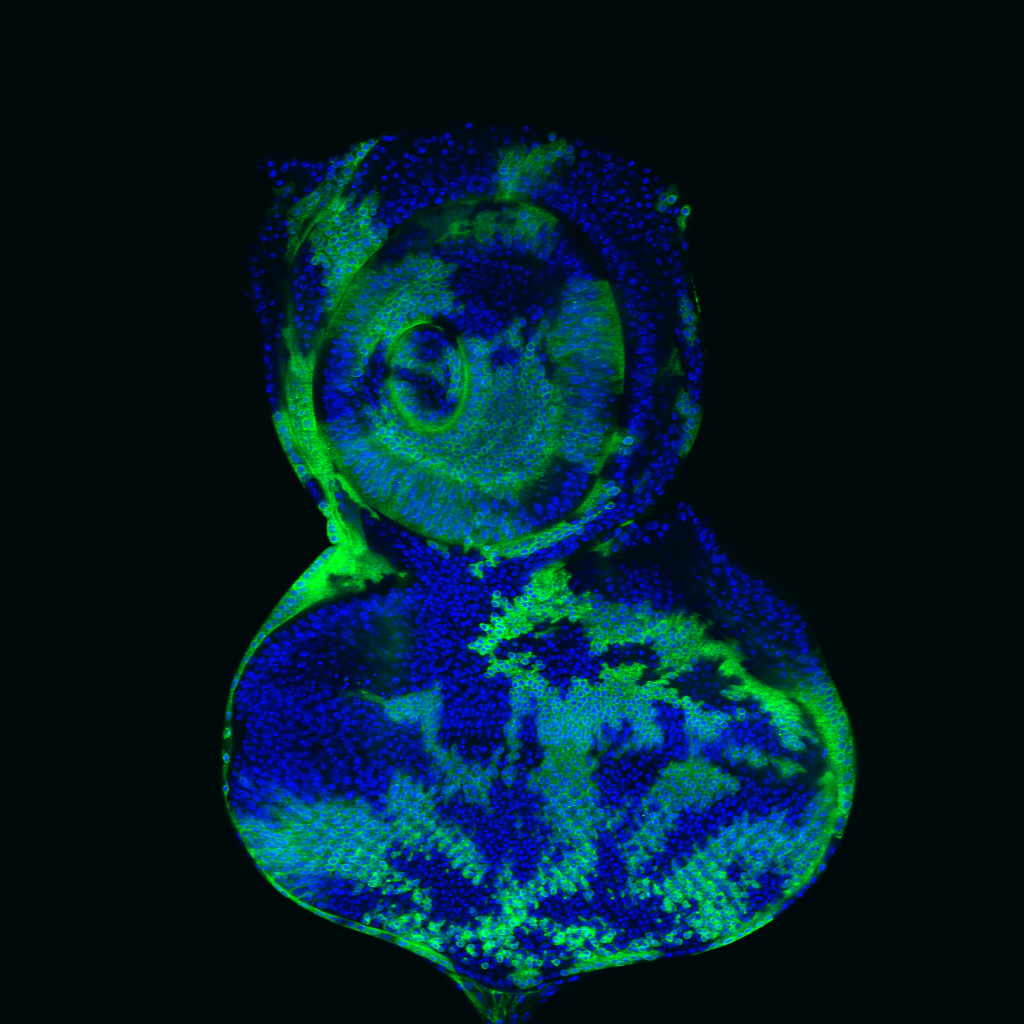

Supplement: Supplementary file 5 — Source data Fig. 1 [file 44318_2025_489_MOESM5_ESM.zip › Figure 1J/2-2 original image.tif]

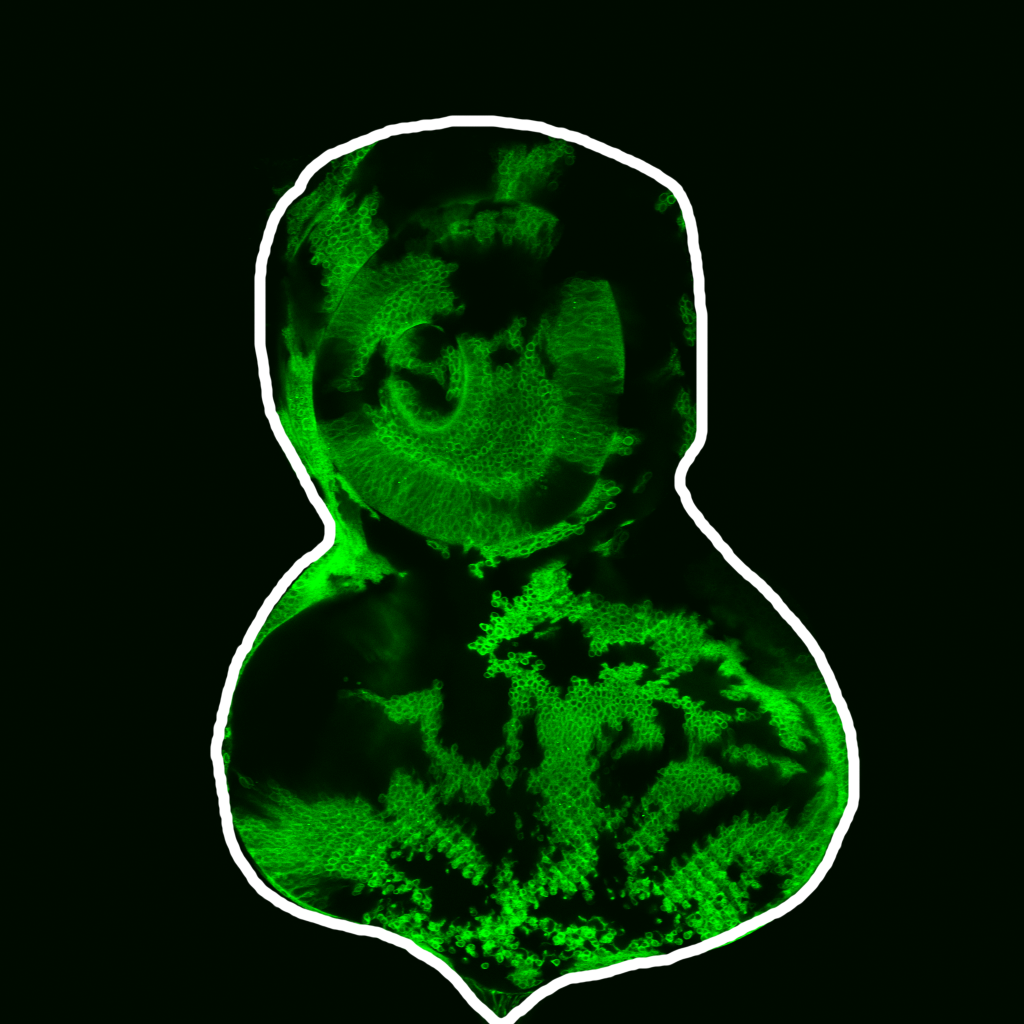

Supplement: Supplementary file 5 — Source data Fig. 1 [file 44318_2025_489_MOESM5_ESM.zip › Figure 1J/3-1 rotated and cut image with border line.tif]

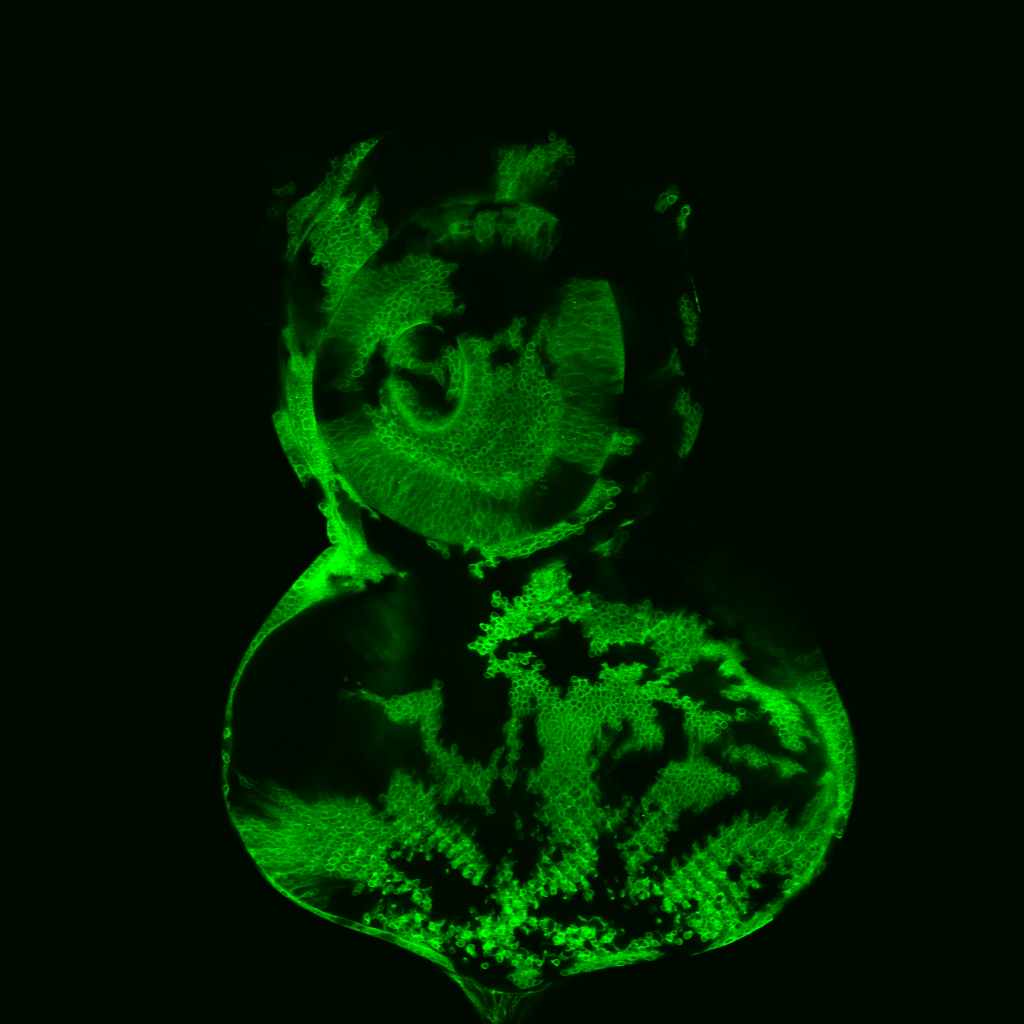

Supplement: Supplementary file 5 — Source data Fig. 1 [file 44318_2025_489_MOESM5_ESM.zip › Figure 1J/3-2 original image.tif]

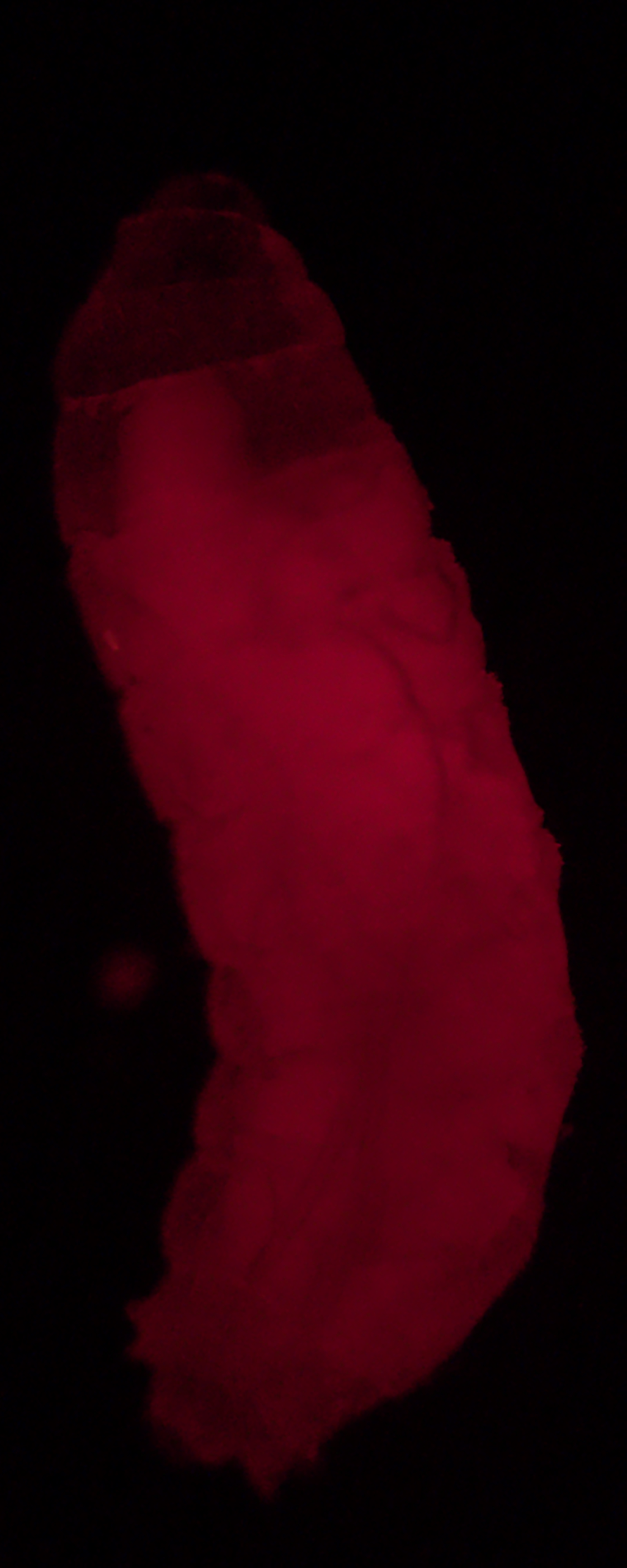

Supplement: Supplementary file 5 — Source data Fig. 1 [file 44318_2025_489_MOESM5_ESM.zip › Figure 1J/4-1 rotated and cut image.tif]

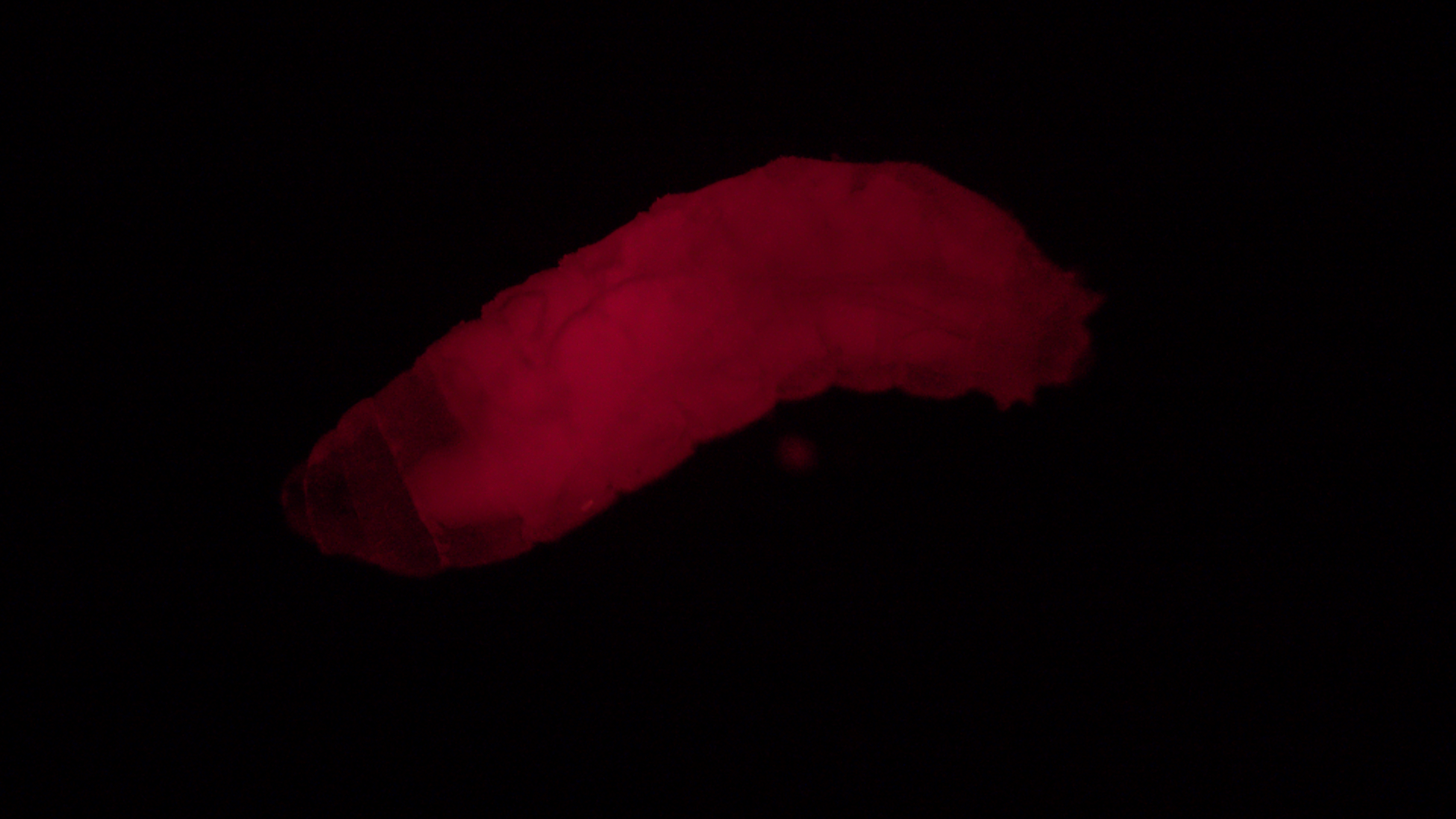

Supplement: Supplementary file 5 — Source data Fig. 1 [file 44318_2025_489_MOESM5_ESM.zip › Figure 1J/4-2 original image.tif]

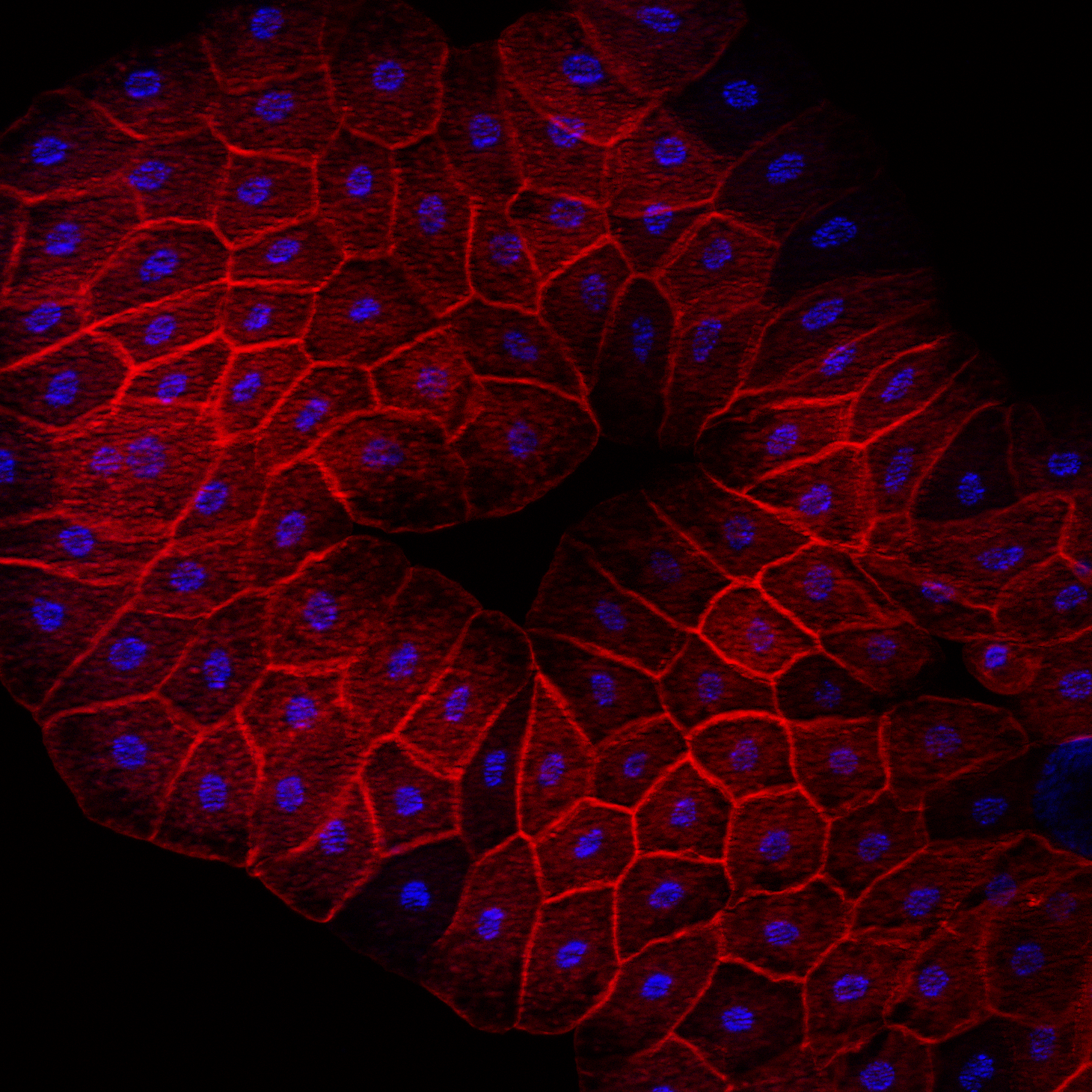

Supplement: Supplementary file 5 — Source data Fig. 1 [file 44318_2025_489_MOESM5_ESM.zip › Figure 1J/5-1 rotated and cut image.tif]

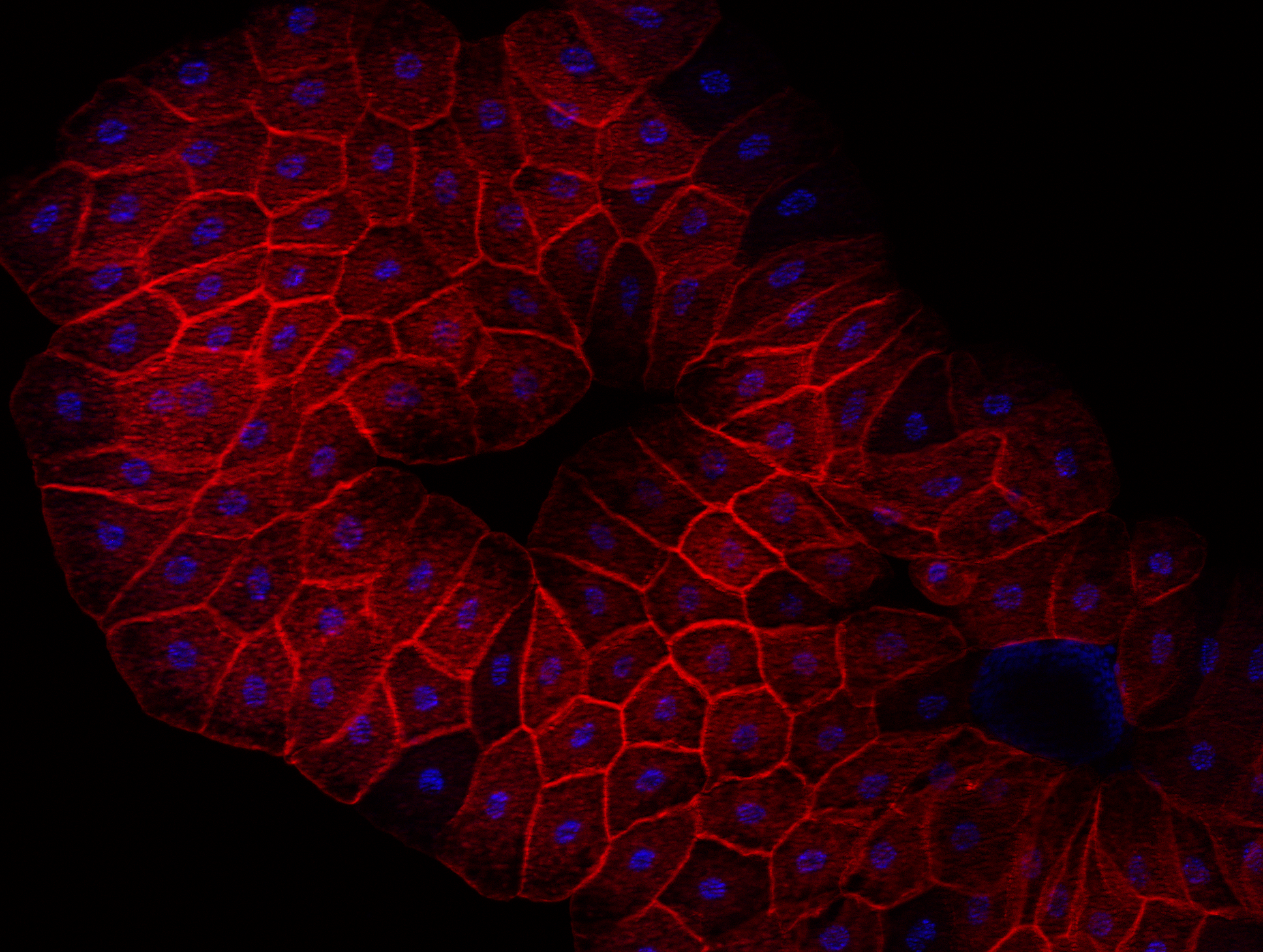

Supplement: Supplementary file 5 — Source data Fig. 1 [file 44318_2025_489_MOESM5_ESM.zip › Figure 1J/5-2 original image.tif]

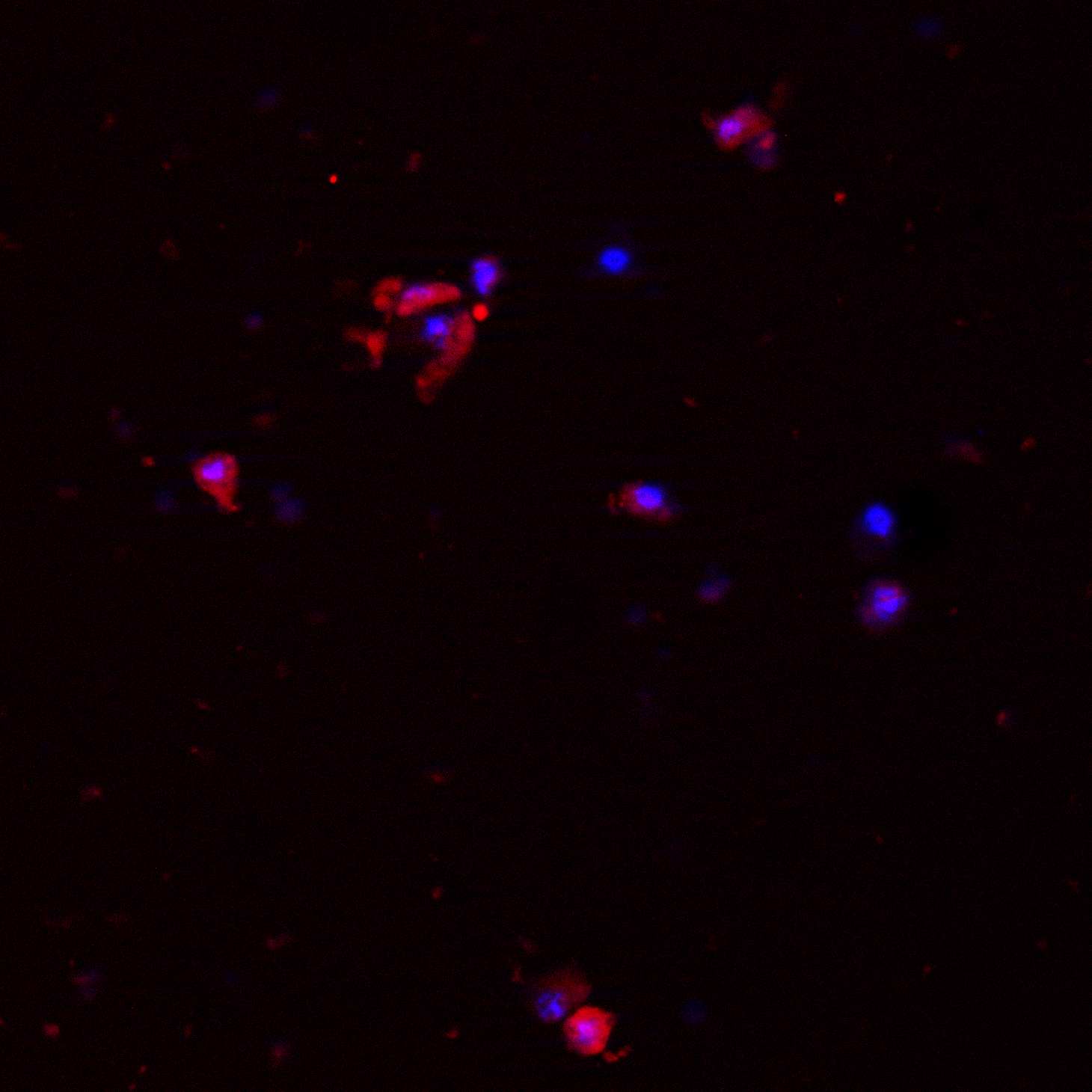

Supplement: Supplementary file 5 — Source data Fig. 1 [file 44318_2025_489_MOESM5_ESM.zip › Figure 1J/6-1 rotated and cut image.tif]

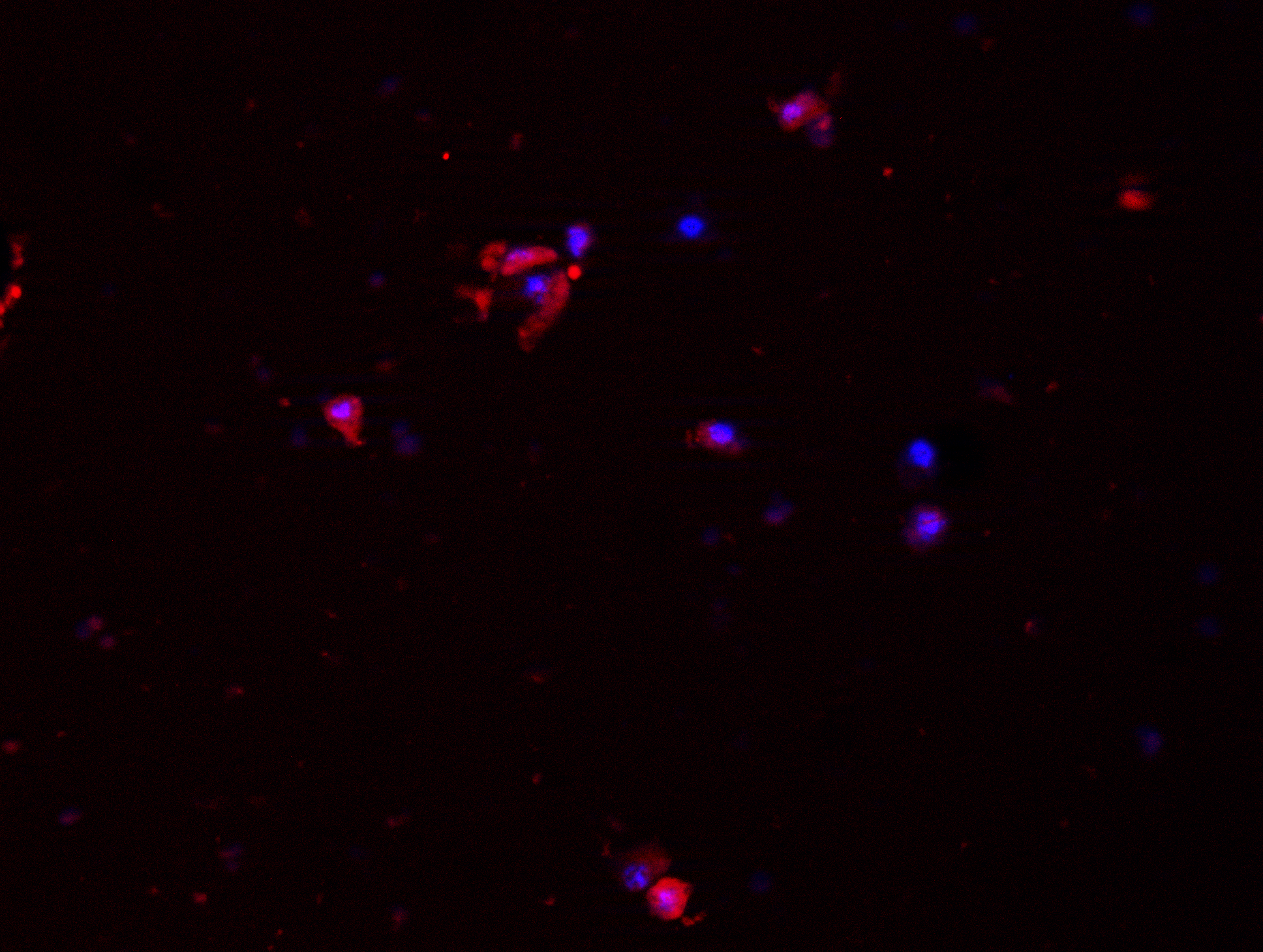

Supplement: Supplementary file 5 — Source data Fig. 1 [file 44318_2025_489_MOESM5_ESM.zip › Figure 1J/6-2 original image.tif]

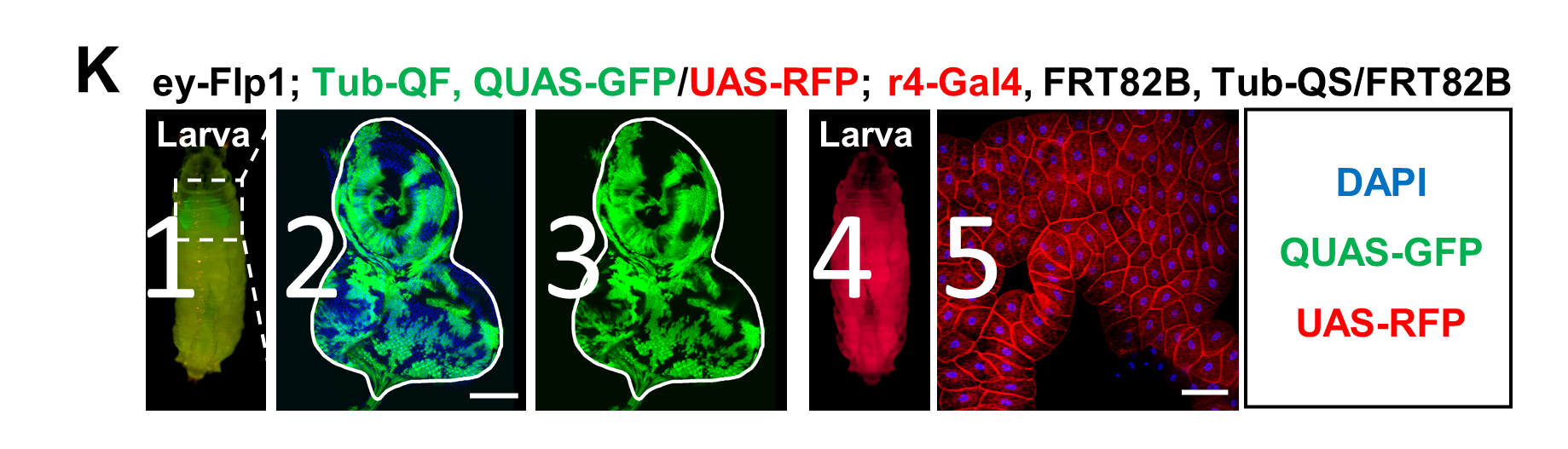

Supplement: Supplementary file 5 — Source data Fig. 1 [file 44318_2025_489_MOESM5_ESM.zip › Figure 1K/0 paper Figure 1K with provided image sequence.tif]

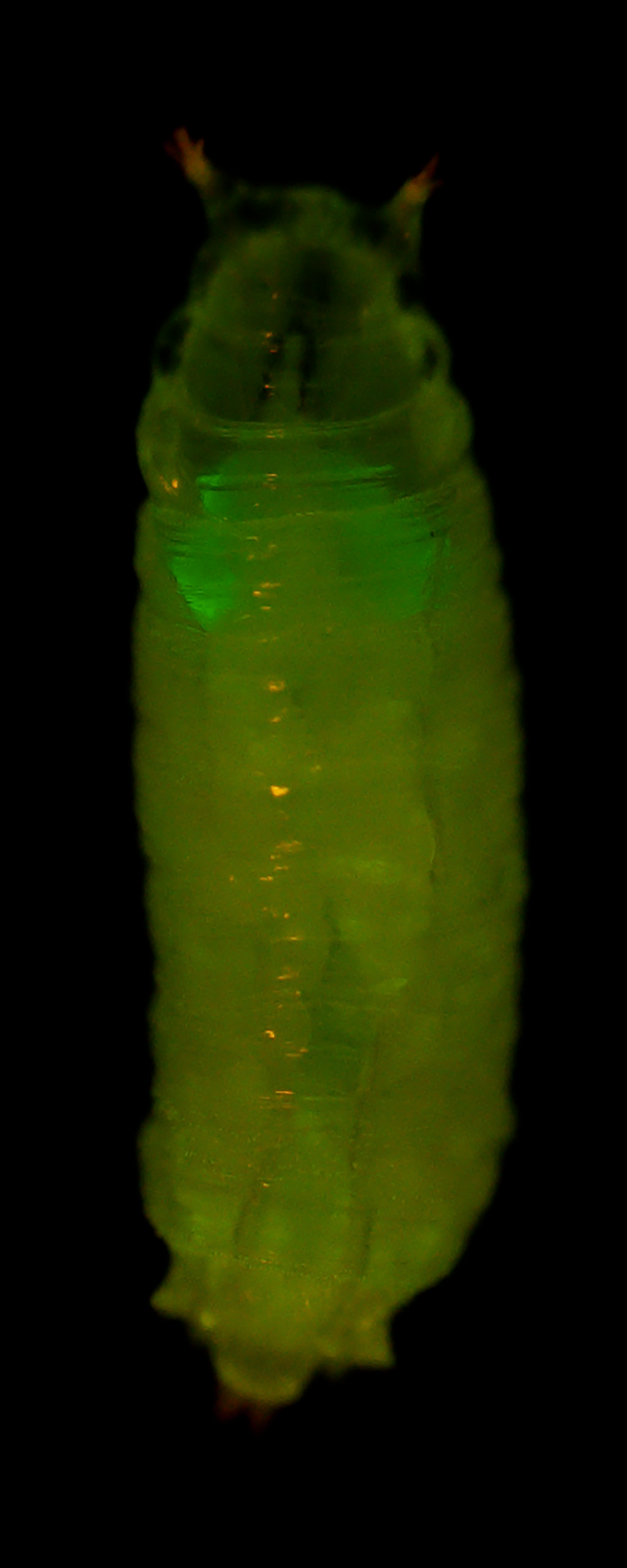

Supplement: Supplementary file 5 — Source data Fig. 1 [file 44318_2025_489_MOESM5_ESM.zip › Figure 1K/1-1 rotated and cut image.tif]

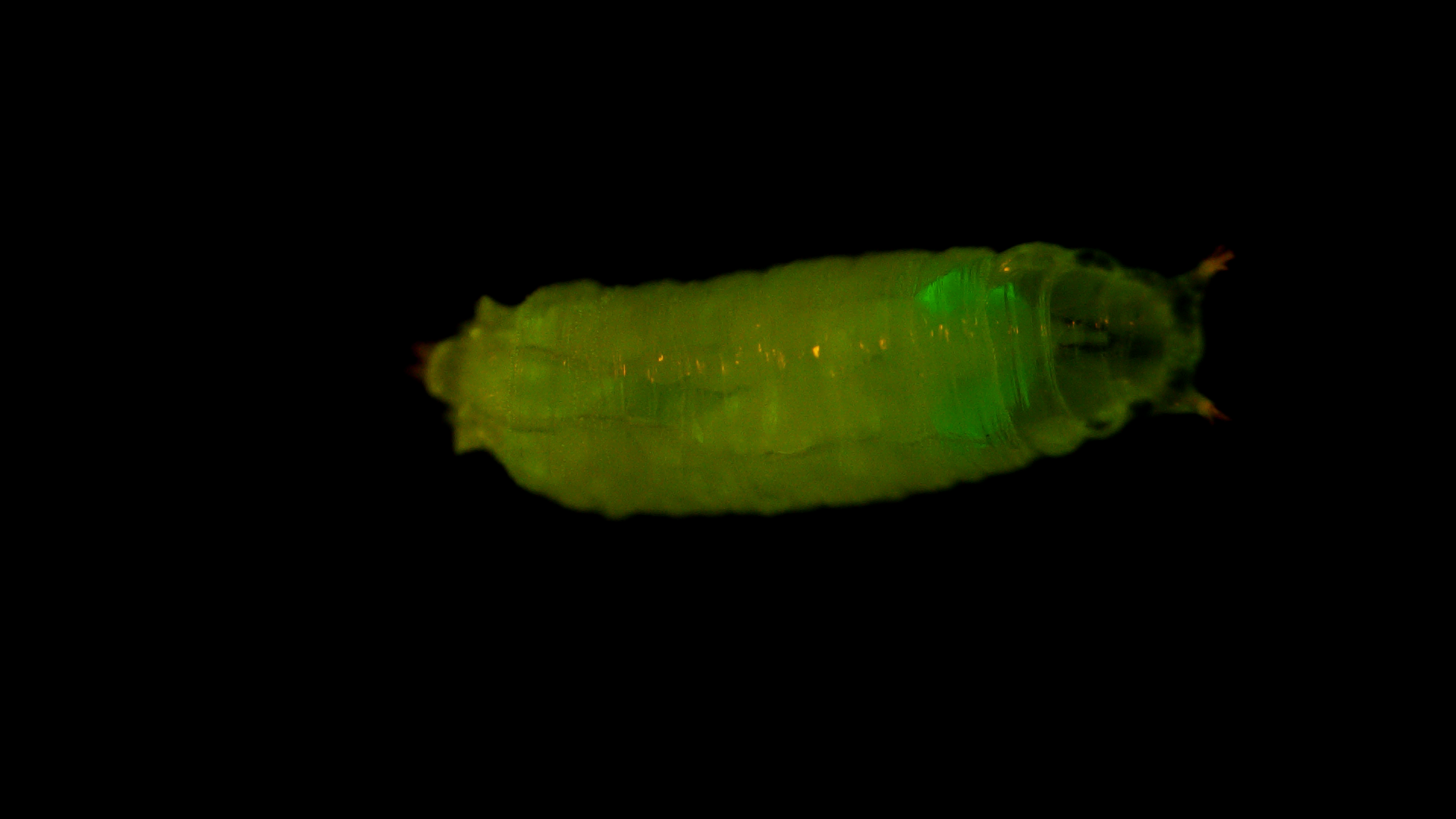

Supplement: Supplementary file 5 — Source data Fig. 1 [file 44318_2025_489_MOESM5_ESM.zip › Figure 1K/1-2 original image.tif]

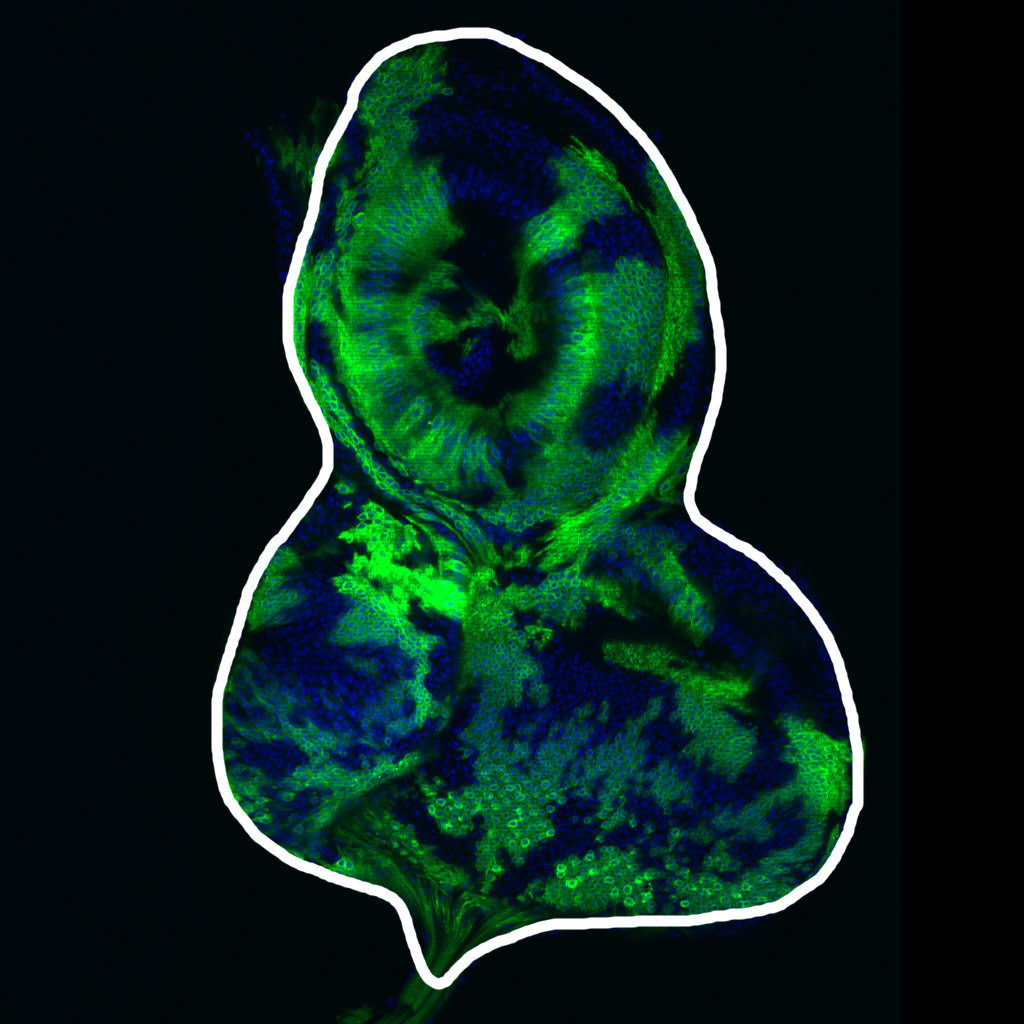

Supplement: Supplementary file 5 — Source data Fig. 1 [file 44318_2025_489_MOESM5_ESM.zip › Figure 1K/2-1 rotated and cut image with border line.tif]

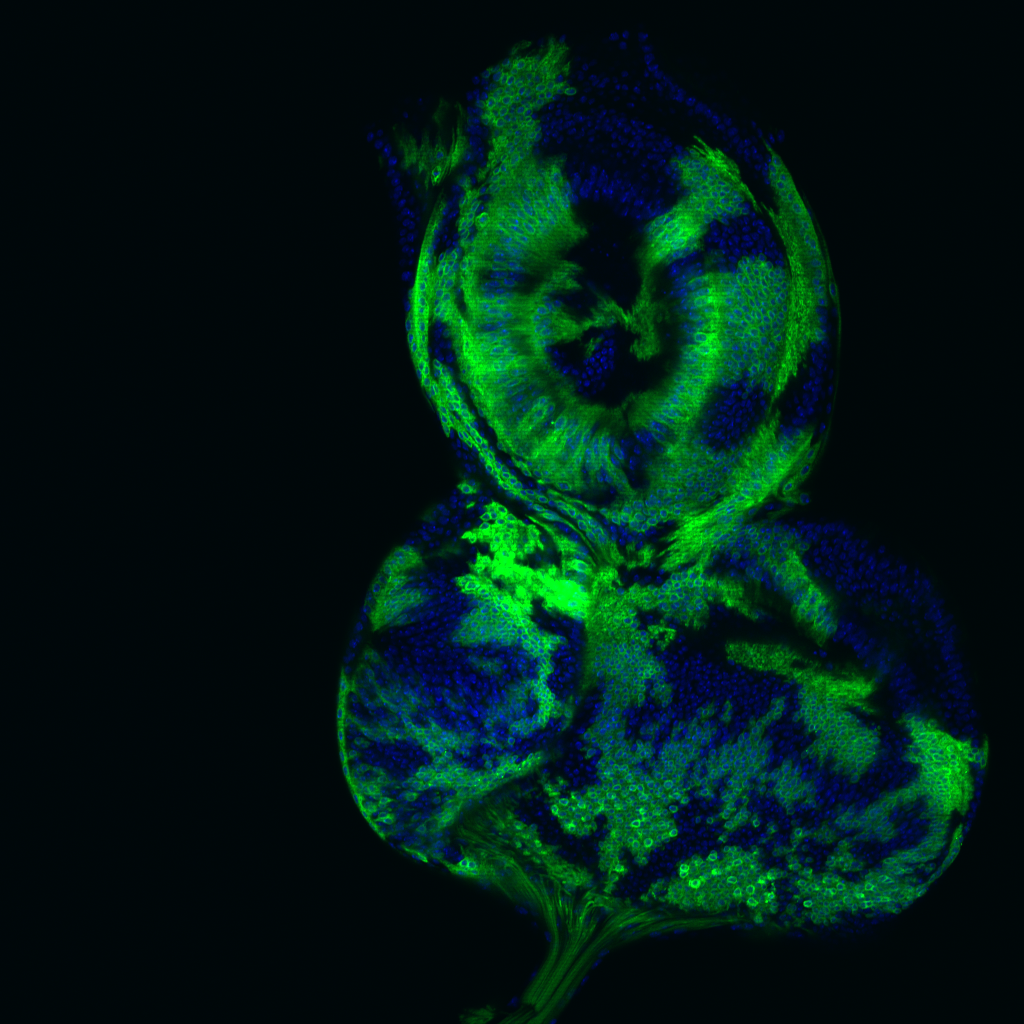

Supplement: Supplementary file 5 — Source data Fig. 1 [file 44318_2025_489_MOESM5_ESM.zip › Figure 1K/2-2 original image.tif]

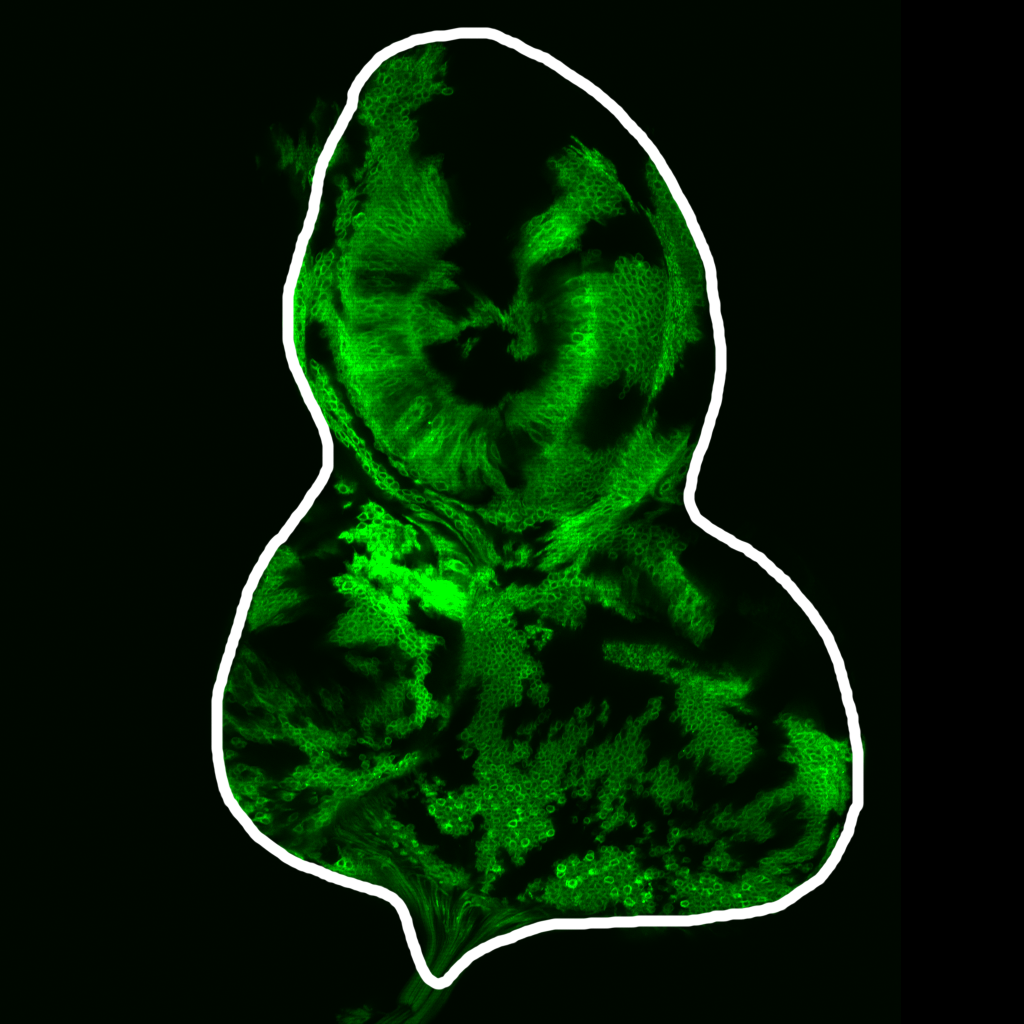

Supplement: Supplementary file 5 — Source data Fig. 1 [file 44318_2025_489_MOESM5_ESM.zip › Figure 1K/3-1 rotated and cut image with border line.tif]

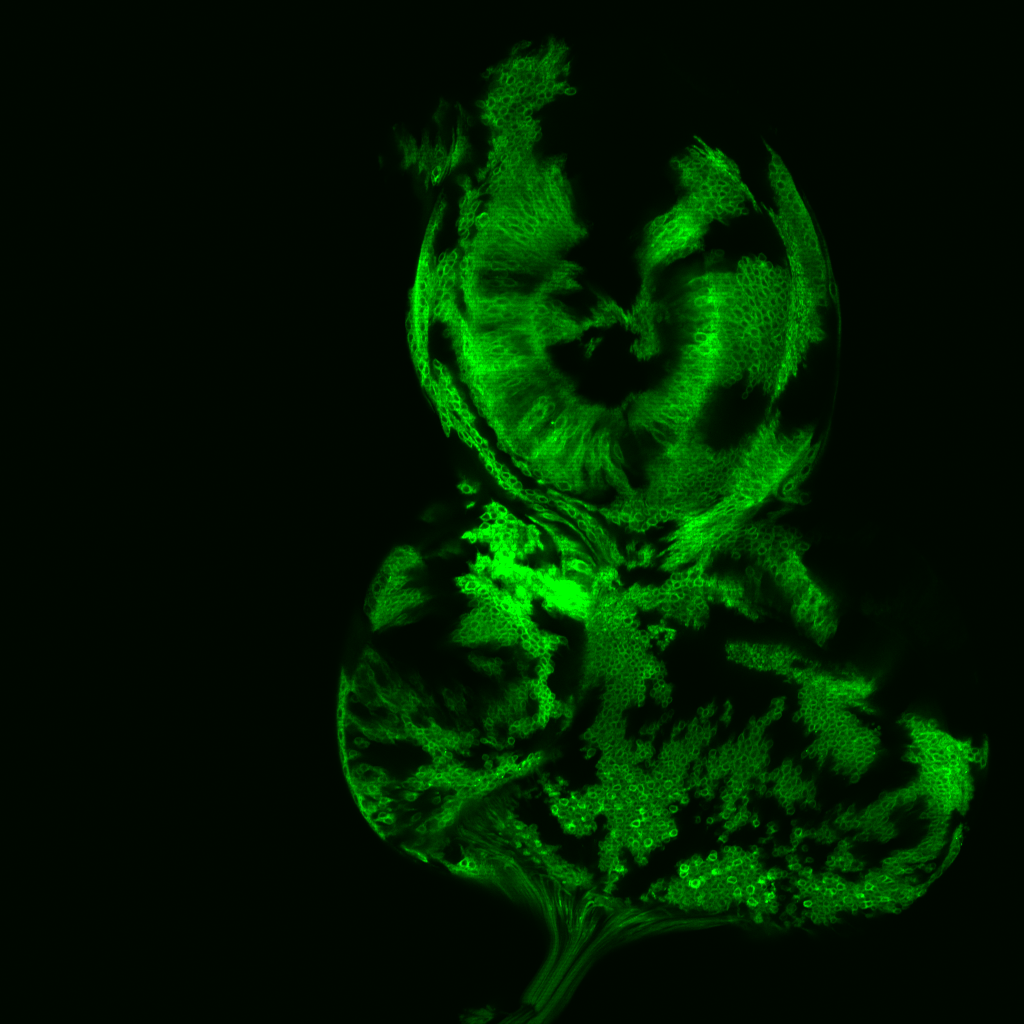

Supplement: Supplementary file 5 — Source data Fig. 1 [file 44318_2025_489_MOESM5_ESM.zip › Figure 1K/3-2 original image.tif]

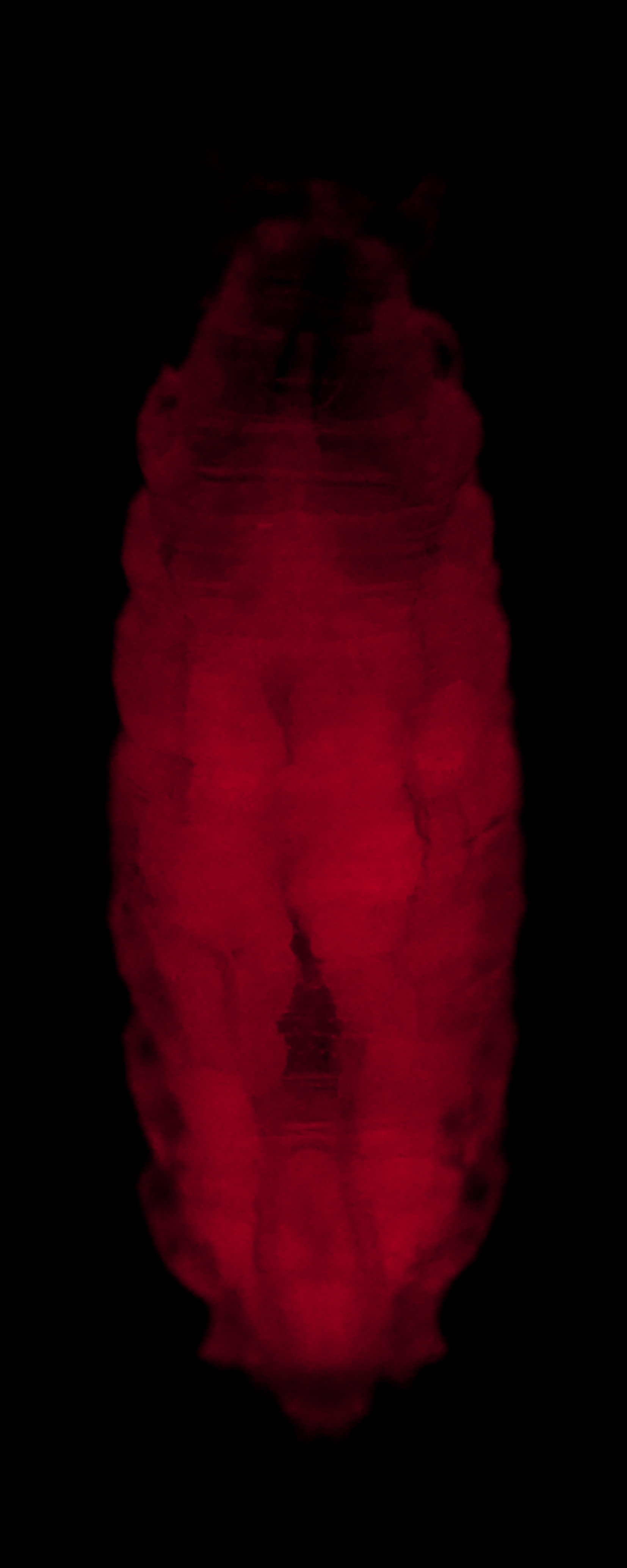

Supplement: Supplementary file 5 — Source data Fig. 1 [file 44318_2025_489_MOESM5_ESM.zip › Figure 1K/4-1 rotated and cut image.tif]

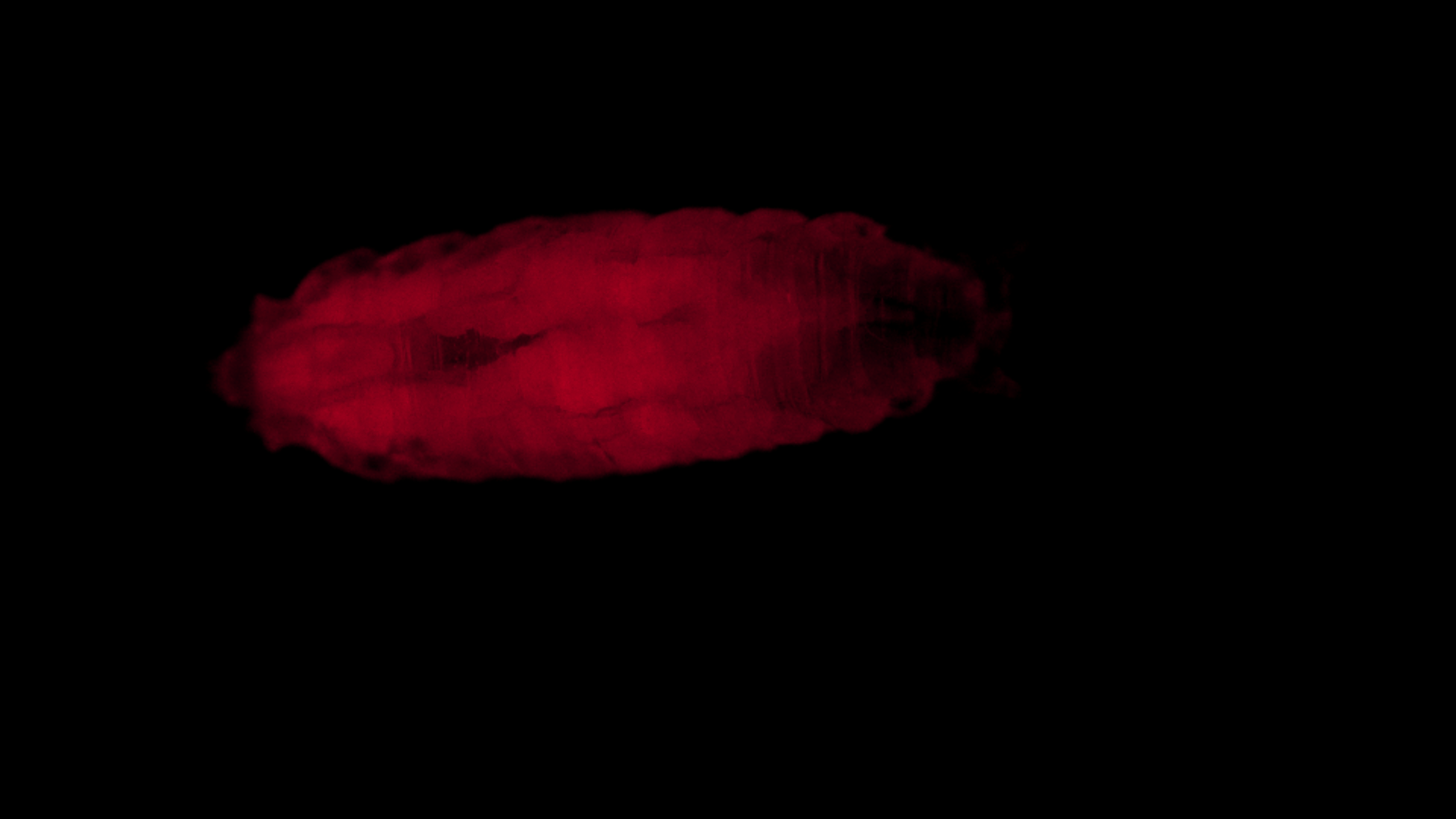

Supplement: Supplementary file 5 — Source data Fig. 1 [file 44318_2025_489_MOESM5_ESM.zip › Figure 1K/4-2 original image.tif]

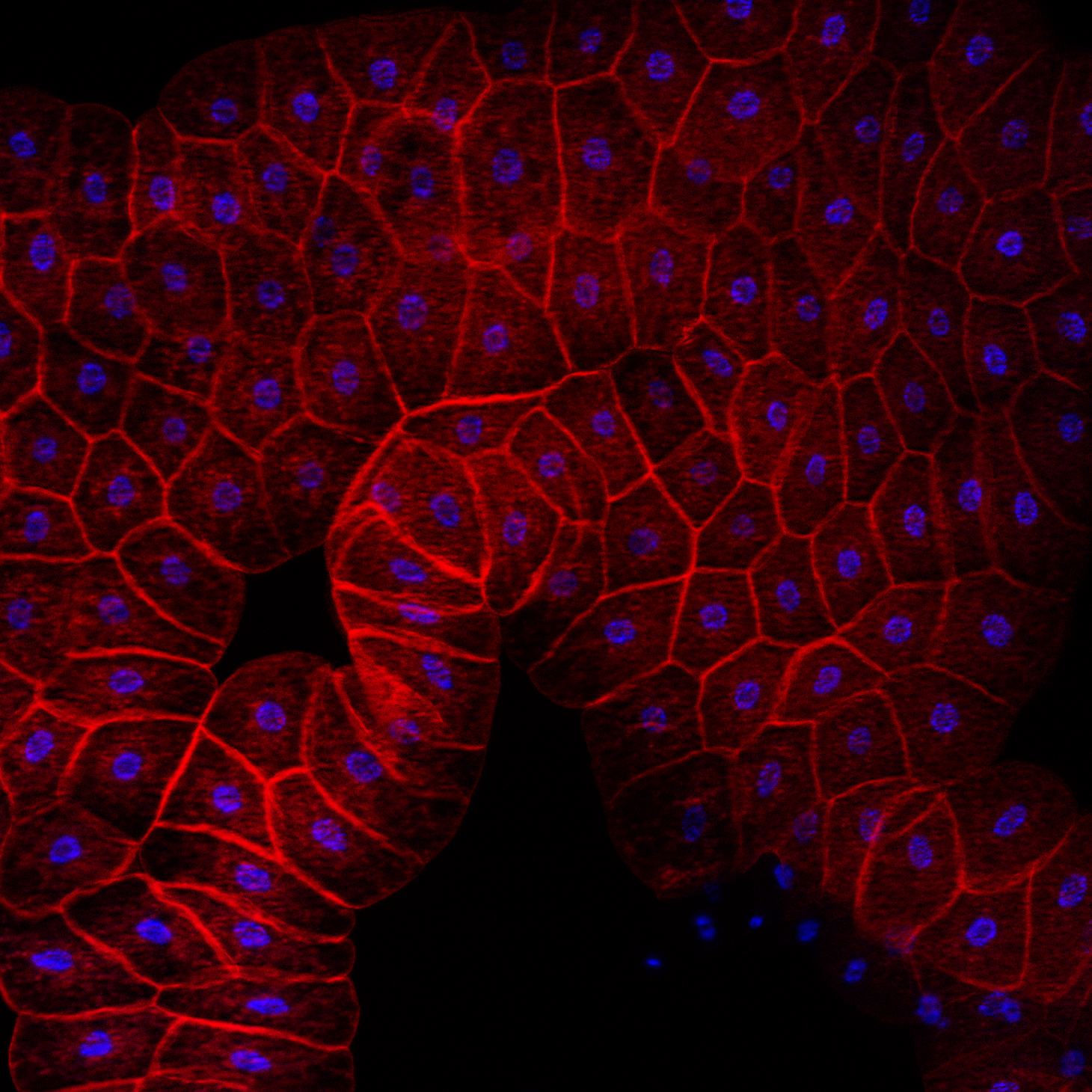

Supplement: Supplementary file 5 — Source data Fig. 1 [file 44318_2025_489_MOESM5_ESM.zip › Figure 1K/5-1 rotated and cut image.tif]

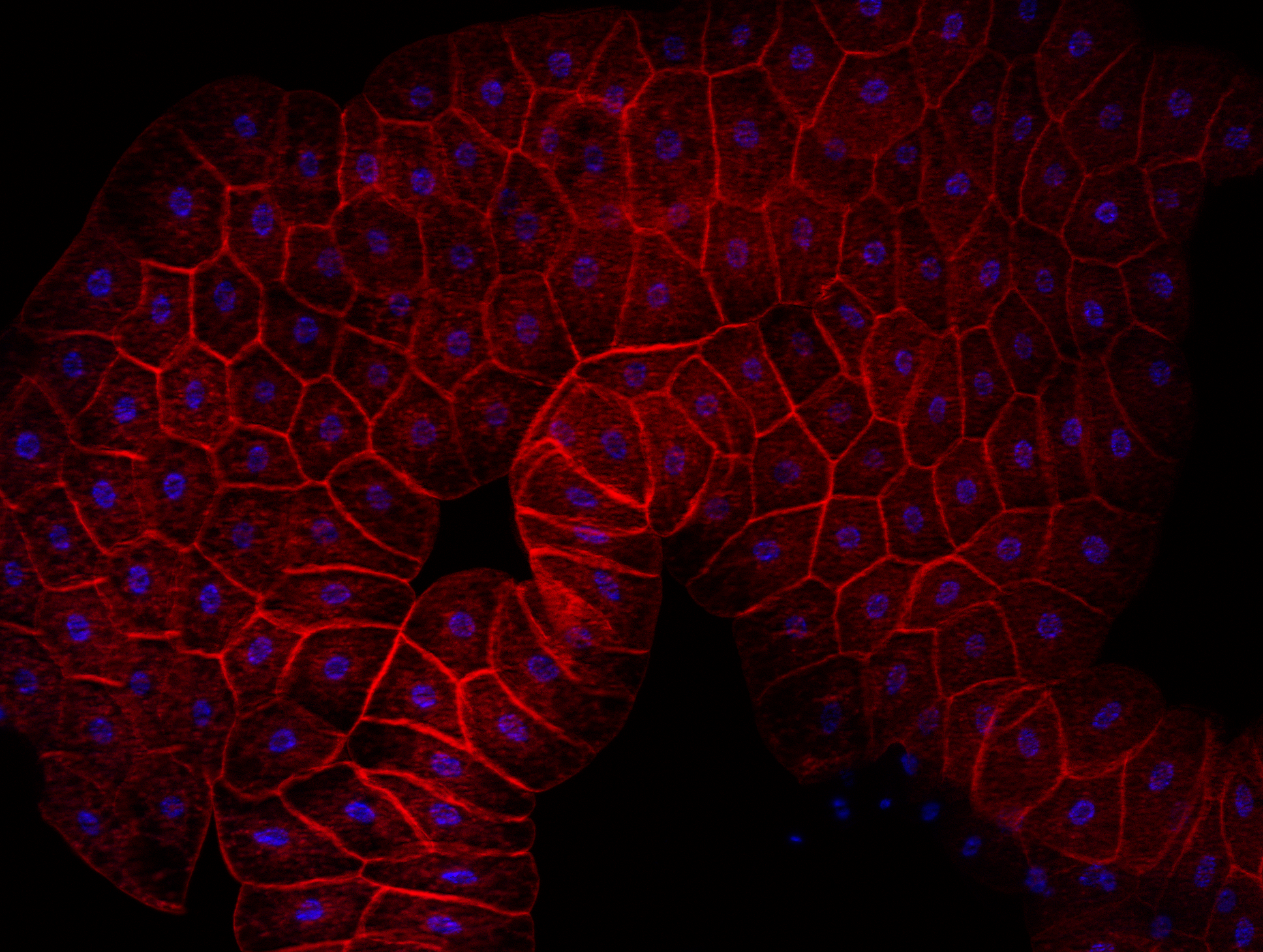

Supplement: Supplementary file 5 — Source data Fig. 1 [file 44318_2025_489_MOESM5_ESM.zip › Figure 1K/5-2 original image.tif]

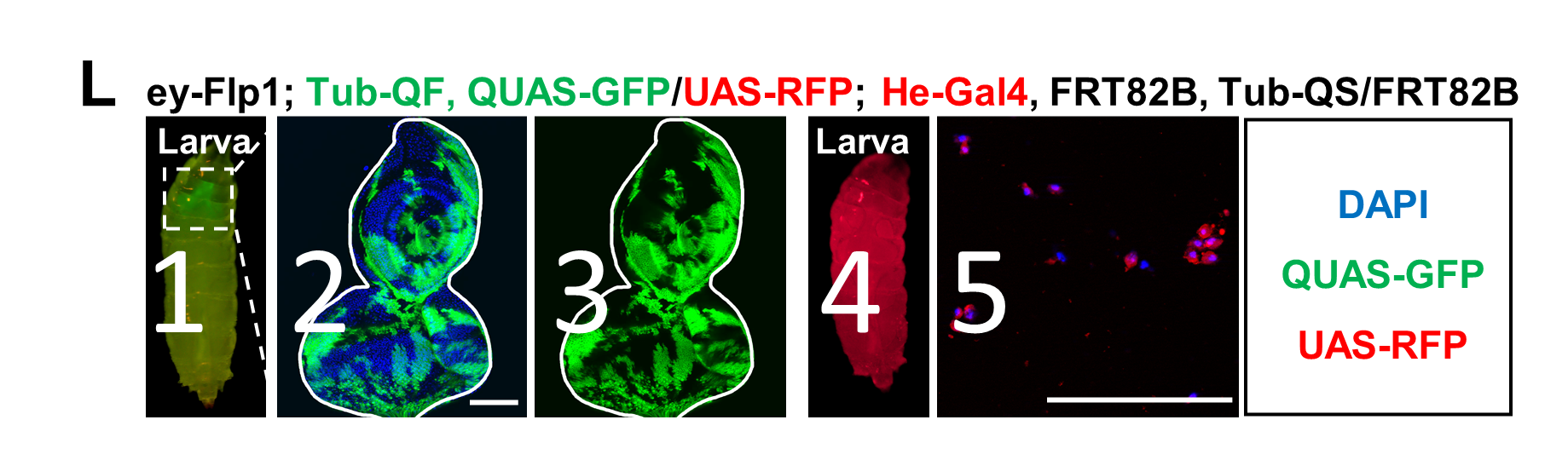

Supplement: Supplementary file 5 — Source data Fig. 1 [file 44318_2025_489_MOESM5_ESM.zip › Figure 1L/0 paper Figure 1L with provided image sequence.tif]

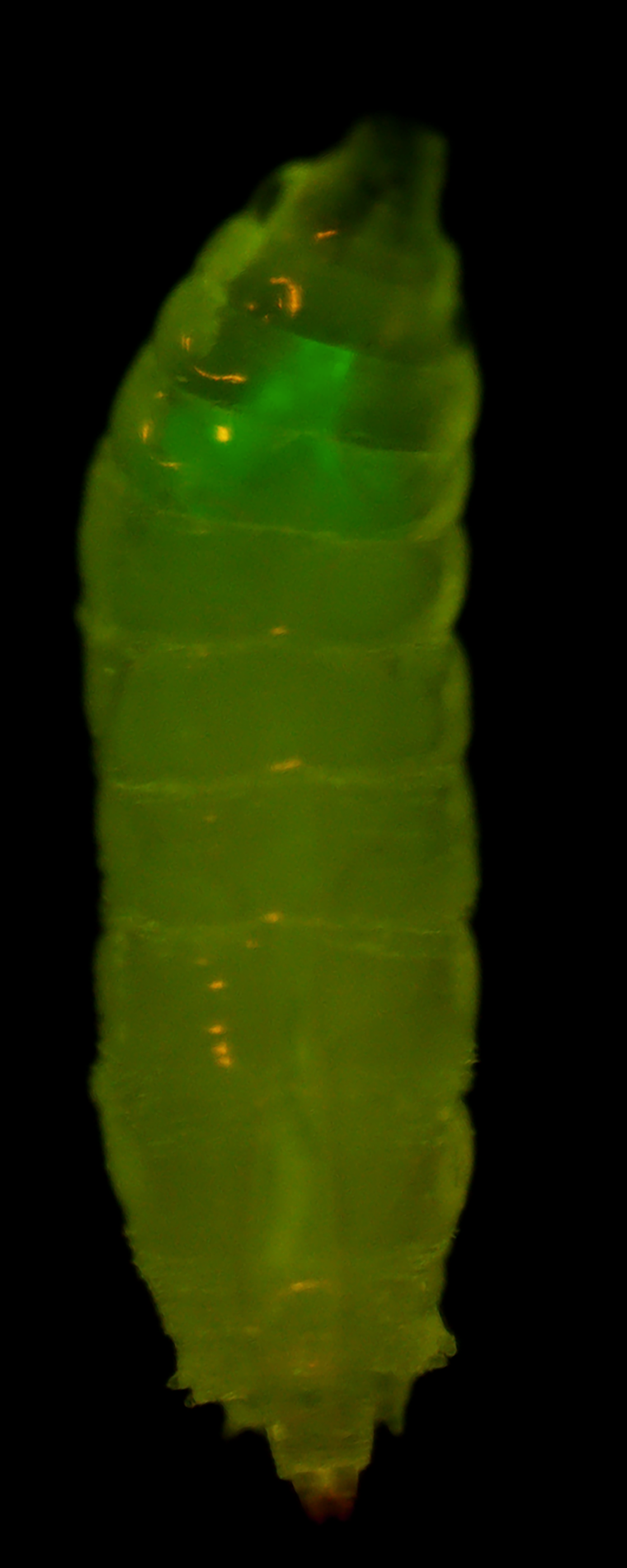

Supplement: Supplementary file 5 — Source data Fig. 1 [file 44318_2025_489_MOESM5_ESM.zip › Figure 1L/1-1 rotated and cut image.tif]

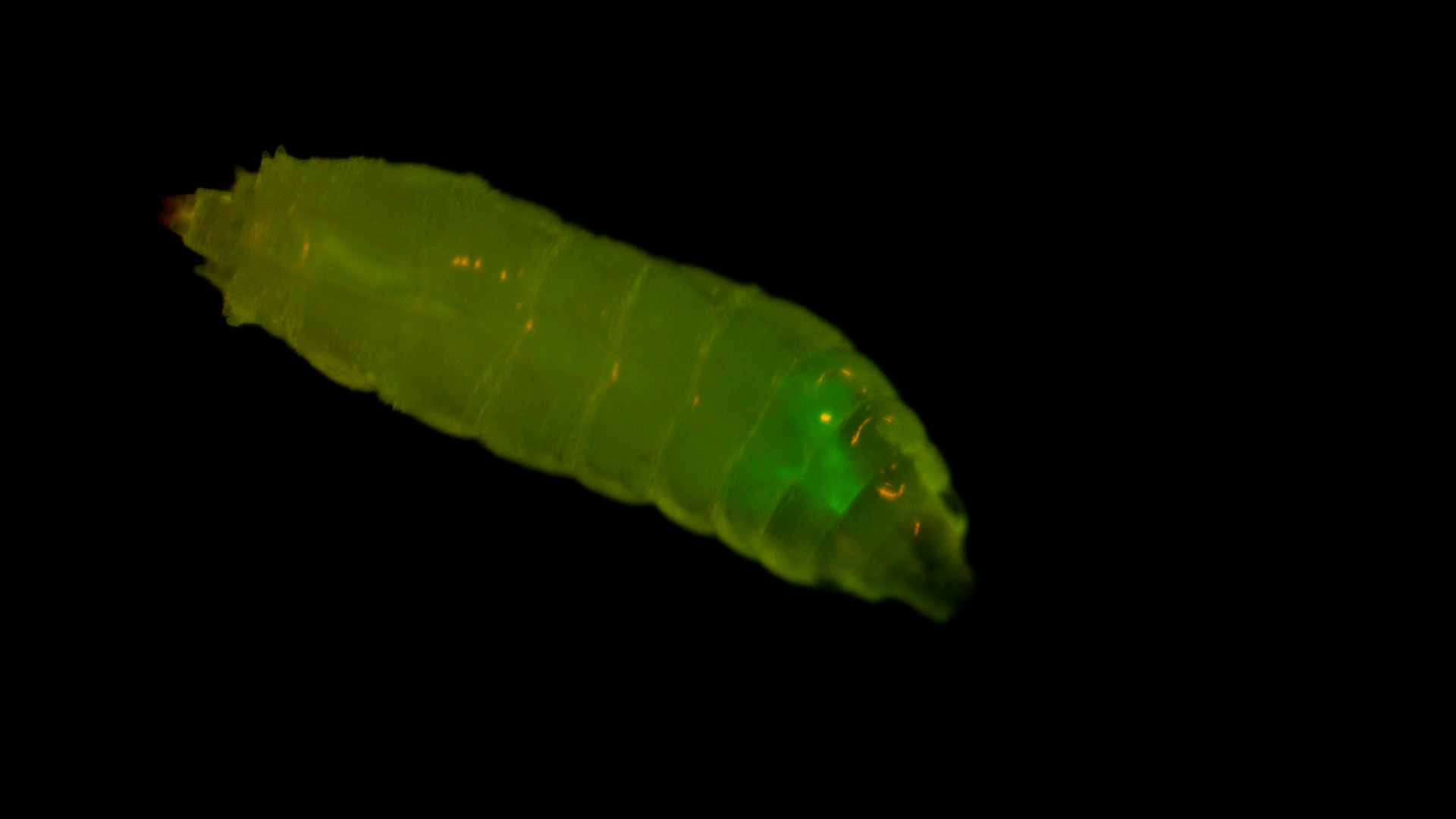

Supplement: Supplementary file 5 — Source data Fig. 1 [file 44318_2025_489_MOESM5_ESM.zip › Figure 1L/1-2 original image.tif]

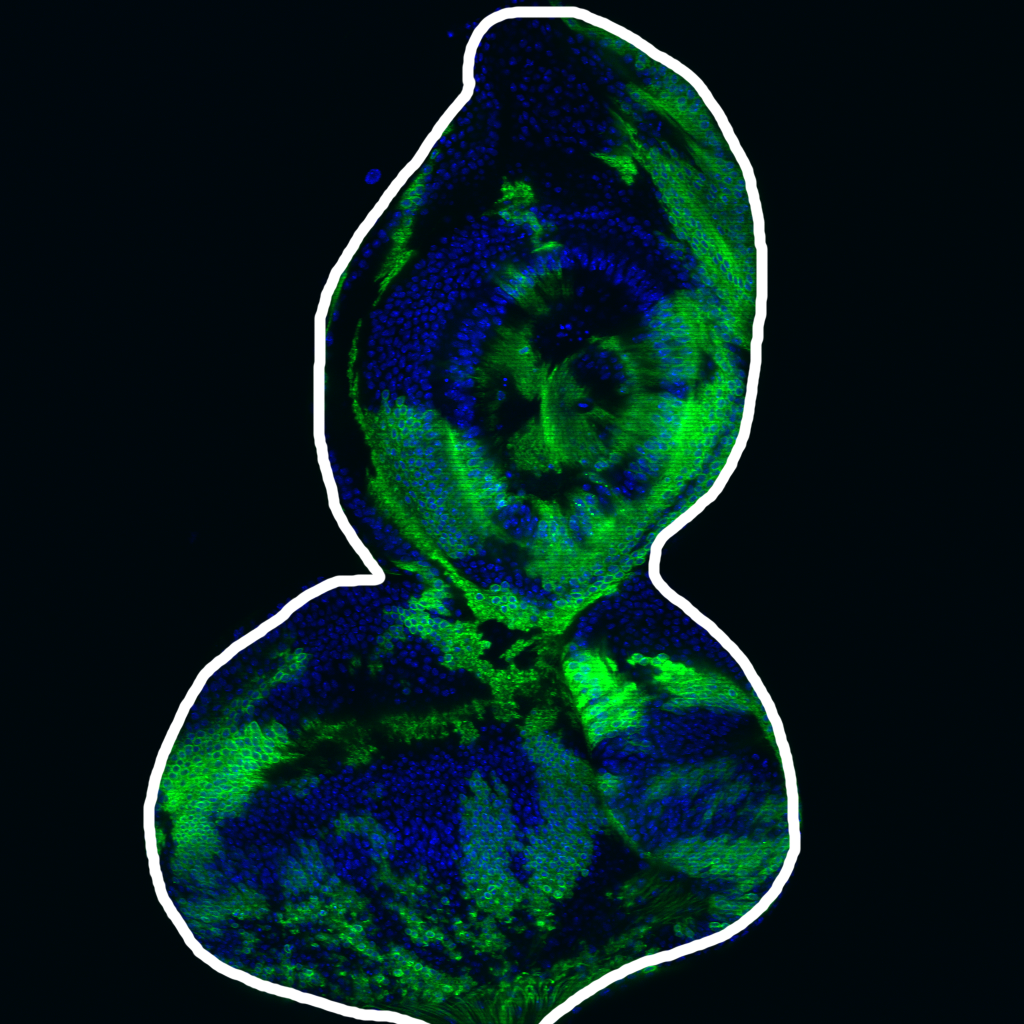

Supplement: Supplementary file 5 — Source data Fig. 1 [file 44318_2025_489_MOESM5_ESM.zip › Figure 1L/2-1 rotated and cut image with border line.tif]

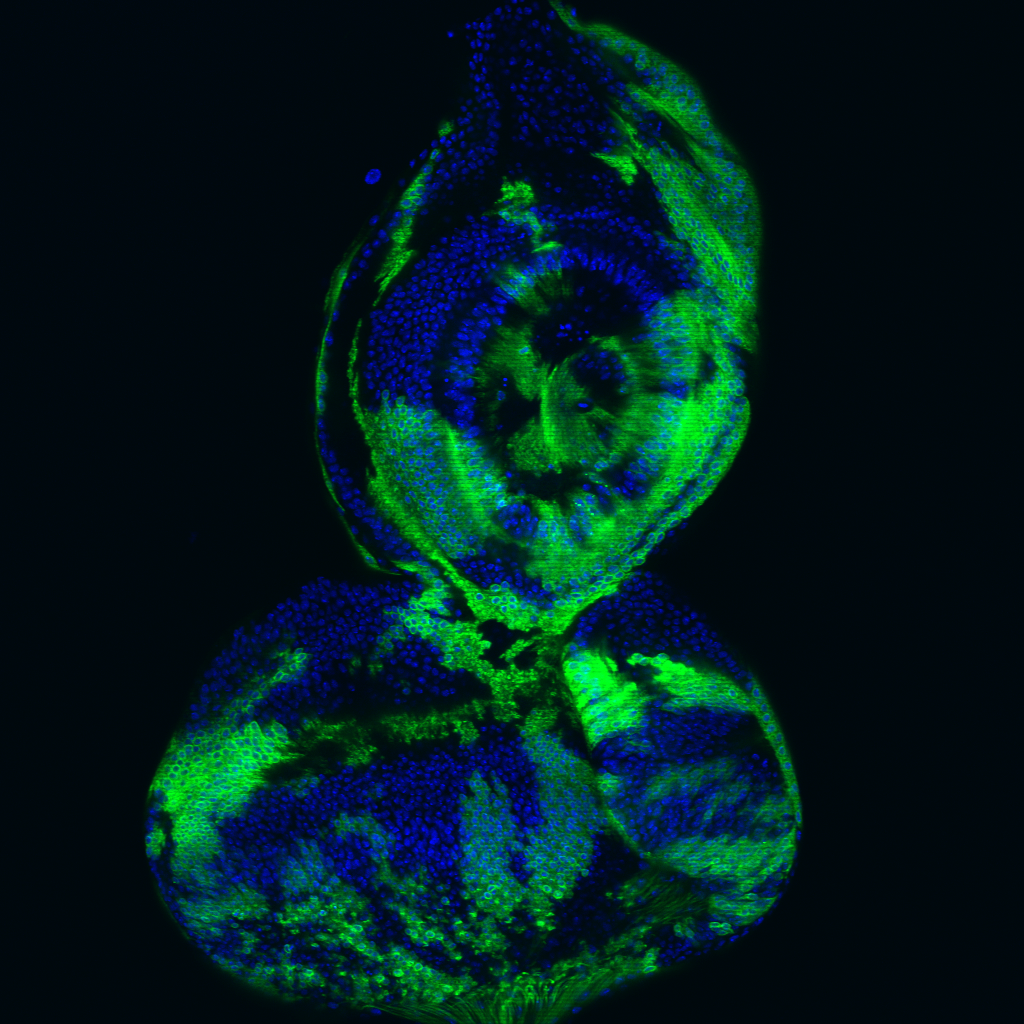

Supplement: Supplementary file 5 — Source data Fig. 1 [file 44318_2025_489_MOESM5_ESM.zip › Figure 1L/2-2 original image.tif]

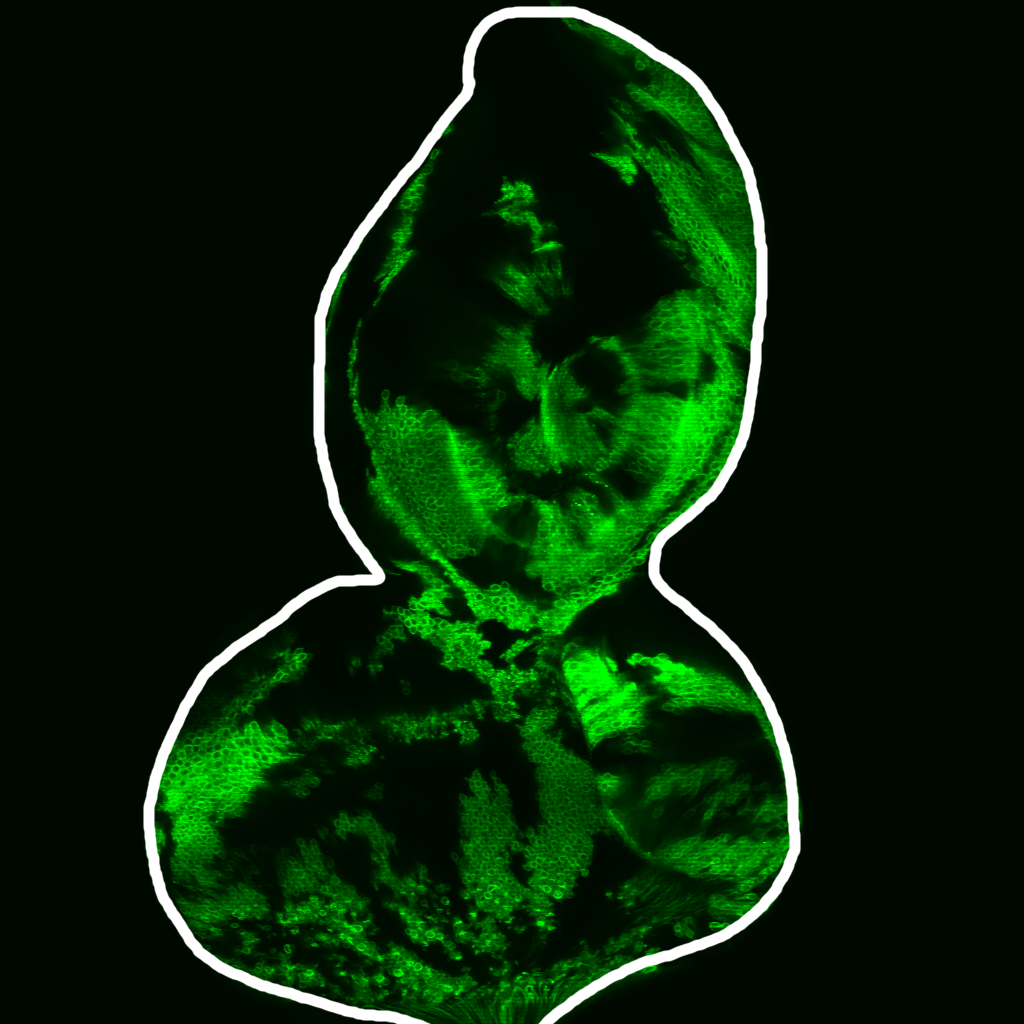

Supplement: Supplementary file 5 — Source data Fig. 1 [file 44318_2025_489_MOESM5_ESM.zip › Figure 1L/3-1 rotated and cut image with border line.tif]

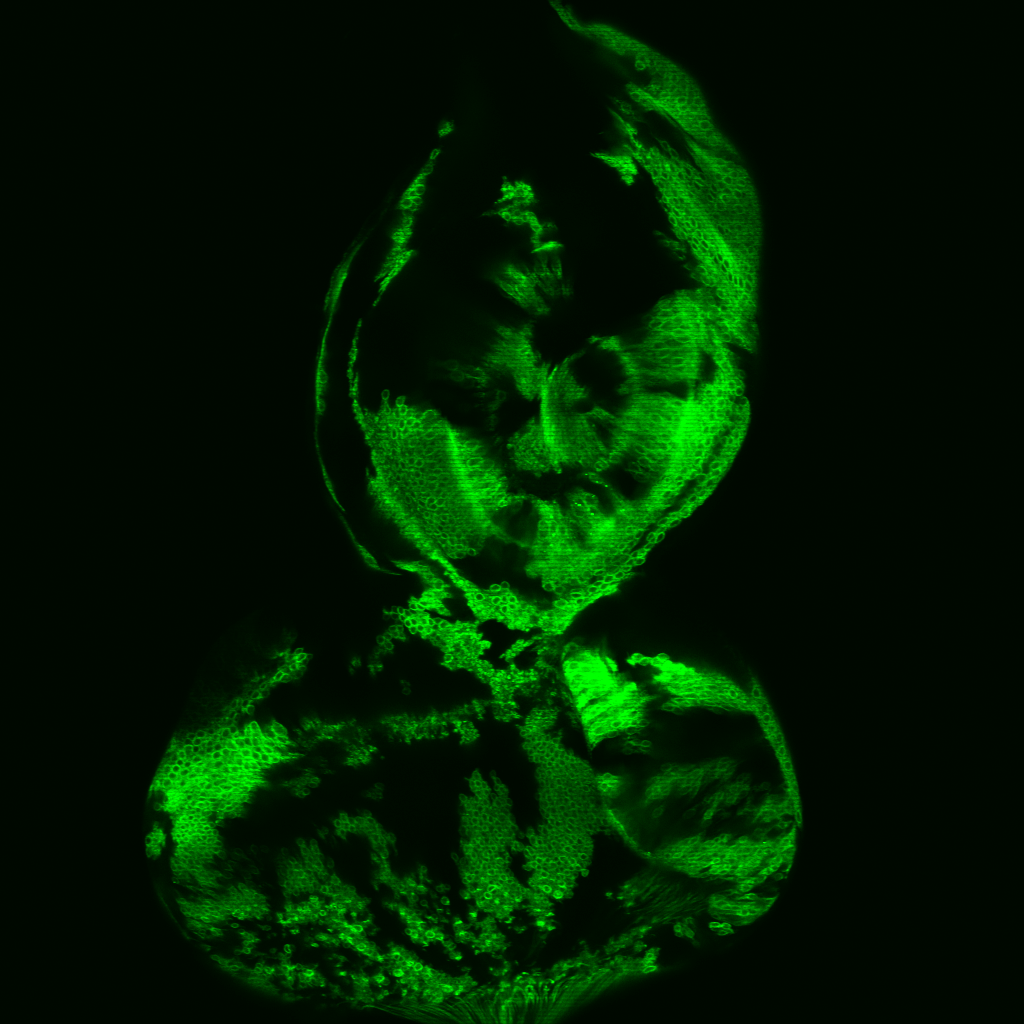

Supplement: Supplementary file 5 — Source data Fig. 1 [file 44318_2025_489_MOESM5_ESM.zip › Figure 1L/3-2 original image.tif]

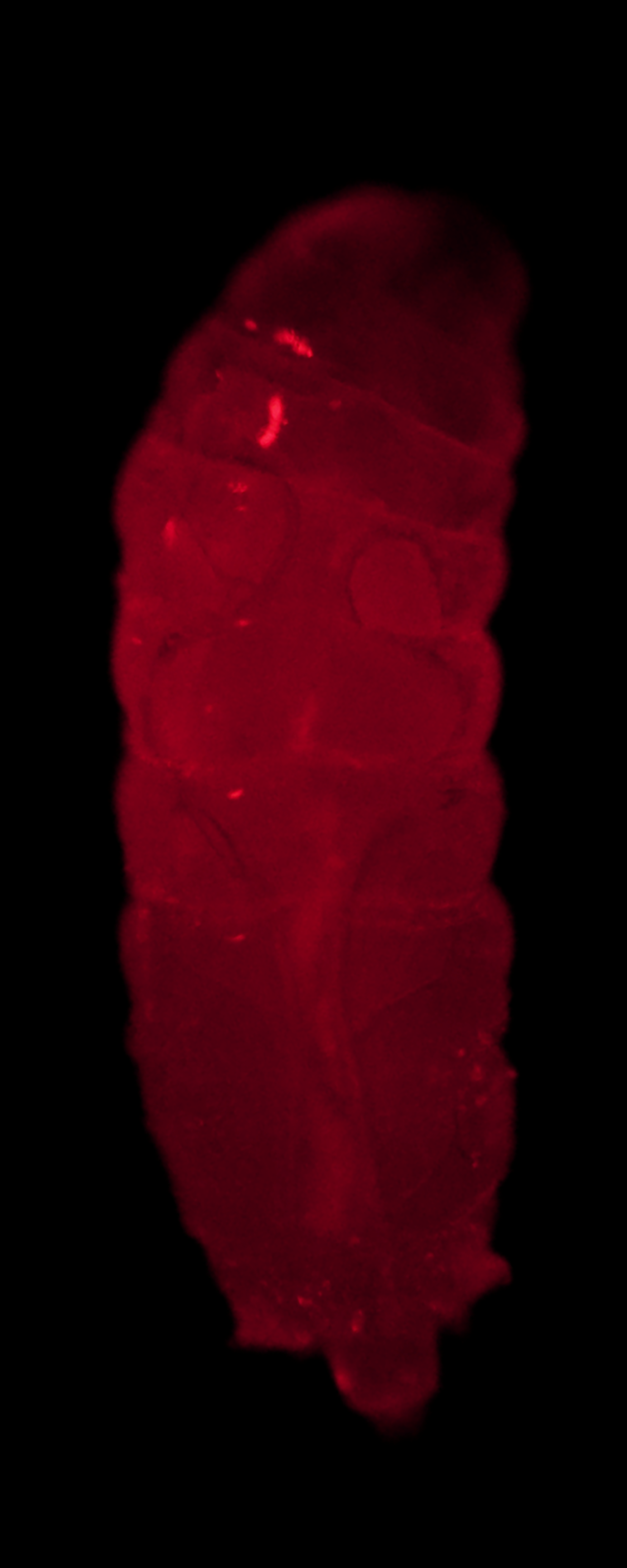

Supplement: Supplementary file 5 — Source data Fig. 1 [file 44318_2025_489_MOESM5_ESM.zip › Figure 1L/4-1 rotated and cut image.tif]

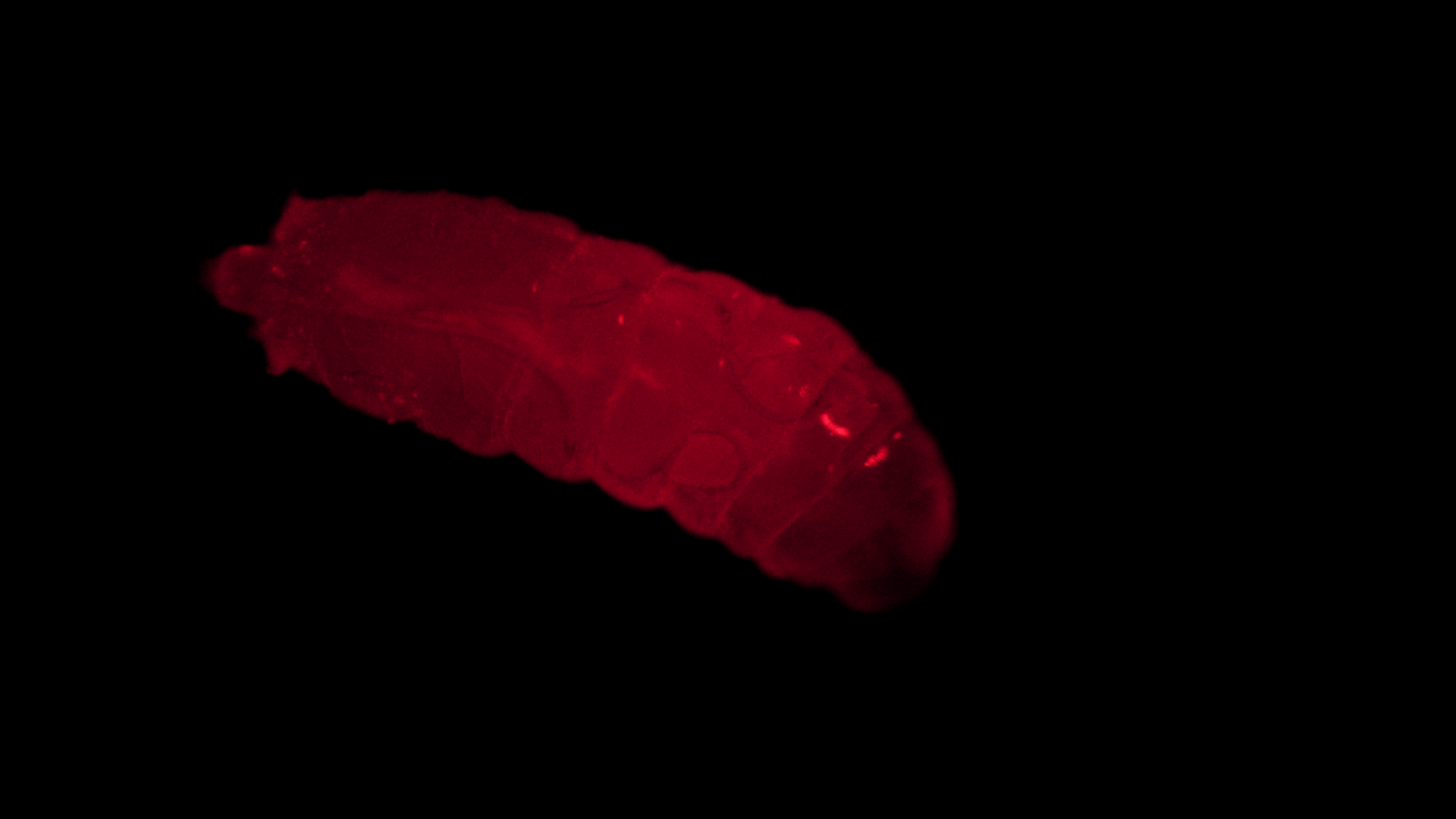

Supplement: Supplementary file 5 — Source data Fig. 1 [file 44318_2025_489_MOESM5_ESM.zip › Figure 1L/4-2 original image.tif]

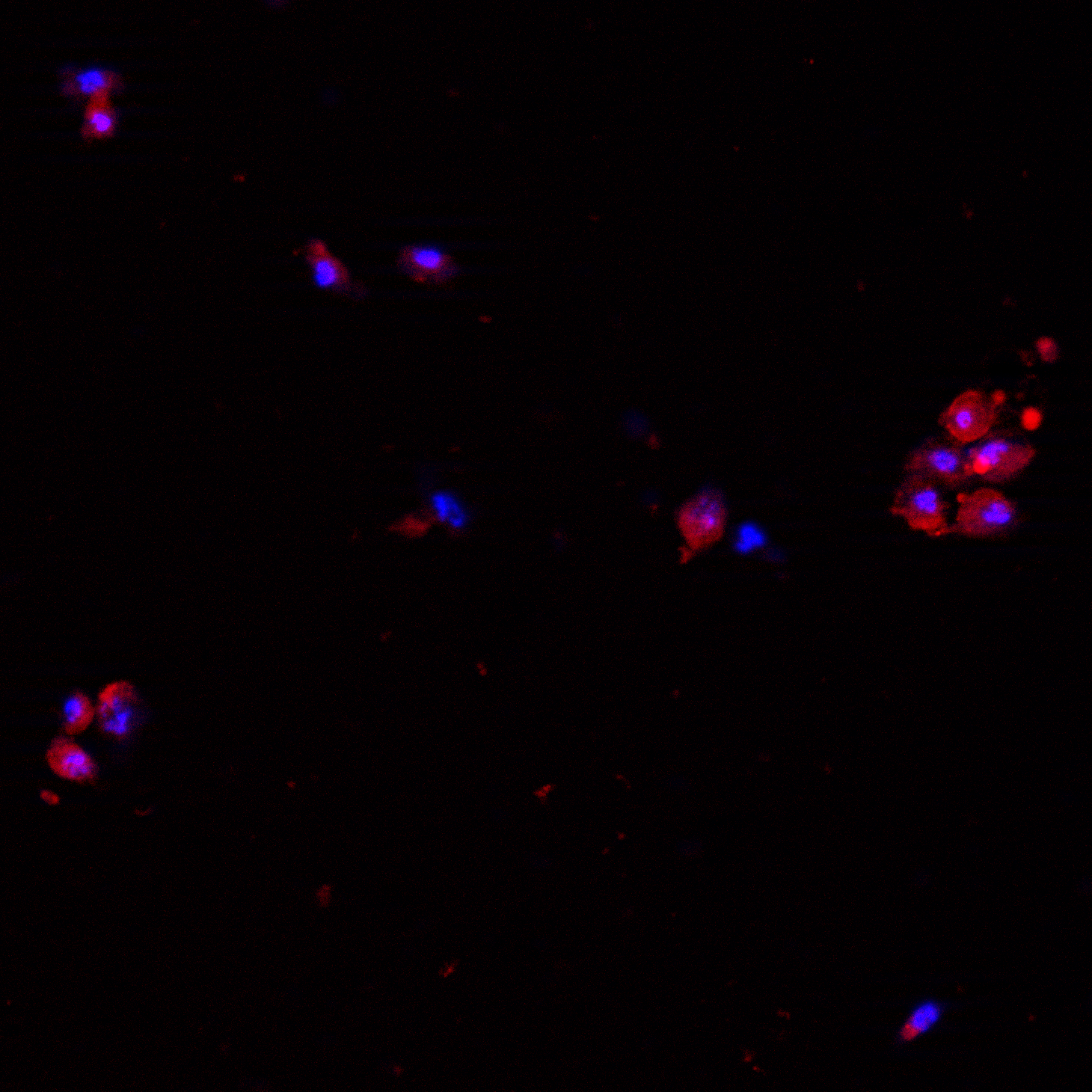

Supplement: Supplementary file 5 — Source data Fig. 1 [file 44318_2025_489_MOESM5_ESM.zip › Figure 1L/5-1 rotated and cut image.tif]

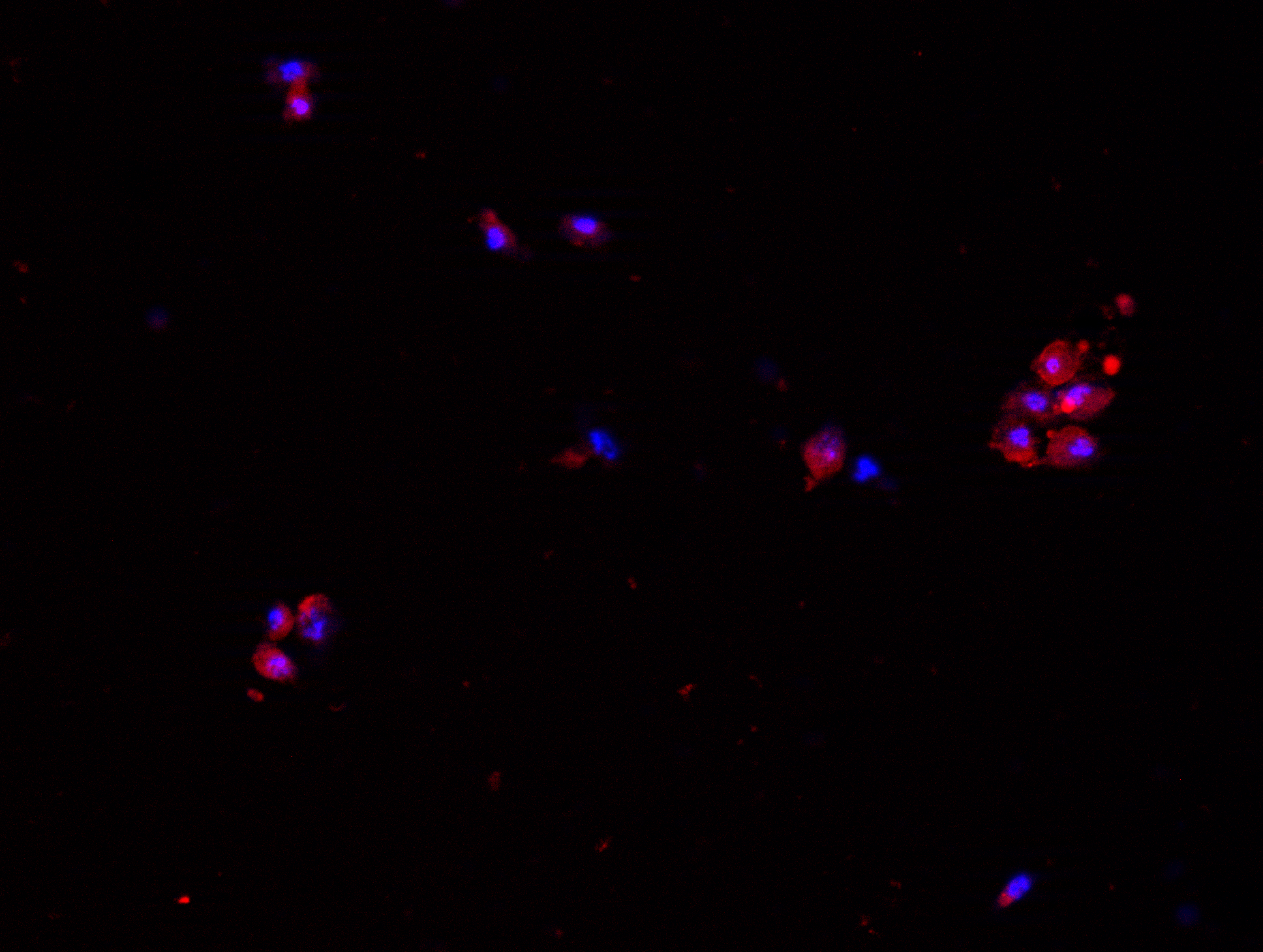

Supplement: Supplementary file 5 — Source data Fig. 1 [file 44318_2025_489_MOESM5_ESM.zip › Figure 1L/5-2 original image.tif]

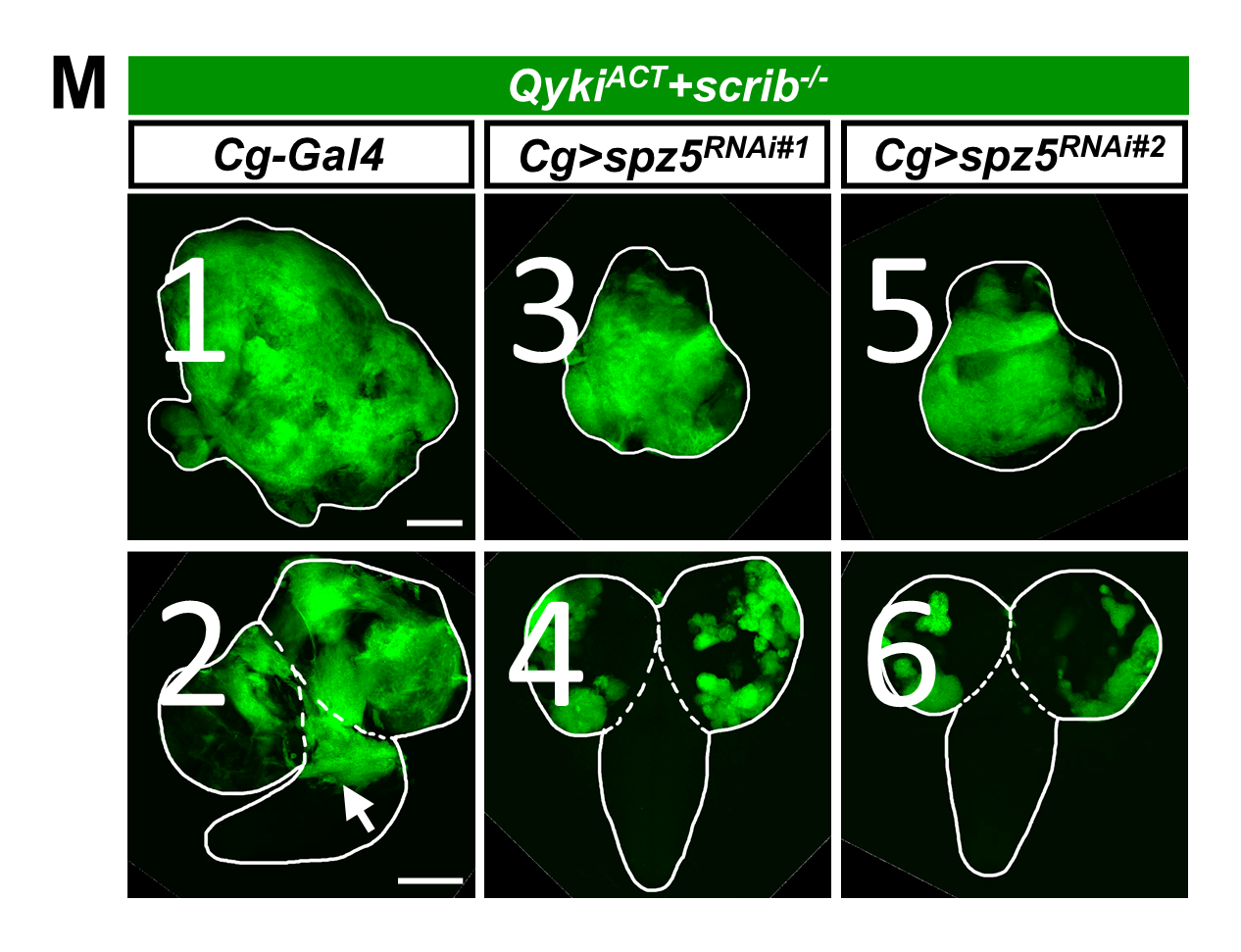

Supplement: Supplementary file 5 — Source data Fig. 1 [file 44318_2025_489_MOESM5_ESM.zip › Figure 1M/0 paper Figure 1M with provided image sequence.tif]

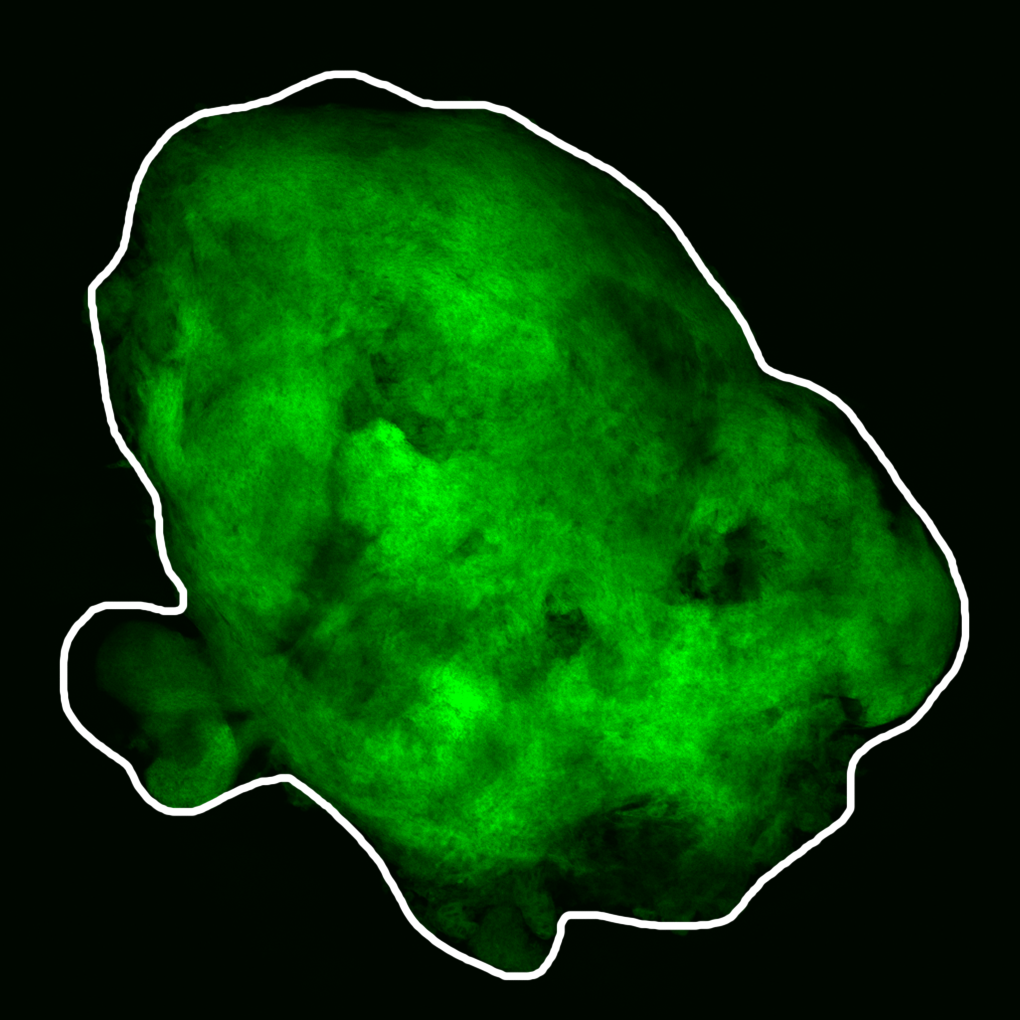

Supplement: Supplementary file 5 — Source data Fig. 1 [file 44318_2025_489_MOESM5_ESM.zip › Figure 1M/1-1 rotated and cut image with border line.tif]

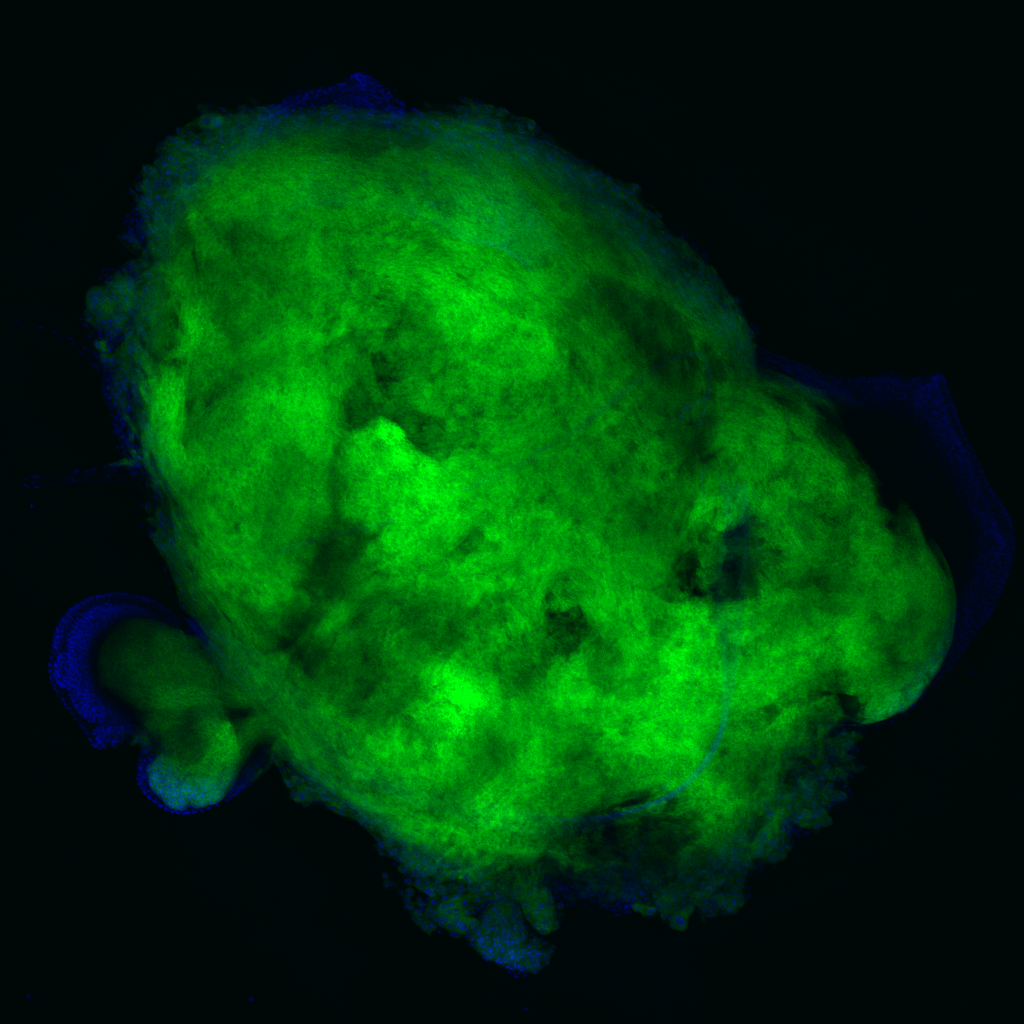

Supplement: Supplementary file 5 — Source data Fig. 1 [file 44318_2025_489_MOESM5_ESM.zip › Figure 1M/1-2 original image.tif]

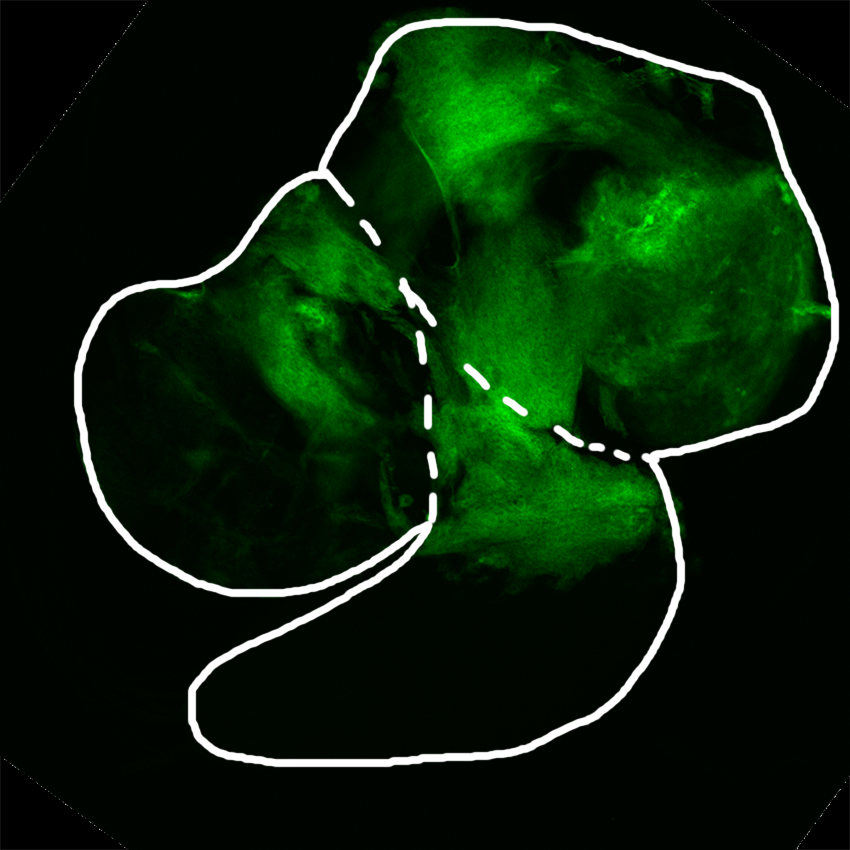

Supplement: Supplementary file 5 — Source data Fig. 1 [file 44318_2025_489_MOESM5_ESM.zip › Figure 1M/2-1 rotated and cut image with border line.tif]

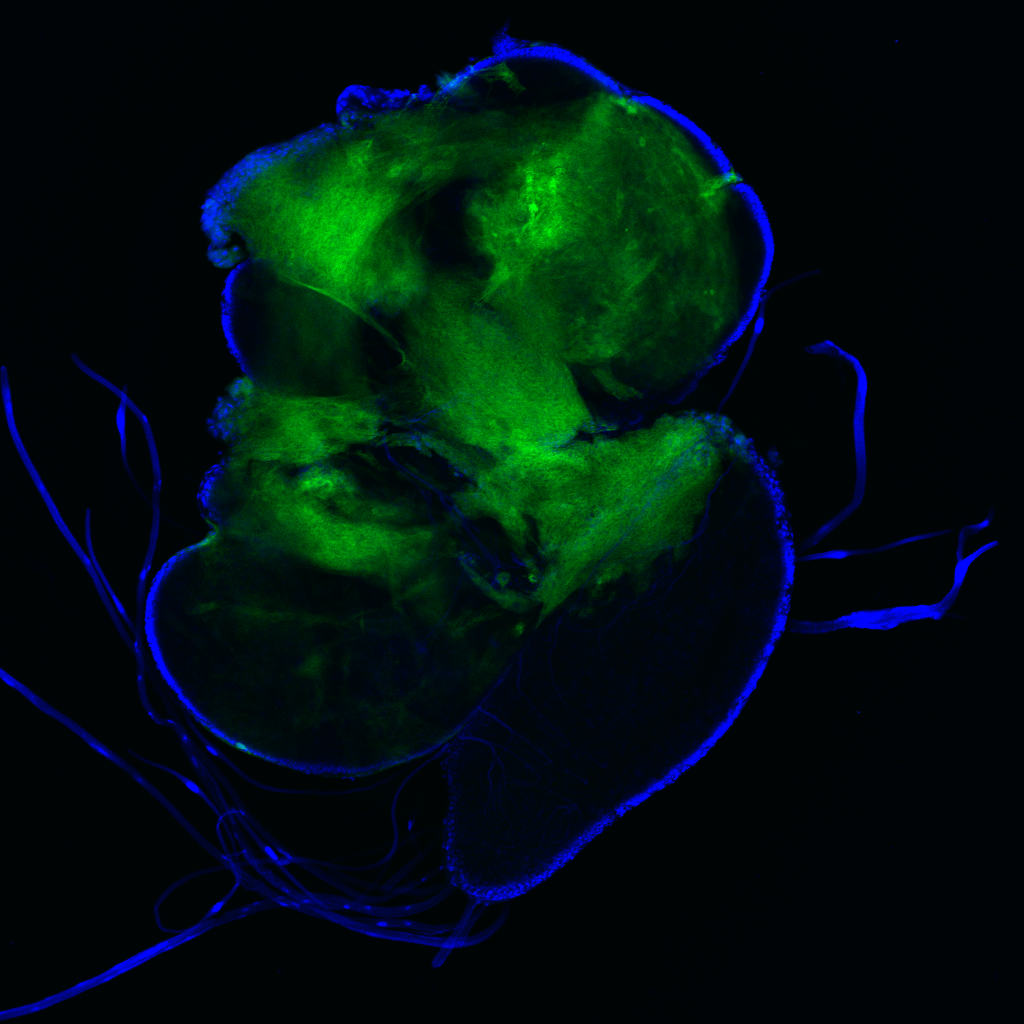

Supplement: Supplementary file 5 — Source data Fig. 1 [file 44318_2025_489_MOESM5_ESM.zip › Figure 1M/2-2 original image.tif]

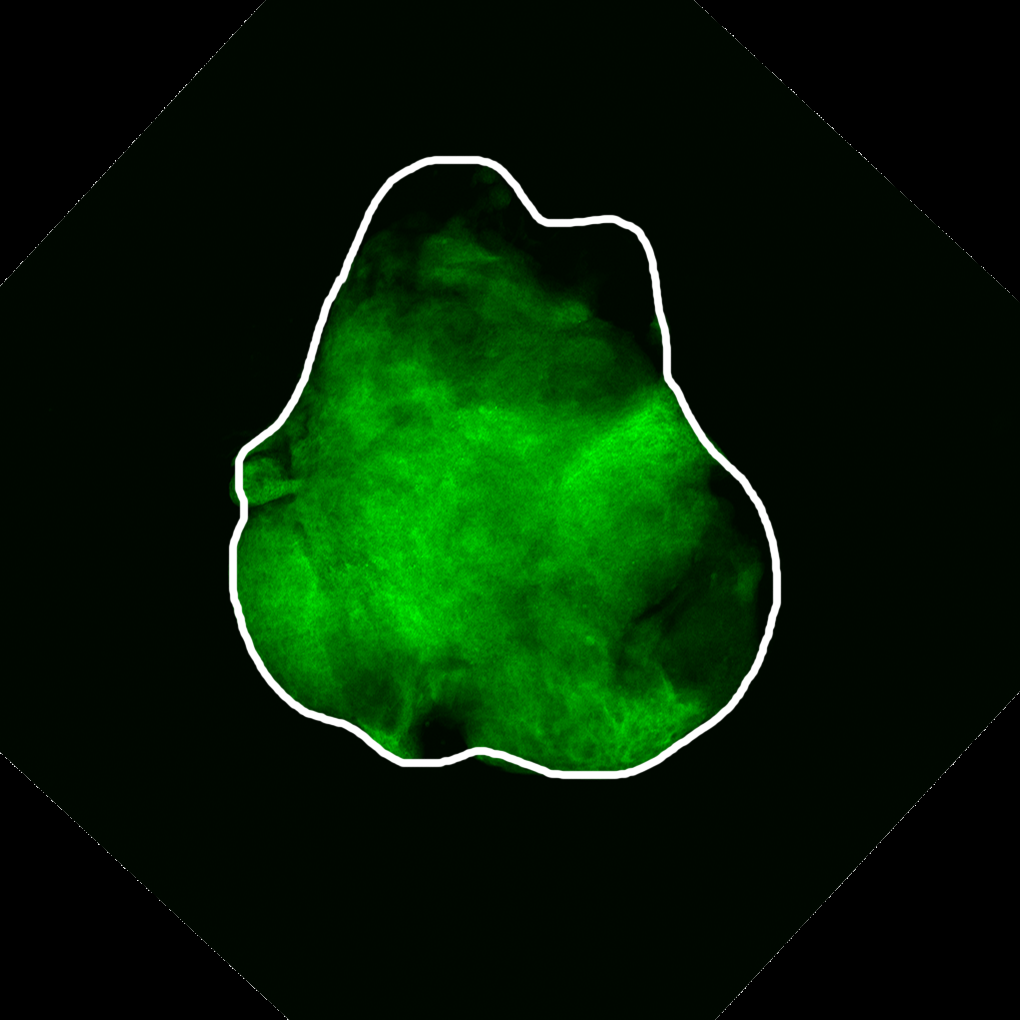

Supplement: Supplementary file 5 — Source data Fig. 1 [file 44318_2025_489_MOESM5_ESM.zip › Figure 1M/3-1 rotated and cut image with border line.tif]

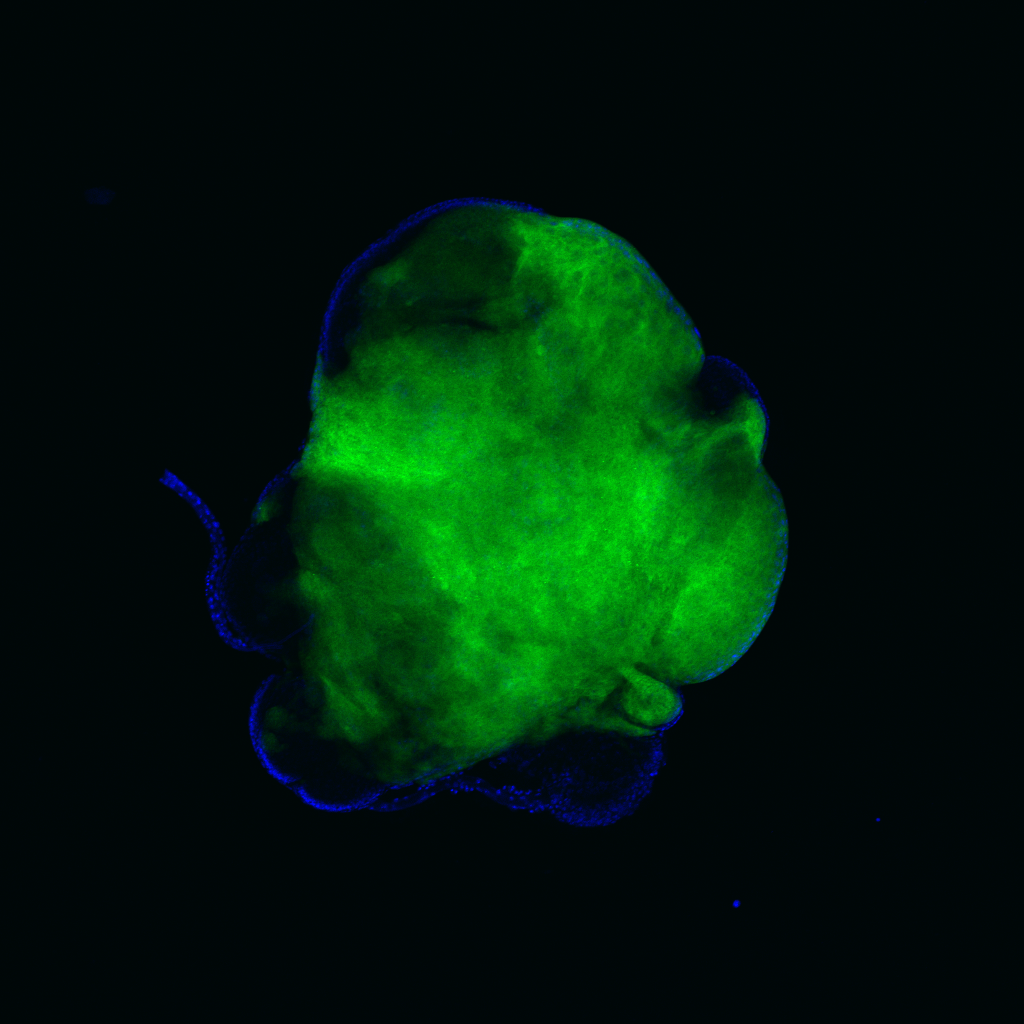

Supplement: Supplementary file 5 — Source data Fig. 1 [file 44318_2025_489_MOESM5_ESM.zip › Figure 1M/3-2 original image.tif]

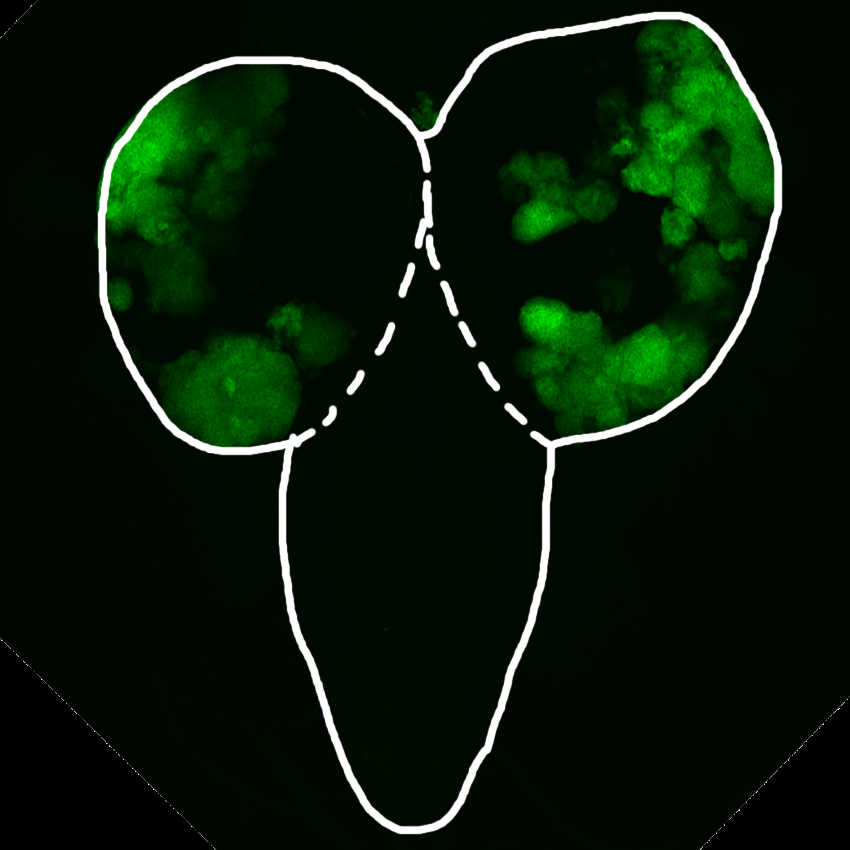

Supplement: Supplementary file 5 — Source data Fig. 1 [file 44318_2025_489_MOESM5_ESM.zip › Figure 1M/4-1 rotated and cut image with border line.tif]

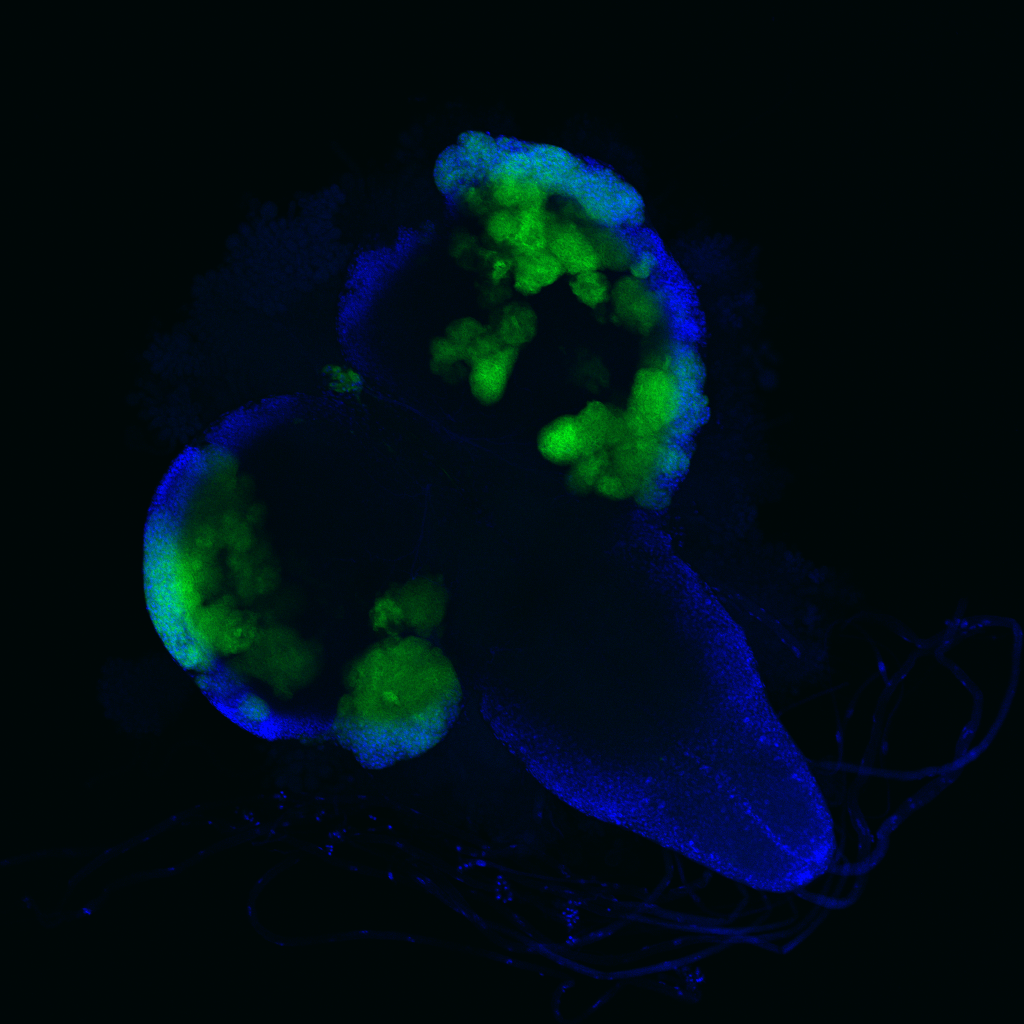

Supplement: Supplementary file 5 — Source data Fig. 1 [file 44318_2025_489_MOESM5_ESM.zip › Figure 1M/4-2 original image.tif]

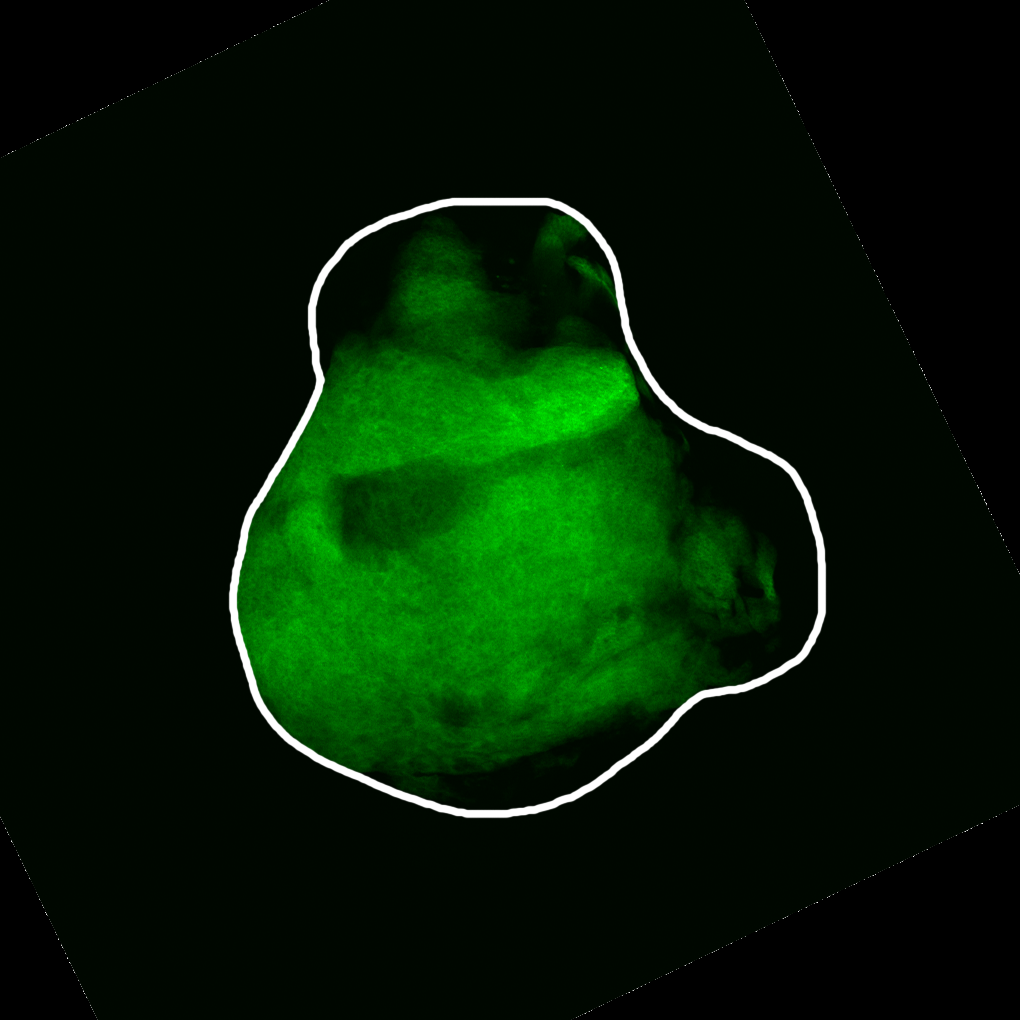

Supplement: Supplementary file 5 — Source data Fig. 1 [file 44318_2025_489_MOESM5_ESM.zip › Figure 1M/5-1 rotated and cut image with border line.tif]

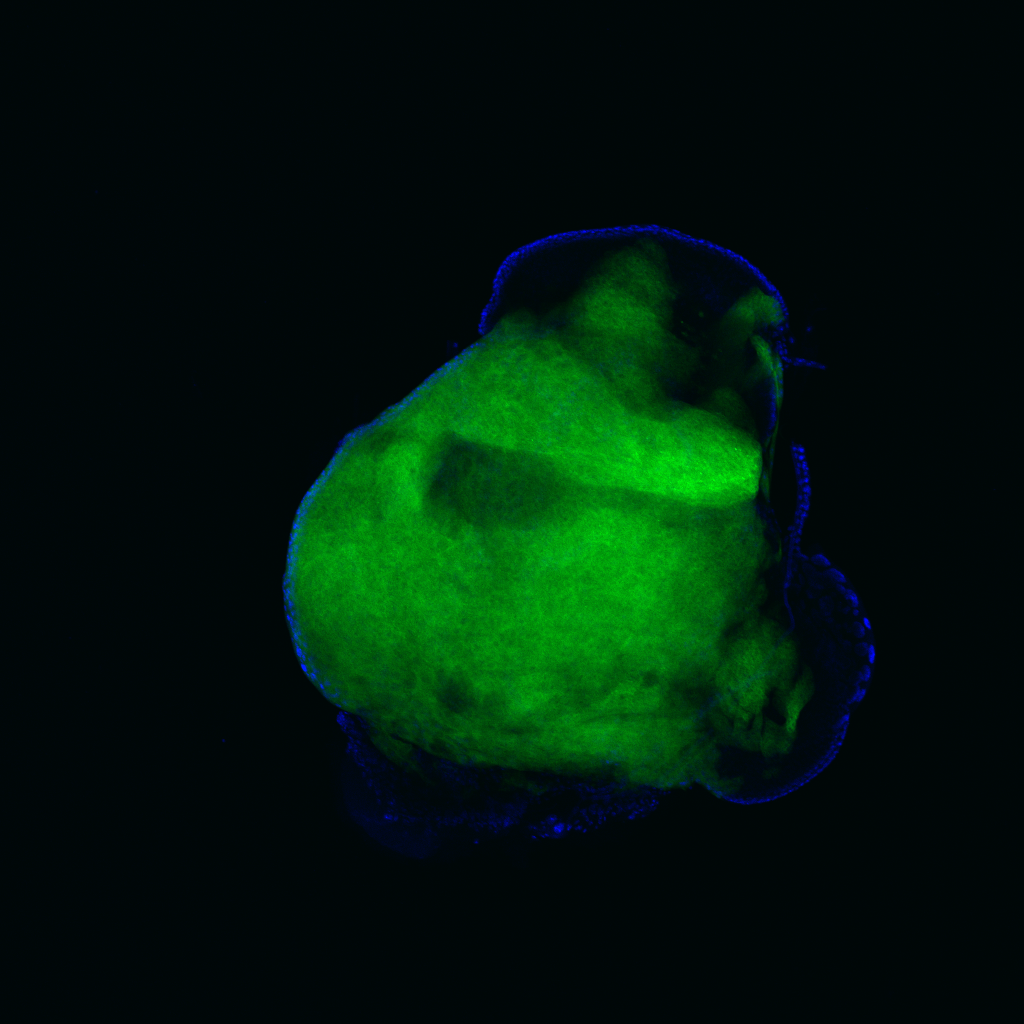

Supplement: Supplementary file 5 — Source data Fig. 1 [file 44318_2025_489_MOESM5_ESM.zip › Figure 1M/5-2 original image.tif]

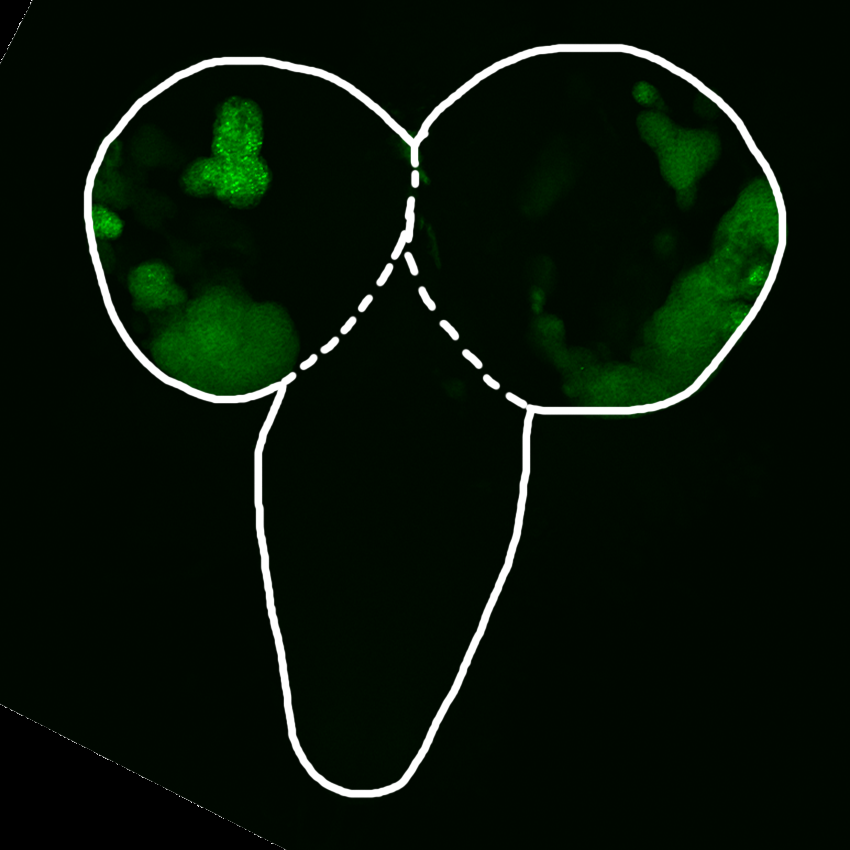

Supplement: Supplementary file 5 — Source data Fig. 1 [file 44318_2025_489_MOESM5_ESM.zip › Figure 1M/6-1 rotated and cut image with border line.tif]

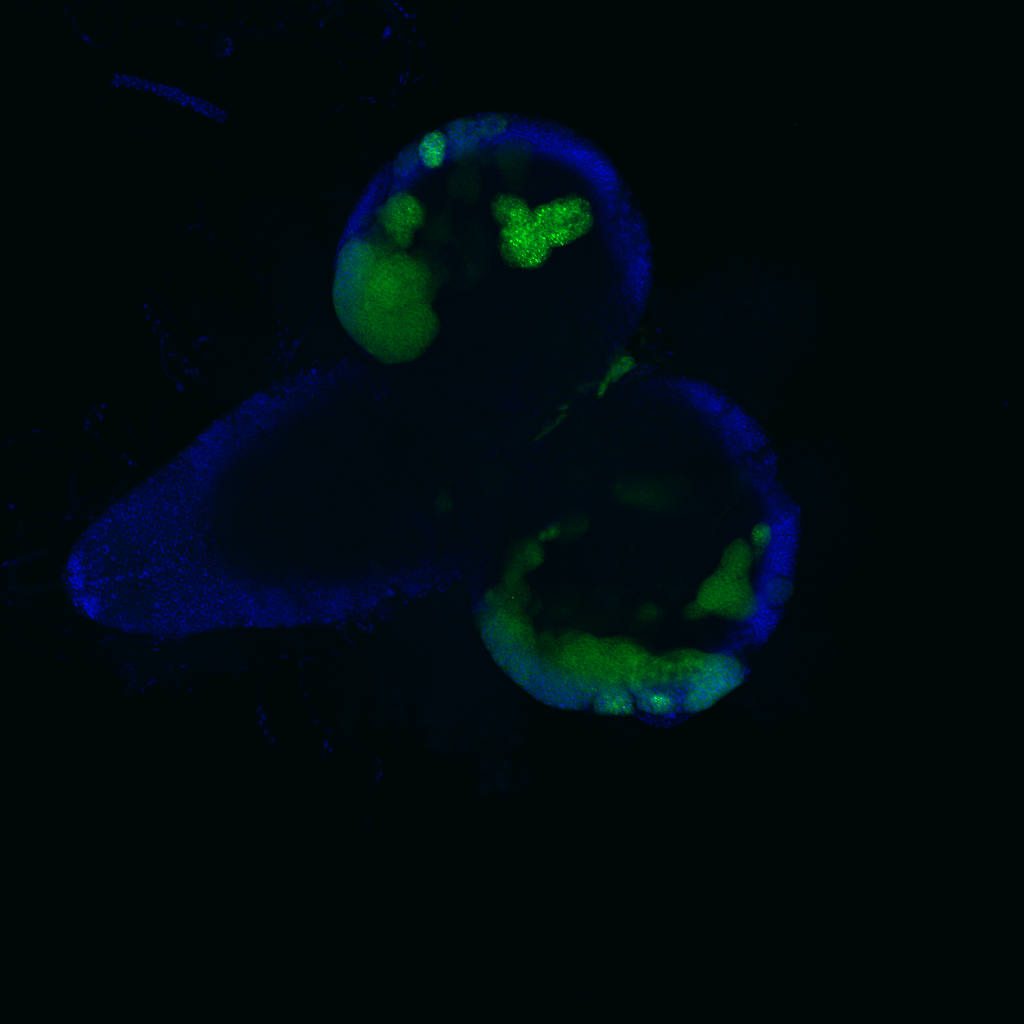

Supplement: Supplementary file 5 — Source data Fig. 1 [file 44318_2025_489_MOESM5_ESM.zip › Figure 1M/6-2 original image.tif]

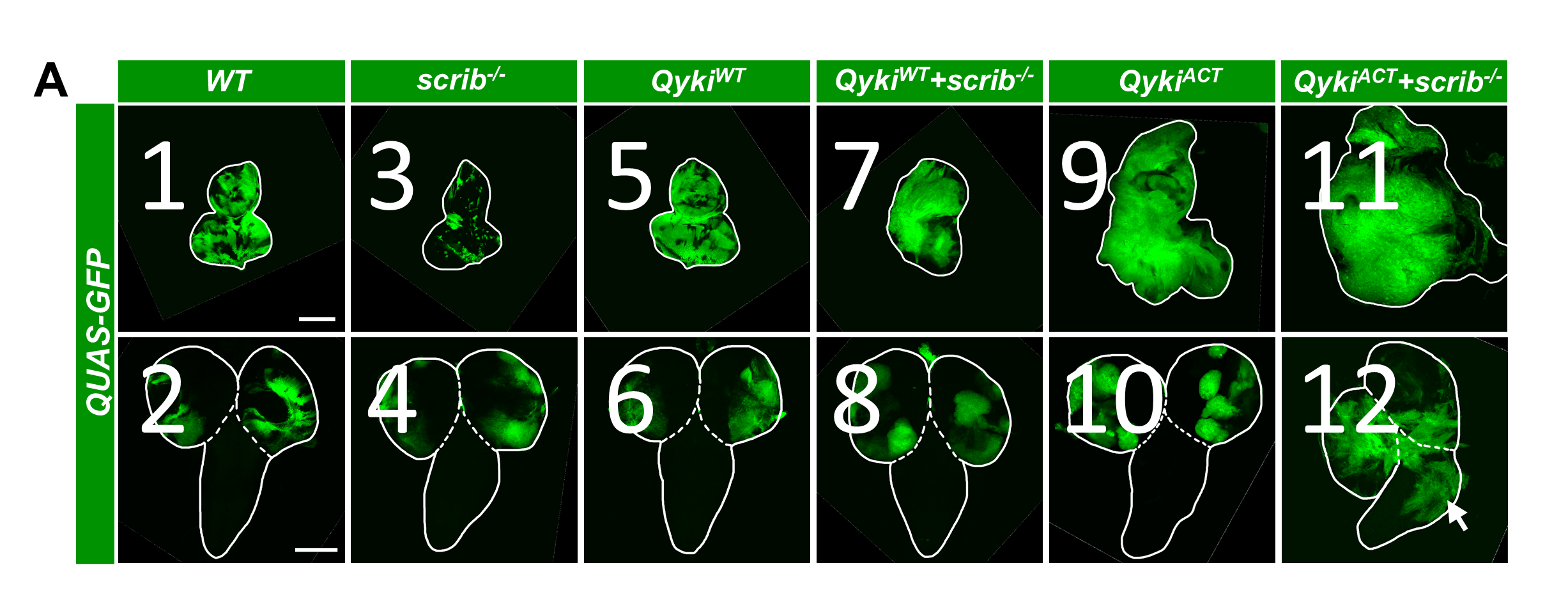

Supplement: Supplementary file 5 — Source data Fig. 1 [file 44318_2025_489_MOESM5_ESM.zip › Figure 1A/0 paper Figure 1A with provided image sequence.tif]

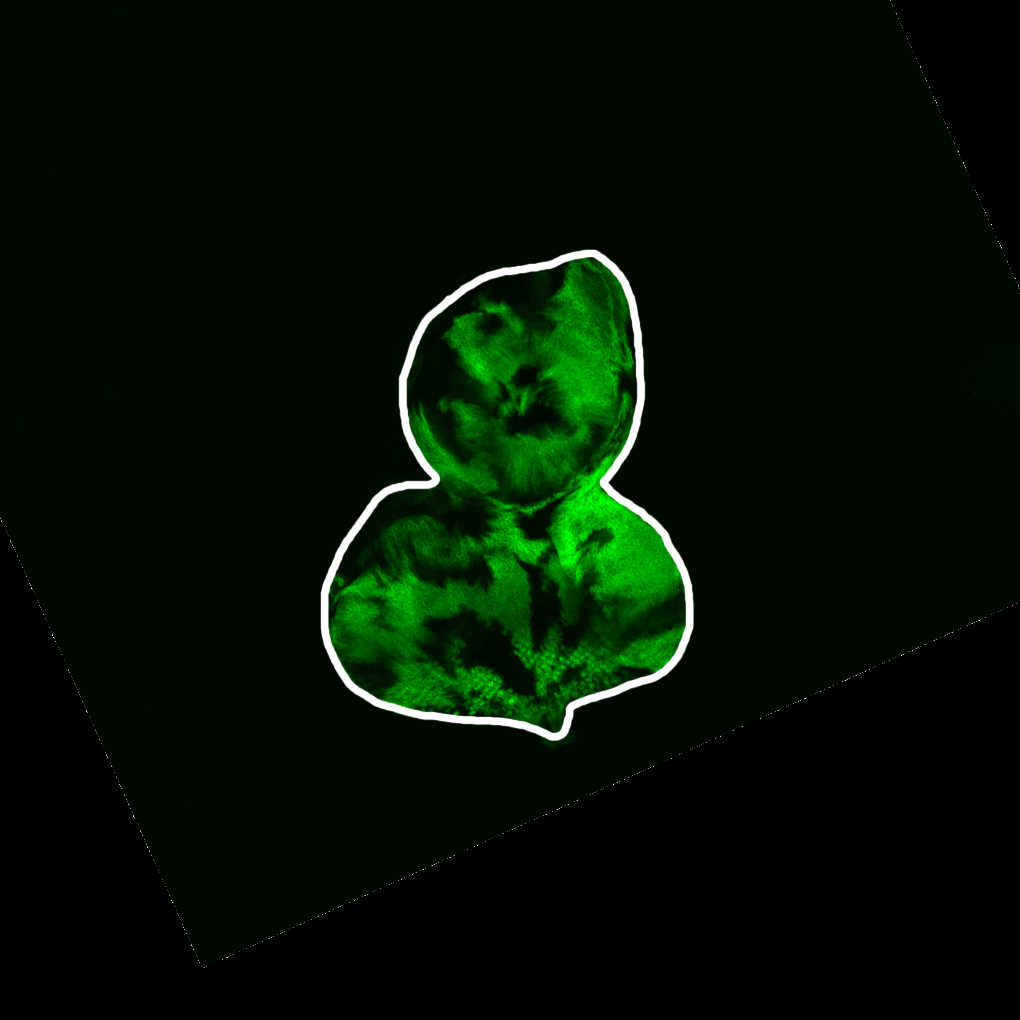

Supplement: Supplementary file 5 — Source data Fig. 1 [file 44318_2025_489_MOESM5_ESM.zip › Figure 1A/1-1 rotated and cut image with border line.tif]

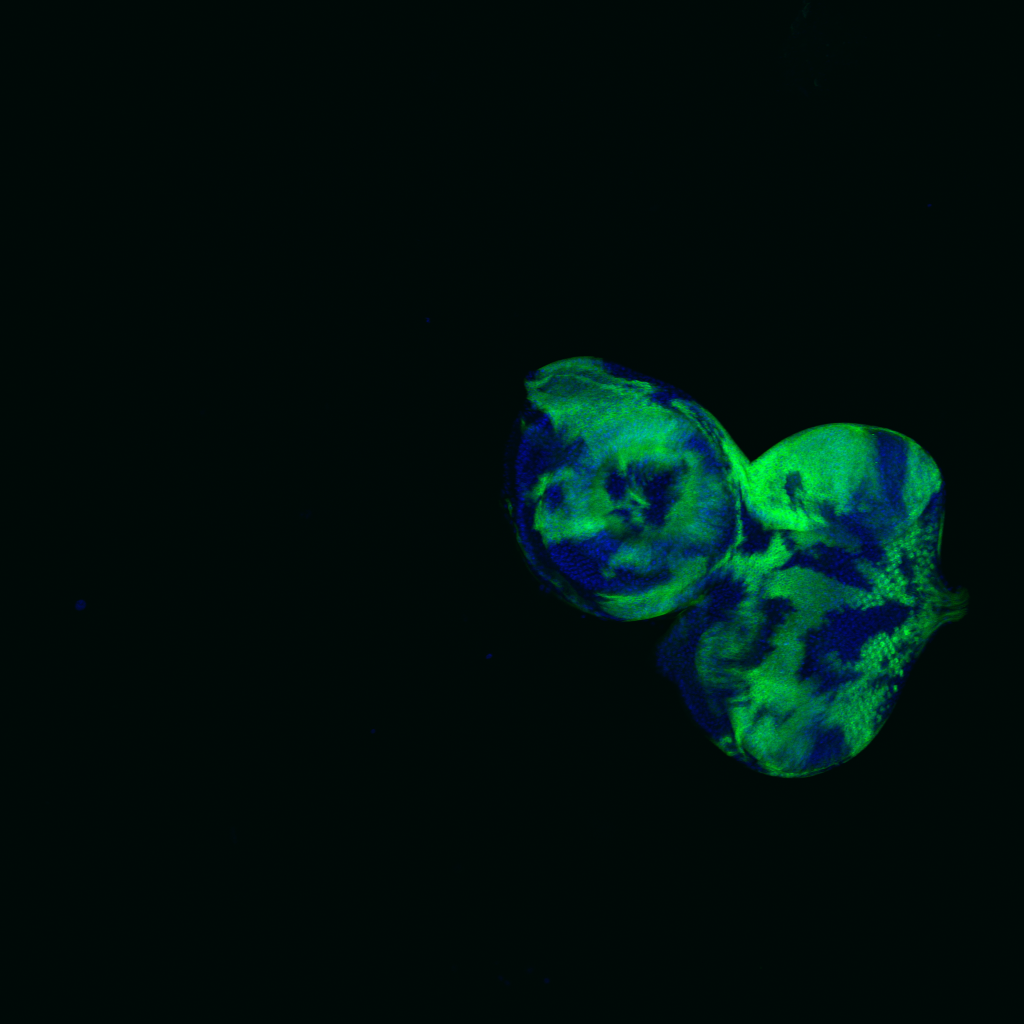

Supplement: Supplementary file 5 — Source data Fig. 1 [file 44318_2025_489_MOESM5_ESM.zip › Figure 1A/1-2 original image.tif]

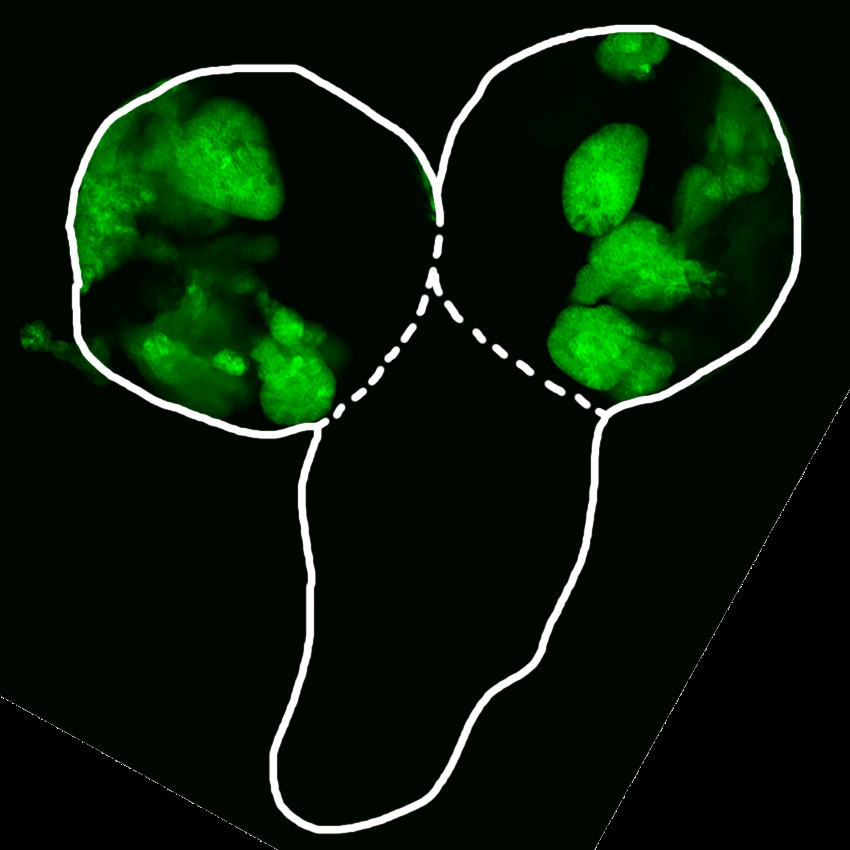

Supplement: Supplementary file 5 — Source data Fig. 1 [file 44318_2025_489_MOESM5_ESM.zip › Figure 1A/10-1 rotated and cut image with border line.tif]

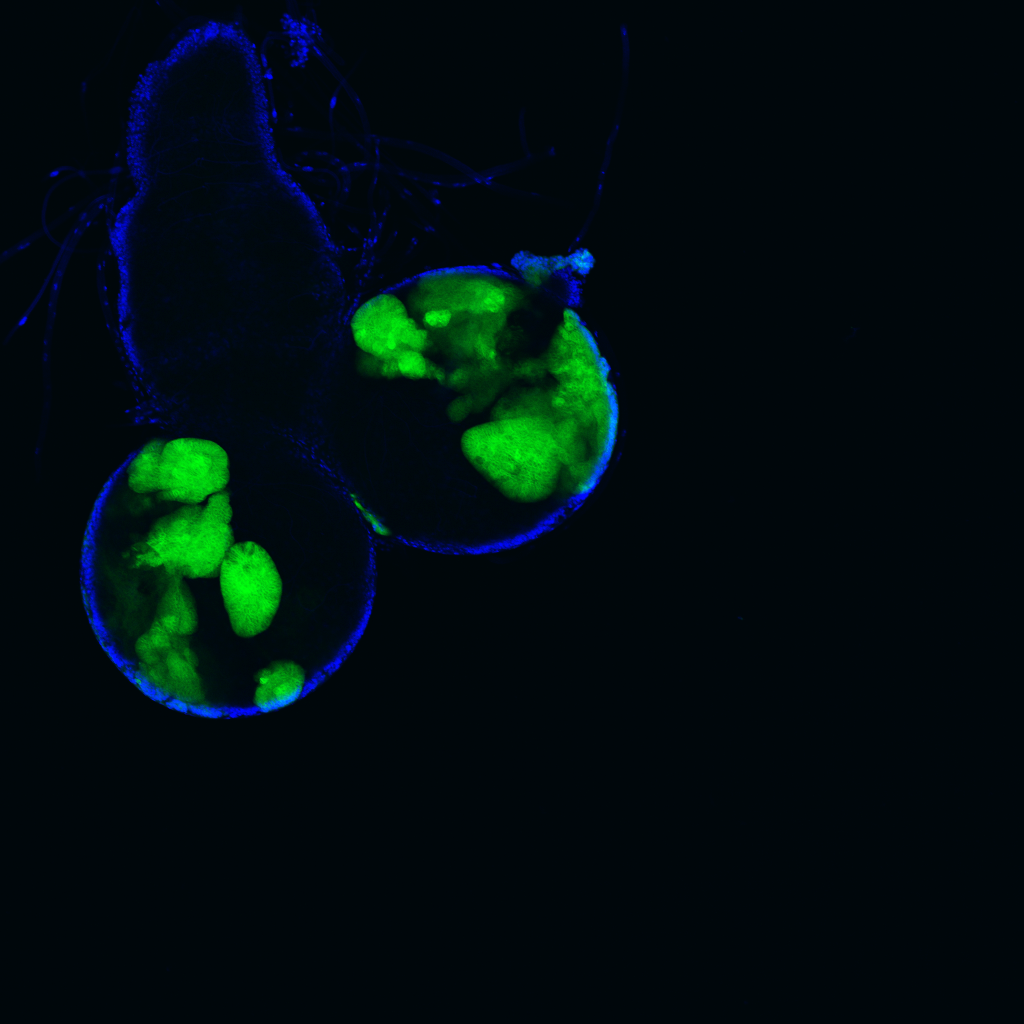

Supplement: Supplementary file 5 — Source data Fig. 1 [file 44318_2025_489_MOESM5_ESM.zip › Figure 1A/10-2 original image.tif]

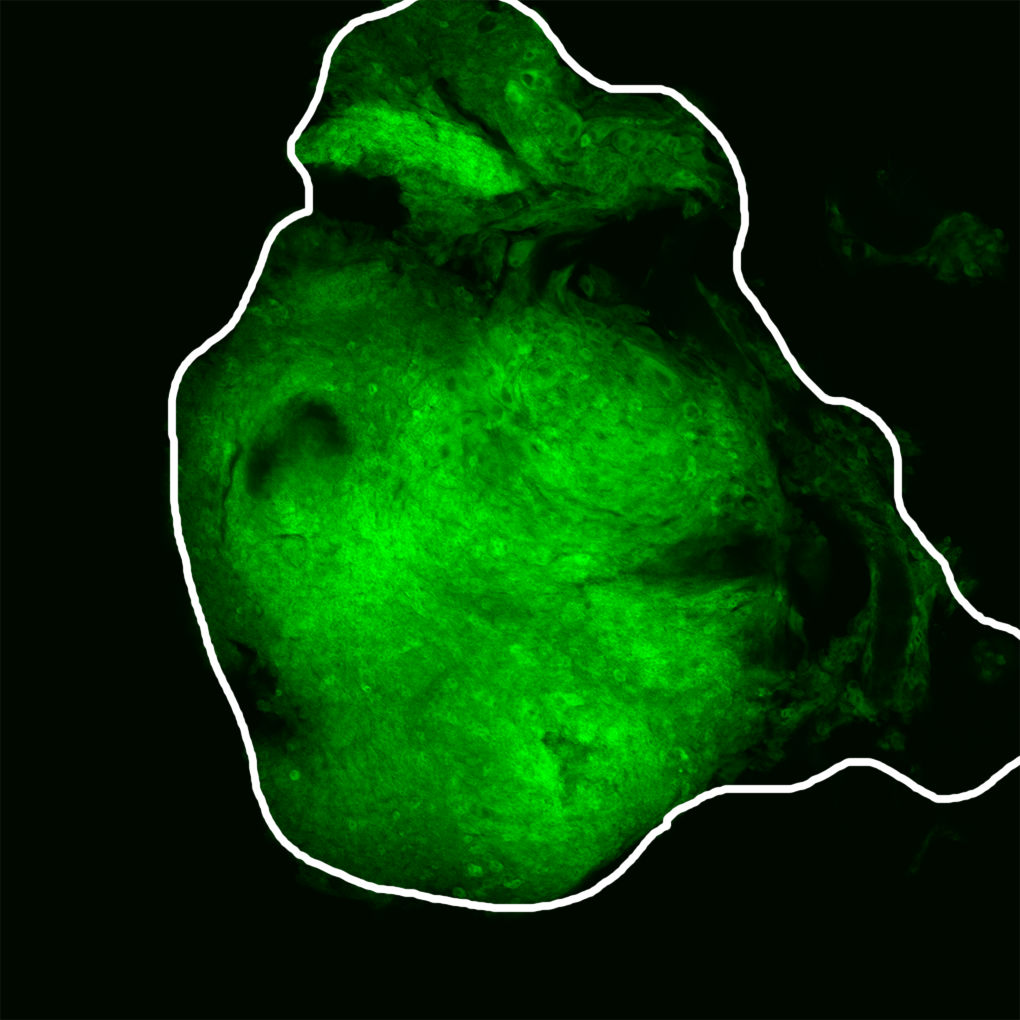

Supplement: Supplementary file 5 — Source data Fig. 1 [file 44318_2025_489_MOESM5_ESM.zip › Figure 1A/11-1 rotated and cut image with border line.tif]

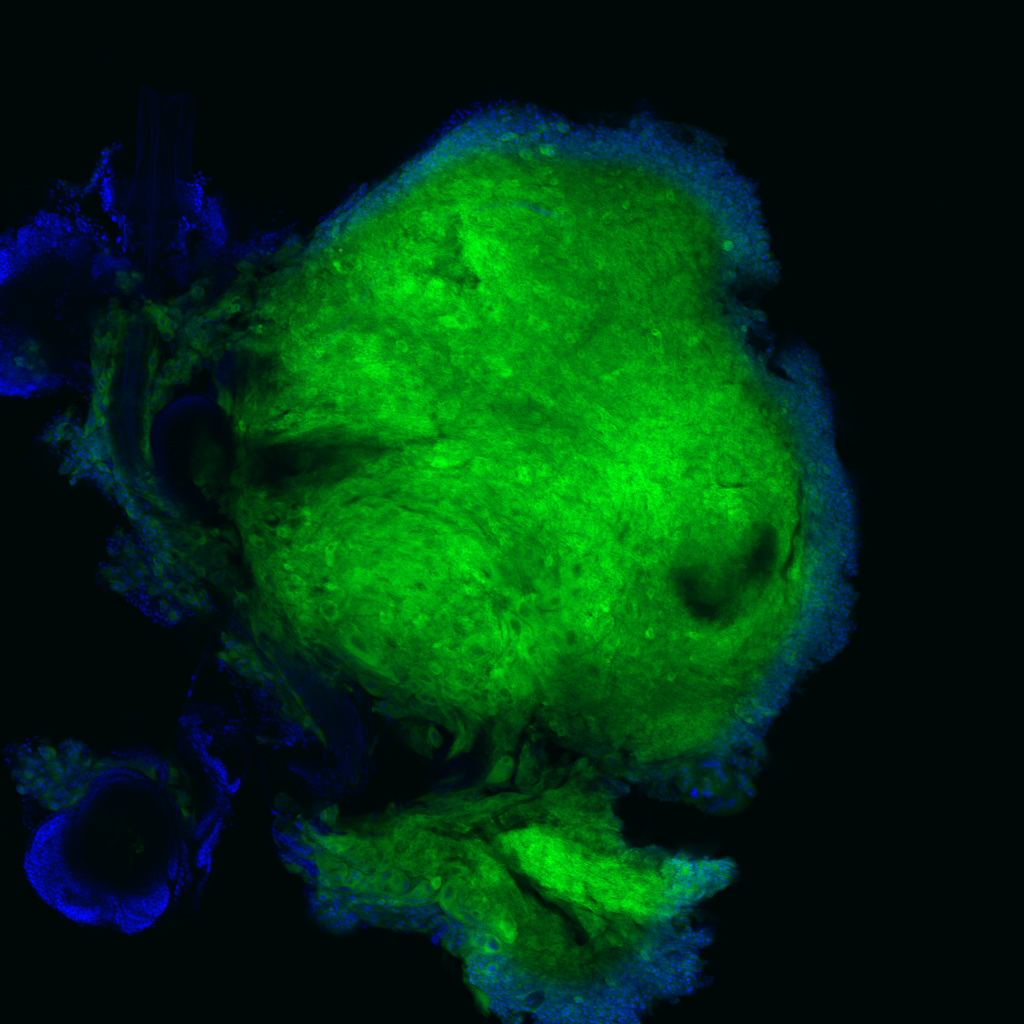

Supplement: Supplementary file 5 — Source data Fig. 1 [file 44318_2025_489_MOESM5_ESM.zip › Figure 1A/11-2 original image.tif]

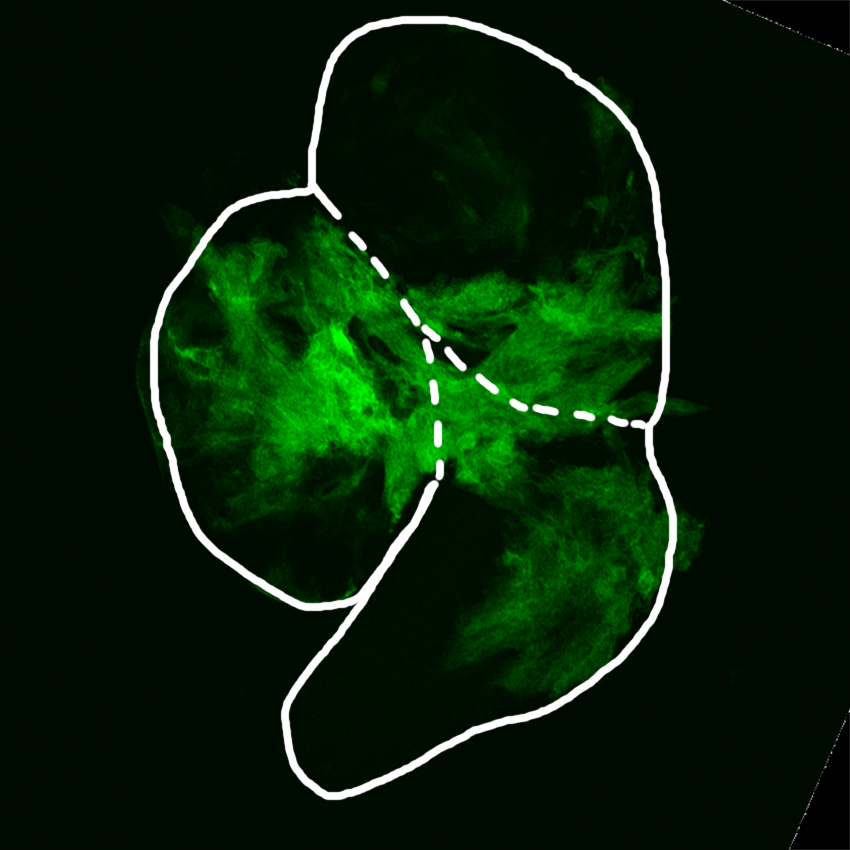

Supplement: Supplementary file 5 — Source data Fig. 1 [file 44318_2025_489_MOESM5_ESM.zip › Figure 1A/12-1 rotated and cut image with border line.tif]

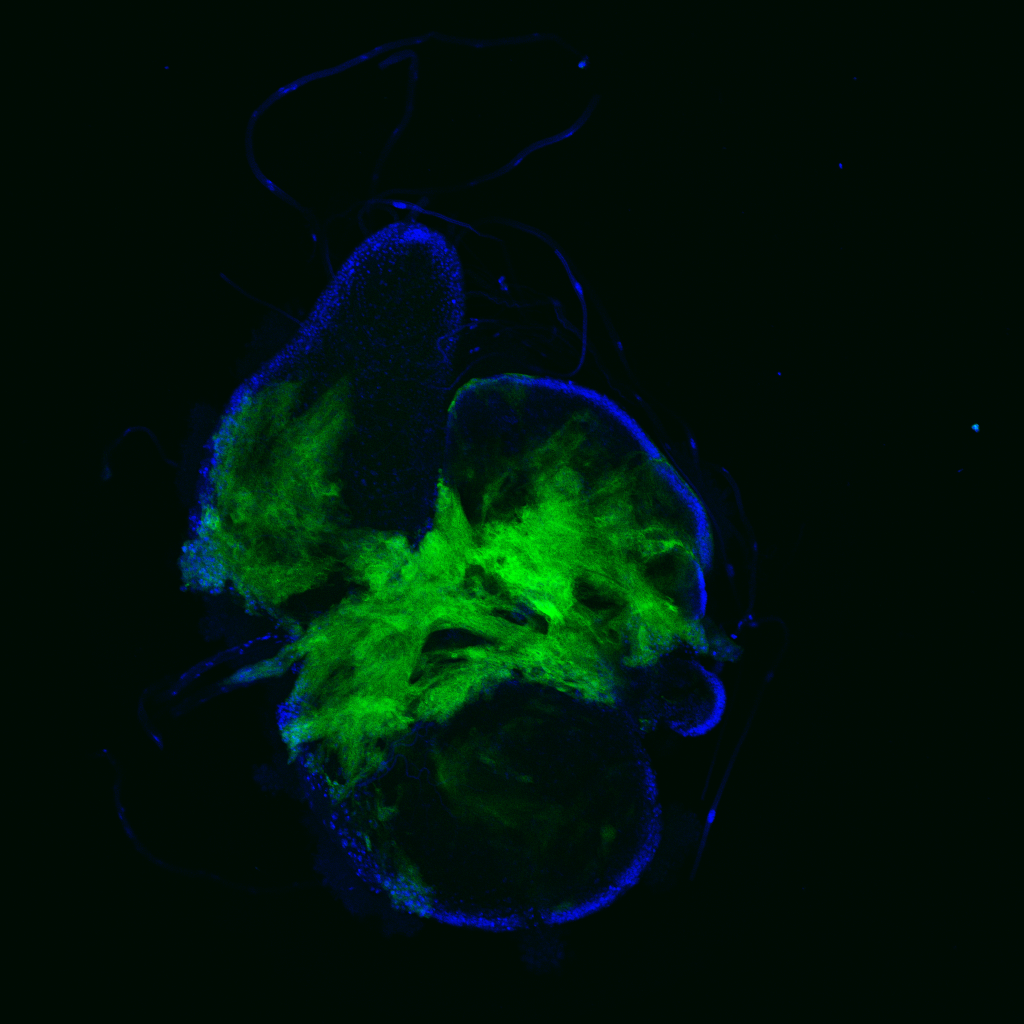

Supplement: Supplementary file 5 — Source data Fig. 1 [file 44318_2025_489_MOESM5_ESM.zip › Figure 1A/12-2 original image.tif]

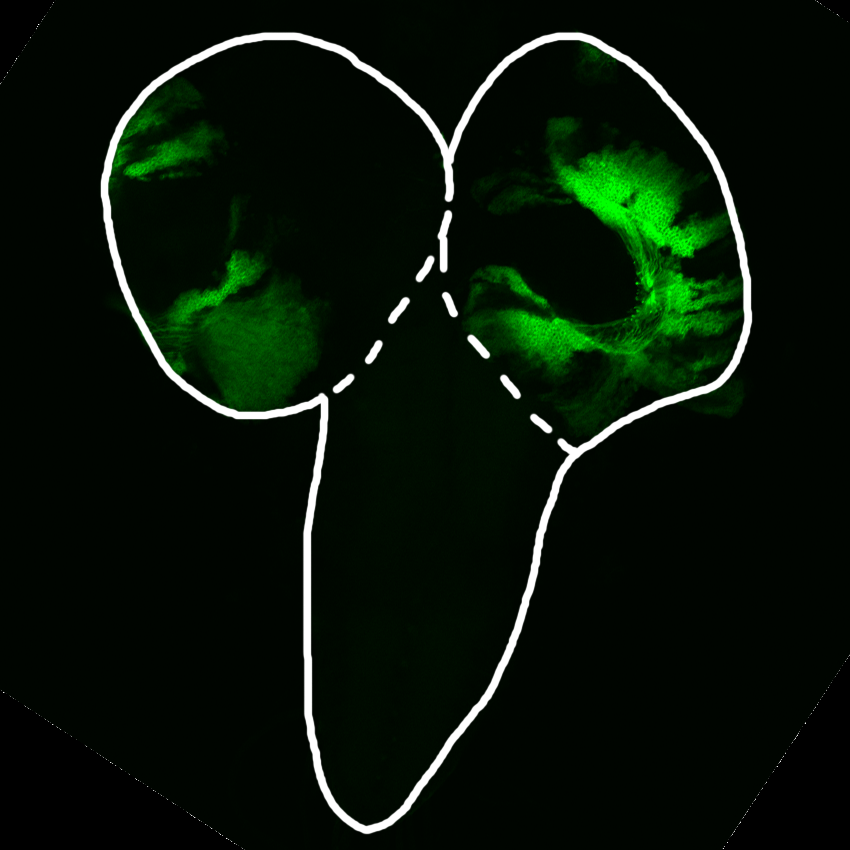

Supplement: Supplementary file 5 — Source data Fig. 1 [file 44318_2025_489_MOESM5_ESM.zip › Figure 1A/2-1 rotated and cut image with border line.tif]

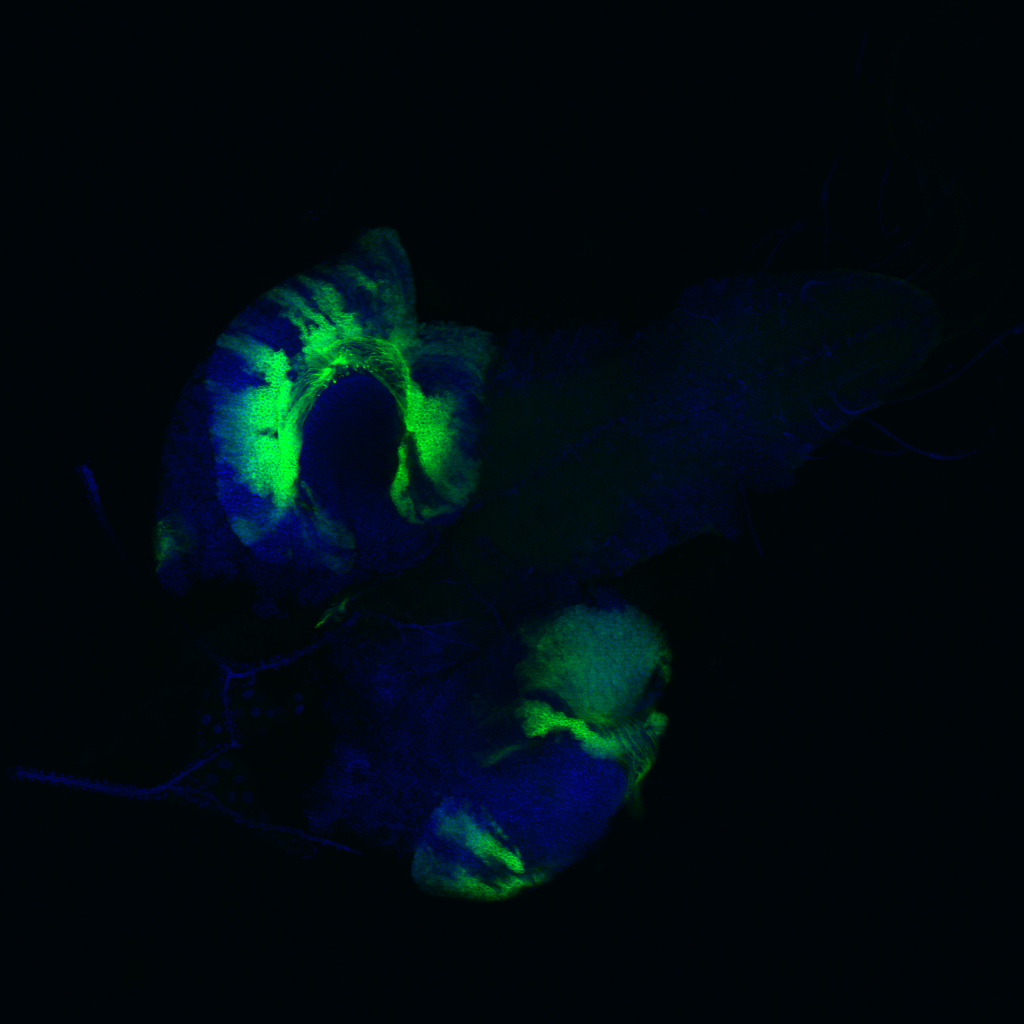

Supplement: Supplementary file 5 — Source data Fig. 1 [file 44318_2025_489_MOESM5_ESM.zip › Figure 1A/2-2 original image.tif]

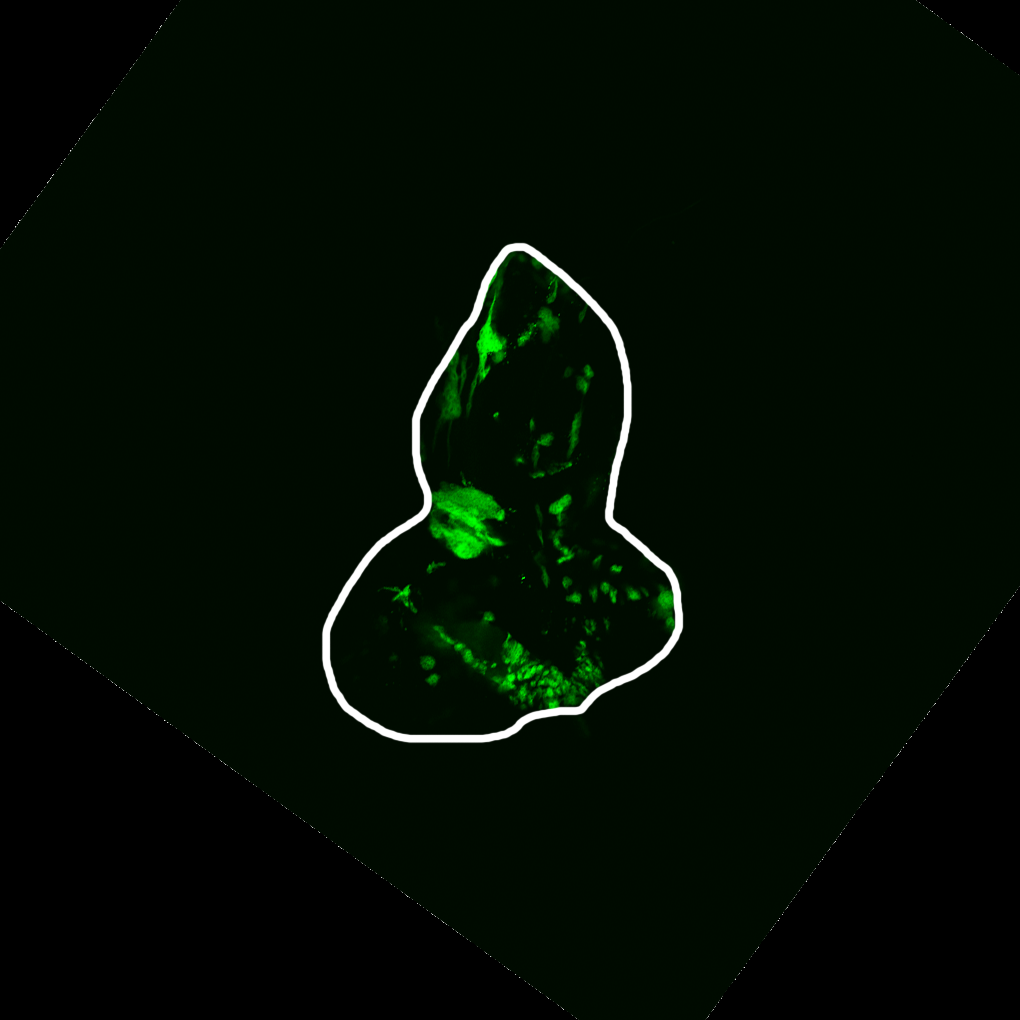

Supplement: Supplementary file 5 — Source data Fig. 1 [file 44318_2025_489_MOESM5_ESM.zip › Figure 1A/3-1 rotated and cut image with border line.tif]

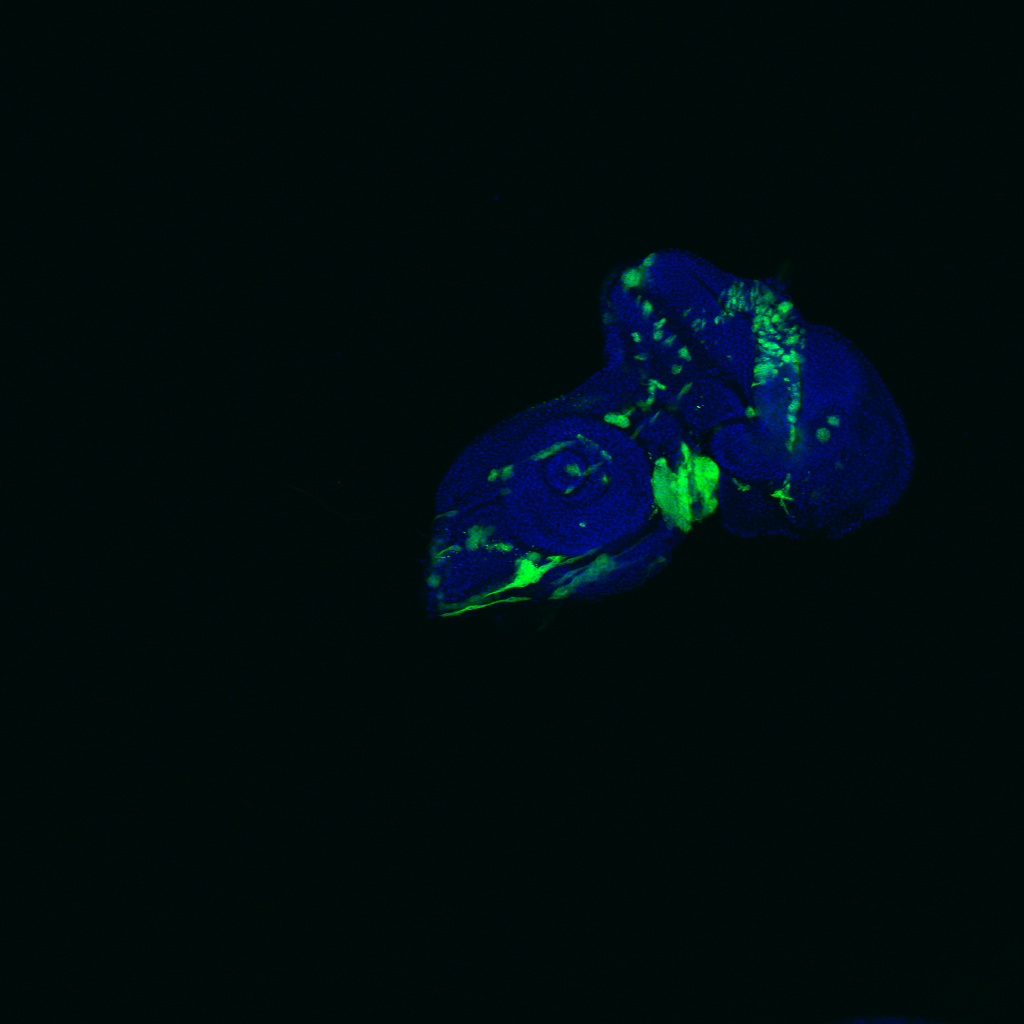

Supplement: Supplementary file 5 — Source data Fig. 1 [file 44318_2025_489_MOESM5_ESM.zip › Figure 1A/3-2 original image.tif]

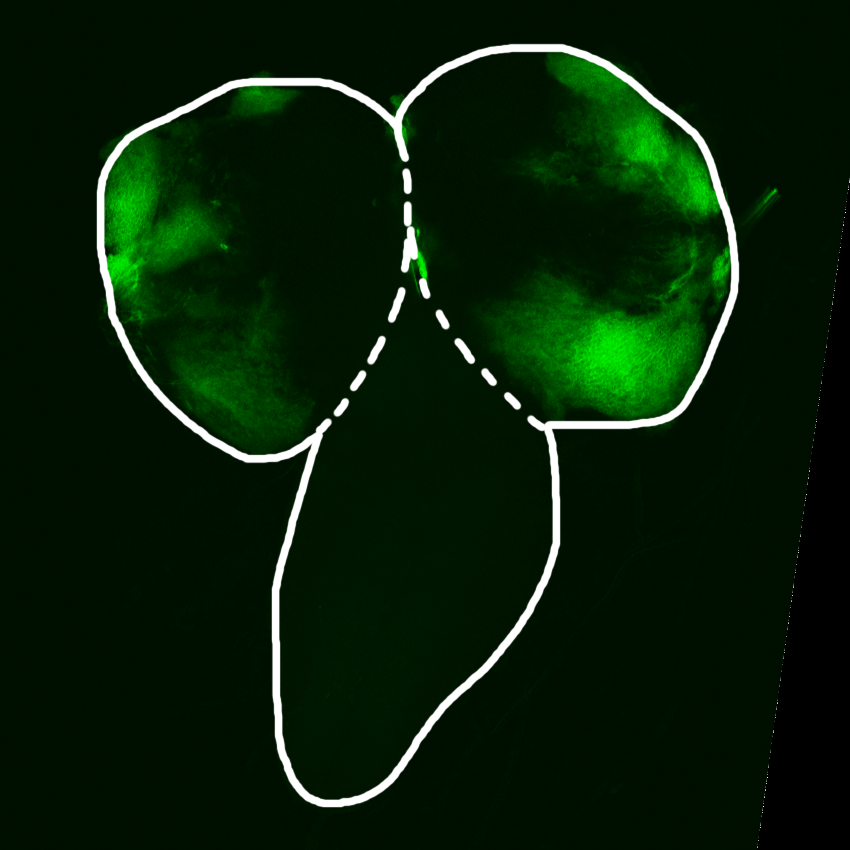

Supplement: Supplementary file 5 — Source data Fig. 1 [file 44318_2025_489_MOESM5_ESM.zip › Figure 1A/4-1 rotated and cut image with border line.tif]

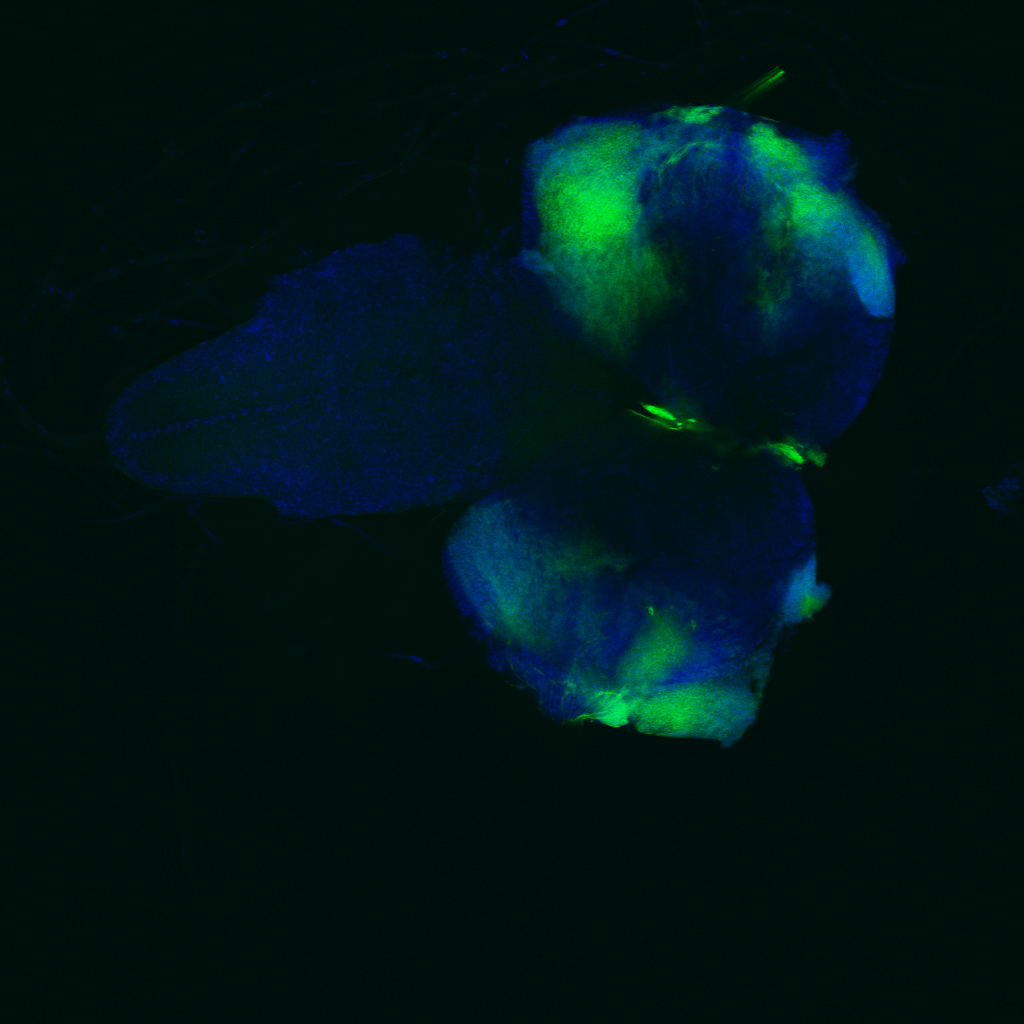

Supplement: Supplementary file 5 — Source data Fig. 1 [file 44318_2025_489_MOESM5_ESM.zip › Figure 1A/4-2 original image.tif]

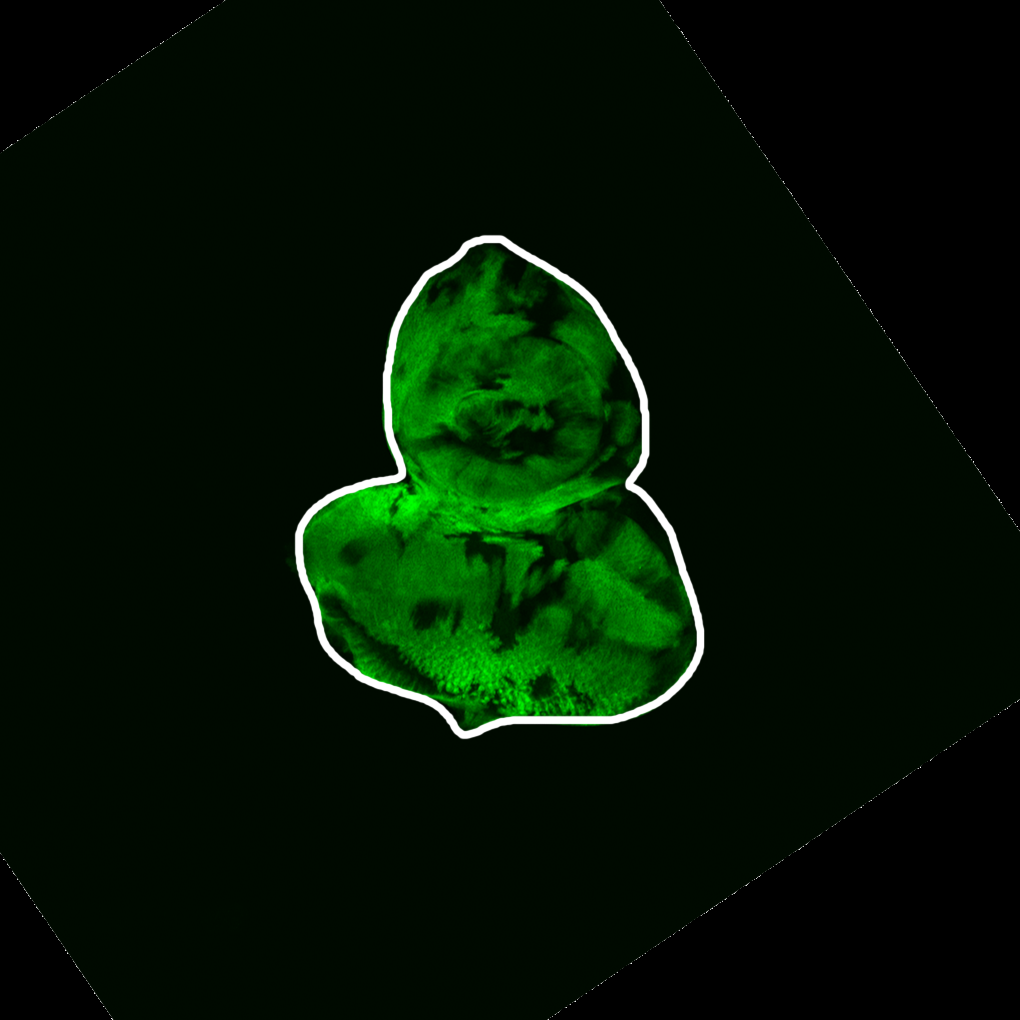

Supplement: Supplementary file 5 — Source data Fig. 1 [file 44318_2025_489_MOESM5_ESM.zip › Figure 1A/5-1 rotated and cut image with border line.tif]

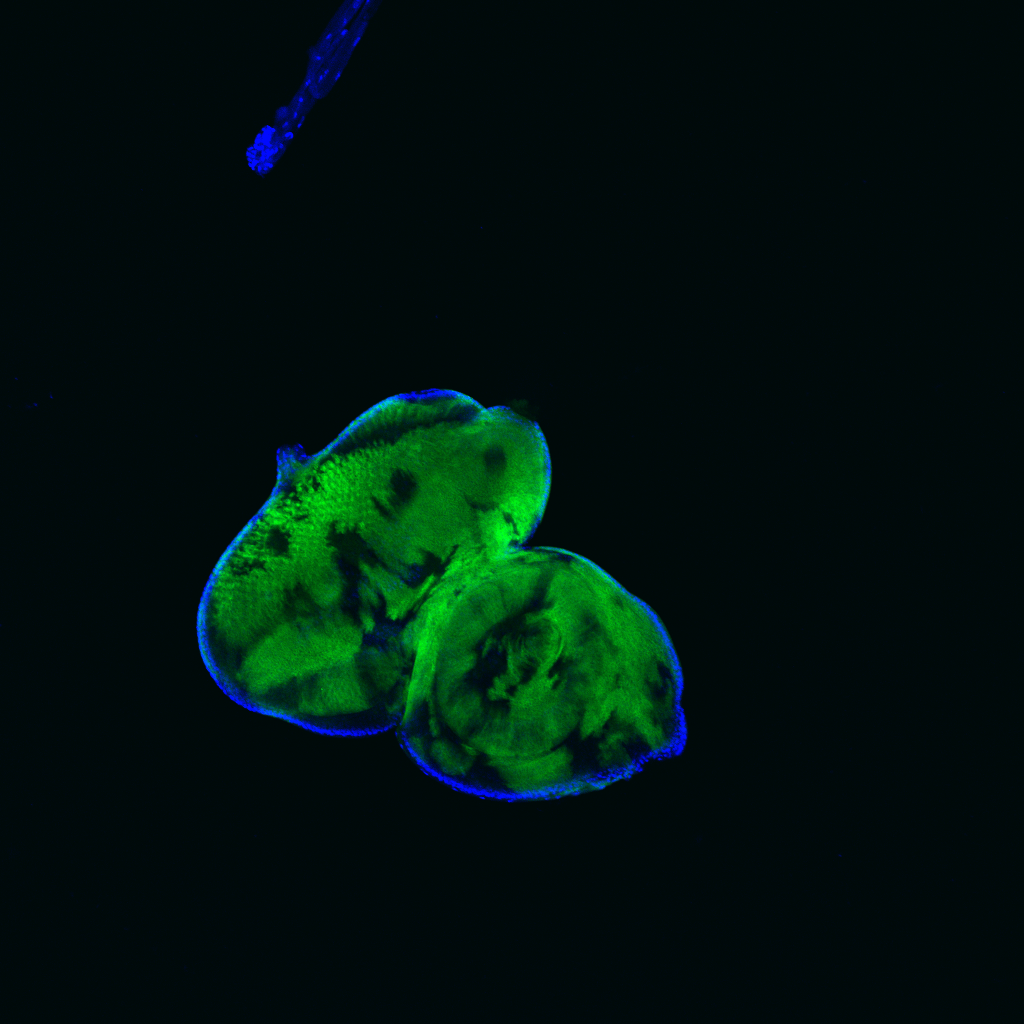

Supplement: Supplementary file 5 — Source data Fig. 1 [file 44318_2025_489_MOESM5_ESM.zip › Figure 1A/5-2 original image.tif]

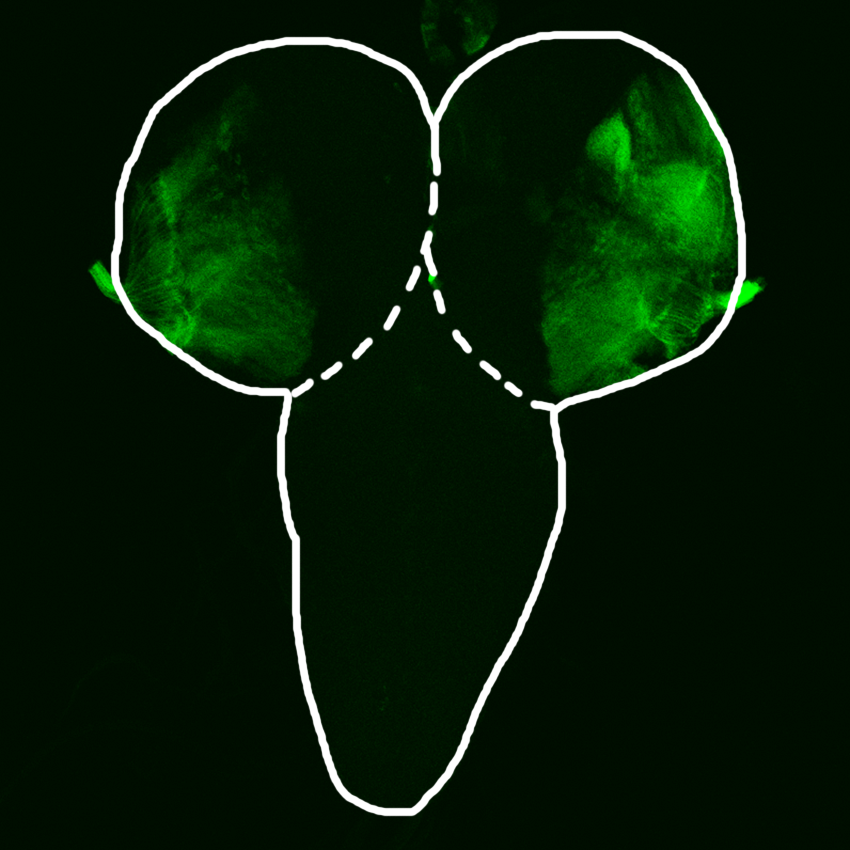

Supplement: Supplementary file 5 — Source data Fig. 1 [file 44318_2025_489_MOESM5_ESM.zip › Figure 1A/6-1 rotated and cut image with border line.tif]

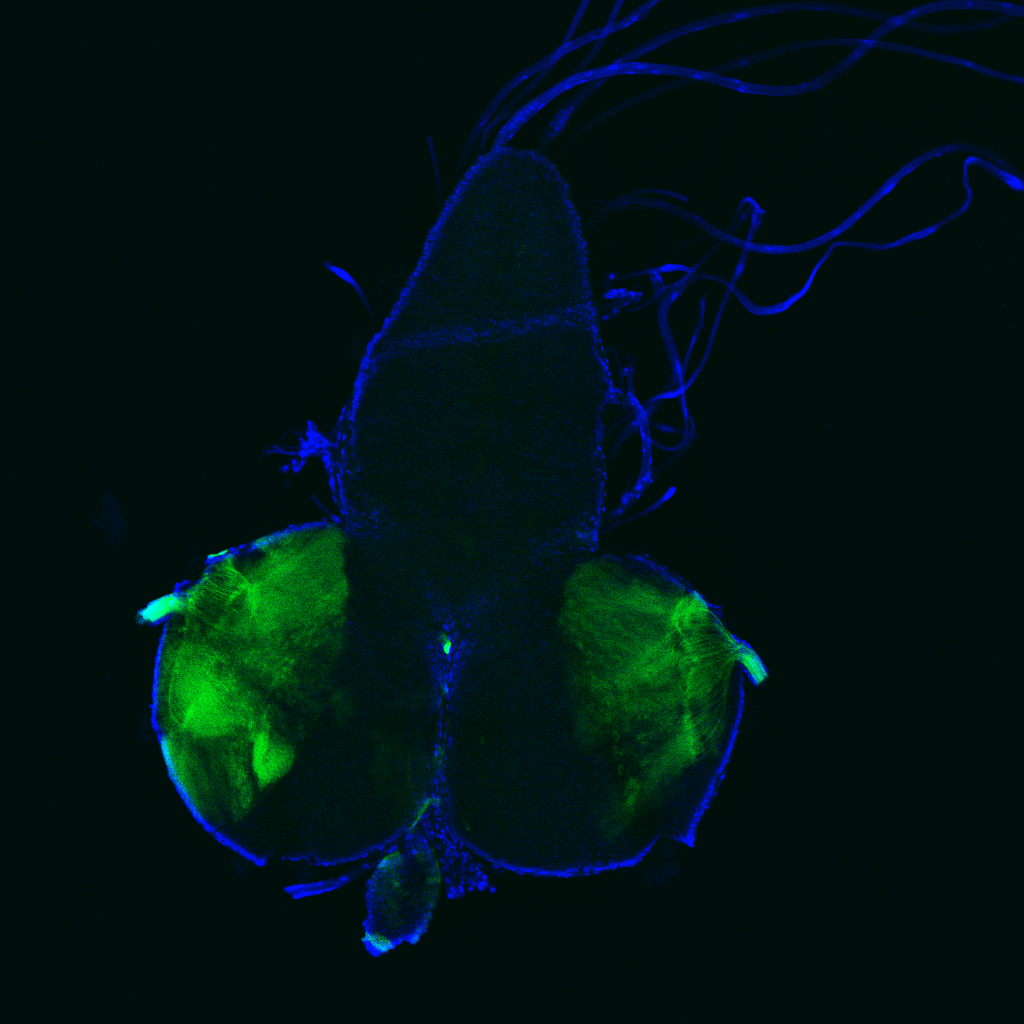

Supplement: Supplementary file 5 — Source data Fig. 1 [file 44318_2025_489_MOESM5_ESM.zip › Figure 1A/6-2 original image.tif]

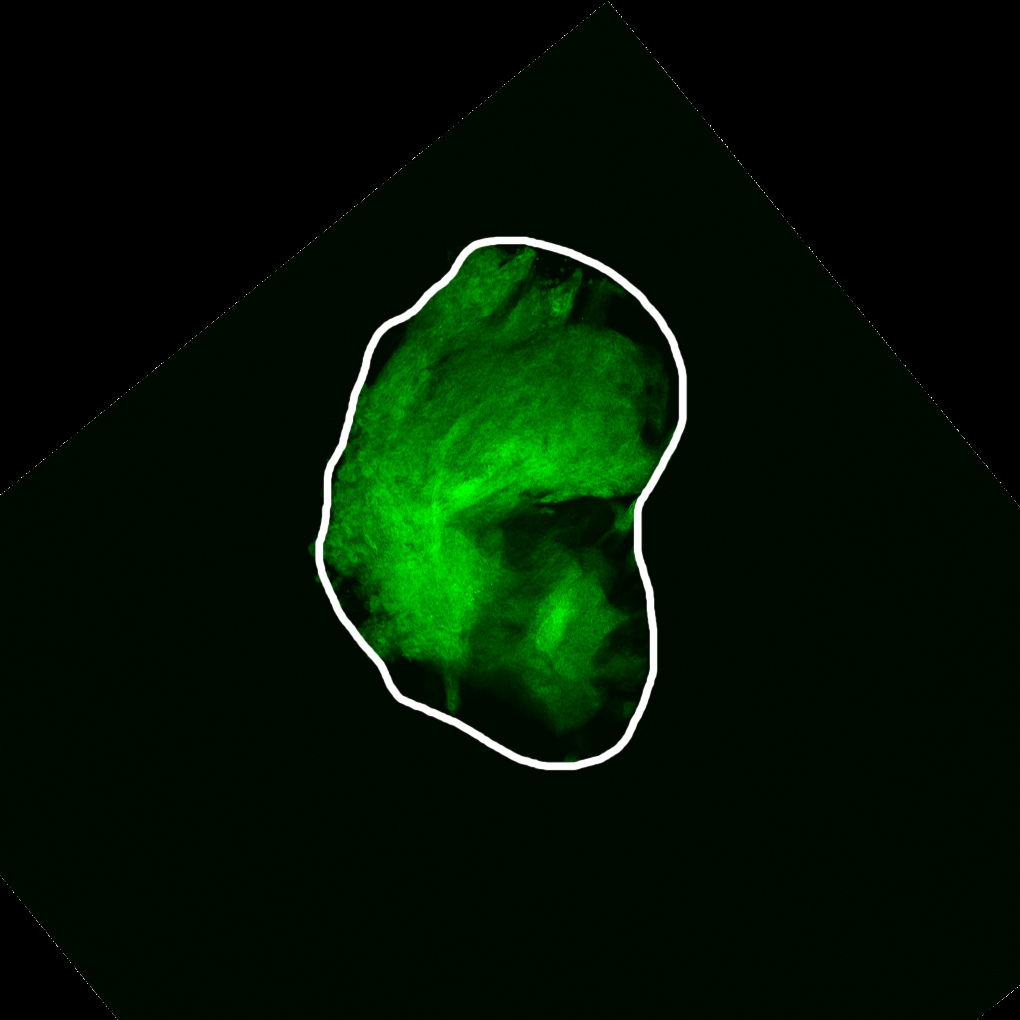

Supplement: Supplementary file 5 — Source data Fig. 1 [file 44318_2025_489_MOESM5_ESM.zip › Figure 1A/7-1 rotated and cut image with border line.tif]

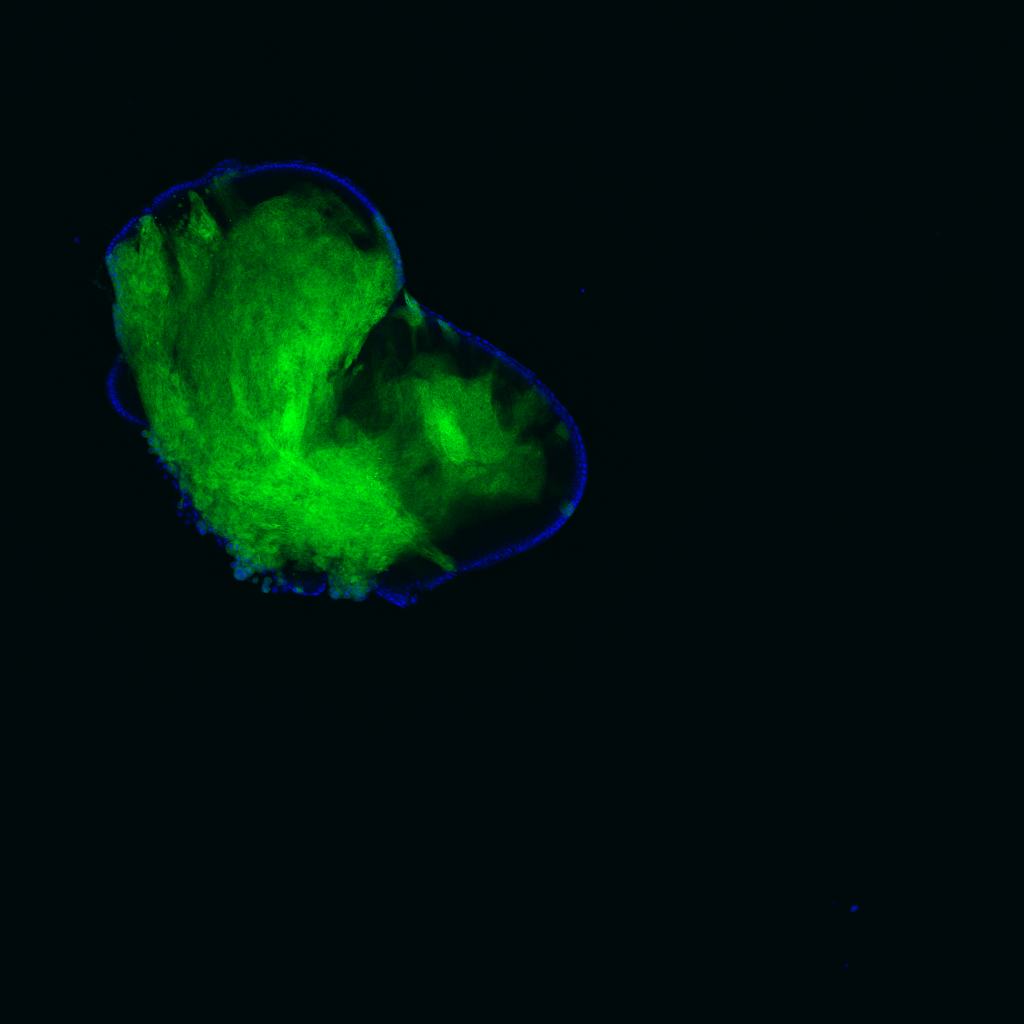

Supplement: Supplementary file 5 — Source data Fig. 1 [file 44318_2025_489_MOESM5_ESM.zip › Figure 1A/7-2 original image.tif]

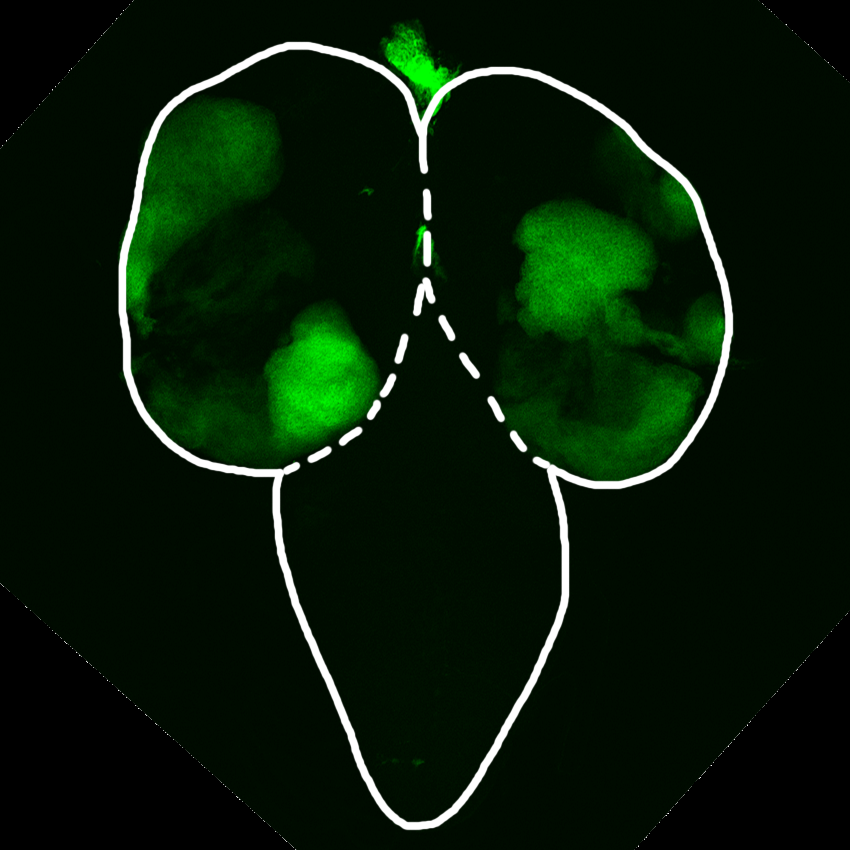

Supplement: Supplementary file 5 — Source data Fig. 1 [file 44318_2025_489_MOESM5_ESM.zip › Figure 1A/8-1 rotated and cut image with border line.tif]

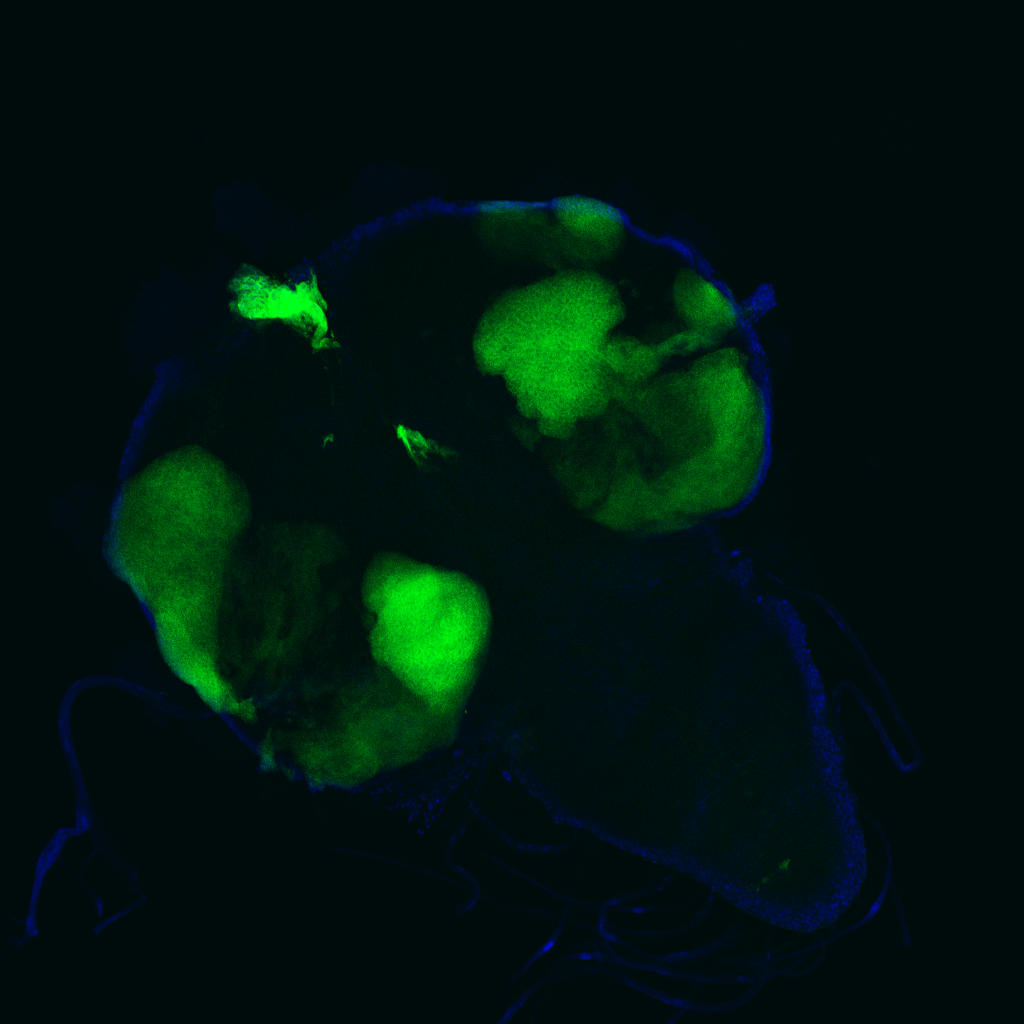

Supplement: Supplementary file 5 — Source data Fig. 1 [file 44318_2025_489_MOESM5_ESM.zip › Figure 1A/8-2 original image.tif]

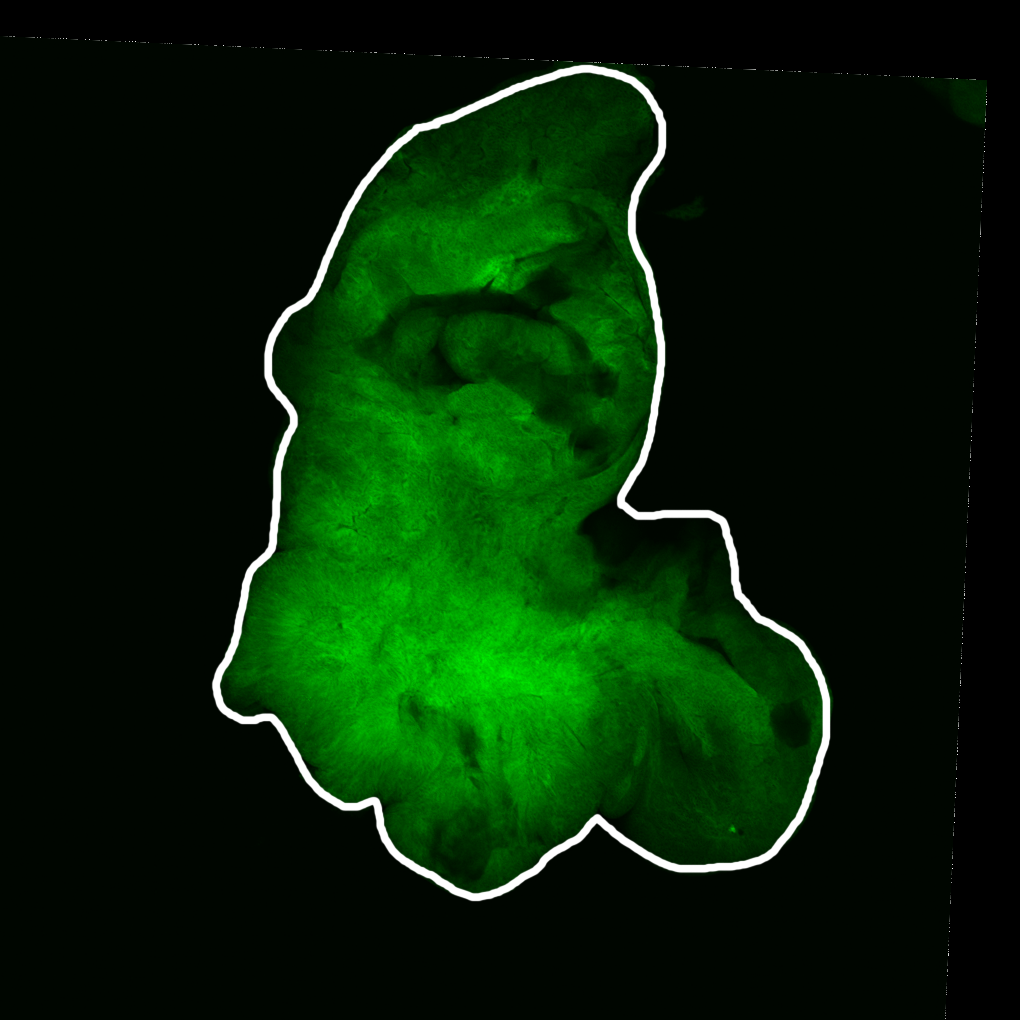

Supplement: Supplementary file 5 — Source data Fig. 1 [file 44318_2025_489_MOESM5_ESM.zip › Figure 1A/9-1 rotated and cut image with border line.tif]

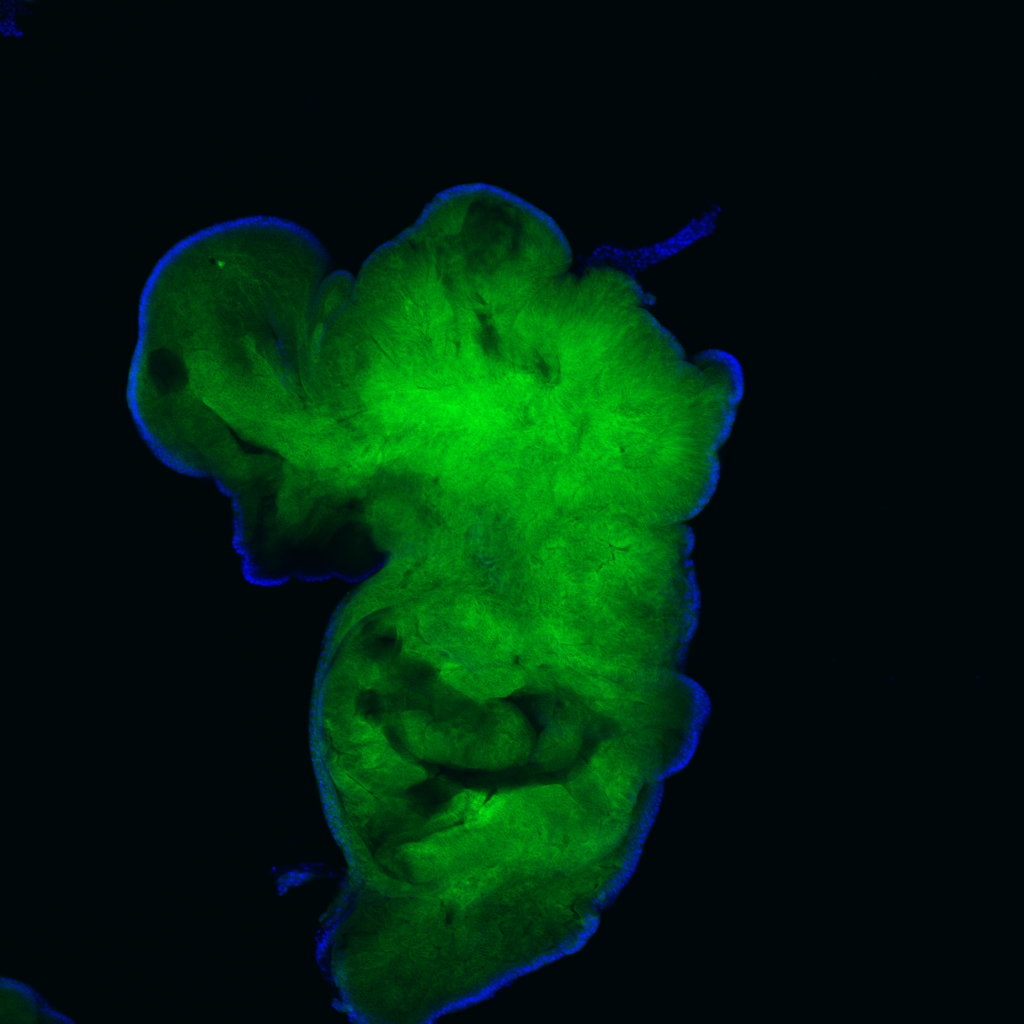

Supplement: Supplementary file 5 — Source data Fig. 1 [file 44318_2025_489_MOESM5_ESM.zip › Figure 1A/9-2 original image.tif]

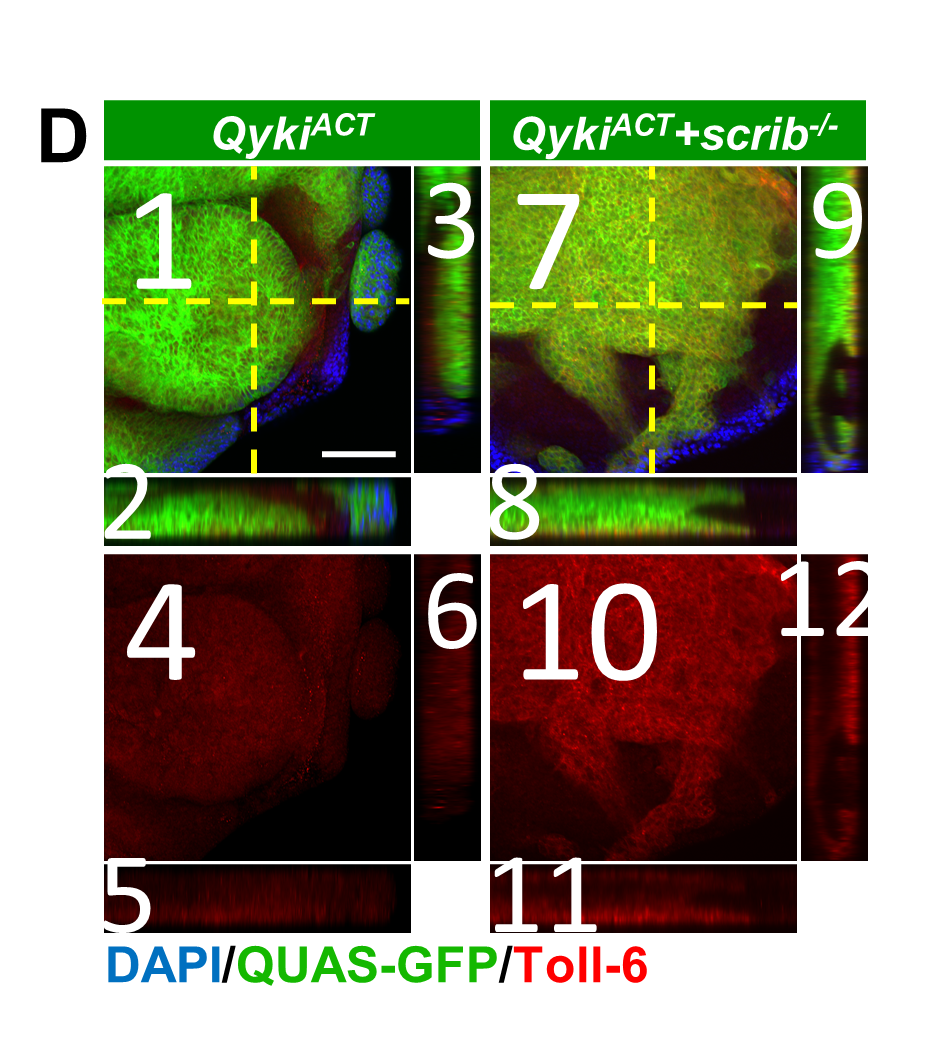

Supplement: Supplementary file 5 — Source data Fig. 1 [file 44318_2025_489_MOESM5_ESM.zip › Figure 1D/0 paper Figure 1D with provided image sequence.tif]

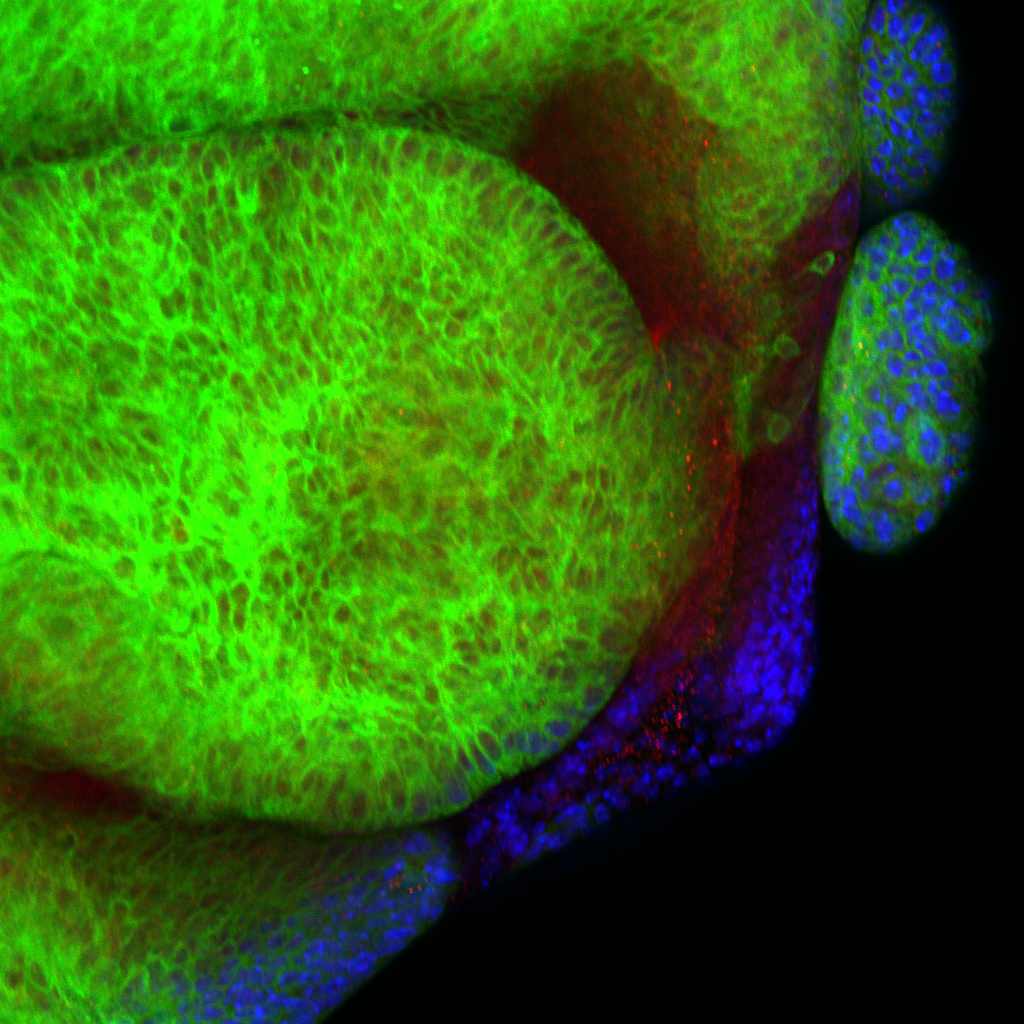

Supplement: Supplementary file 5 — Source data Fig. 1 [file 44318_2025_489_MOESM5_ESM.zip › Figure 1D/1 original image.tif]

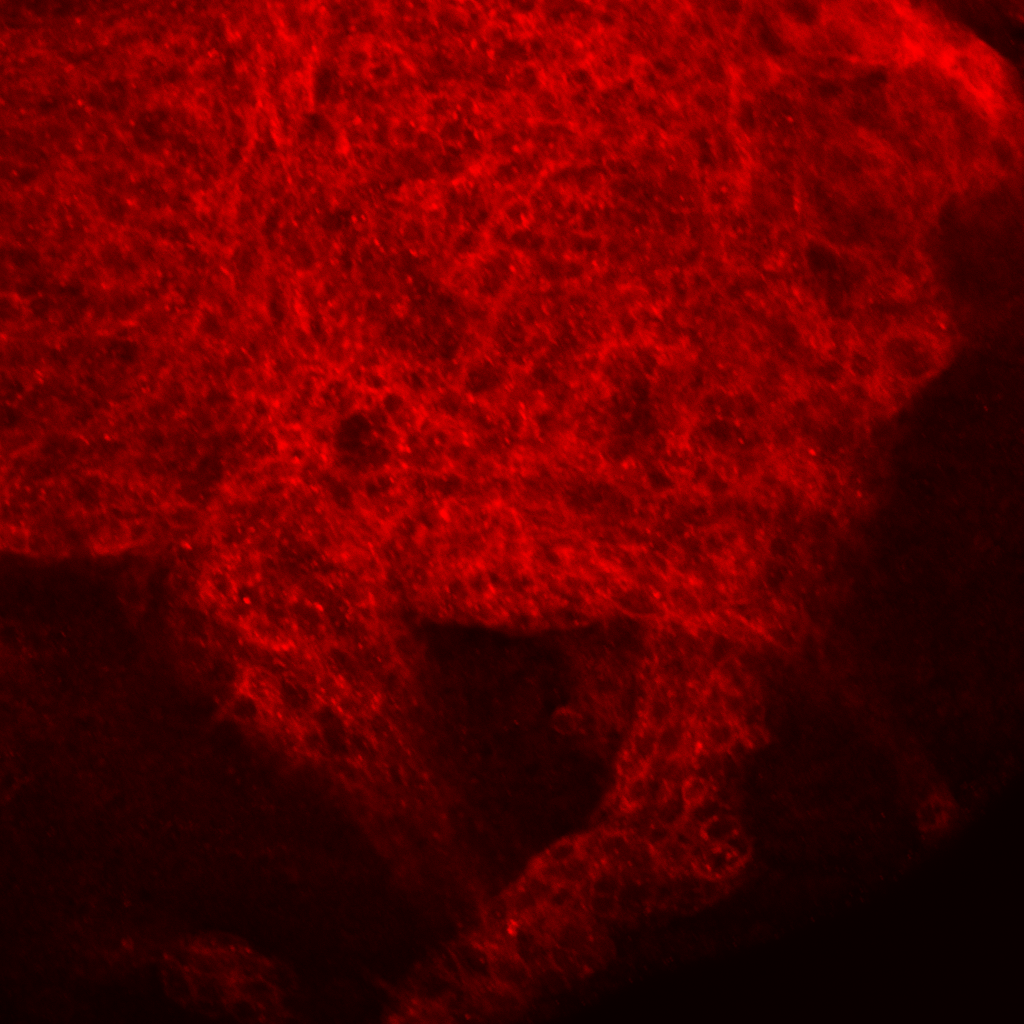

Supplement: Supplementary file 5 — Source data Fig. 1 [file 44318_2025_489_MOESM5_ESM.zip › Figure 1D/10 original image.tif]

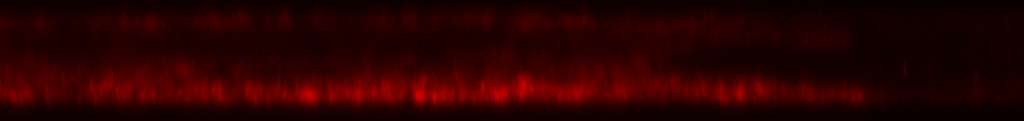

Supplement: Supplementary file 5 — Source data Fig. 1 [file 44318_2025_489_MOESM5_ESM.zip › Figure 1D/11 original image.tif]

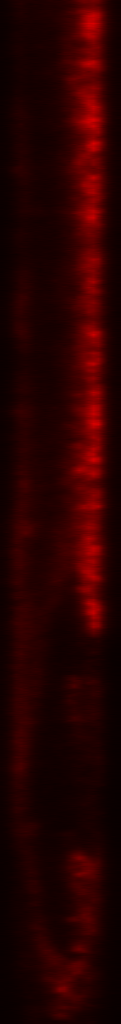

Supplement: Supplementary file 5 — Source data Fig. 1 [file 44318_2025_489_MOESM5_ESM.zip › Figure 1D/12 original image.tif]

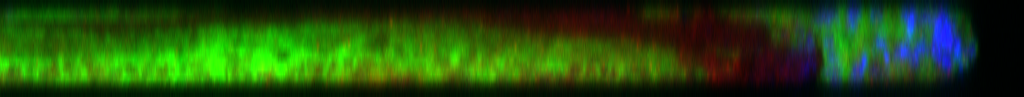

Supplement: Supplementary file 5 — Source data Fig. 1 [file 44318_2025_489_MOESM5_ESM.zip › Figure 1D/2 original image.tif]

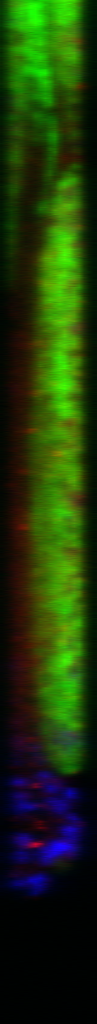

Supplement: Supplementary file 5 — Source data Fig. 1 [file 44318_2025_489_MOESM5_ESM.zip › Figure 1D/3 original image.tif]

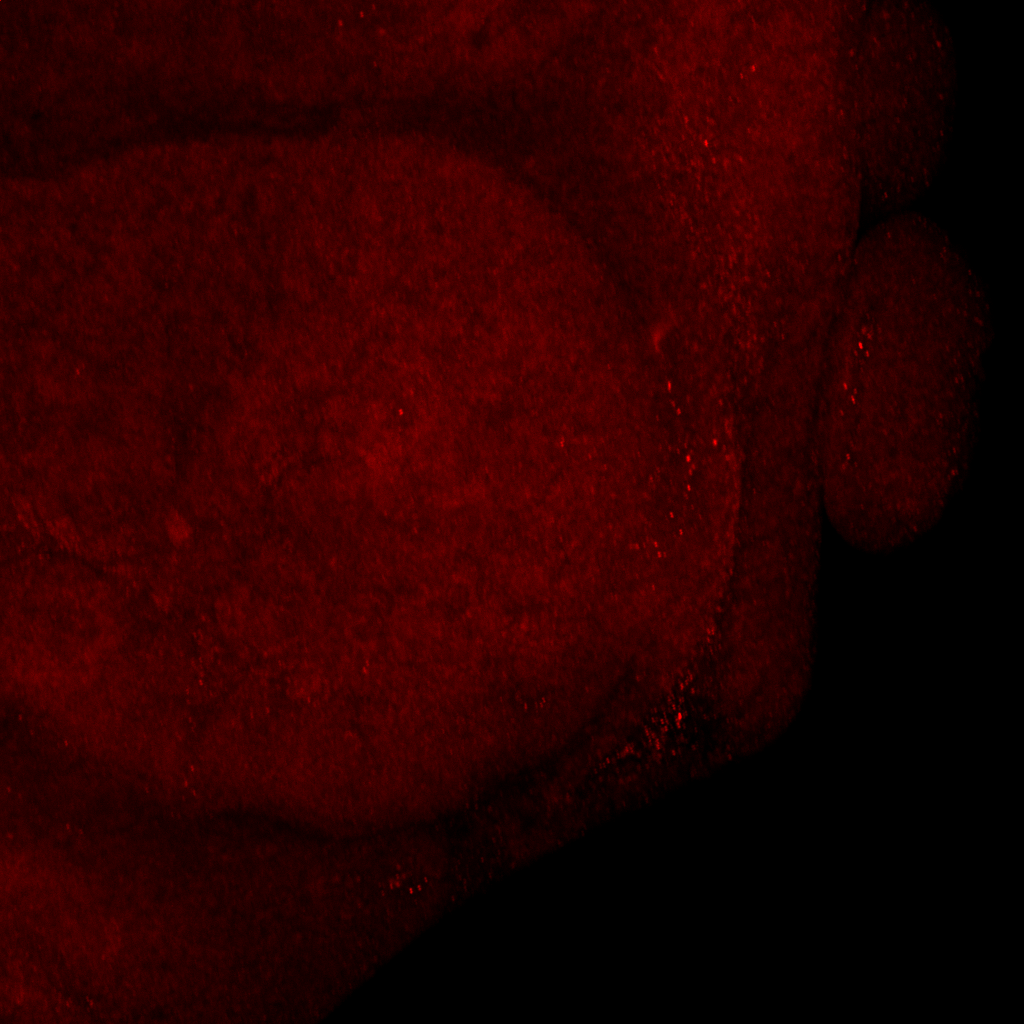

Supplement: Supplementary file 5 — Source data Fig. 1 [file 44318_2025_489_MOESM5_ESM.zip › Figure 1D/4 original image.tif]

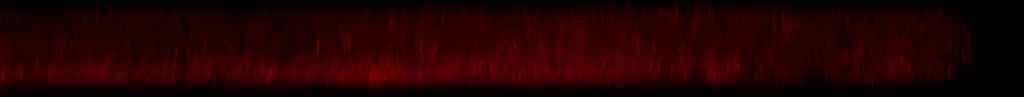

Supplement: Supplementary file 5 — Source data Fig. 1 [file 44318_2025_489_MOESM5_ESM.zip › Figure 1D/5 original image.tif]

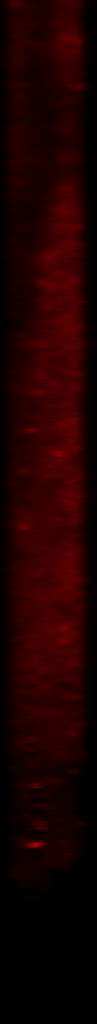

Supplement: Supplementary file 5 — Source data Fig. 1 [file 44318_2025_489_MOESM5_ESM.zip › Figure 1D/6 original image.tif]

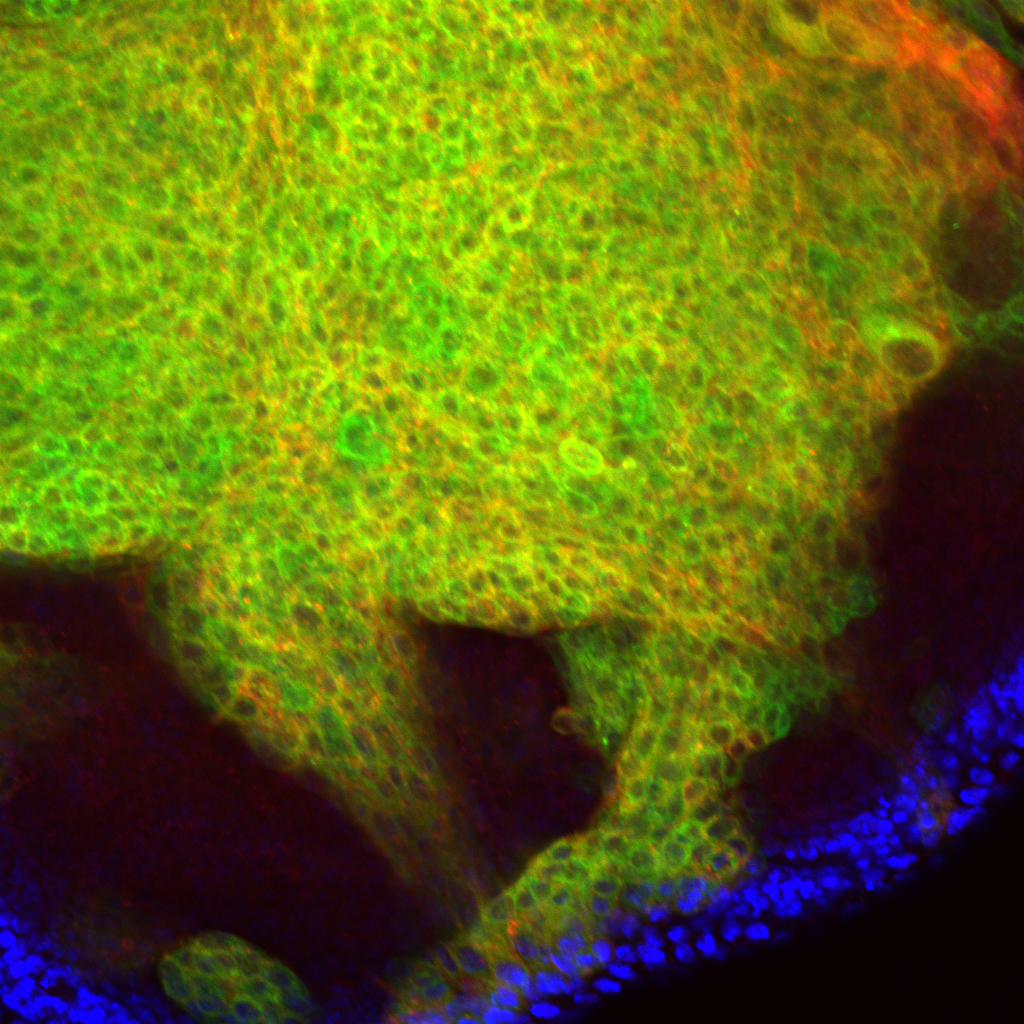

Supplement: Supplementary file 5 — Source data Fig. 1 [file 44318_2025_489_MOESM5_ESM.zip › Figure 1D/7 original image.tif]

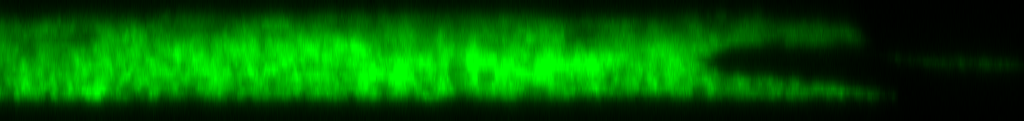

Supplement: Supplementary file 5 — Source data Fig. 1 [file 44318_2025_489_MOESM5_ESM.zip › Figure 1D/8 original image.tif]

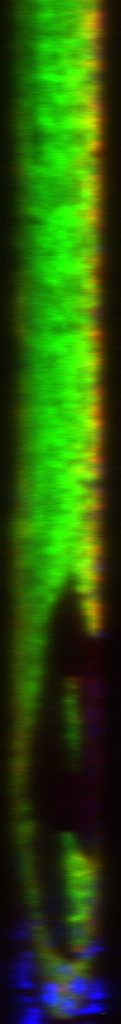

Supplement: Supplementary file 5 — Source data Fig. 1 [file 44318_2025_489_MOESM5_ESM.zip › Figure 1D/9 original image.tif]

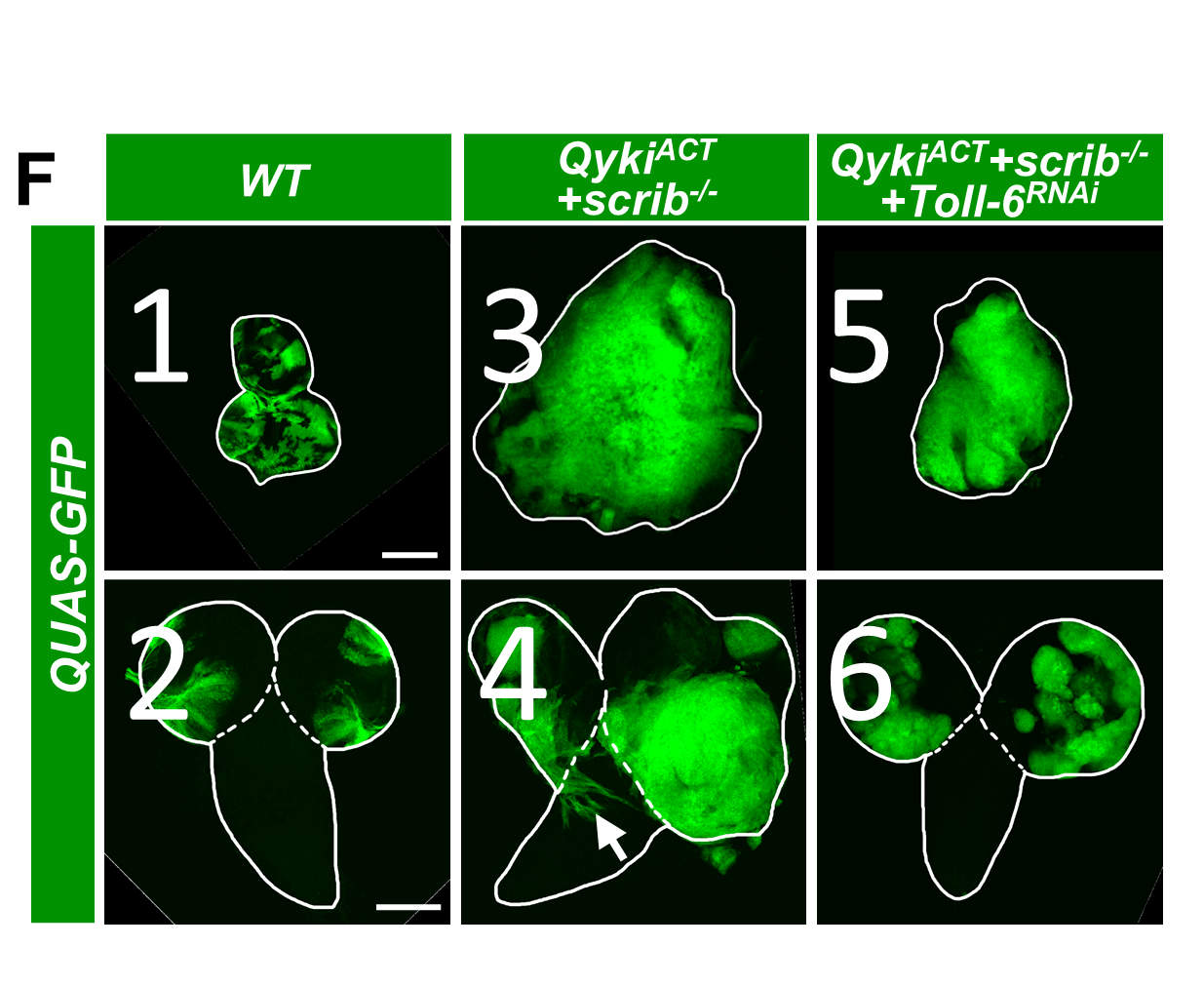

Supplement: Supplementary file 5 — Source data Fig. 1 [file 44318_2025_489_MOESM5_ESM.zip › Figure 1F/0 paper Figure 1F with provided image sequence.tif]

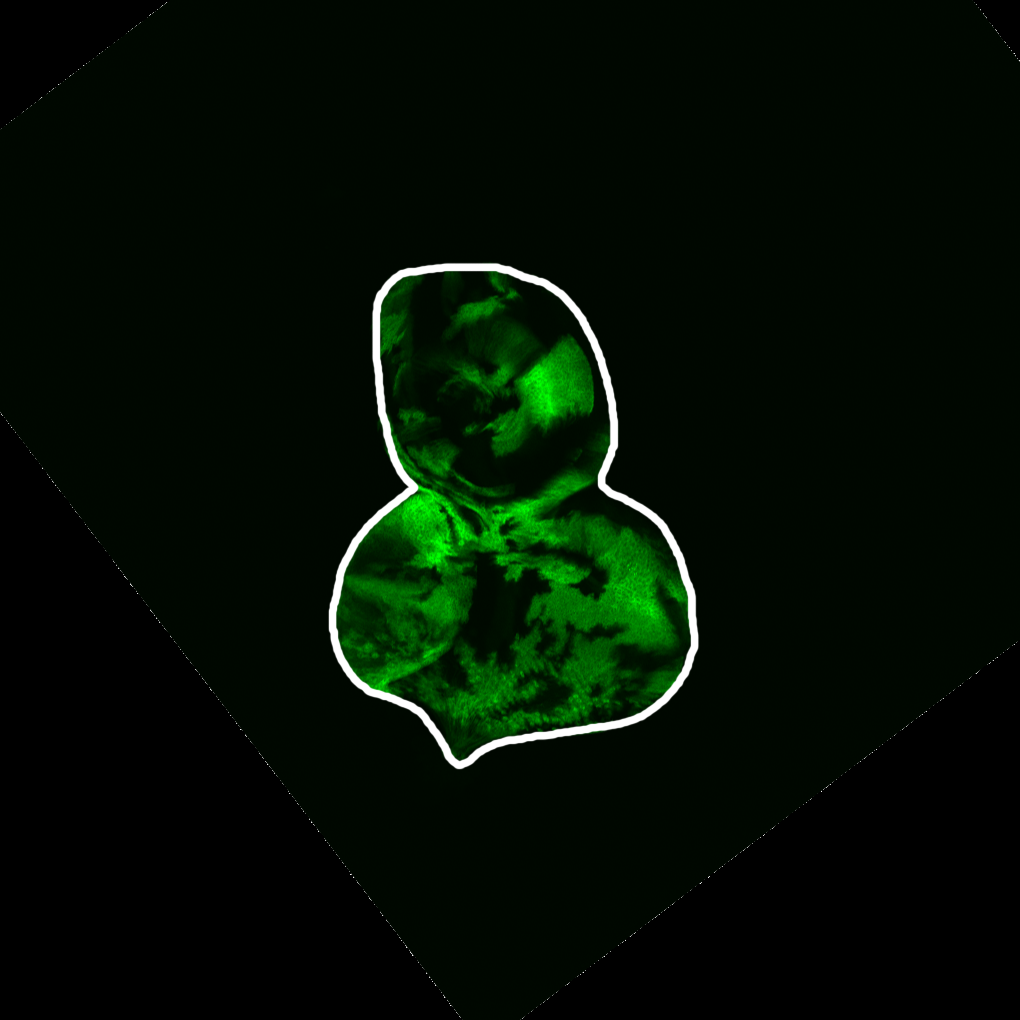

Supplement: Supplementary file 5 — Source data Fig. 1 [file 44318_2025_489_MOESM5_ESM.zip › Figure 1F/1-1 rotated and cut image with border line.tif]

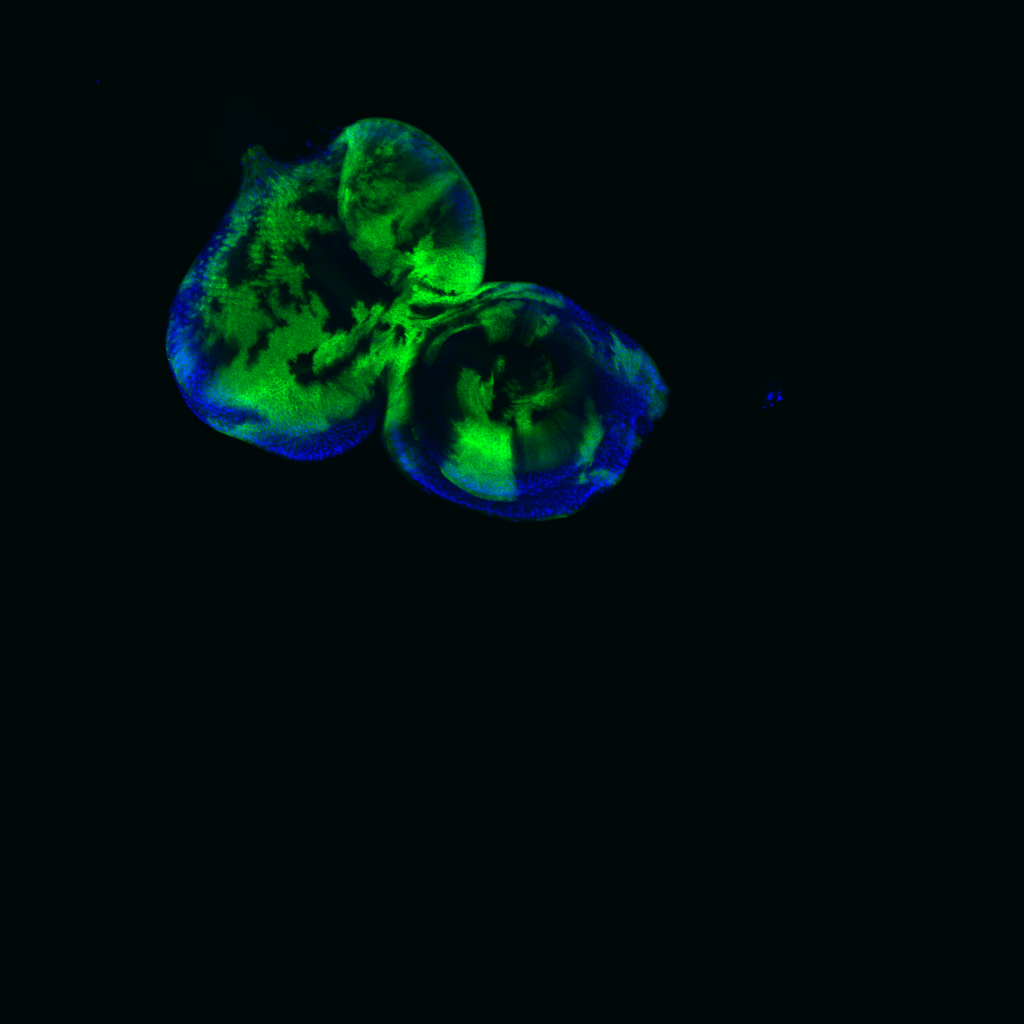

Supplement: Supplementary file 5 — Source data Fig. 1 [file 44318_2025_489_MOESM5_ESM.zip › Figure 1F/1-2 original image.tif]

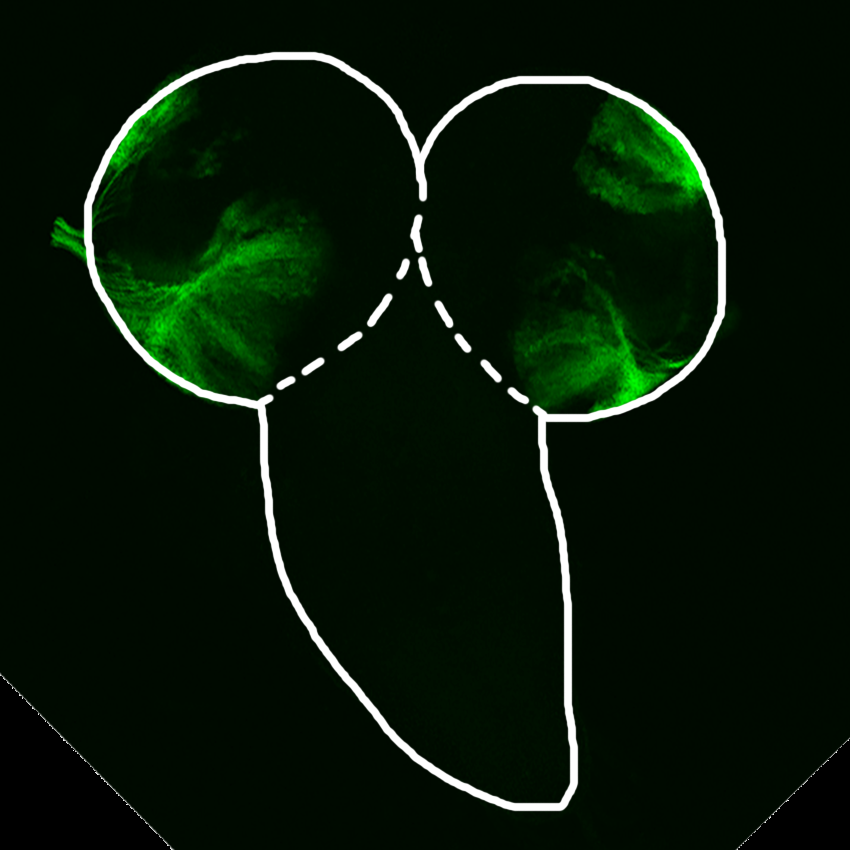

Supplement: Supplementary file 5 — Source data Fig. 1 [file 44318_2025_489_MOESM5_ESM.zip › Figure 1F/2-1 rotated and cut image with border line.tif]

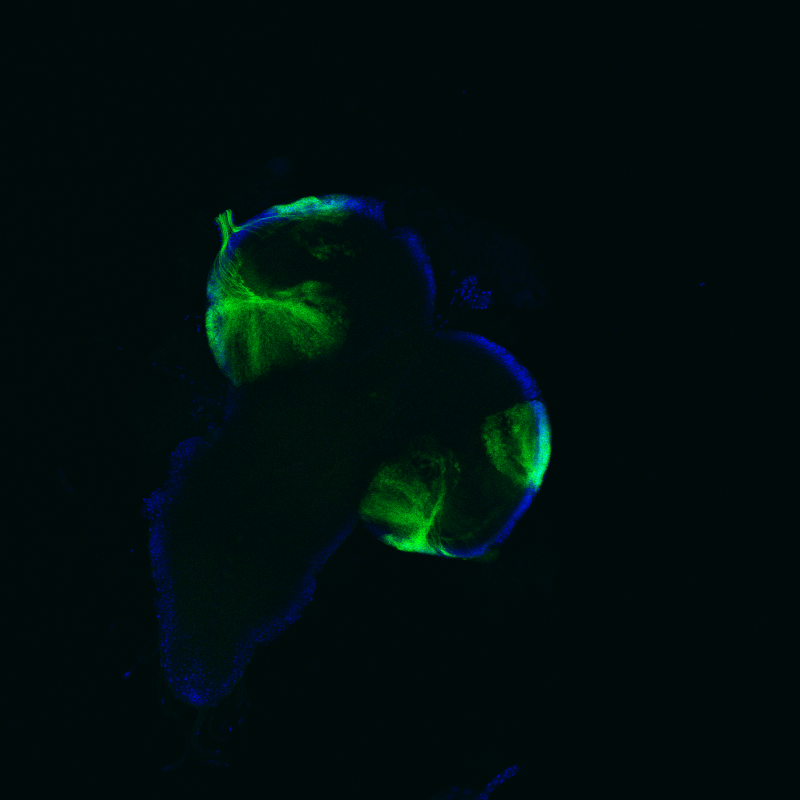

Supplement: Supplementary file 5 — Source data Fig. 1 [file 44318_2025_489_MOESM5_ESM.zip › Figure 1F/2-2 original image.tif]

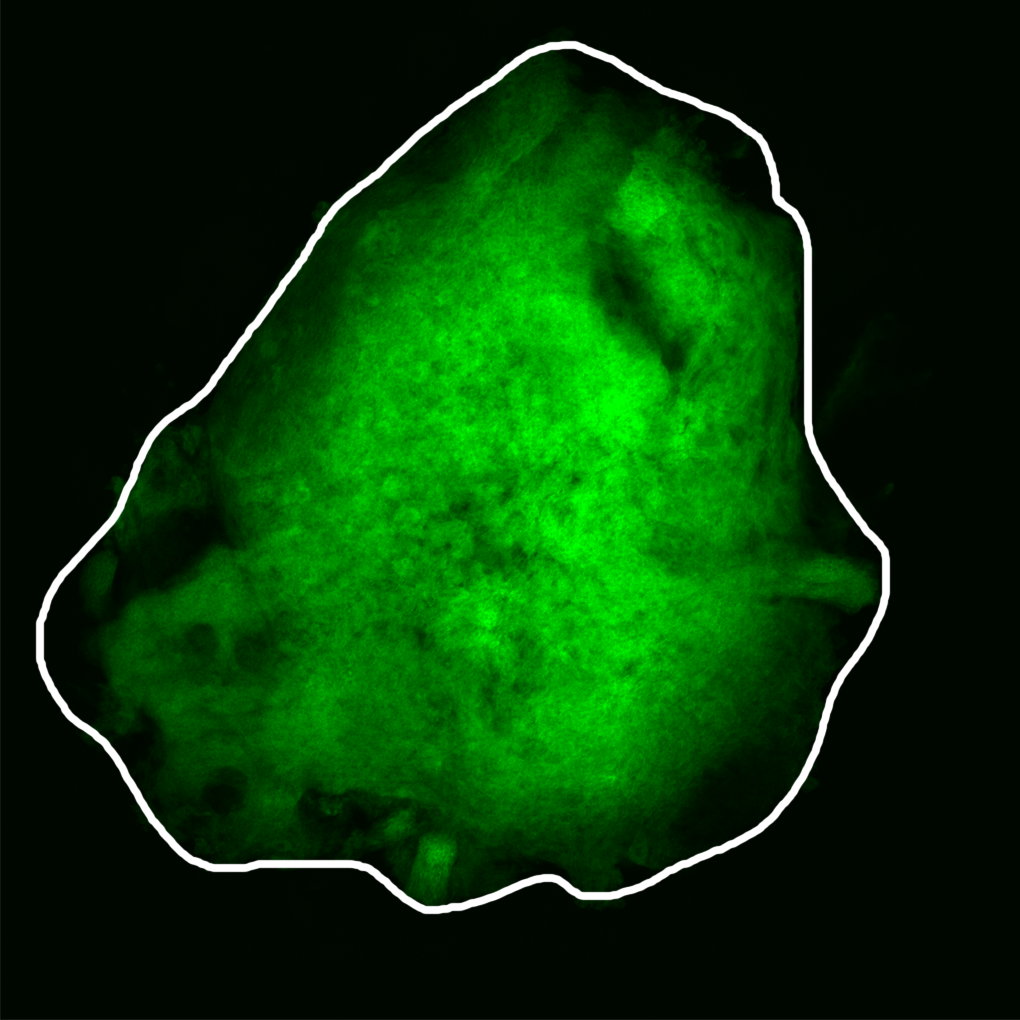

Supplement: Supplementary file 5 — Source data Fig. 1 [file 44318_2025_489_MOESM5_ESM.zip › Figure 1F/3-1 rotated and cut image with border line.tif]

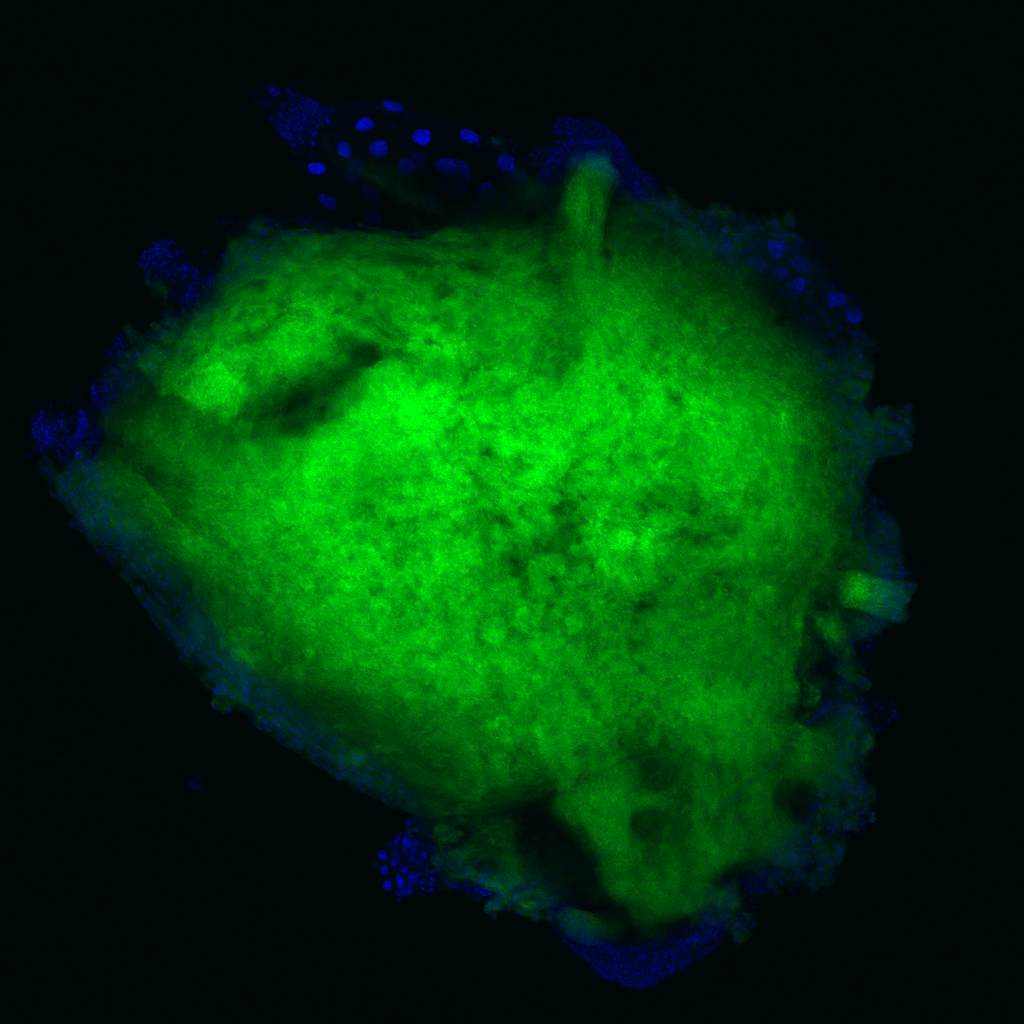

Supplement: Supplementary file 5 — Source data Fig. 1 [file 44318_2025_489_MOESM5_ESM.zip › Figure 1F/3-2 original image.tif]

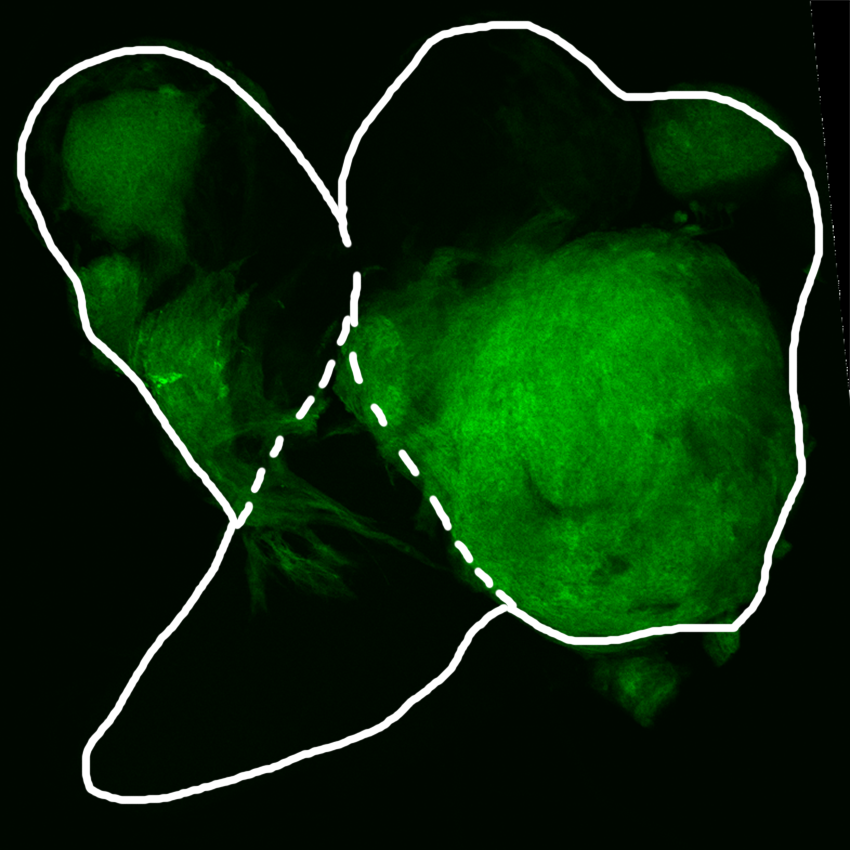

Supplement: Supplementary file 5 — Source data Fig. 1 [file 44318_2025_489_MOESM5_ESM.zip › Figure 1F/4-1 rotated and cut image with border line.tif]

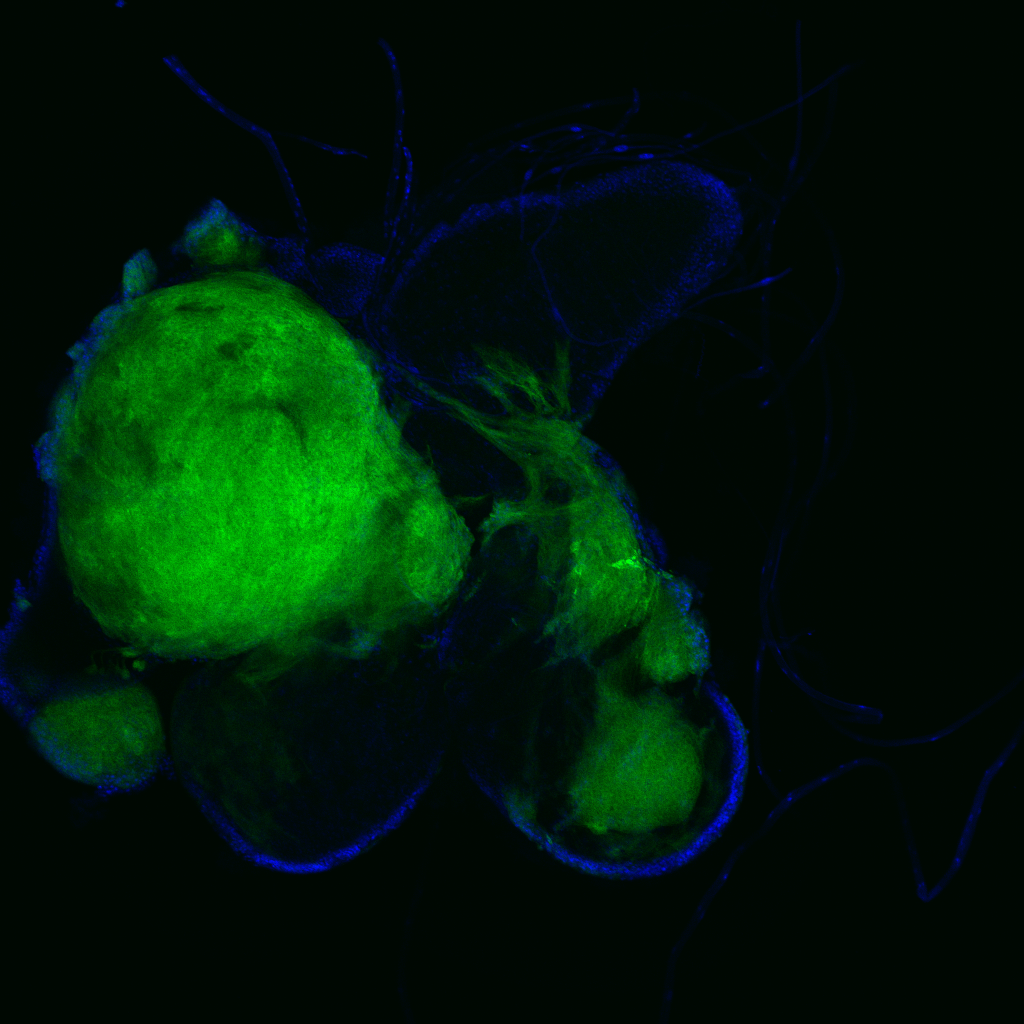

Supplement: Supplementary file 5 — Source data Fig. 1 [file 44318_2025_489_MOESM5_ESM.zip › Figure 1F/4-2 original image.tif]

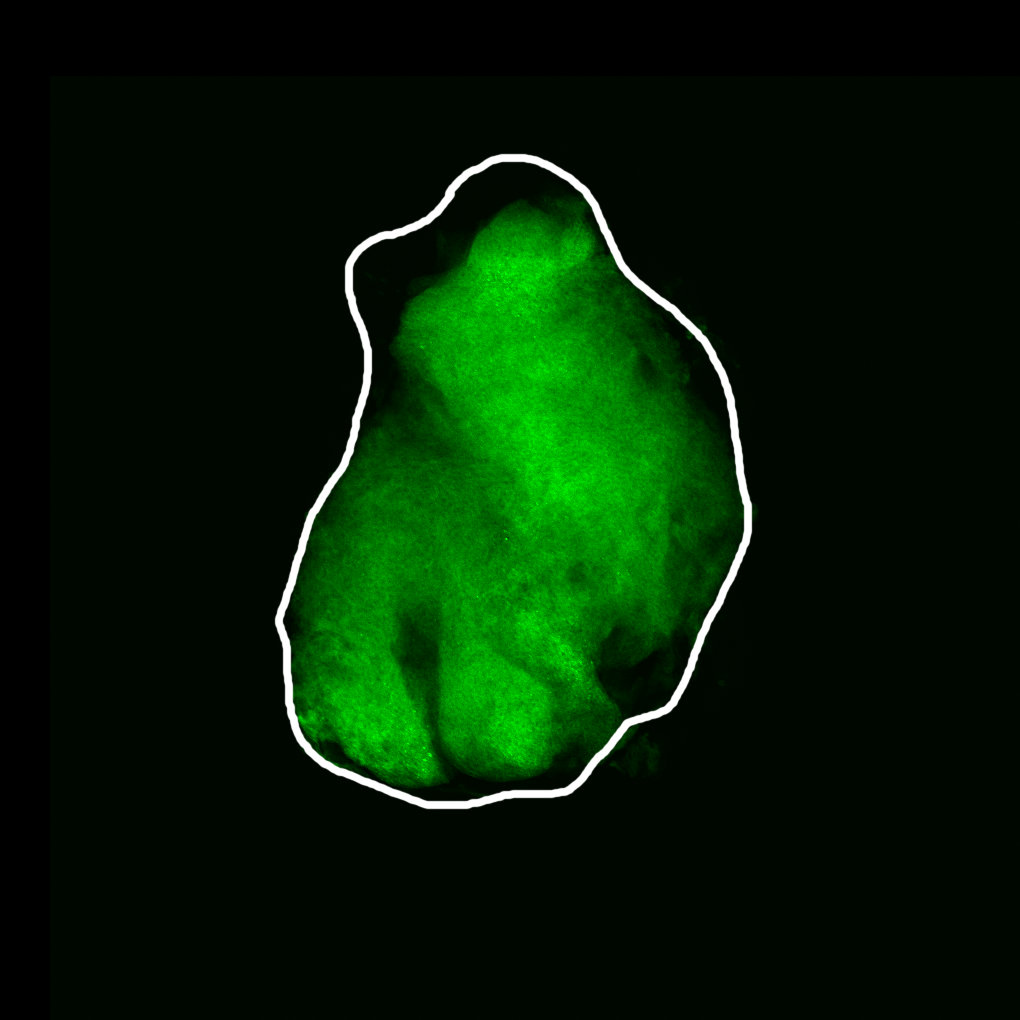

Supplement: Supplementary file 5 — Source data Fig. 1 [file 44318_2025_489_MOESM5_ESM.zip › Figure 1F/5-1 rotated and cut image with border line.tif]

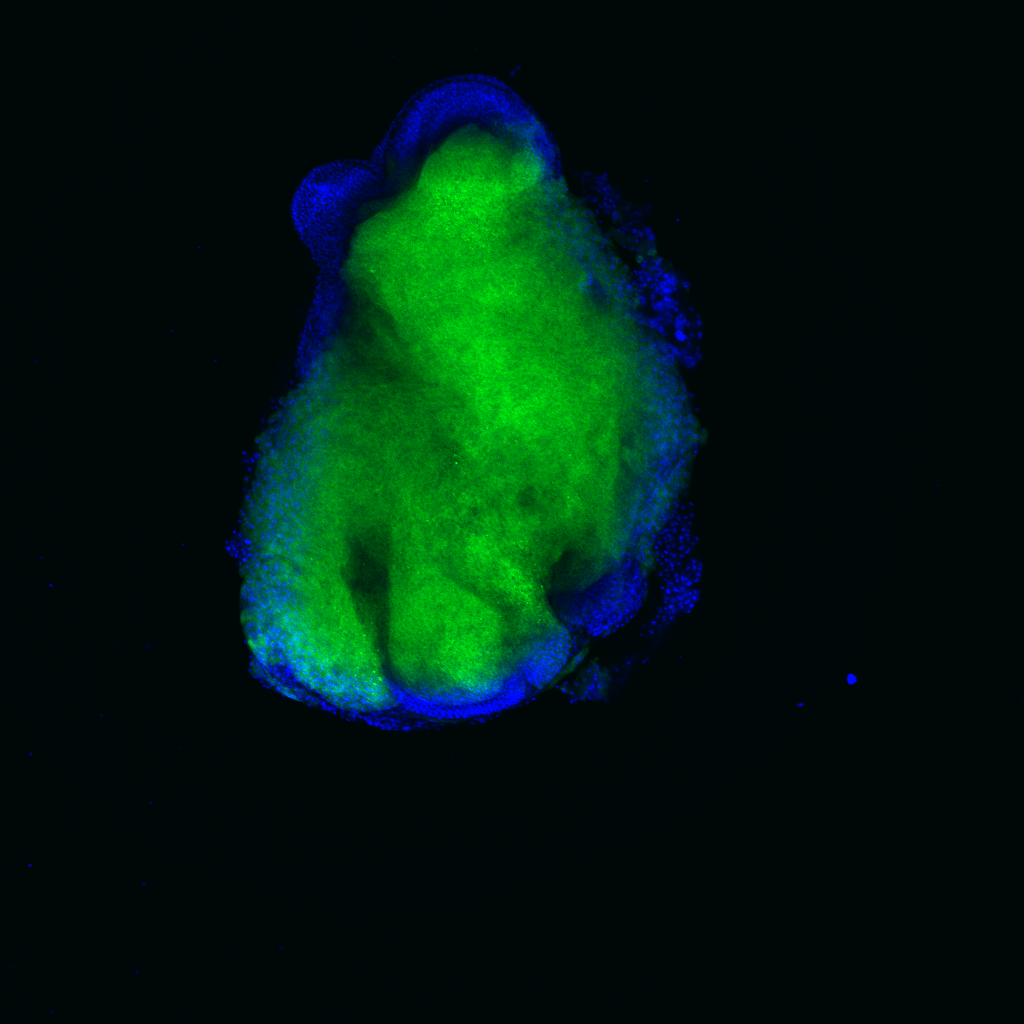

Supplement: Supplementary file 5 — Source data Fig. 1 [file 44318_2025_489_MOESM5_ESM.zip › Figure 1F/5-2 original image.tif]

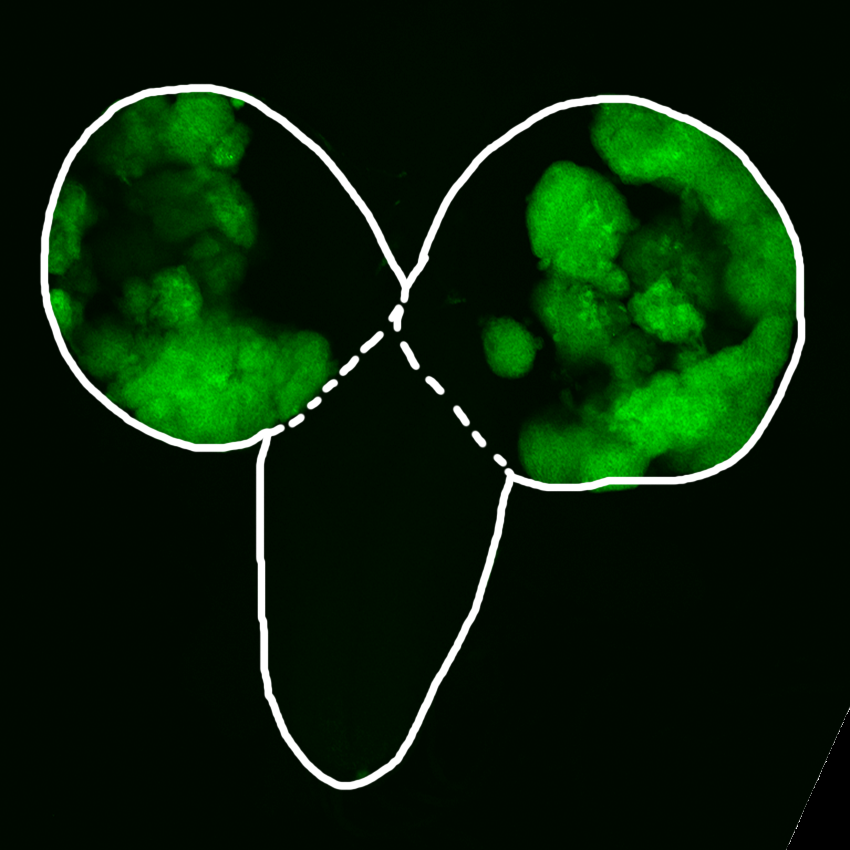

Supplement: Supplementary file 5 — Source data Fig. 1 [file 44318_2025_489_MOESM5_ESM.zip › Figure 1F/6-1 rotated and cut image with border line.tif]

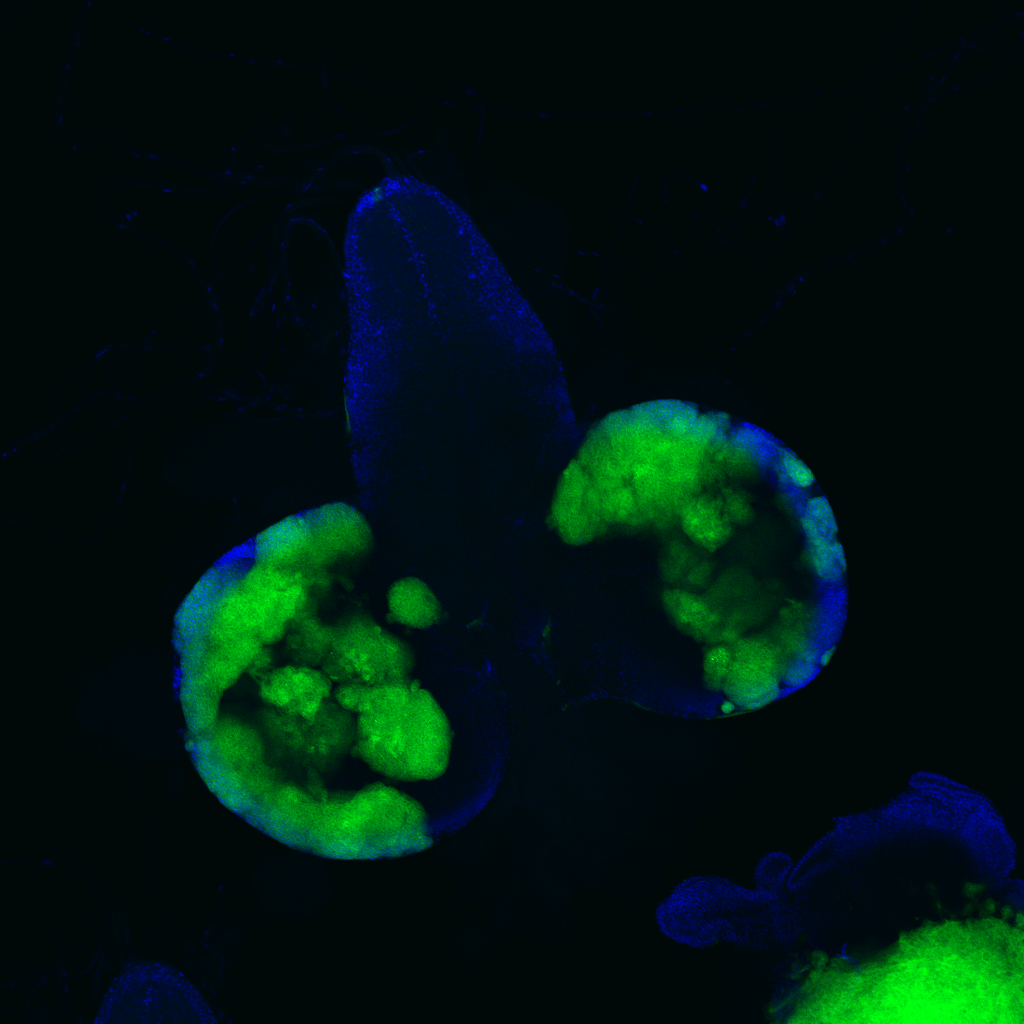

Supplement: Supplementary file 5 — Source data Fig. 1 [file 44318_2025_489_MOESM5_ESM.zip › Figure 1F/6-2 original image.tif]

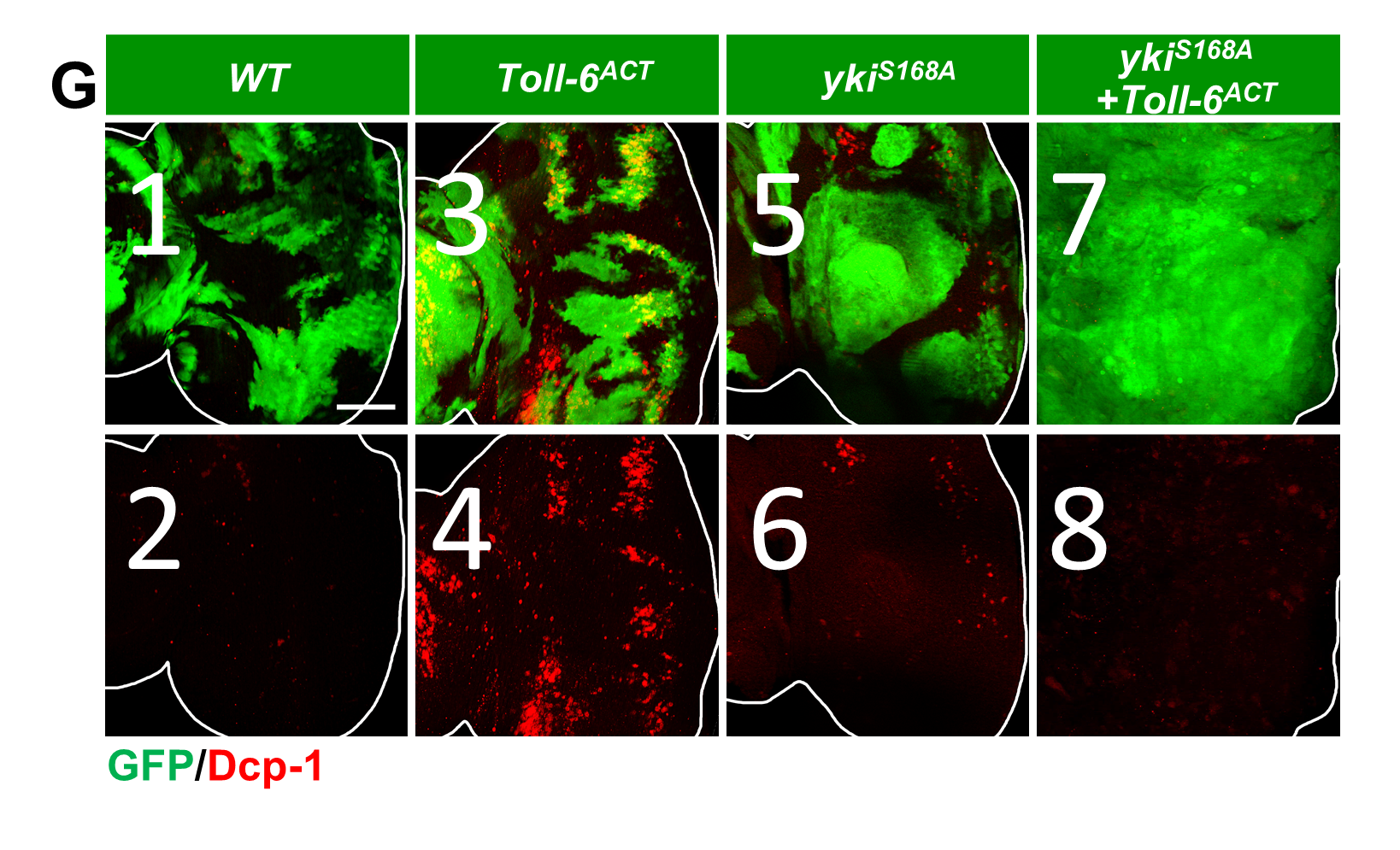

Supplement: Supplementary file 6 — Source data Fig. 2 [file 44318_2025_489_MOESM6_ESM.zip › Figure 2G/0 paper Figure 2G with provided image sequence.tif]
