# Supplementary figures and images for: Adipose tissue-secreted Spz5 promotes distal tumor progression via Toll-6-mediated Hh pathway activation in Drosophila (part 2 of 5)
Source: EMBO J. 2025 Jun 23;44(15):4301–30. doi: 10.1038/s44318-025-00489-y (PMC12317064; doi:10.1038/s44318-025-00489-y)

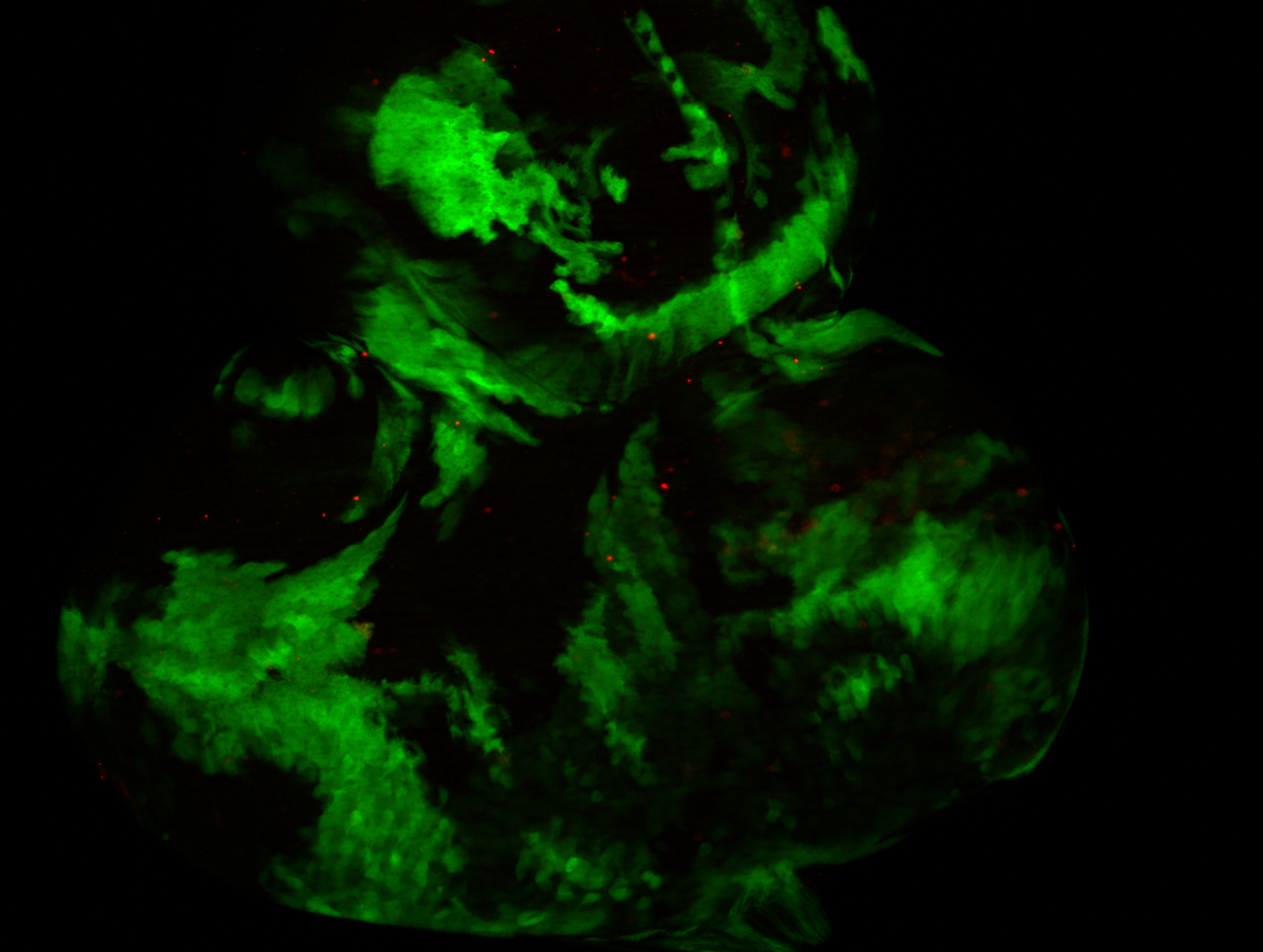

Supplement: Supplementary file 6 — Source data Fig. 2 [file 44318_2025_489_MOESM6_ESM.zip › Figure 2G/1 original image.tif]

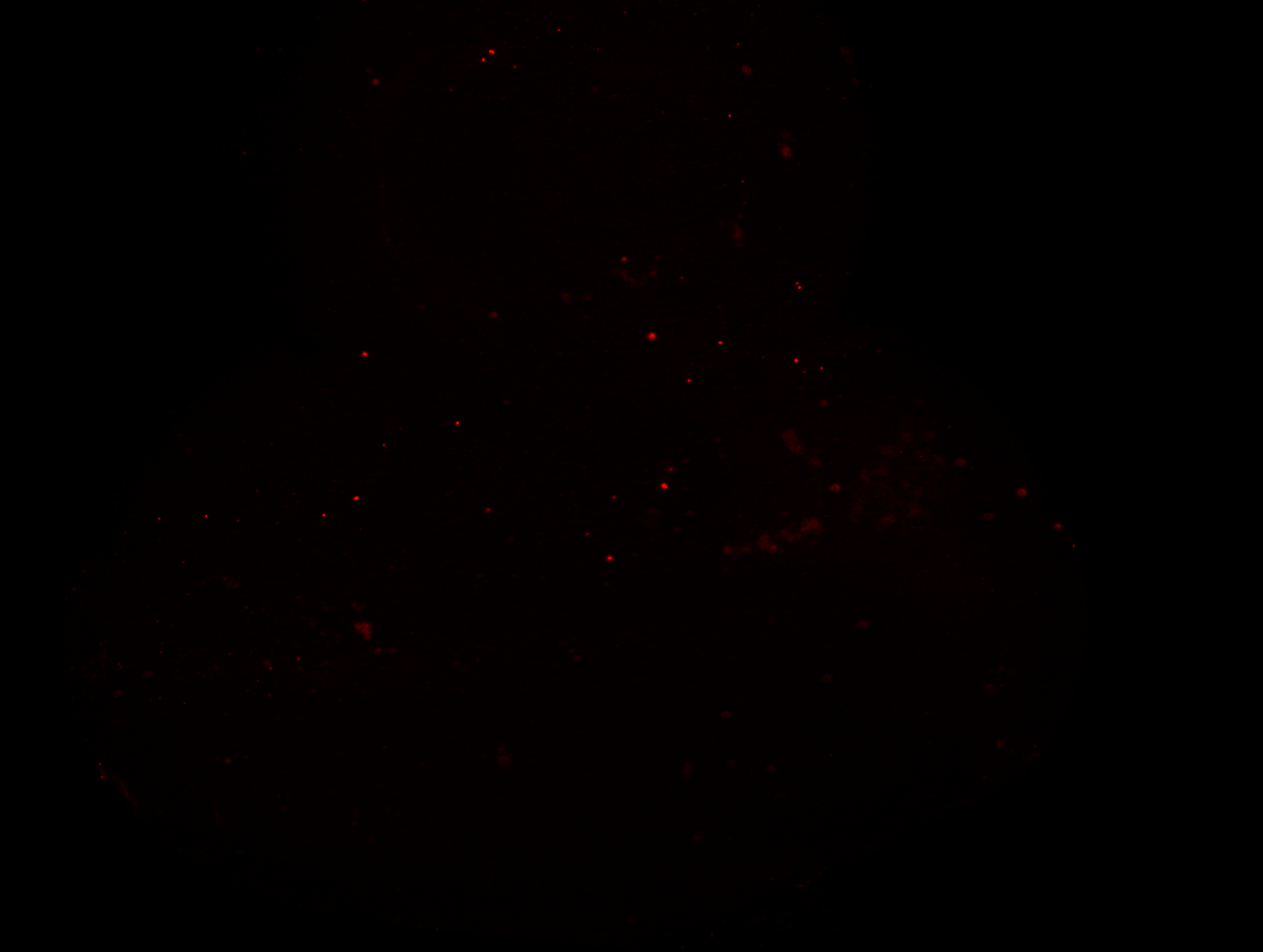

Supplement: Supplementary file 6 — Source data Fig. 2 [file 44318_2025_489_MOESM6_ESM.zip › Figure 2G/2 original image.tif]

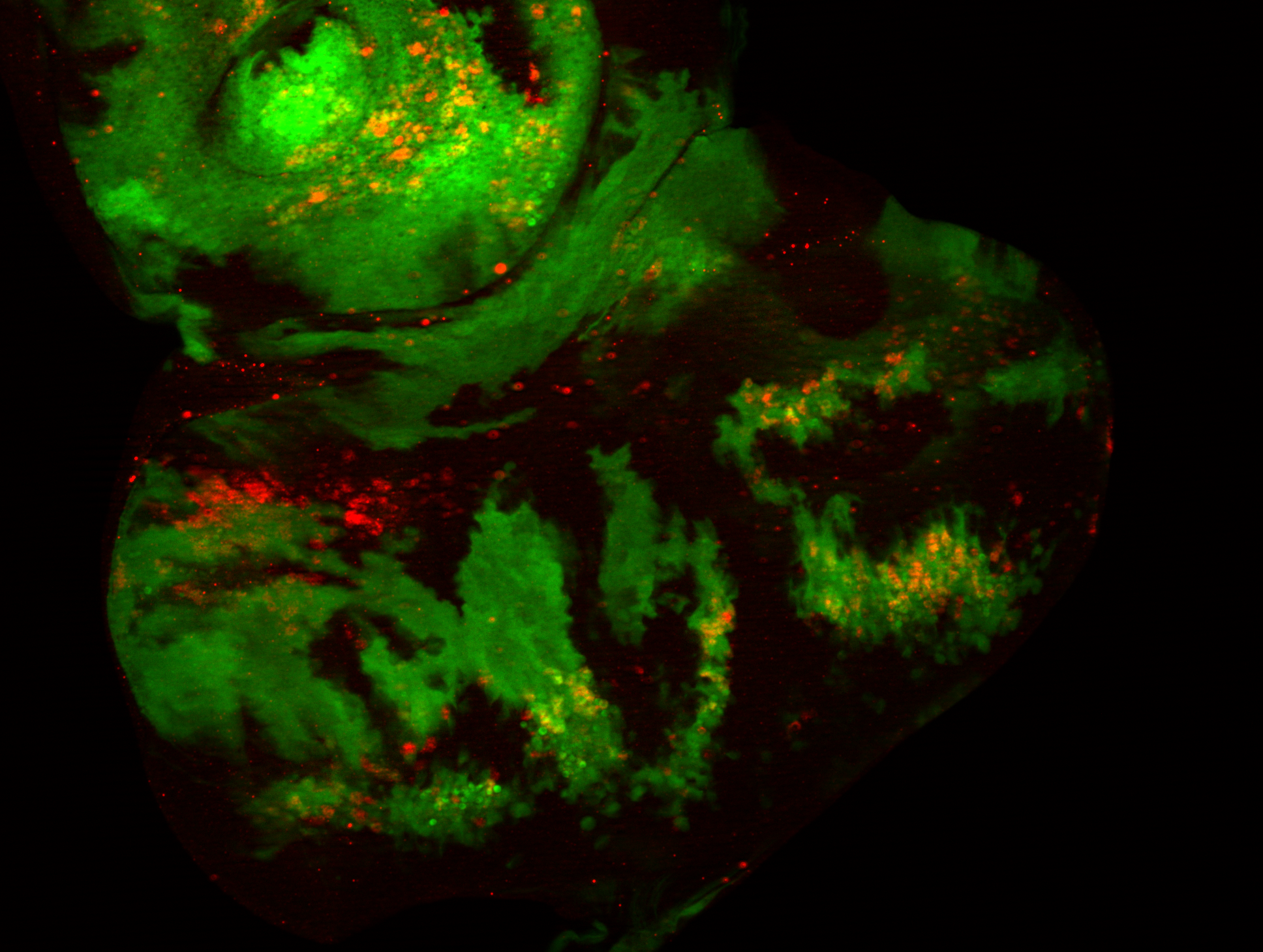

Supplement: Supplementary file 6 — Source data Fig. 2 [file 44318_2025_489_MOESM6_ESM.zip › Figure 2G/3 original image.tif]

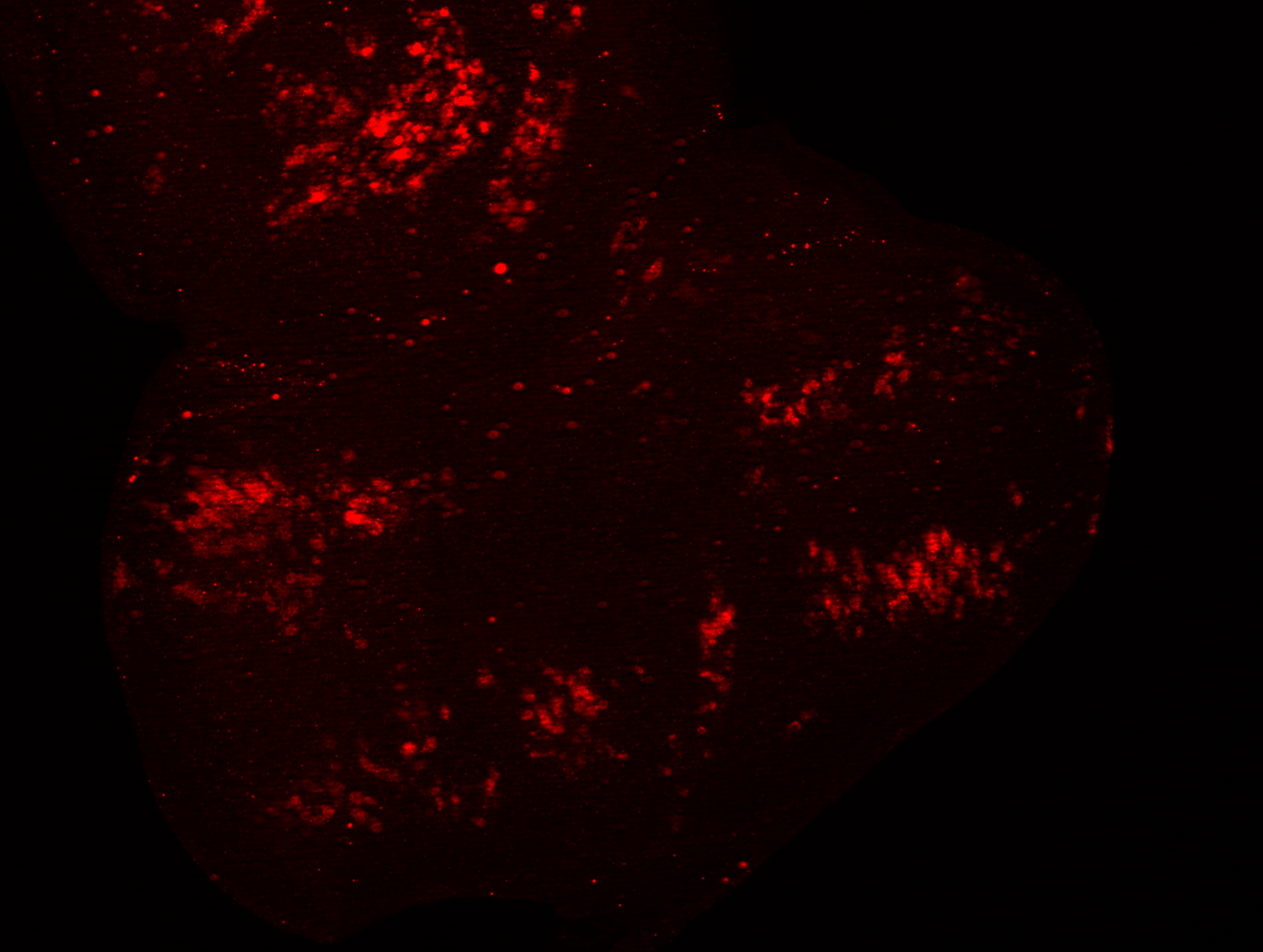

Supplement: Supplementary file 6 — Source data Fig. 2 [file 44318_2025_489_MOESM6_ESM.zip › Figure 2G/4 original image.tif]

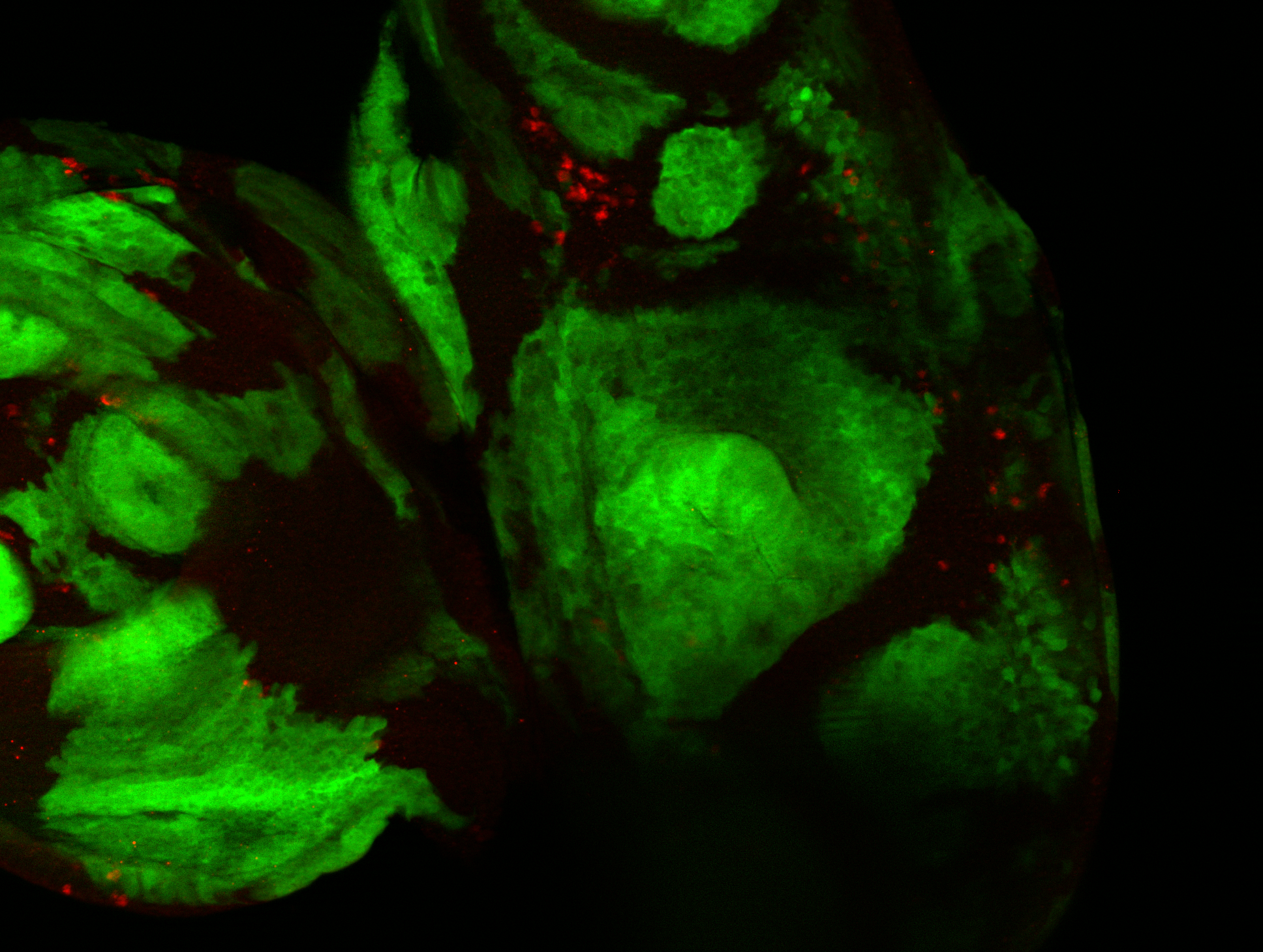

Supplement: Supplementary file 6 — Source data Fig. 2 [file 44318_2025_489_MOESM6_ESM.zip › Figure 2G/5 original image.tif]

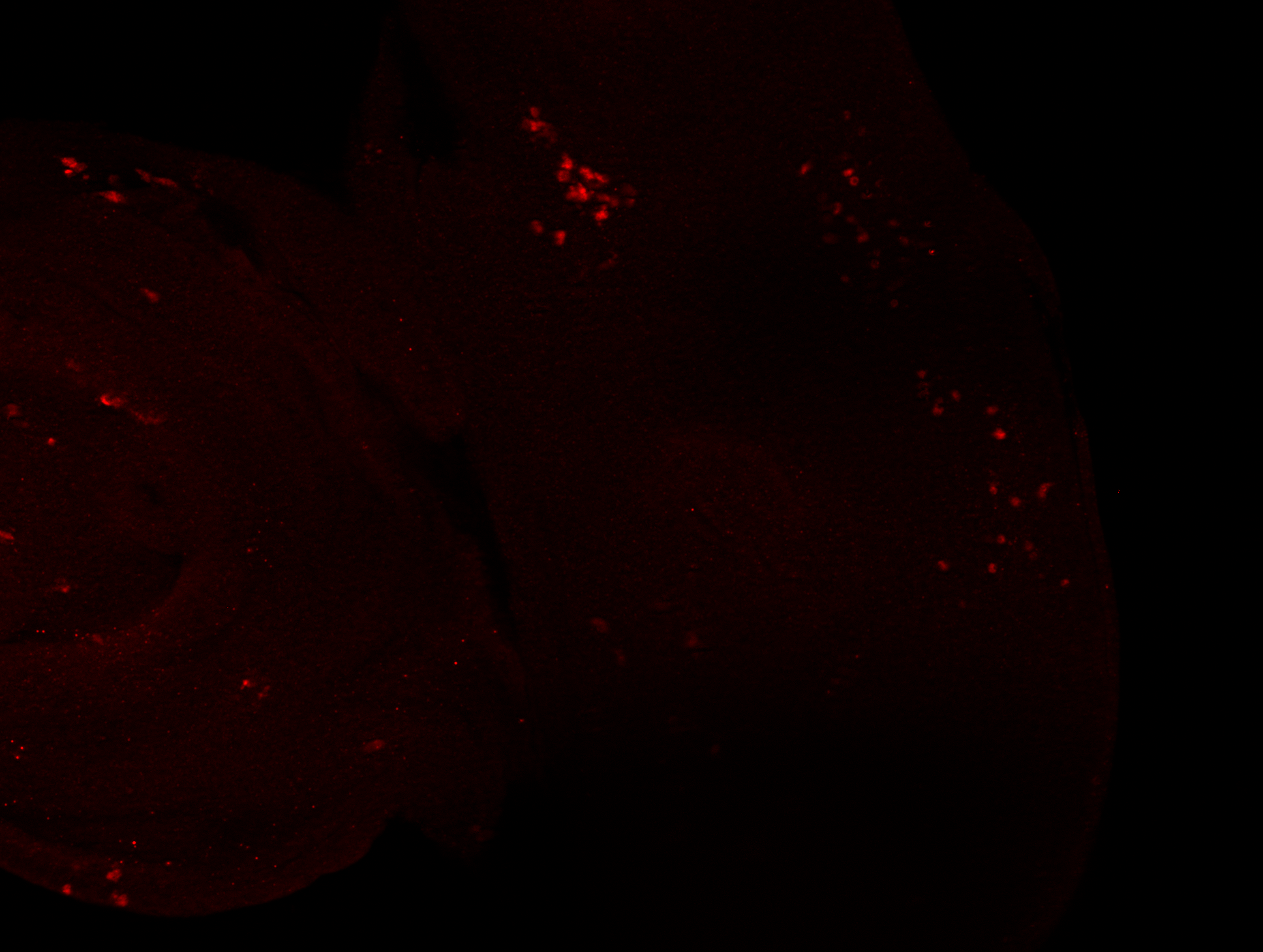

Supplement: Supplementary file 6 — Source data Fig. 2 [file 44318_2025_489_MOESM6_ESM.zip › Figure 2G/6 original image.tif]

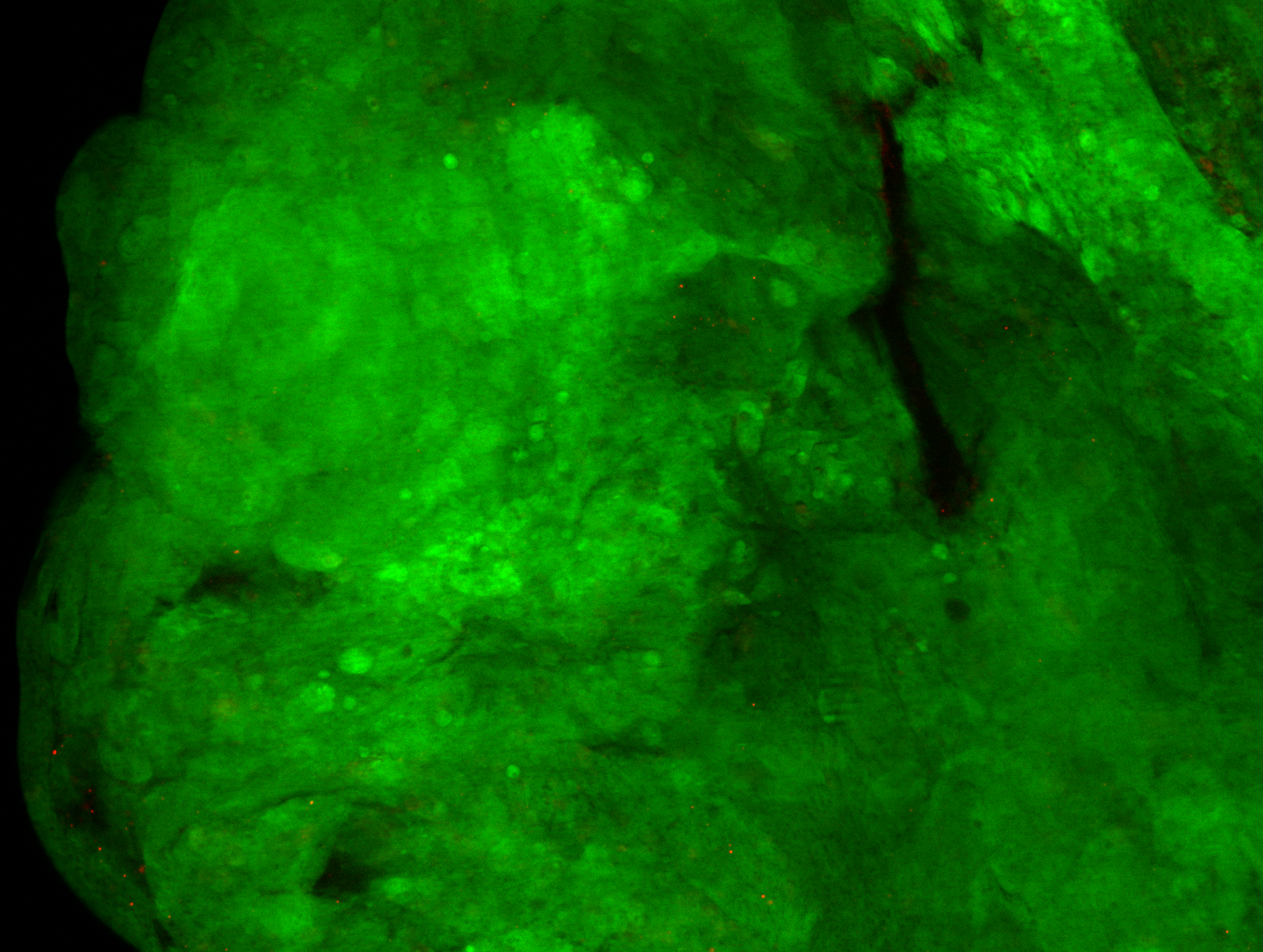

Supplement: Supplementary file 6 — Source data Fig. 2 [file 44318_2025_489_MOESM6_ESM.zip › Figure 2G/7 original image.tif]

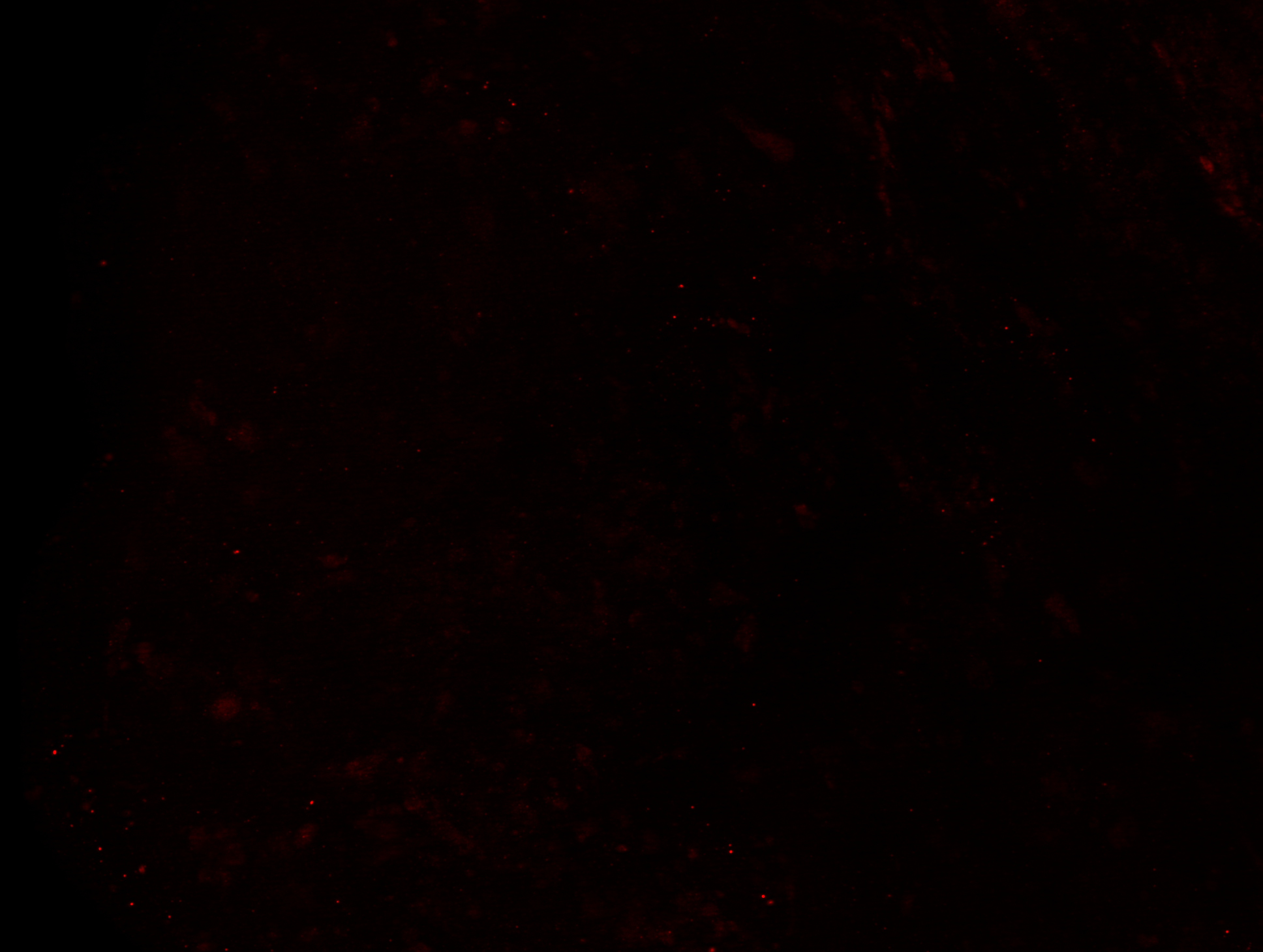

Supplement: Supplementary file 6 — Source data Fig. 2 [file 44318_2025_489_MOESM6_ESM.zip › Figure 2G/8 original image.tif]

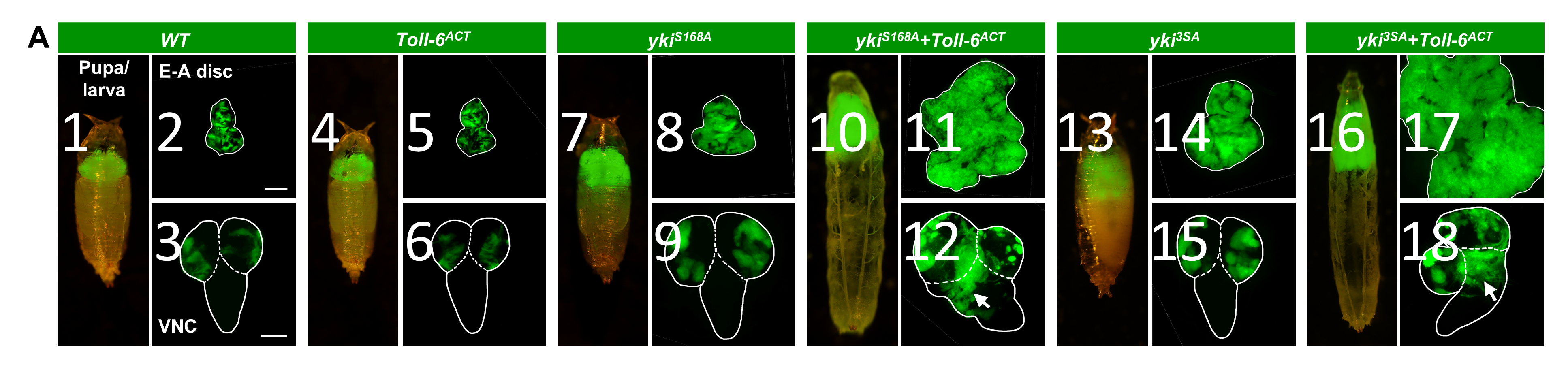

Supplement: Supplementary file 6 — Source data Fig. 2 [file 44318_2025_489_MOESM6_ESM.zip › Figure 2A/0 paper Figure 2A with provided image sequence.tif]

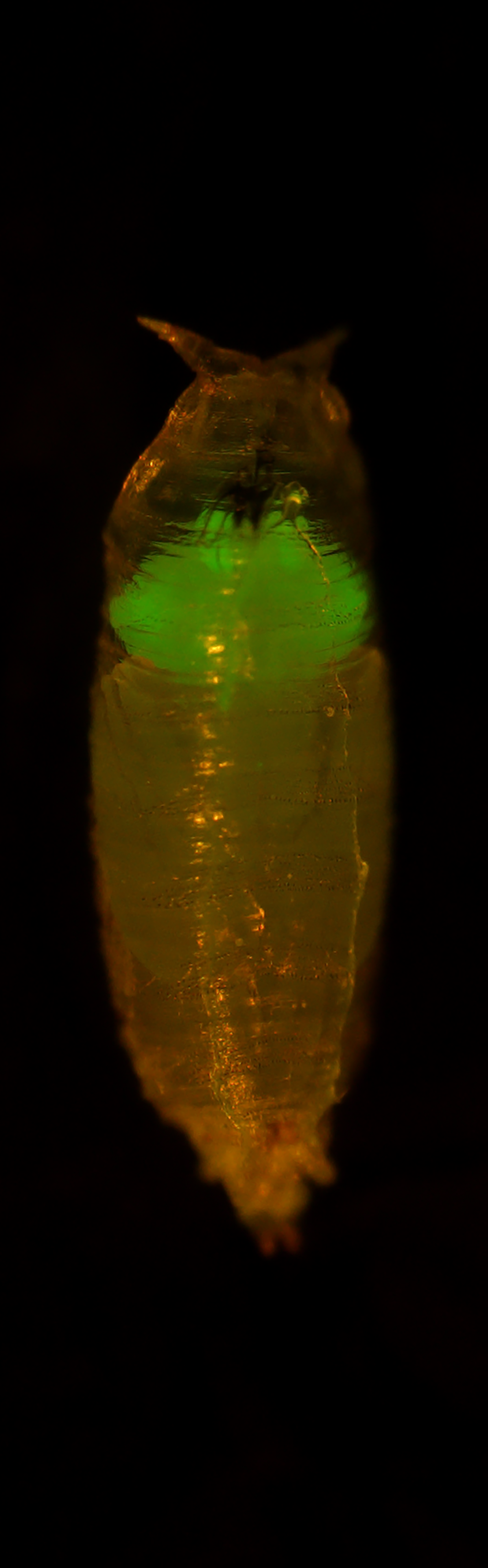

Supplement: Supplementary file 6 — Source data Fig. 2 [file 44318_2025_489_MOESM6_ESM.zip › Figure 2A/1-1 rotated and cut image.tif]

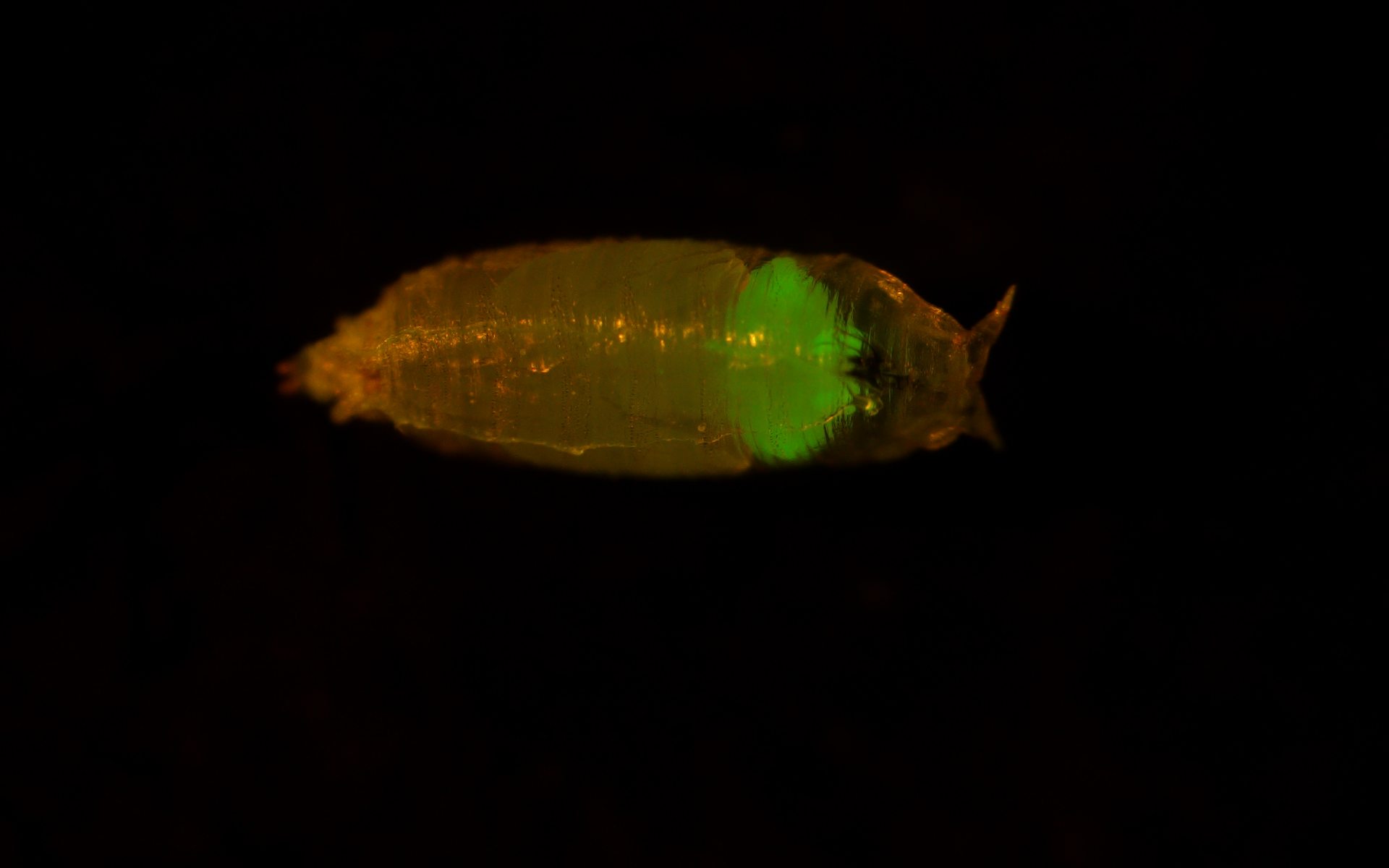

Supplement: Supplementary file 6 — Source data Fig. 2 [file 44318_2025_489_MOESM6_ESM.zip › Figure 2A/1-2 original image.tif]

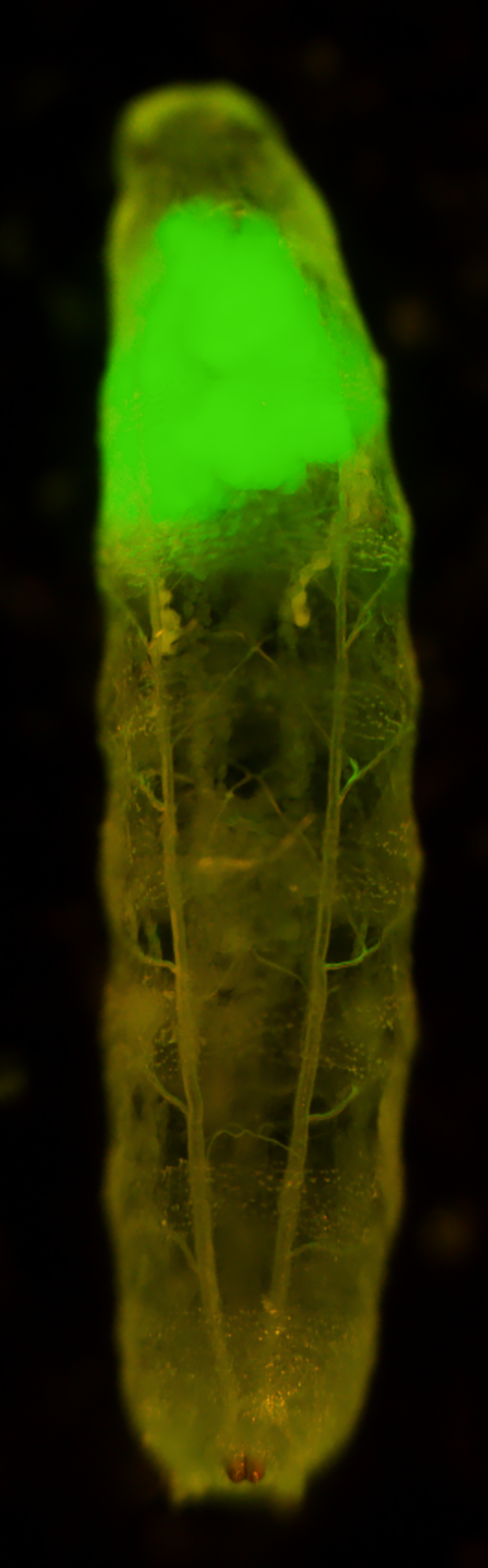

Supplement: Supplementary file 6 — Source data Fig. 2 [file 44318_2025_489_MOESM6_ESM.zip › Figure 2A/10-1 rotated and cut image.tif]

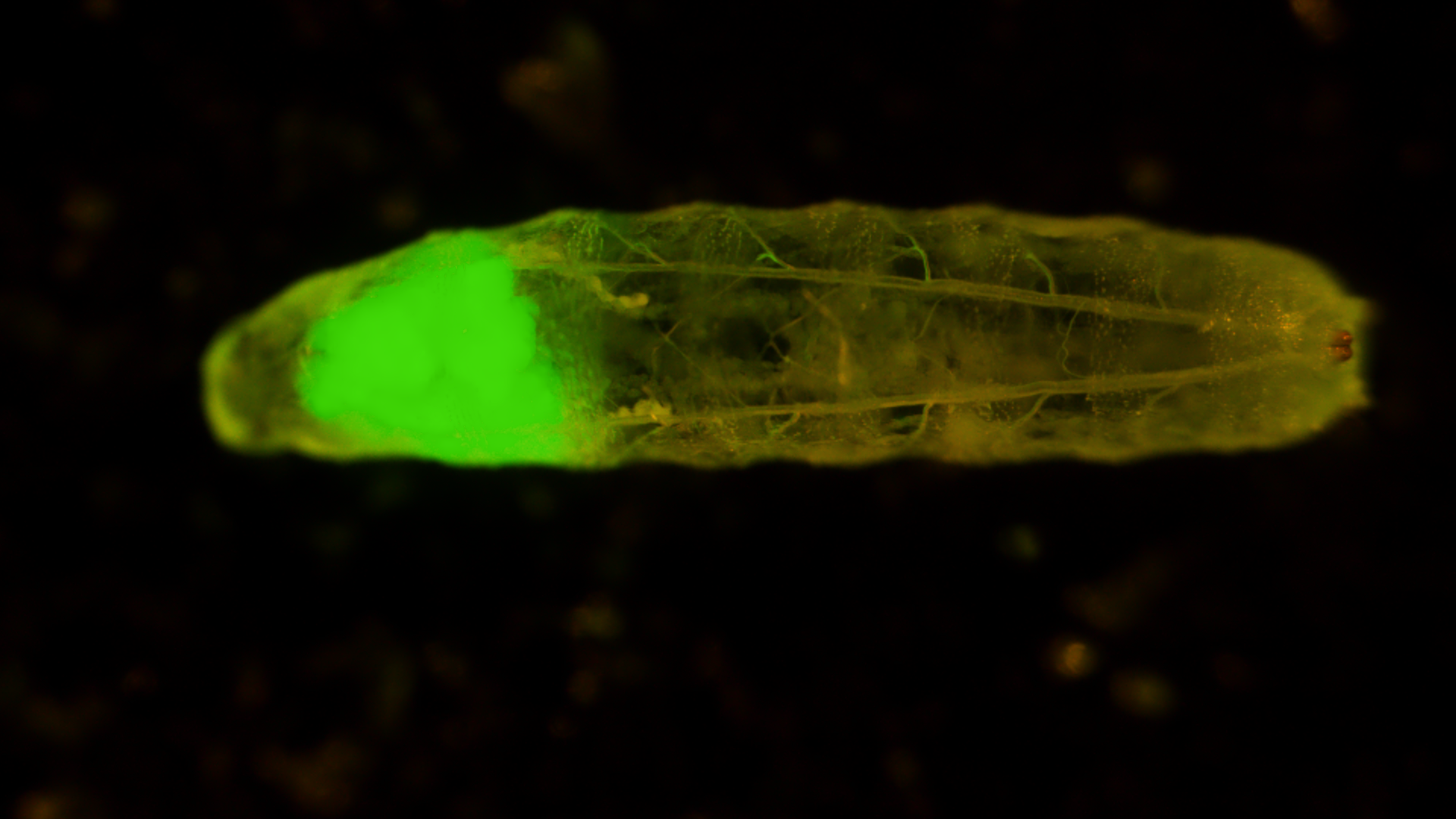

Supplement: Supplementary file 6 — Source data Fig. 2 [file 44318_2025_489_MOESM6_ESM.zip › Figure 2A/10-2 original image.tif]

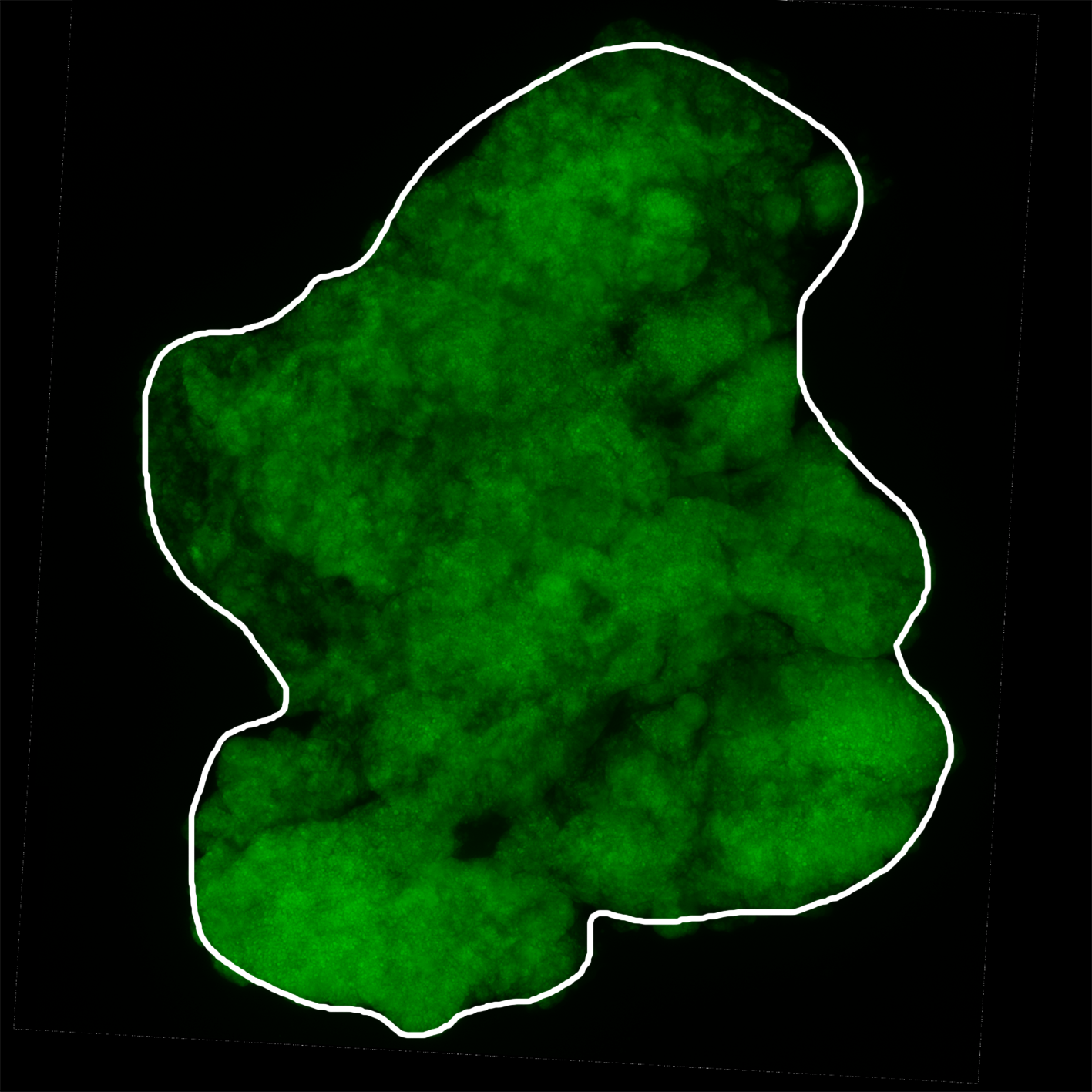

Supplement: Supplementary file 6 — Source data Fig. 2 [file 44318_2025_489_MOESM6_ESM.zip › Figure 2A/11-1 rotated and cut image with border line.tif]

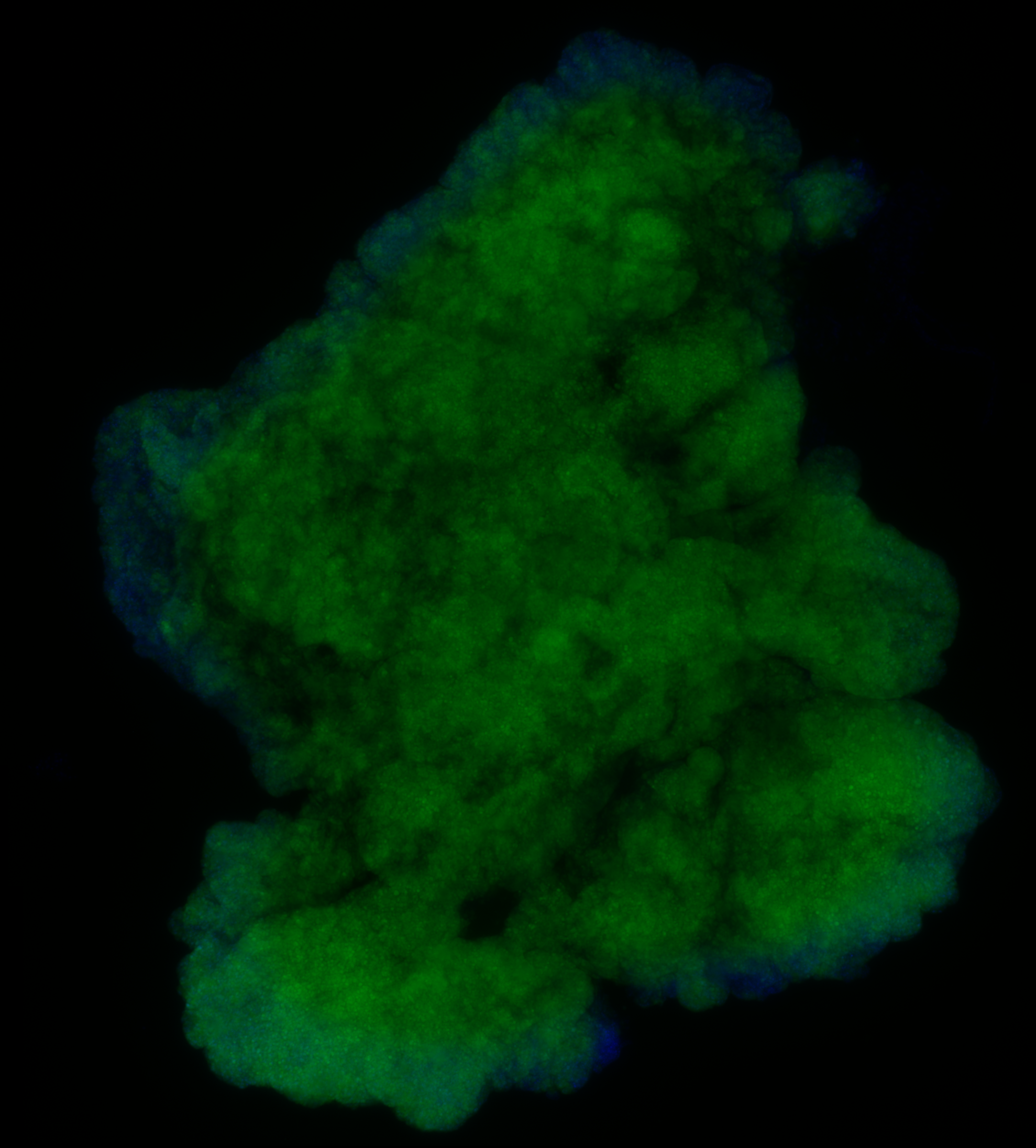

Supplement: Supplementary file 6 — Source data Fig. 2 [file 44318_2025_489_MOESM6_ESM.zip › Figure 2A/11-2 original image.tif]

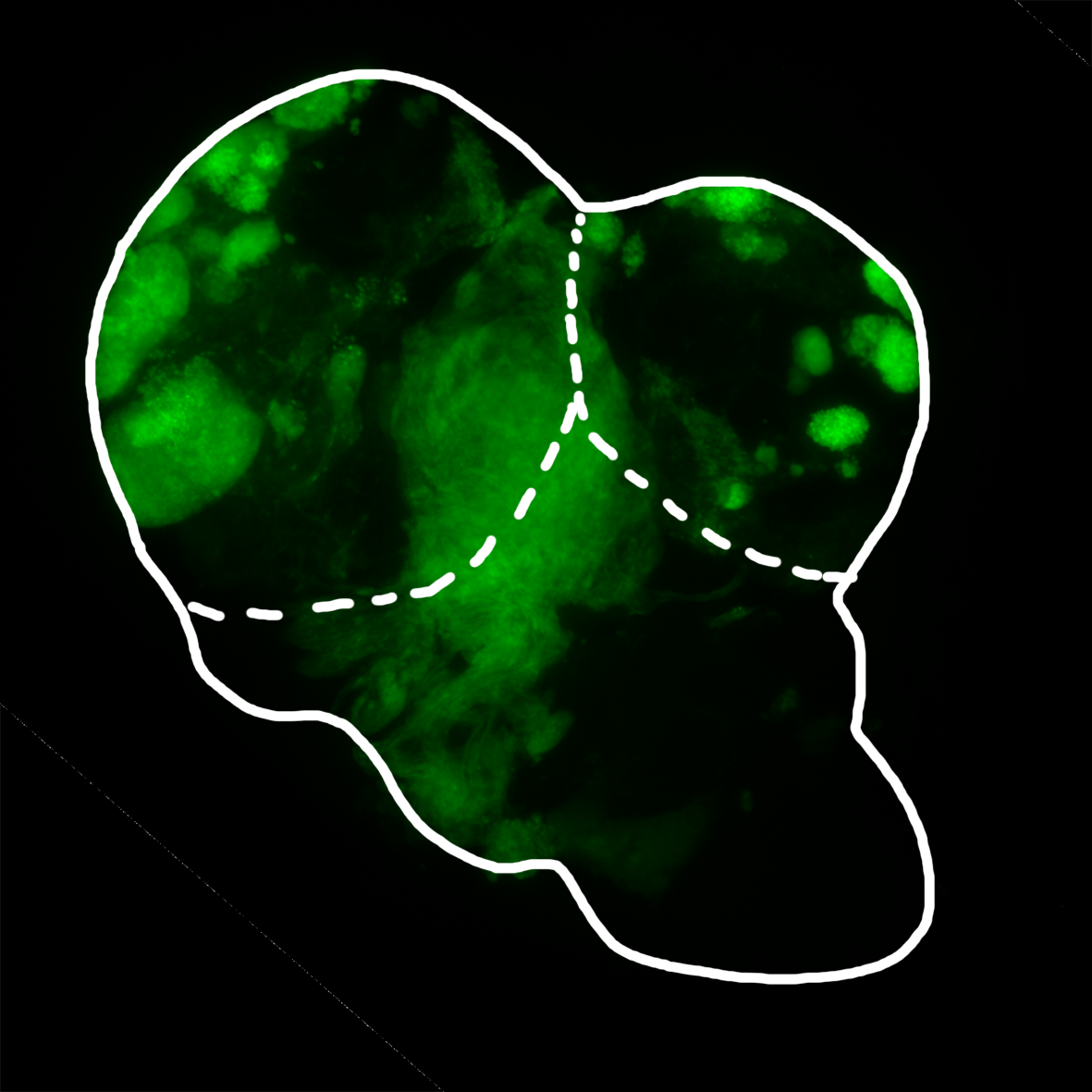

Supplement: Supplementary file 6 — Source data Fig. 2 [file 44318_2025_489_MOESM6_ESM.zip › Figure 2A/12-1 rotated and cut image with border line.tif]

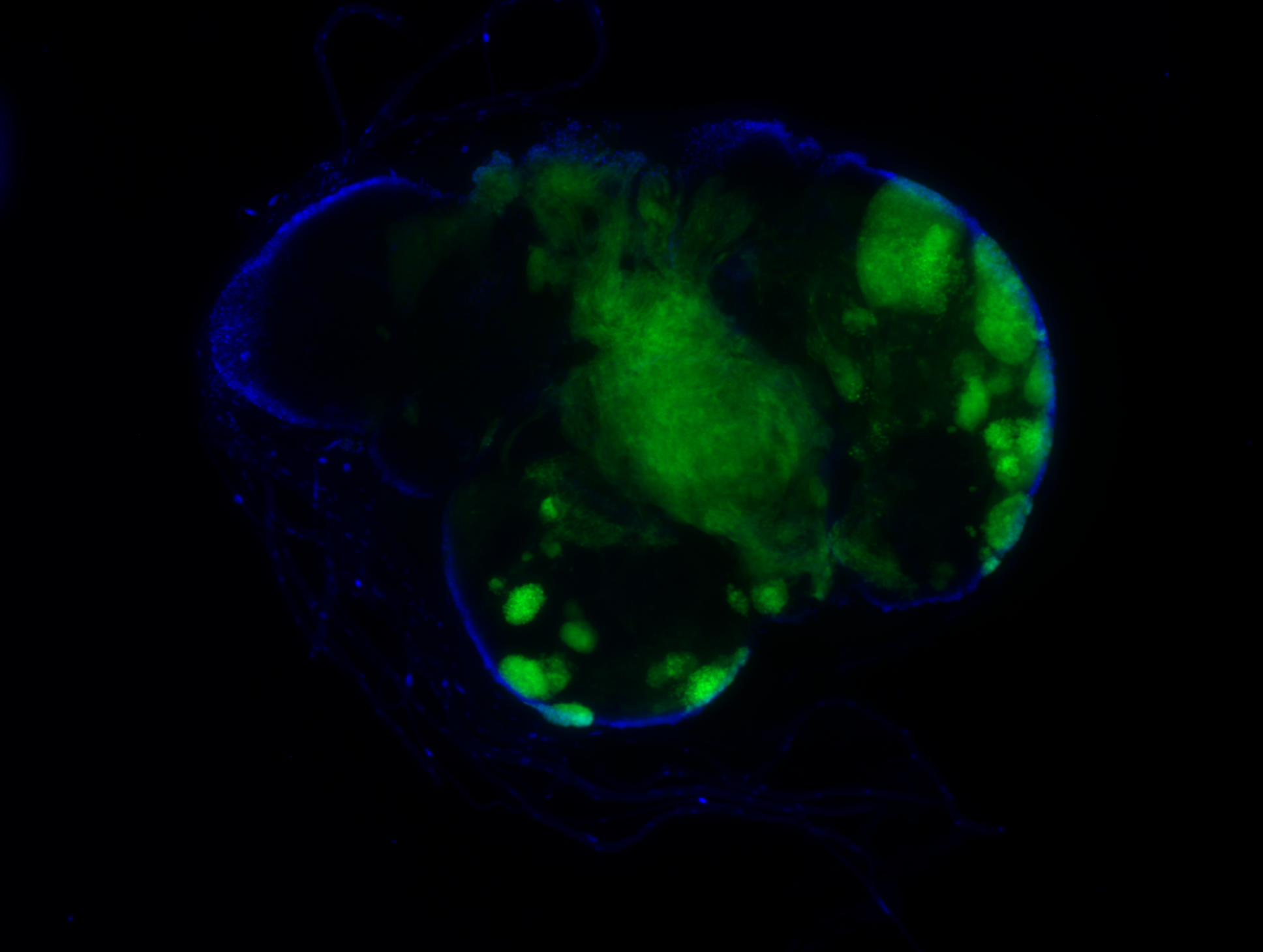

Supplement: Supplementary file 6 — Source data Fig. 2 [file 44318_2025_489_MOESM6_ESM.zip › Figure 2A/12-2 original image.tif]

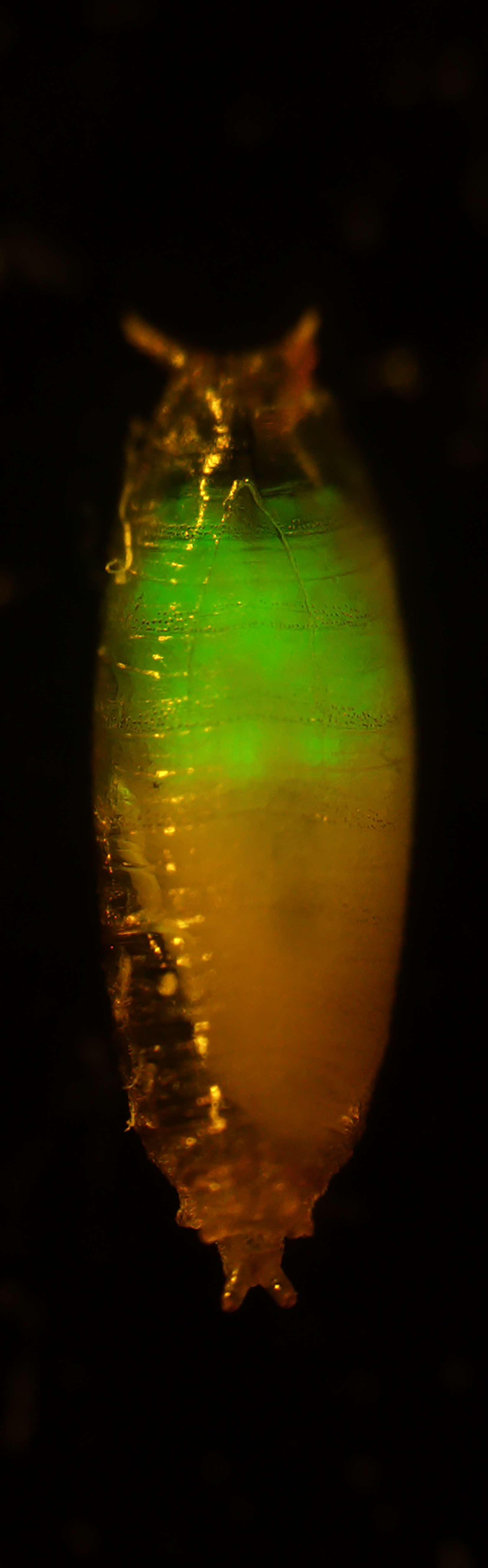

Supplement: Supplementary file 6 — Source data Fig. 2 [file 44318_2025_489_MOESM6_ESM.zip › Figure 2A/13-1 rotated and cut image.tif]

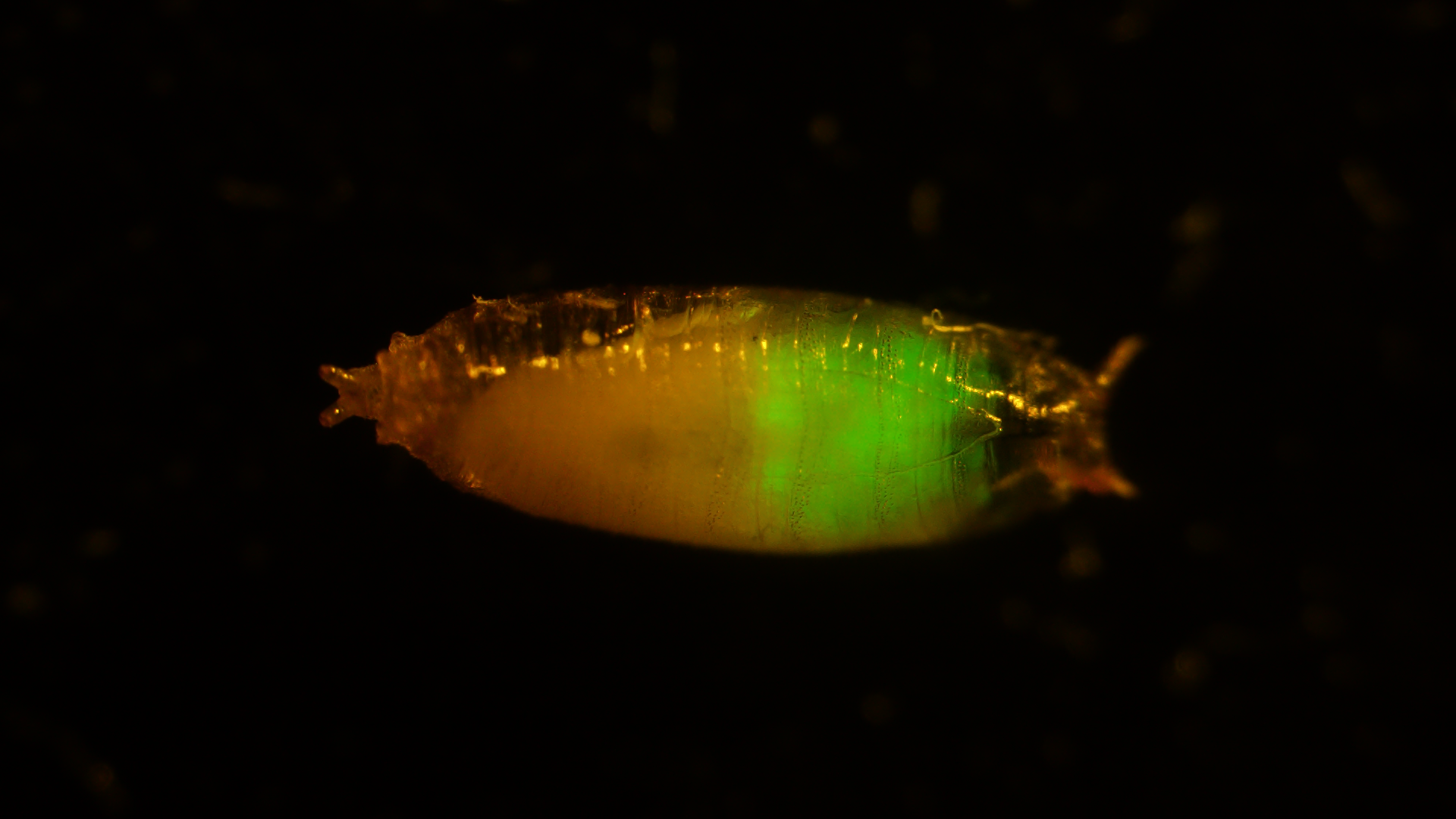

Supplement: Supplementary file 6 — Source data Fig. 2 [file 44318_2025_489_MOESM6_ESM.zip › Figure 2A/13-2 original image.tif]

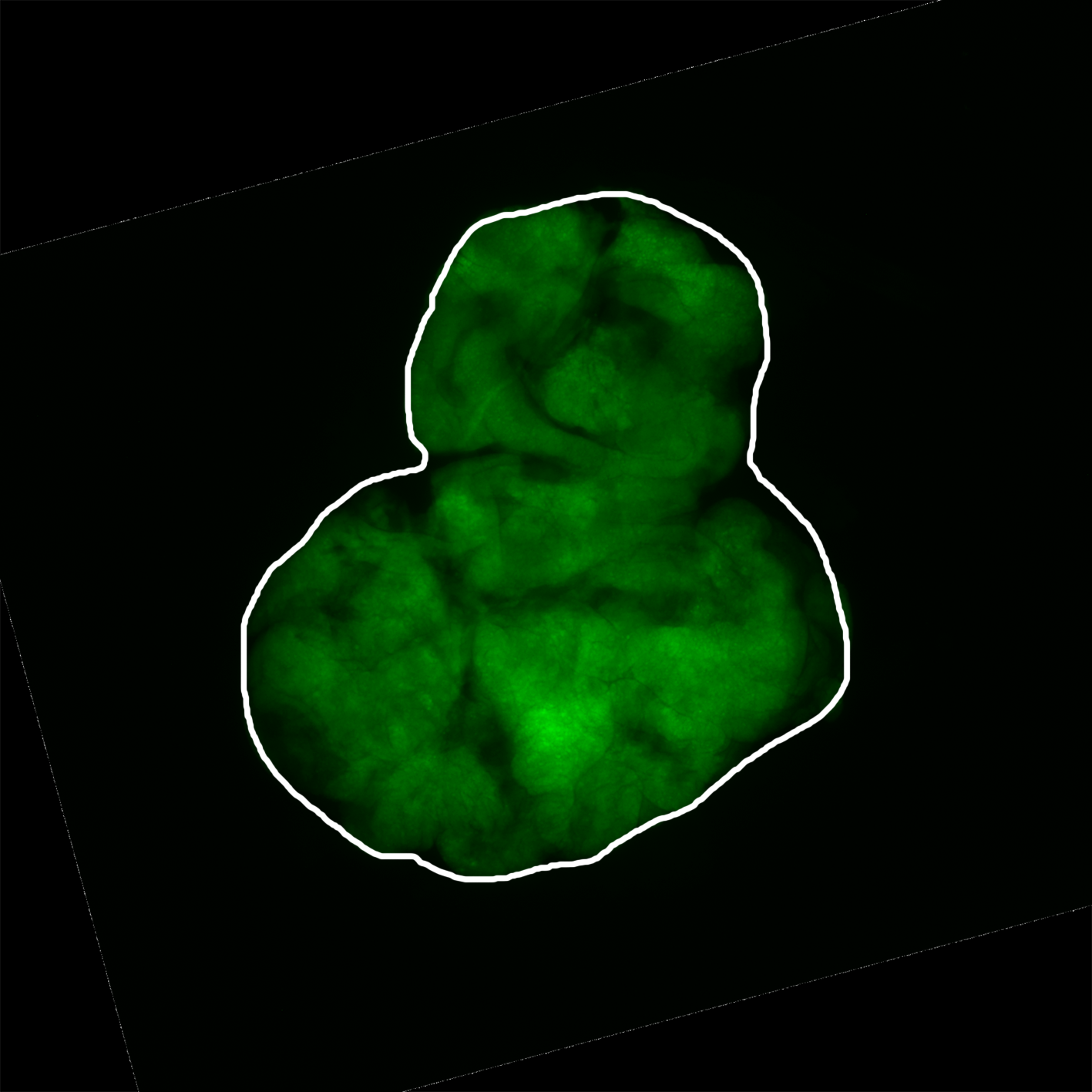

Supplement: Supplementary file 6 — Source data Fig. 2 [file 44318_2025_489_MOESM6_ESM.zip › Figure 2A/14-1 rotated and cut image with border line.tif]

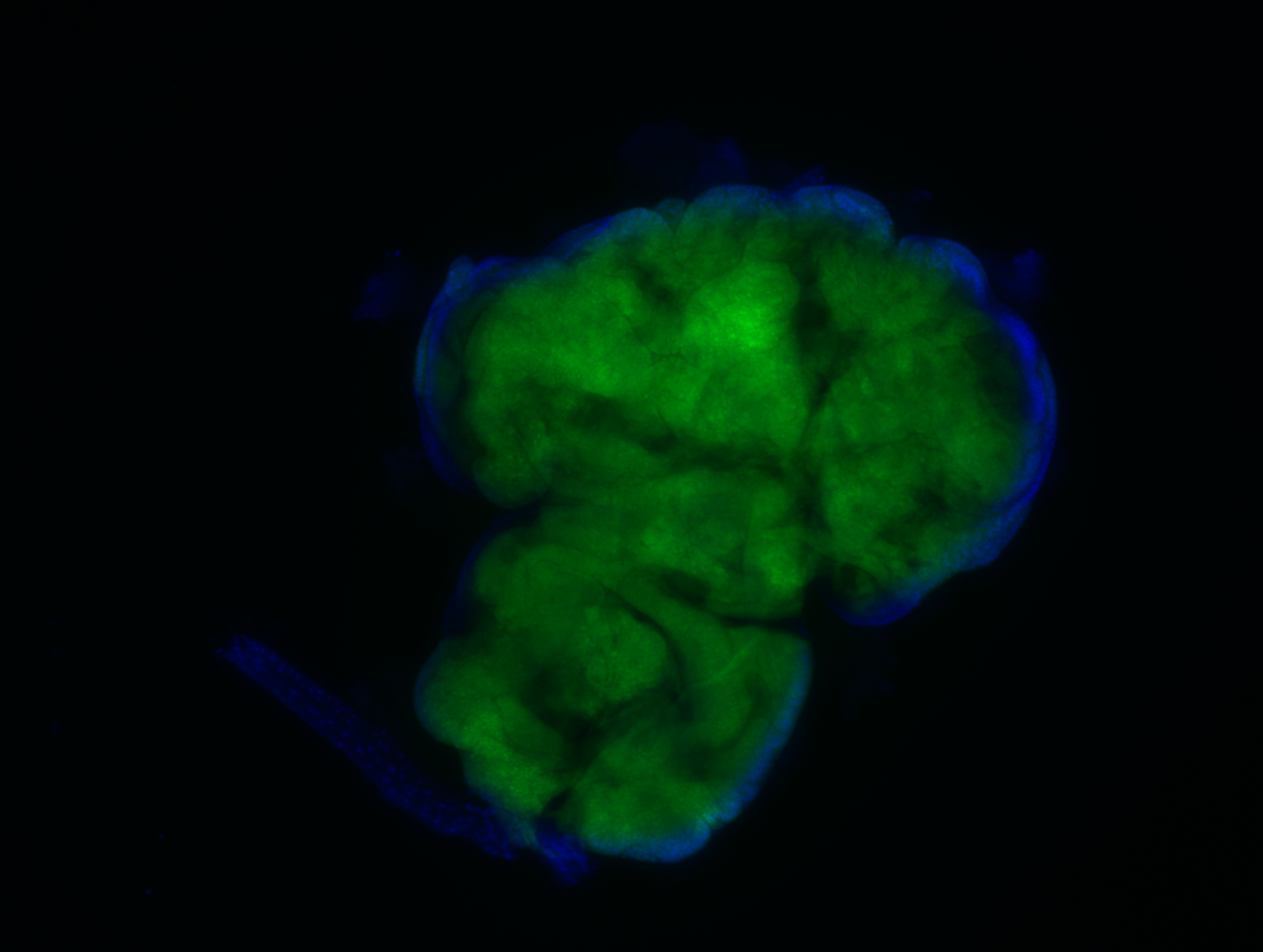

Supplement: Supplementary file 6 — Source data Fig. 2 [file 44318_2025_489_MOESM6_ESM.zip › Figure 2A/14-2 original image.tif]

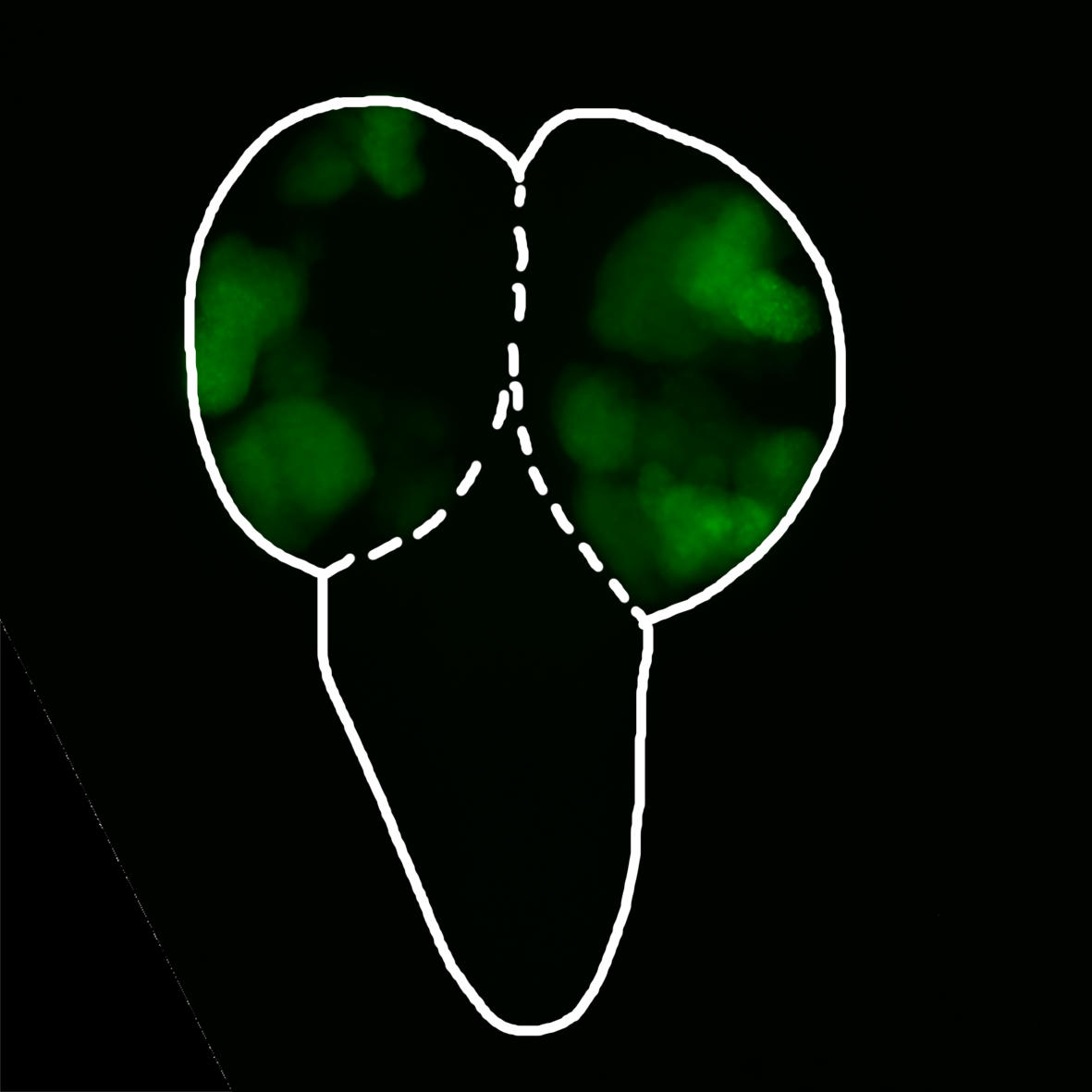

Supplement: Supplementary file 6 — Source data Fig. 2 [file 44318_2025_489_MOESM6_ESM.zip › Figure 2A/15-1 rotated and cut image with border line.tif]

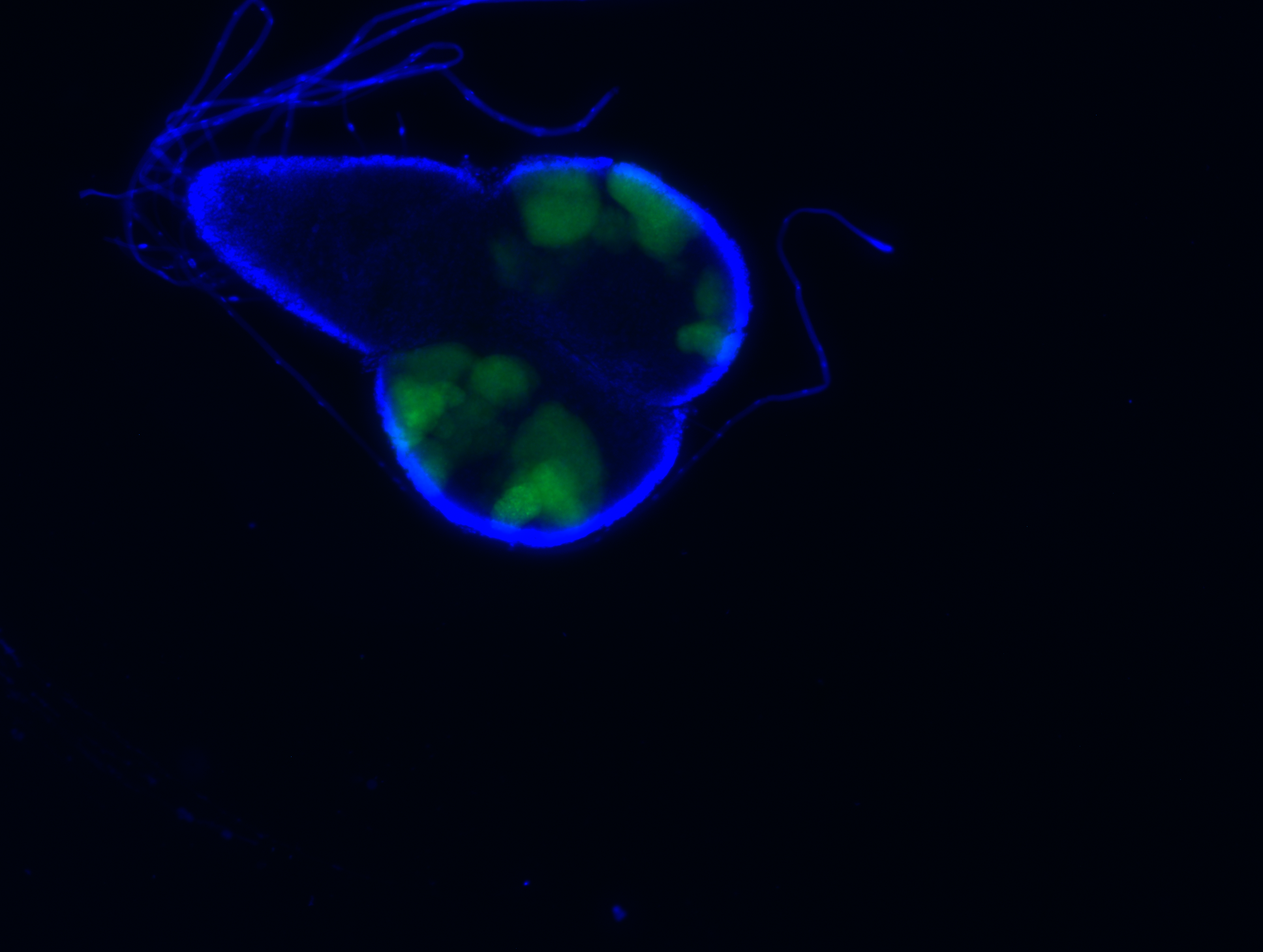

Supplement: Supplementary file 6 — Source data Fig. 2 [file 44318_2025_489_MOESM6_ESM.zip › Figure 2A/15-2 original image.tif]

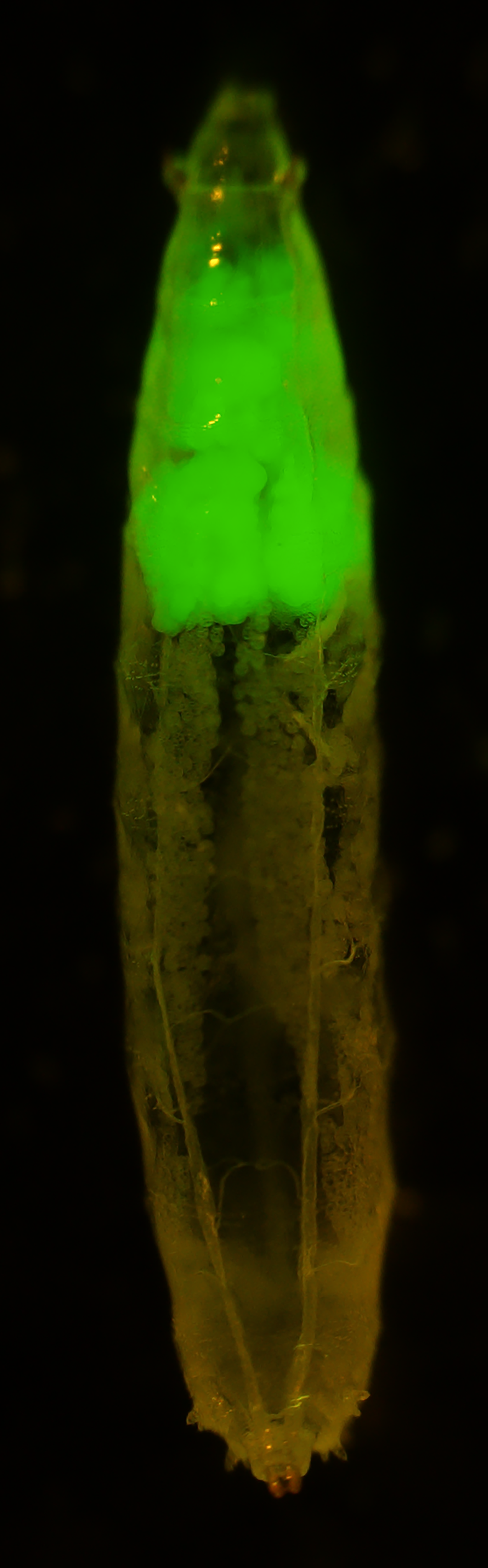

Supplement: Supplementary file 6 — Source data Fig. 2 [file 44318_2025_489_MOESM6_ESM.zip › Figure 2A/16-1 rotated and cut image.tif]

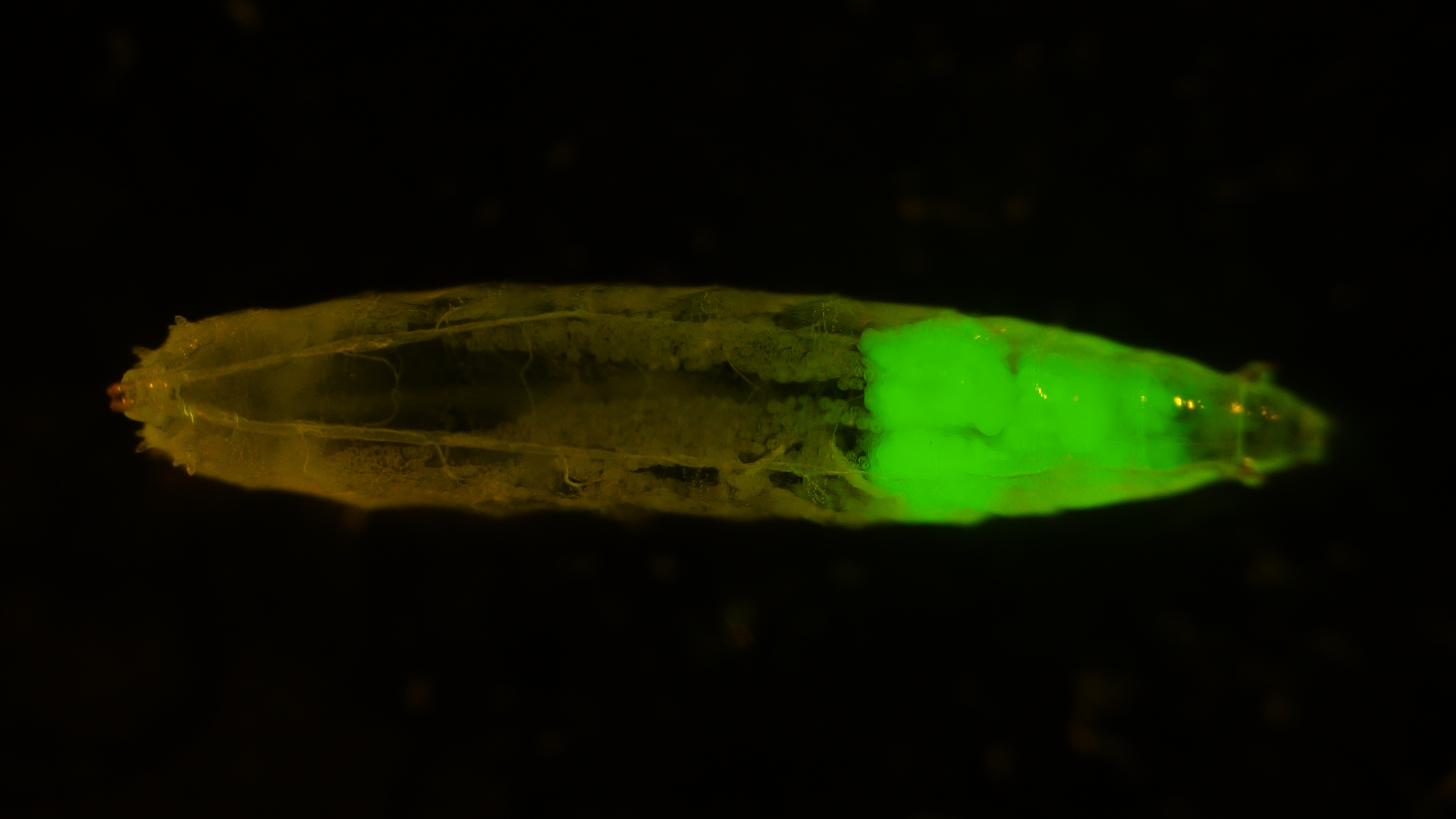

Supplement: Supplementary file 6 — Source data Fig. 2 [file 44318_2025_489_MOESM6_ESM.zip › Figure 2A/16-2 original image.tif]

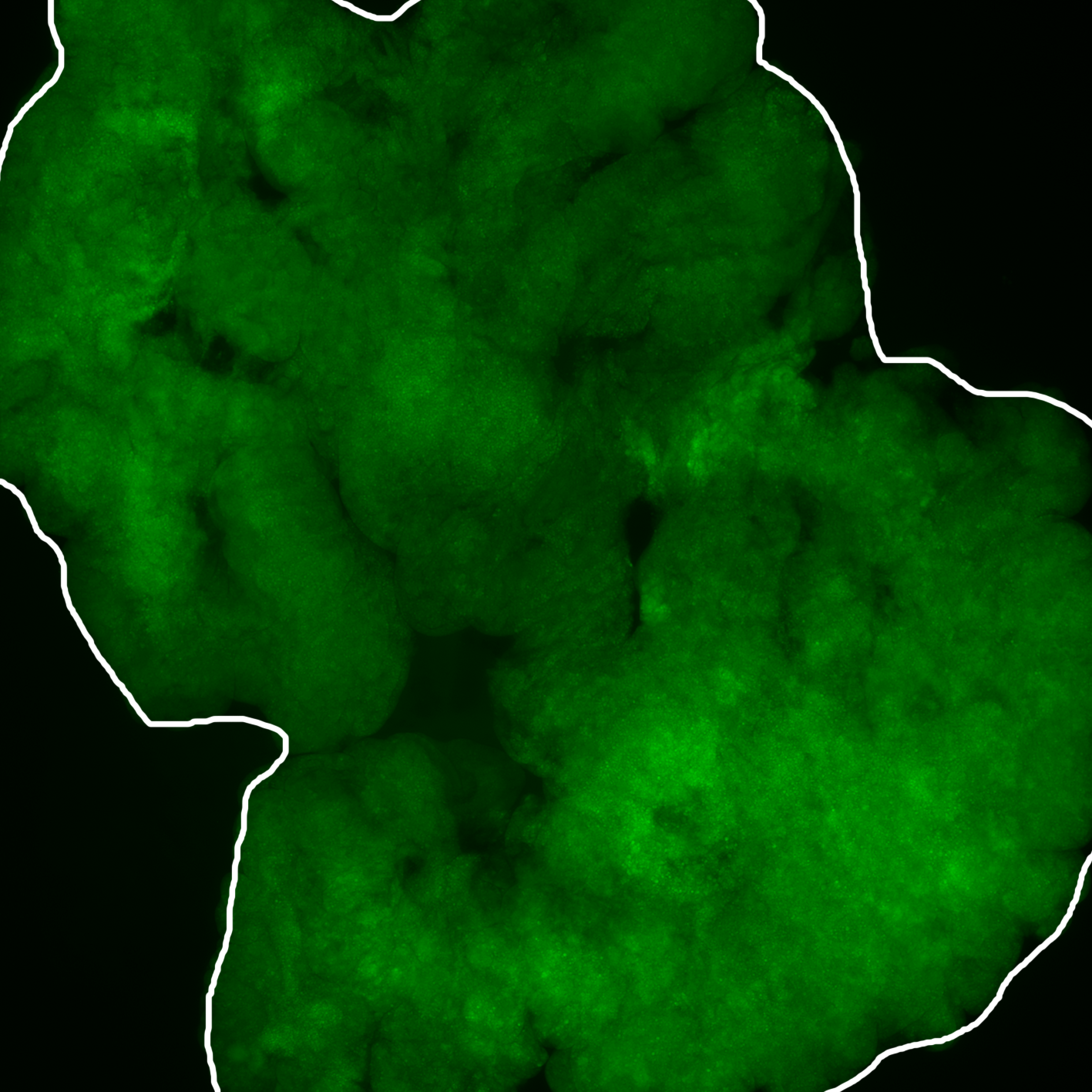

Supplement: Supplementary file 6 — Source data Fig. 2 [file 44318_2025_489_MOESM6_ESM.zip › Figure 2A/17-1 rotated and cut image with border line.tif]

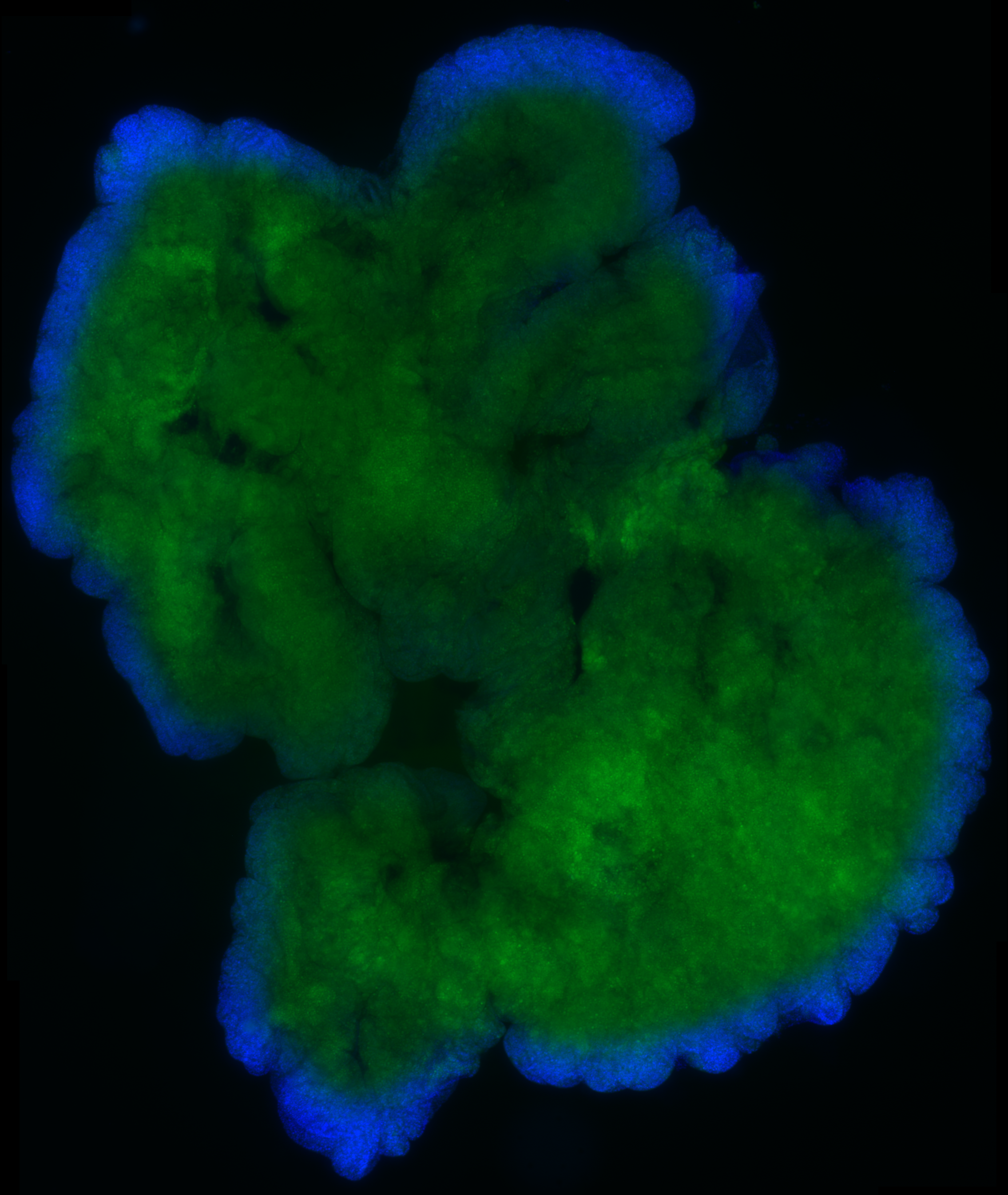

Supplement: Supplementary file 6 — Source data Fig. 2 [file 44318_2025_489_MOESM6_ESM.zip › Figure 2A/17-2 original image.tif]

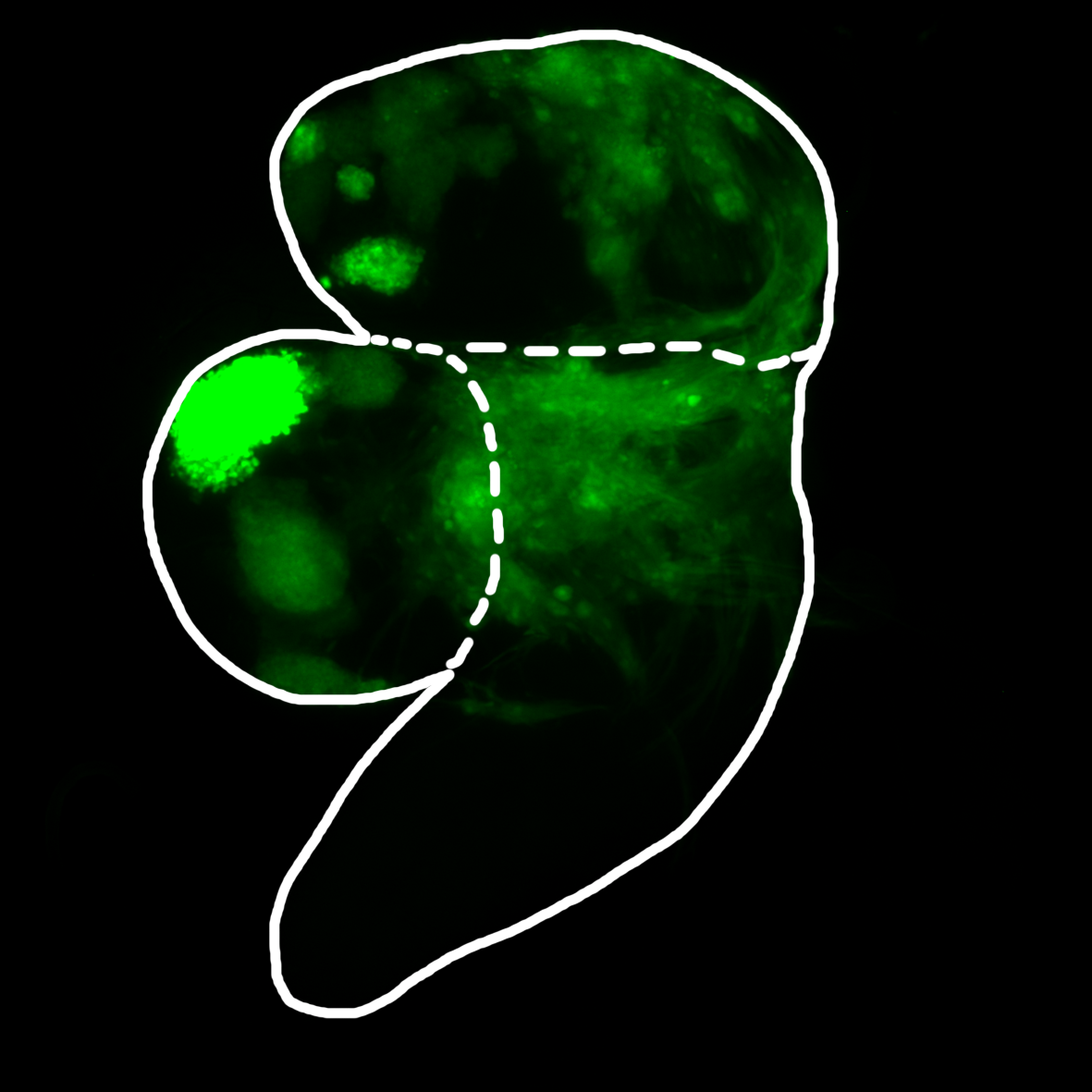

Supplement: Supplementary file 6 — Source data Fig. 2 [file 44318_2025_489_MOESM6_ESM.zip › Figure 2A/18-1 rotated and cut image with border line.tif]

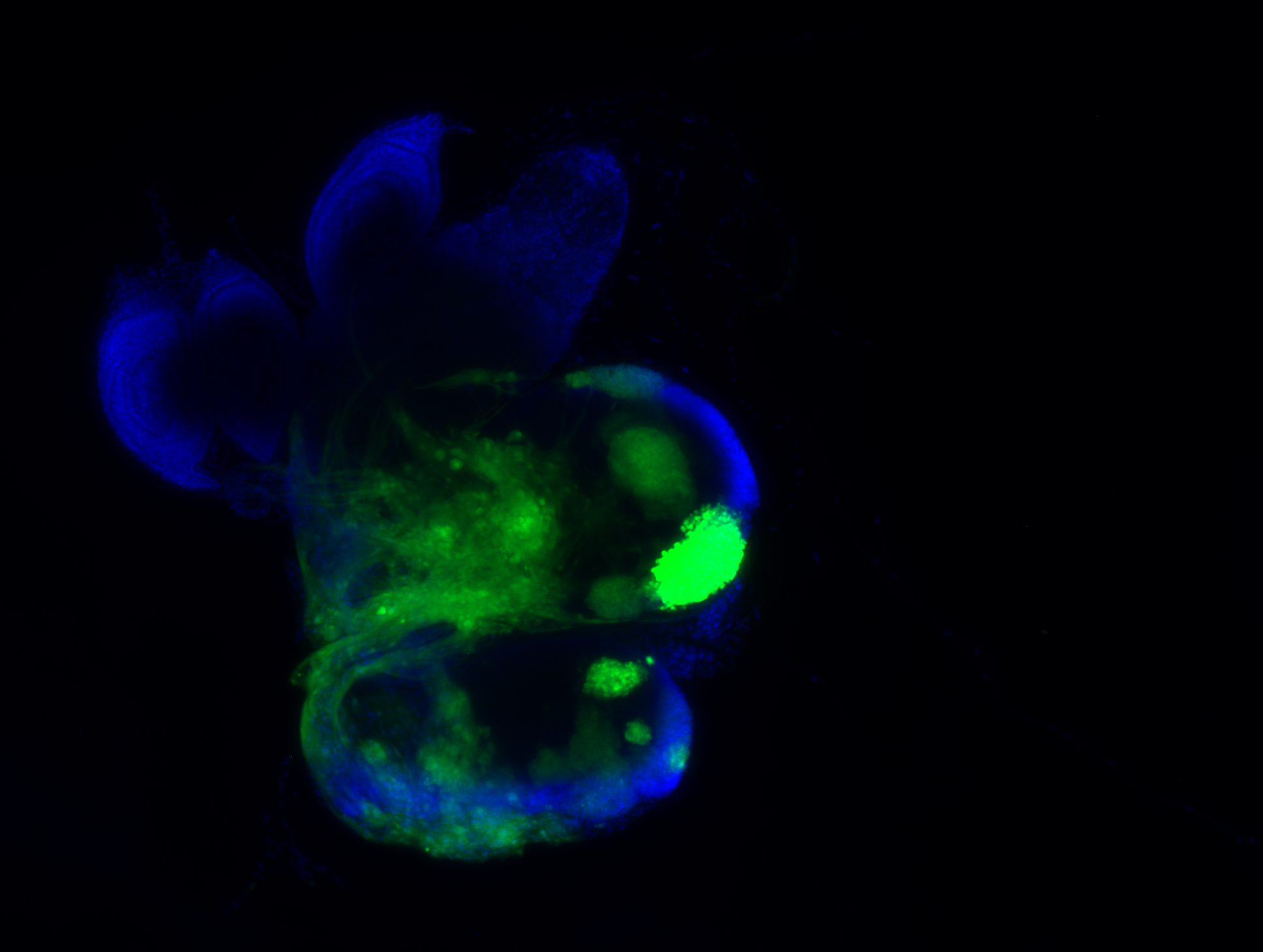

Supplement: Supplementary file 6 — Source data Fig. 2 [file 44318_2025_489_MOESM6_ESM.zip › Figure 2A/18-2 original image.tif]

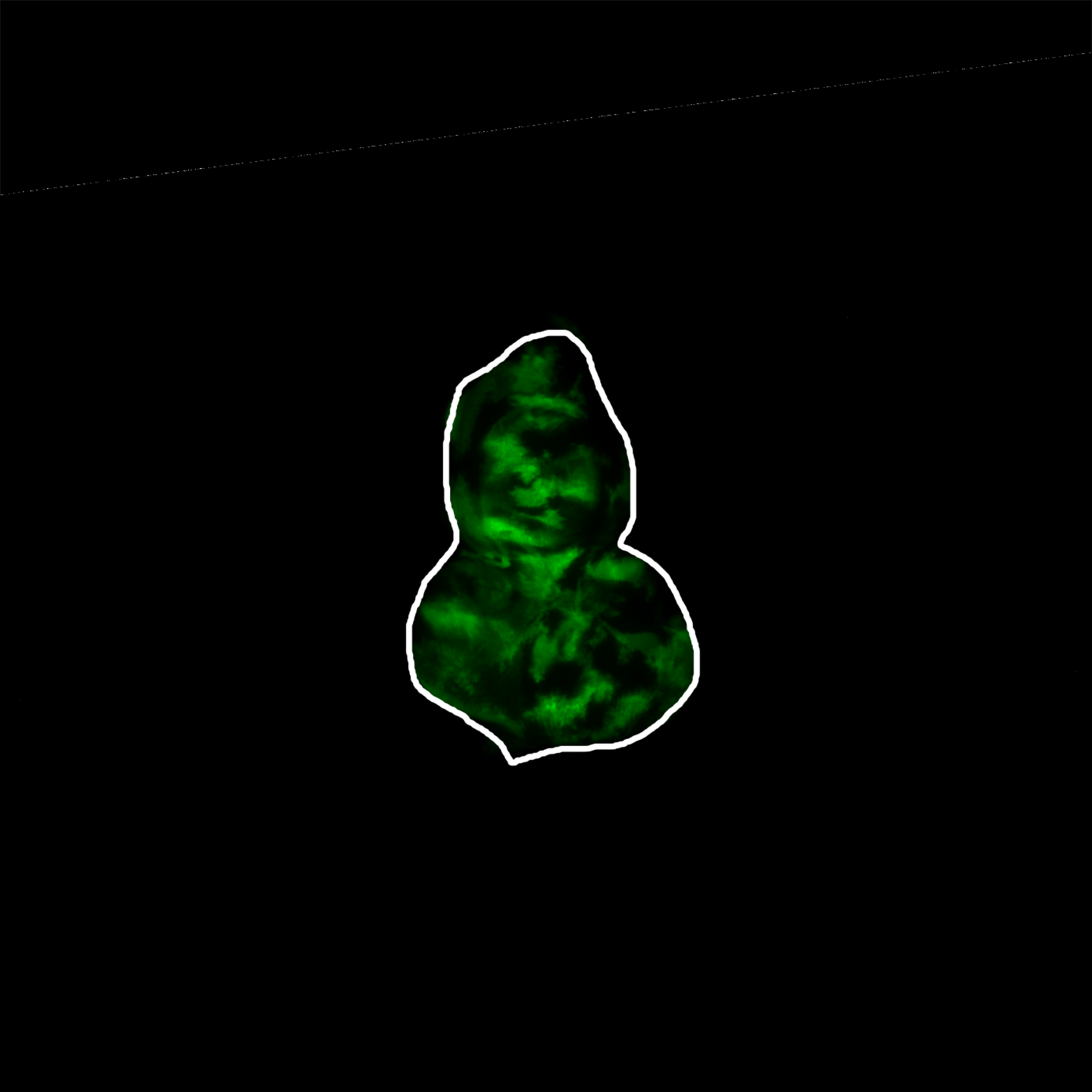

Supplement: Supplementary file 6 — Source data Fig. 2 [file 44318_2025_489_MOESM6_ESM.zip › Figure 2A/2-1 rotated and cut image with border line.tif]

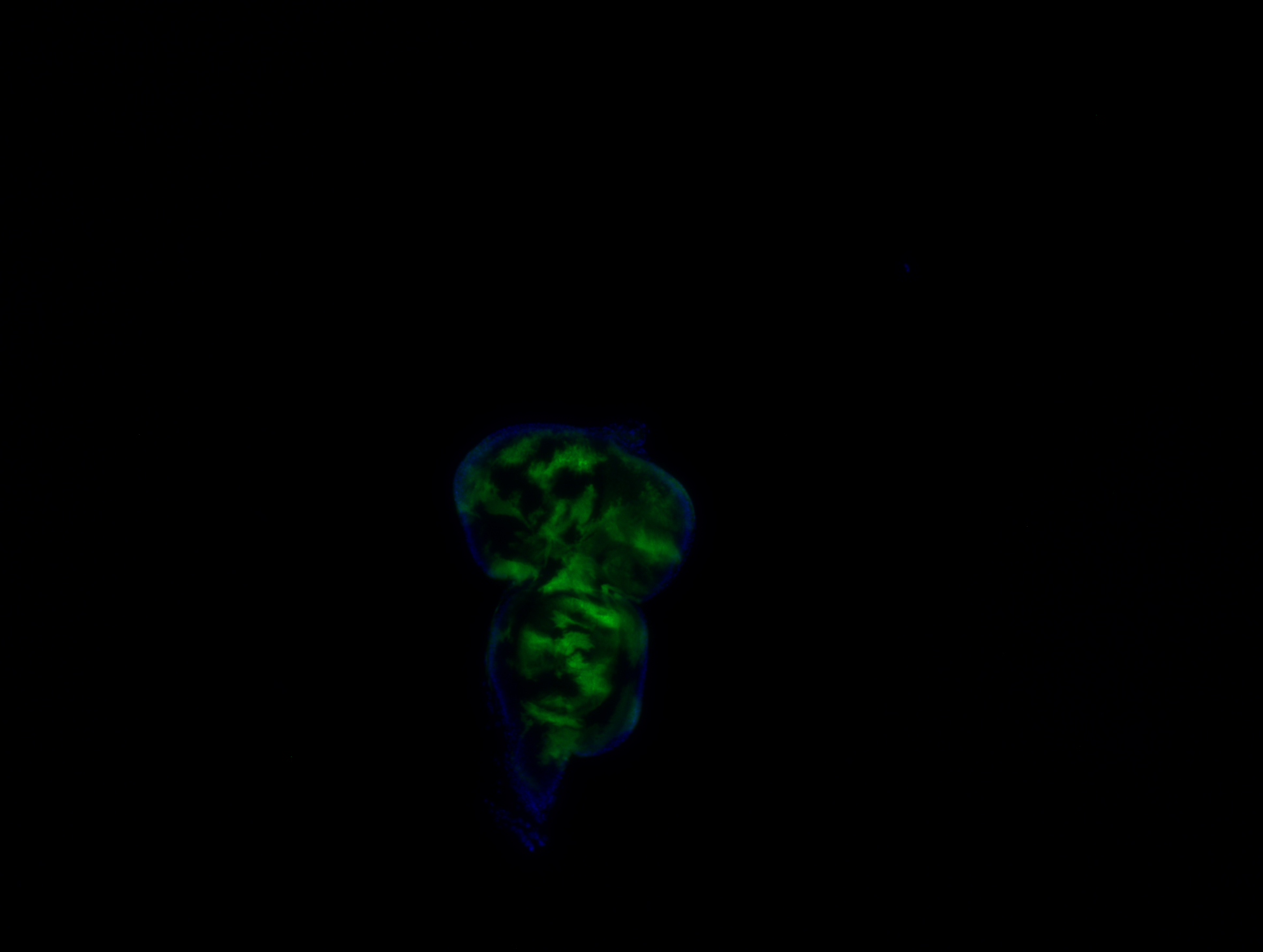

Supplement: Supplementary file 6 — Source data Fig. 2 [file 44318_2025_489_MOESM6_ESM.zip › Figure 2A/2-2 original image.tif]

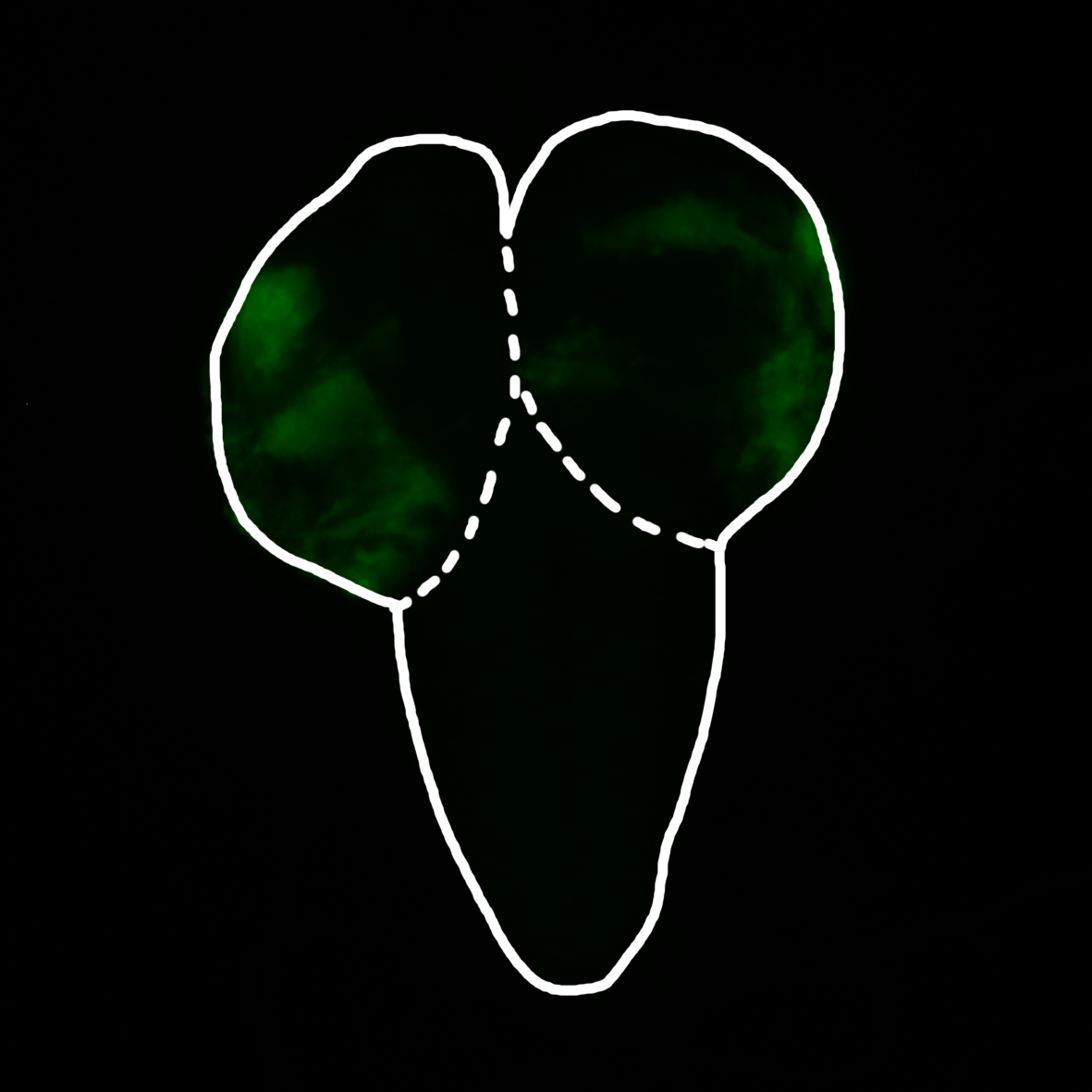

Supplement: Supplementary file 6 — Source data Fig. 2 [file 44318_2025_489_MOESM6_ESM.zip › Figure 2A/3-1 rotated and cut image with border line.tif]

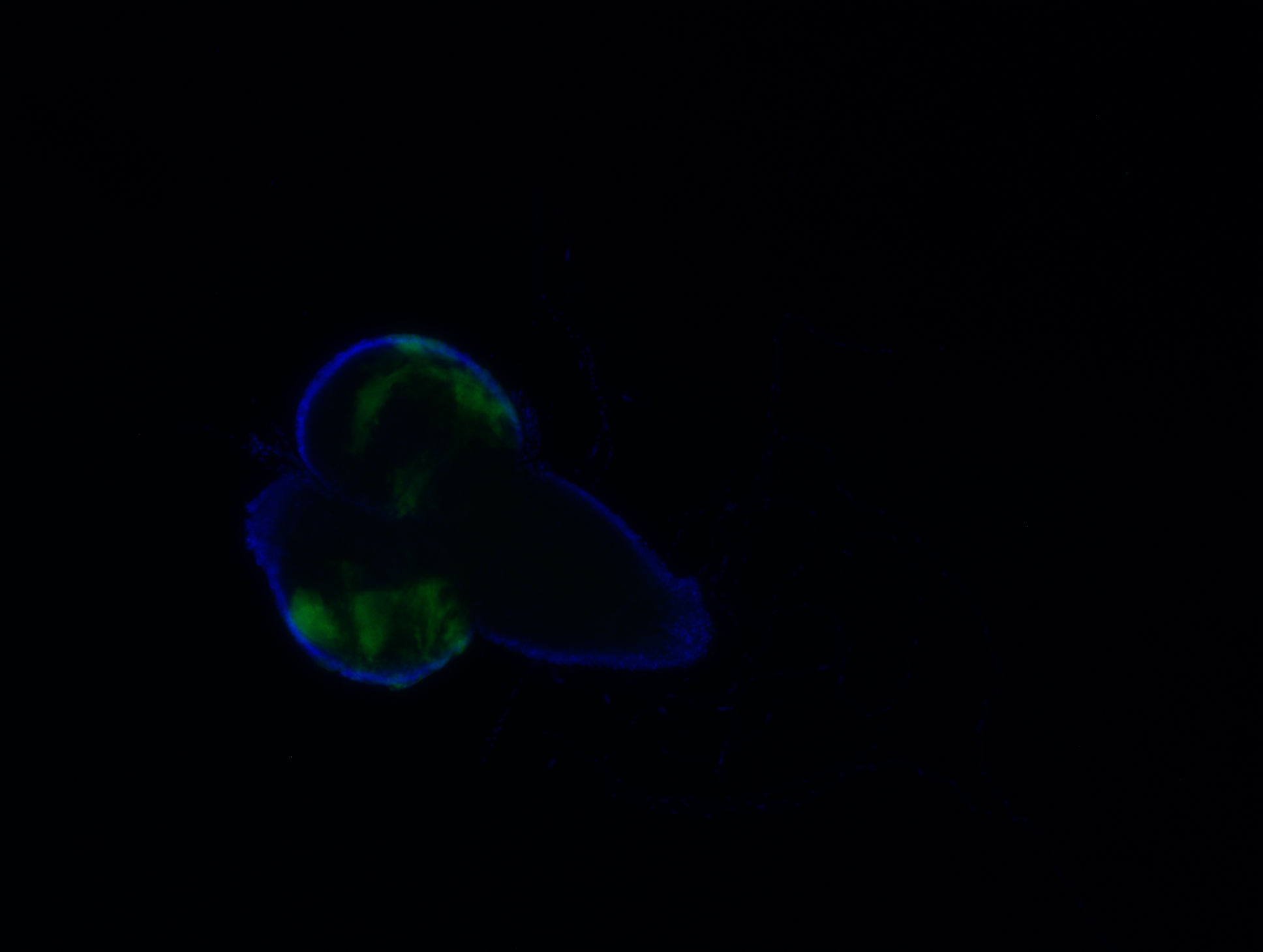

Supplement: Supplementary file 6 — Source data Fig. 2 [file 44318_2025_489_MOESM6_ESM.zip › Figure 2A/3-2 original image.tif]

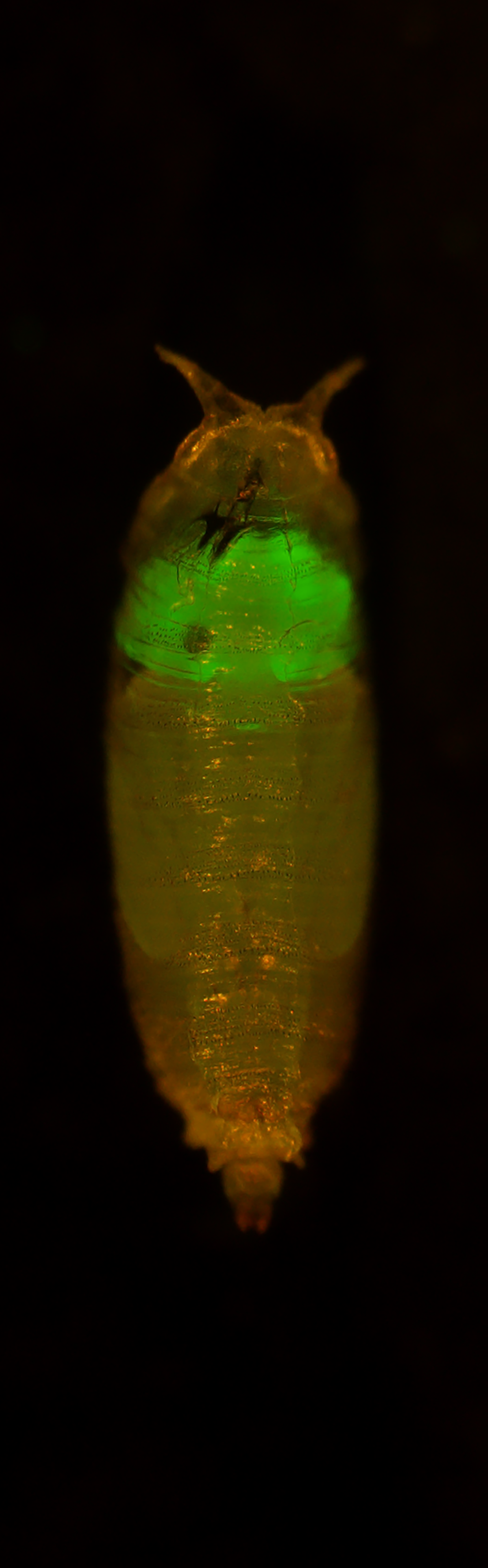

Supplement: Supplementary file 6 — Source data Fig. 2 [file 44318_2025_489_MOESM6_ESM.zip › Figure 2A/4-1 rotated and cut image.tif]

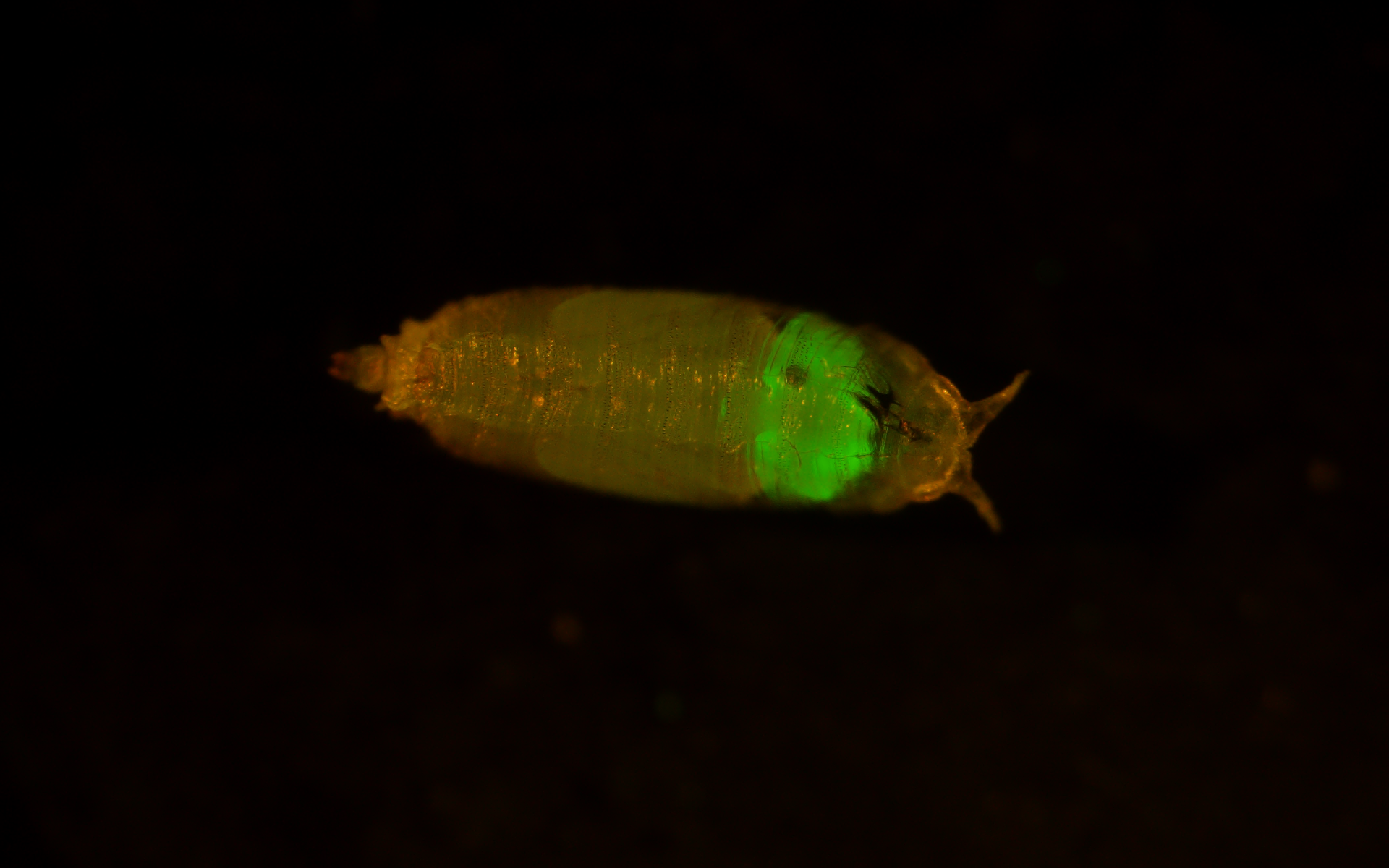

Supplement: Supplementary file 6 — Source data Fig. 2 [file 44318_2025_489_MOESM6_ESM.zip › Figure 2A/4-2 original image.tif]

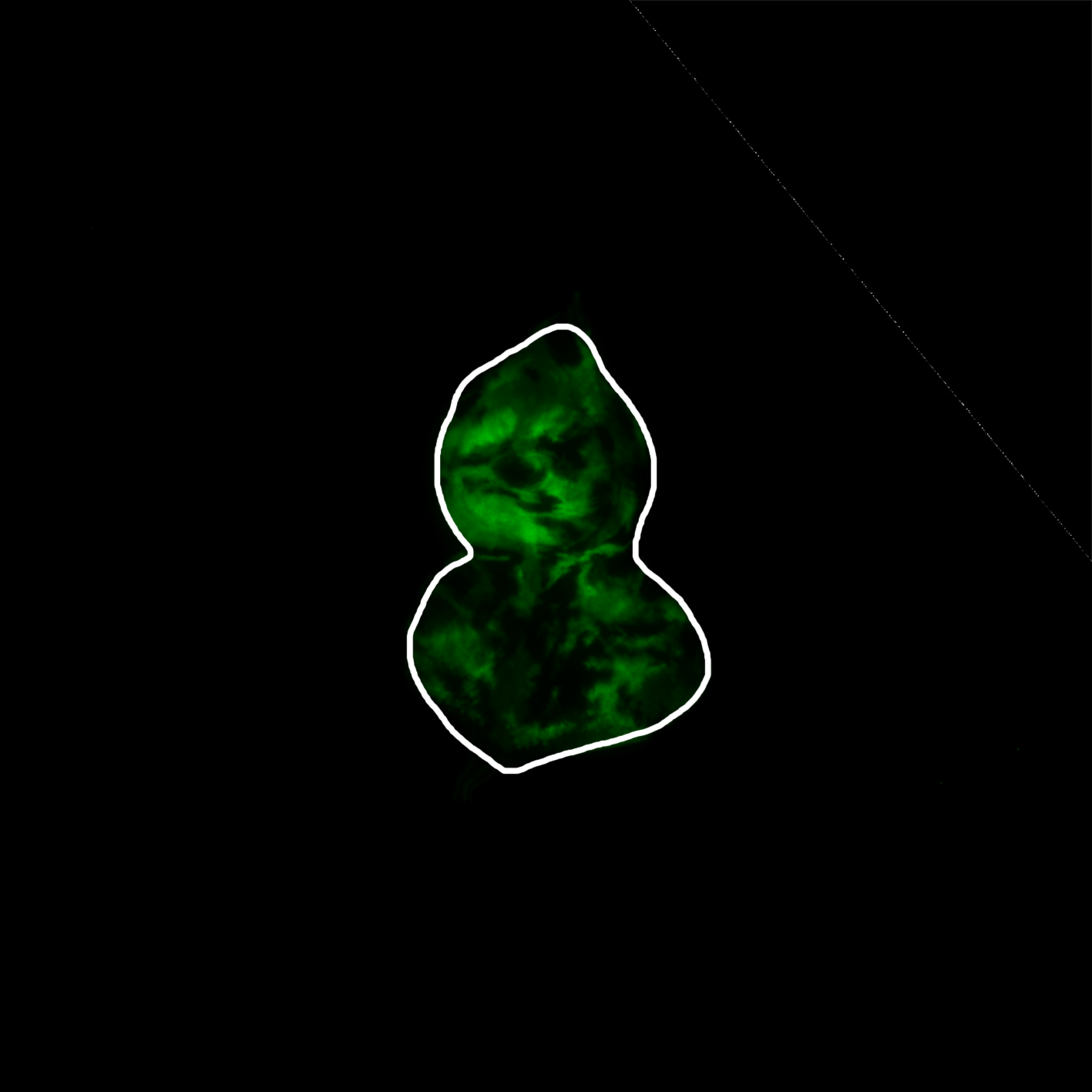

Supplement: Supplementary file 6 — Source data Fig. 2 [file 44318_2025_489_MOESM6_ESM.zip › Figure 2A/5-1 rotated and cut image with border line.tif]

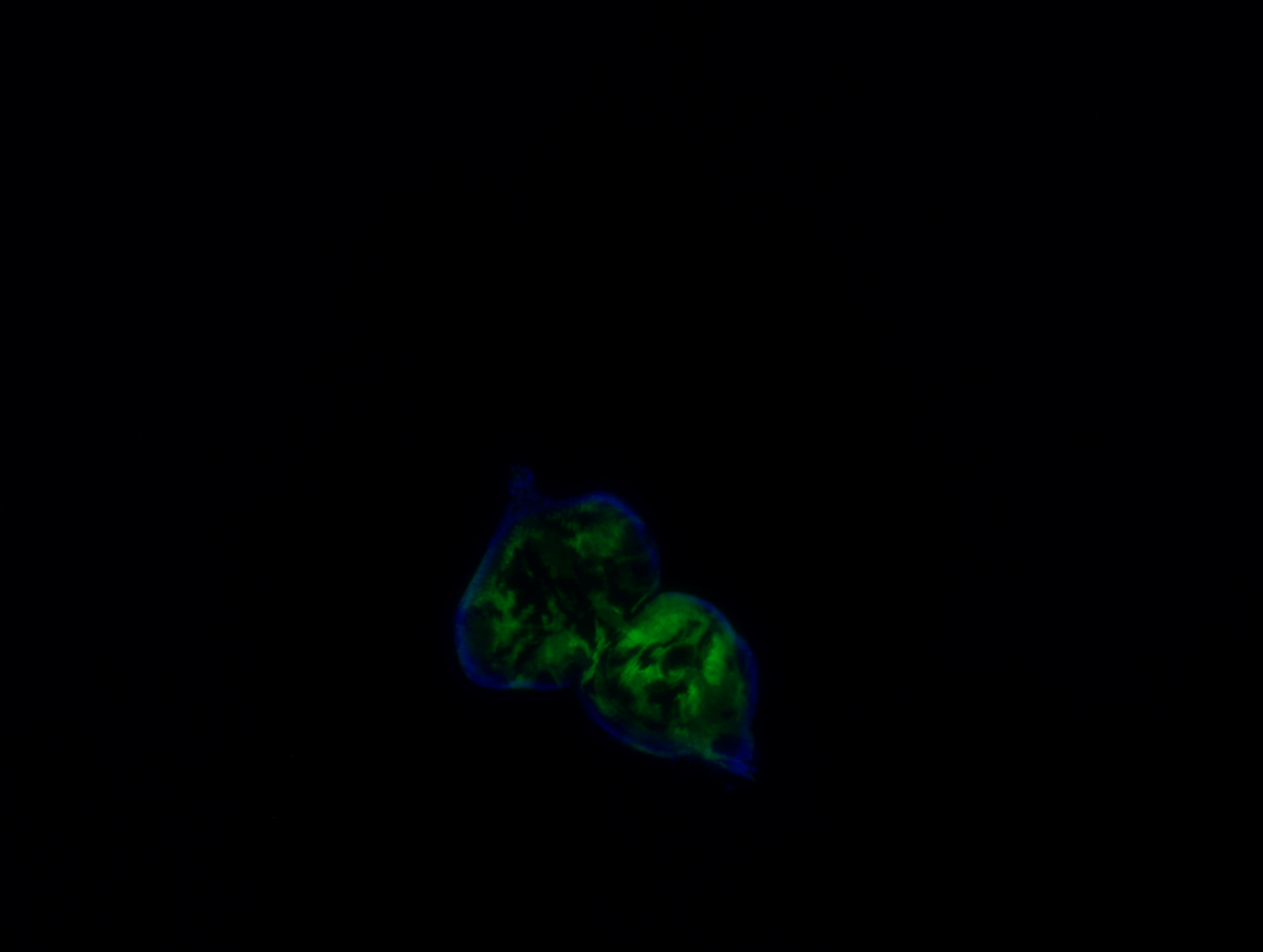

Supplement: Supplementary file 6 — Source data Fig. 2 [file 44318_2025_489_MOESM6_ESM.zip › Figure 2A/5-2 original image.tif]

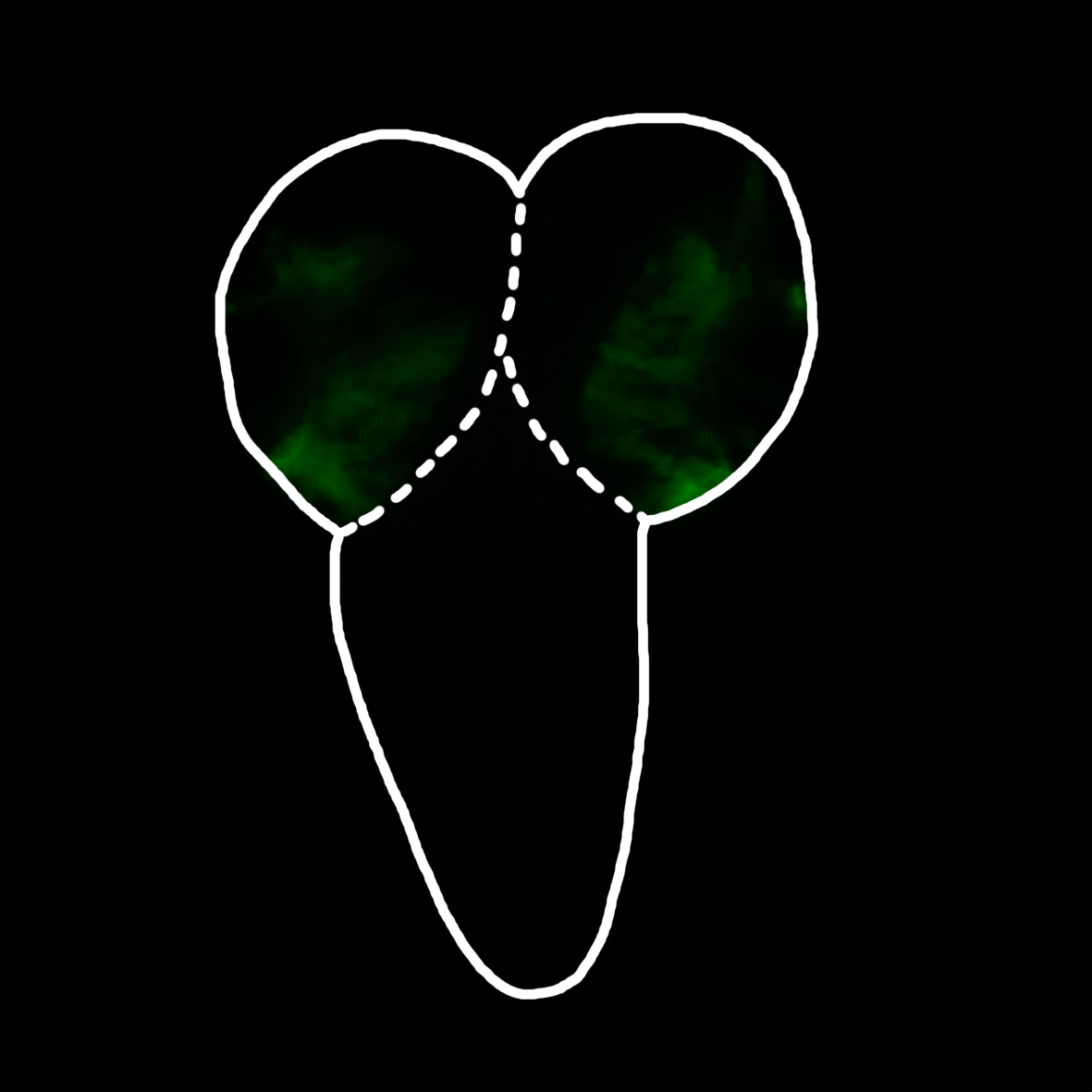

Supplement: Supplementary file 6 — Source data Fig. 2 [file 44318_2025_489_MOESM6_ESM.zip › Figure 2A/6-1 rotated and cut image with border line.tif]

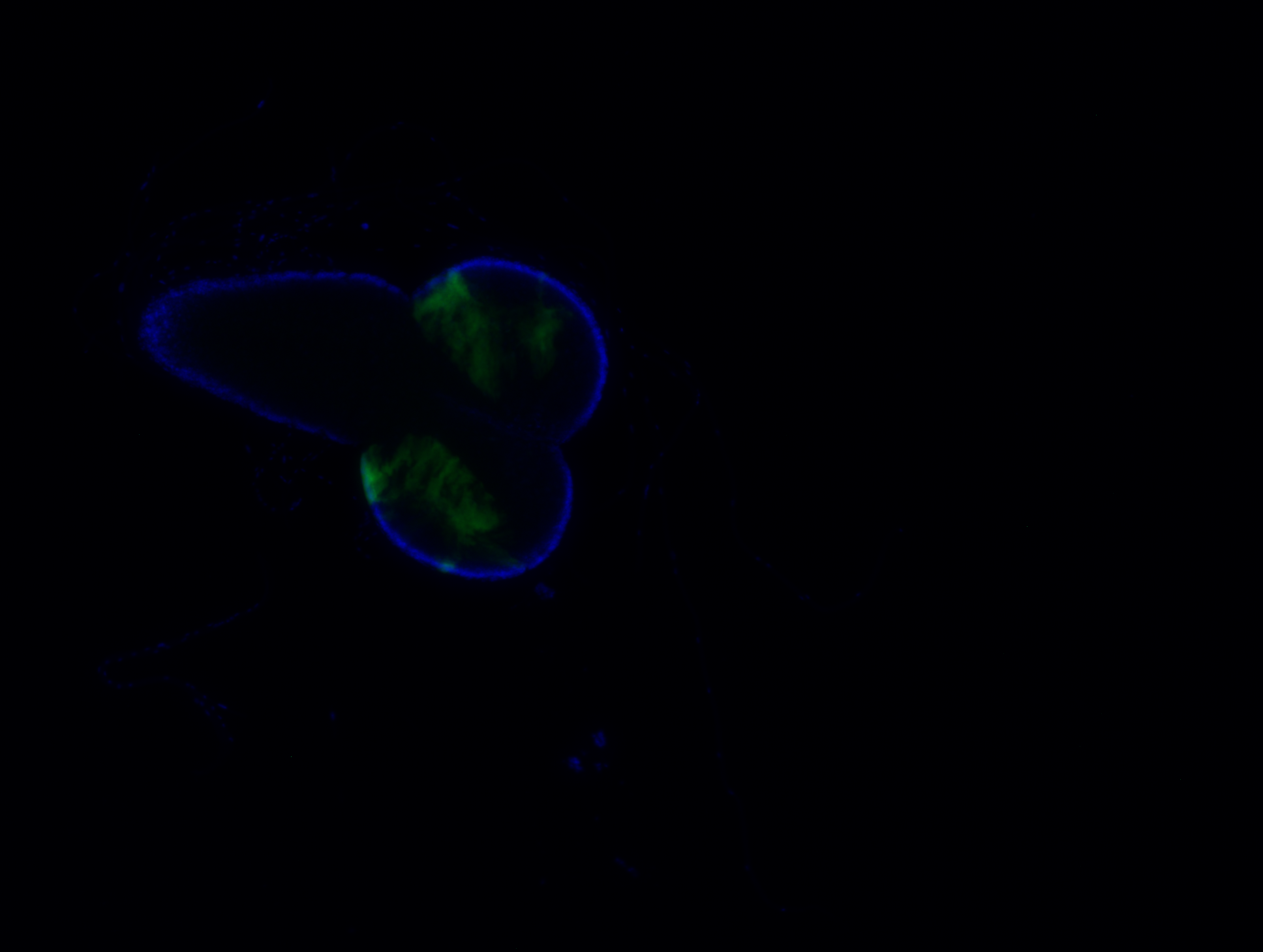

Supplement: Supplementary file 6 — Source data Fig. 2 [file 44318_2025_489_MOESM6_ESM.zip › Figure 2A/6-2 original image.tif]

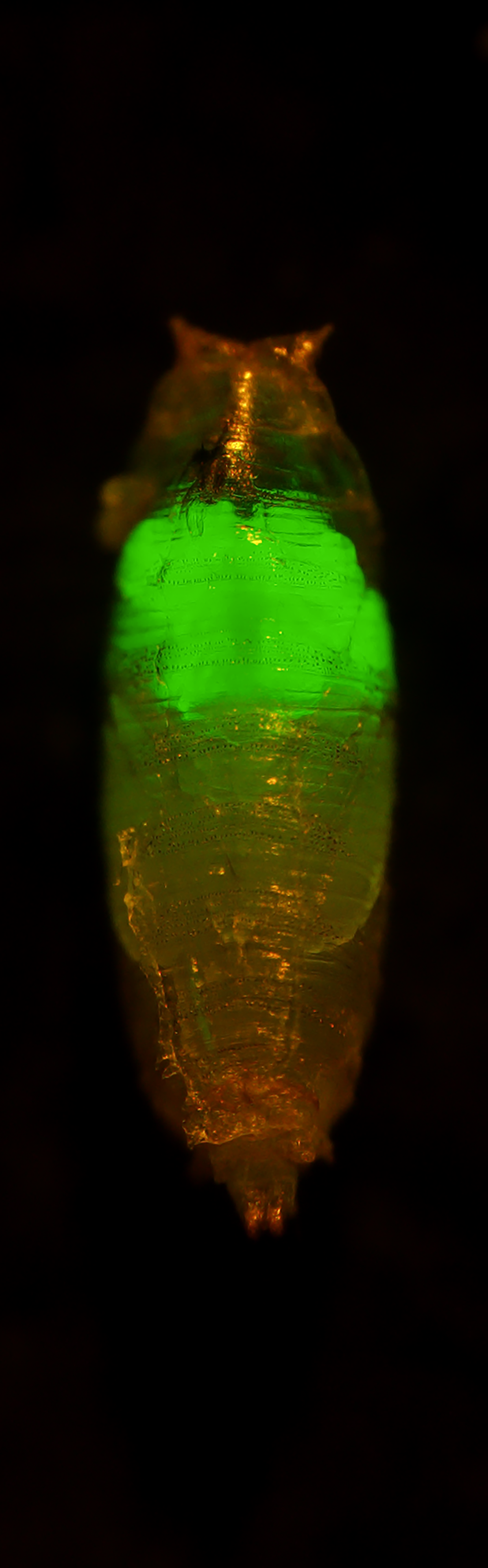

Supplement: Supplementary file 6 — Source data Fig. 2 [file 44318_2025_489_MOESM6_ESM.zip › Figure 2A/7-1 rotated and cut image.tif]

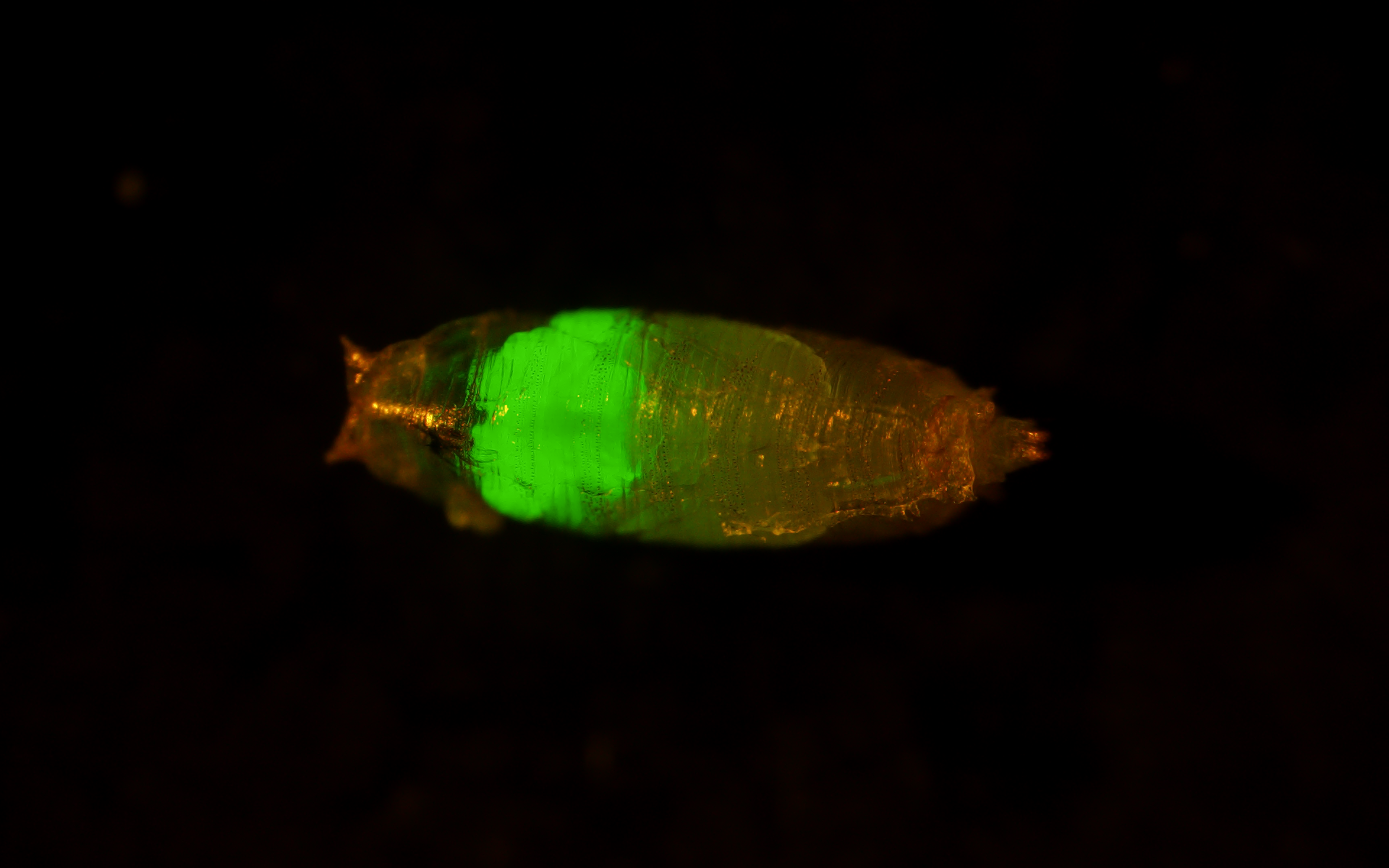

Supplement: Supplementary file 6 — Source data Fig. 2 [file 44318_2025_489_MOESM6_ESM.zip › Figure 2A/7-2 original image.tif]

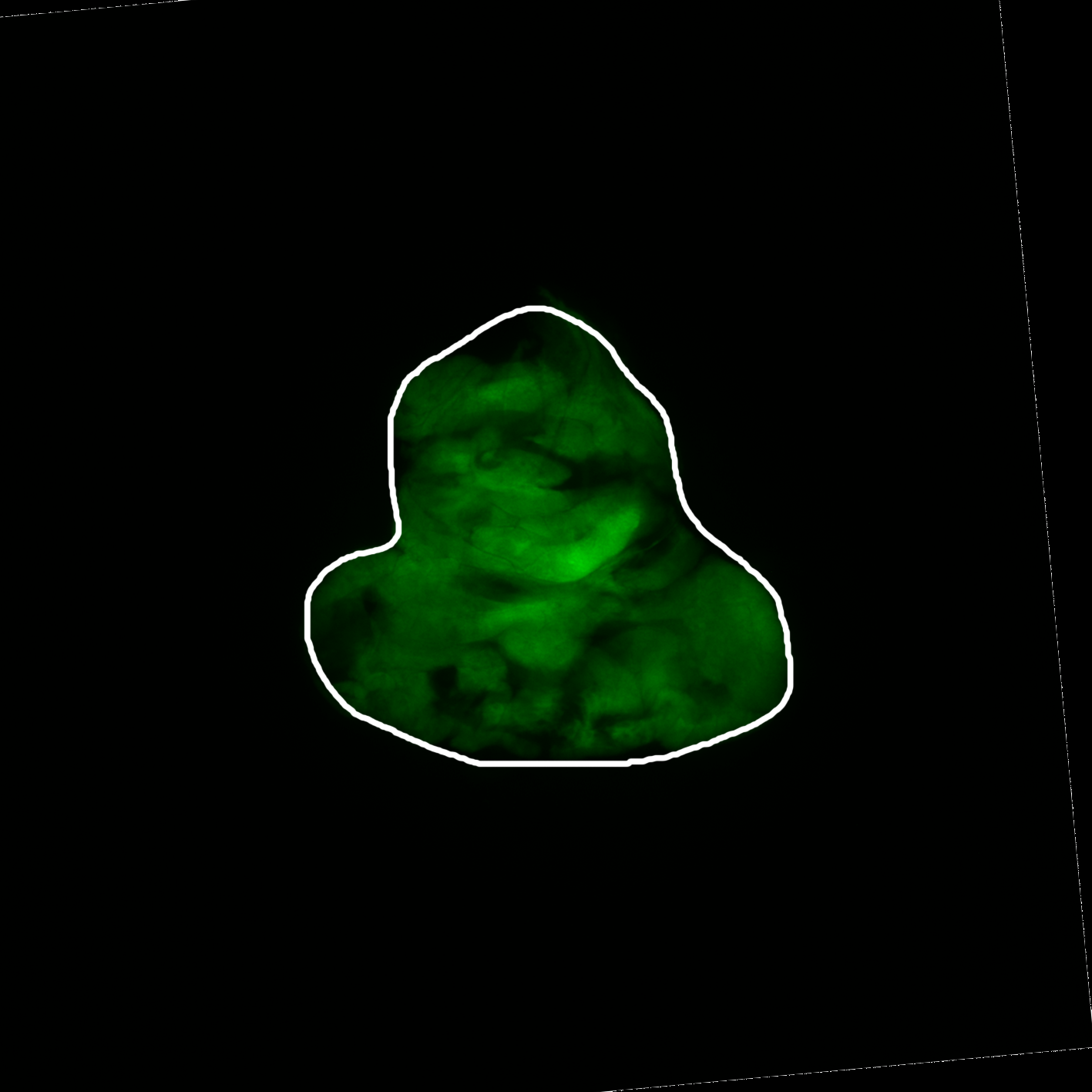

Supplement: Supplementary file 6 — Source data Fig. 2 [file 44318_2025_489_MOESM6_ESM.zip › Figure 2A/8-1 rotated and cut image with border line.tif]

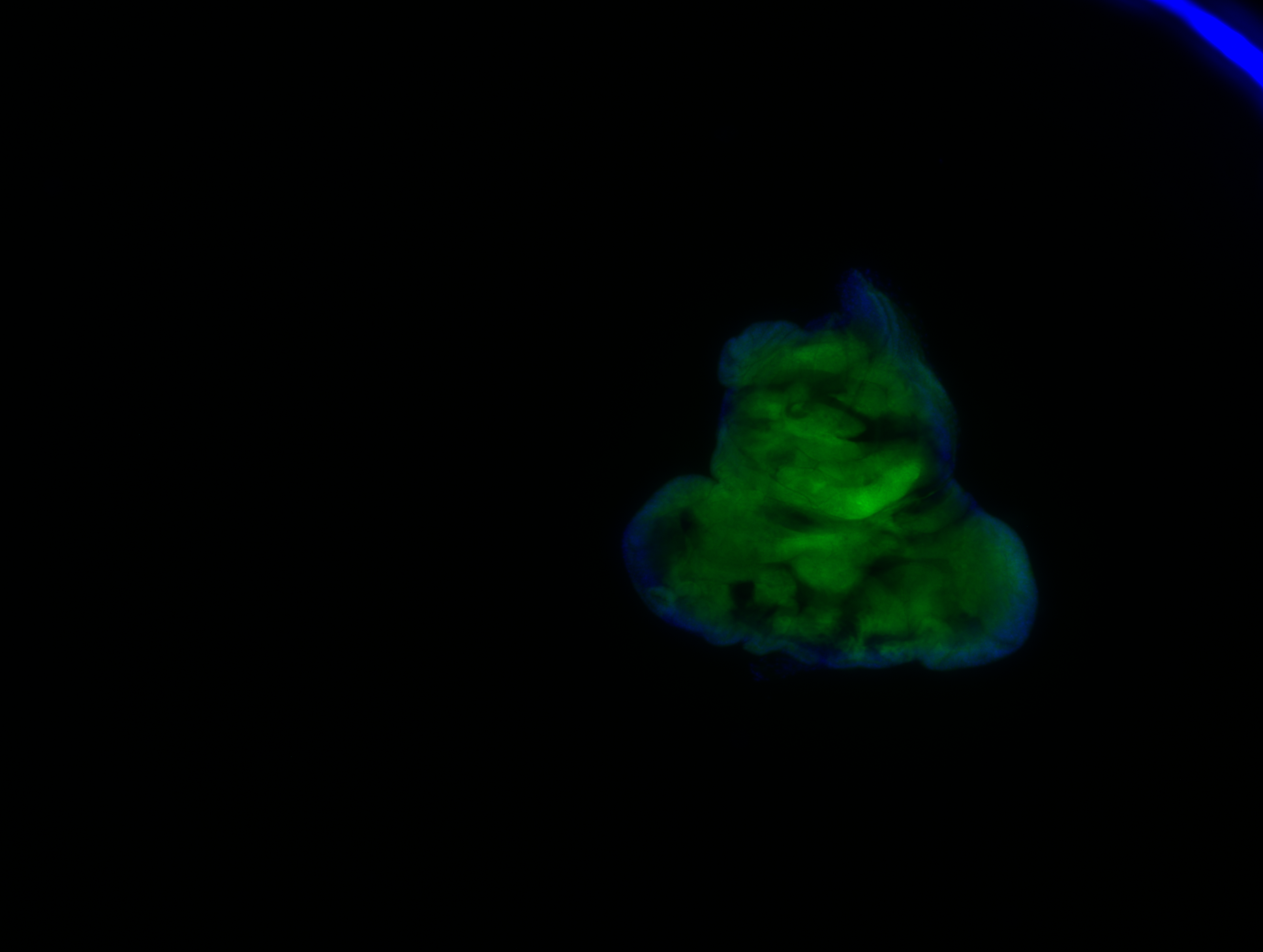

Supplement: Supplementary file 6 — Source data Fig. 2 [file 44318_2025_489_MOESM6_ESM.zip › Figure 2A/8-2 original image.tif]

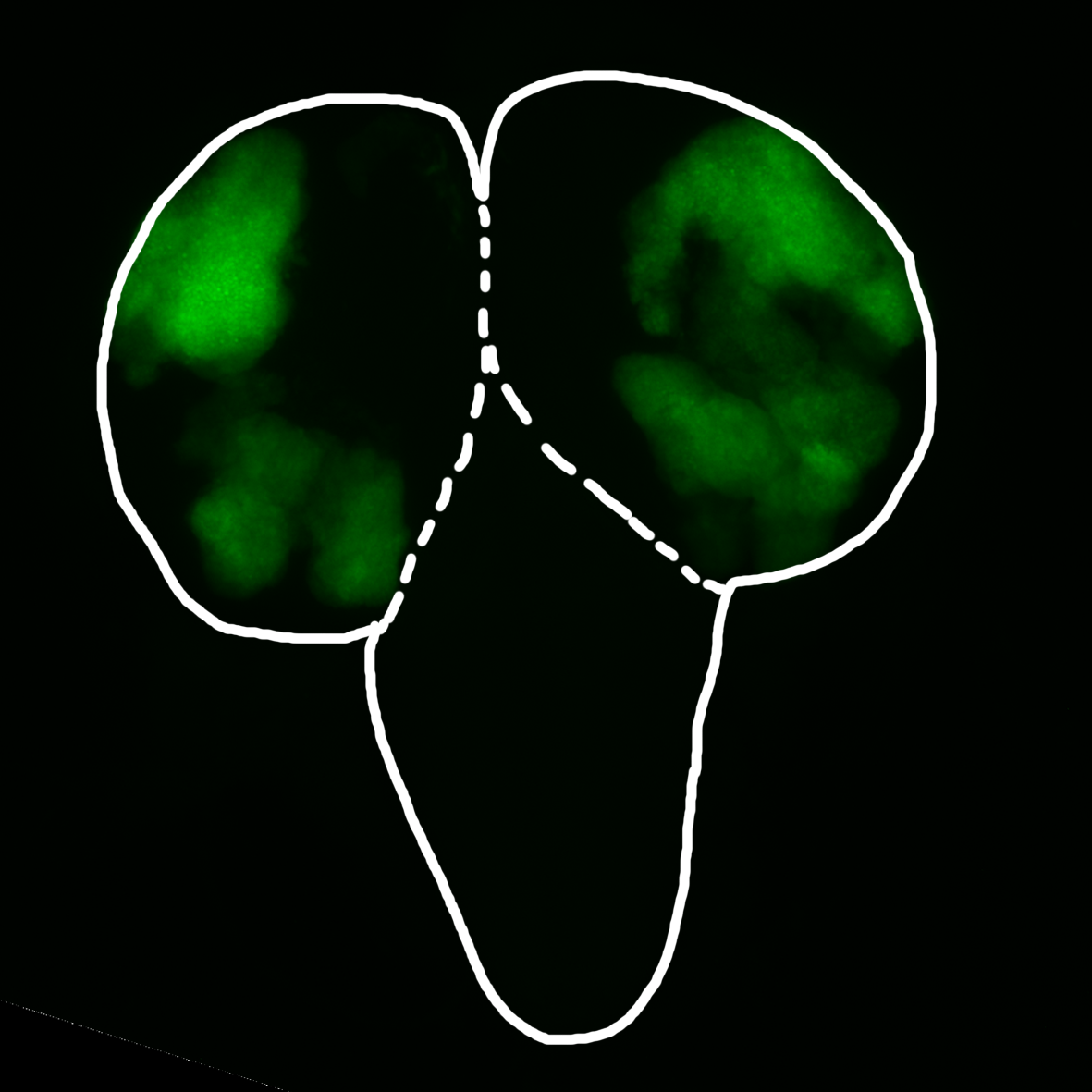

Supplement: Supplementary file 6 — Source data Fig. 2 [file 44318_2025_489_MOESM6_ESM.zip › Figure 2A/9-1 rotated and cut image with border line.tif]

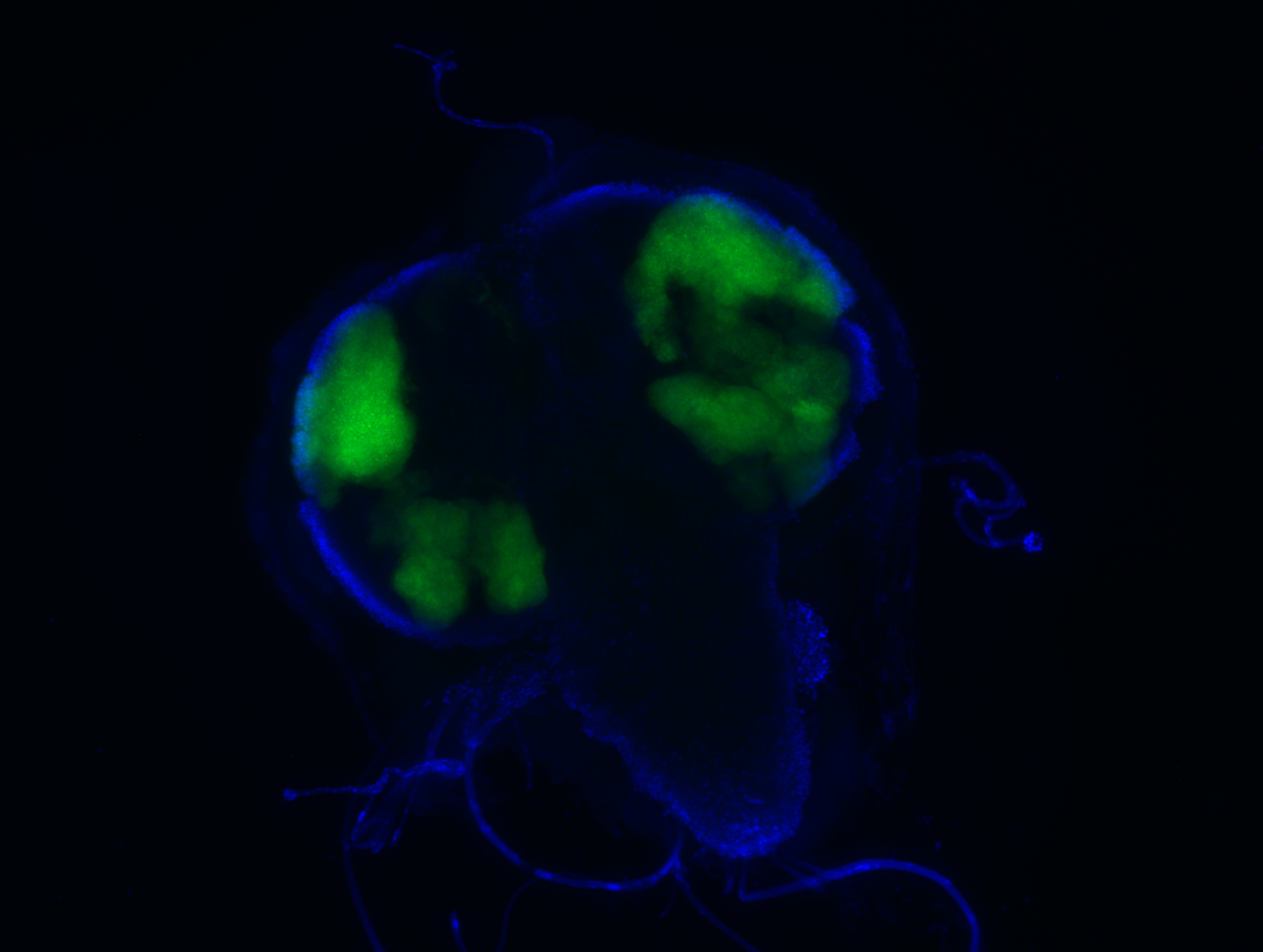

Supplement: Supplementary file 6 — Source data Fig. 2 [file 44318_2025_489_MOESM6_ESM.zip › Figure 2A/9-2 original image.tif]

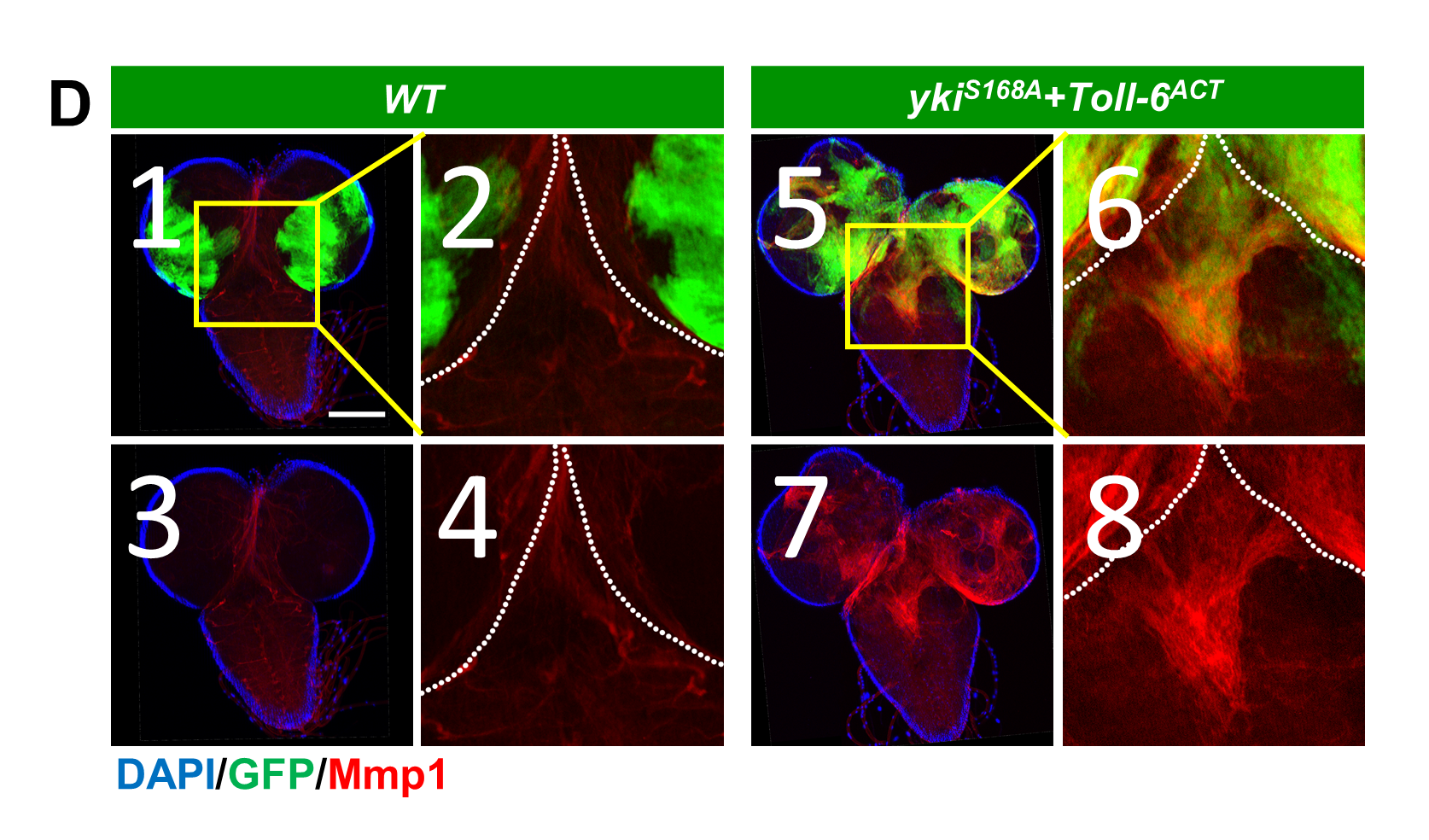

Supplement: Supplementary file 6 — Source data Fig. 2 [file 44318_2025_489_MOESM6_ESM.zip › Figure 2D/0 paper Figure 2D with provided image sequence.tif]

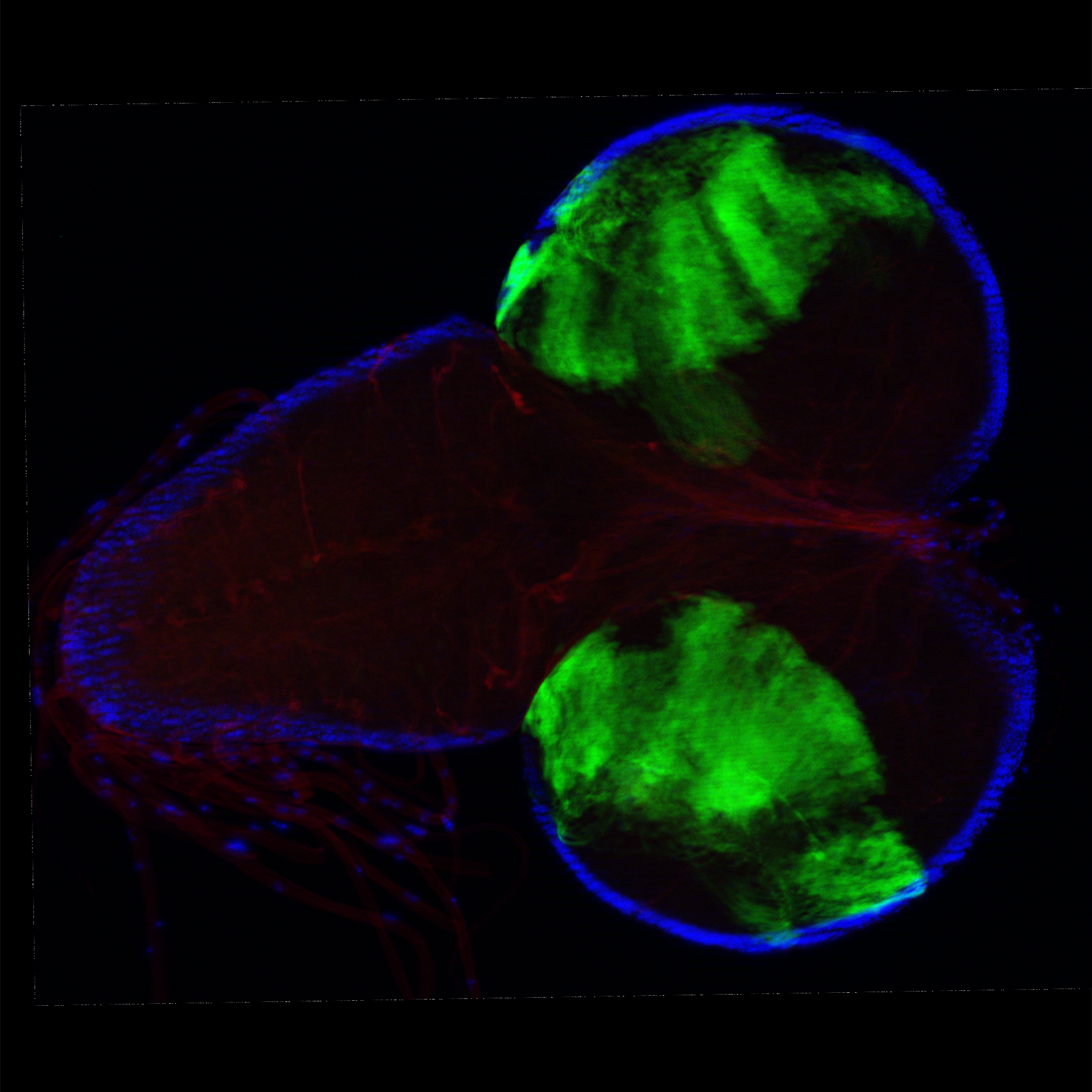

Supplement: Supplementary file 6 — Source data Fig. 2 [file 44318_2025_489_MOESM6_ESM.zip › Figure 2D/1 original image.tif]

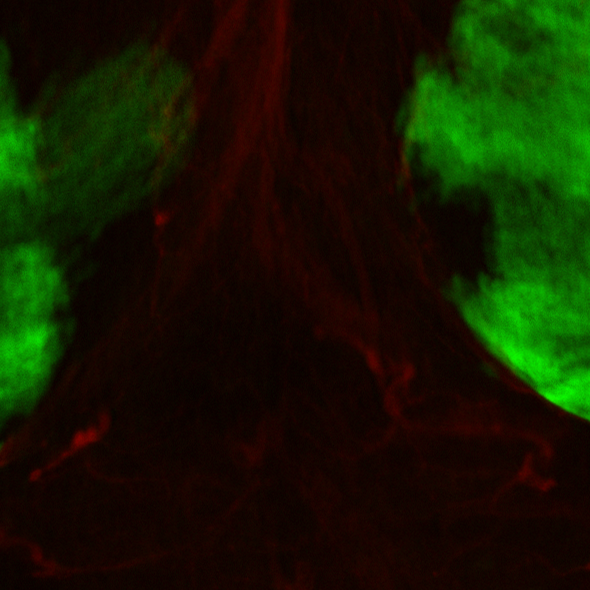

Supplement: Supplementary file 6 — Source data Fig. 2 [file 44318_2025_489_MOESM6_ESM.zip › Figure 2D/2 original image.tif]

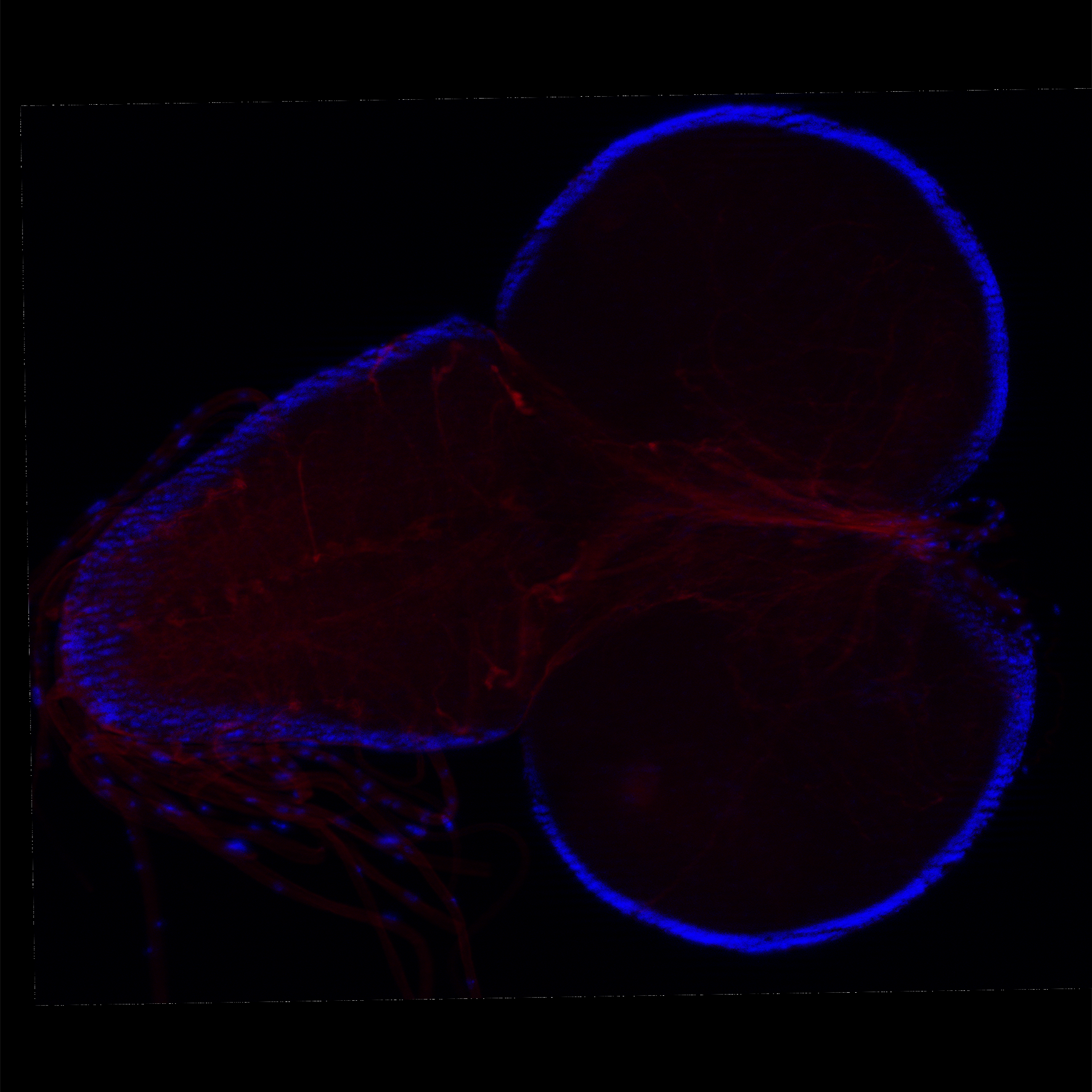

Supplement: Supplementary file 6 — Source data Fig. 2 [file 44318_2025_489_MOESM6_ESM.zip › Figure 2D/3 original image.tif]

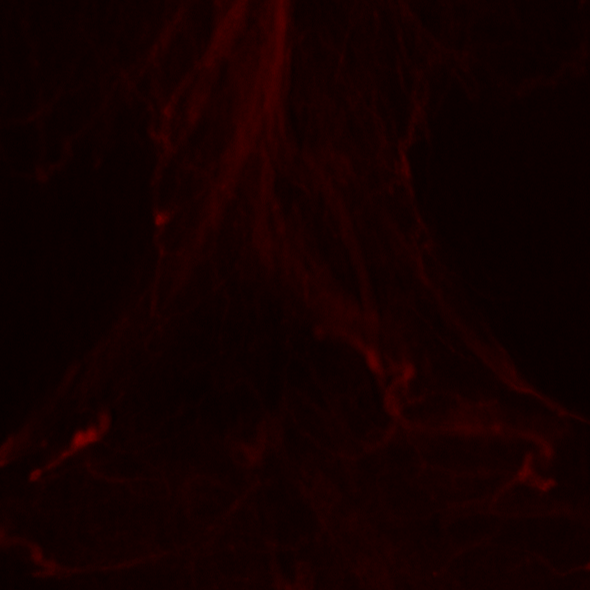

Supplement: Supplementary file 6 — Source data Fig. 2 [file 44318_2025_489_MOESM6_ESM.zip › Figure 2D/4 original image.tif]

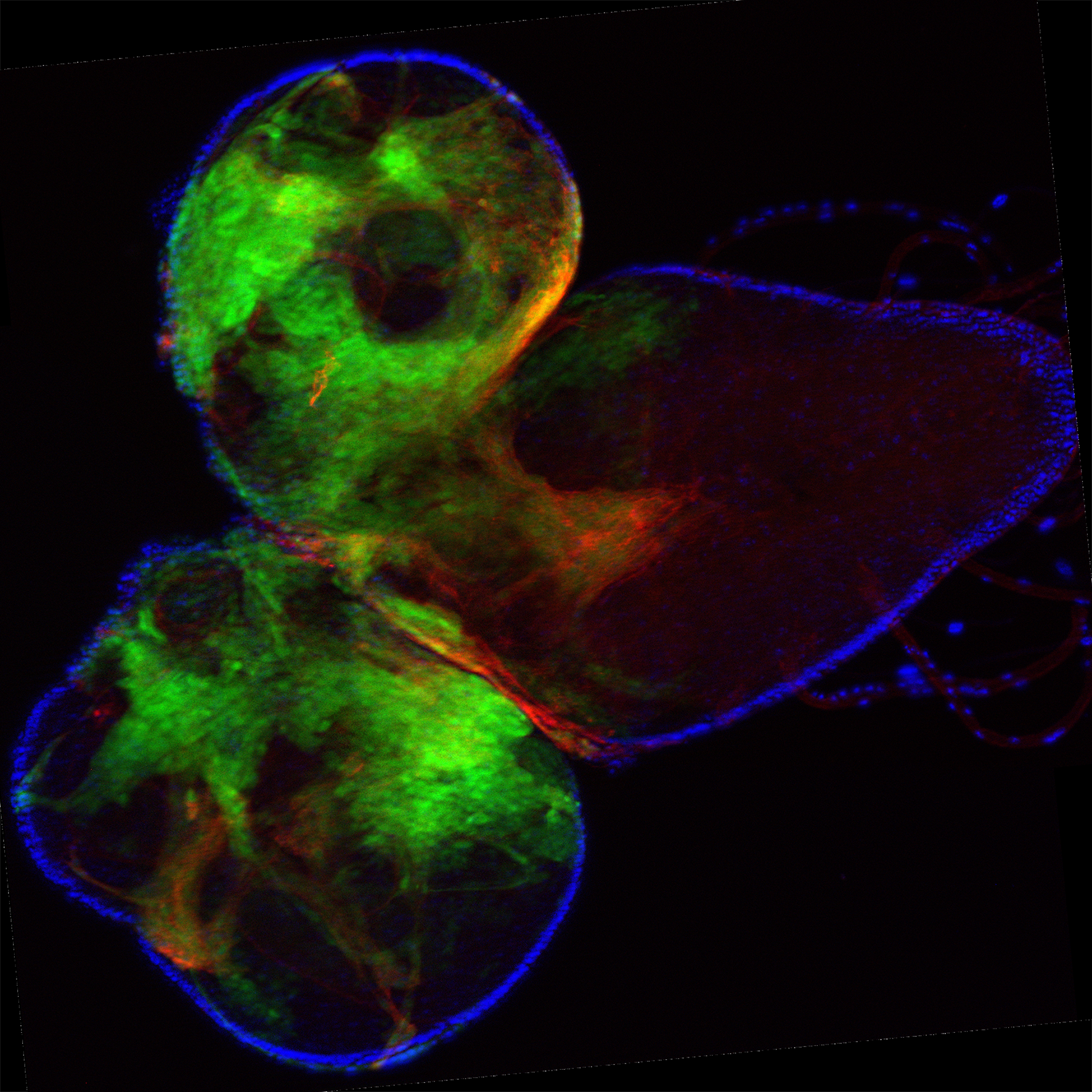

Supplement: Supplementary file 6 — Source data Fig. 2 [file 44318_2025_489_MOESM6_ESM.zip › Figure 2D/5 original image.tif]

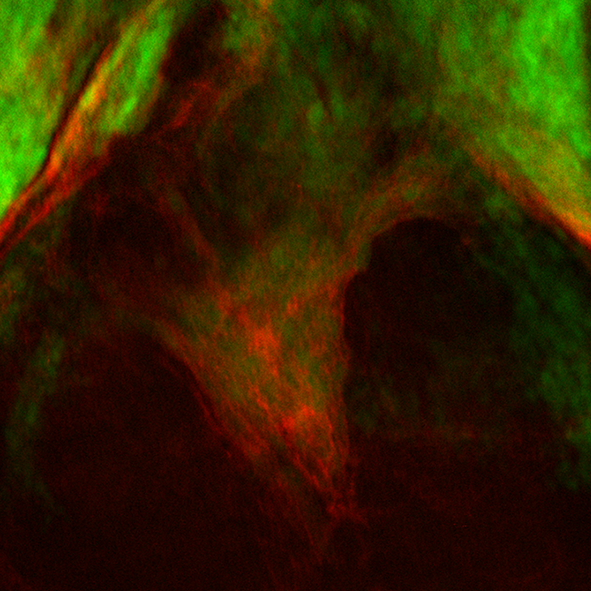

Supplement: Supplementary file 6 — Source data Fig. 2 [file 44318_2025_489_MOESM6_ESM.zip › Figure 2D/6 original image.tif]

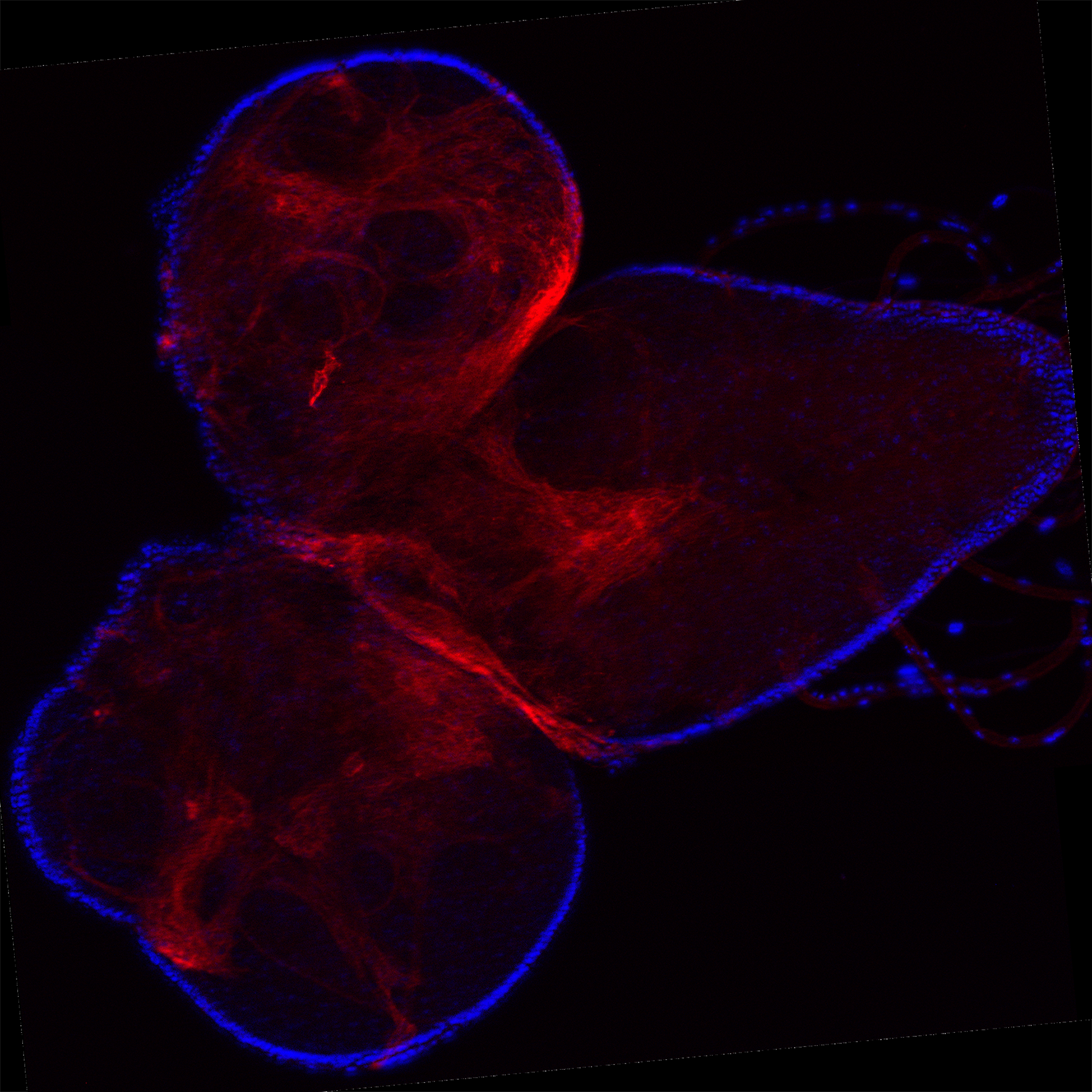

Supplement: Supplementary file 6 — Source data Fig. 2 [file 44318_2025_489_MOESM6_ESM.zip › Figure 2D/7 original image.tif]

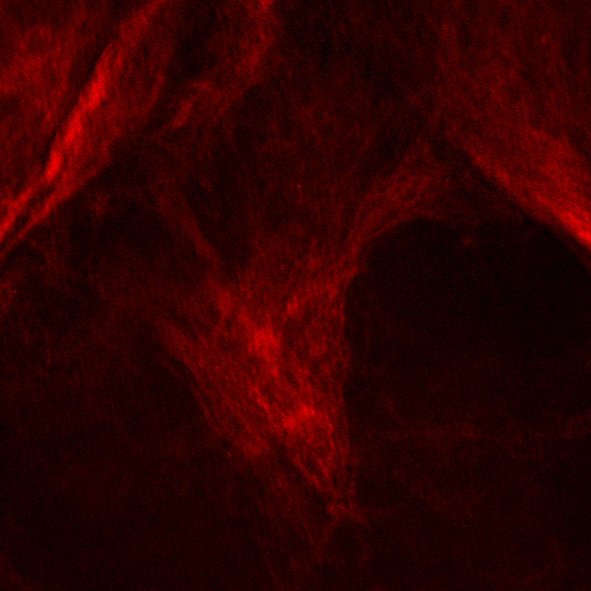

Supplement: Supplementary file 6 — Source data Fig. 2 [file 44318_2025_489_MOESM6_ESM.zip › Figure 2D/8 image with border line.tif]

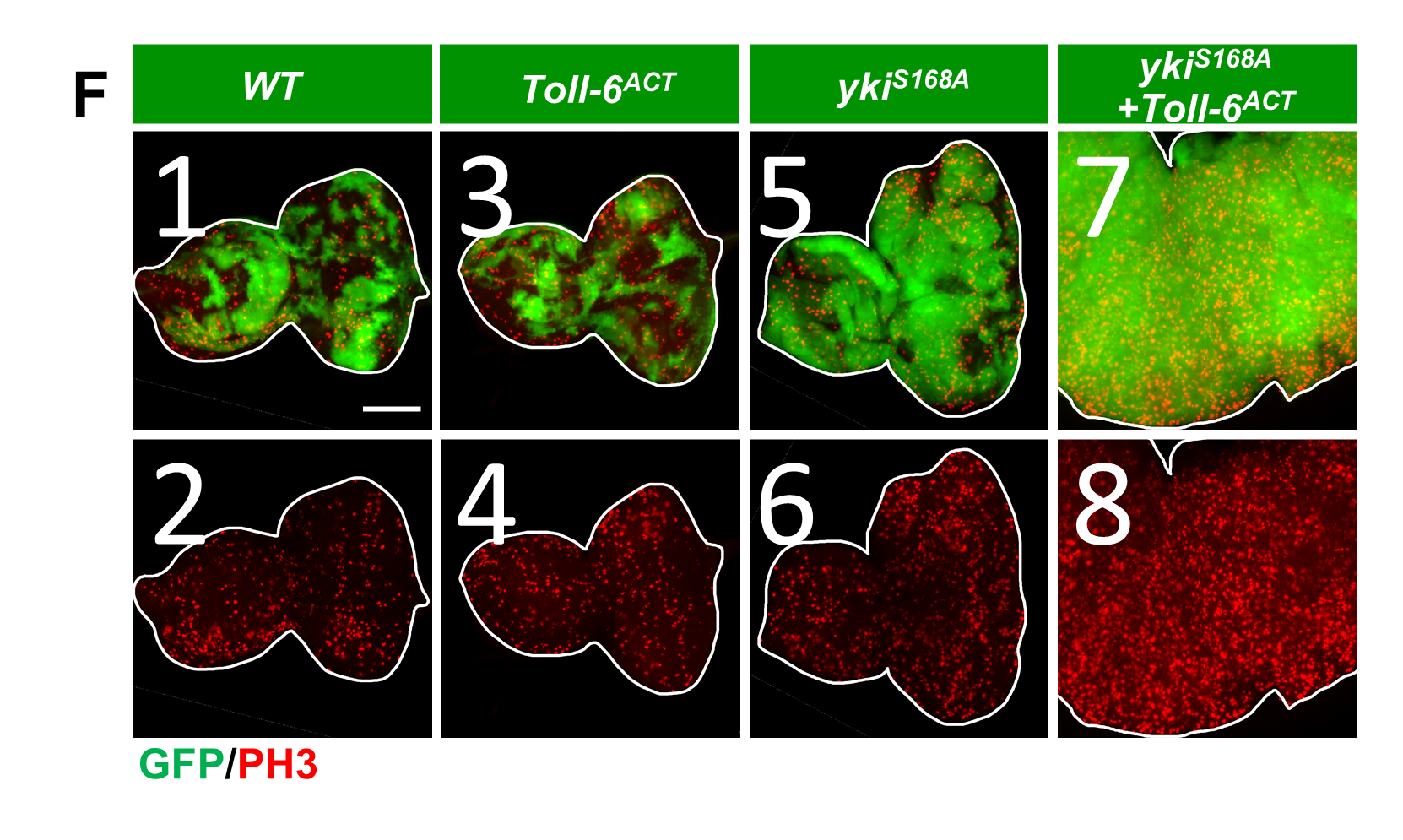

Supplement: Supplementary file 6 — Source data Fig. 2 [file 44318_2025_489_MOESM6_ESM.zip › Figure 2F/0 paper Figure 2F with provided image sequence.tif]

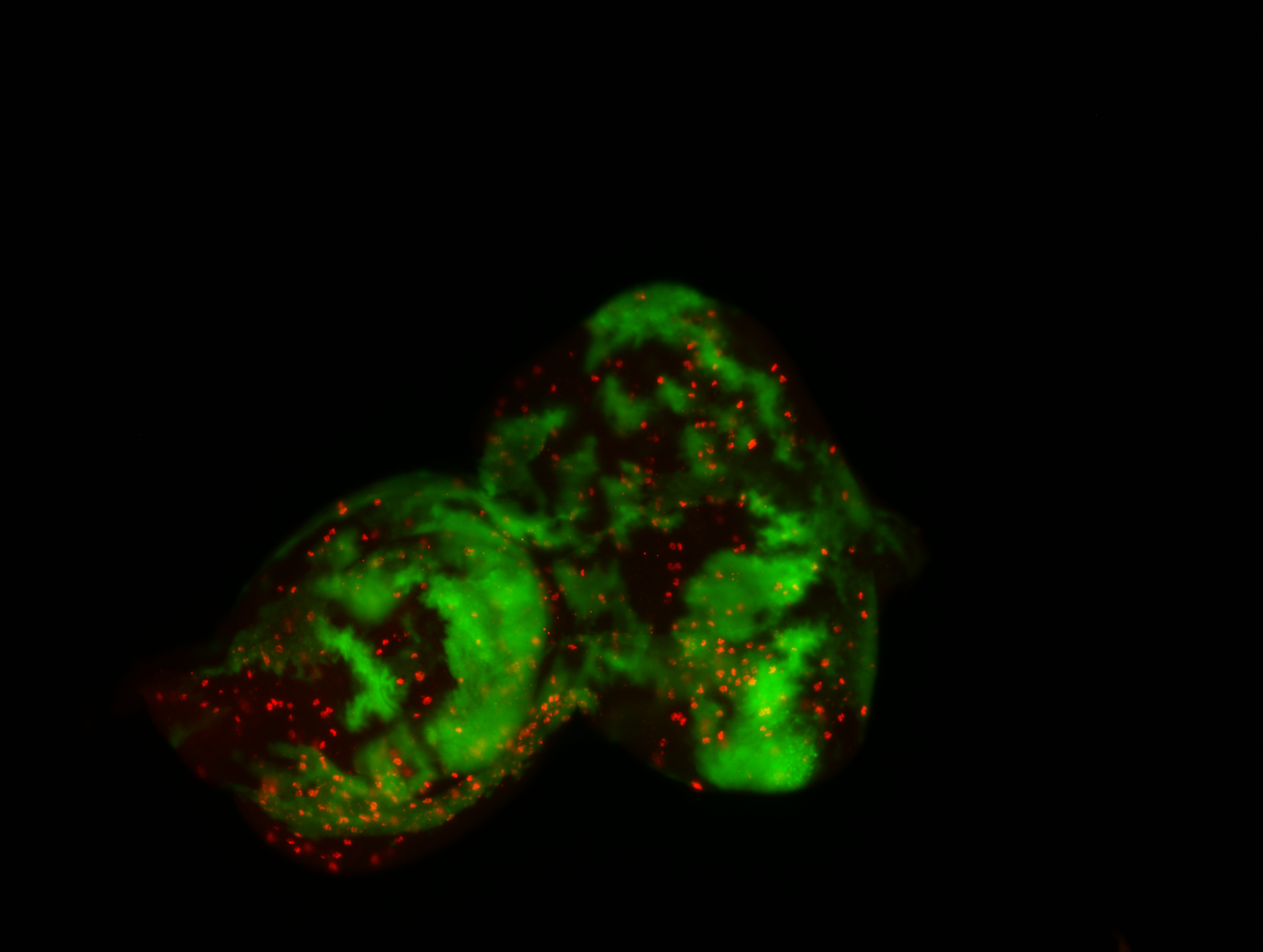

Supplement: Supplementary file 6 — Source data Fig. 2 [file 44318_2025_489_MOESM6_ESM.zip › Figure 2F/1 original image.tif]

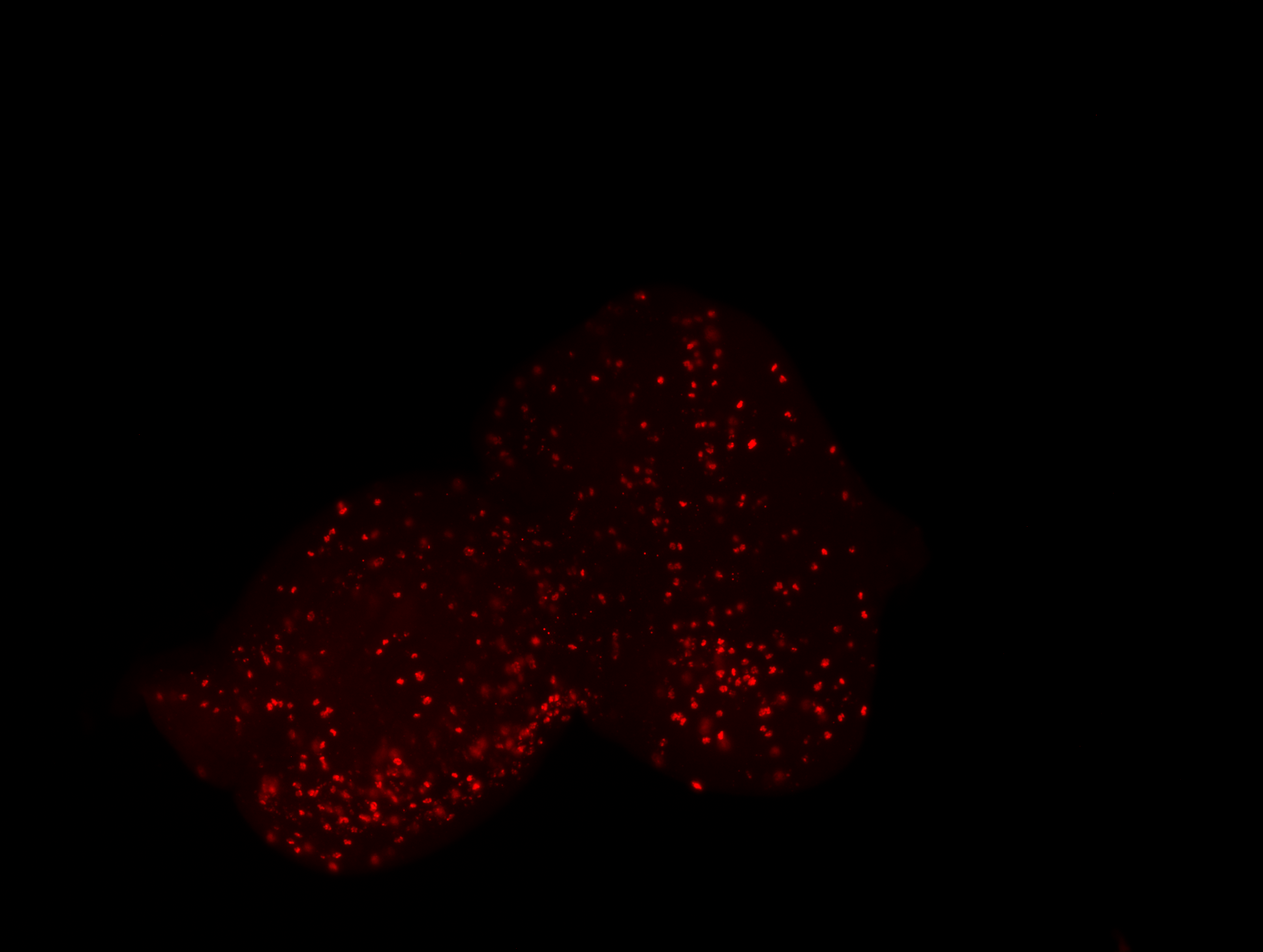

Supplement: Supplementary file 6 — Source data Fig. 2 [file 44318_2025_489_MOESM6_ESM.zip › Figure 2F/2 original image.tif]

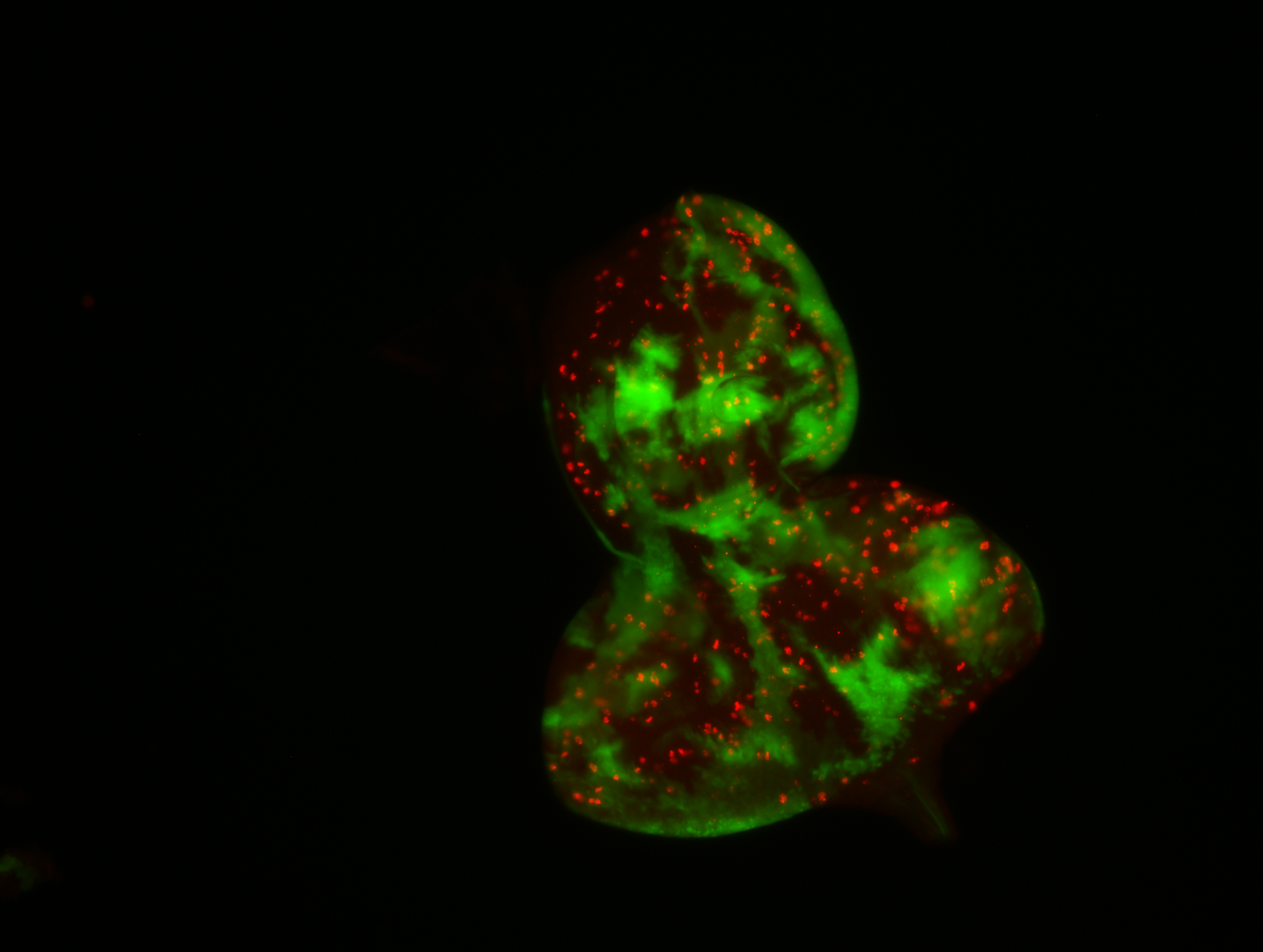

Supplement: Supplementary file 6 — Source data Fig. 2 [file 44318_2025_489_MOESM6_ESM.zip › Figure 2F/3 original image.tif]

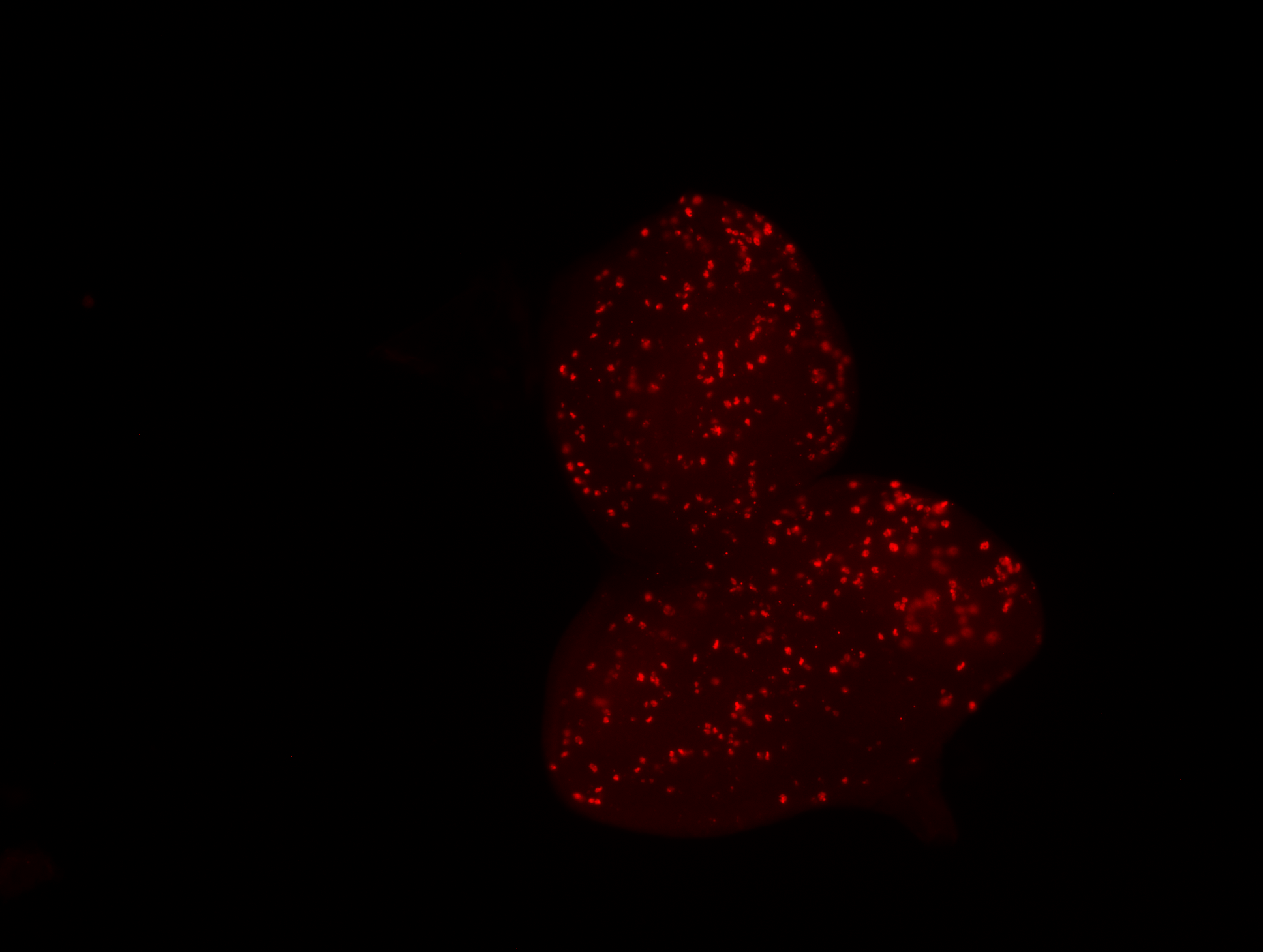

Supplement: Supplementary file 6 — Source data Fig. 2 [file 44318_2025_489_MOESM6_ESM.zip › Figure 2F/4 original image.tif]

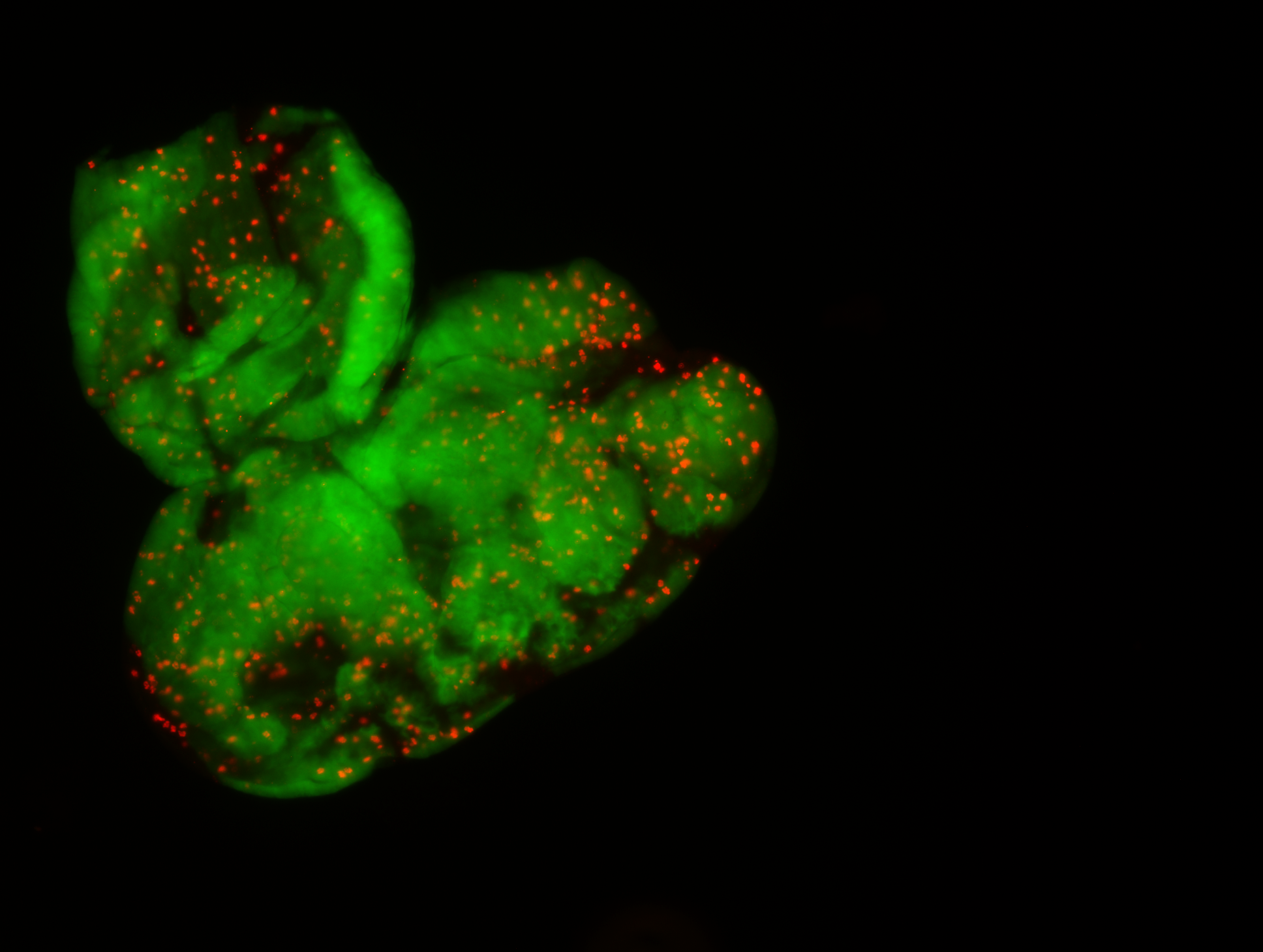

Supplement: Supplementary file 6 — Source data Fig. 2 [file 44318_2025_489_MOESM6_ESM.zip › Figure 2F/5 original image.tif]

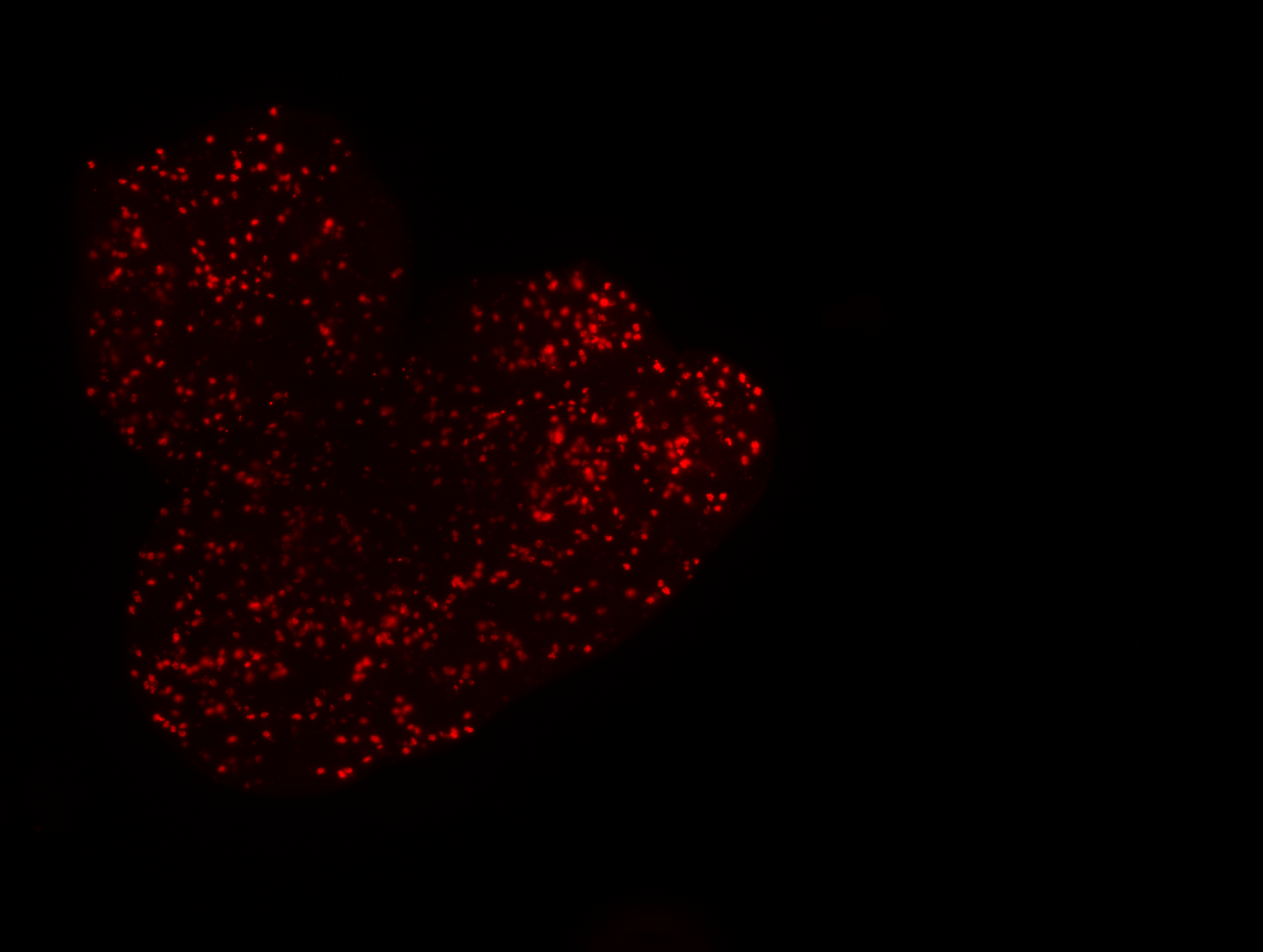

Supplement: Supplementary file 6 — Source data Fig. 2 [file 44318_2025_489_MOESM6_ESM.zip › Figure 2F/6 original image.tif]

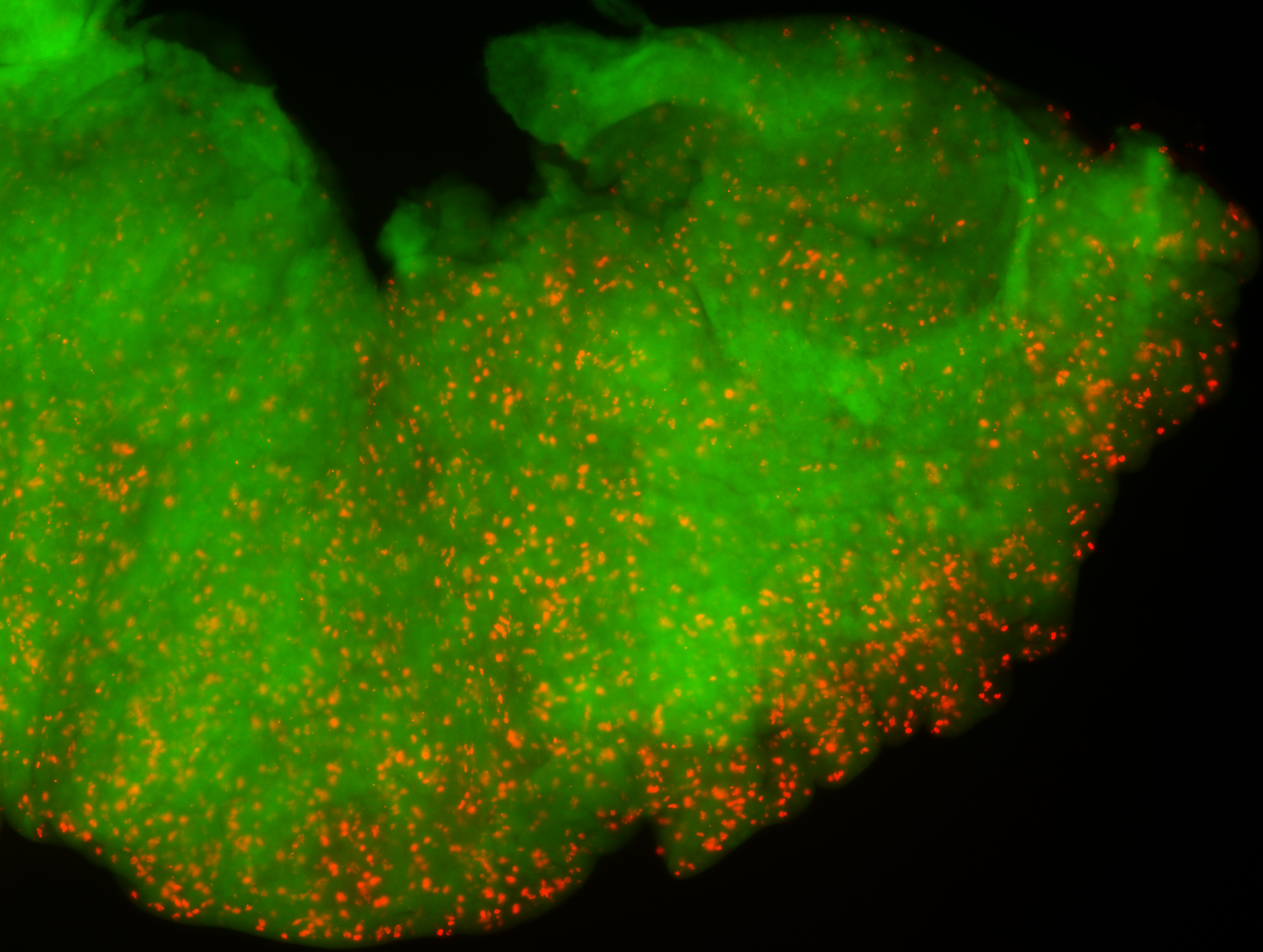

Supplement: Supplementary file 6 — Source data Fig. 2 [file 44318_2025_489_MOESM6_ESM.zip › Figure 2F/7 original image.tif]

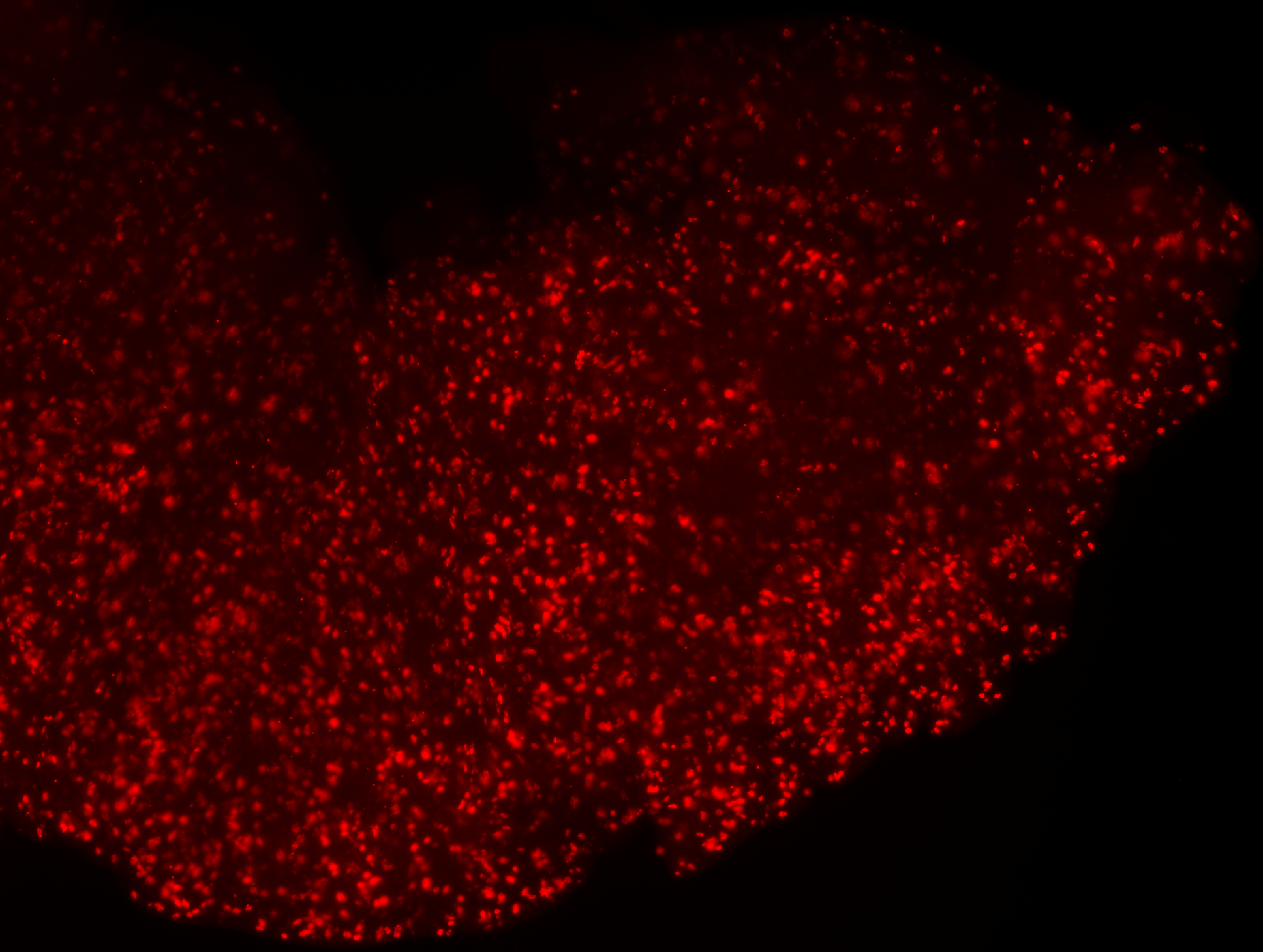

Supplement: Supplementary file 6 — Source data Fig. 2 [file 44318_2025_489_MOESM6_ESM.zip › Figure 2F/8 original image.tif]

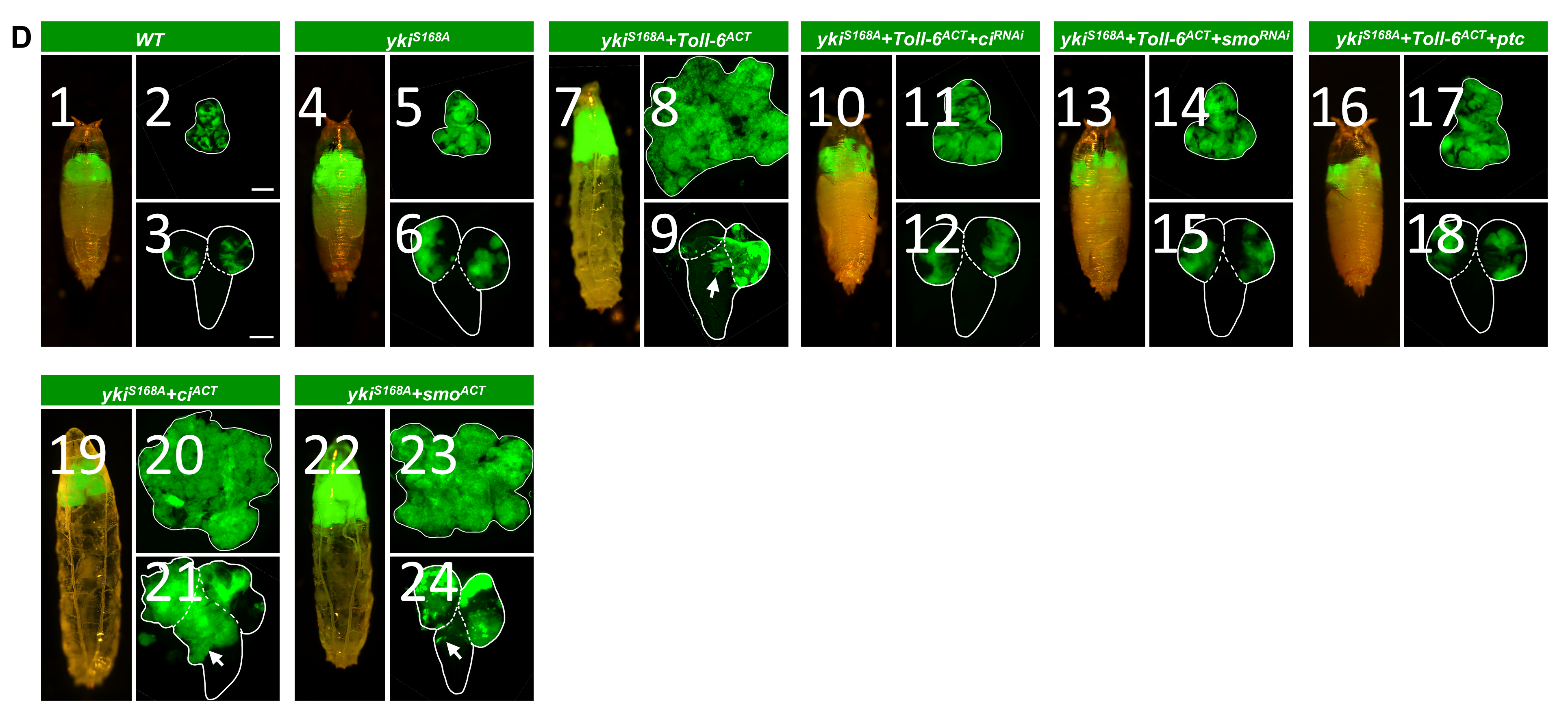

Supplement: Supplementary file 7 — Source data Fig. 3 [file 44318_2025_489_MOESM7_ESM.zip › Figure 3D/0 paper Figure 3D with provided image sequence.tif]

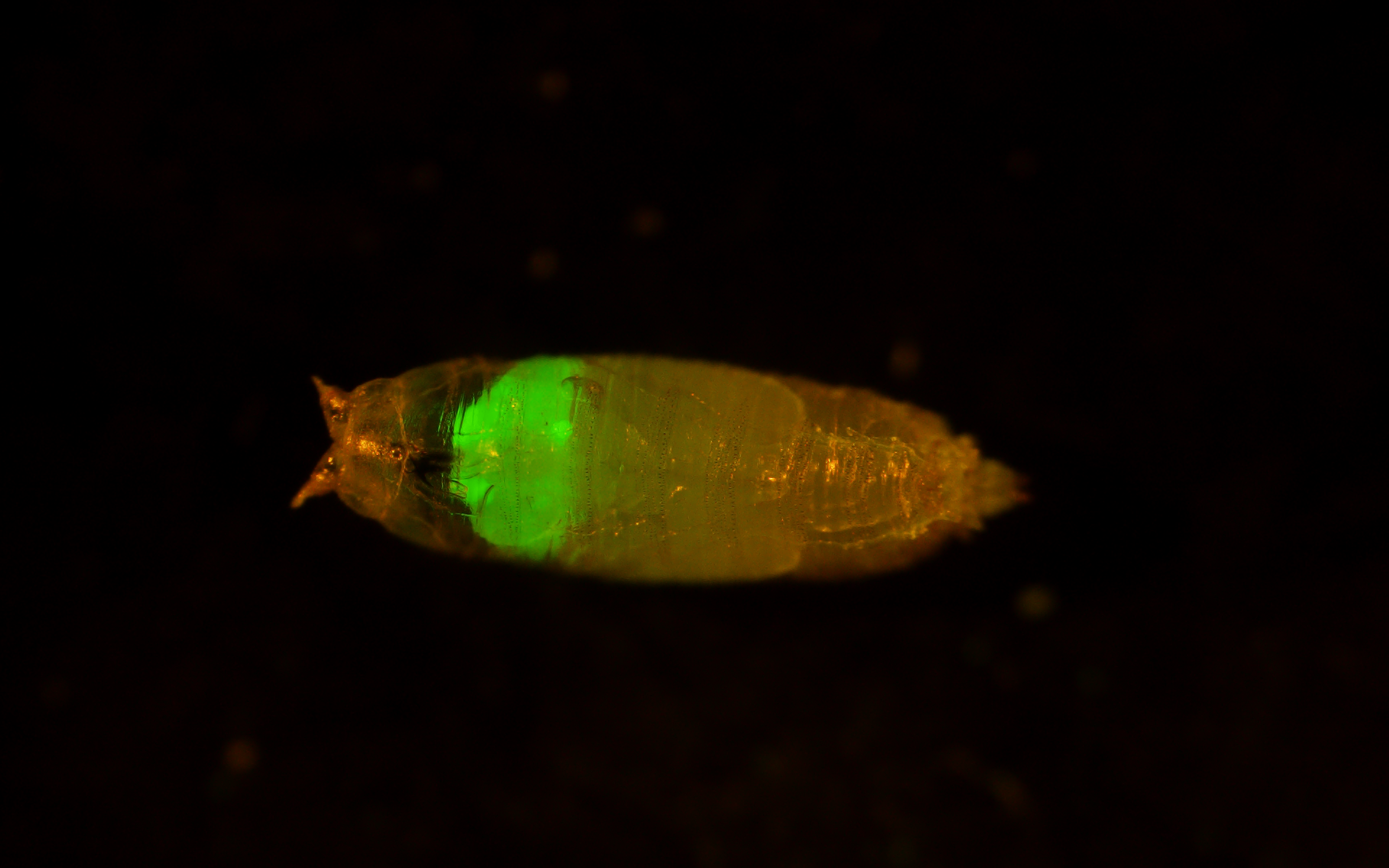

Supplement: Supplementary file 7 — Source data Fig. 3 [file 44318_2025_489_MOESM7_ESM.zip › Figure 3D/1 original image.tif]

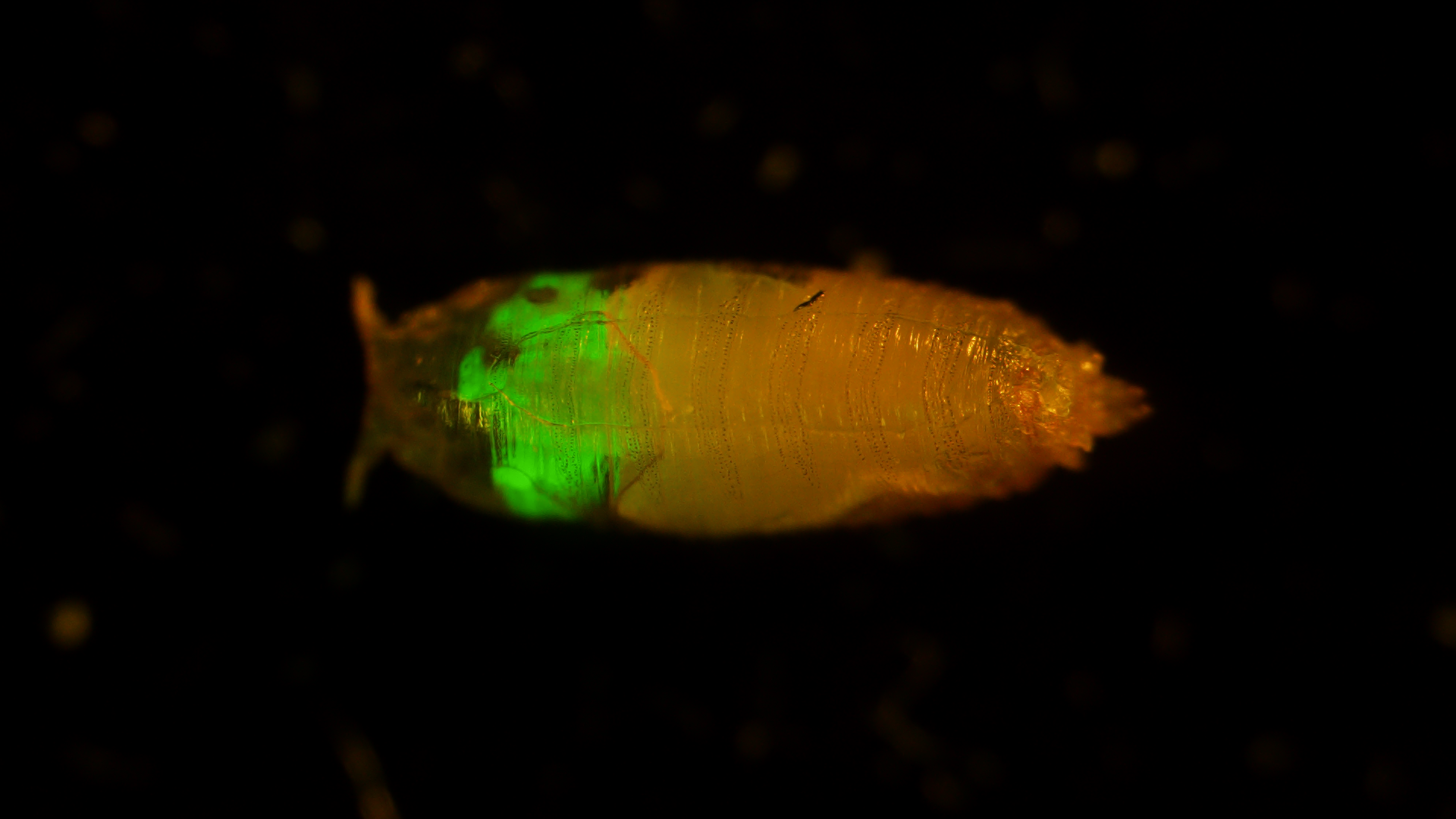

Supplement: Supplementary file 7 — Source data Fig. 3 [file 44318_2025_489_MOESM7_ESM.zip › Figure 3D/10 original image.tif]

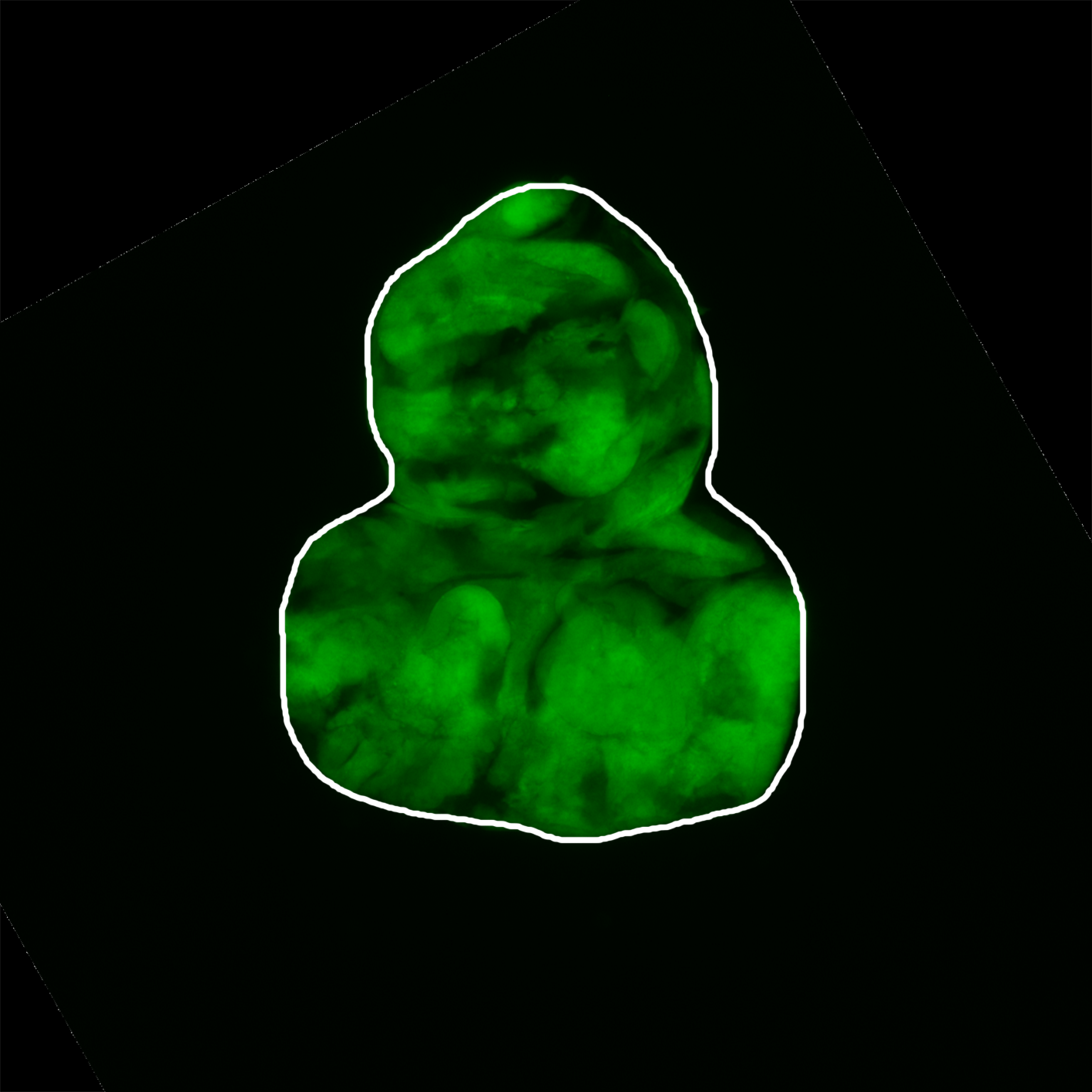

Supplement: Supplementary file 7 — Source data Fig. 3 [file 44318_2025_489_MOESM7_ESM.zip › Figure 3D/11-1 rotated and cut image with border line.tif]

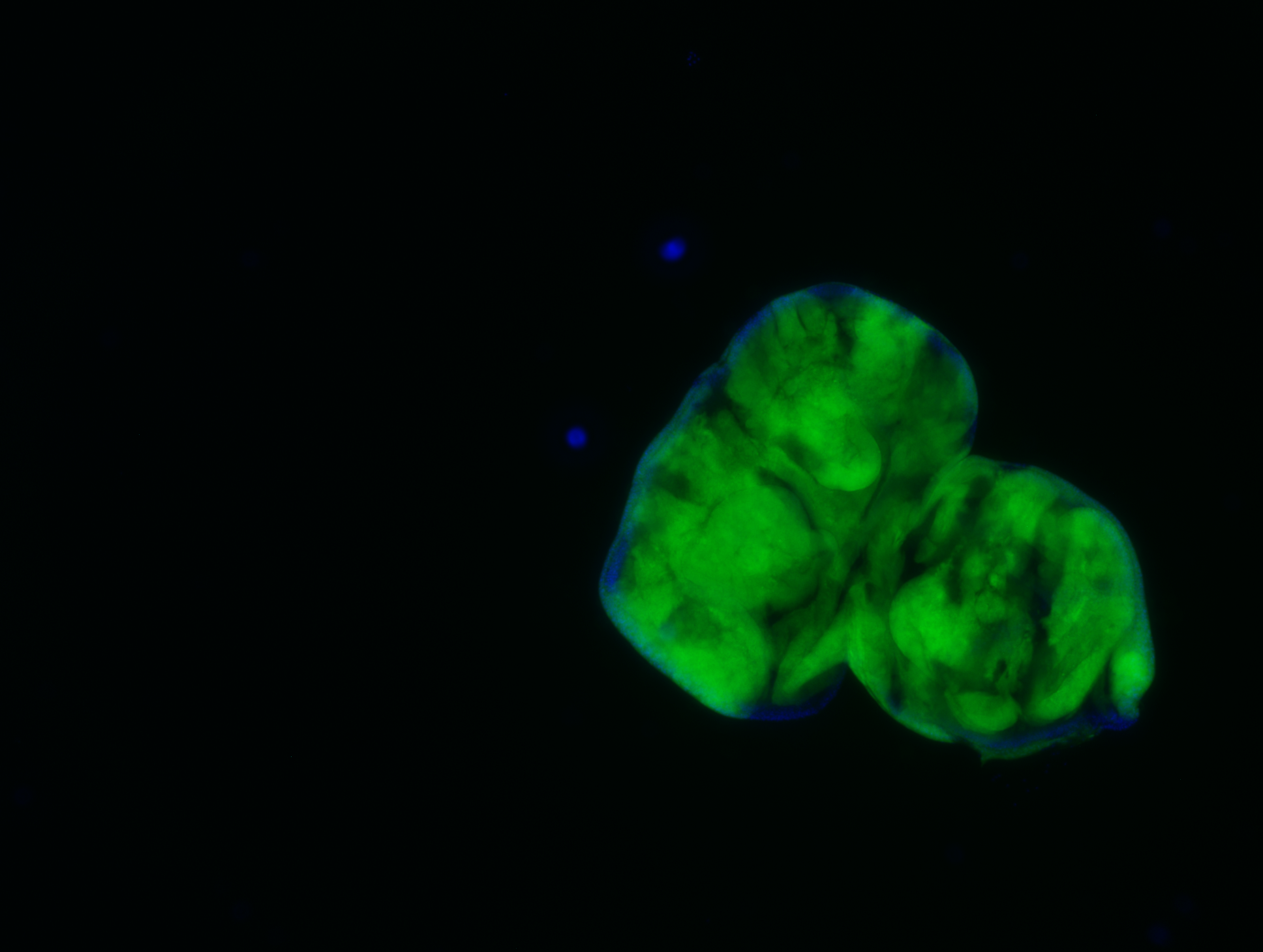

Supplement: Supplementary file 7 — Source data Fig. 3 [file 44318_2025_489_MOESM7_ESM.zip › Figure 3D/11-2 original image.tif]

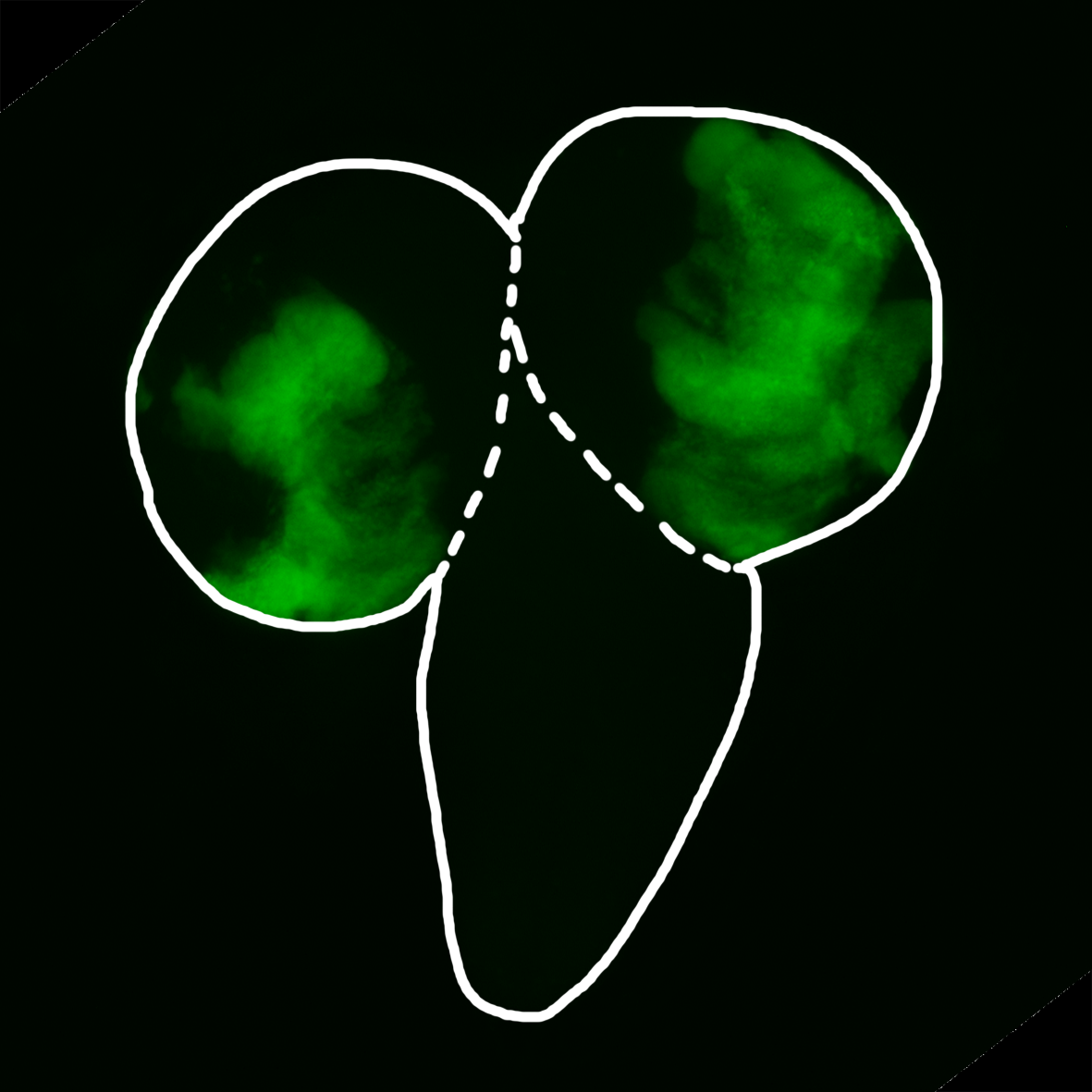

Supplement: Supplementary file 7 — Source data Fig. 3 [file 44318_2025_489_MOESM7_ESM.zip › Figure 3D/12-1 rotated and cut image with border line.tif]

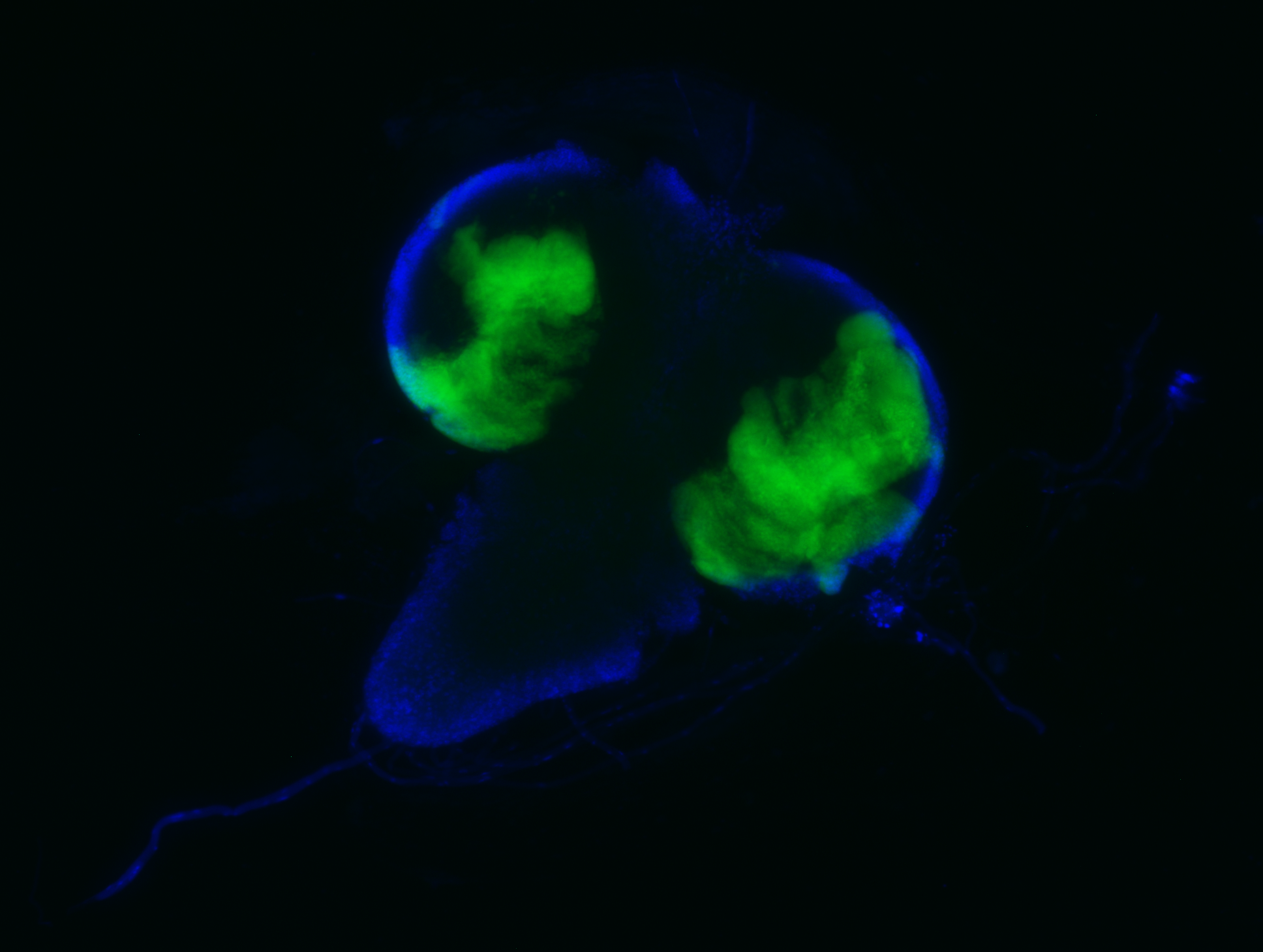

Supplement: Supplementary file 7 — Source data Fig. 3 [file 44318_2025_489_MOESM7_ESM.zip › Figure 3D/12-2 original image.tif]

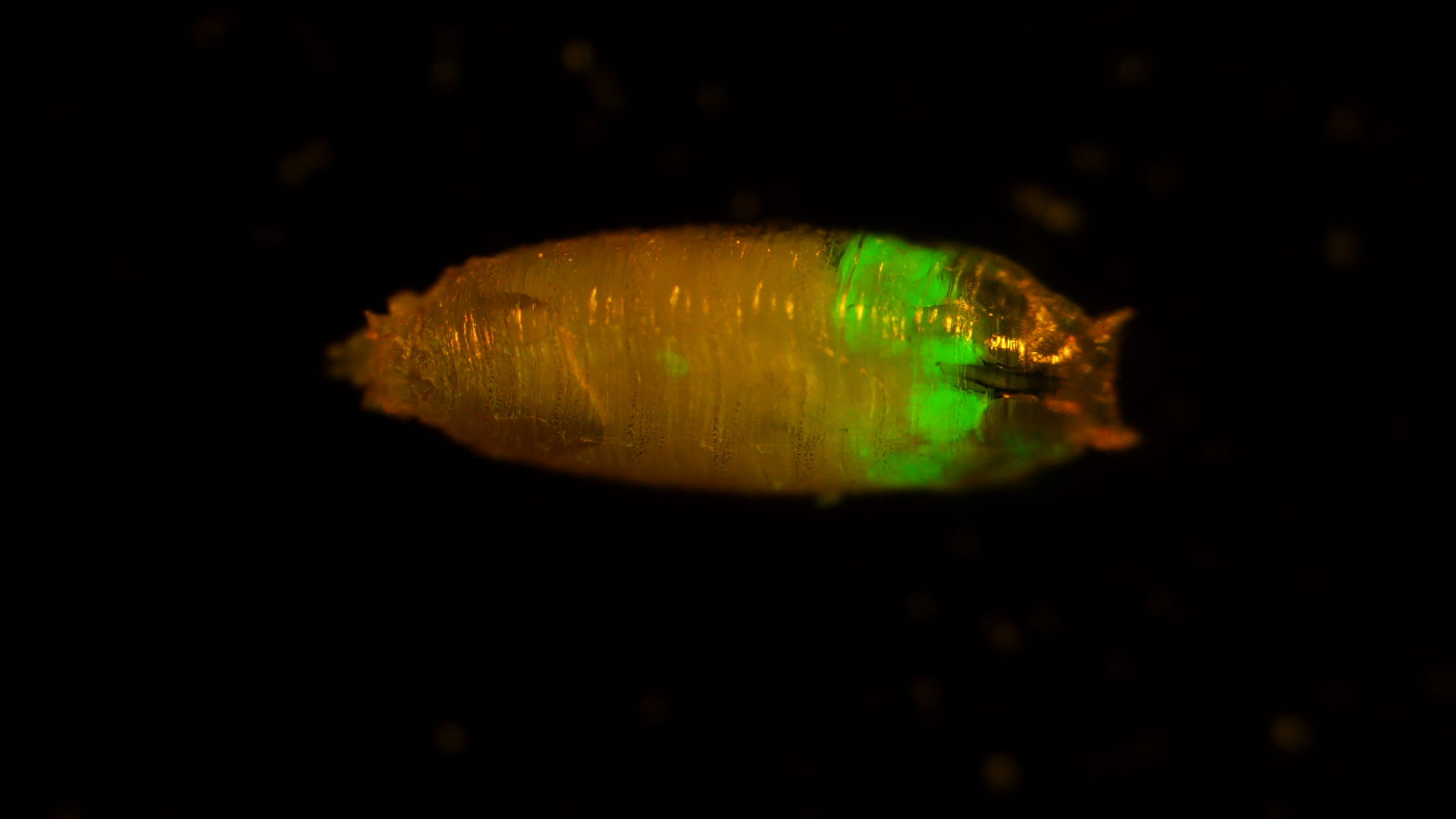

Supplement: Supplementary file 7 — Source data Fig. 3 [file 44318_2025_489_MOESM7_ESM.zip › Figure 3D/13 original image.tif]

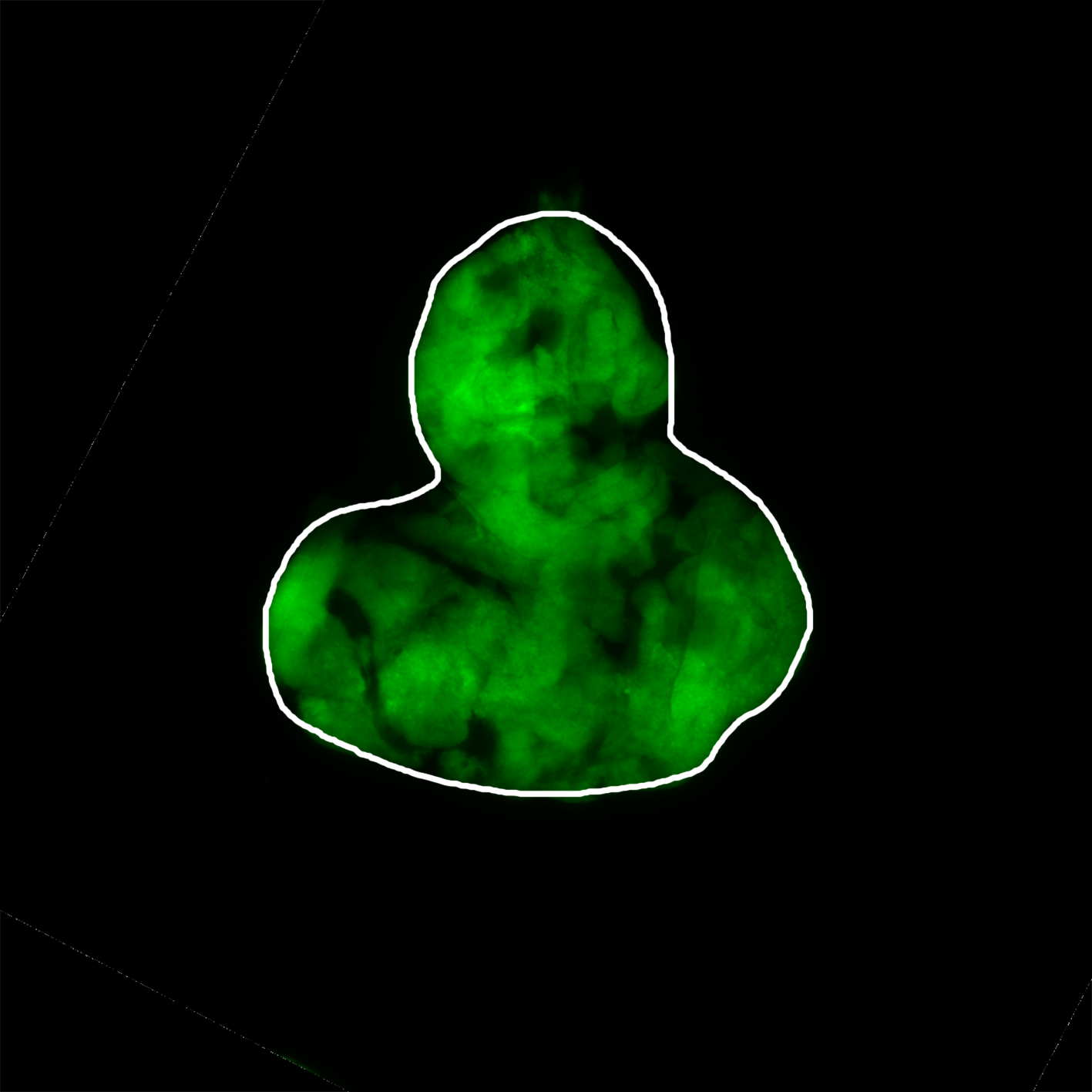

Supplement: Supplementary file 7 — Source data Fig. 3 [file 44318_2025_489_MOESM7_ESM.zip › Figure 3D/14-1 rotated and cut image with border line.tif]

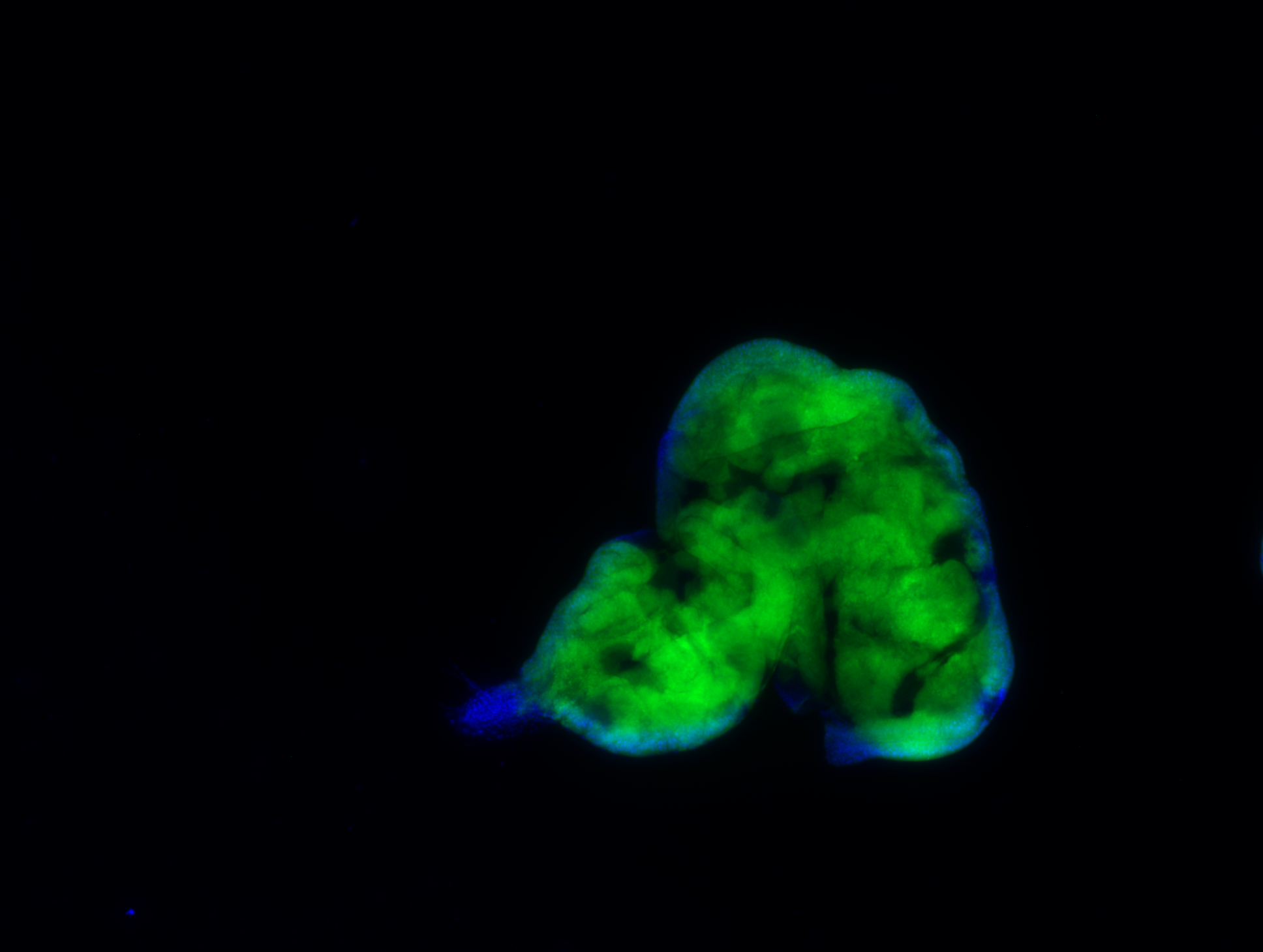

Supplement: Supplementary file 7 — Source data Fig. 3 [file 44318_2025_489_MOESM7_ESM.zip › Figure 3D/14-2 original image.tif]

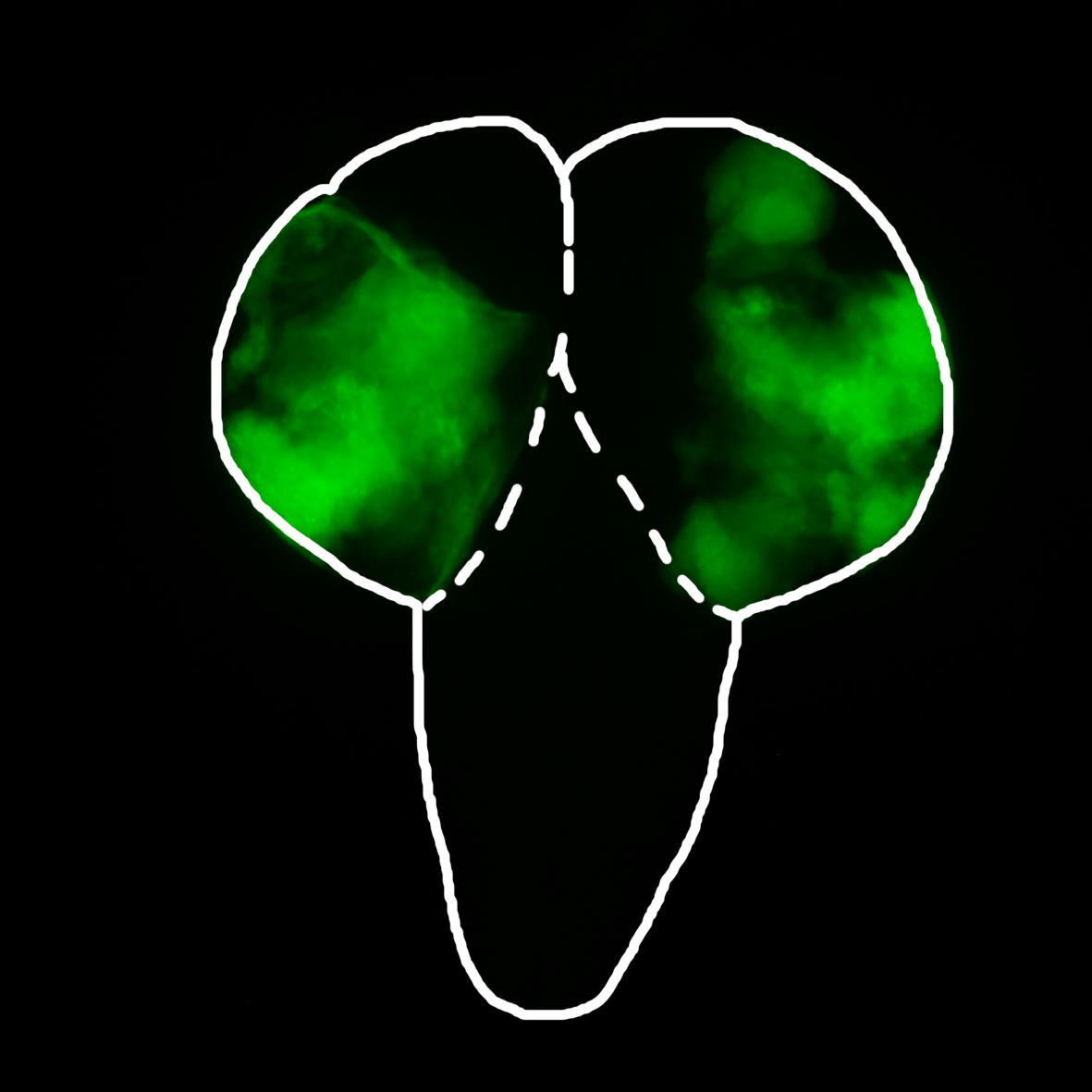

Supplement: Supplementary file 7 — Source data Fig. 3 [file 44318_2025_489_MOESM7_ESM.zip › Figure 3D/15-1 rotated and cut image with border line.tif]

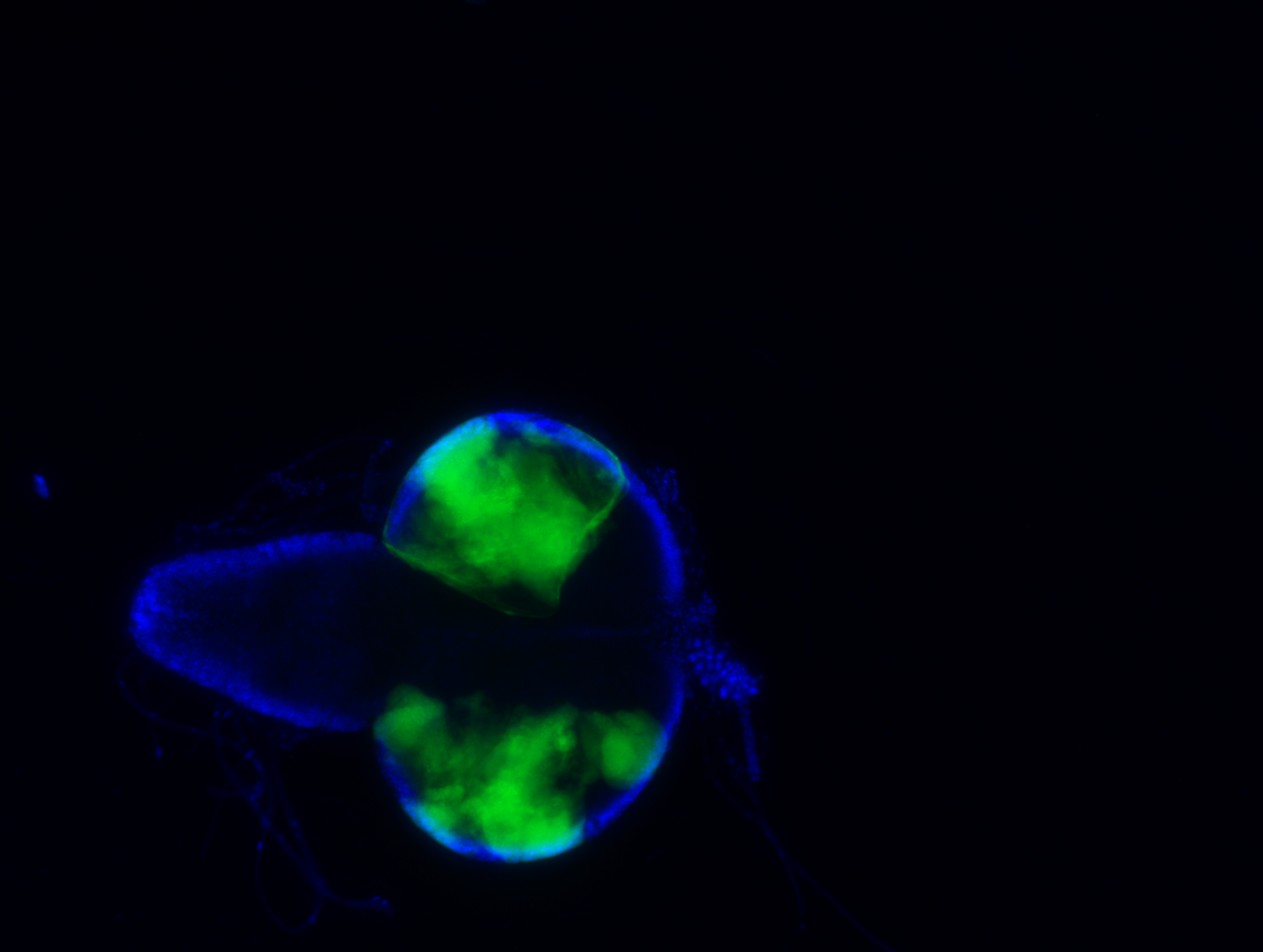

Supplement: Supplementary file 7 — Source data Fig. 3 [file 44318_2025_489_MOESM7_ESM.zip › Figure 3D/15-2 original image.tif]

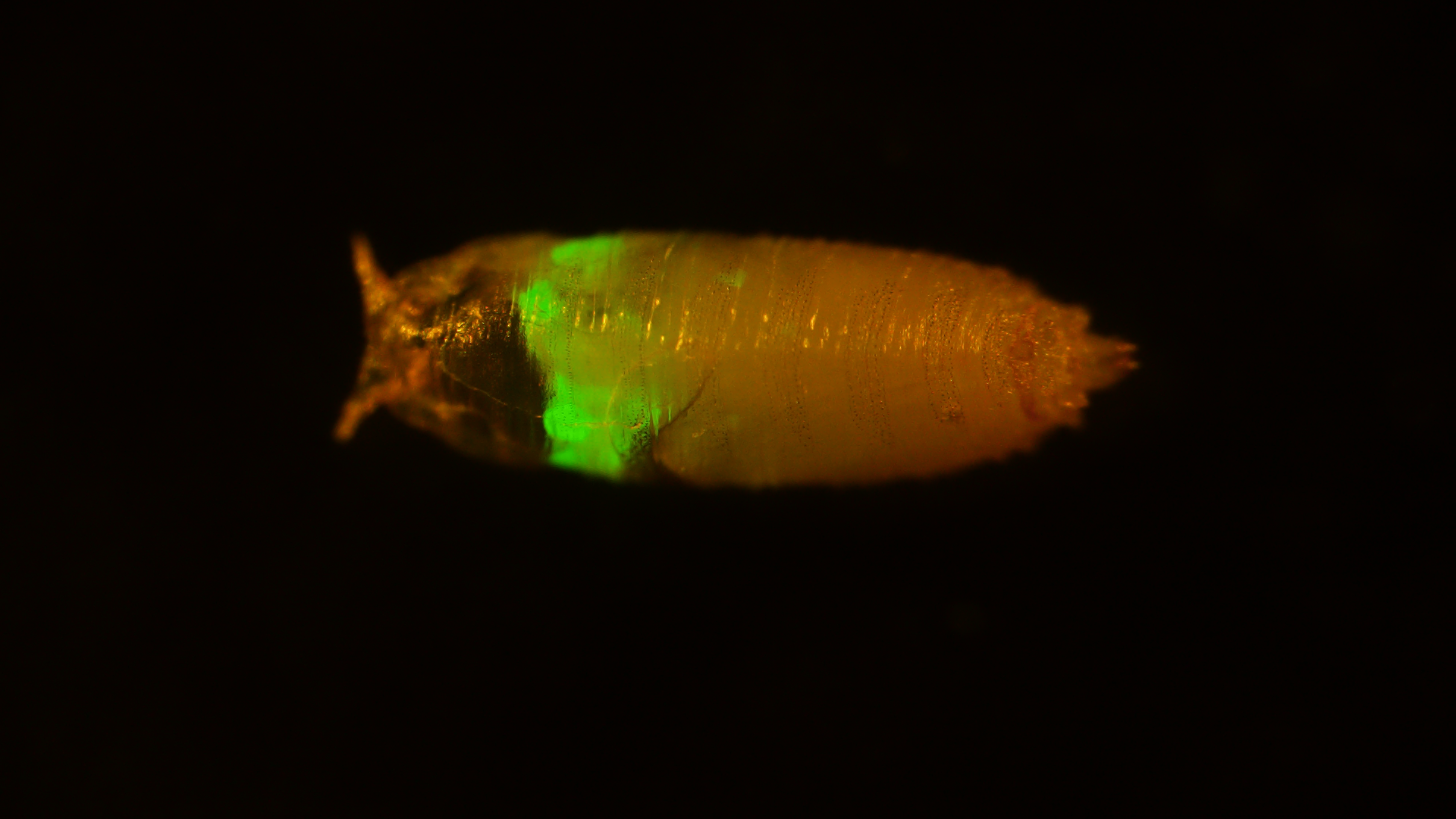

Supplement: Supplementary file 7 — Source data Fig. 3 [file 44318_2025_489_MOESM7_ESM.zip › Figure 3D/16 original image.tif]

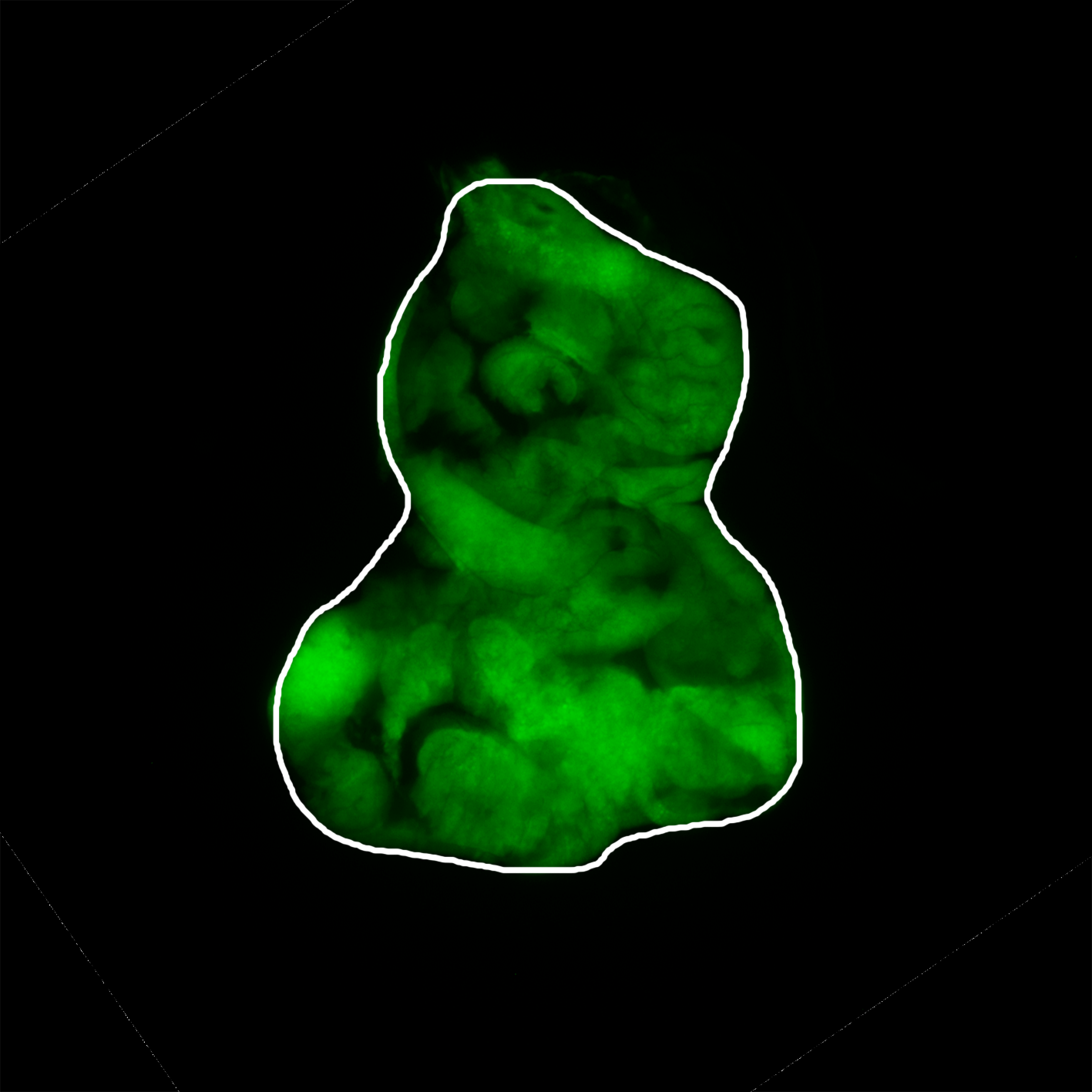

Supplement: Supplementary file 7 — Source data Fig. 3 [file 44318_2025_489_MOESM7_ESM.zip › Figure 3D/17-1 rotated and cut image with border line.tif]

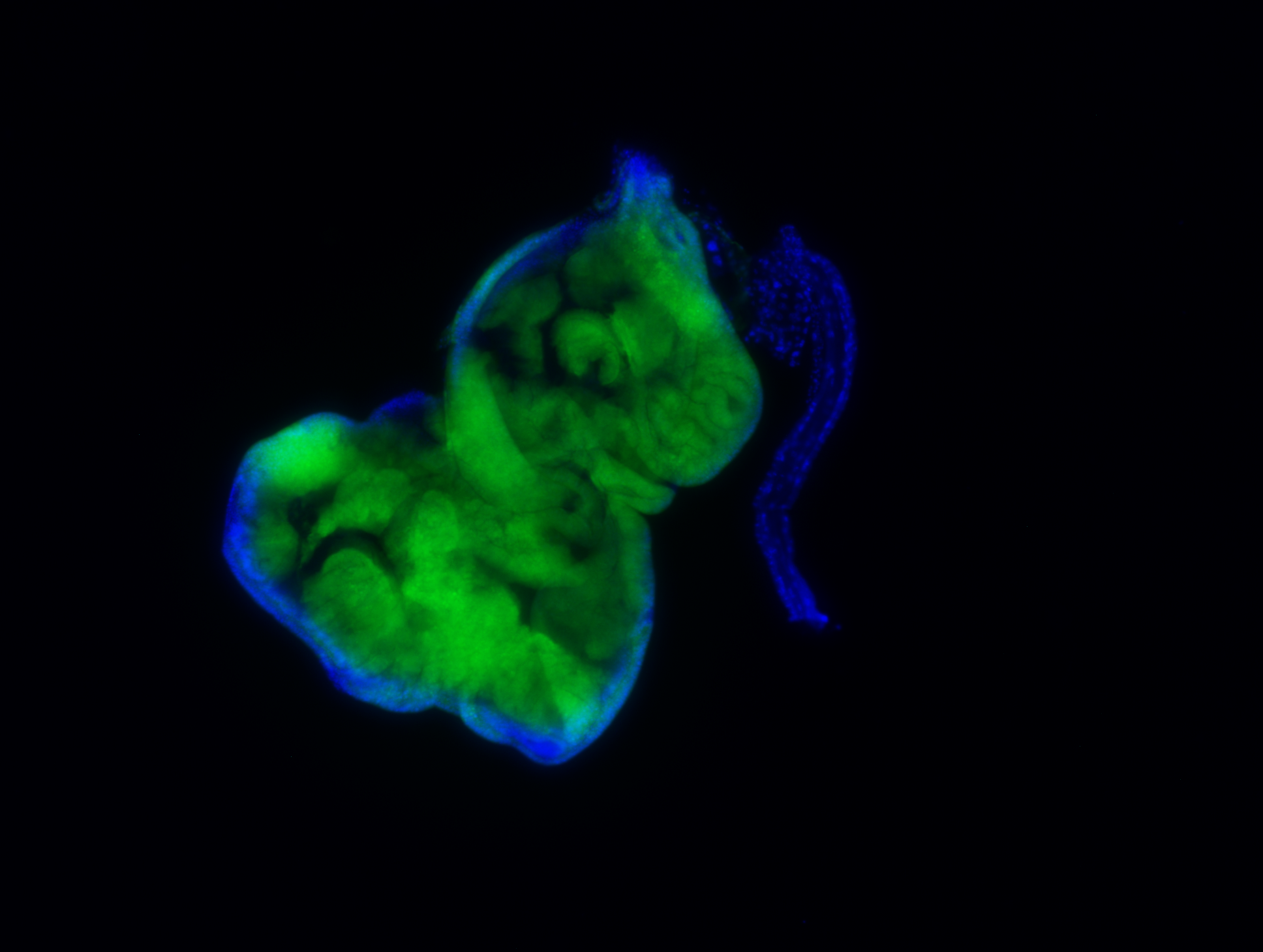

Supplement: Supplementary file 7 — Source data Fig. 3 [file 44318_2025_489_MOESM7_ESM.zip › Figure 3D/17-2 original image.tif]

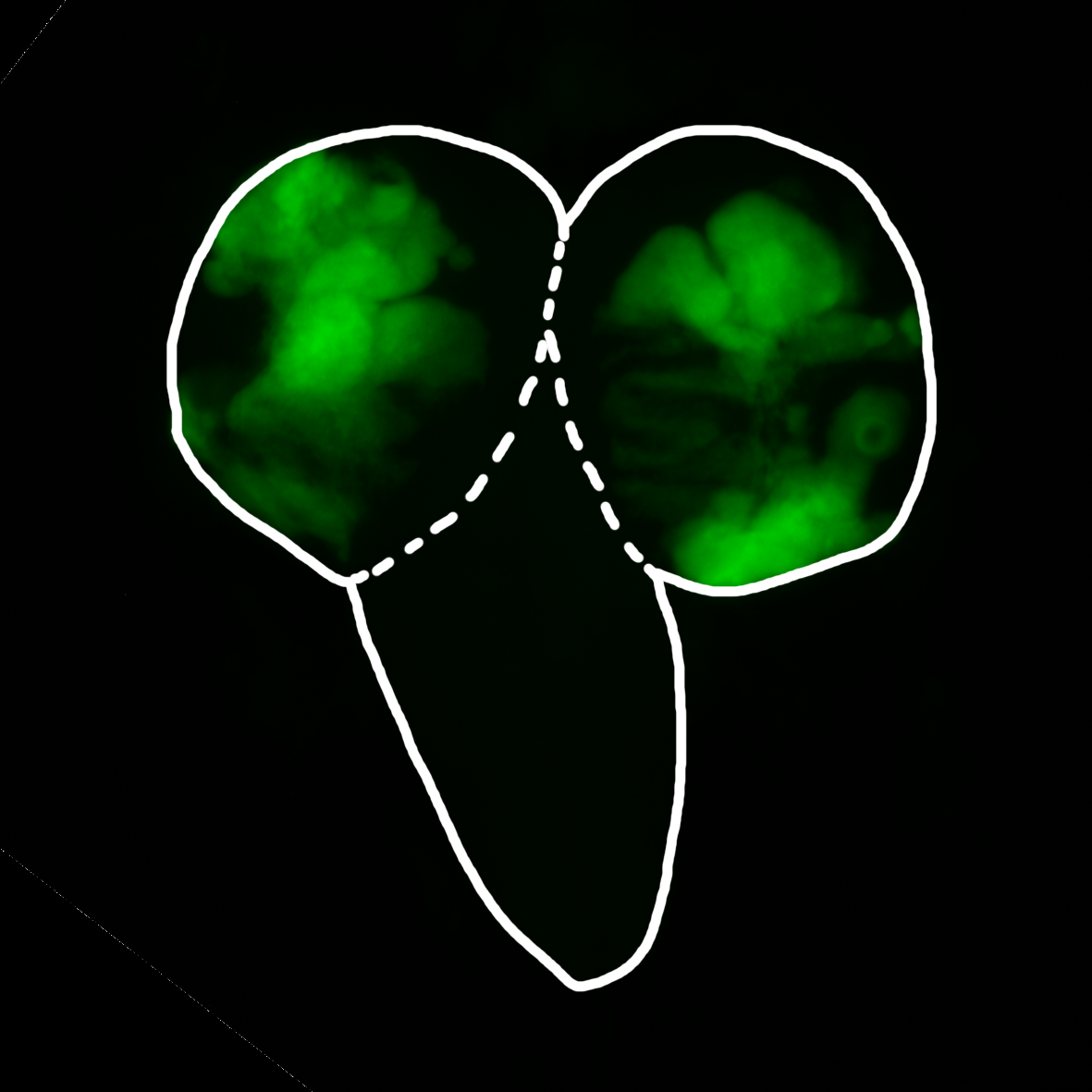

Supplement: Supplementary file 7 — Source data Fig. 3 [file 44318_2025_489_MOESM7_ESM.zip › Figure 3D/18-1 rotated and cut image with border line.tif]

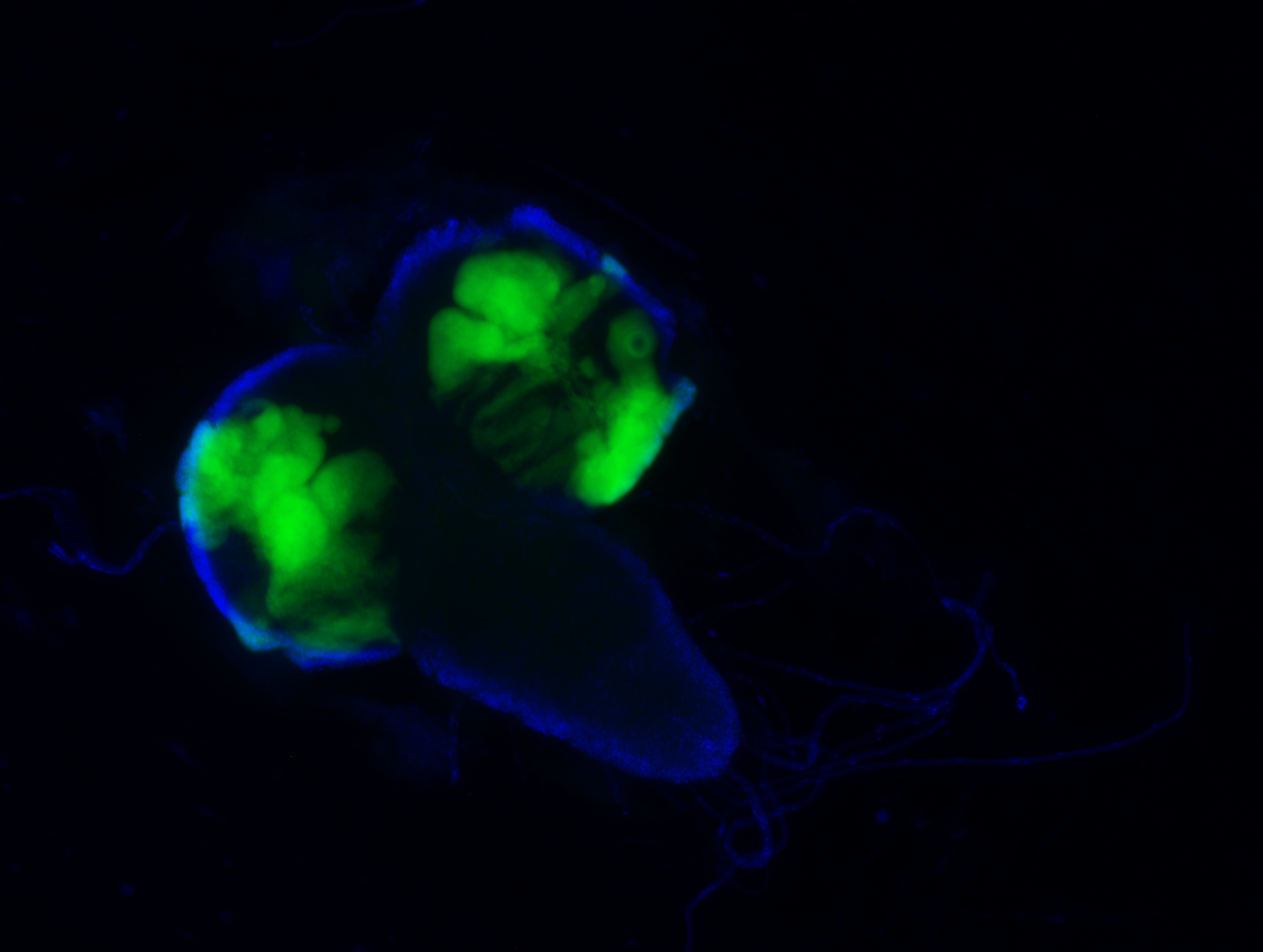

Supplement: Supplementary file 7 — Source data Fig. 3 [file 44318_2025_489_MOESM7_ESM.zip › Figure 3D/18-2 original image.tif]

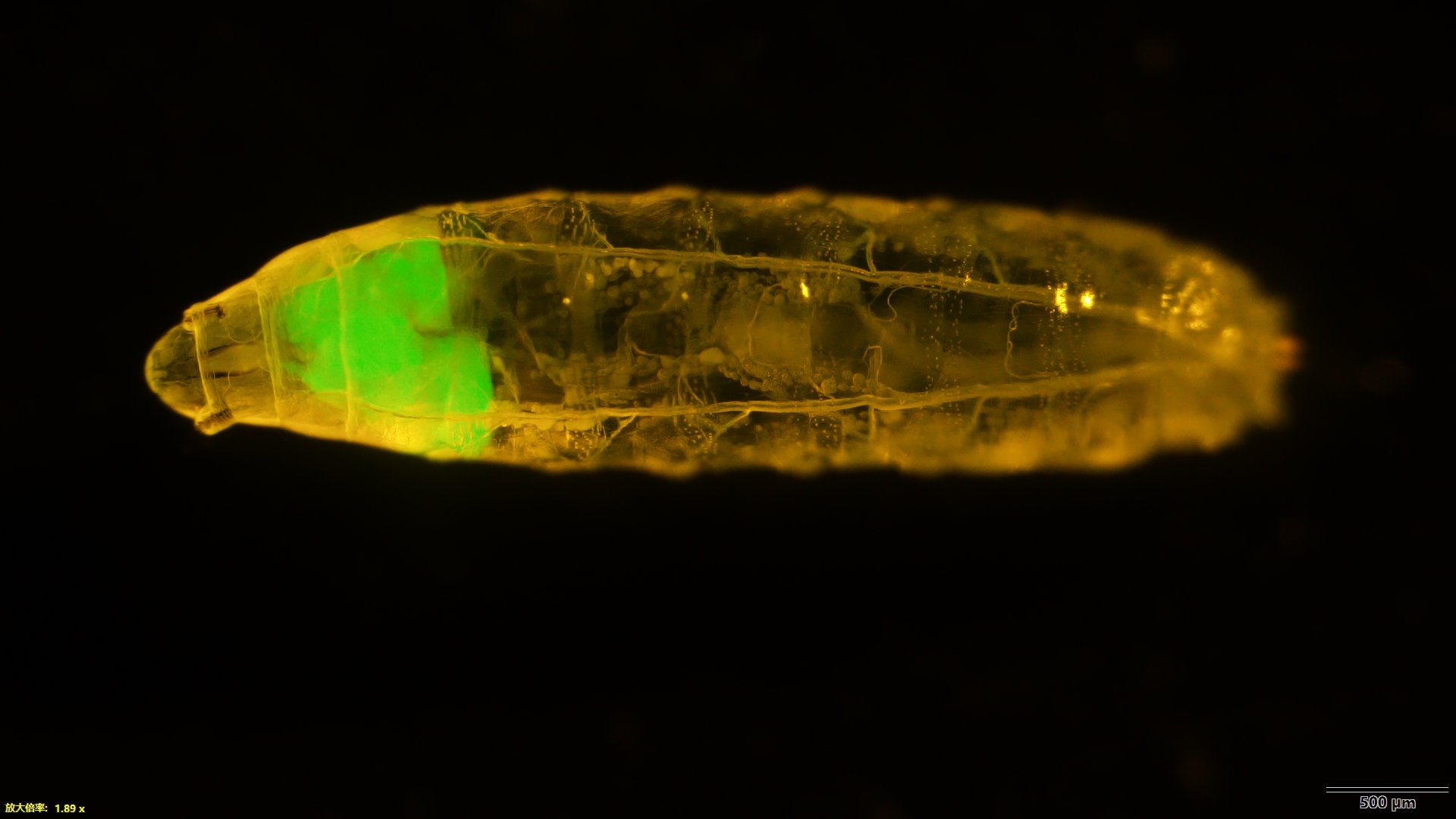

Supplement: Supplementary file 7 — Source data Fig. 3 [file 44318_2025_489_MOESM7_ESM.zip › Figure 3D/19 original image.jpg]

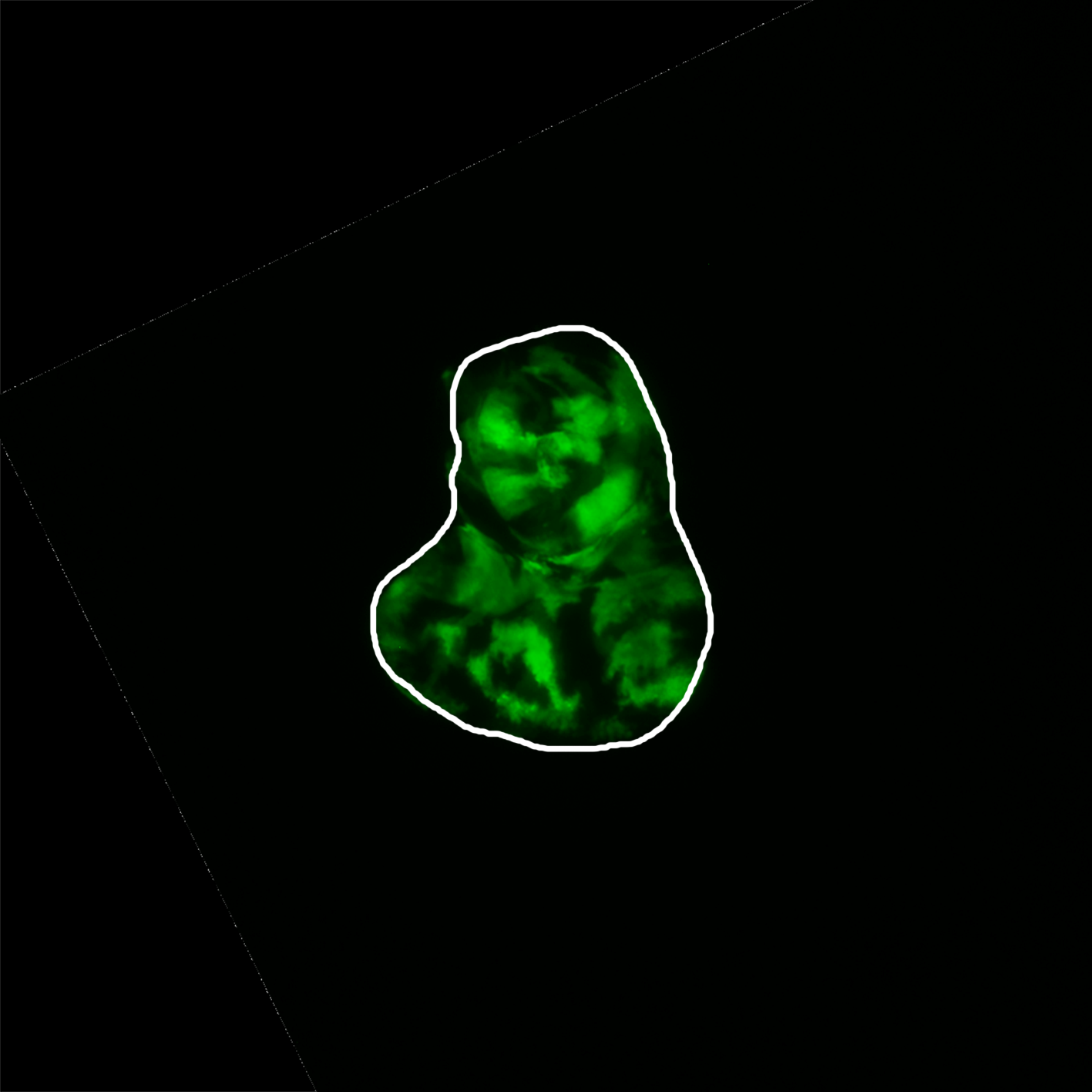

Supplement: Supplementary file 7 — Source data Fig. 3 [file 44318_2025_489_MOESM7_ESM.zip › Figure 3D/2-1 rotated and cut image with border line.tif]

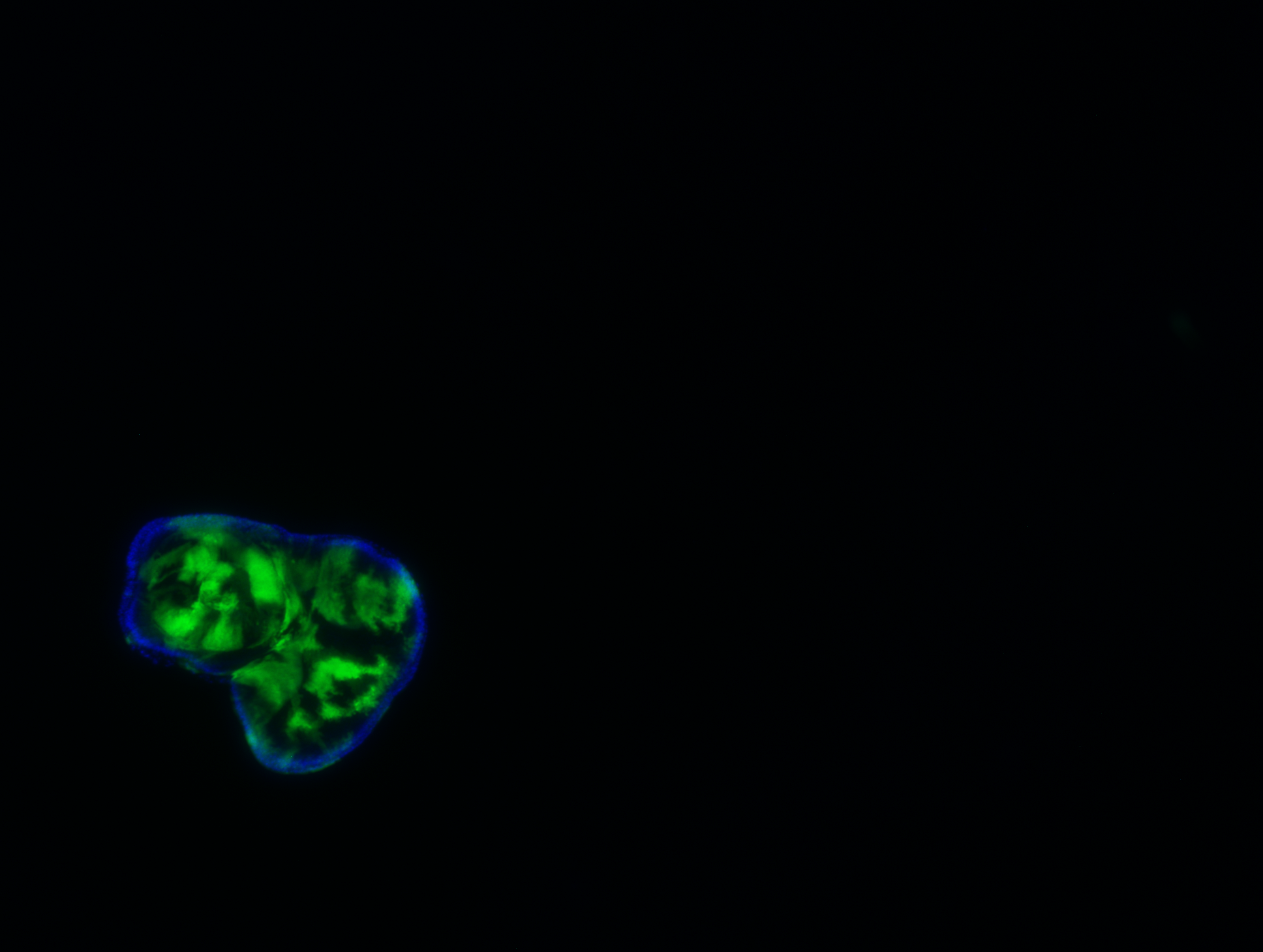

Supplement: Supplementary file 7 — Source data Fig. 3 [file 44318_2025_489_MOESM7_ESM.zip › Figure 3D/2-2 original image.tif]

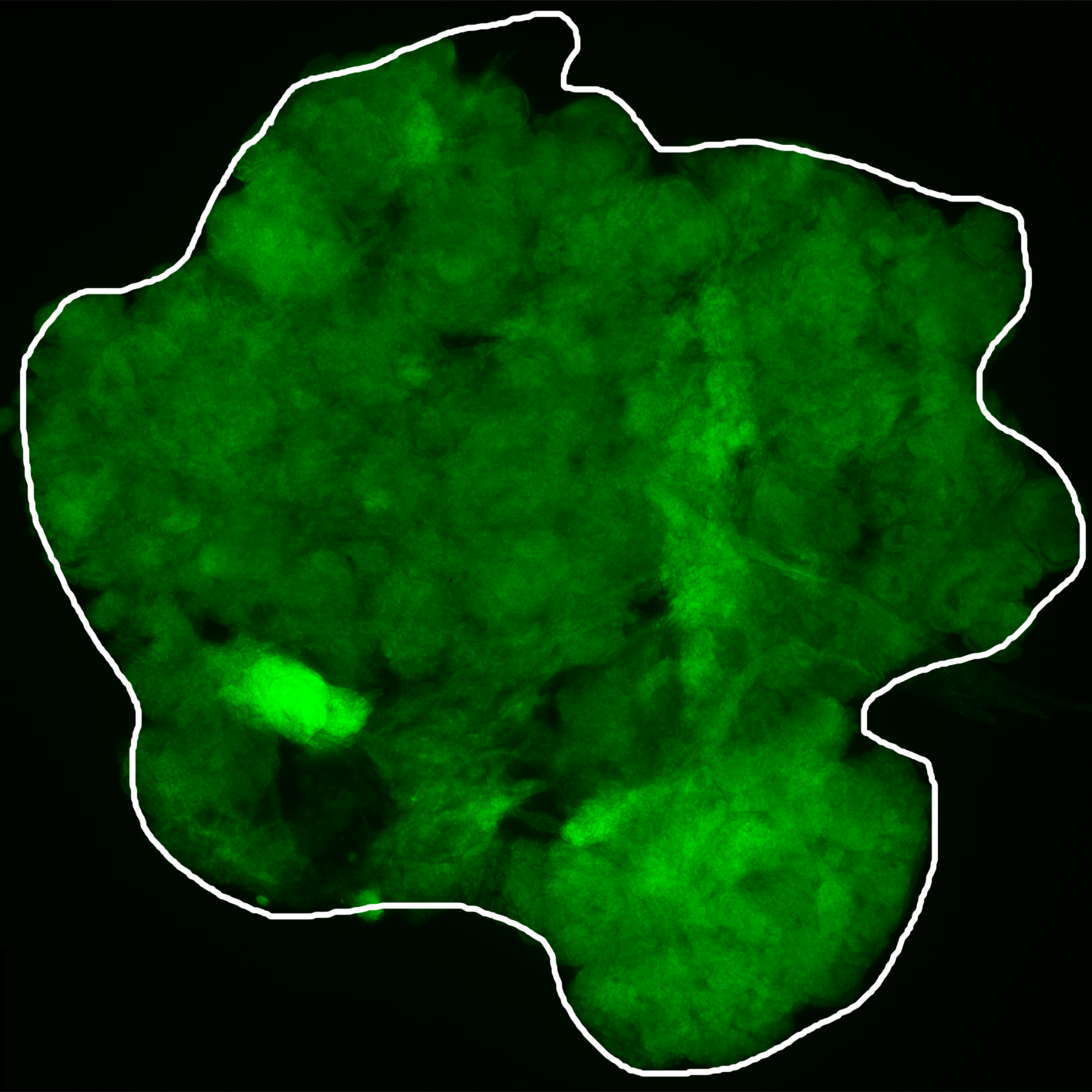

Supplement: Supplementary file 7 — Source data Fig. 3 [file 44318_2025_489_MOESM7_ESM.zip › Figure 3D/20-1 rotated and cut image with border line.tif]

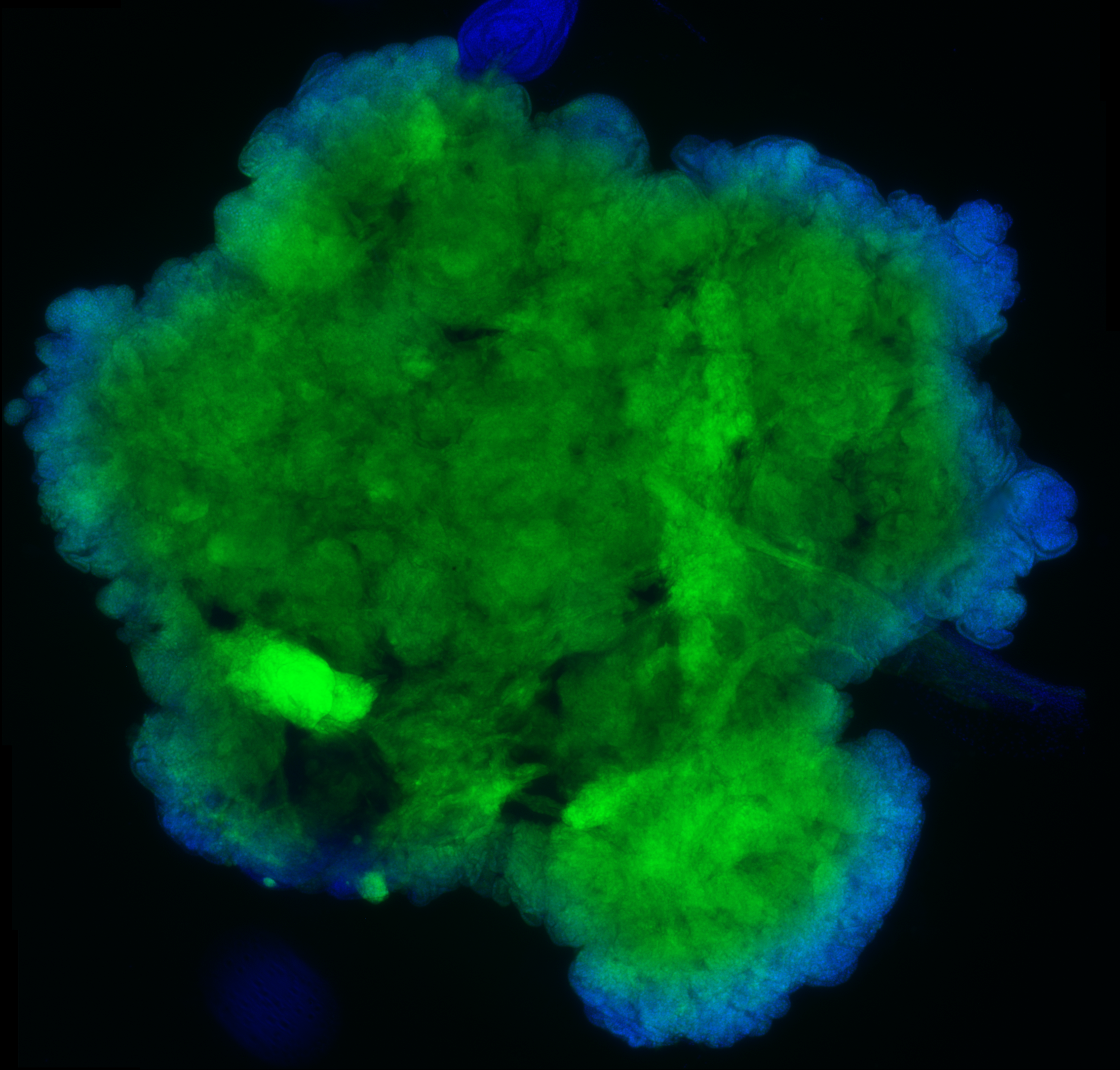

Supplement: Supplementary file 7 — Source data Fig. 3 [file 44318_2025_489_MOESM7_ESM.zip › Figure 3D/20-2 original image.tif]

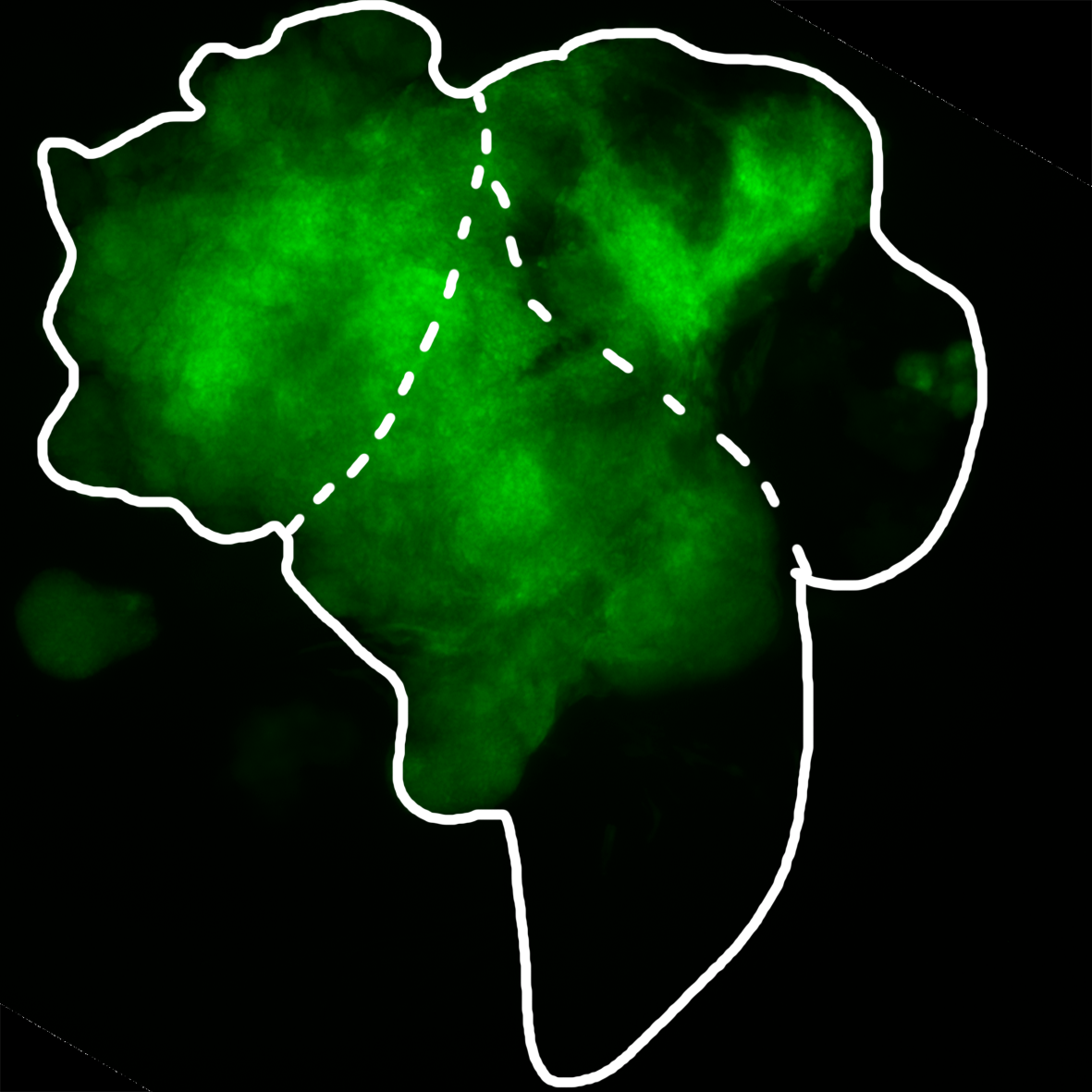

Supplement: Supplementary file 7 — Source data Fig. 3 [file 44318_2025_489_MOESM7_ESM.zip › Figure 3D/21-1 rotated and cut image with border line.tif]

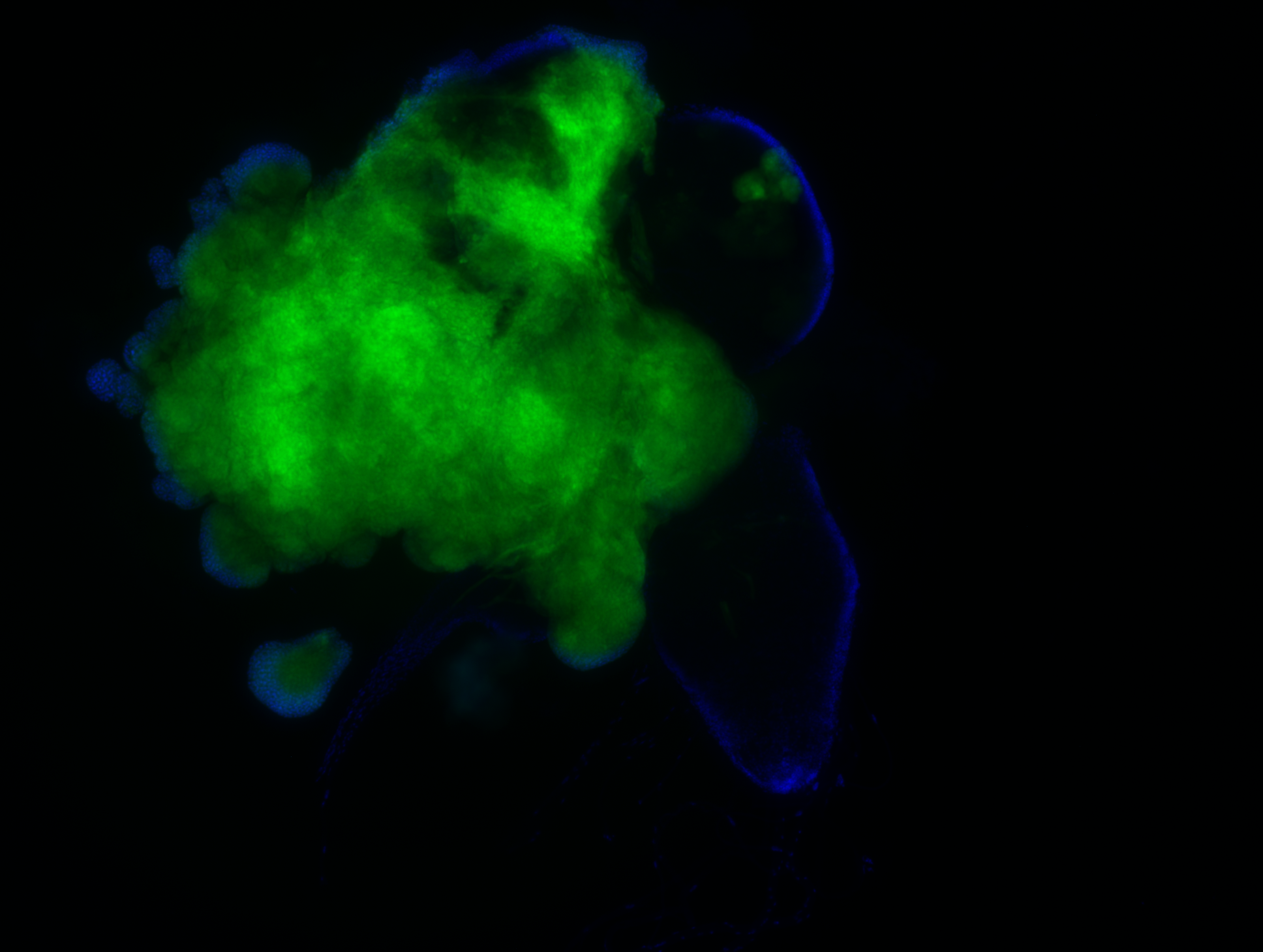

Supplement: Supplementary file 7 — Source data Fig. 3 [file 44318_2025_489_MOESM7_ESM.zip › Figure 3D/21-2 original image.tif]

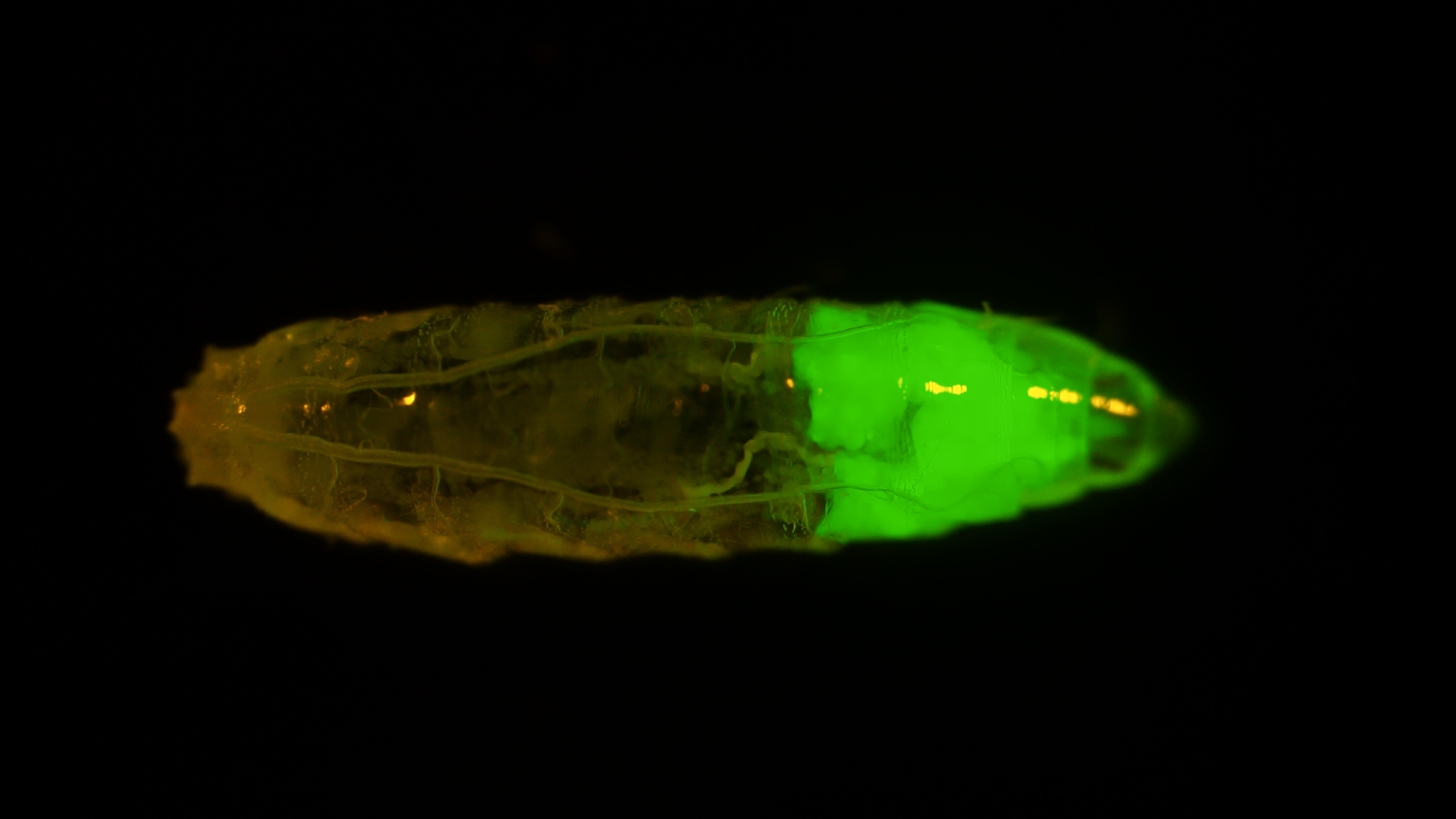

Supplement: Supplementary file 7 — Source data Fig. 3 [file 44318_2025_489_MOESM7_ESM.zip › Figure 3D/22 original image.tif]

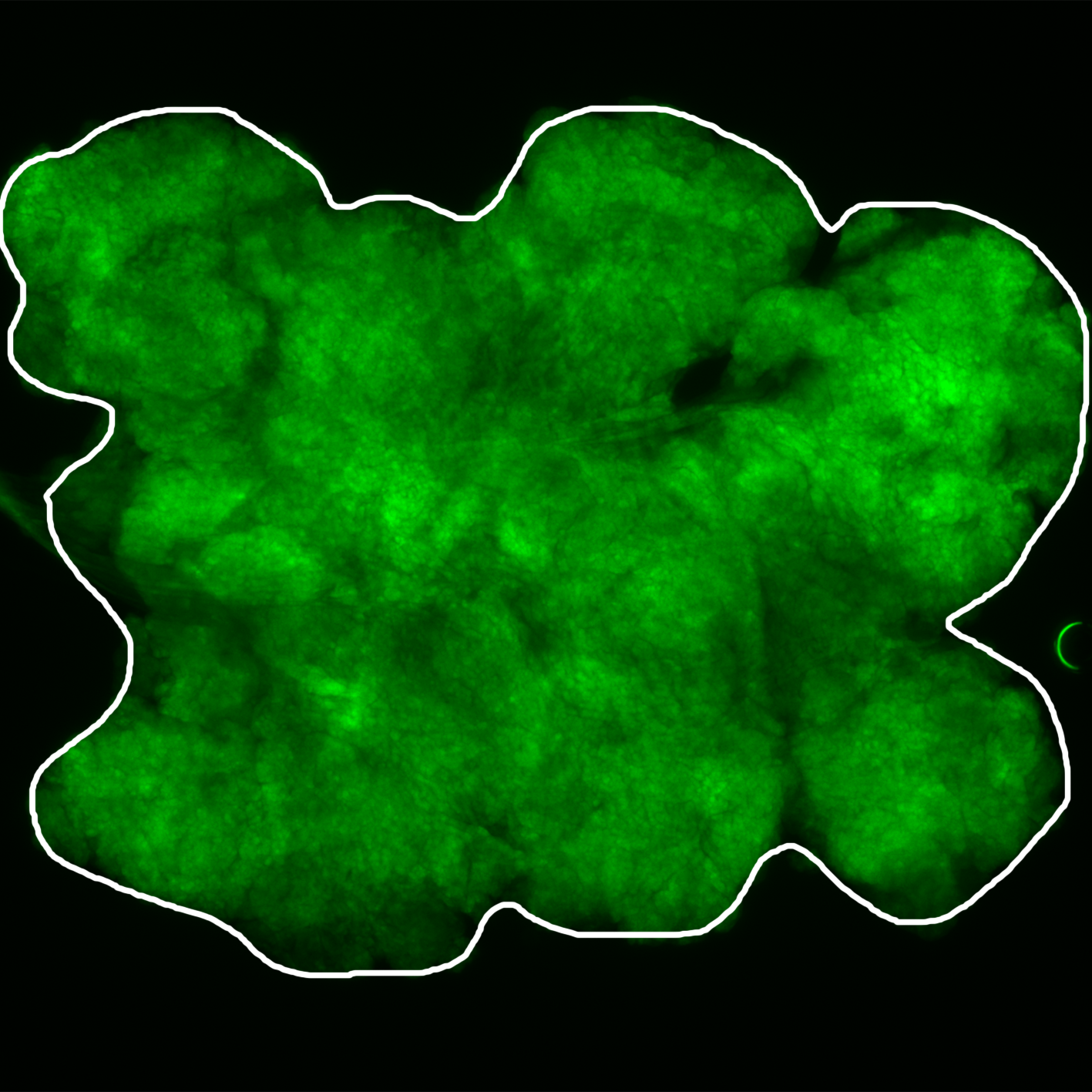

Supplement: Supplementary file 7 — Source data Fig. 3 [file 44318_2025_489_MOESM7_ESM.zip › Figure 3D/23-1 rotated and cut image with border line.tif]

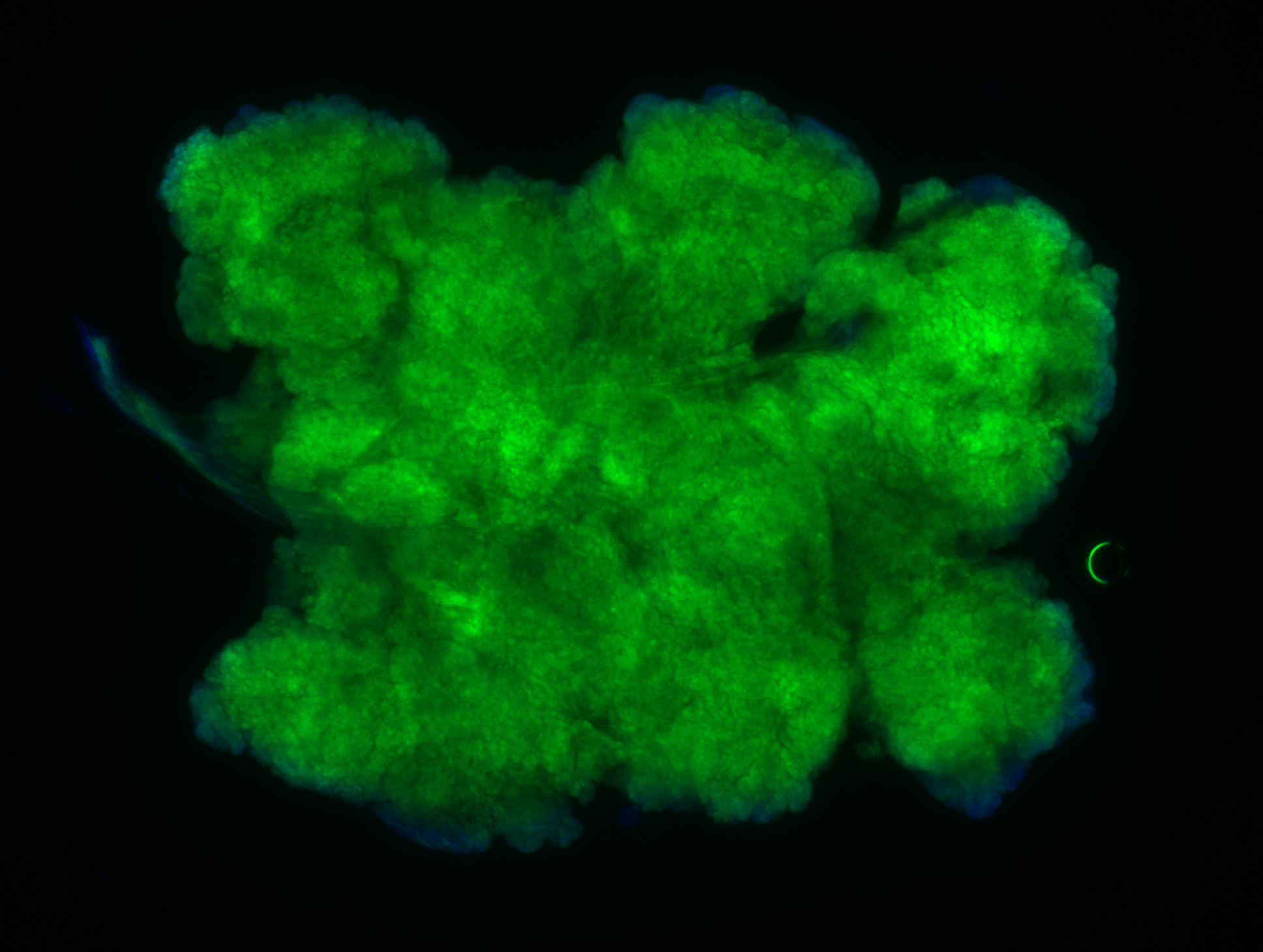

Supplement: Supplementary file 7 — Source data Fig. 3 [file 44318_2025_489_MOESM7_ESM.zip › Figure 3D/23-2 original image.tif]

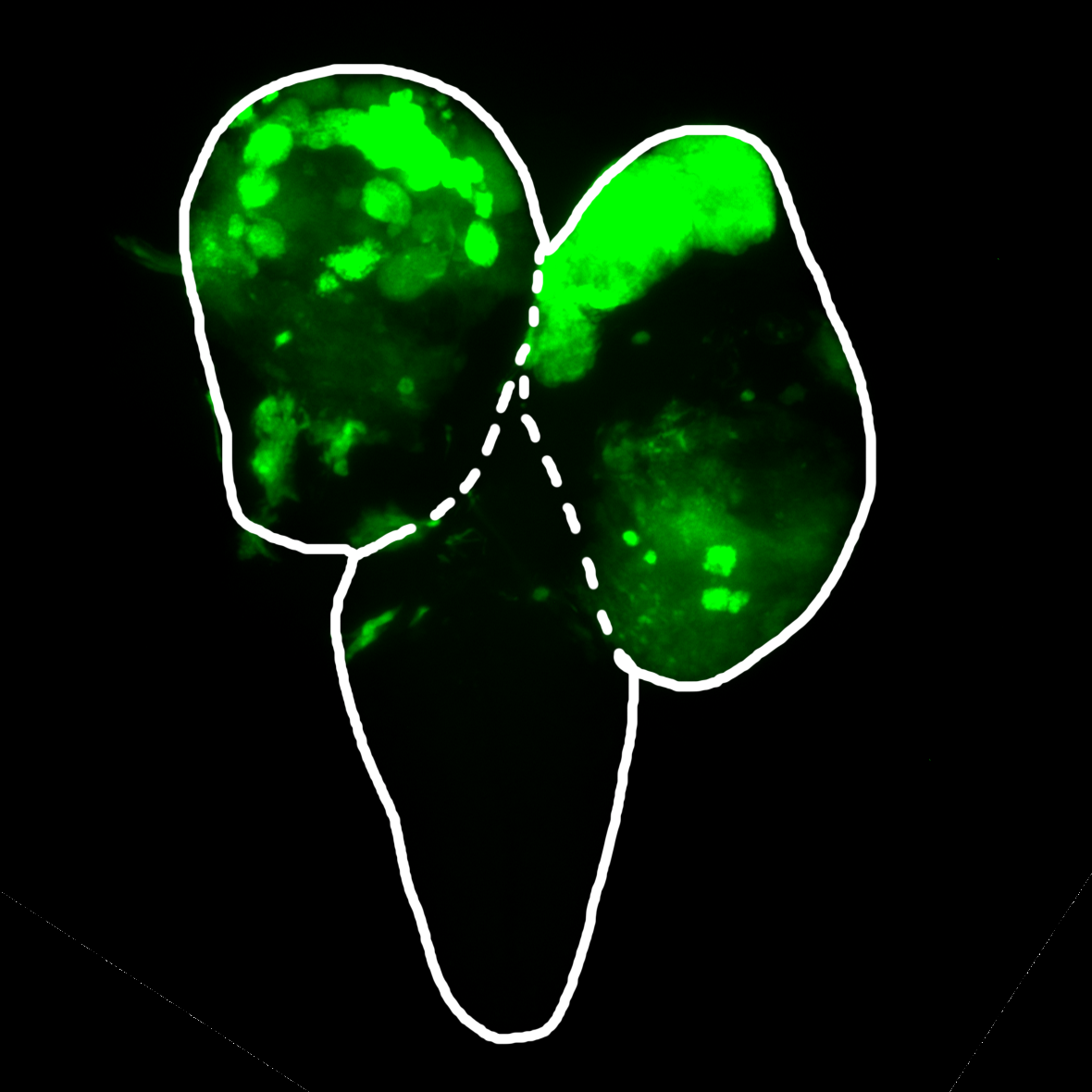

Supplement: Supplementary file 7 — Source data Fig. 3 [file 44318_2025_489_MOESM7_ESM.zip › Figure 3D/24-1 rotated and cut image with border line.tif]

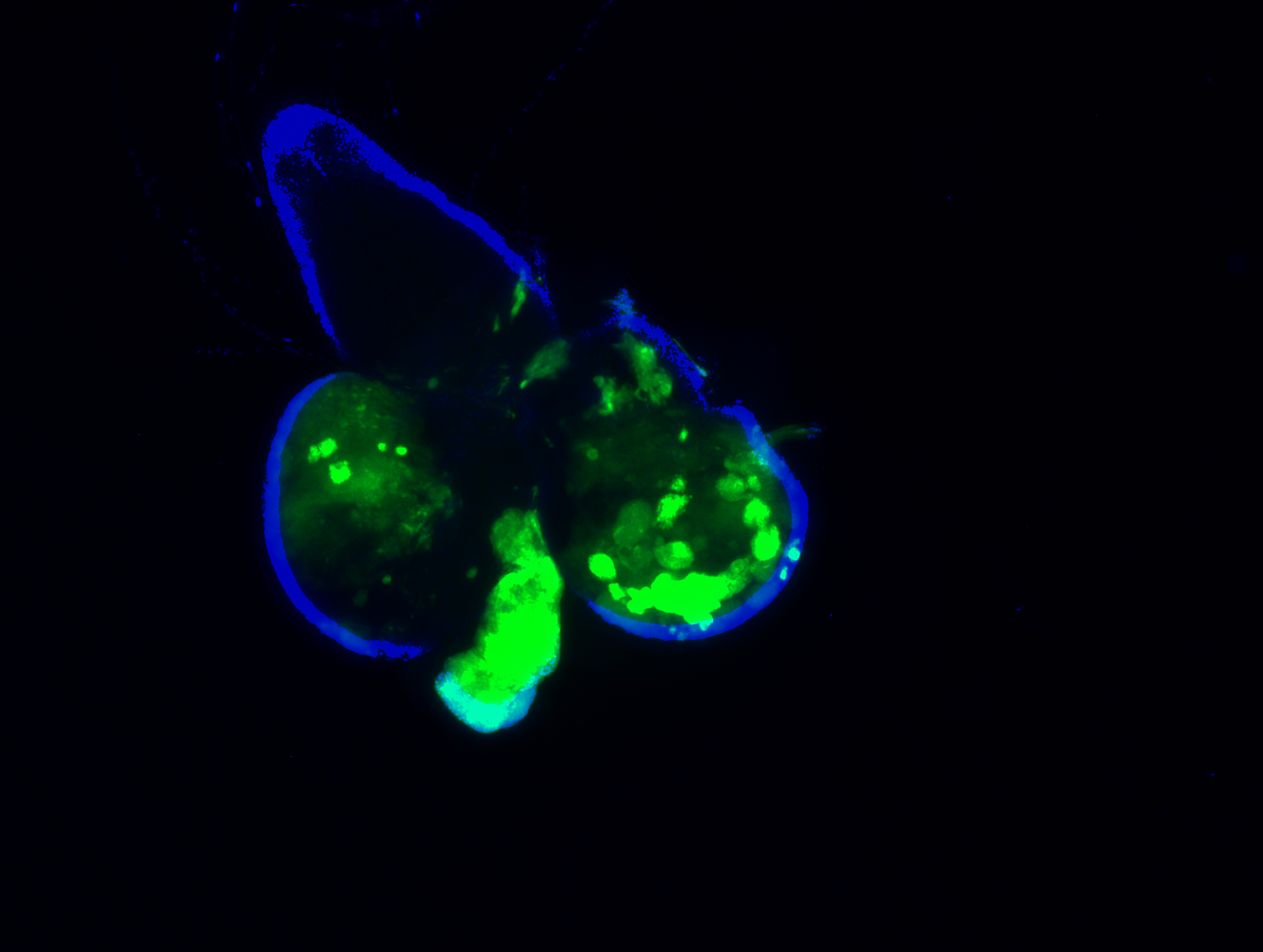

Supplement: Supplementary file 7 — Source data Fig. 3 [file 44318_2025_489_MOESM7_ESM.zip › Figure 3D/24-2 original image.tif]

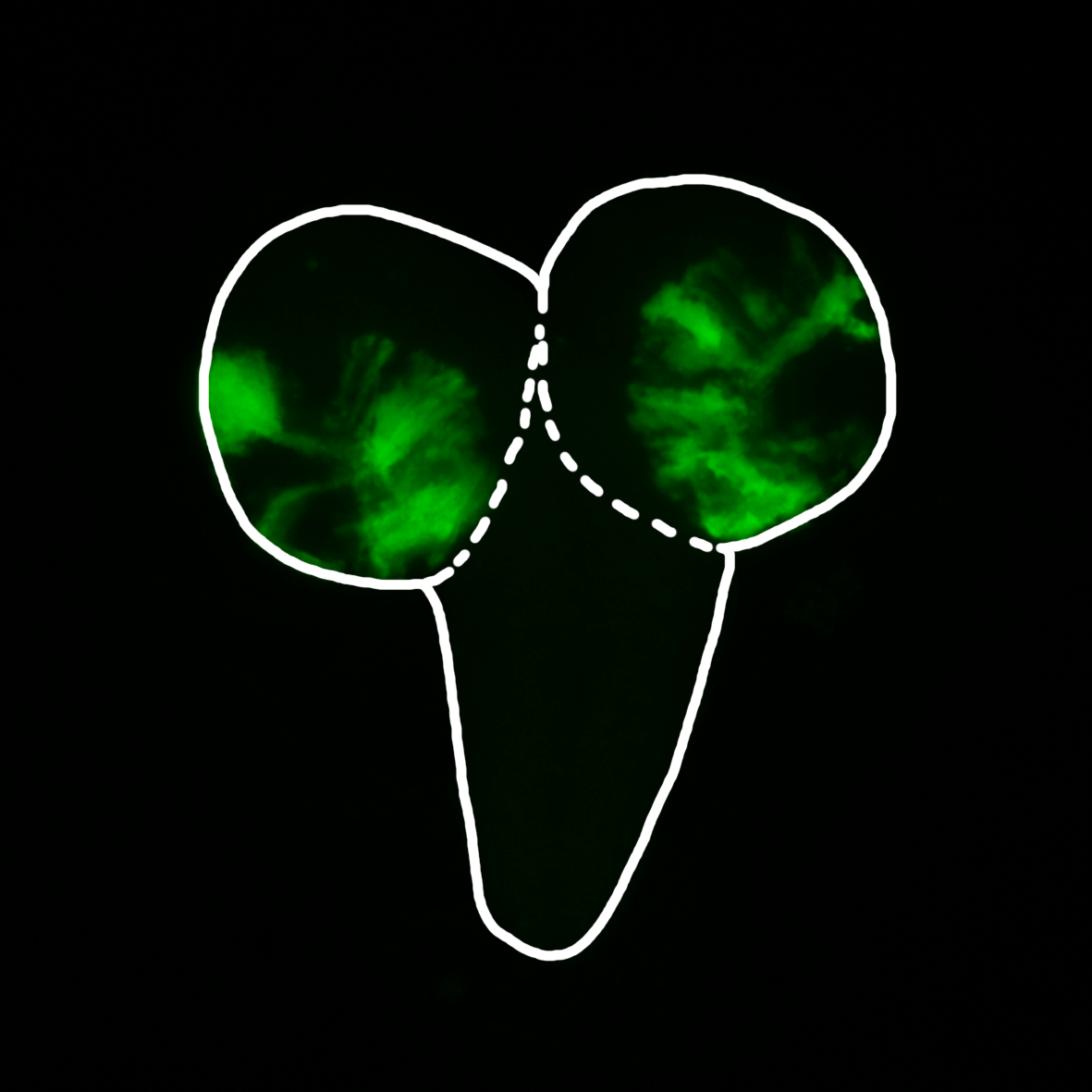

Supplement: Supplementary file 7 — Source data Fig. 3 [file 44318_2025_489_MOESM7_ESM.zip › Figure 3D/3-1 rotated and cut image with border line.tif]

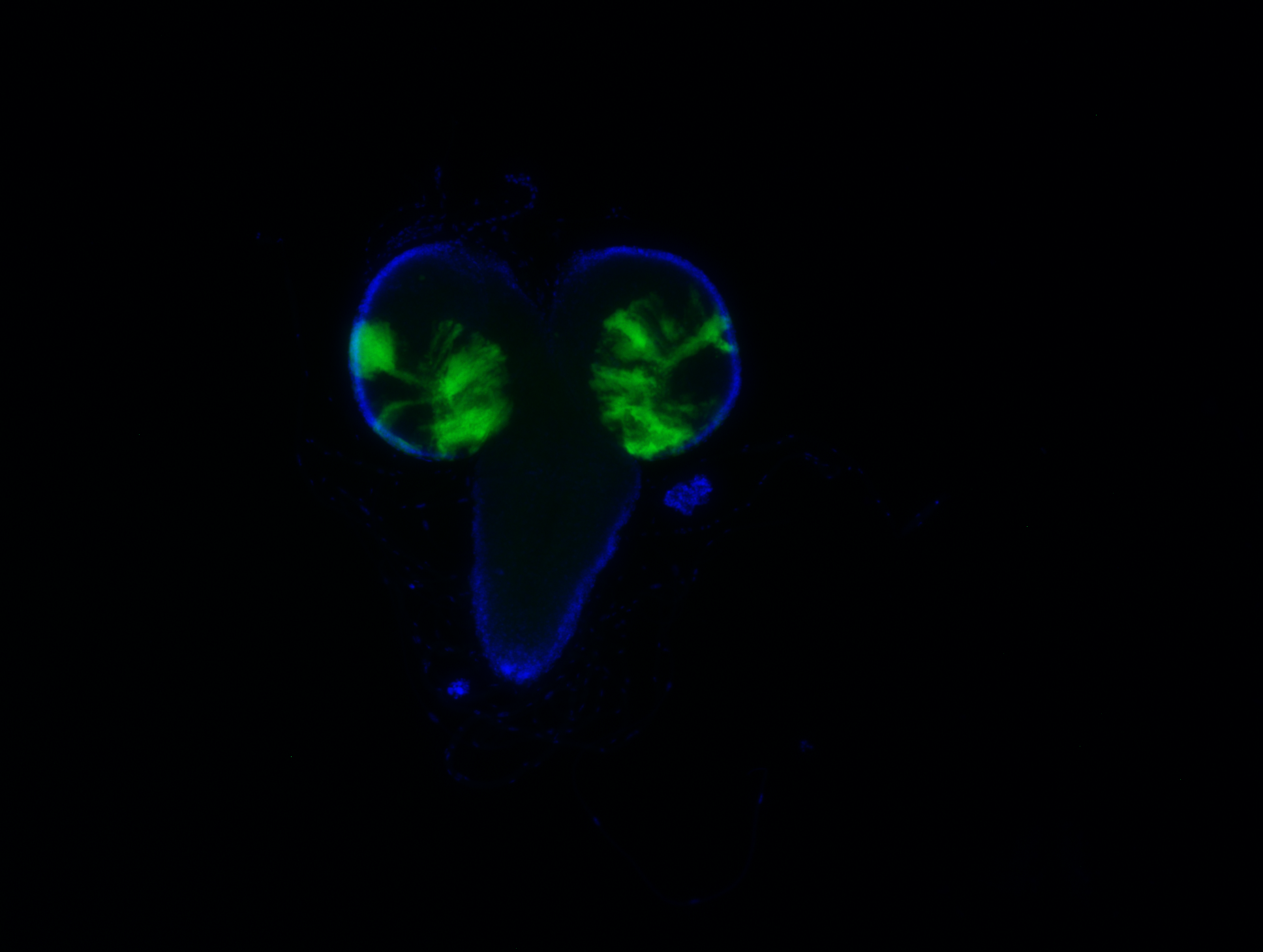

Supplement: Supplementary file 7 — Source data Fig. 3 [file 44318_2025_489_MOESM7_ESM.zip › Figure 3D/3-2 original image.tif]

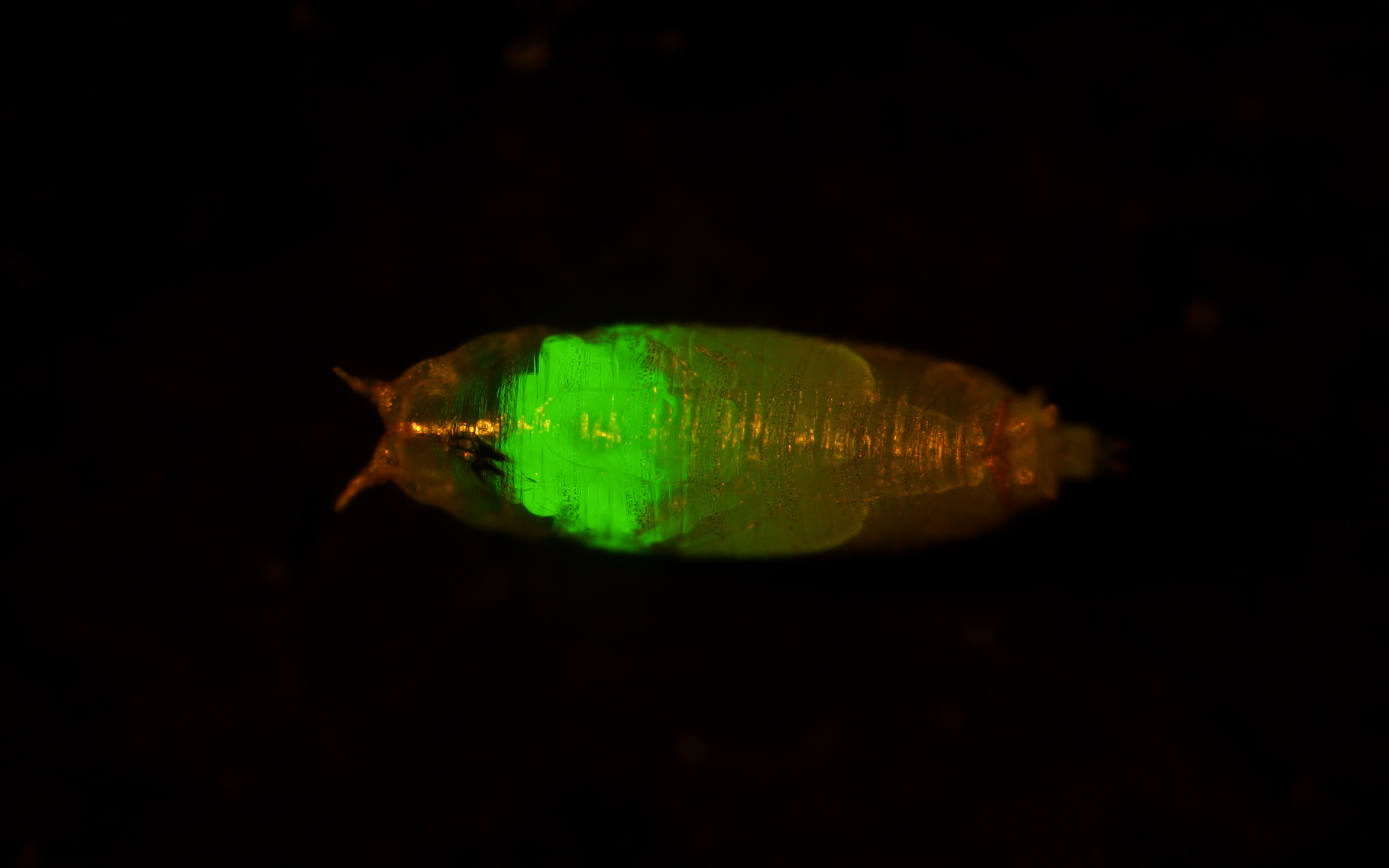

Supplement: Supplementary file 7 — Source data Fig. 3 [file 44318_2025_489_MOESM7_ESM.zip › Figure 3D/4 original image.tif]

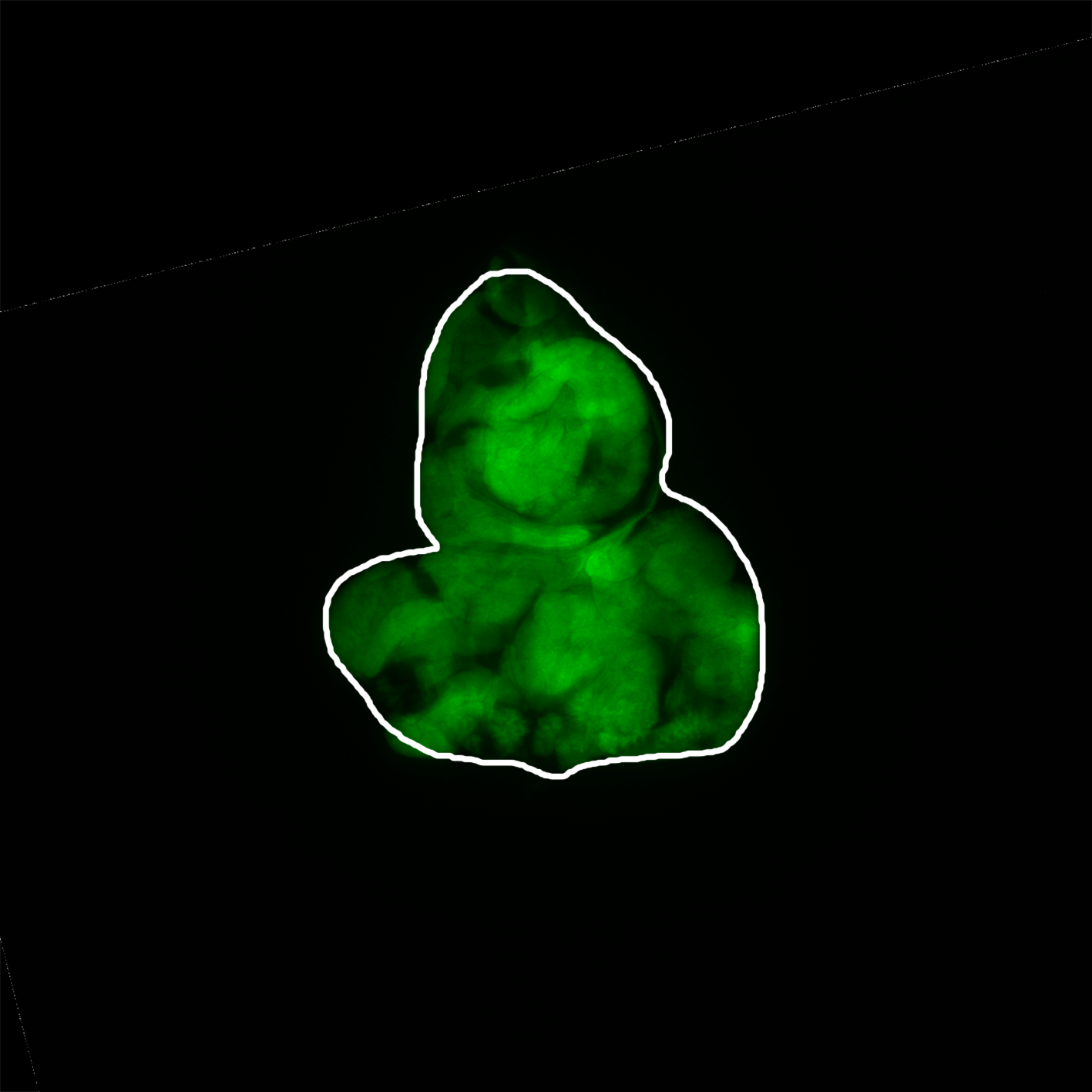

Supplement: Supplementary file 7 — Source data Fig. 3 [file 44318_2025_489_MOESM7_ESM.zip › Figure 3D/5-1 rotated and cut image with border line.tif]

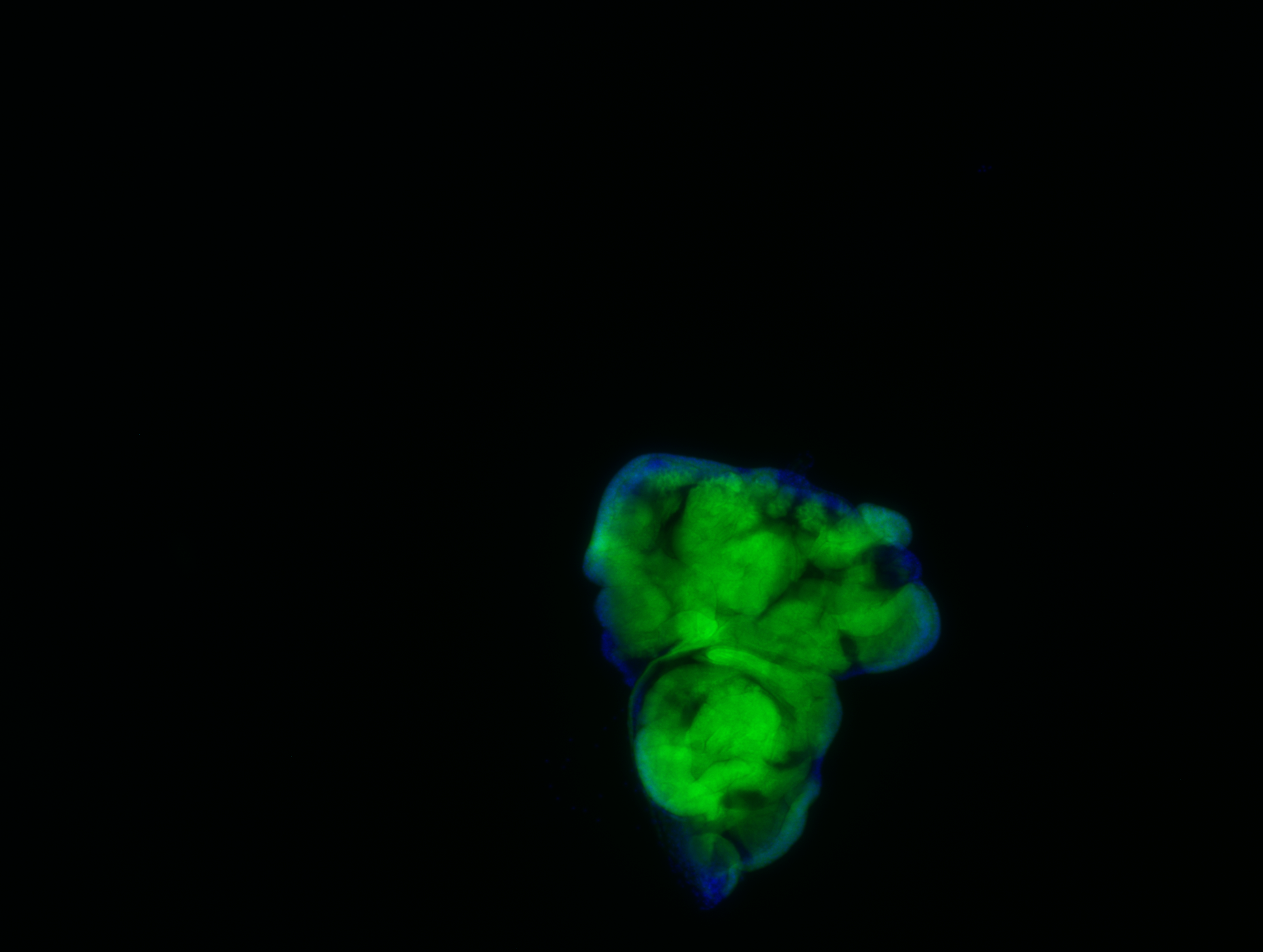

Supplement: Supplementary file 7 — Source data Fig. 3 [file 44318_2025_489_MOESM7_ESM.zip › Figure 3D/5-2 original image.tif]

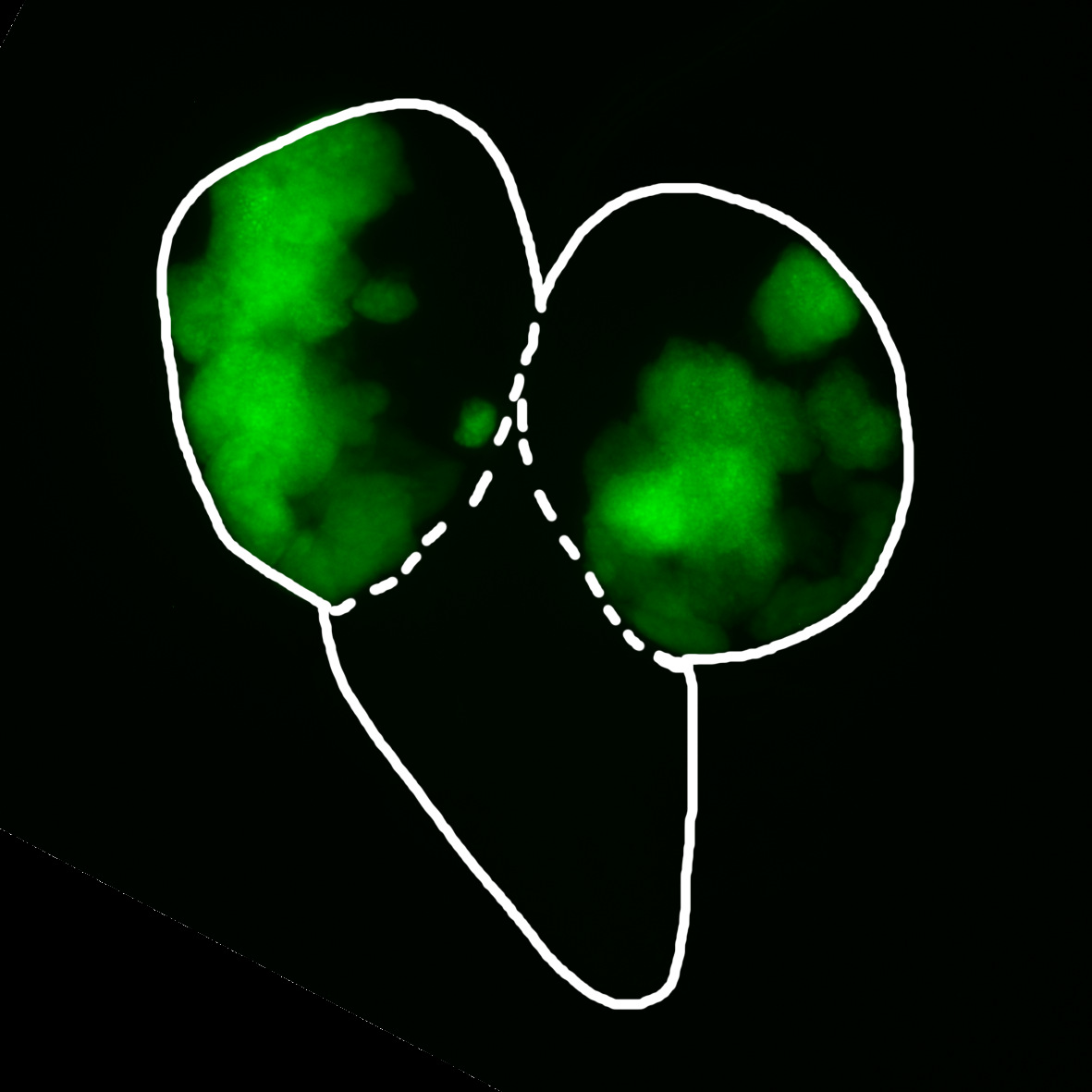

Supplement: Supplementary file 7 — Source data Fig. 3 [file 44318_2025_489_MOESM7_ESM.zip › Figure 3D/6-1 rotated and cut image with border line.tif]

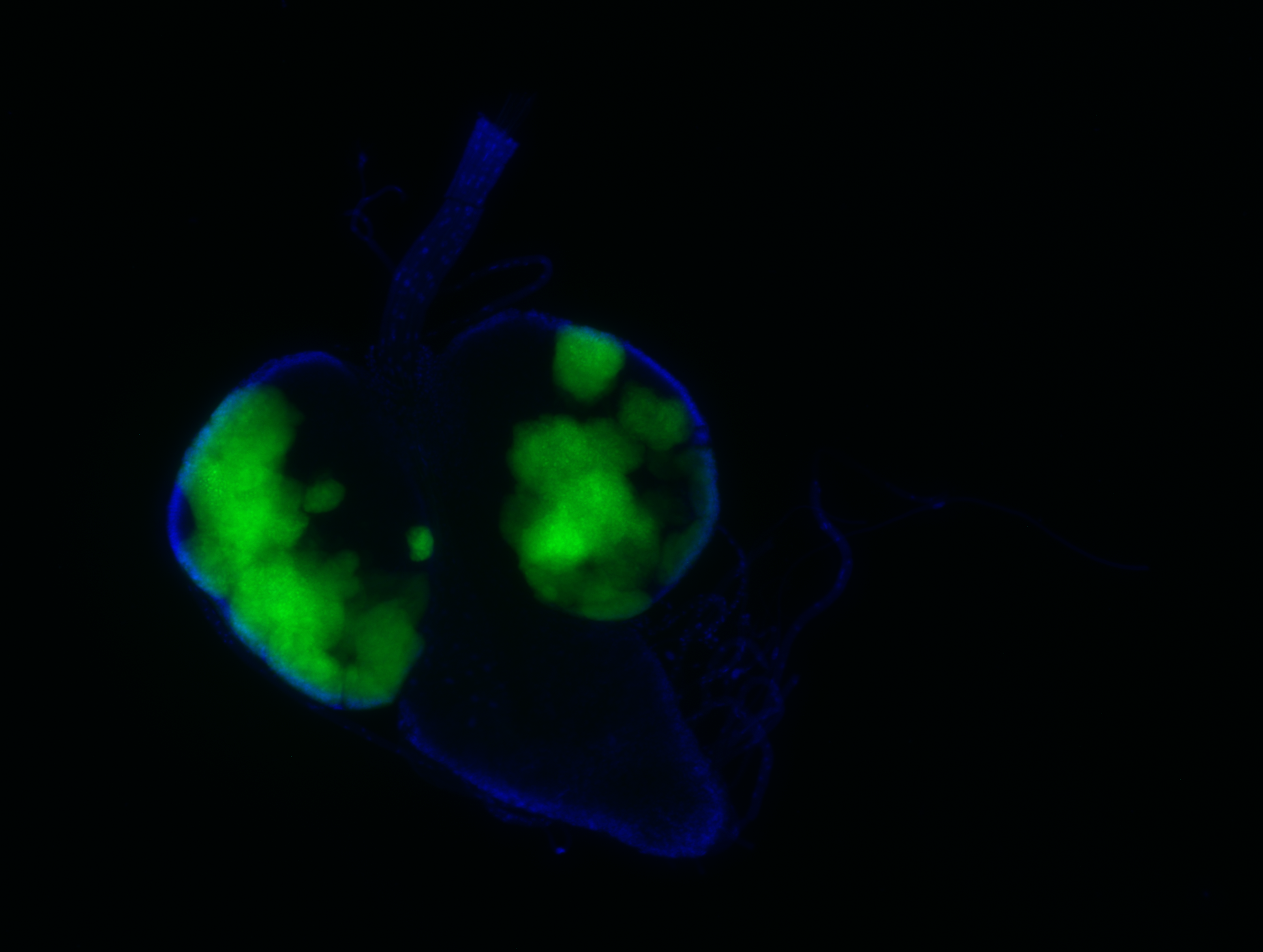

Supplement: Supplementary file 7 — Source data Fig. 3 [file 44318_2025_489_MOESM7_ESM.zip › Figure 3D/6-2 original image.tif]

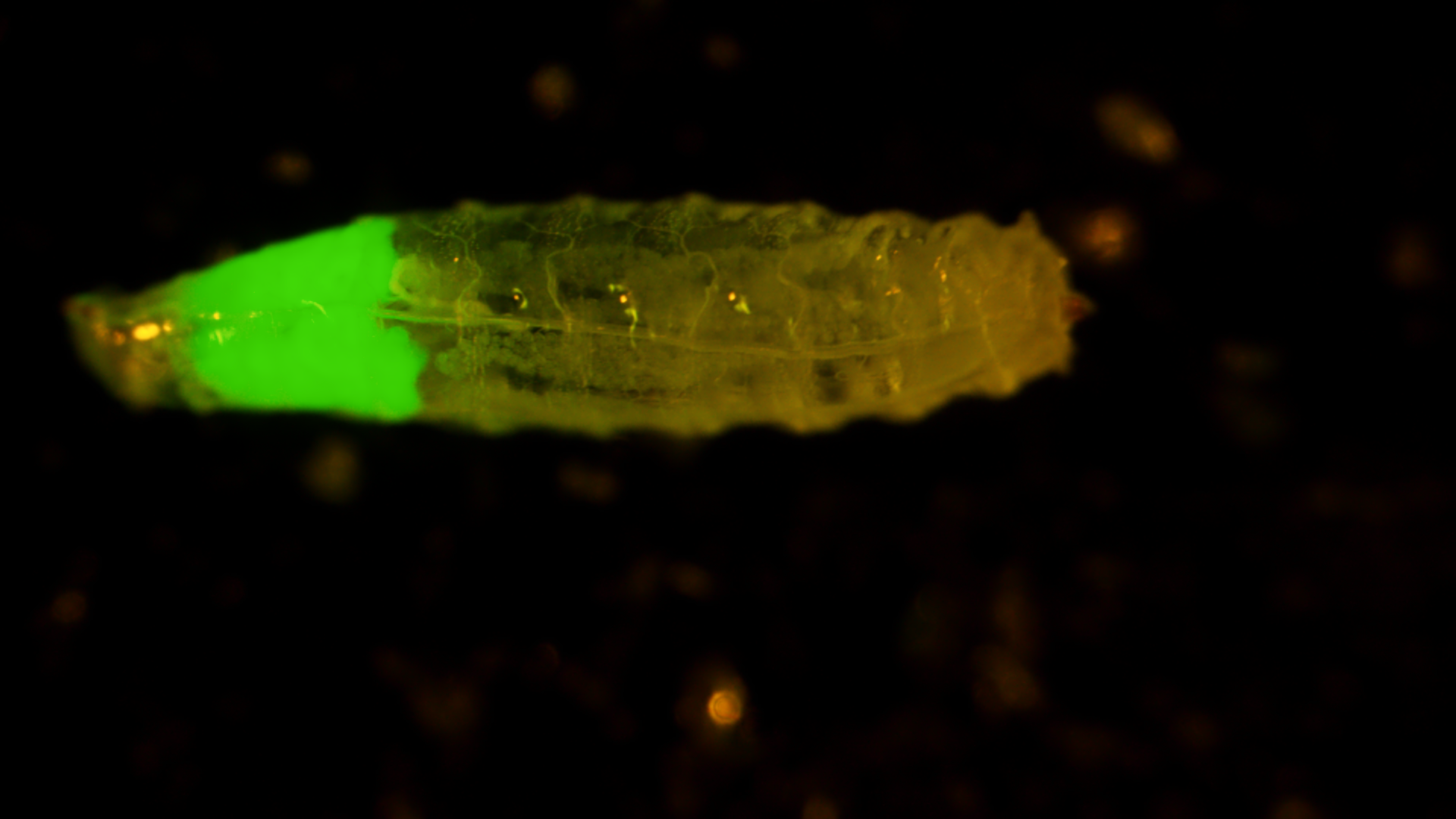

Supplement: Supplementary file 7 — Source data Fig. 3 [file 44318_2025_489_MOESM7_ESM.zip › Figure 3D/7 original image.tif]
