# Supplementary figures and images for: Adipose tissue-secreted Spz5 promotes distal tumor progression via Toll-6-mediated Hh pathway activation in Drosophila (part 3 of 5)
Source: EMBO J. 2025 Jun 23;44(15):4301–30. doi: 10.1038/s44318-025-00489-y (PMC12317064; doi:10.1038/s44318-025-00489-y)

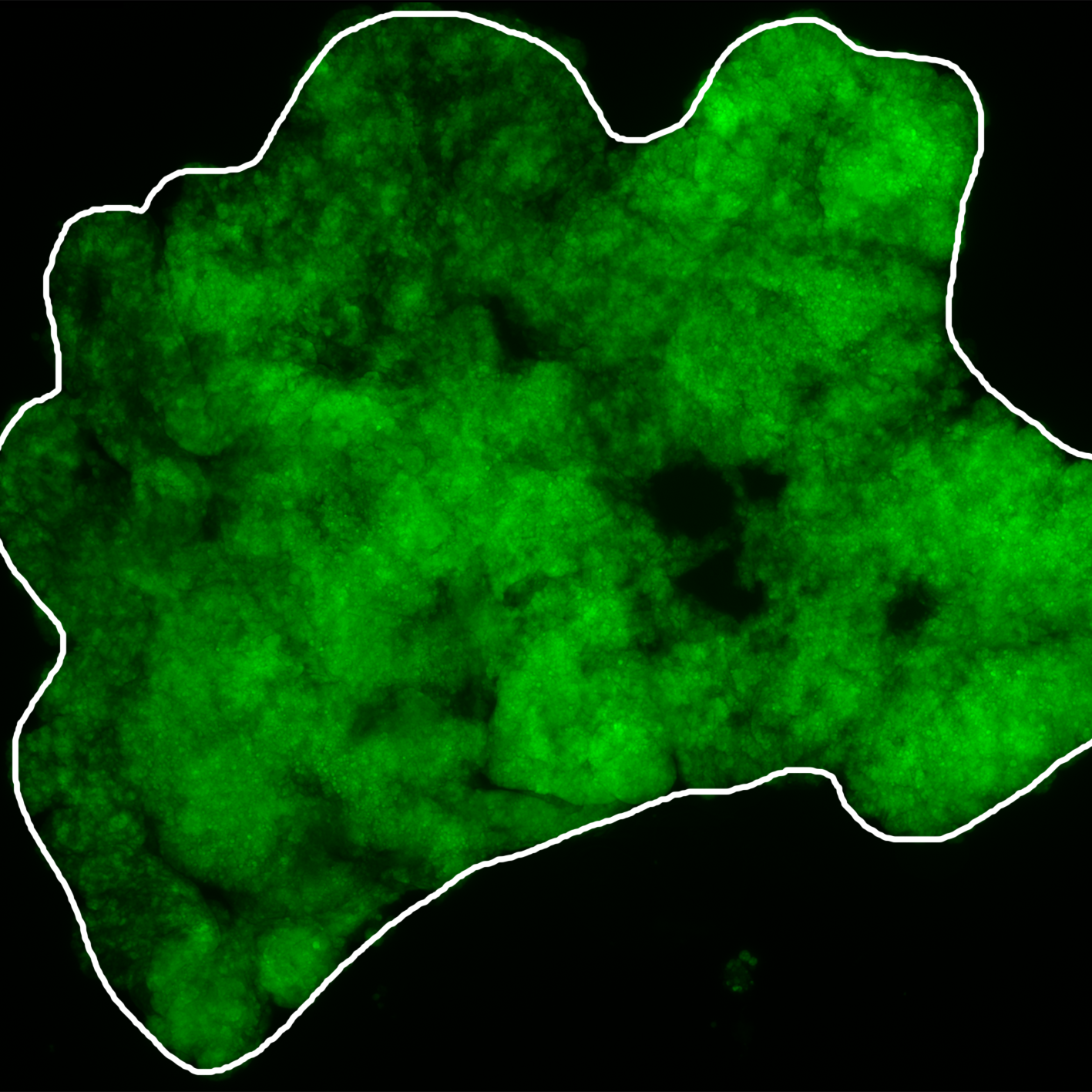

Supplement: Supplementary file 7 — Source data Fig. 3 [file 44318_2025_489_MOESM7_ESM.zip › Figure 3D/8-1 rotated and cut image with border line.tif]

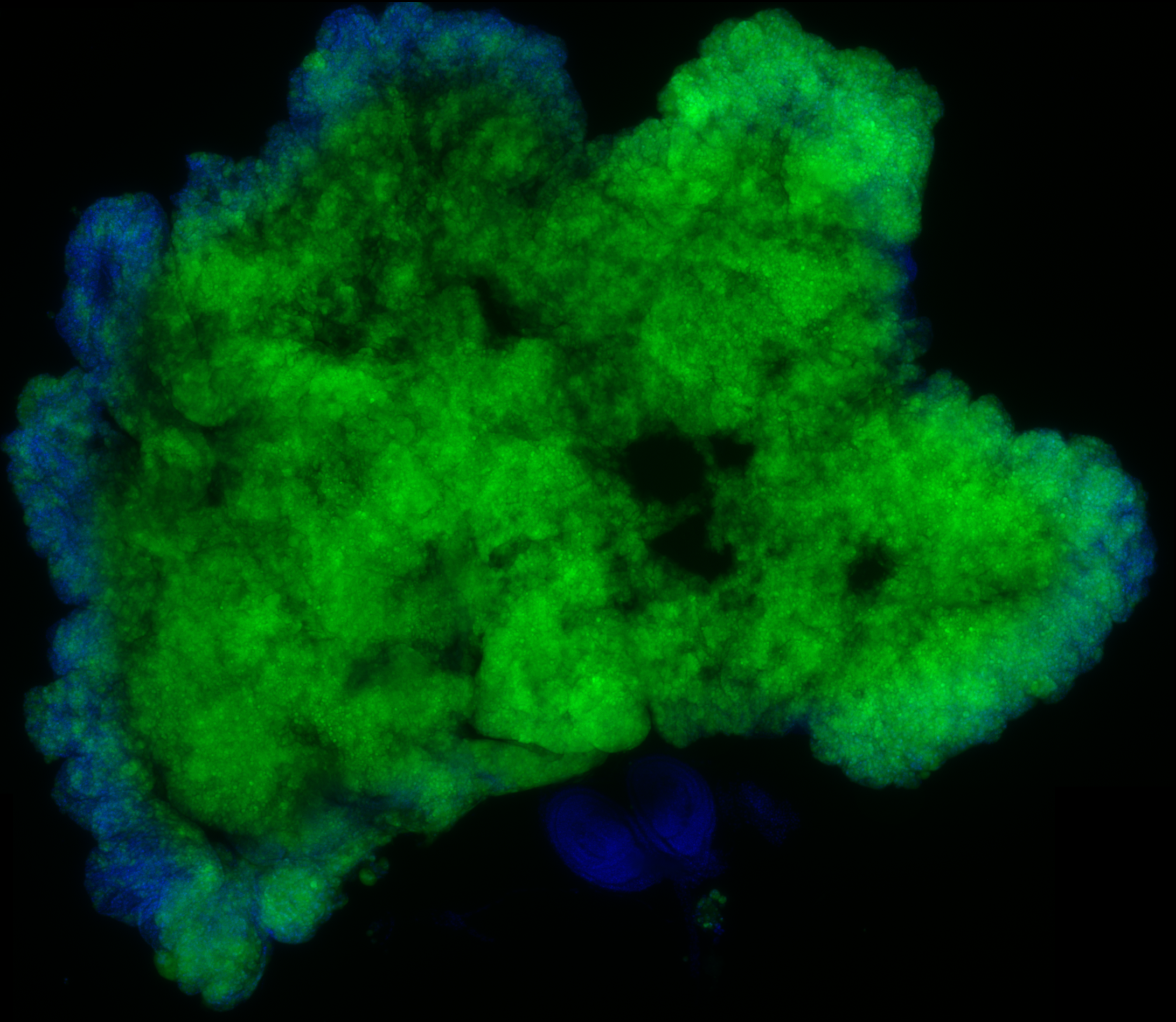

Supplement: Supplementary file 7 — Source data Fig. 3 [file 44318_2025_489_MOESM7_ESM.zip › Figure 3D/8-2 original image.tif]

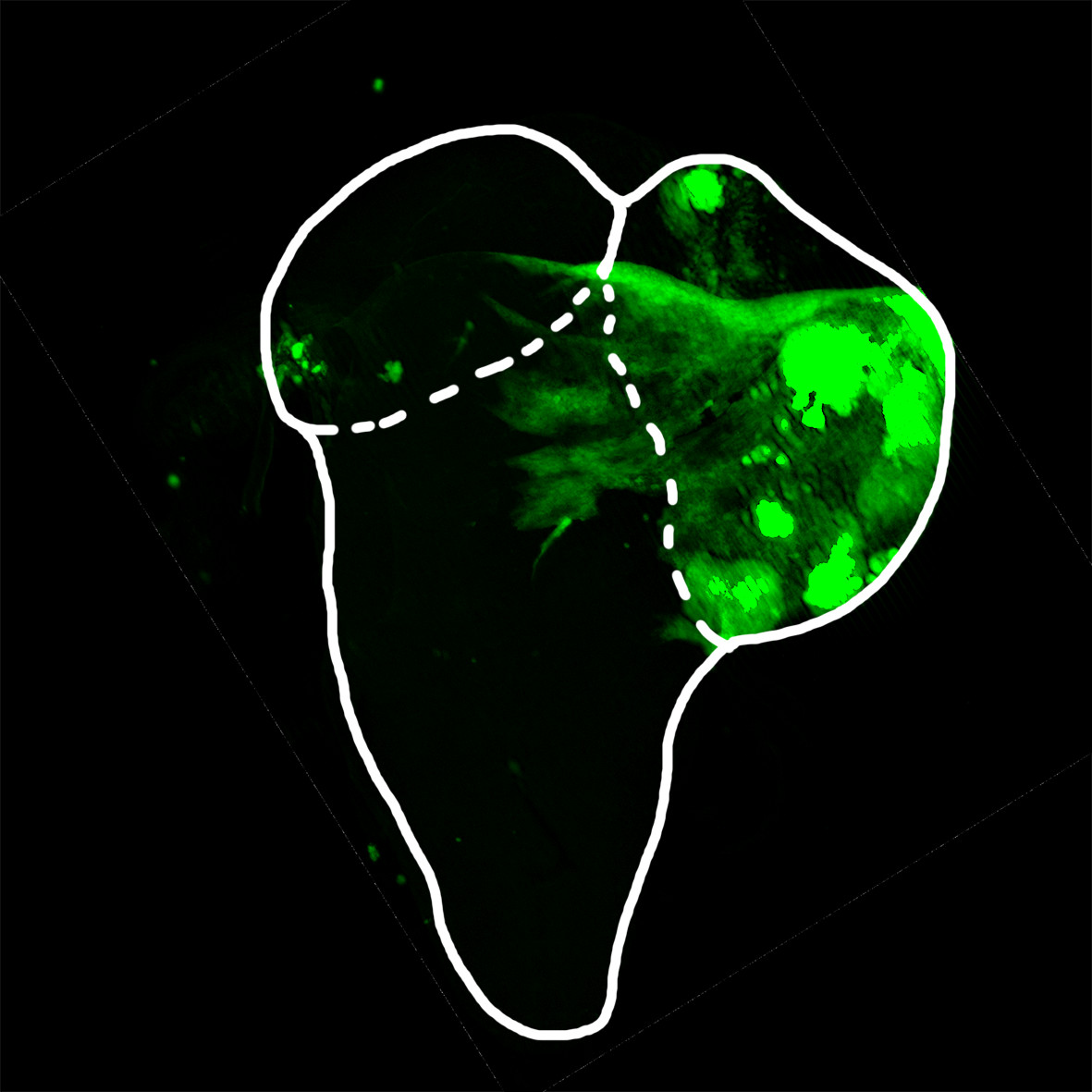

Supplement: Supplementary file 7 — Source data Fig. 3 [file 44318_2025_489_MOESM7_ESM.zip › Figure 3D/9-1 rotated and cut image with border line.tif]

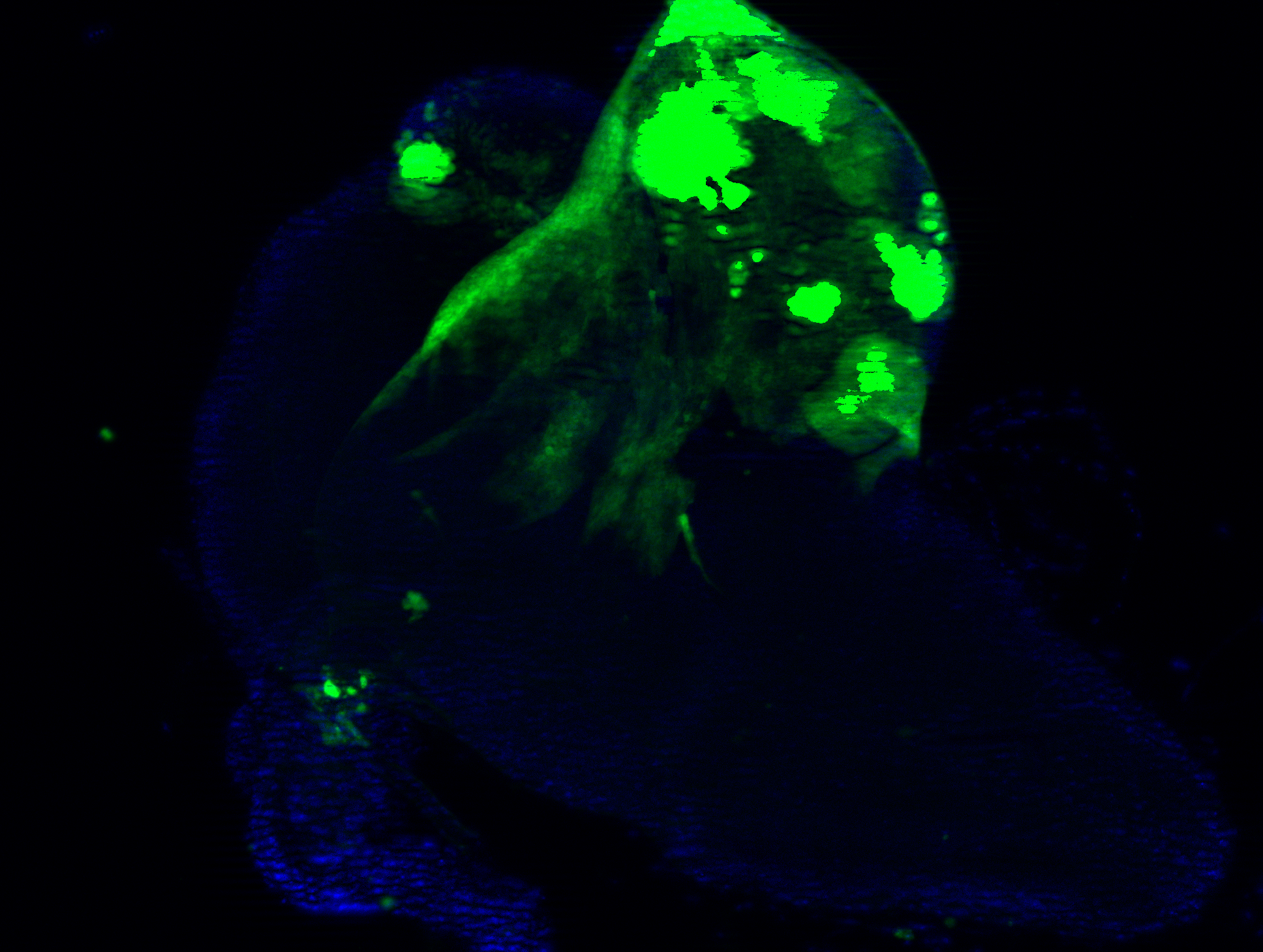

Supplement: Supplementary file 7 — Source data Fig. 3 [file 44318_2025_489_MOESM7_ESM.zip › Figure 3D/9-2 original image.tif]

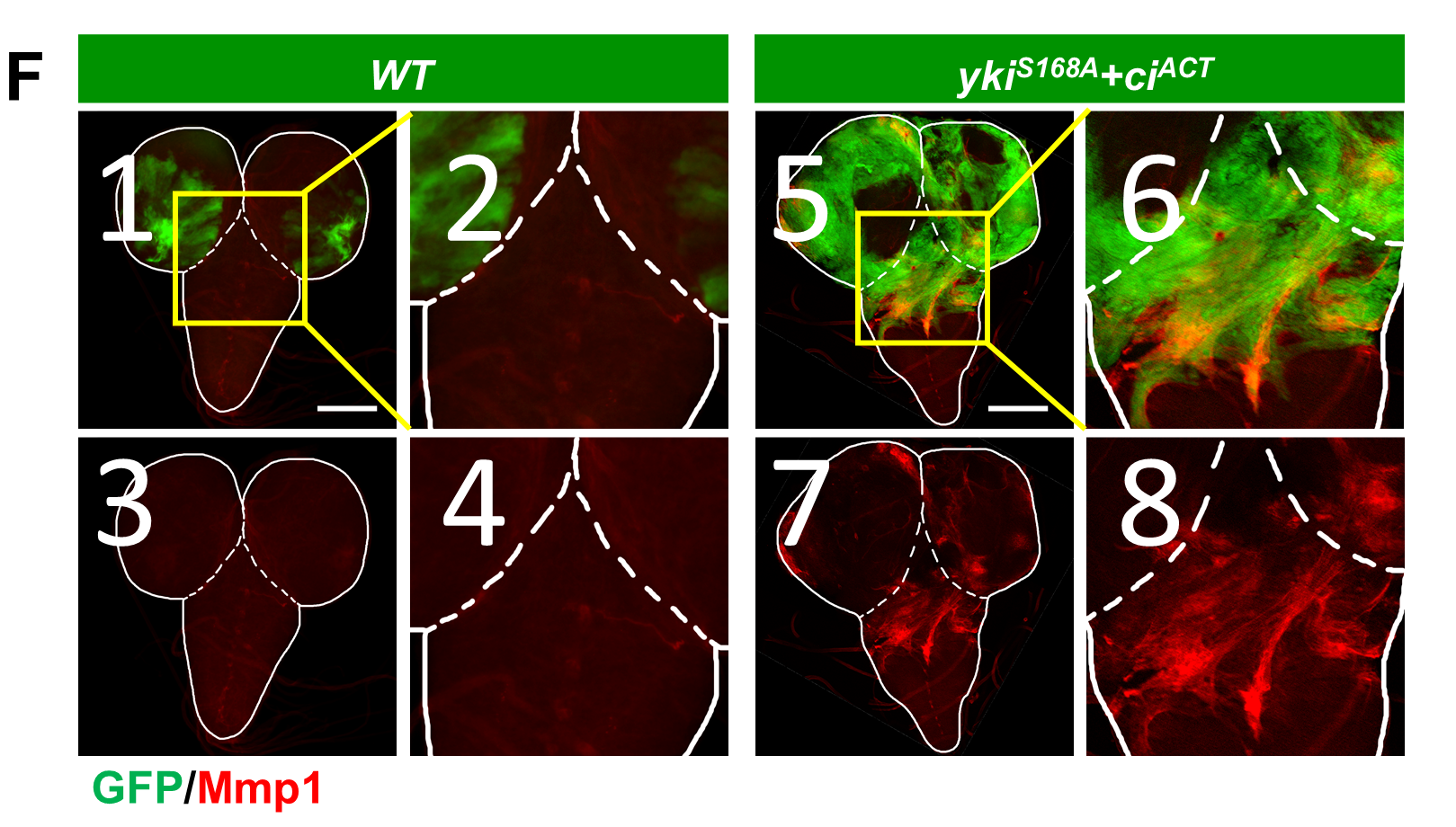

Supplement: Supplementary file 7 — Source data Fig. 3 [file 44318_2025_489_MOESM7_ESM.zip › Figure 3F/0 paper Figure 3F with provided image sequence.tif]

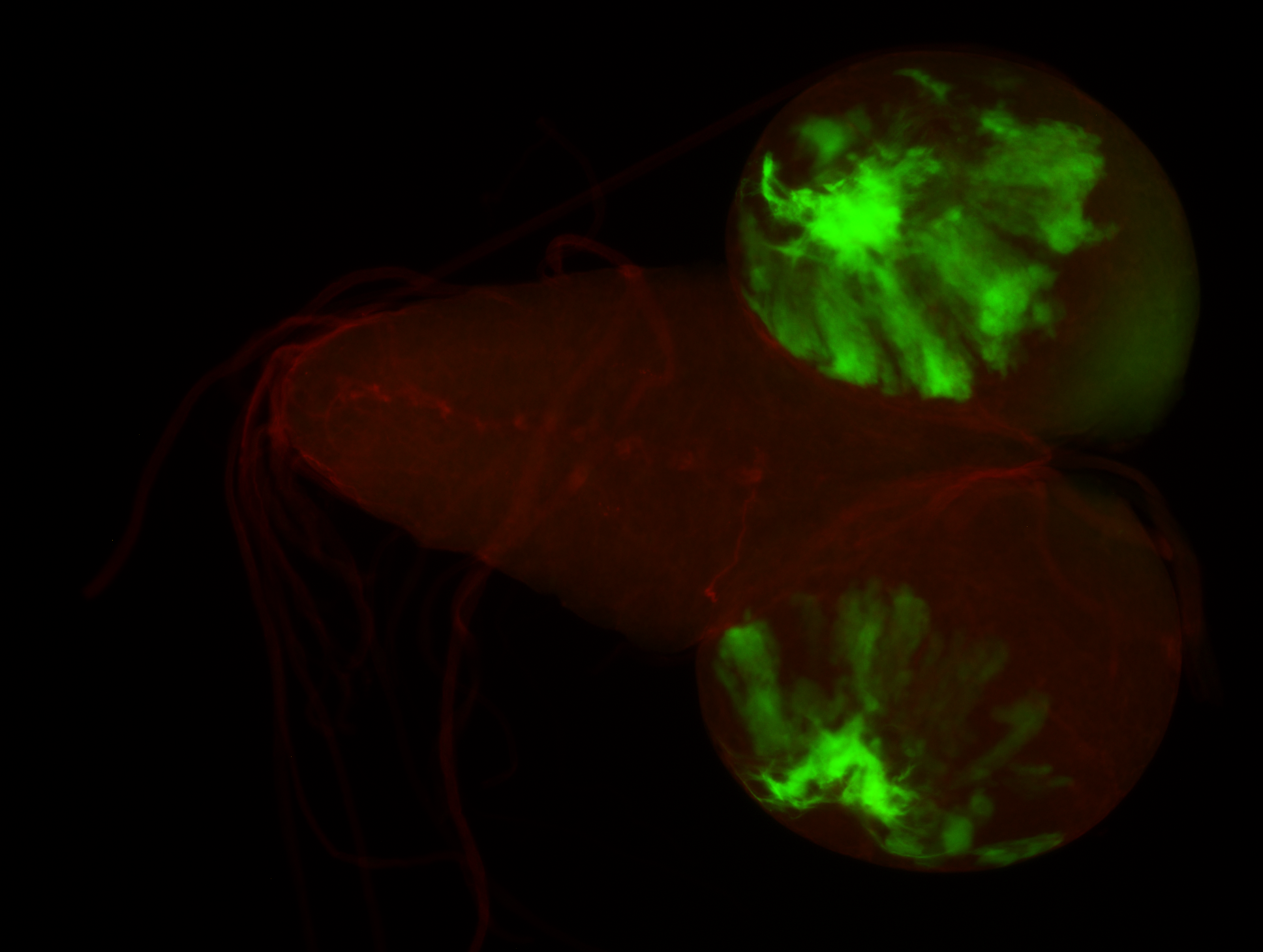

Supplement: Supplementary file 7 — Source data Fig. 3 [file 44318_2025_489_MOESM7_ESM.zip › Figure 3F/1 original image.tif]

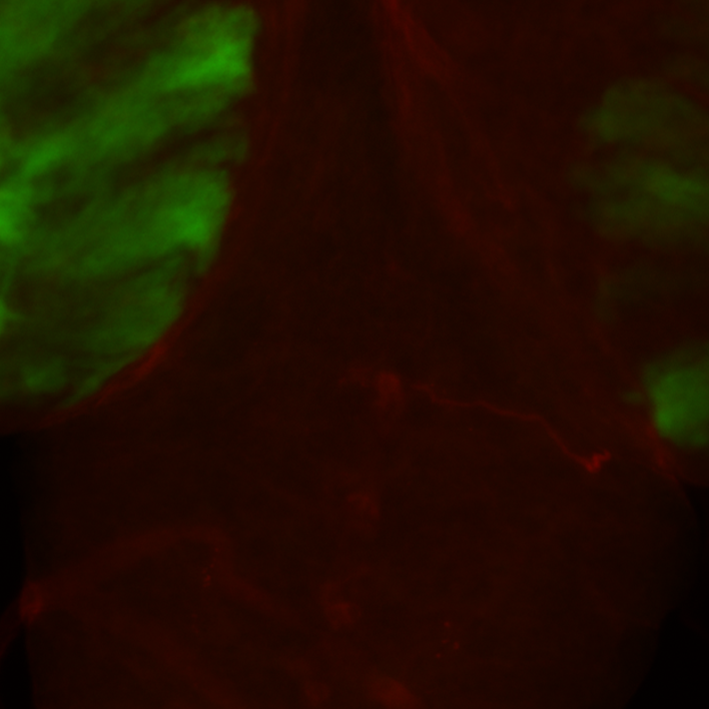

Supplement: Supplementary file 7 — Source data Fig. 3 [file 44318_2025_489_MOESM7_ESM.zip › Figure 3F/2 original image.tif]

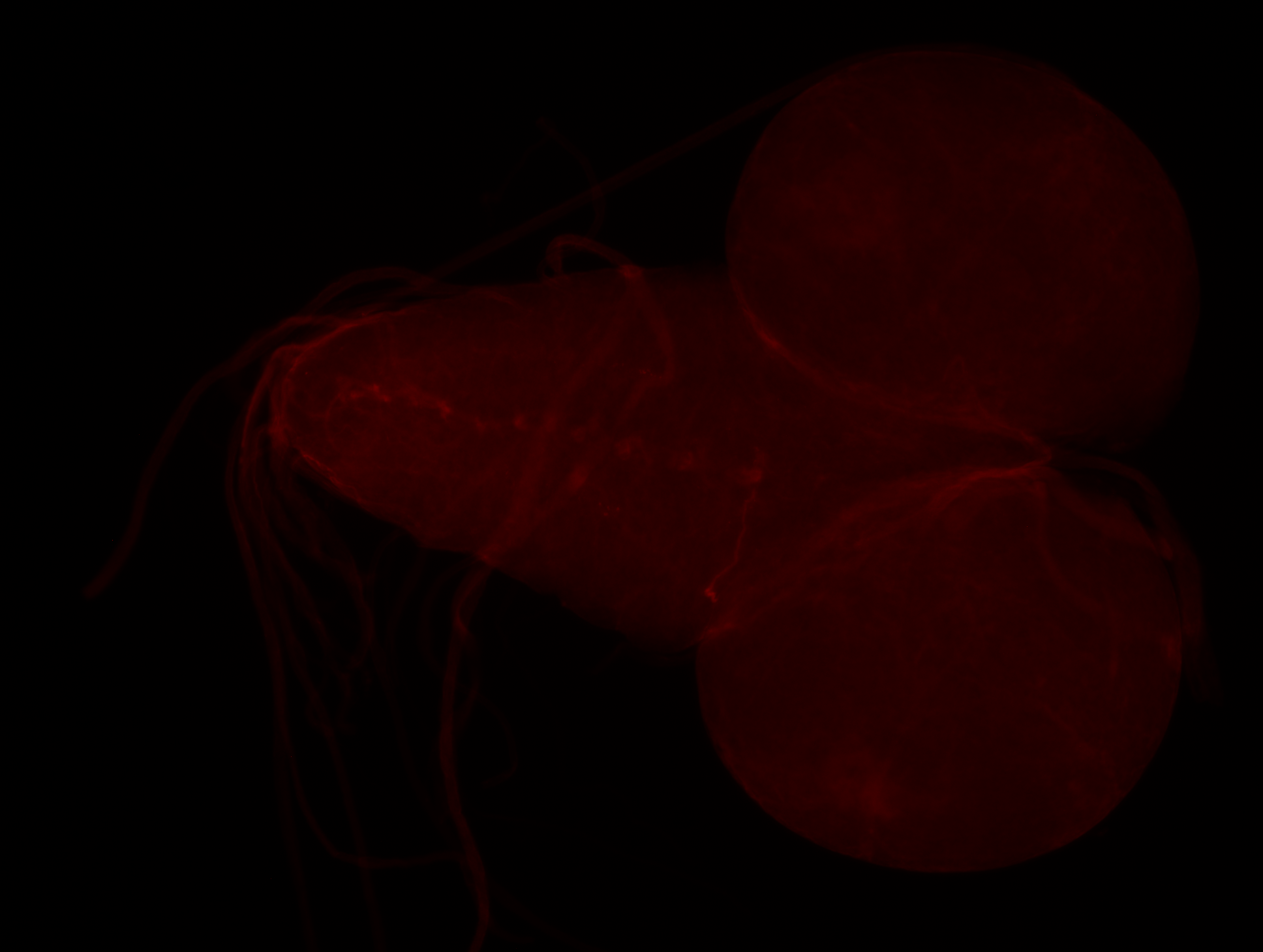

Supplement: Supplementary file 7 — Source data Fig. 3 [file 44318_2025_489_MOESM7_ESM.zip › Figure 3F/3 original image.tif]

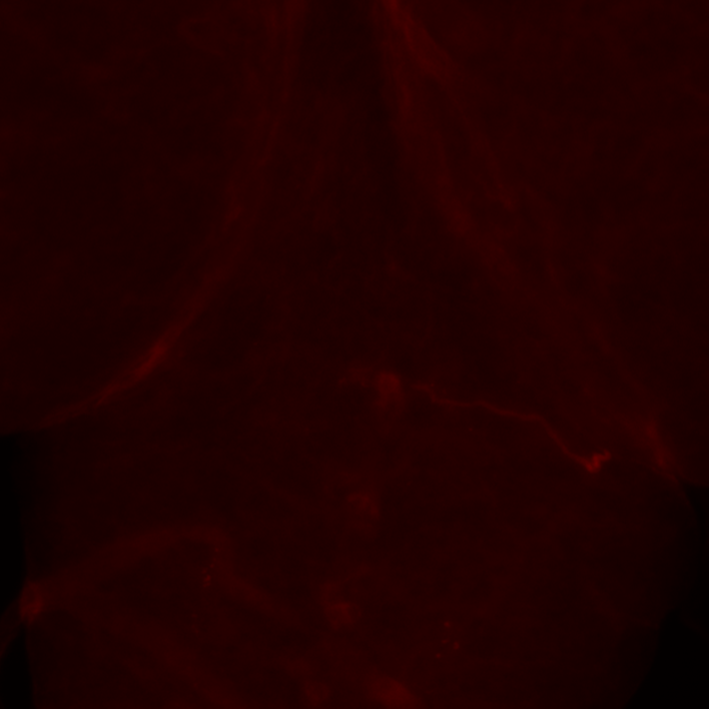

Supplement: Supplementary file 7 — Source data Fig. 3 [file 44318_2025_489_MOESM7_ESM.zip › Figure 3F/4 original image.tif]

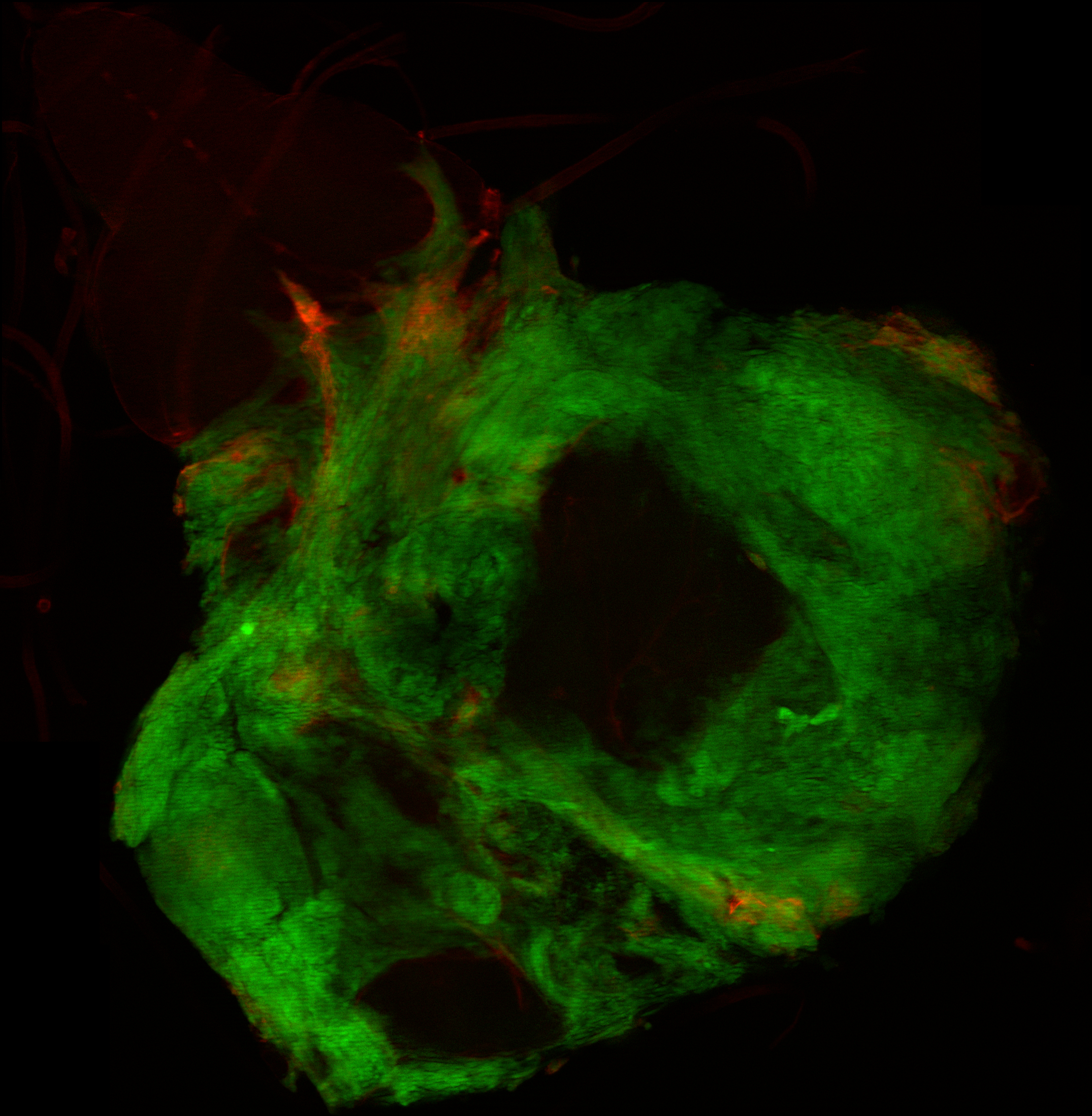

Supplement: Supplementary file 7 — Source data Fig. 3 [file 44318_2025_489_MOESM7_ESM.zip › Figure 3F/5 original image.tif]

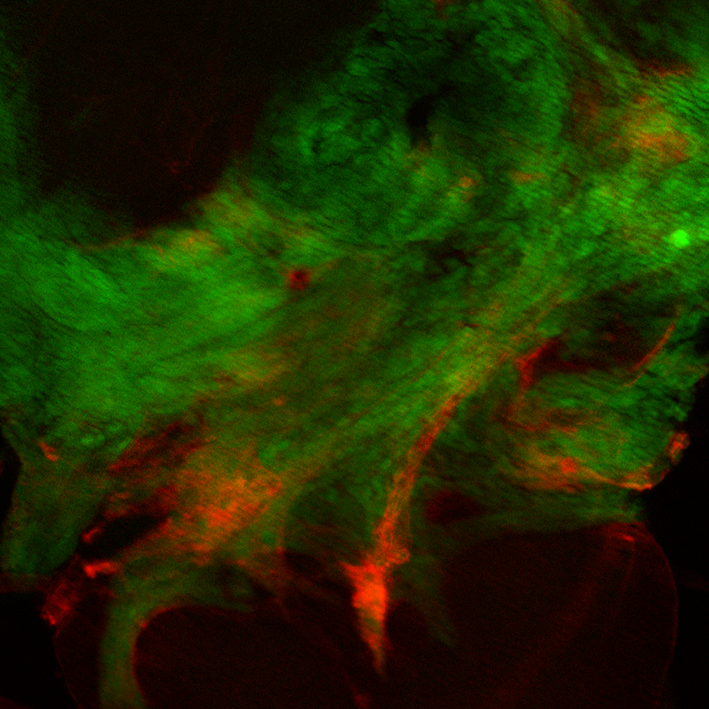

Supplement: Supplementary file 7 — Source data Fig. 3 [file 44318_2025_489_MOESM7_ESM.zip › Figure 3F/6 original image.tif]

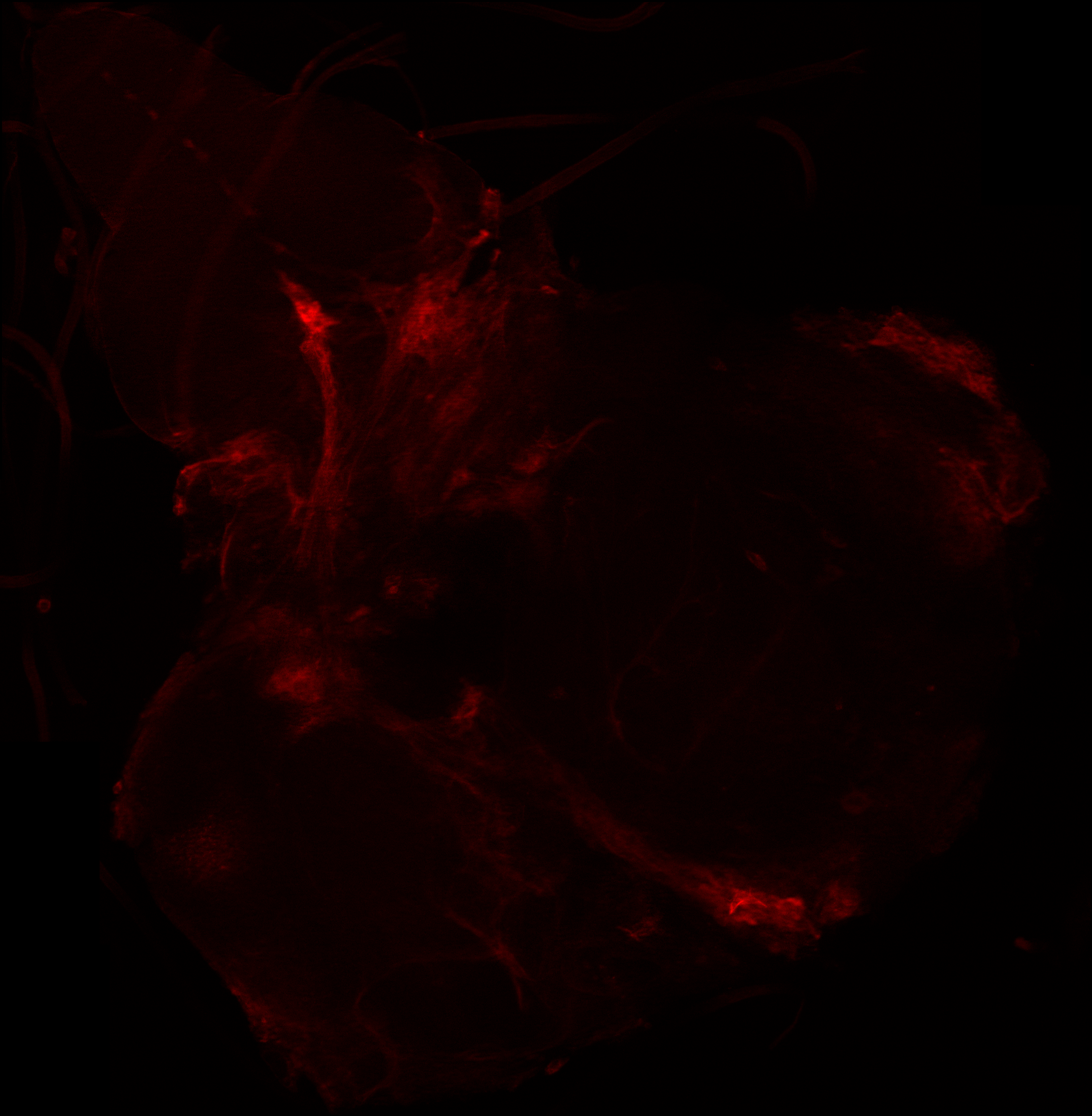

Supplement: Supplementary file 7 — Source data Fig. 3 [file 44318_2025_489_MOESM7_ESM.zip › Figure 3F/7 original image.tif]

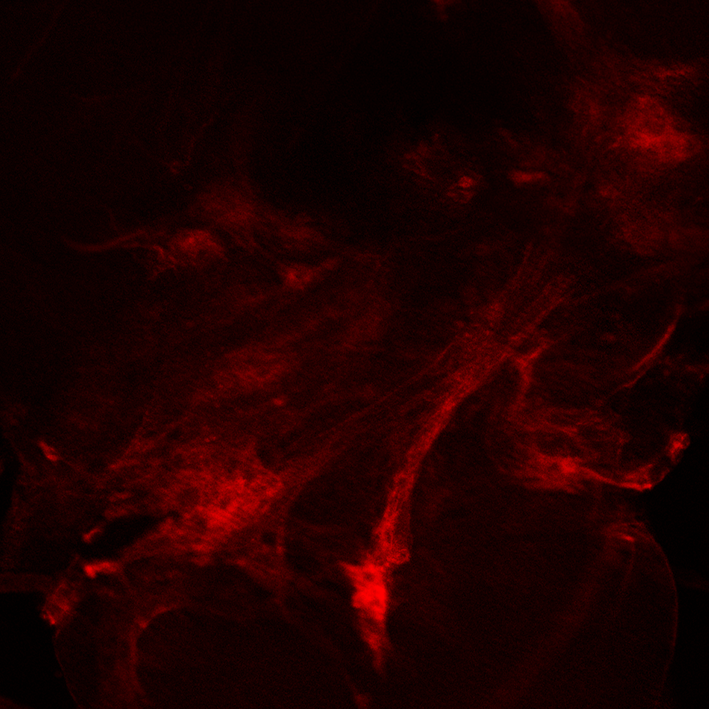

Supplement: Supplementary file 7 — Source data Fig. 3 [file 44318_2025_489_MOESM7_ESM.zip › Figure 3F/8 original image.tif]

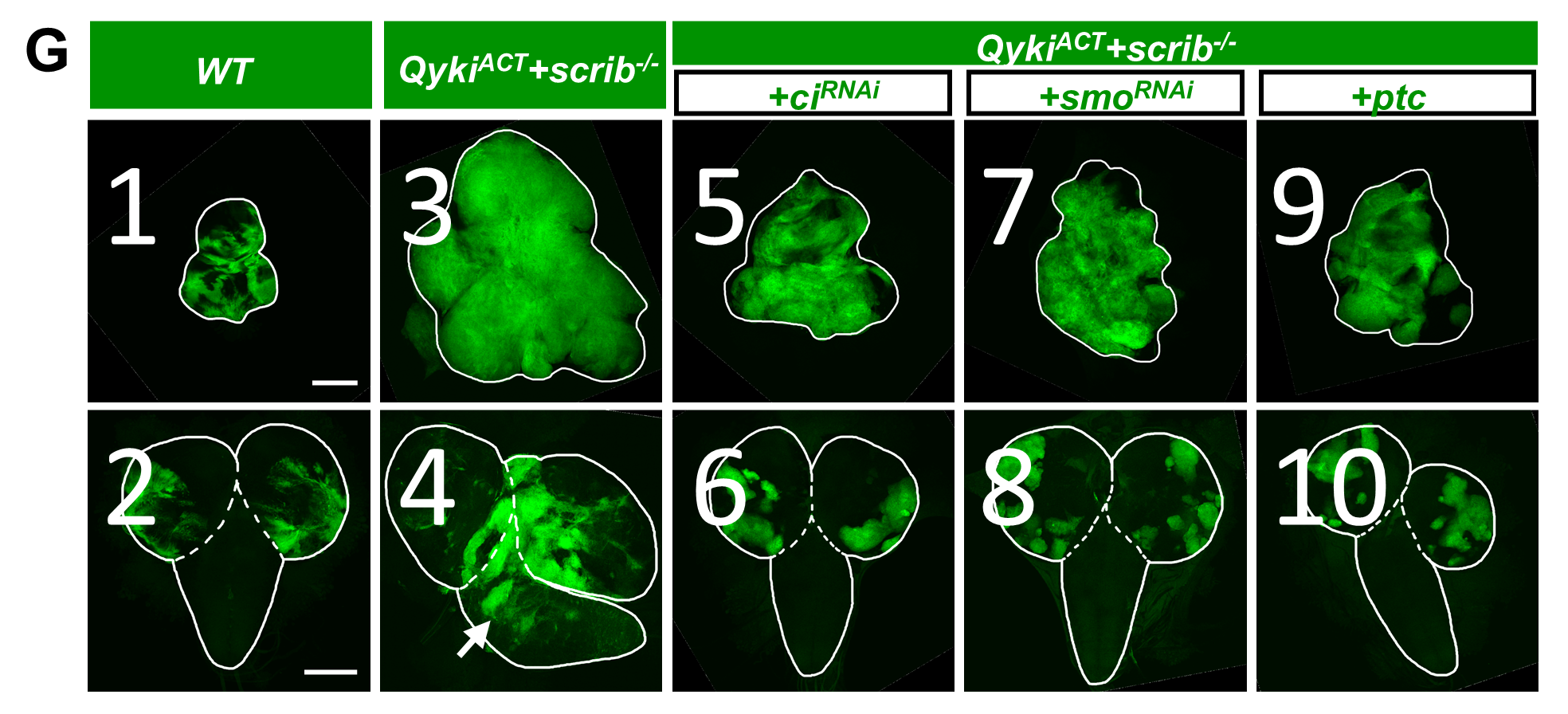

Supplement: Supplementary file 7 — Source data Fig. 3 [file 44318_2025_489_MOESM7_ESM.zip › Figure 3G/0 paper Figure 3G with provided image sequence.tif]

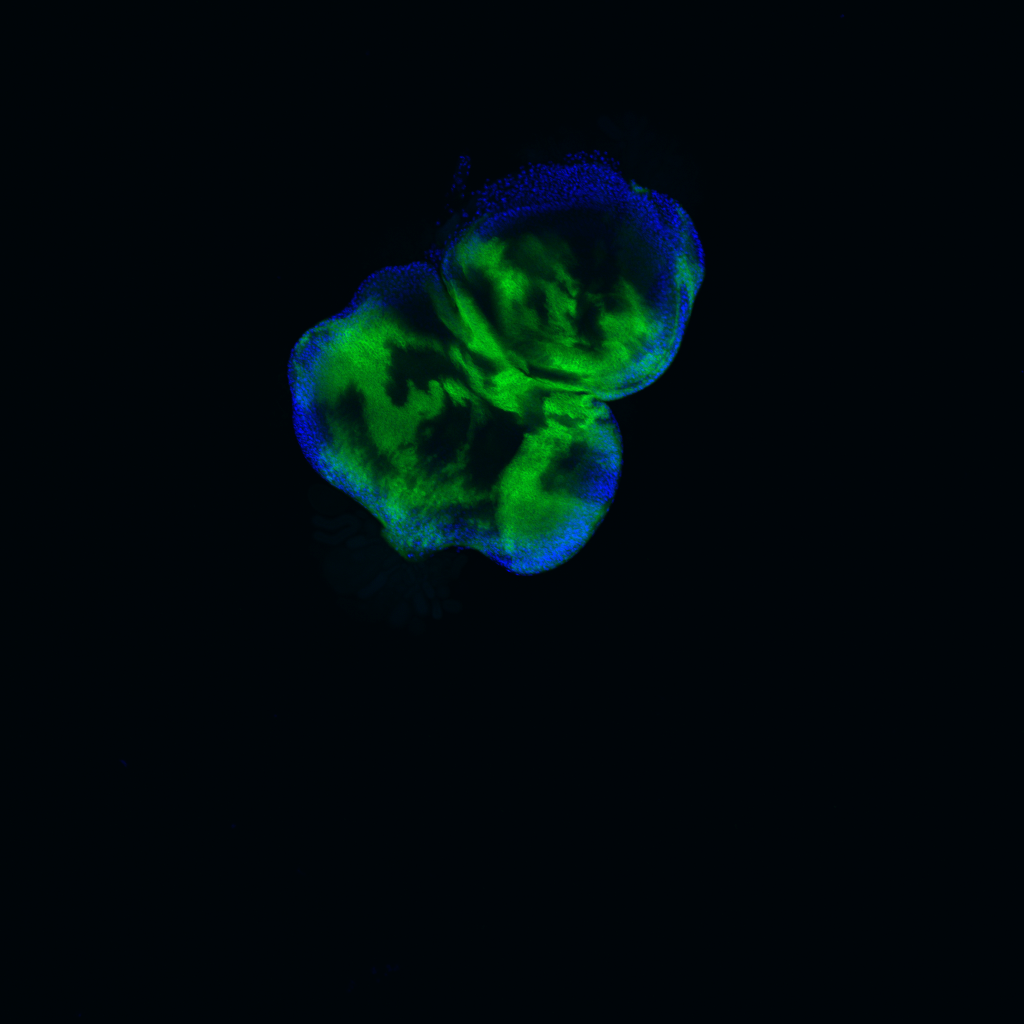

Supplement: Supplementary file 7 — Source data Fig. 3 [file 44318_2025_489_MOESM7_ESM.zip › Figure 3G/1 original image.tif]

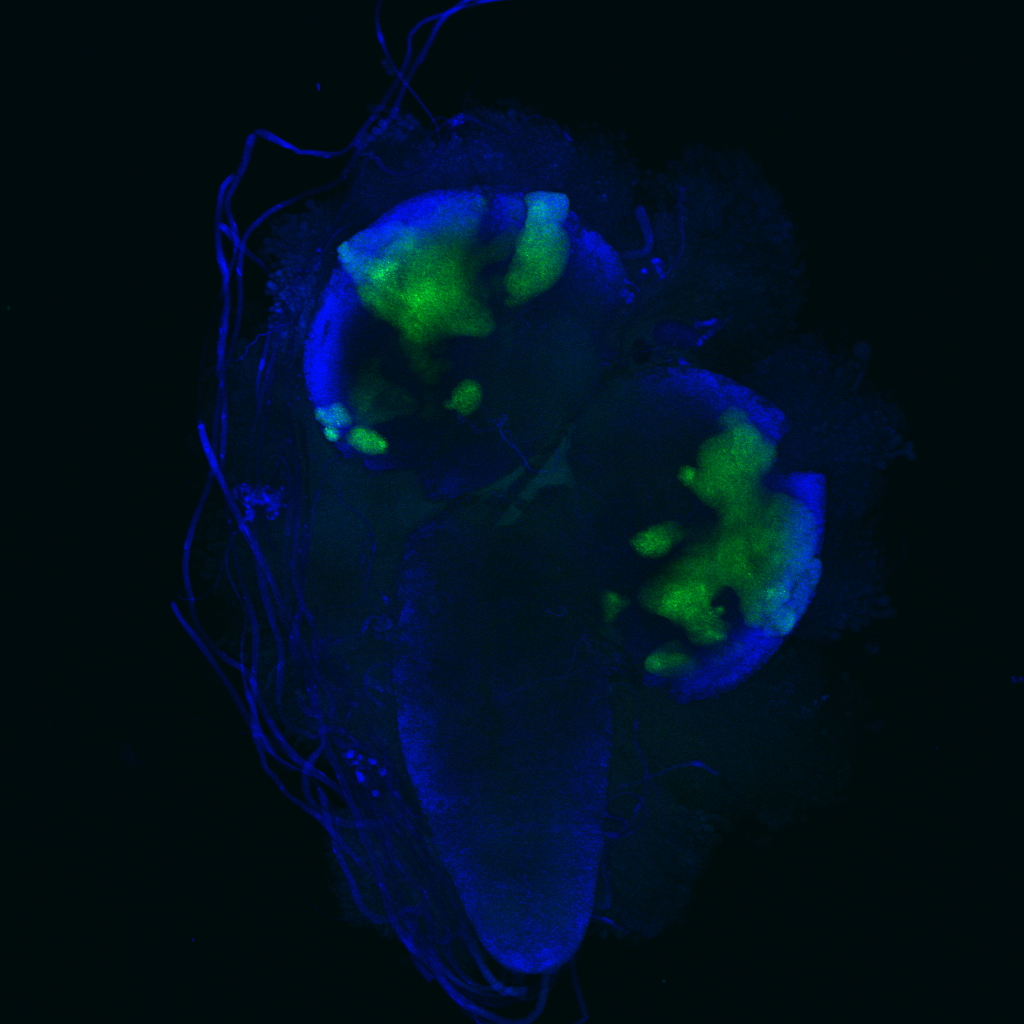

Supplement: Supplementary file 7 — Source data Fig. 3 [file 44318_2025_489_MOESM7_ESM.zip › Figure 3G/10 original image.tif]

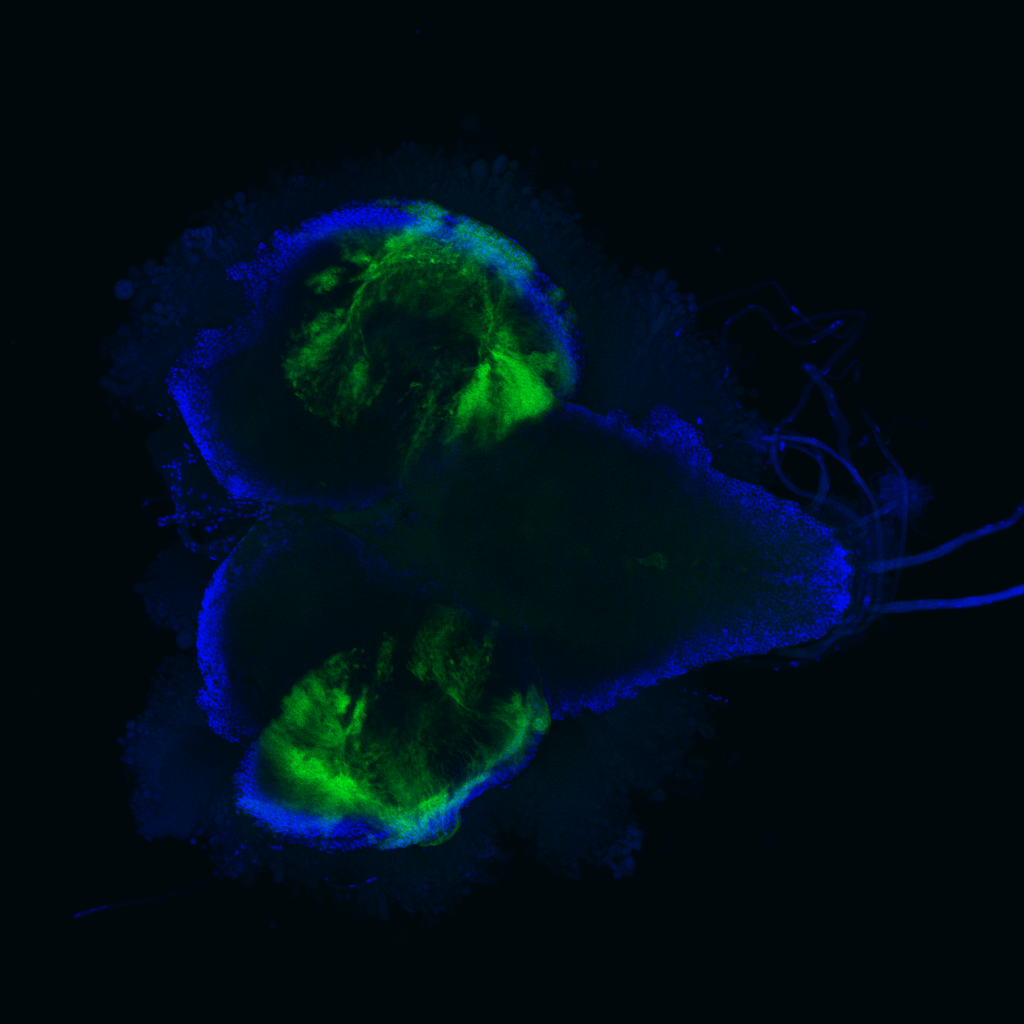

Supplement: Supplementary file 7 — Source data Fig. 3 [file 44318_2025_489_MOESM7_ESM.zip › Figure 3G/2 original image.tif]

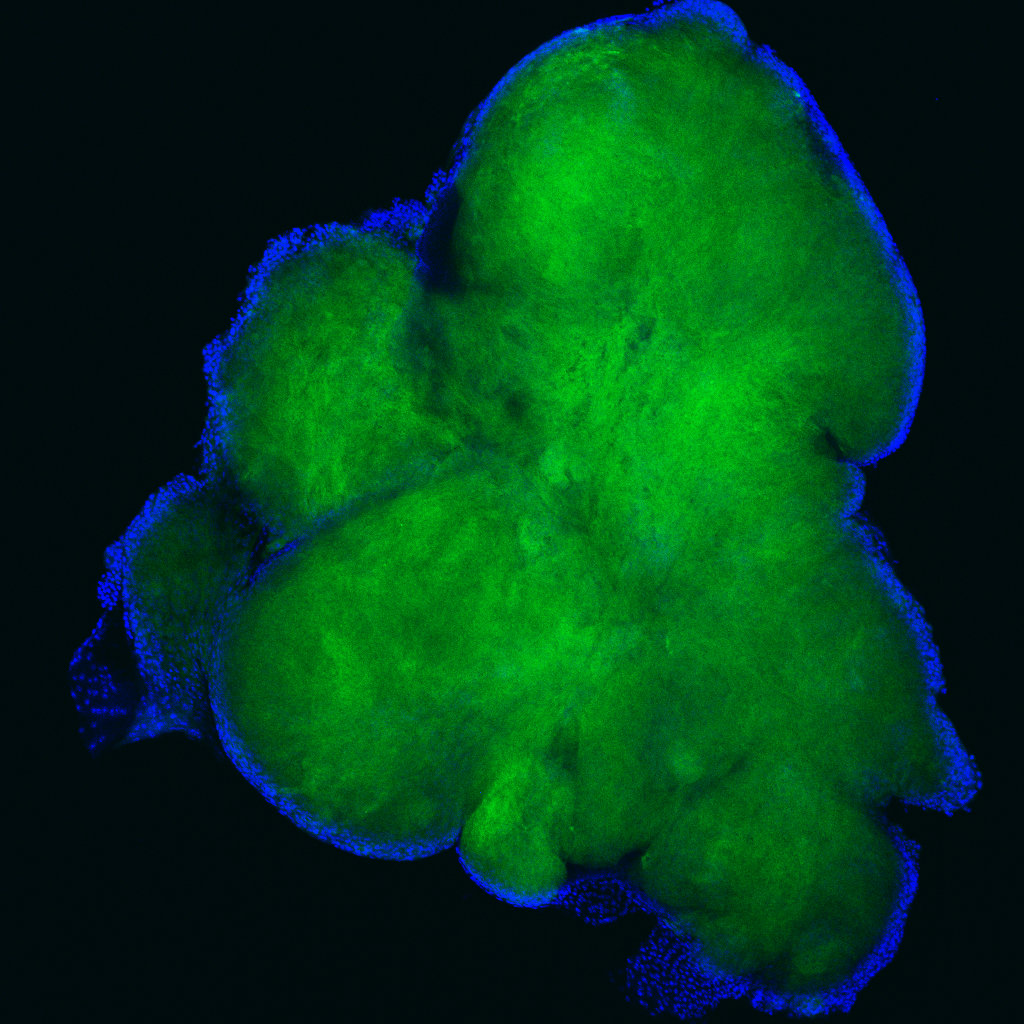

Supplement: Supplementary file 7 — Source data Fig. 3 [file 44318_2025_489_MOESM7_ESM.zip › Figure 3G/3 original image.tif]

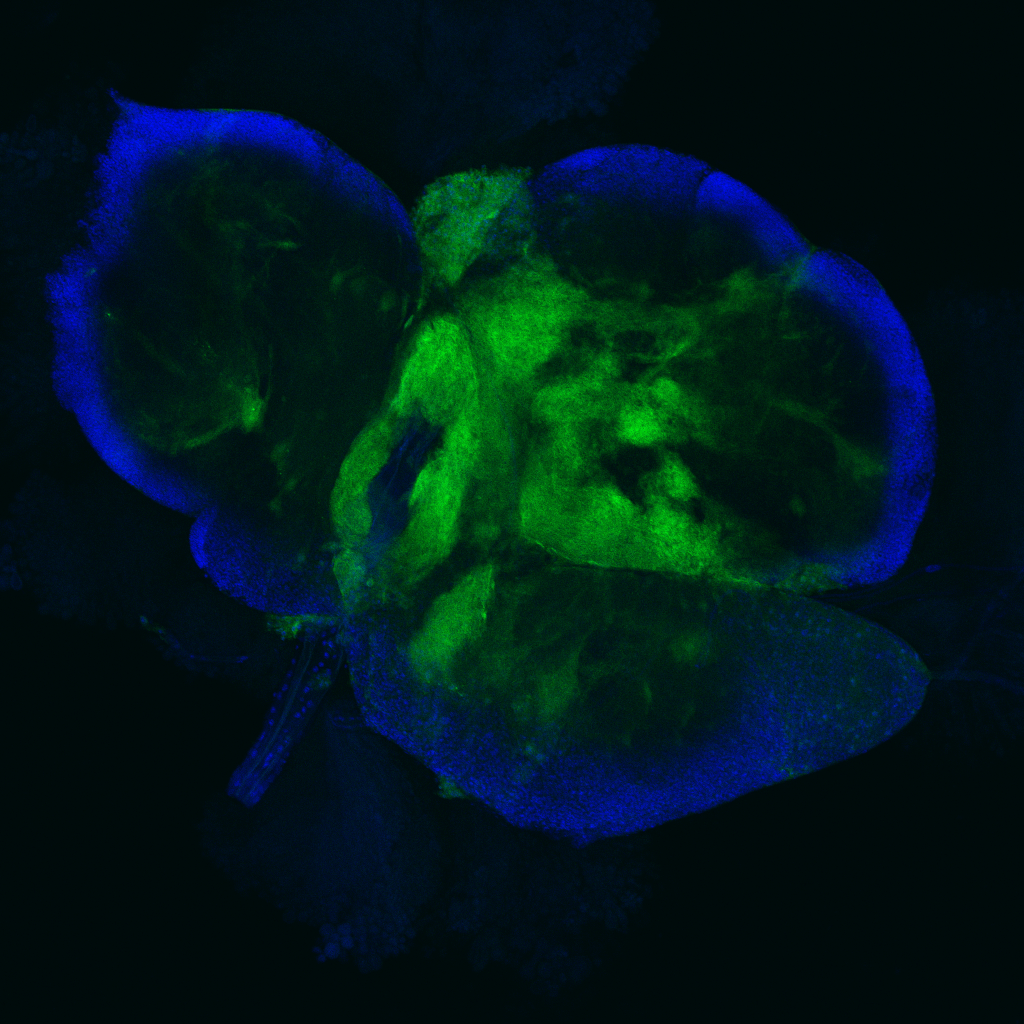

Supplement: Supplementary file 7 — Source data Fig. 3 [file 44318_2025_489_MOESM7_ESM.zip › Figure 3G/4 original image.tif]

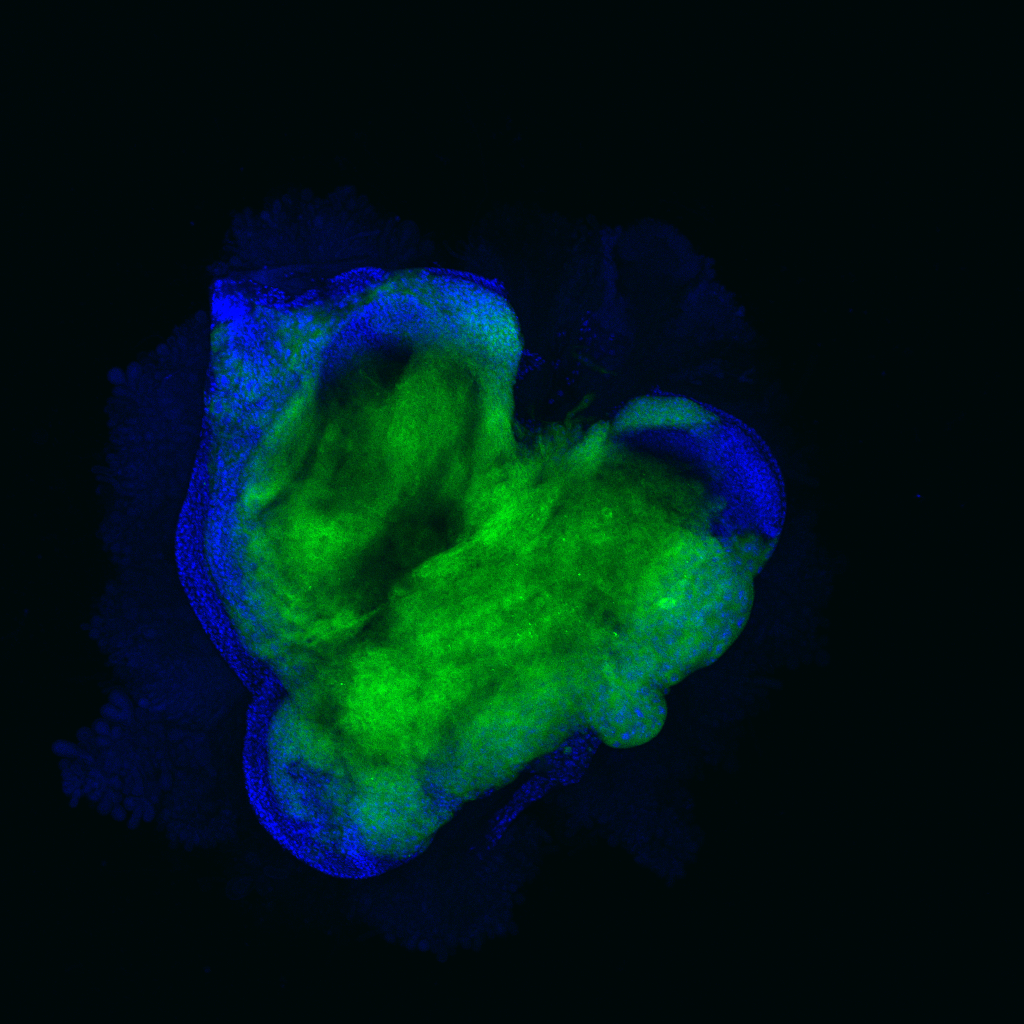

Supplement: Supplementary file 7 — Source data Fig. 3 [file 44318_2025_489_MOESM7_ESM.zip › Figure 3G/5 original image.tif]

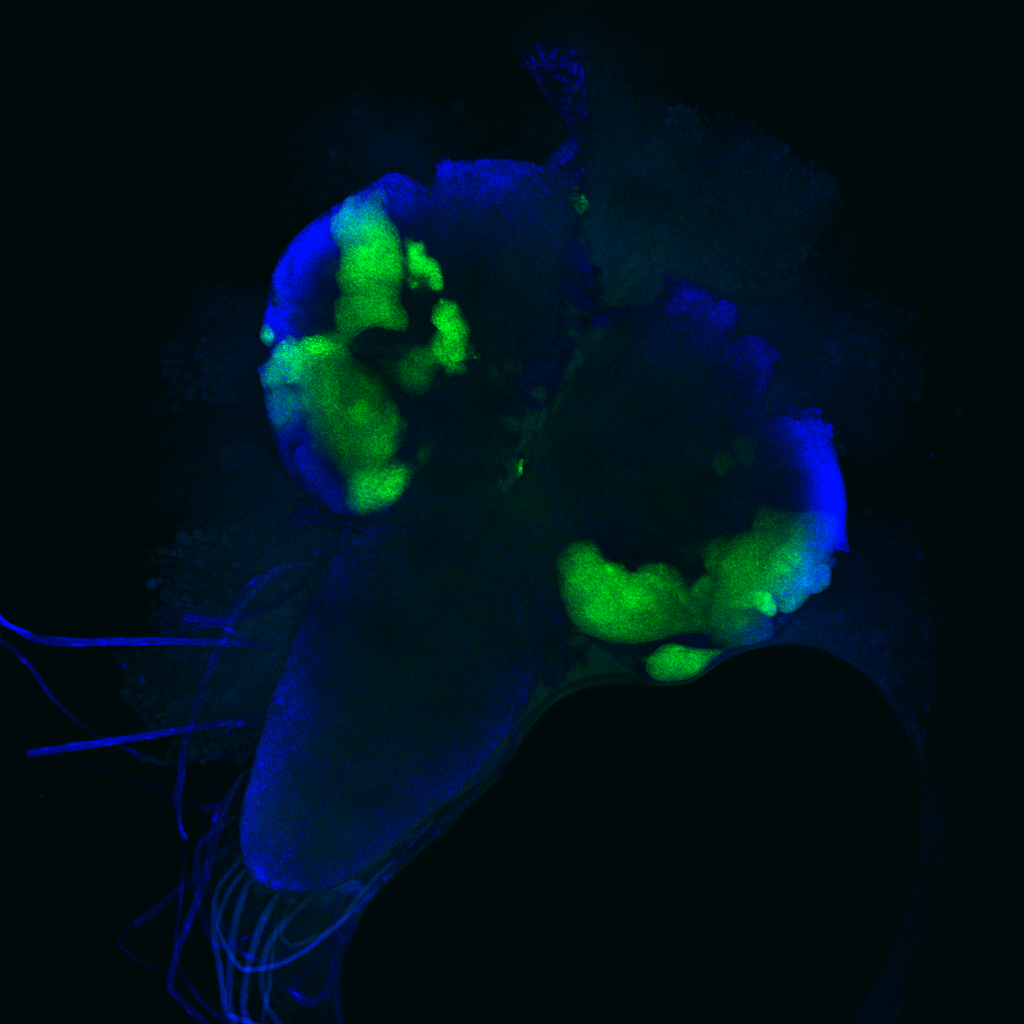

Supplement: Supplementary file 7 — Source data Fig. 3 [file 44318_2025_489_MOESM7_ESM.zip › Figure 3G/6 original image.tif]

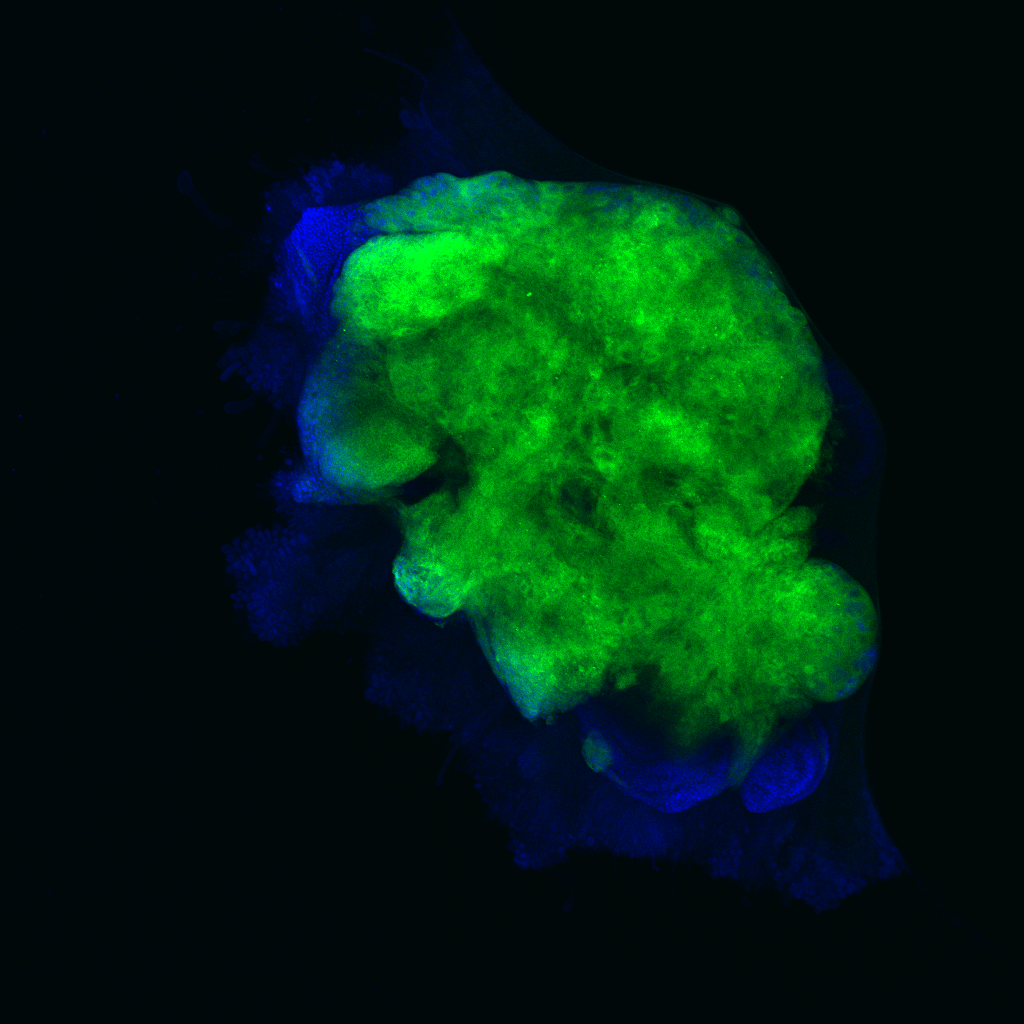

Supplement: Supplementary file 7 — Source data Fig. 3 [file 44318_2025_489_MOESM7_ESM.zip › Figure 3G/7 original image.tif]

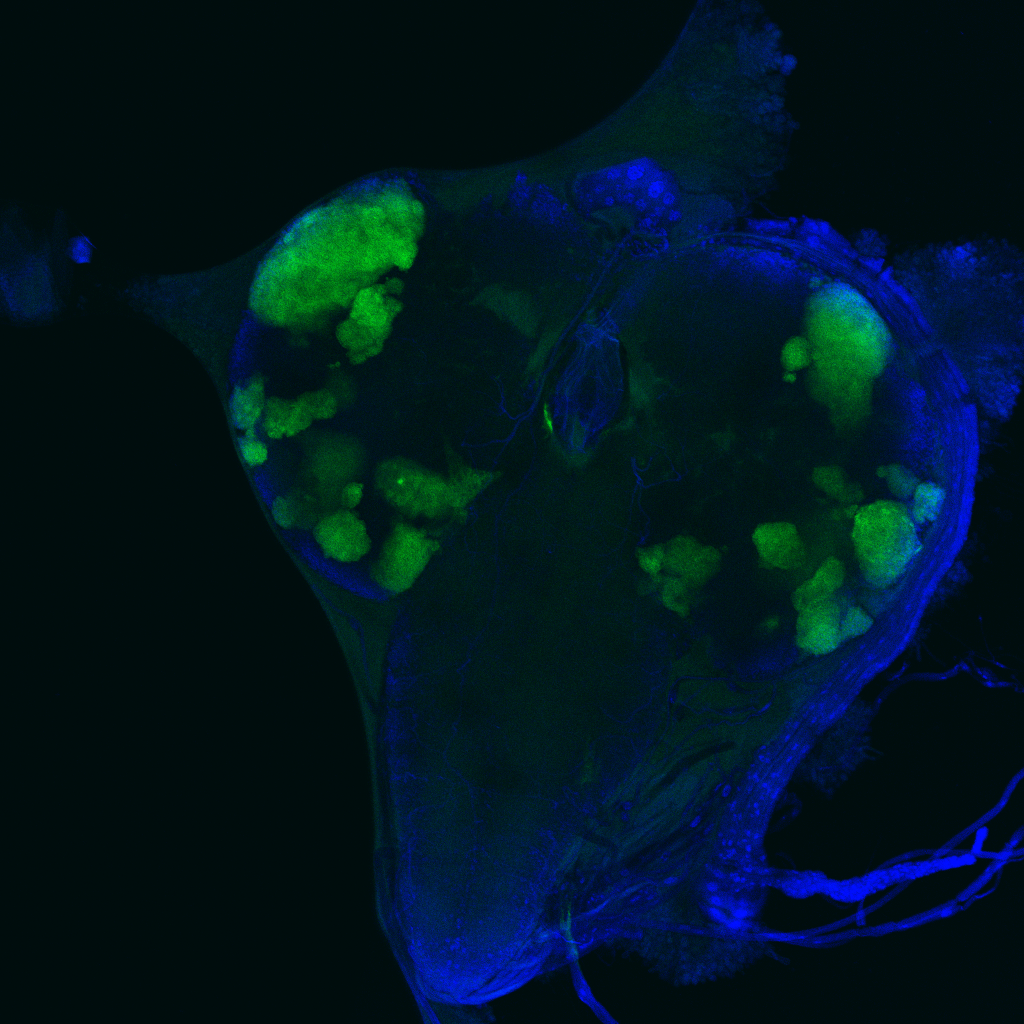

Supplement: Supplementary file 7 — Source data Fig. 3 [file 44318_2025_489_MOESM7_ESM.zip › Figure 3G/8 original image.tif]

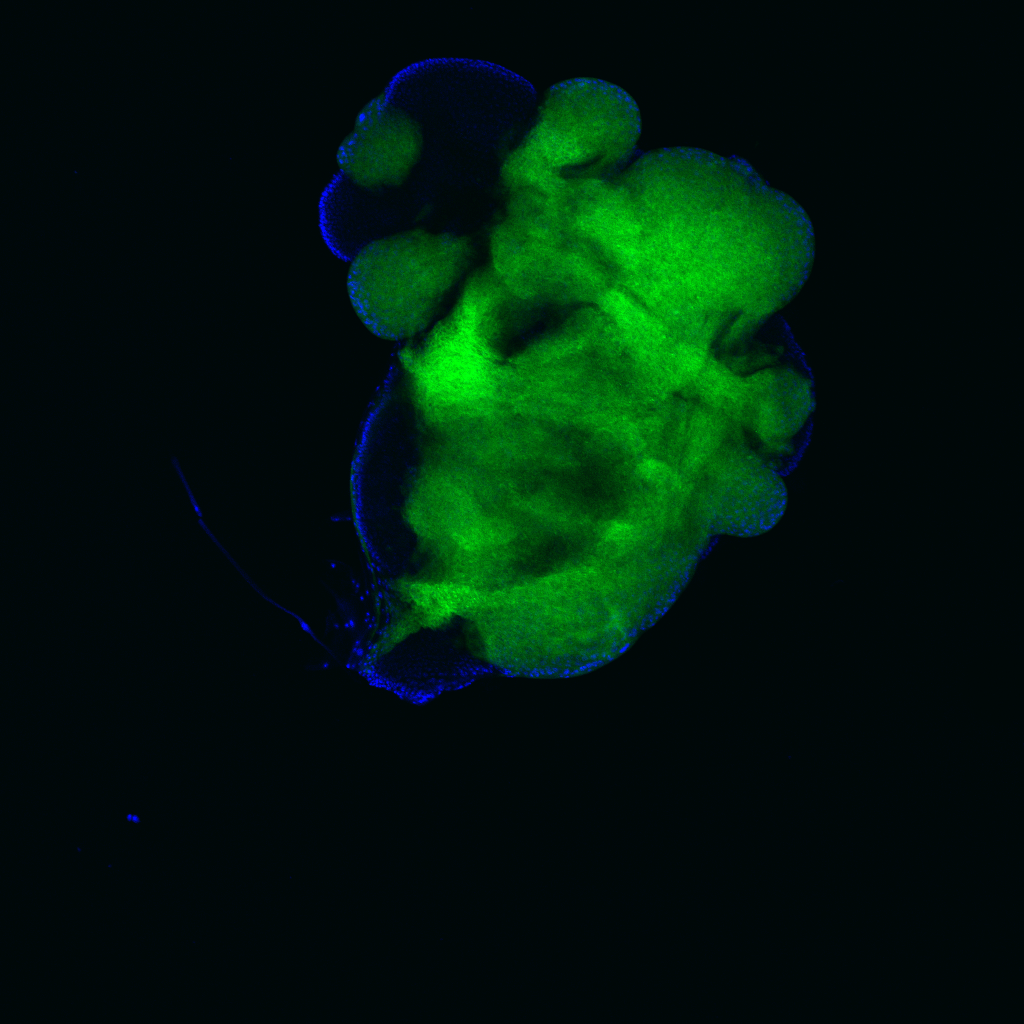

Supplement: Supplementary file 7 — Source data Fig. 3 [file 44318_2025_489_MOESM7_ESM.zip › Figure 3G/9 original image.tif]

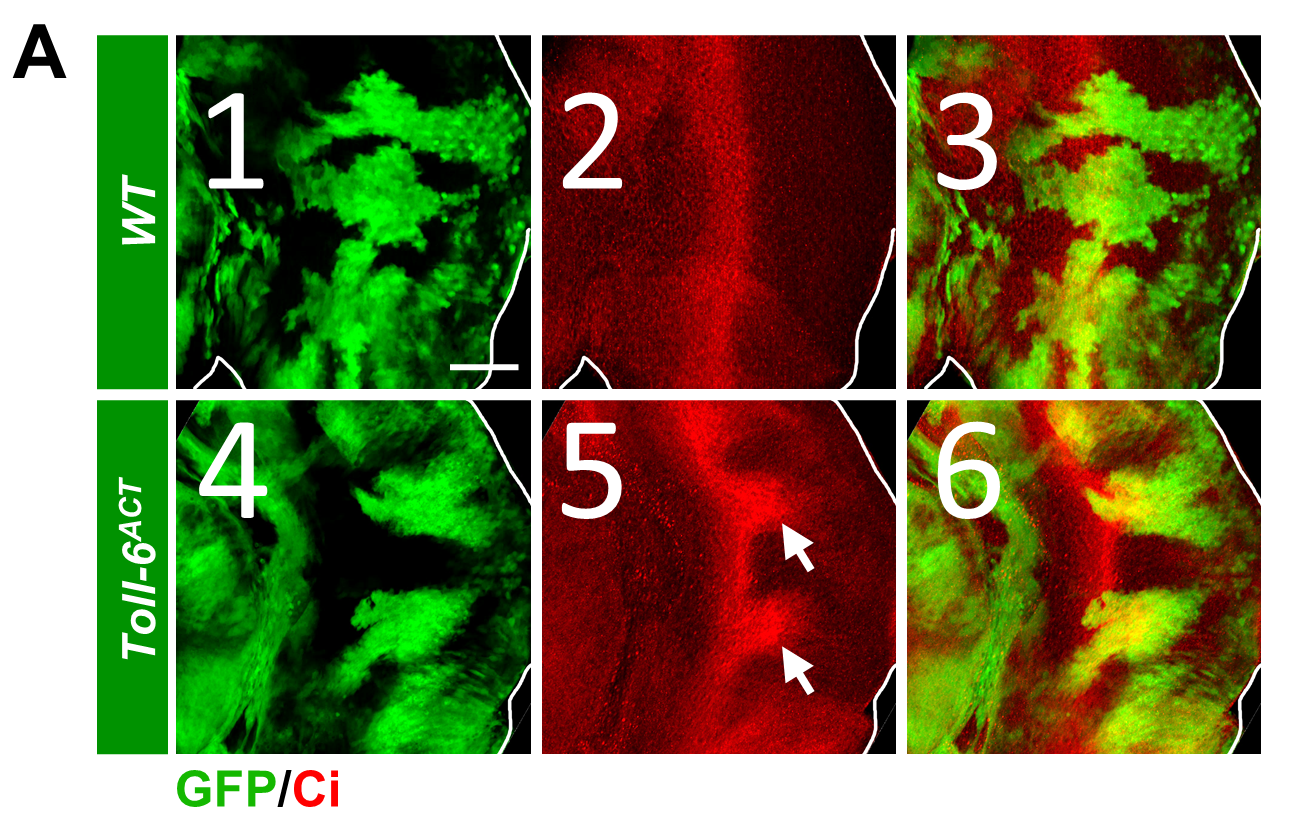

Supplement: Supplementary file 7 — Source data Fig. 3 [file 44318_2025_489_MOESM7_ESM.zip › Figure 3A/0 paper Figure 3A with provided image sequence.tif]

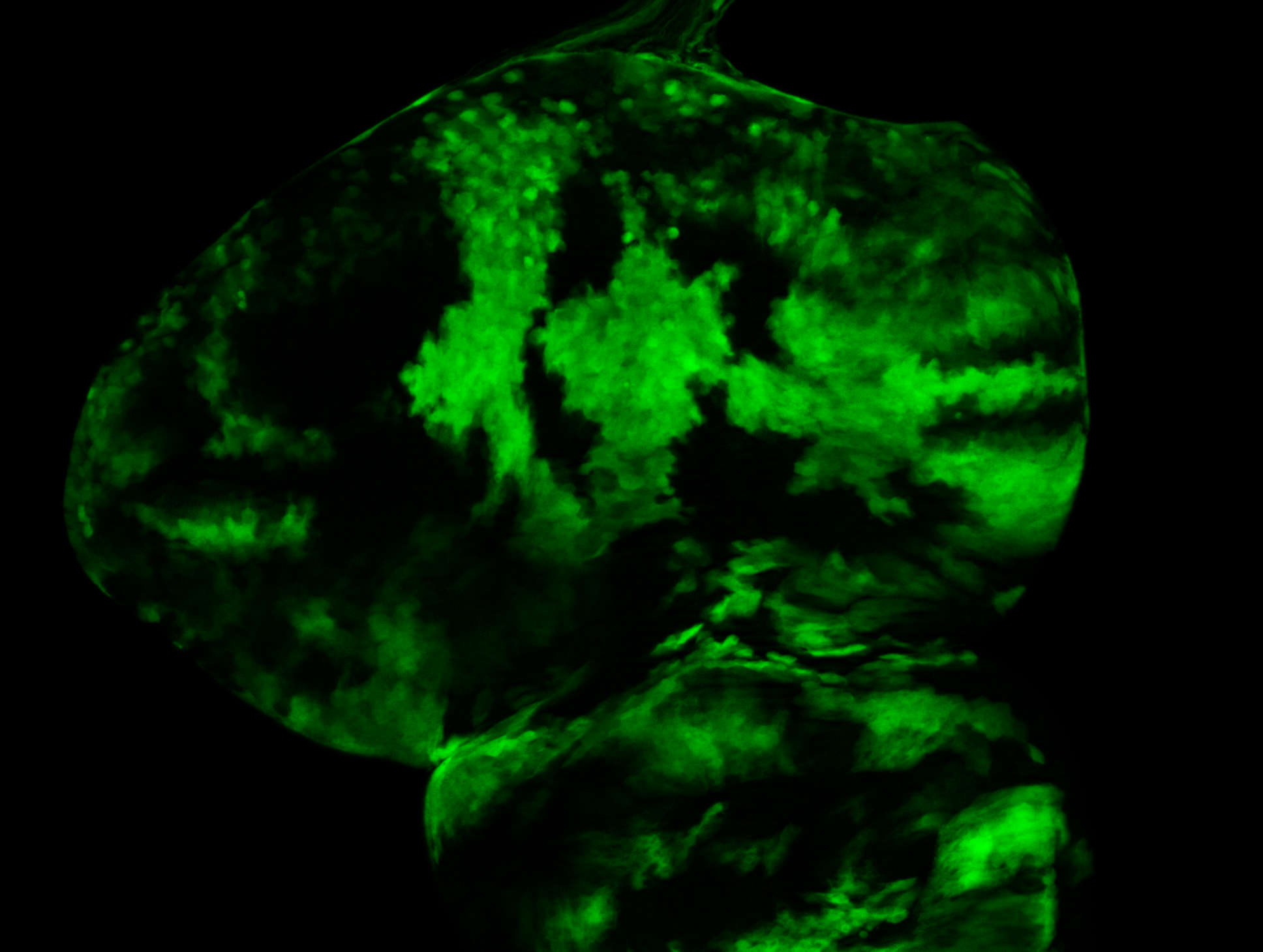

Supplement: Supplementary file 7 — Source data Fig. 3 [file 44318_2025_489_MOESM7_ESM.zip › Figure 3A/1 original image.tif]

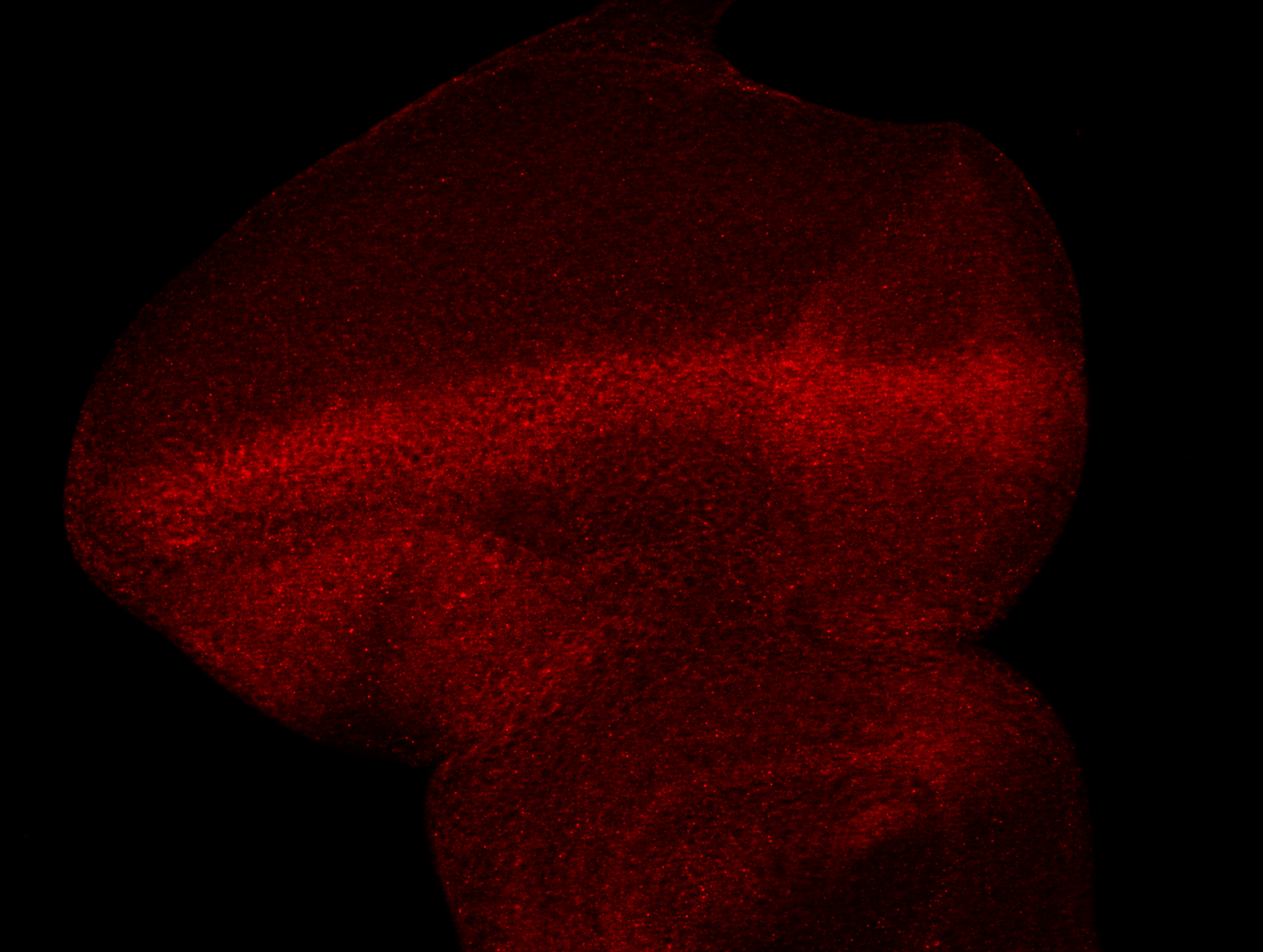

Supplement: Supplementary file 7 — Source data Fig. 3 [file 44318_2025_489_MOESM7_ESM.zip › Figure 3A/2 original image.tif]

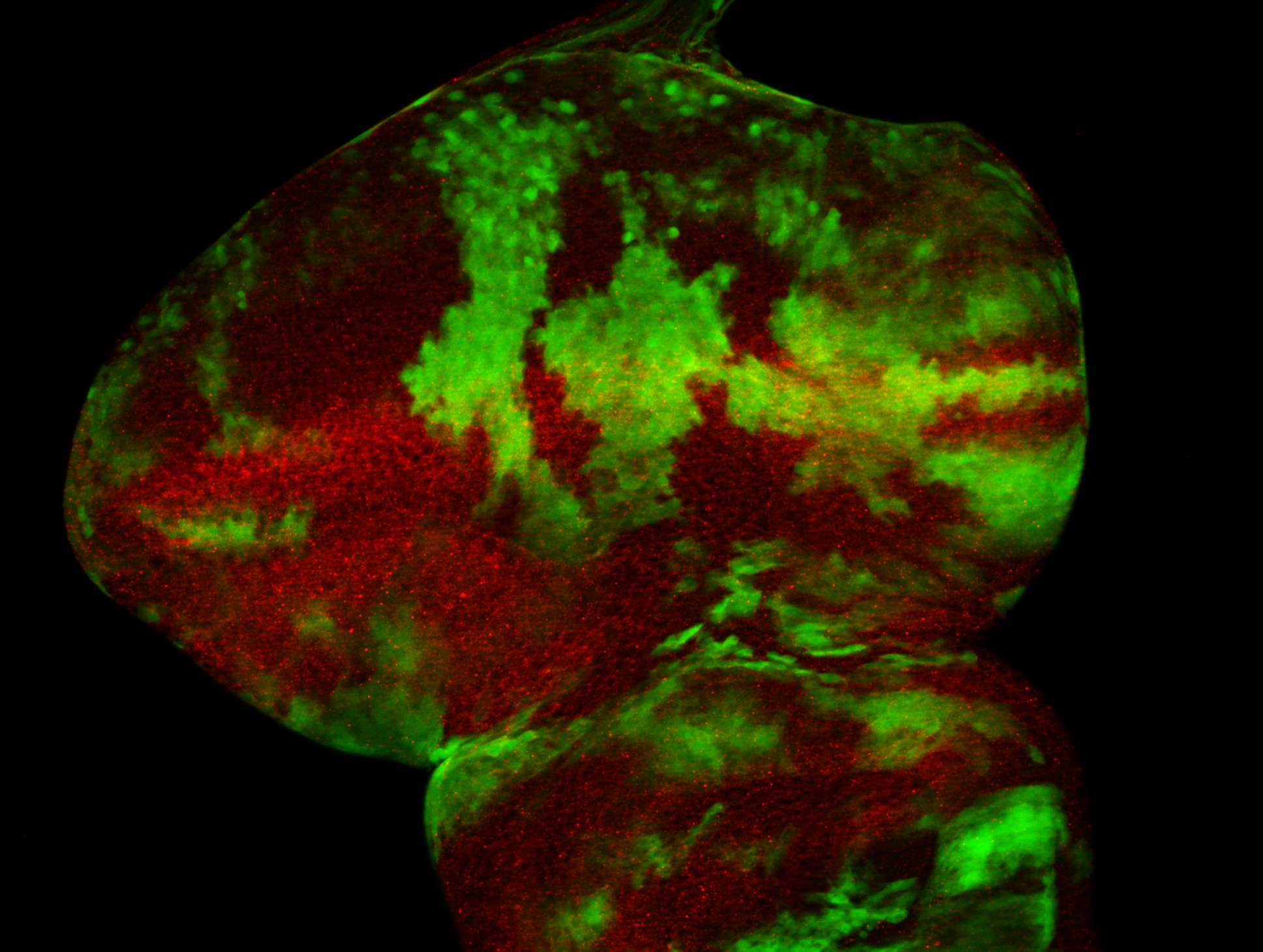

Supplement: Supplementary file 7 — Source data Fig. 3 [file 44318_2025_489_MOESM7_ESM.zip › Figure 3A/3 original image.tif]

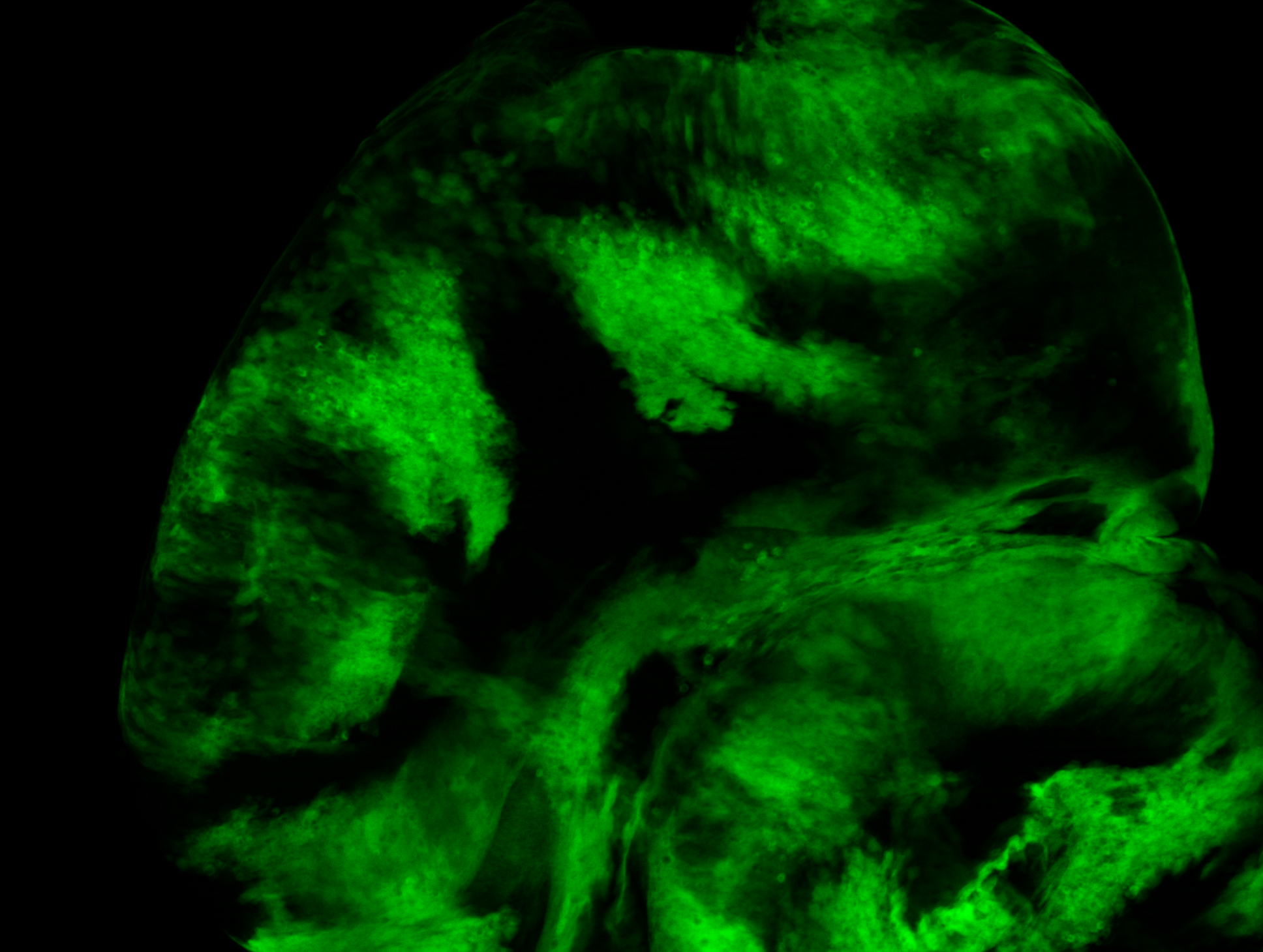

Supplement: Supplementary file 7 — Source data Fig. 3 [file 44318_2025_489_MOESM7_ESM.zip › Figure 3A/4 original image.tif]

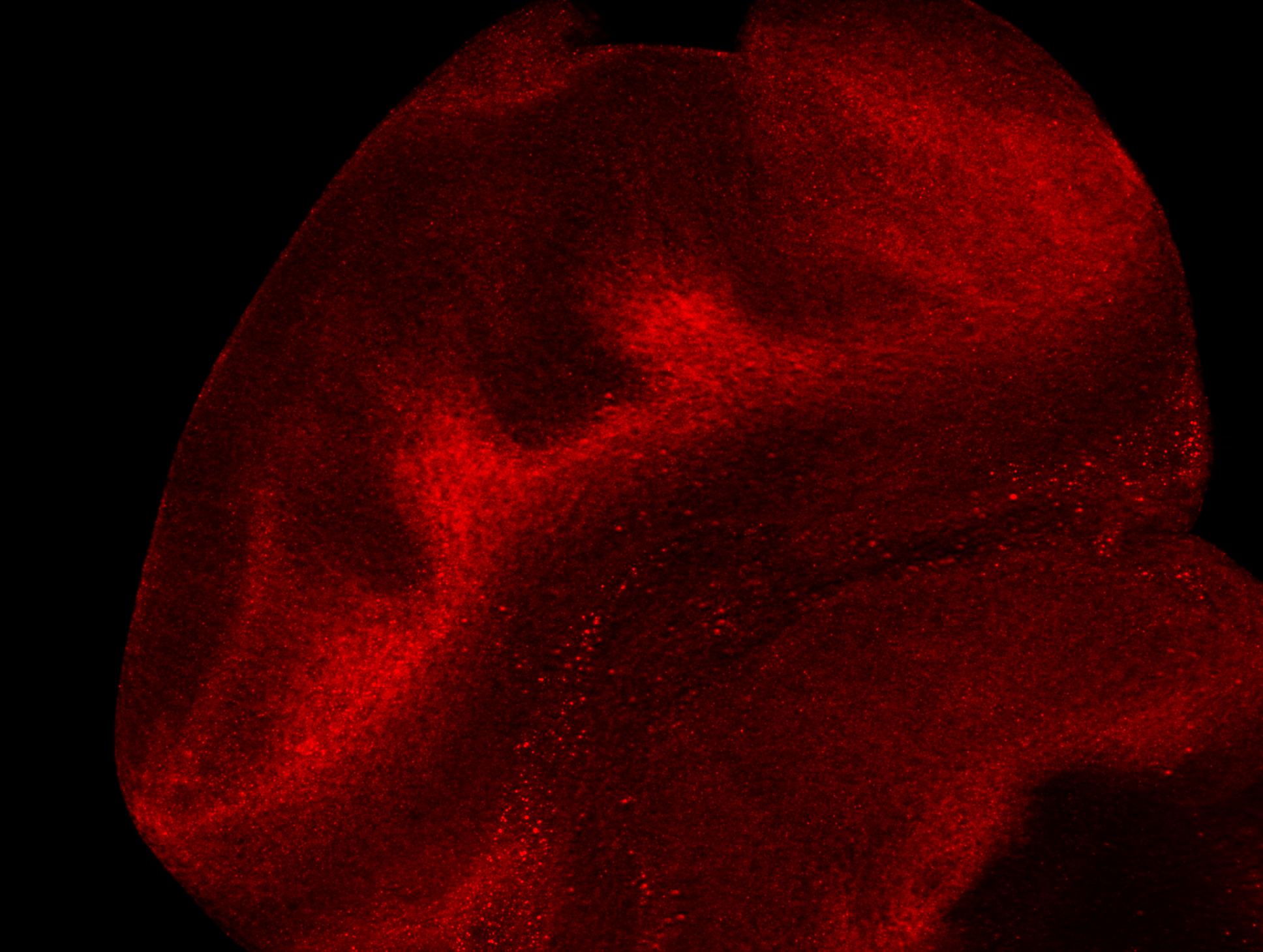

Supplement: Supplementary file 7 — Source data Fig. 3 [file 44318_2025_489_MOESM7_ESM.zip › Figure 3A/5 original image.tif]

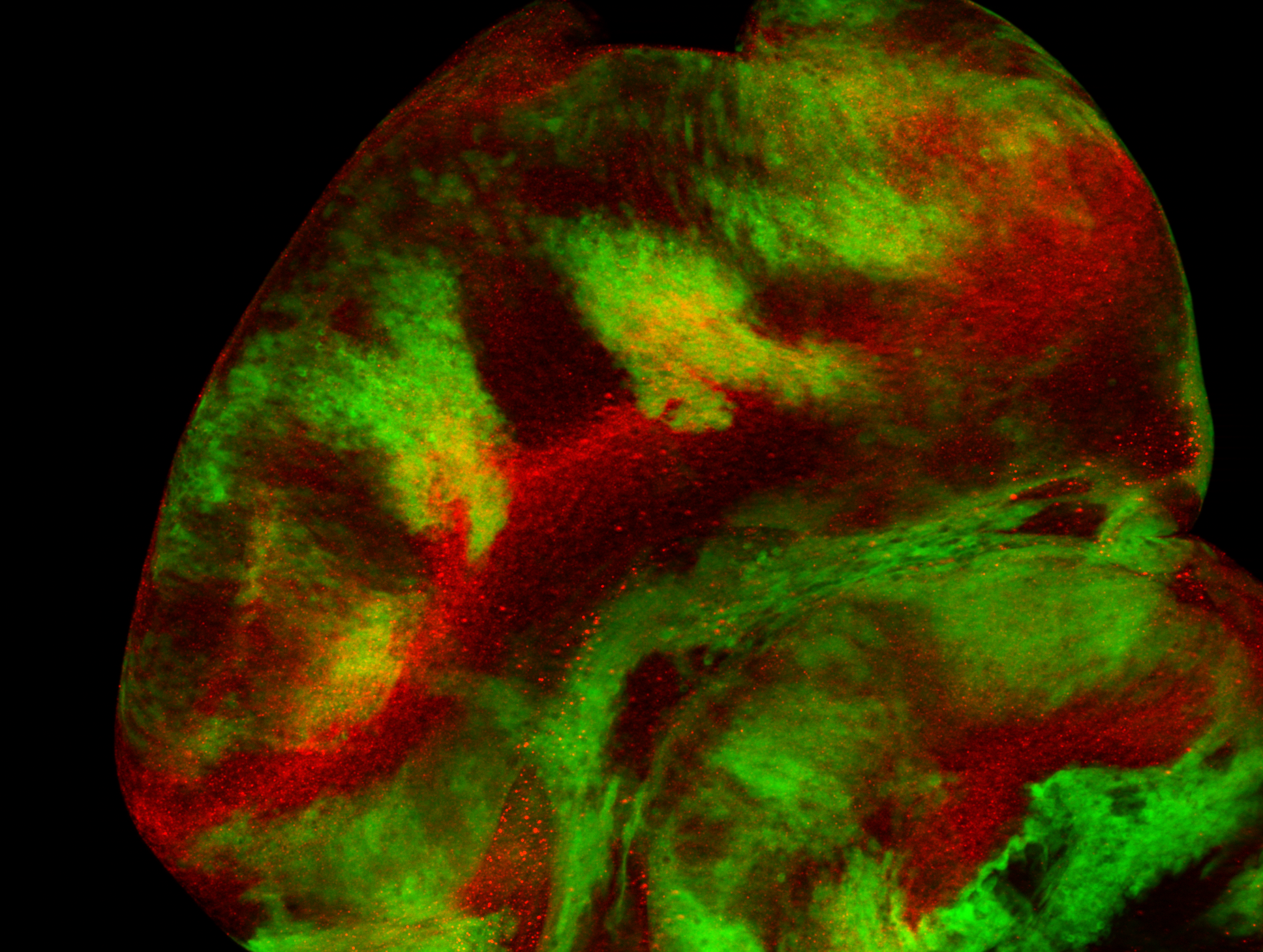

Supplement: Supplementary file 7 — Source data Fig. 3 [file 44318_2025_489_MOESM7_ESM.zip › Figure 3A/6 original image.tif]

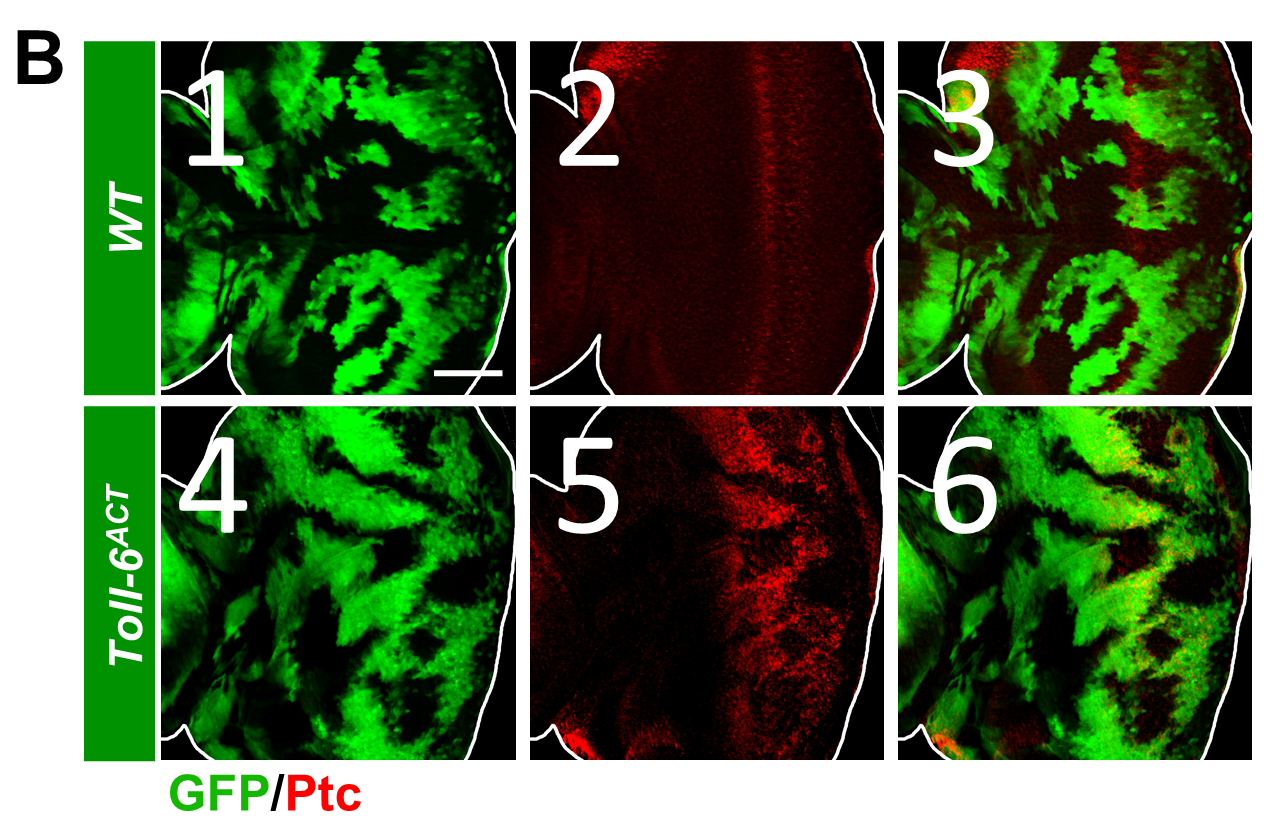

Supplement: Supplementary file 7 — Source data Fig. 3 [file 44318_2025_489_MOESM7_ESM.zip › Figure 3B/0 paper Figure 3B with provided image sequence.tif]

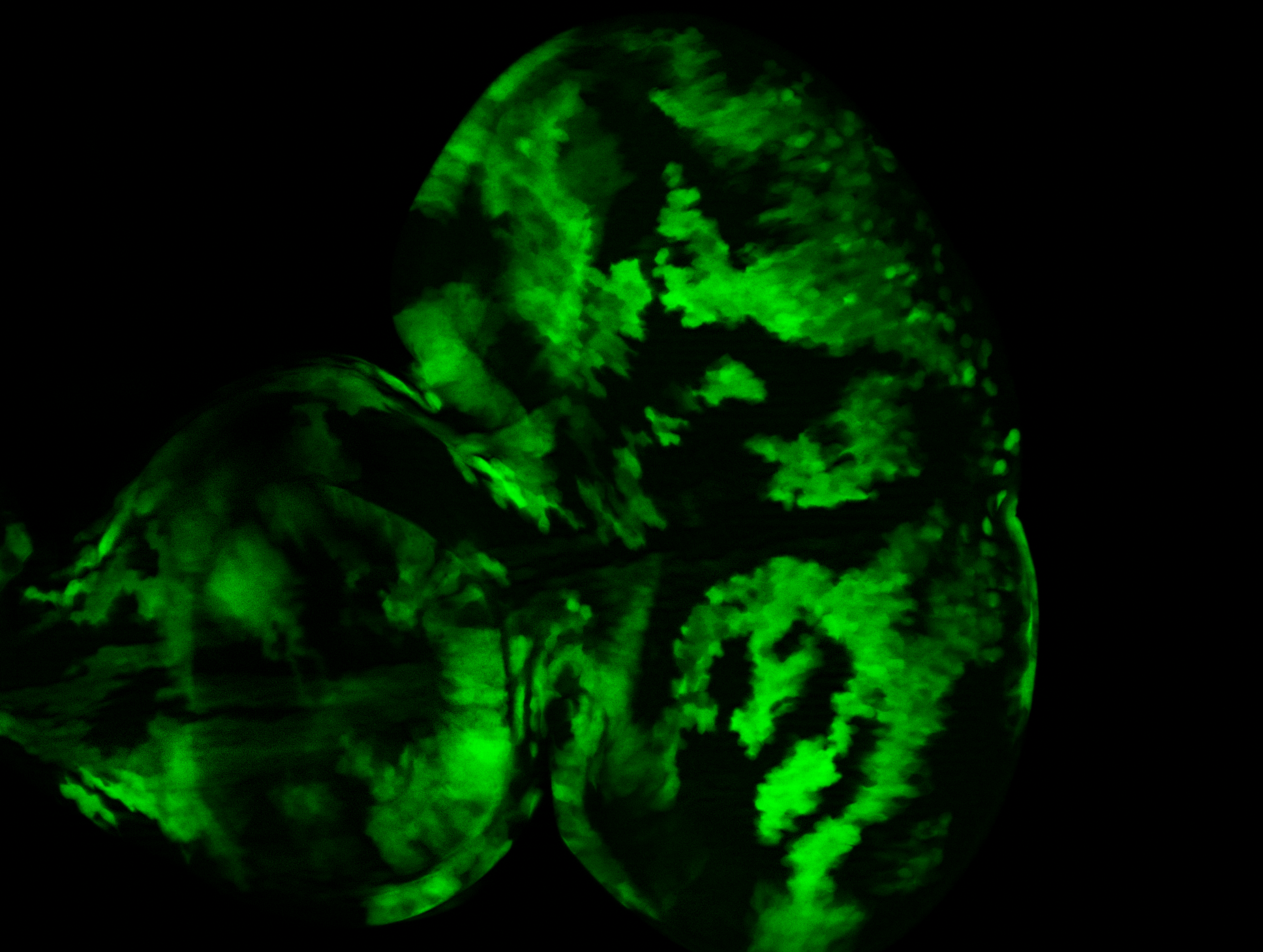

Supplement: Supplementary file 7 — Source data Fig. 3 [file 44318_2025_489_MOESM7_ESM.zip › Figure 3B/1 original image.tif]

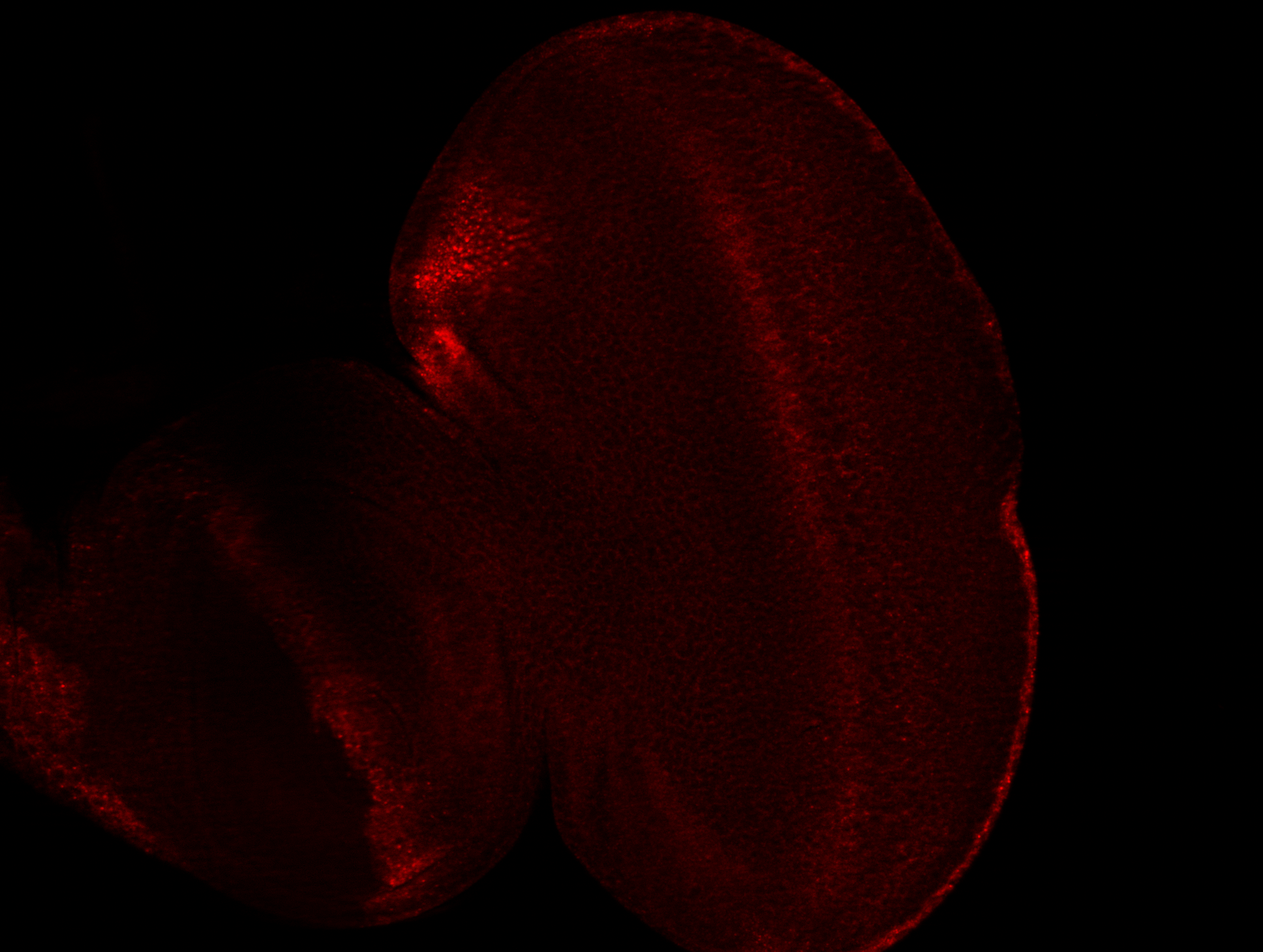

Supplement: Supplementary file 7 — Source data Fig. 3 [file 44318_2025_489_MOESM7_ESM.zip › Figure 3B/2 original image.tif]

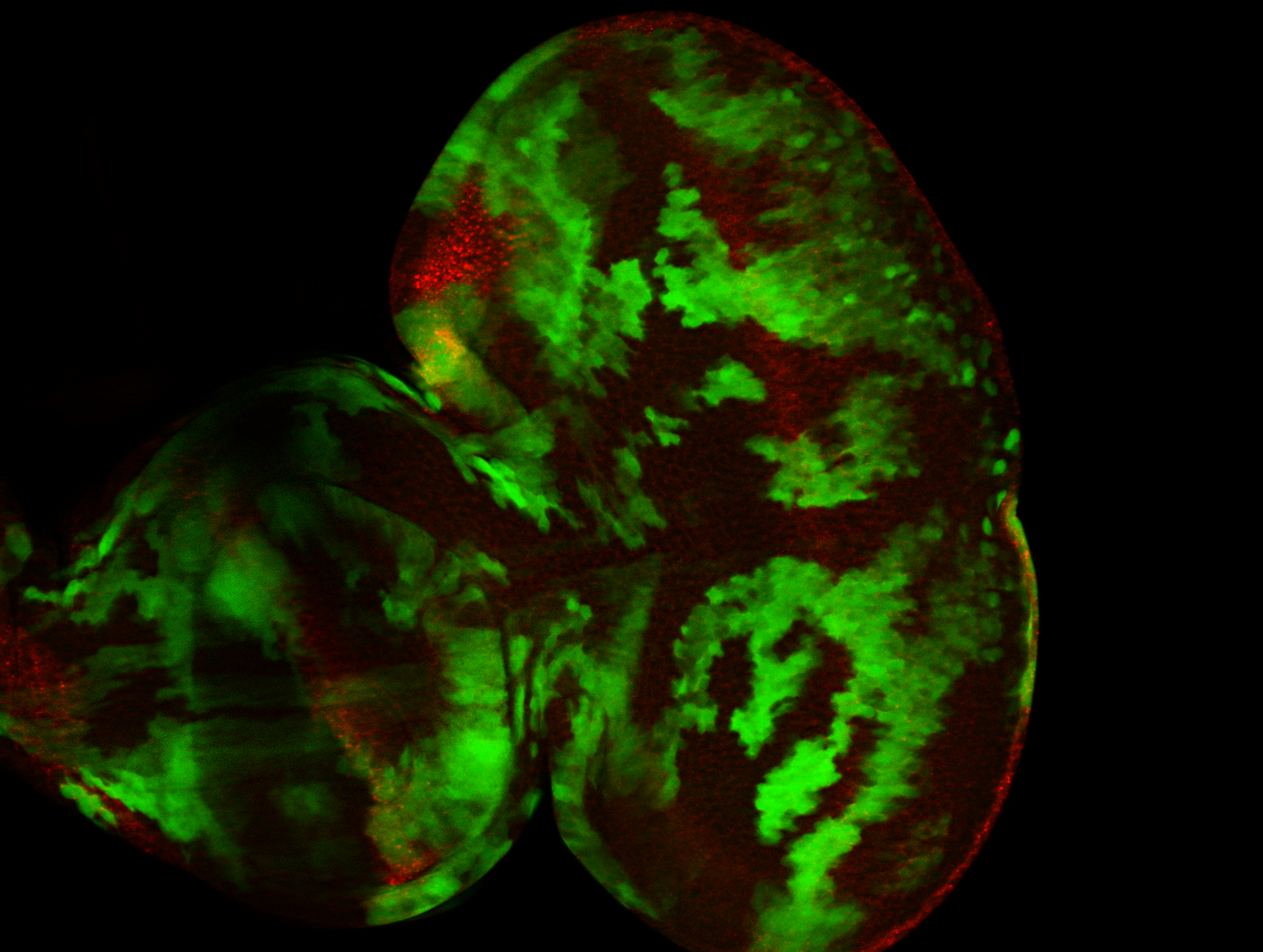

Supplement: Supplementary file 7 — Source data Fig. 3 [file 44318_2025_489_MOESM7_ESM.zip › Figure 3B/3 original image.tif]

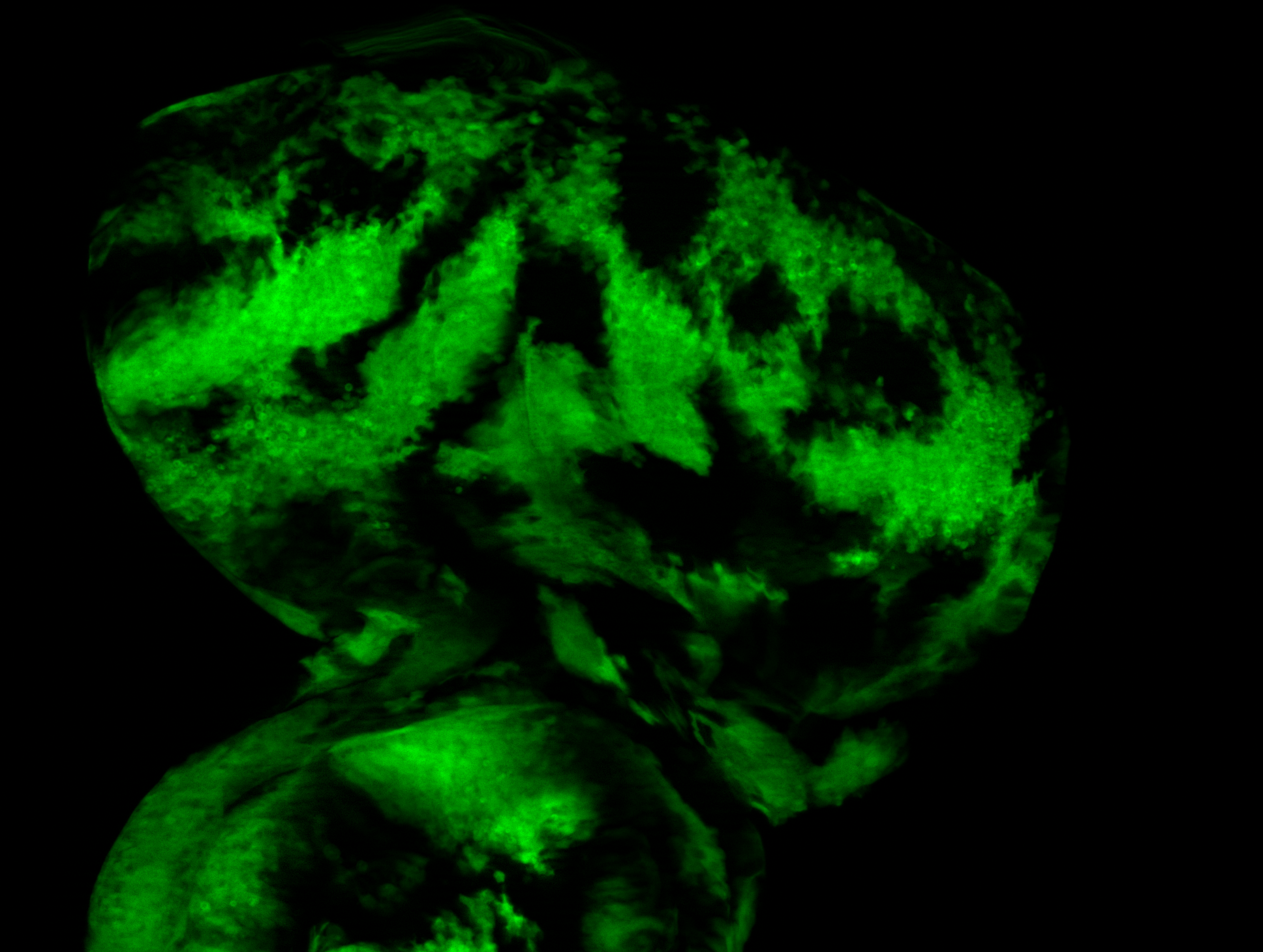

Supplement: Supplementary file 7 — Source data Fig. 3 [file 44318_2025_489_MOESM7_ESM.zip › Figure 3B/4 original image.tif]

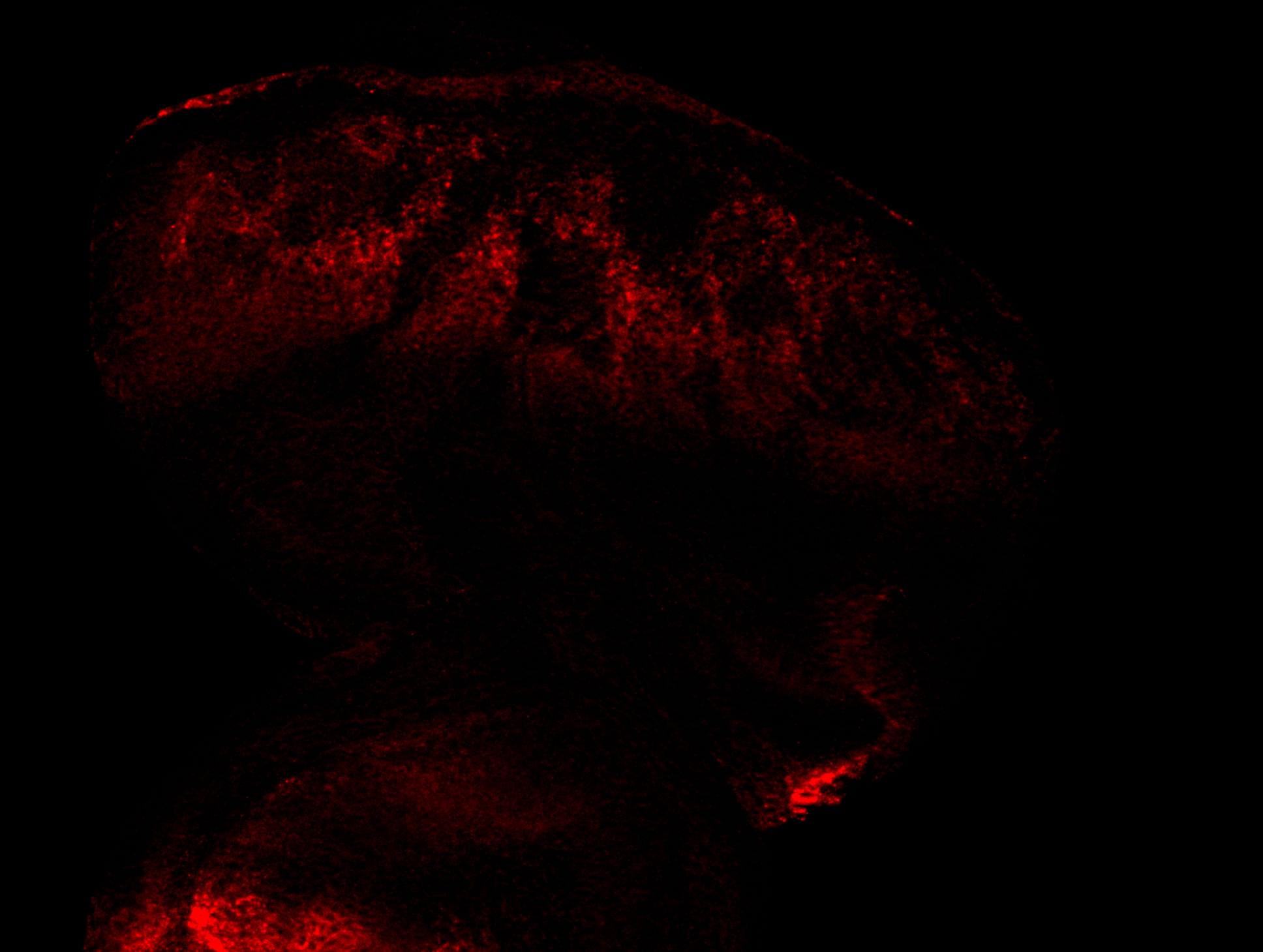

Supplement: Supplementary file 7 — Source data Fig. 3 [file 44318_2025_489_MOESM7_ESM.zip › Figure 3B/5 original image.tif]

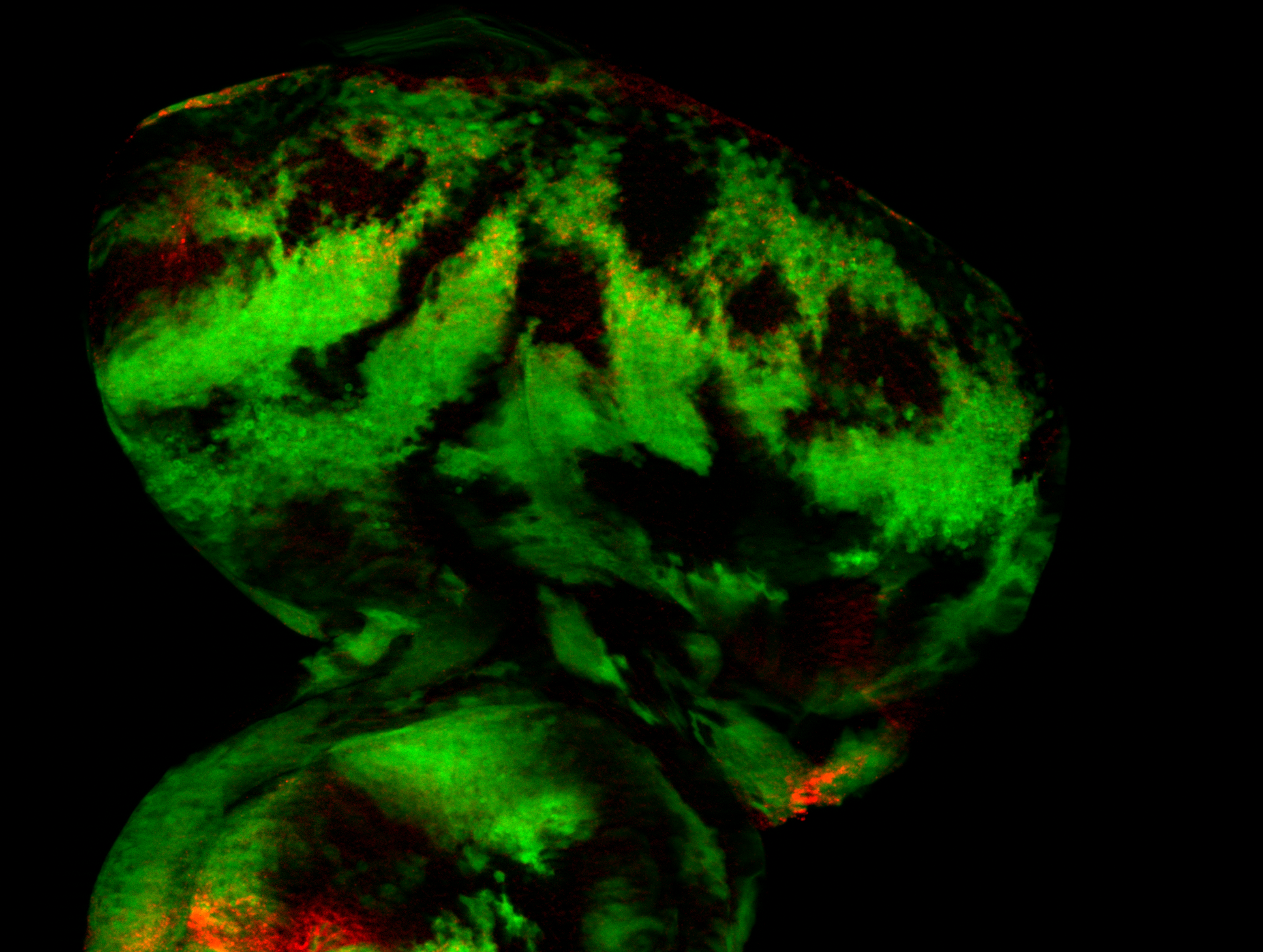

Supplement: Supplementary file 7 — Source data Fig. 3 [file 44318_2025_489_MOESM7_ESM.zip › Figure 3B/6 original image.tif]

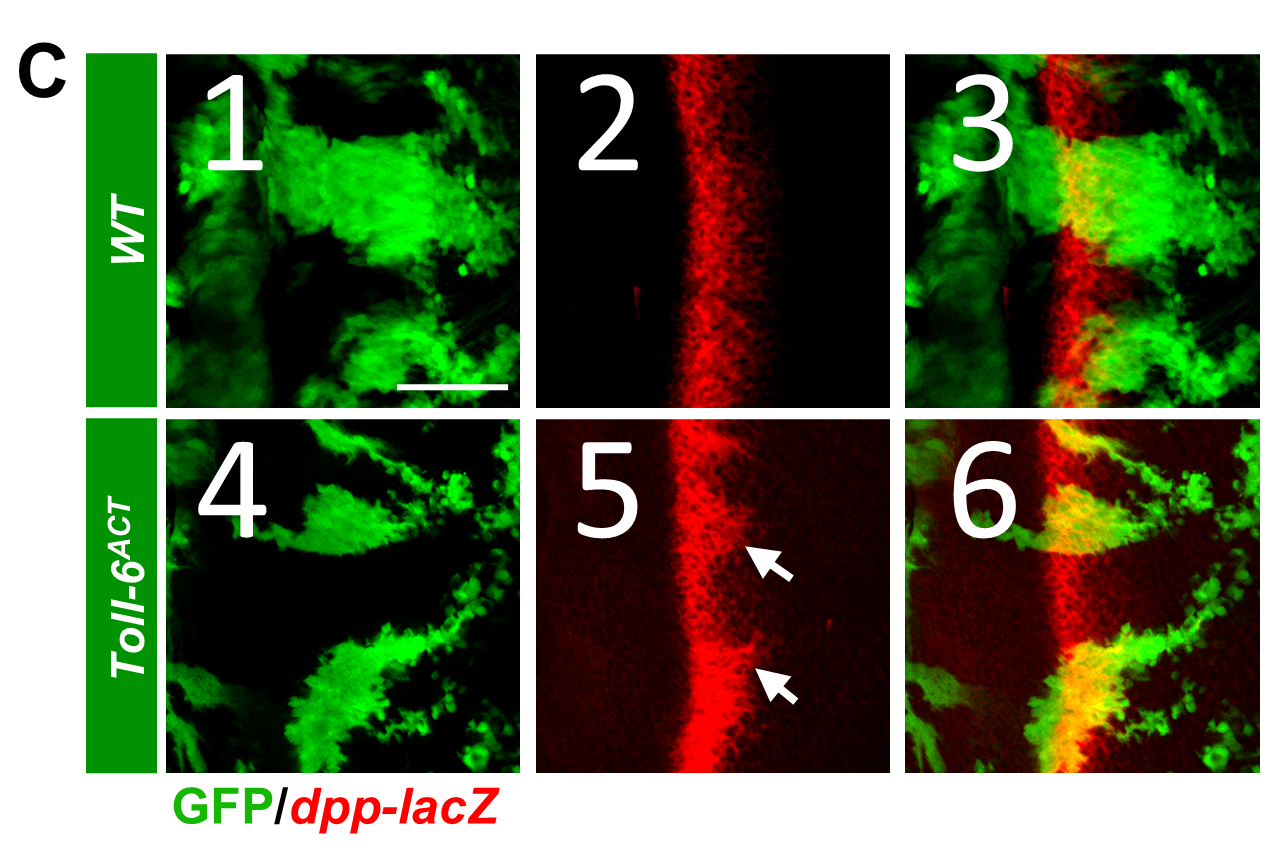

Supplement: Supplementary file 7 — Source data Fig. 3 [file 44318_2025_489_MOESM7_ESM.zip › Figure 3C/0 paper Figure 3C with provided image sequence.tif]

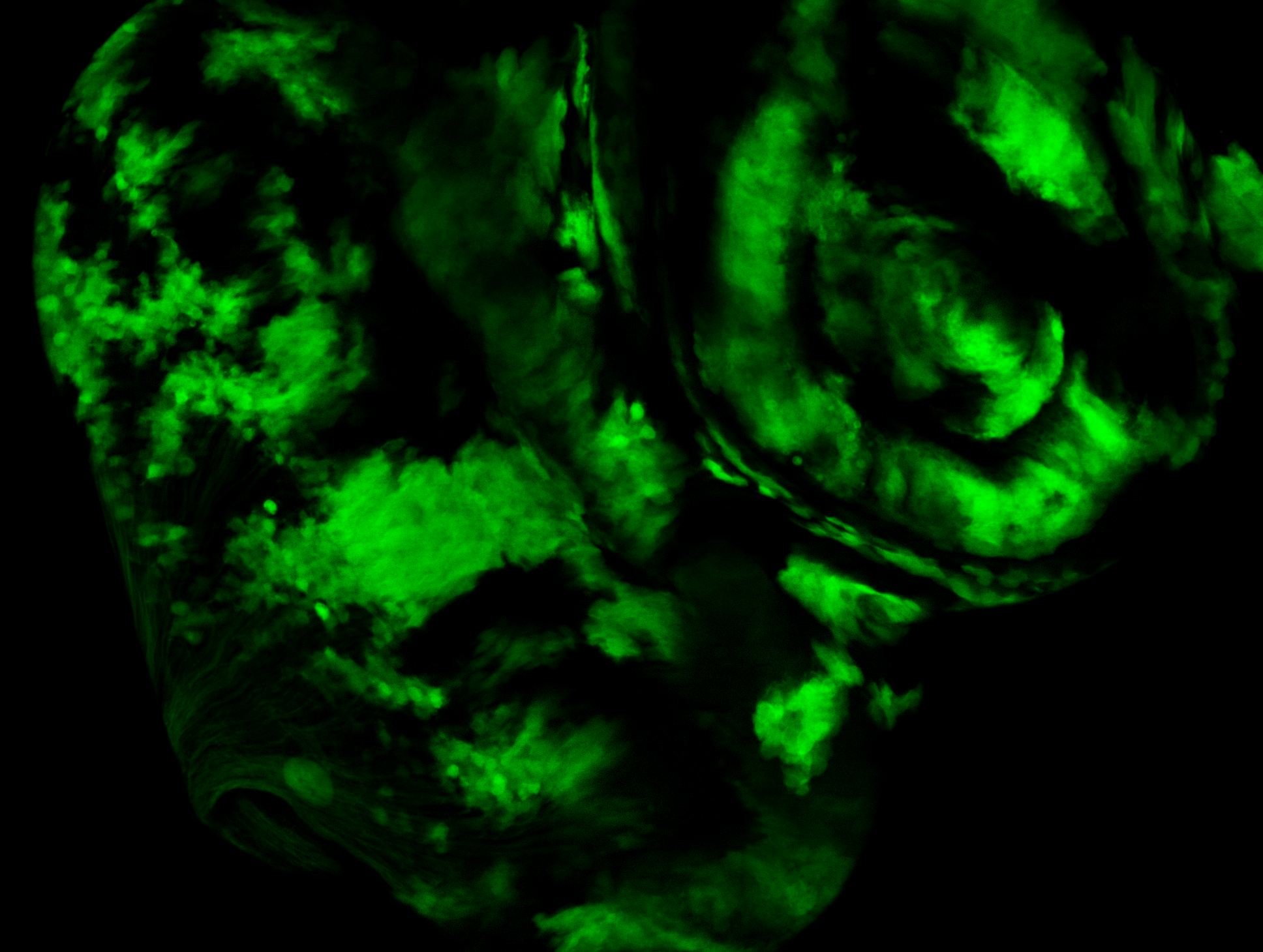

Supplement: Supplementary file 7 — Source data Fig. 3 [file 44318_2025_489_MOESM7_ESM.zip › Figure 3C/1 original image.tif]

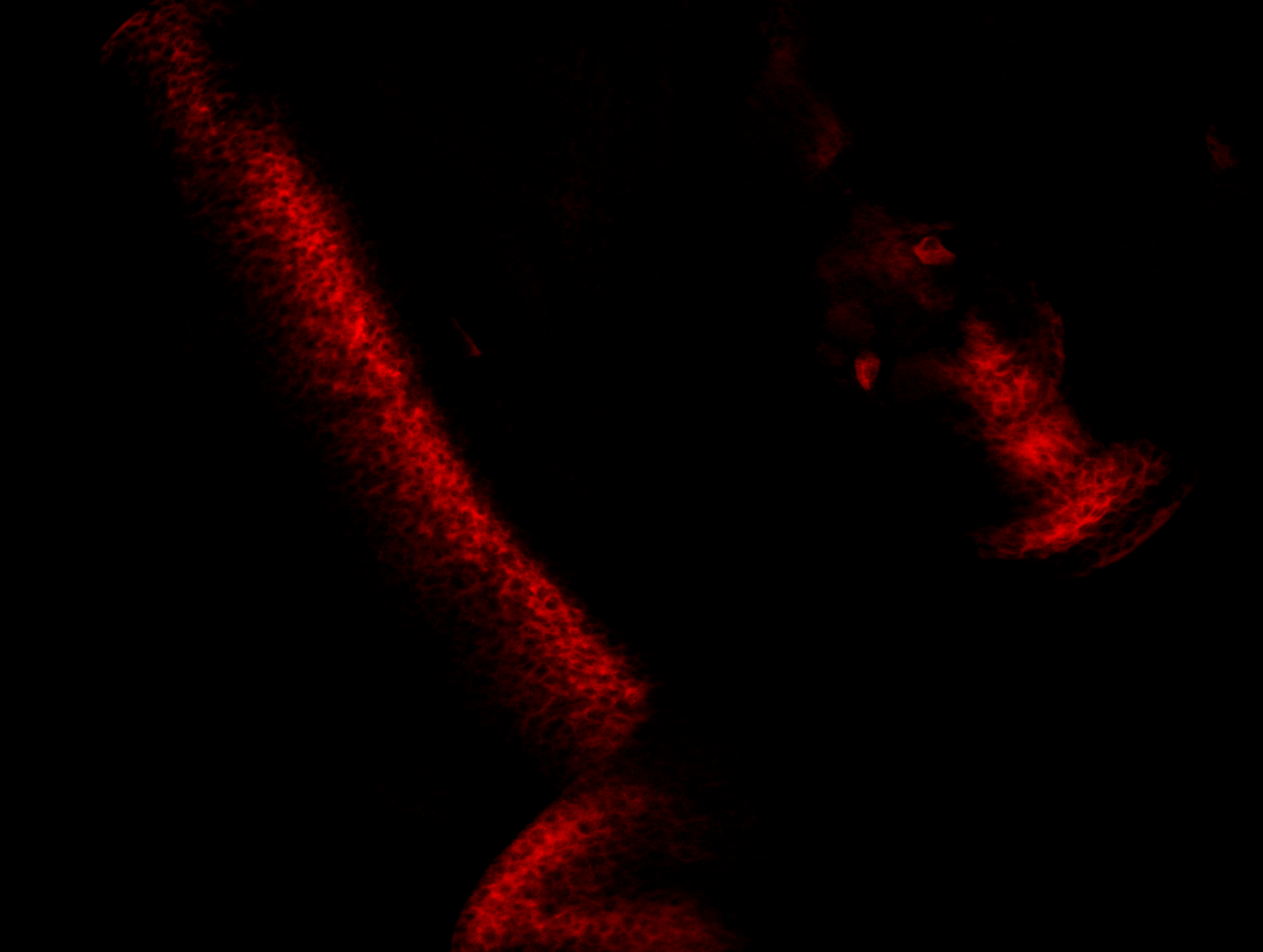

Supplement: Supplementary file 7 — Source data Fig. 3 [file 44318_2025_489_MOESM7_ESM.zip › Figure 3C/2 original image.tif]

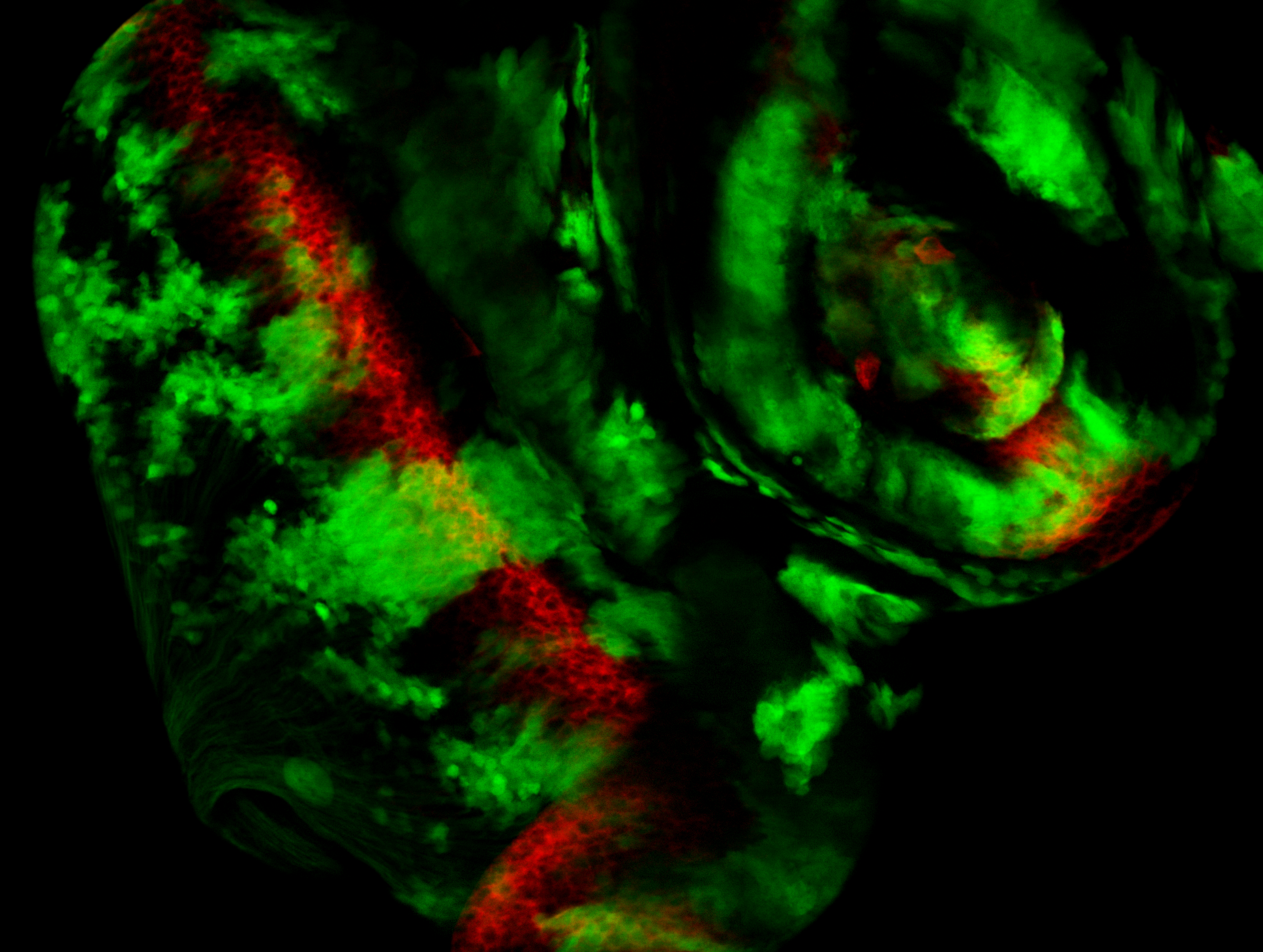

Supplement: Supplementary file 7 — Source data Fig. 3 [file 44318_2025_489_MOESM7_ESM.zip › Figure 3C/3 original image.tif]

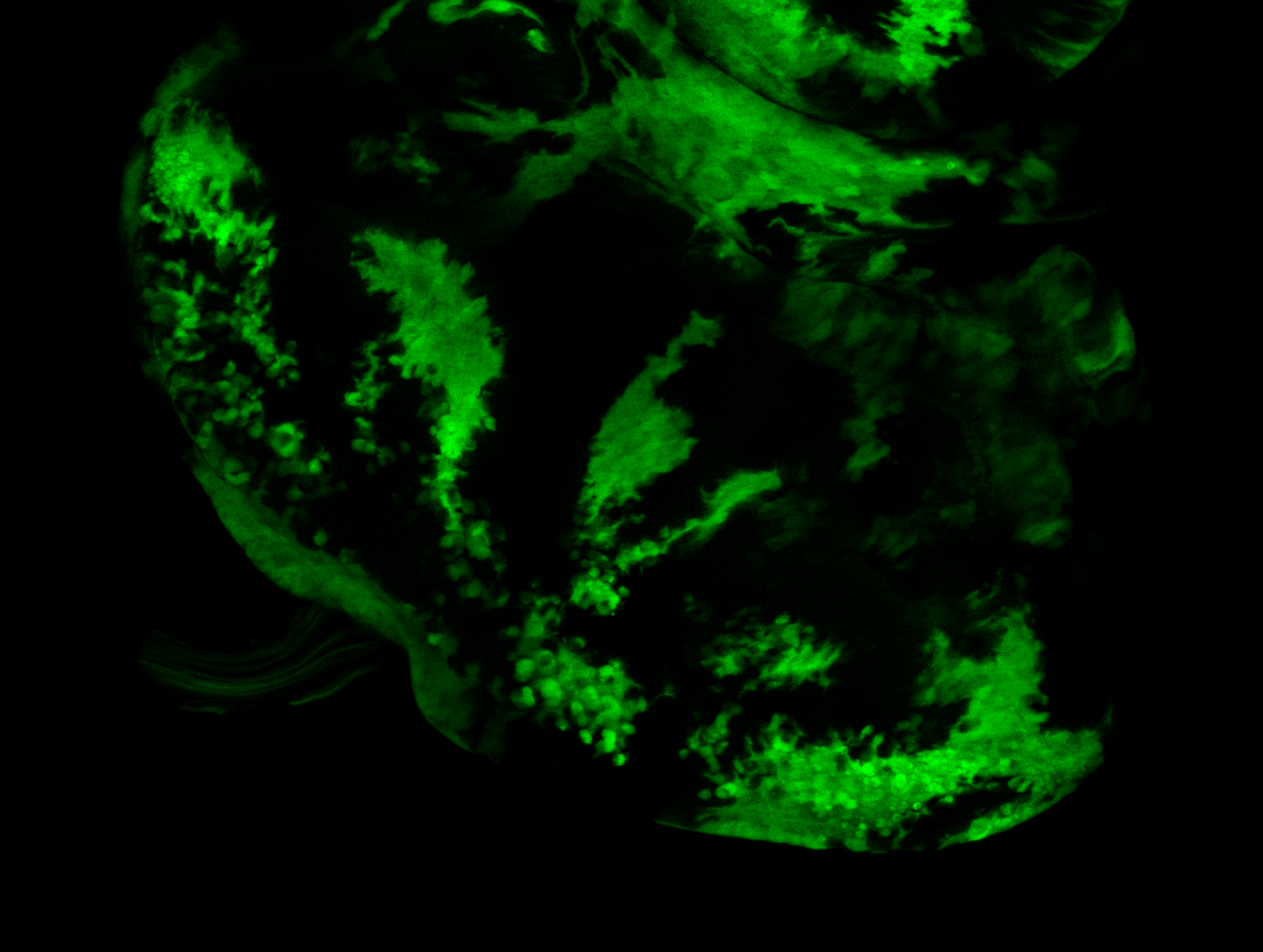

Supplement: Supplementary file 7 — Source data Fig. 3 [file 44318_2025_489_MOESM7_ESM.zip › Figure 3C/4 original image.tif]

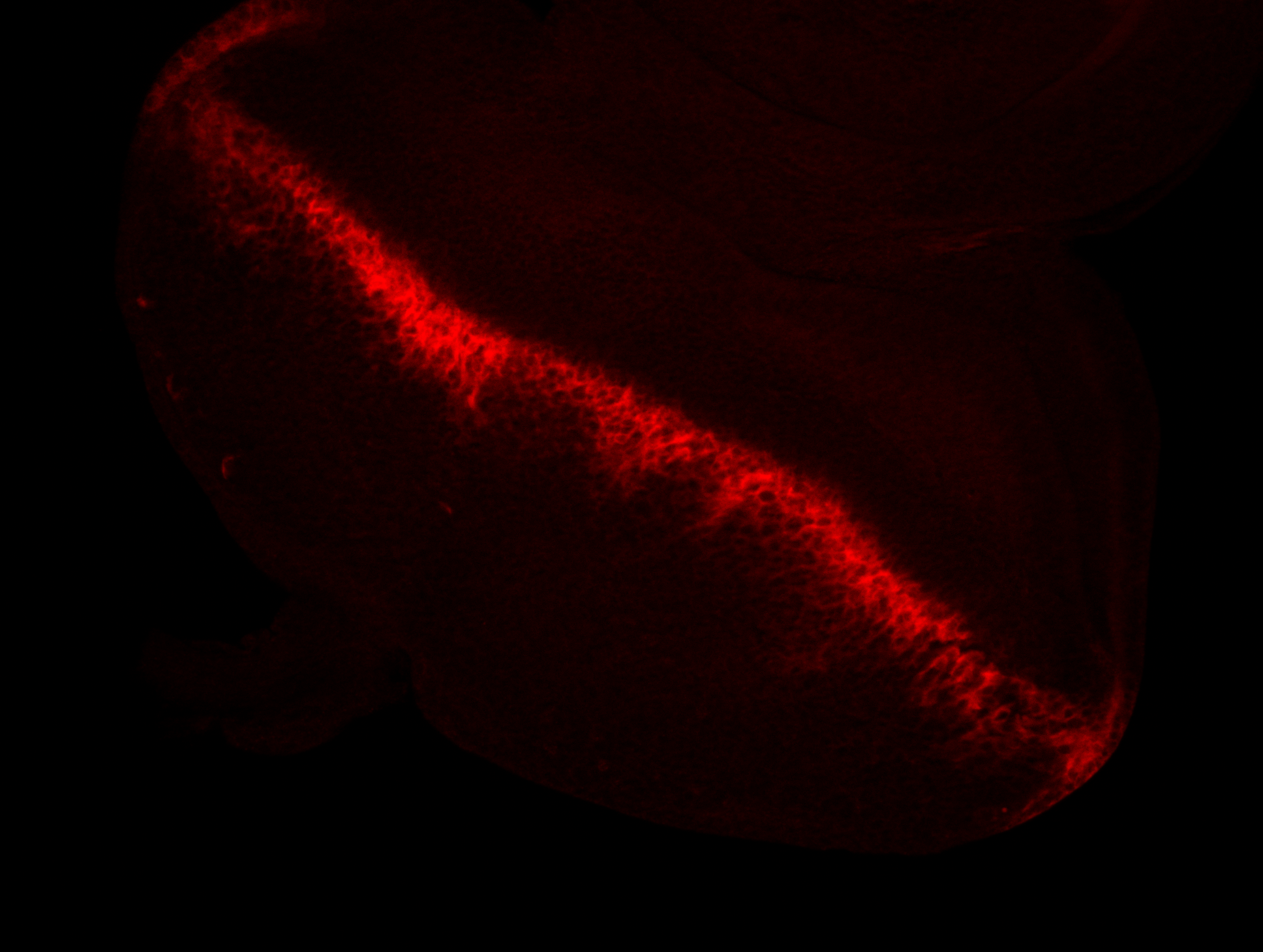

Supplement: Supplementary file 7 — Source data Fig. 3 [file 44318_2025_489_MOESM7_ESM.zip › Figure 3C/5 original image.tif]

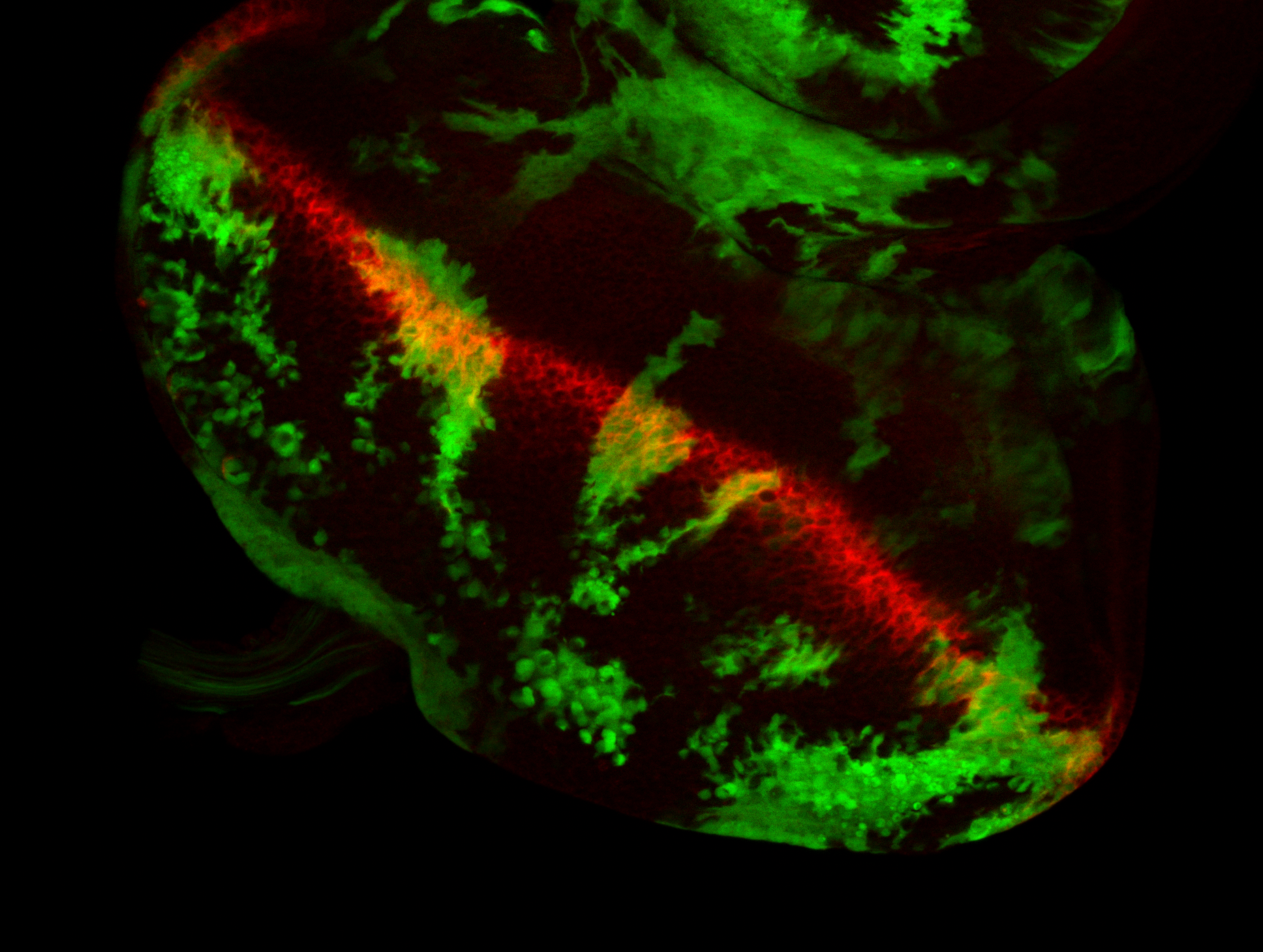

Supplement: Supplementary file 7 — Source data Fig. 3 [file 44318_2025_489_MOESM7_ESM.zip › Figure 3C/6 original image.tif]

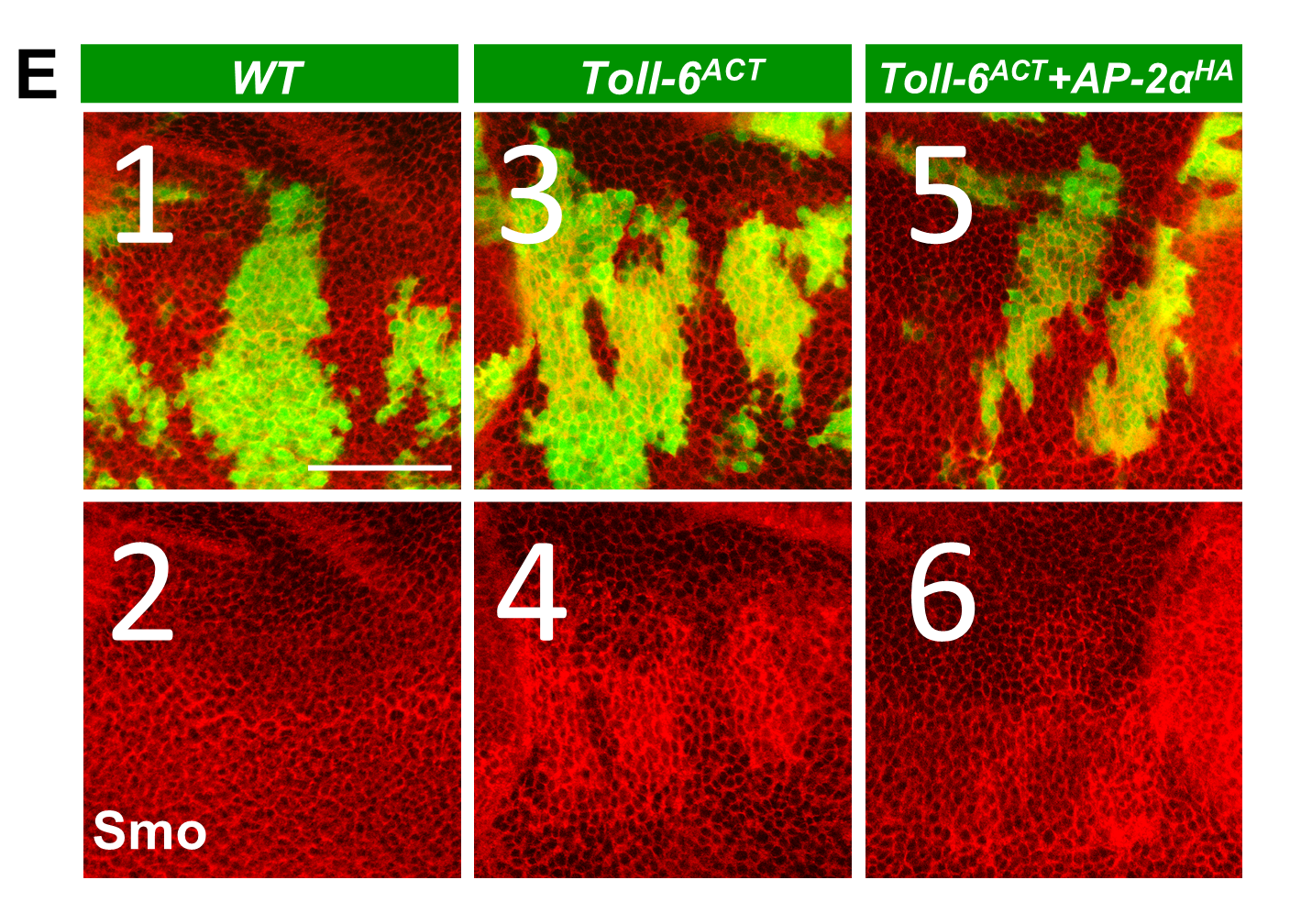

Supplement: Supplementary file 8 — Source data Fig. 4 [file 44318_2025_489_MOESM8_ESM.zip › Figure 4E/0 paper Figure 4E with provided image sequence.tif]

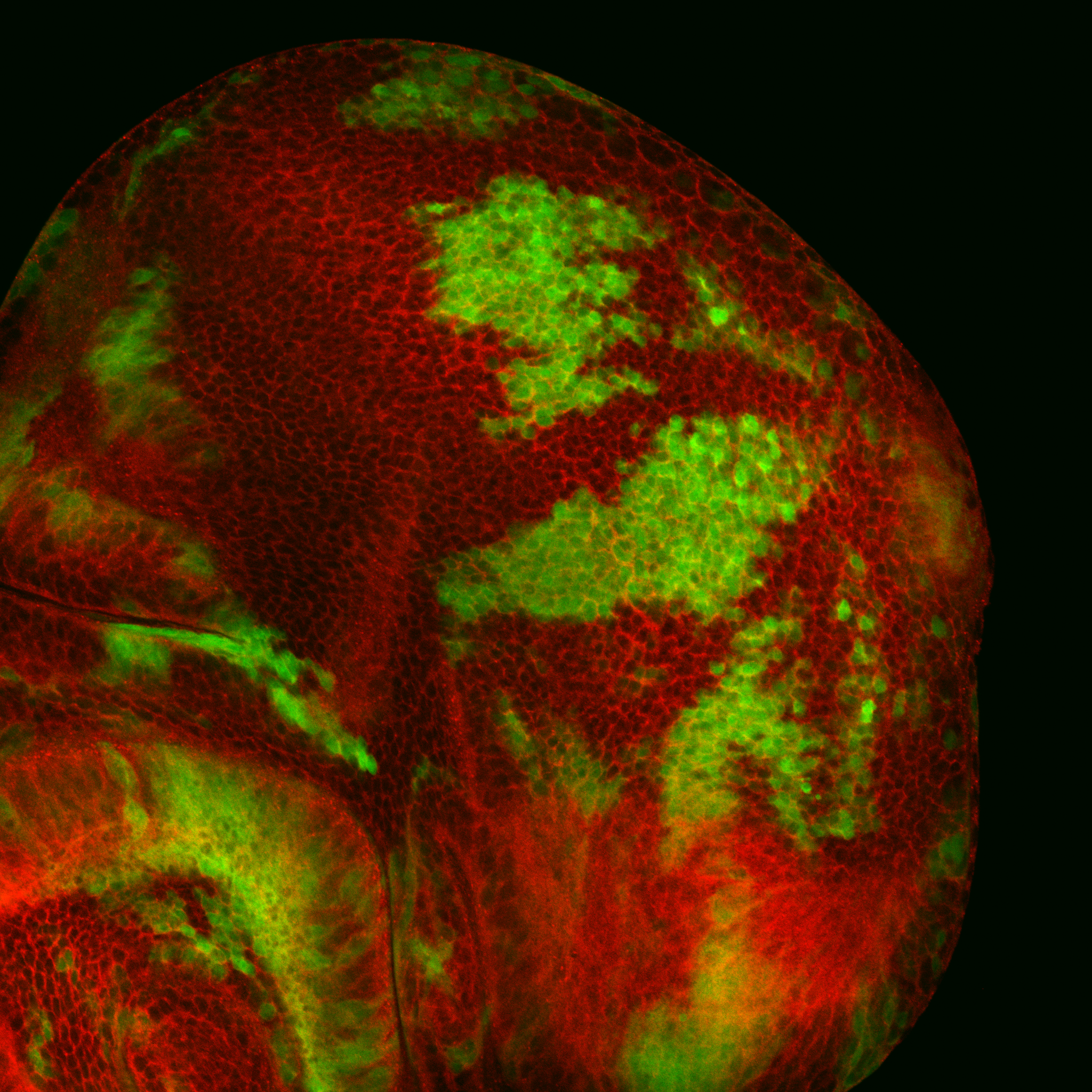

Supplement: Supplementary file 8 — Source data Fig. 4 [file 44318_2025_489_MOESM8_ESM.zip › Figure 4E/1 original image.tif]

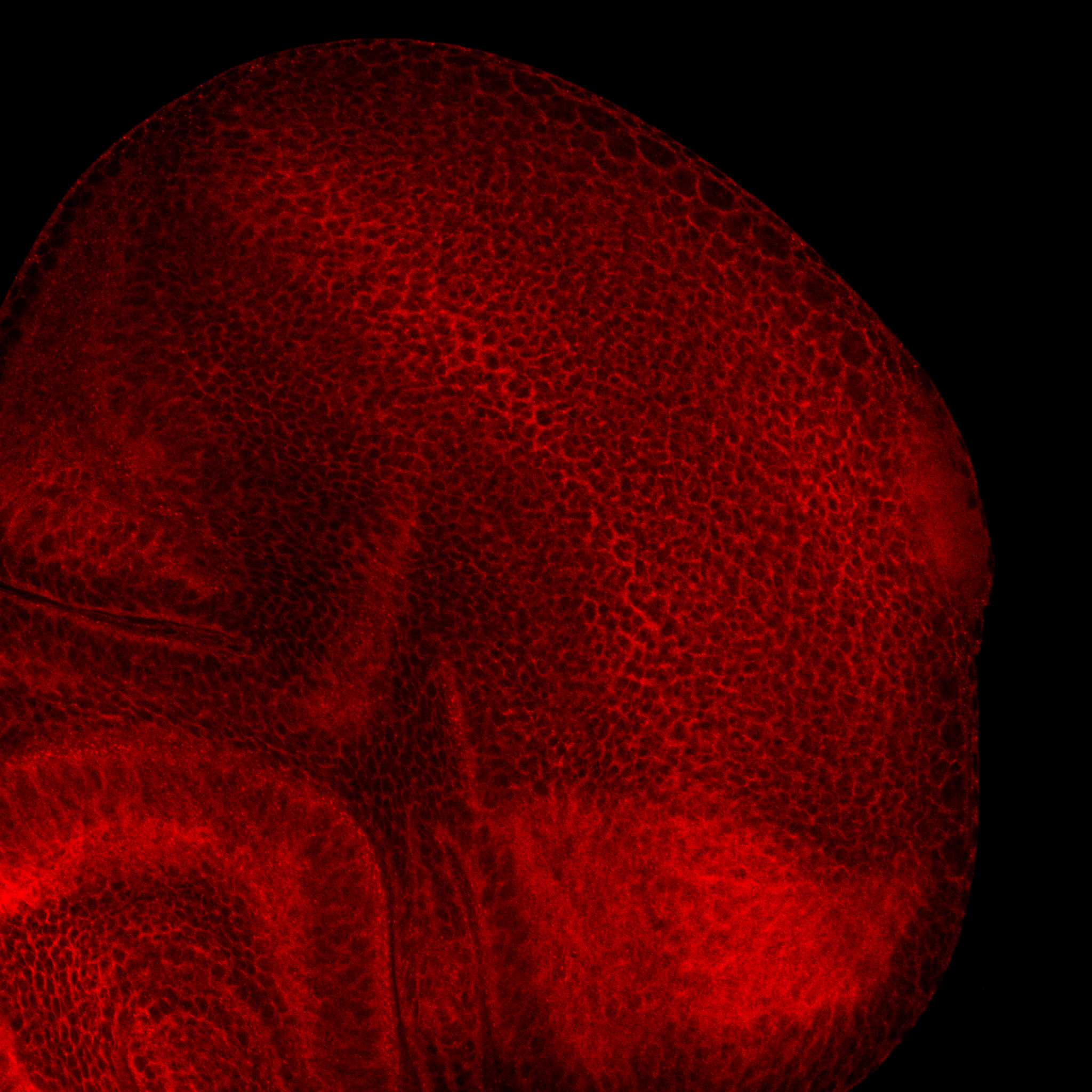

Supplement: Supplementary file 8 — Source data Fig. 4 [file 44318_2025_489_MOESM8_ESM.zip › Figure 4E/2 original image.tif]

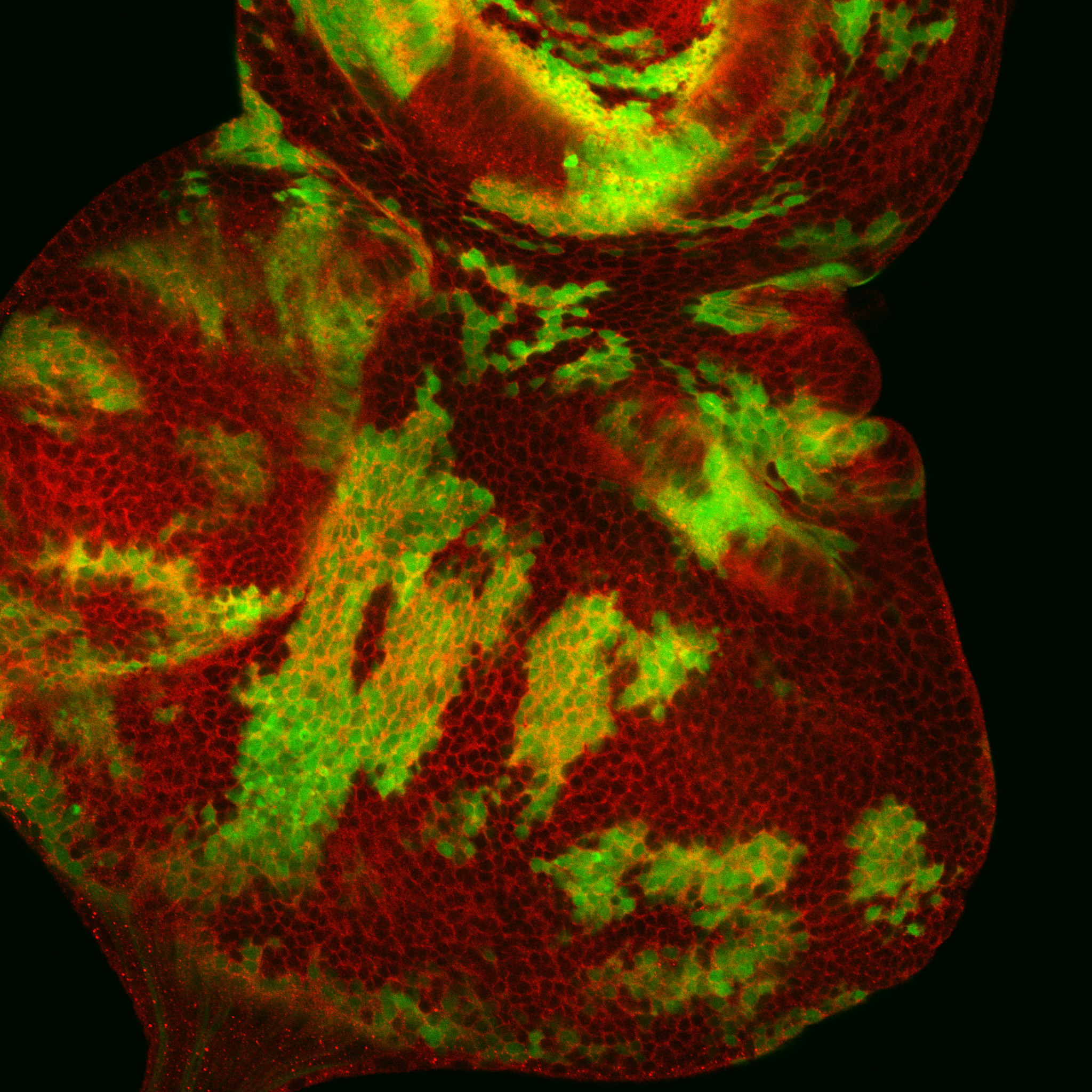

Supplement: Supplementary file 8 — Source data Fig. 4 [file 44318_2025_489_MOESM8_ESM.zip › Figure 4E/3 original image.tif]

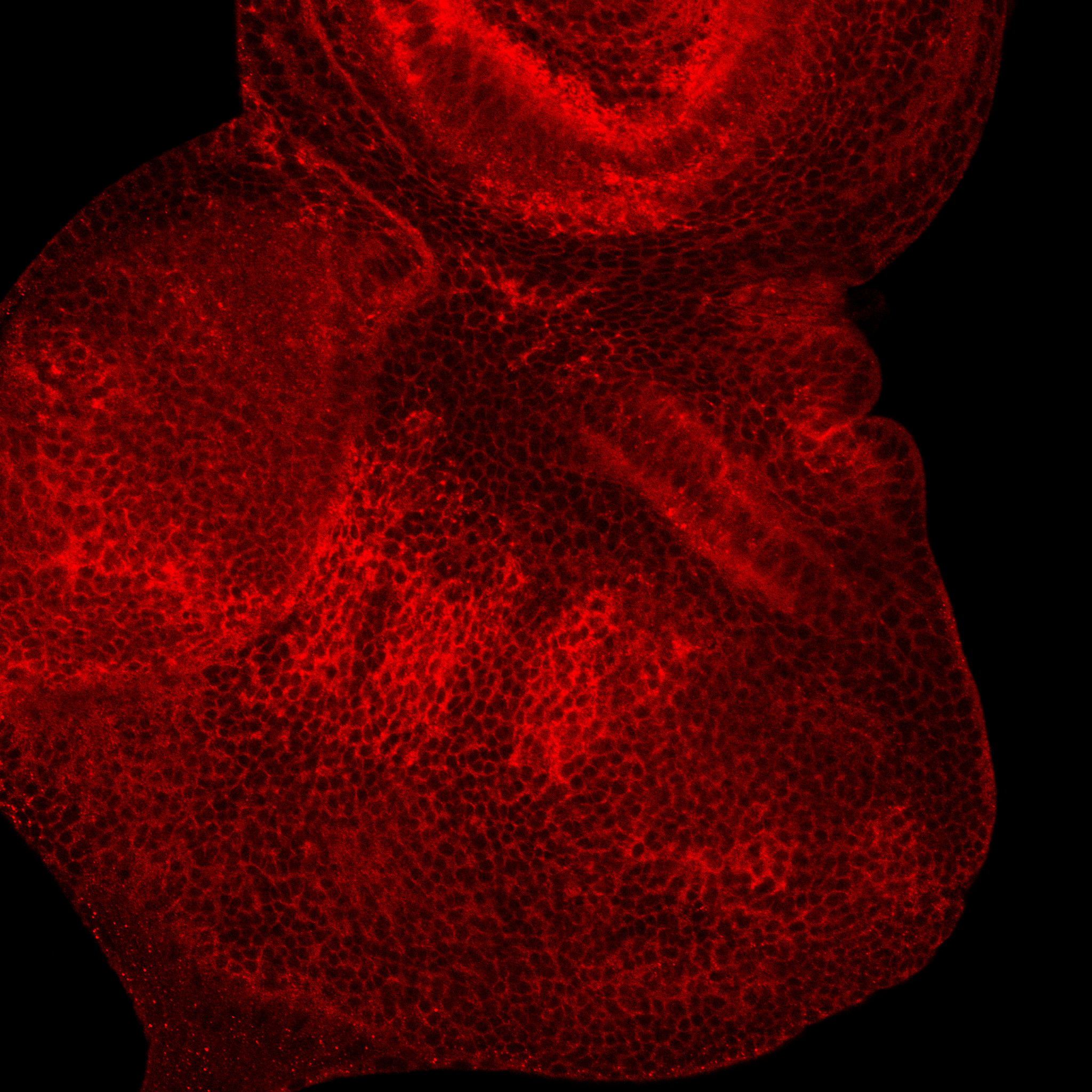

Supplement: Supplementary file 8 — Source data Fig. 4 [file 44318_2025_489_MOESM8_ESM.zip › Figure 4E/4 original image.tif]

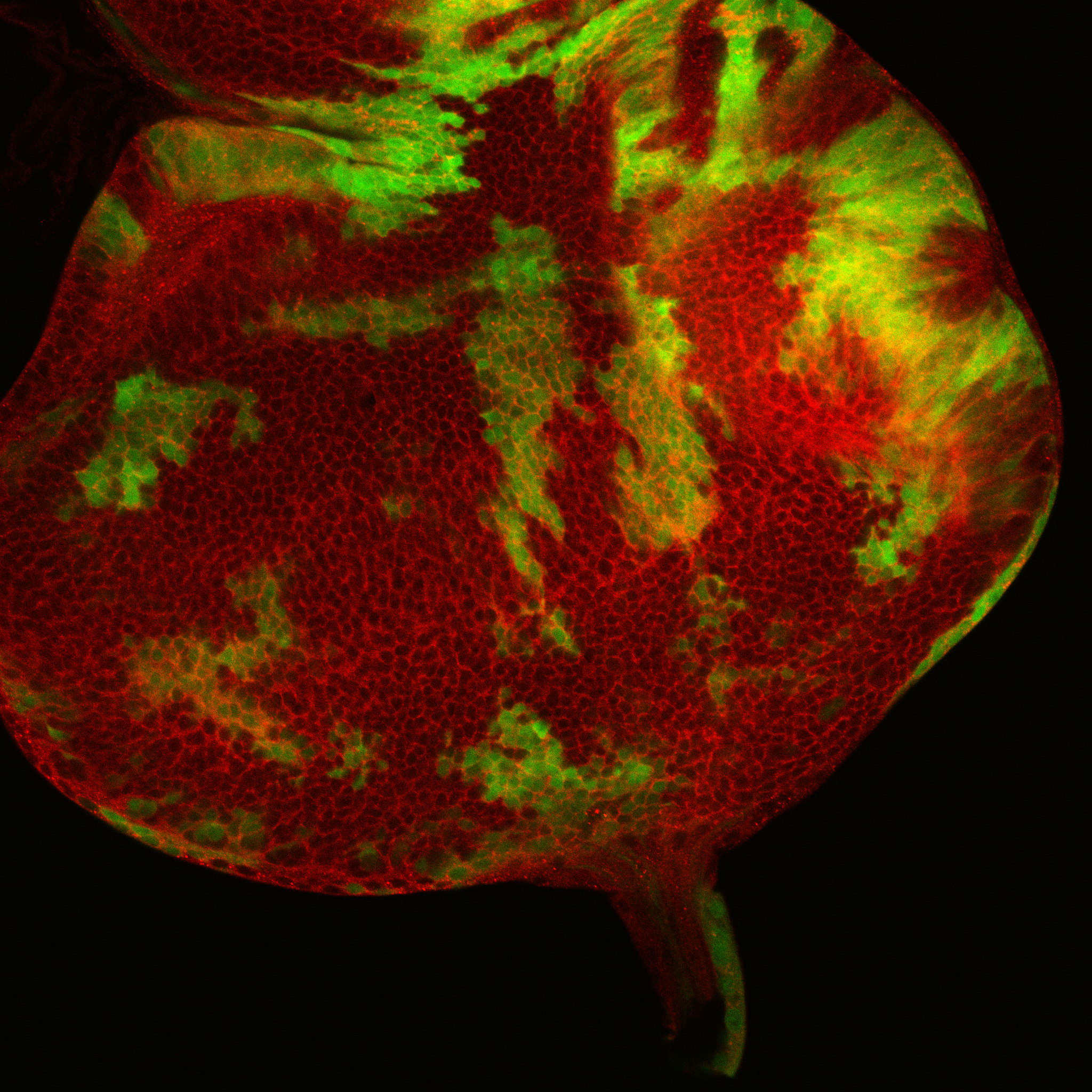

Supplement: Supplementary file 8 — Source data Fig. 4 [file 44318_2025_489_MOESM8_ESM.zip › Figure 4E/5 original image.tif]

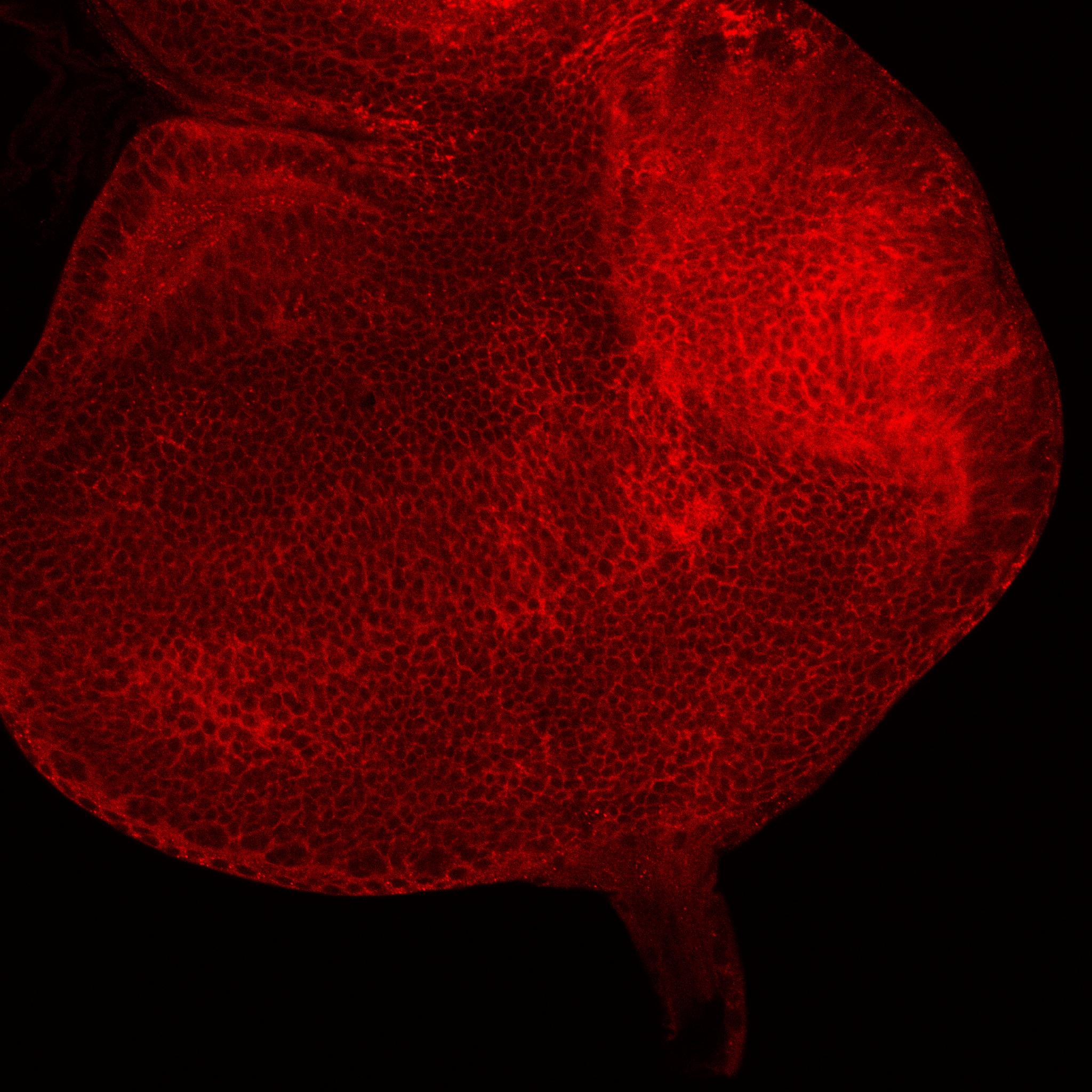

Supplement: Supplementary file 8 — Source data Fig. 4 [file 44318_2025_489_MOESM8_ESM.zip › Figure 4E/6 original image.tif]

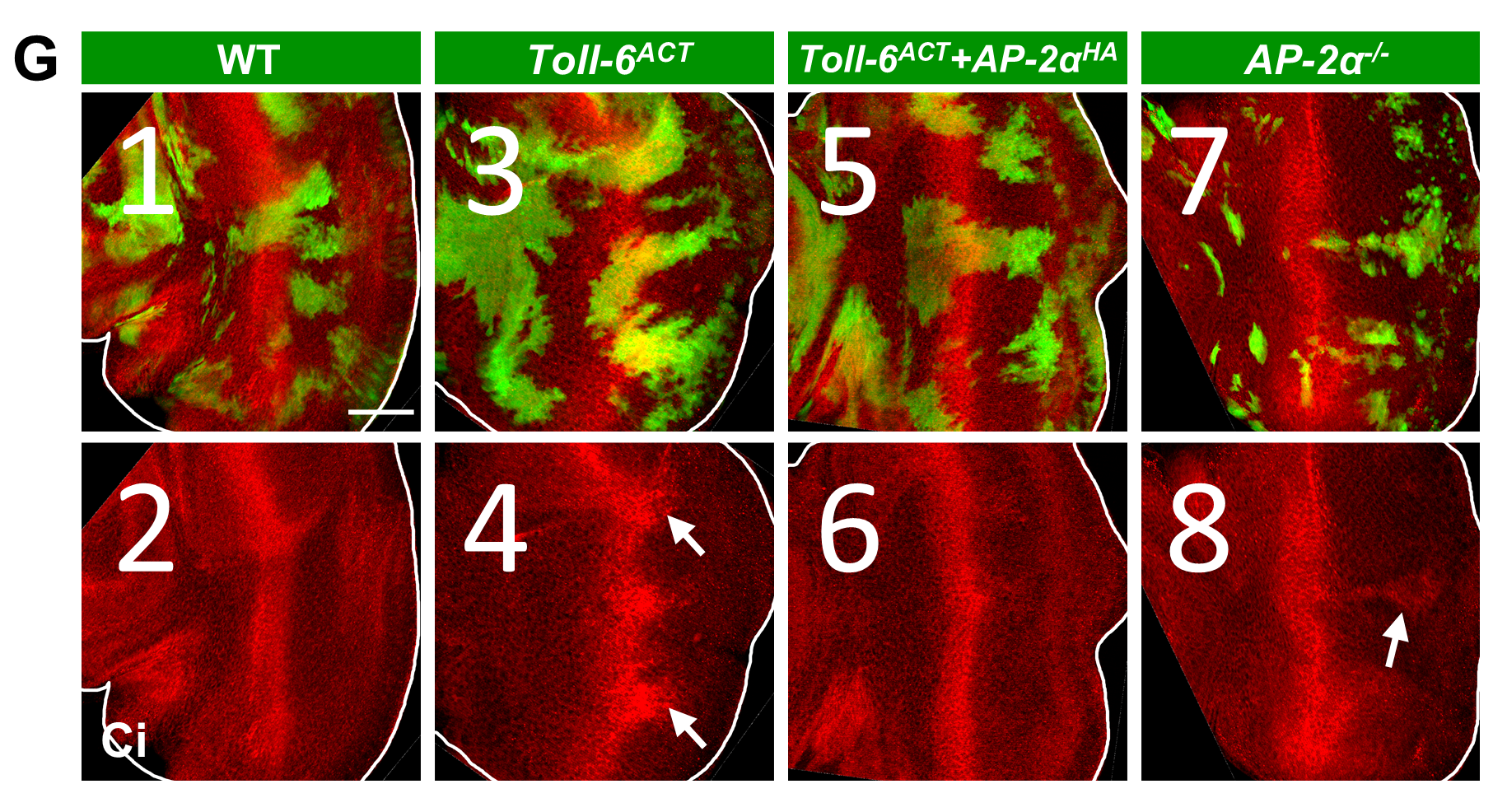

Supplement: Supplementary file 8 — Source data Fig. 4 [file 44318_2025_489_MOESM8_ESM.zip › Figure 4G/0 paper Figure 4G with provided image sequence.tif]

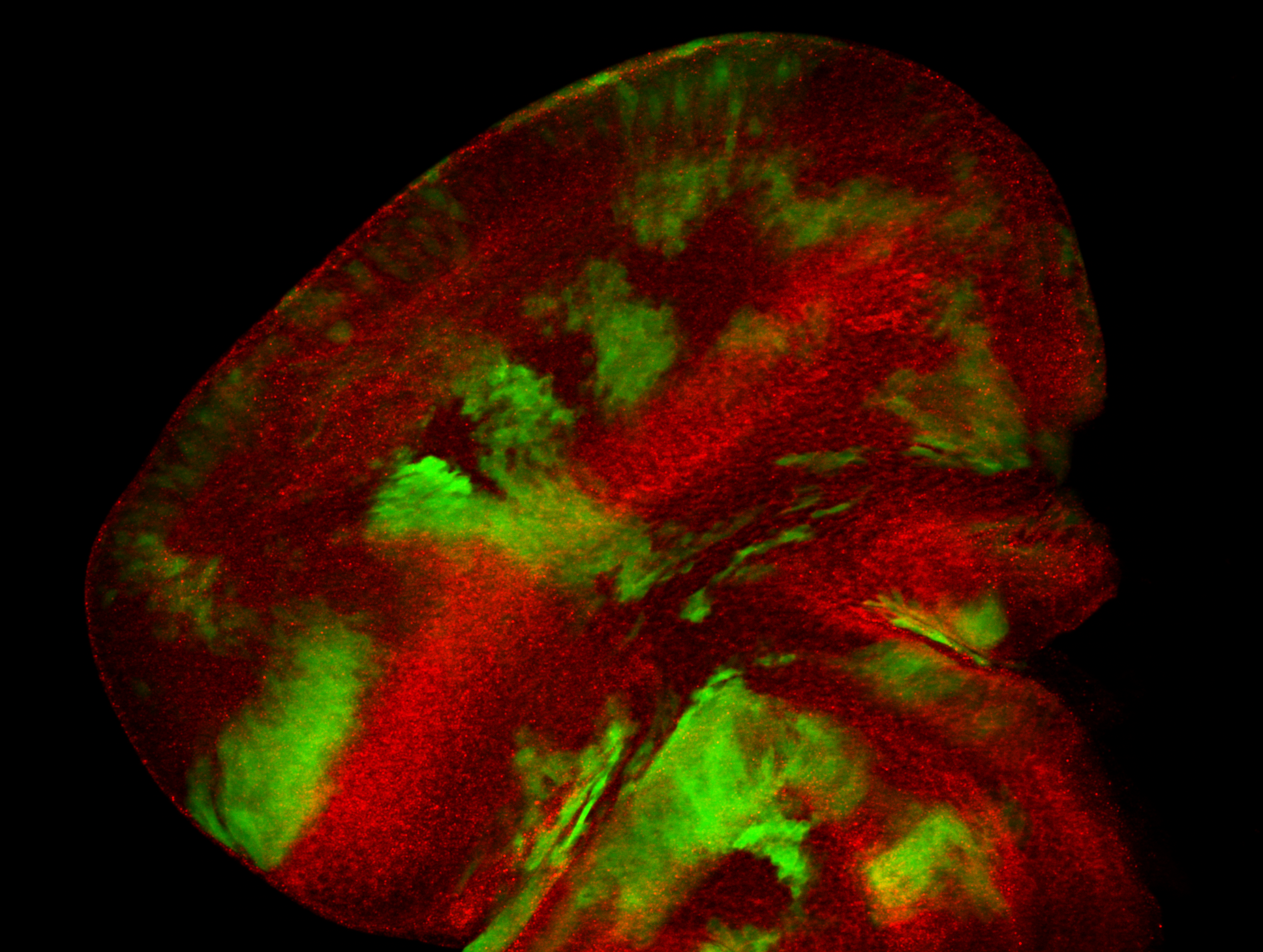

Supplement: Supplementary file 8 — Source data Fig. 4 [file 44318_2025_489_MOESM8_ESM.zip › Figure 4G/1 original image.tif]

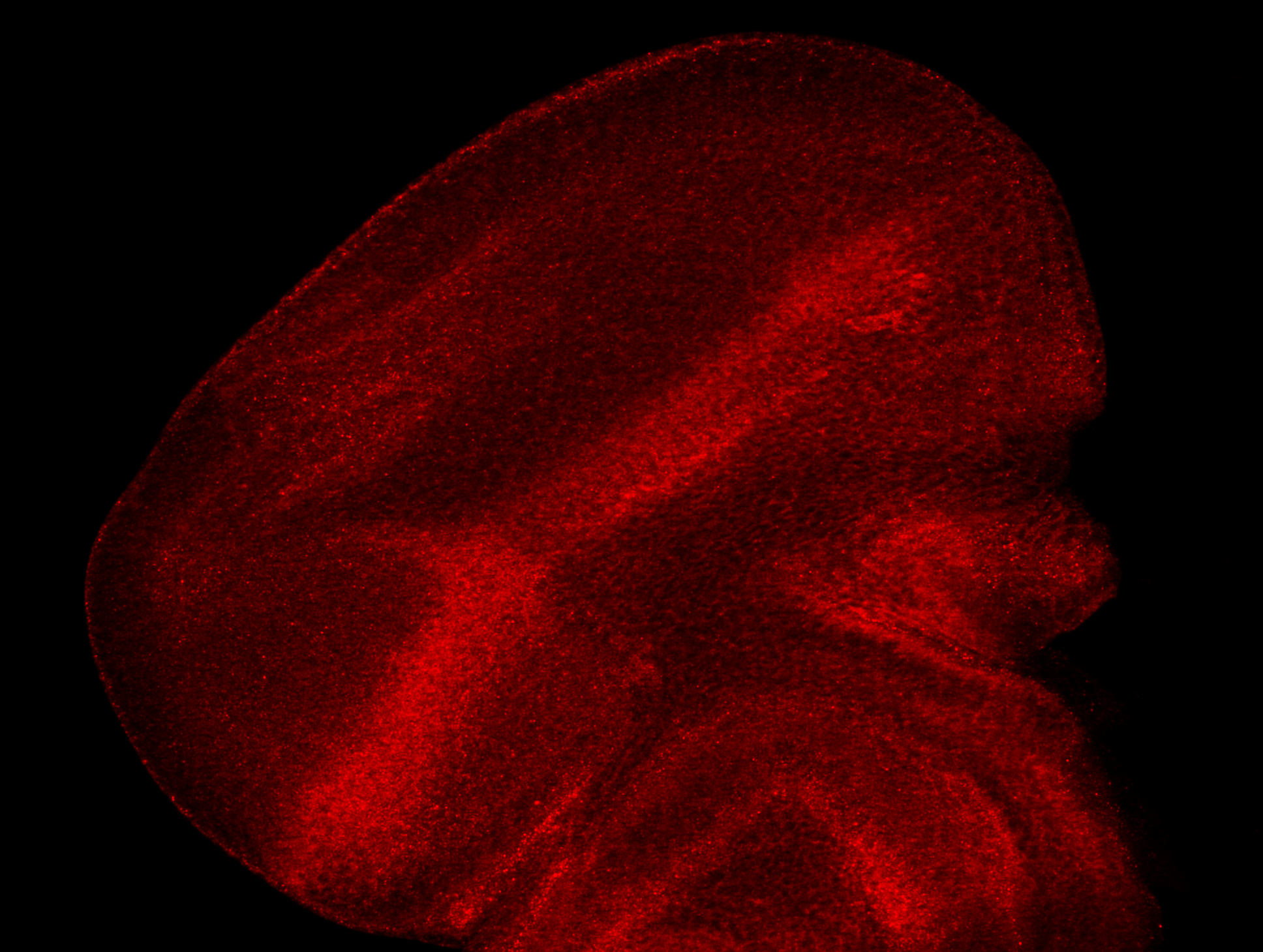

Supplement: Supplementary file 8 — Source data Fig. 4 [file 44318_2025_489_MOESM8_ESM.zip › Figure 4G/2 original image.tif]

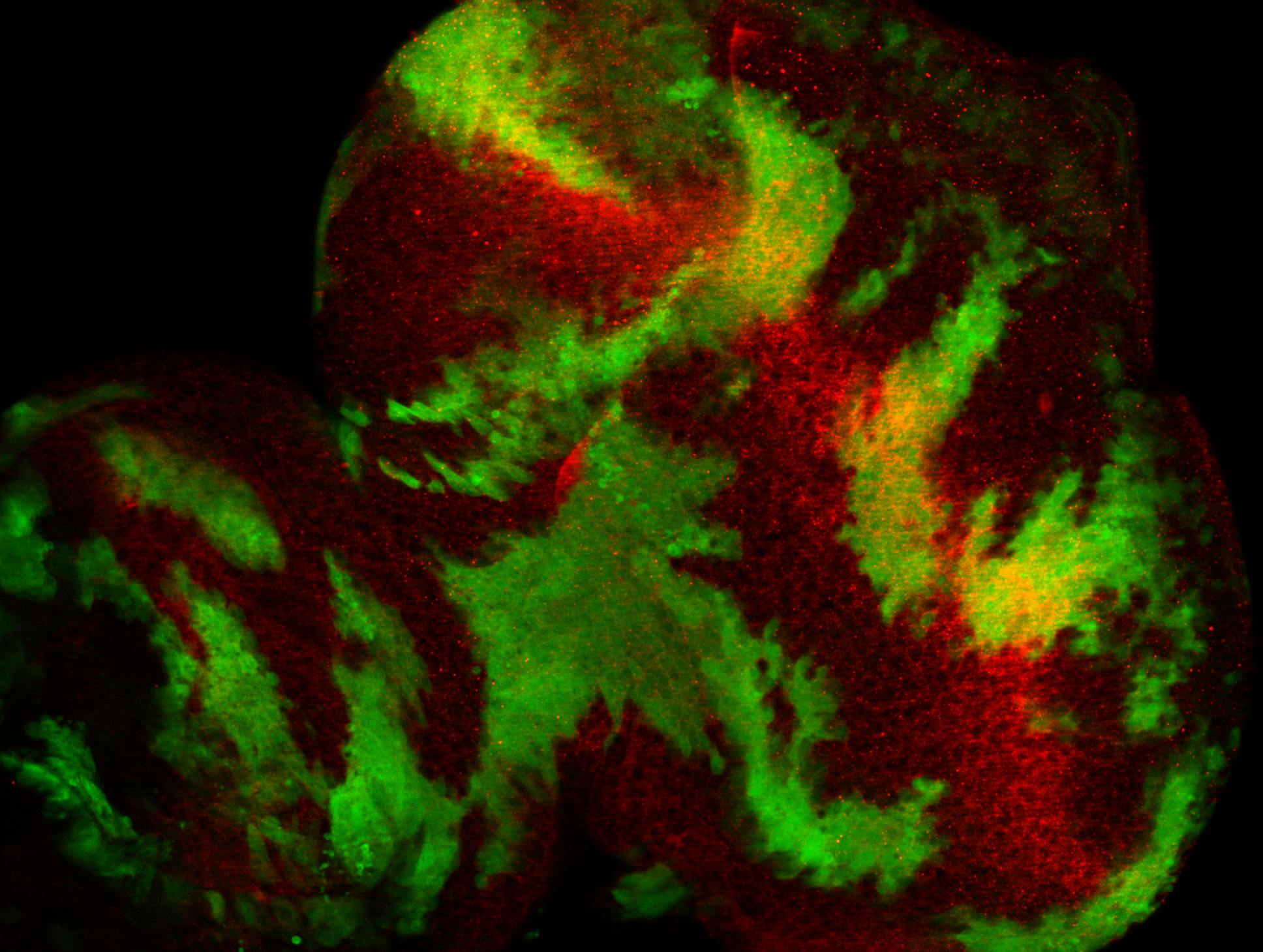

Supplement: Supplementary file 8 — Source data Fig. 4 [file 44318_2025_489_MOESM8_ESM.zip › Figure 4G/3 original image.tif]

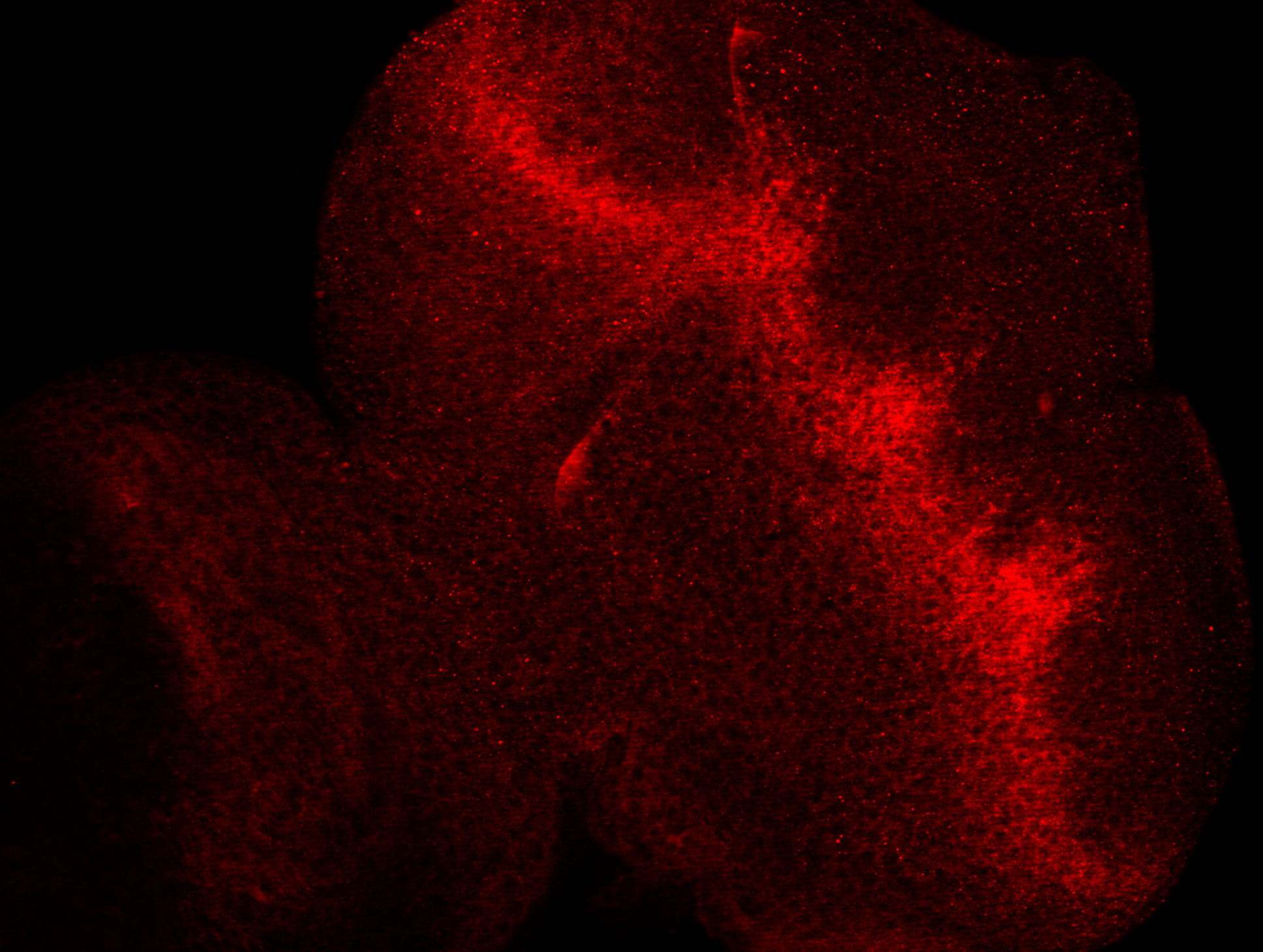

Supplement: Supplementary file 8 — Source data Fig. 4 [file 44318_2025_489_MOESM8_ESM.zip › Figure 4G/4 original image.tif]

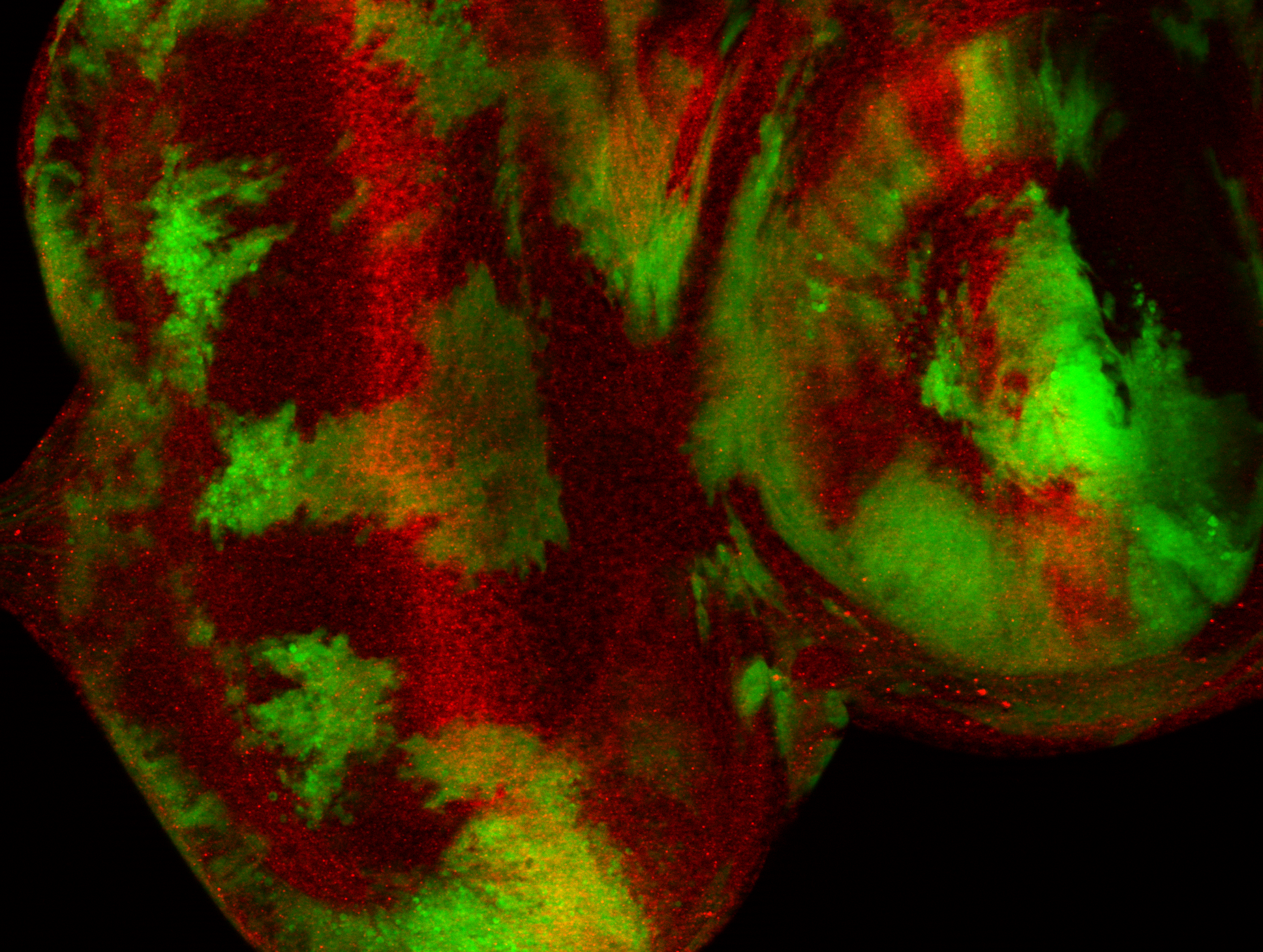

Supplement: Supplementary file 8 — Source data Fig. 4 [file 44318_2025_489_MOESM8_ESM.zip › Figure 4G/5 original image.tif]

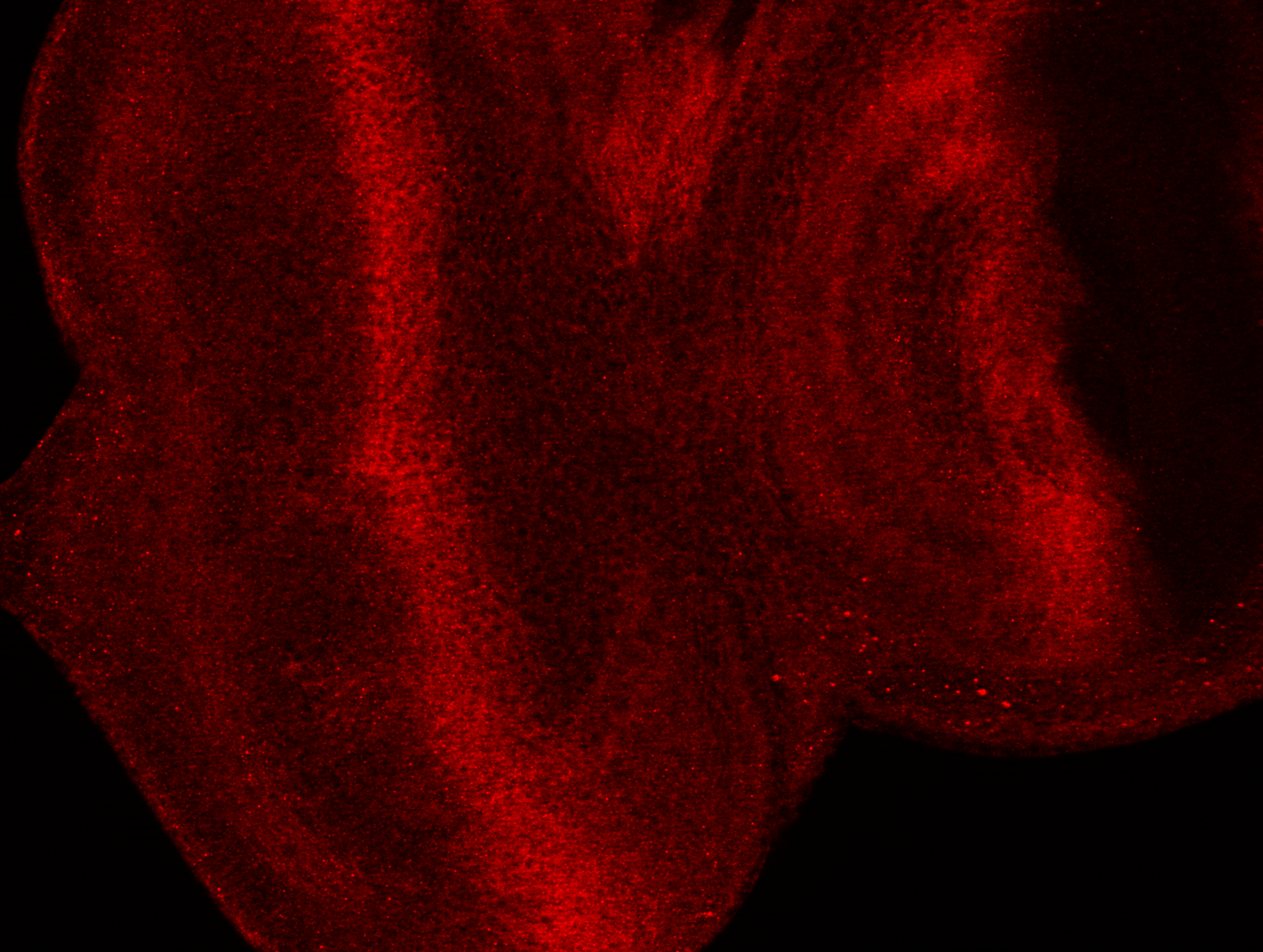

Supplement: Supplementary file 8 — Source data Fig. 4 [file 44318_2025_489_MOESM8_ESM.zip › Figure 4G/6 original image.tif]

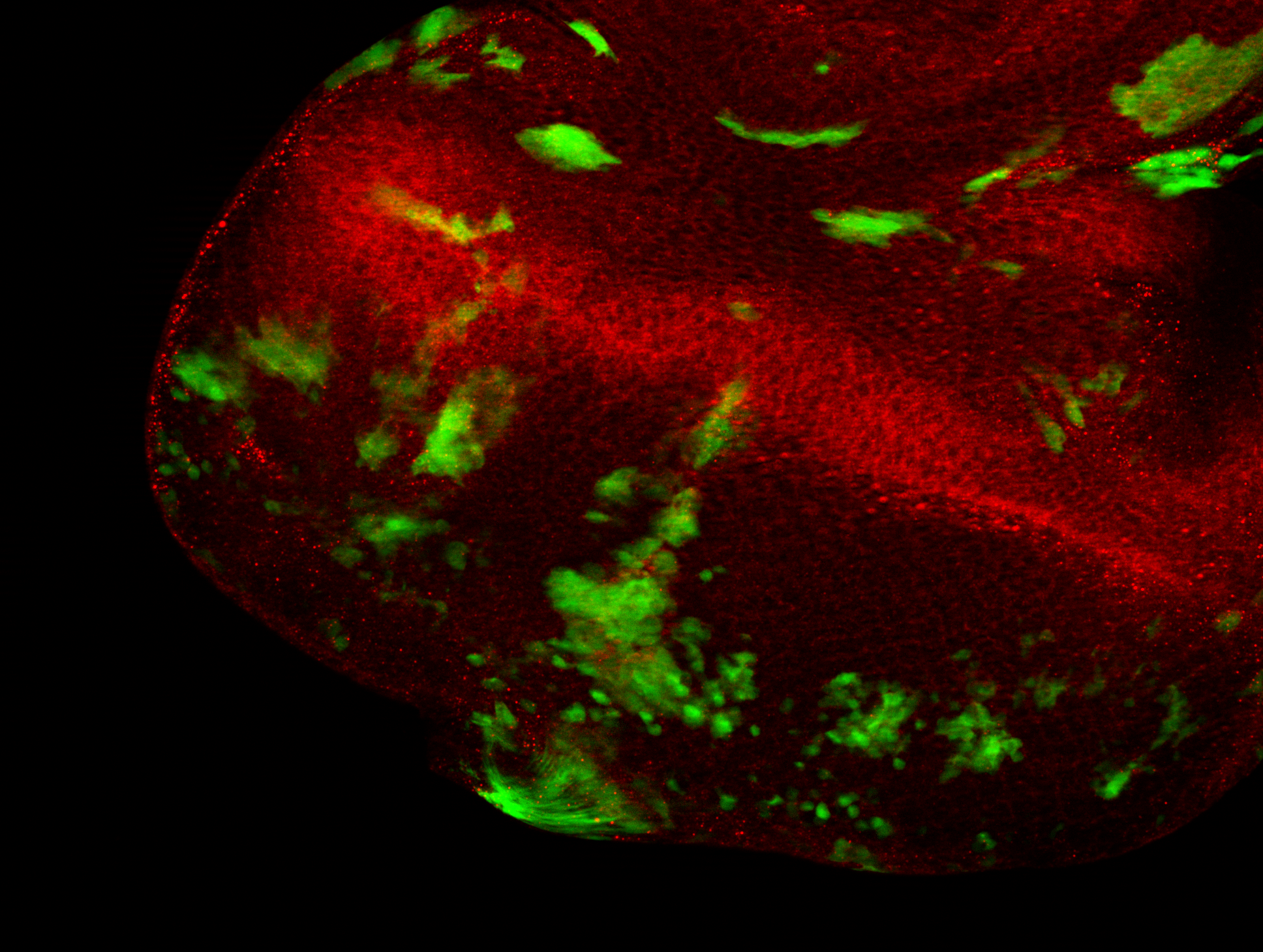

Supplement: Supplementary file 8 — Source data Fig. 4 [file 44318_2025_489_MOESM8_ESM.zip › Figure 4G/7 original image.tif]

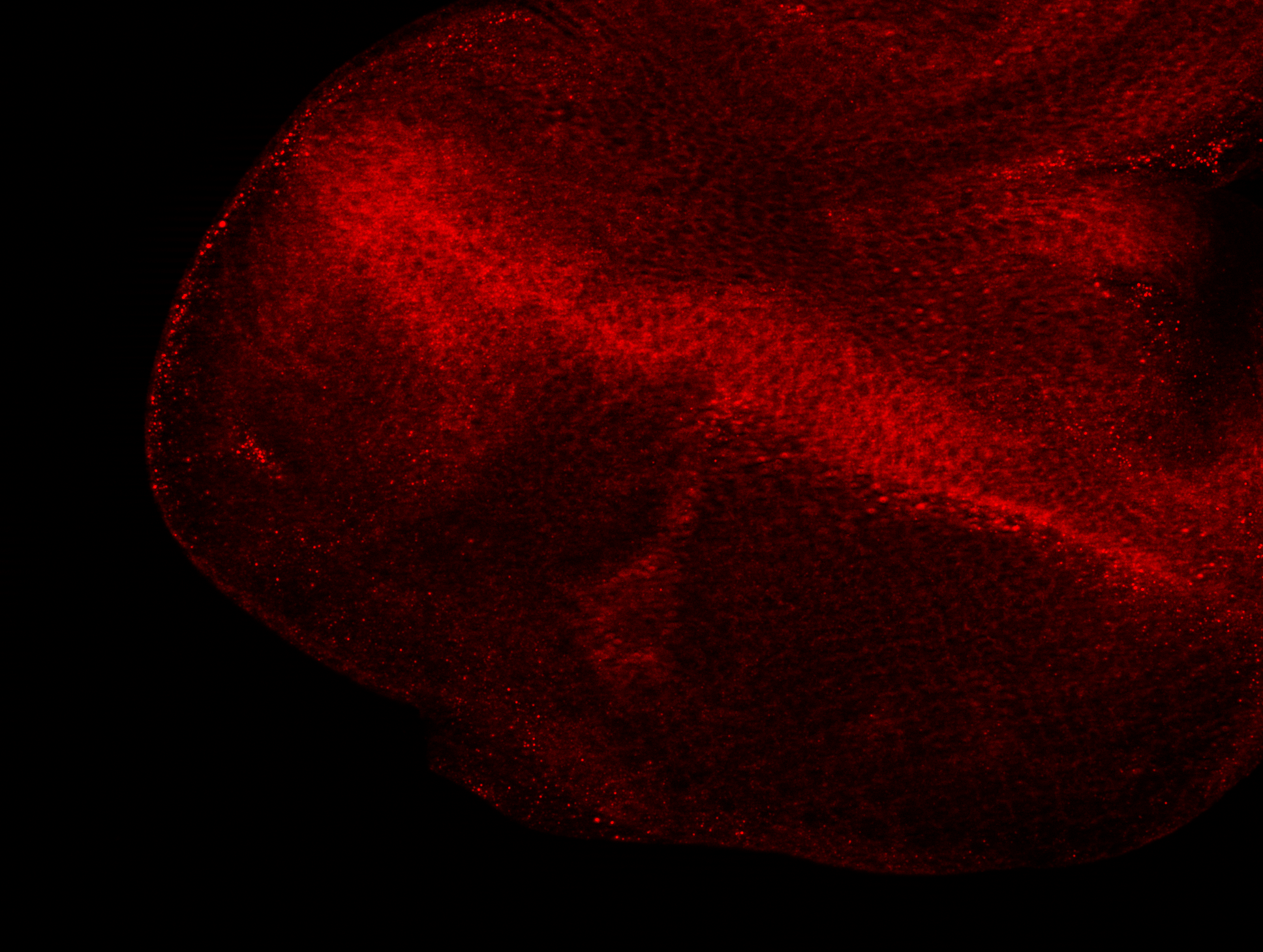

Supplement: Supplementary file 8 — Source data Fig. 4 [file 44318_2025_489_MOESM8_ESM.zip › Figure 4G/8 original image.tif]

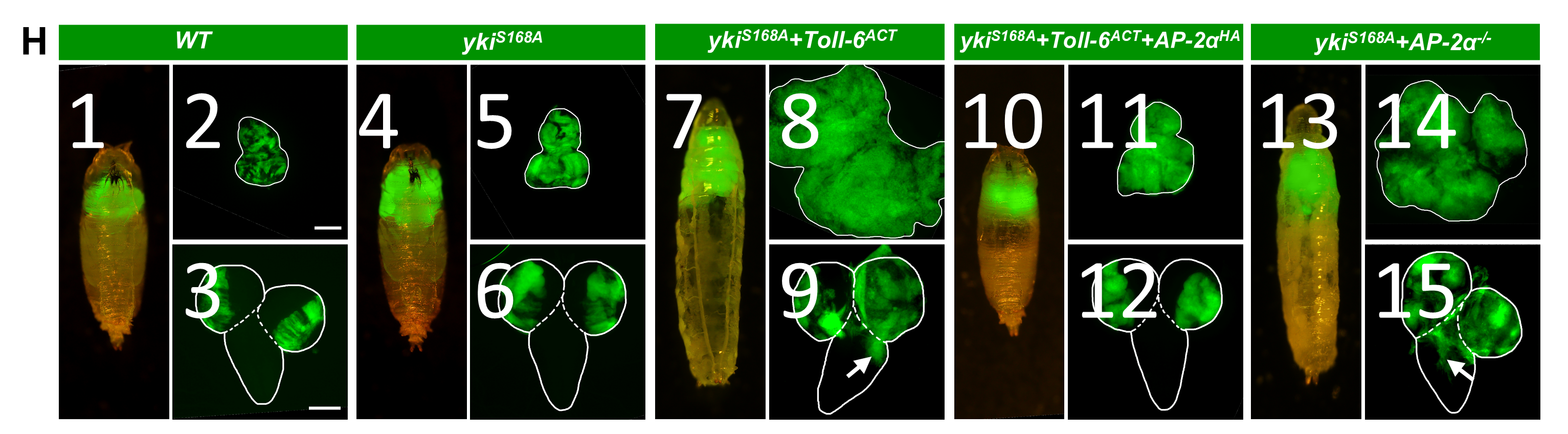

Supplement: Supplementary file 8 — Source data Fig. 4 [file 44318_2025_489_MOESM8_ESM.zip › Figure 4H/0 paper Figure 4H with provided image sequence.tif]

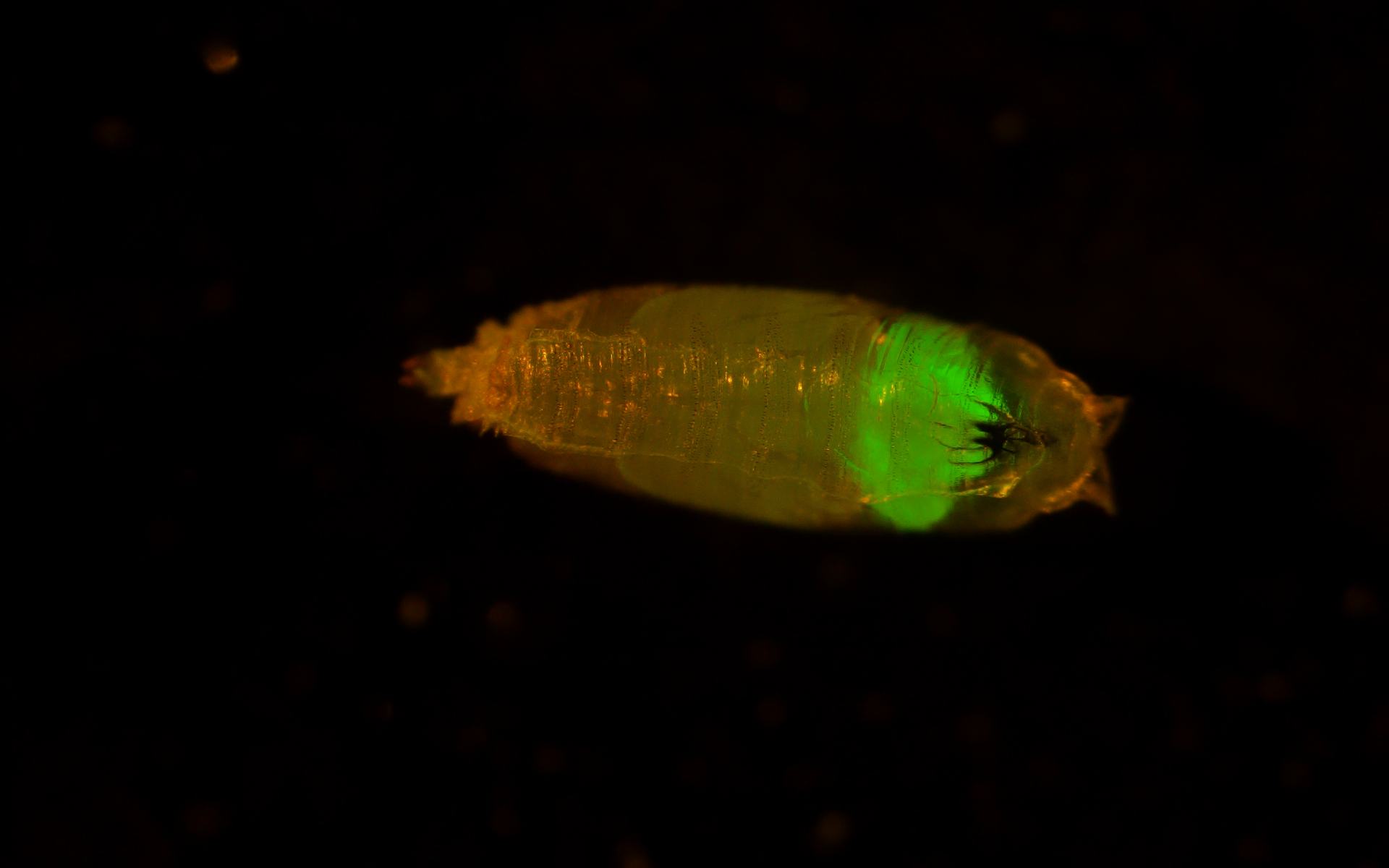

Supplement: Supplementary file 8 — Source data Fig. 4 [file 44318_2025_489_MOESM8_ESM.zip › Figure 4H/1 original image.tif]

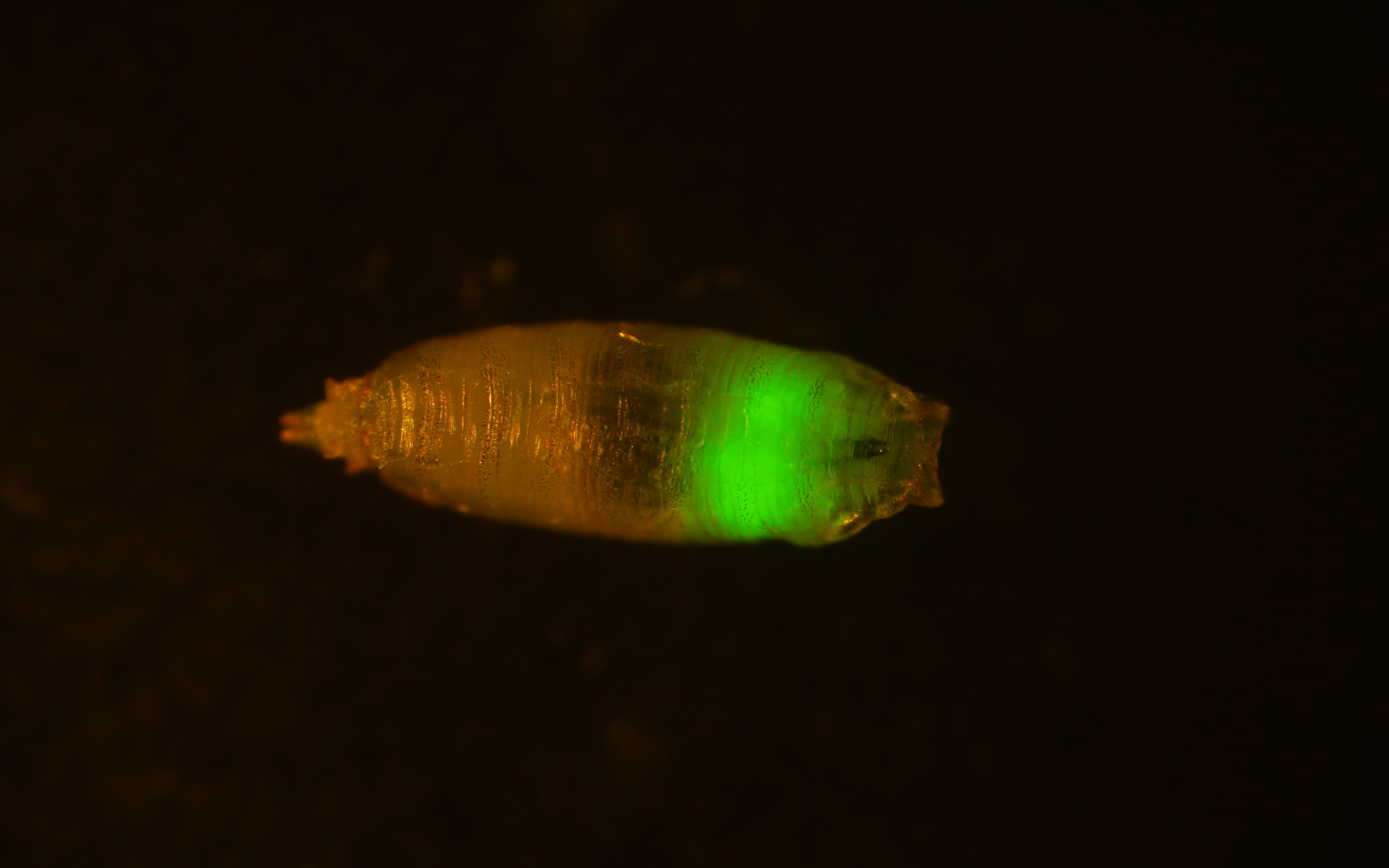

Supplement: Supplementary file 8 — Source data Fig. 4 [file 44318_2025_489_MOESM8_ESM.zip › Figure 4H/10 original image.tif]

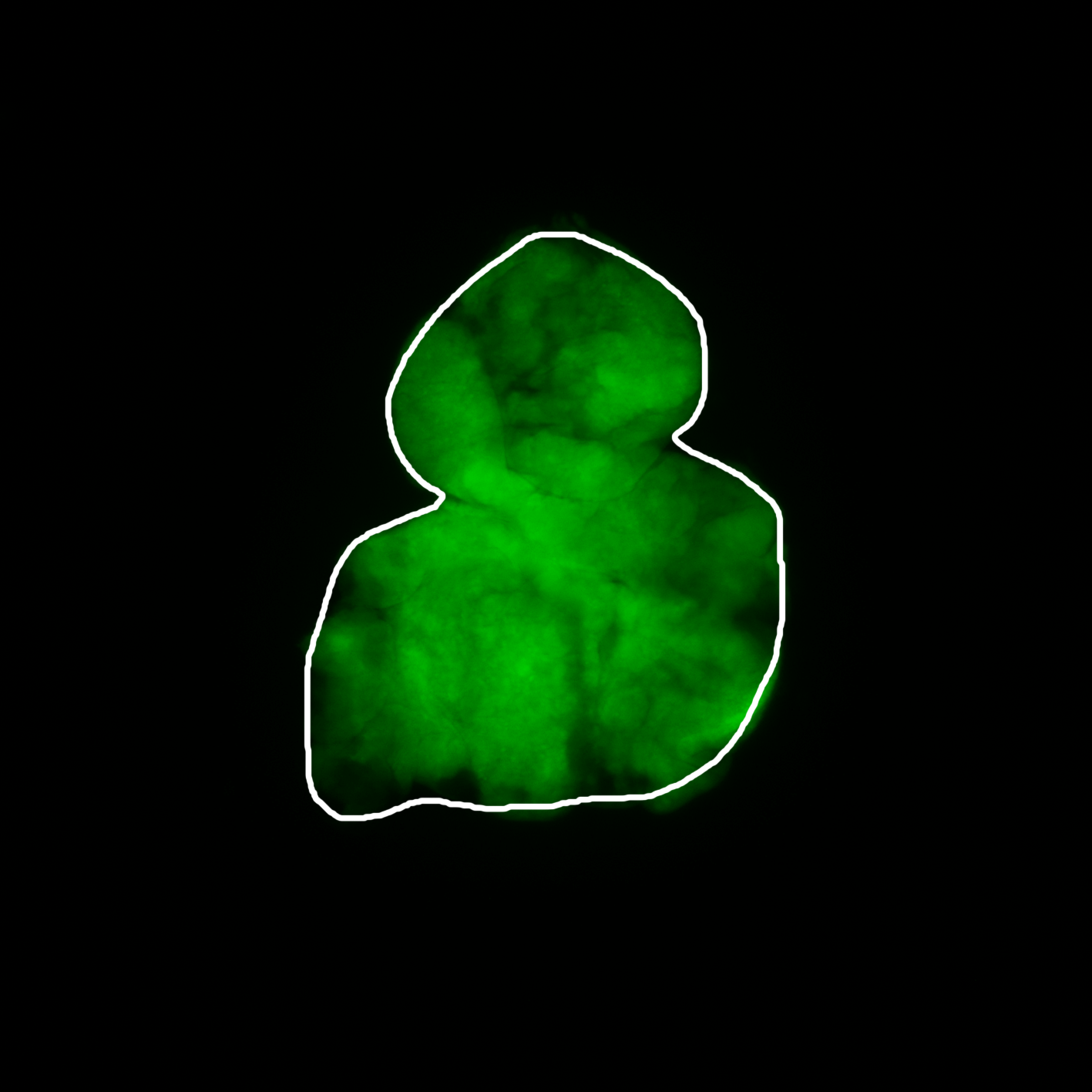

Supplement: Supplementary file 8 — Source data Fig. 4 [file 44318_2025_489_MOESM8_ESM.zip › Figure 4H/11-1 rotated and cut image with border line.tif]

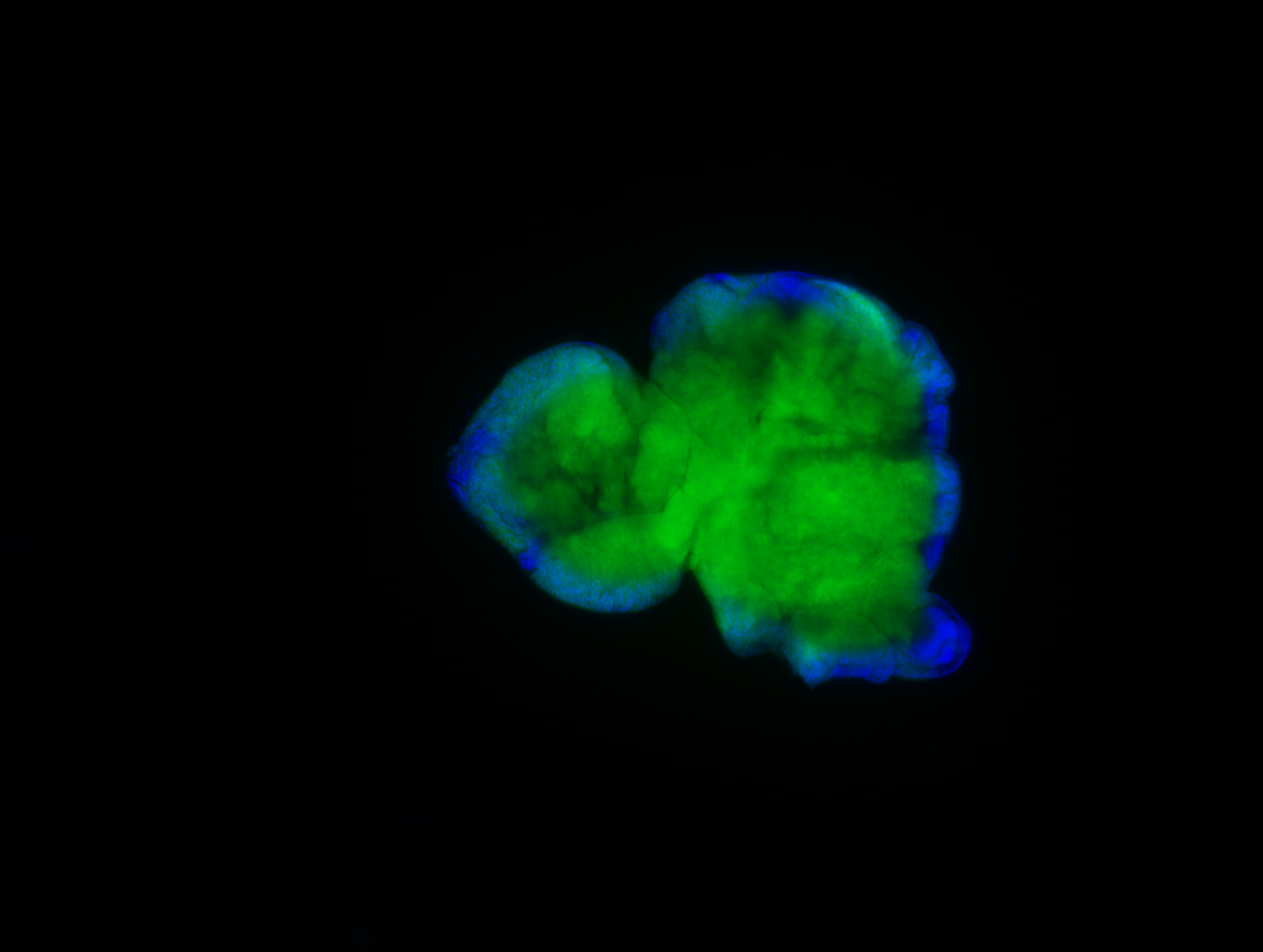

Supplement: Supplementary file 8 — Source data Fig. 4 [file 44318_2025_489_MOESM8_ESM.zip › Figure 4H/11-2 original image.tif]

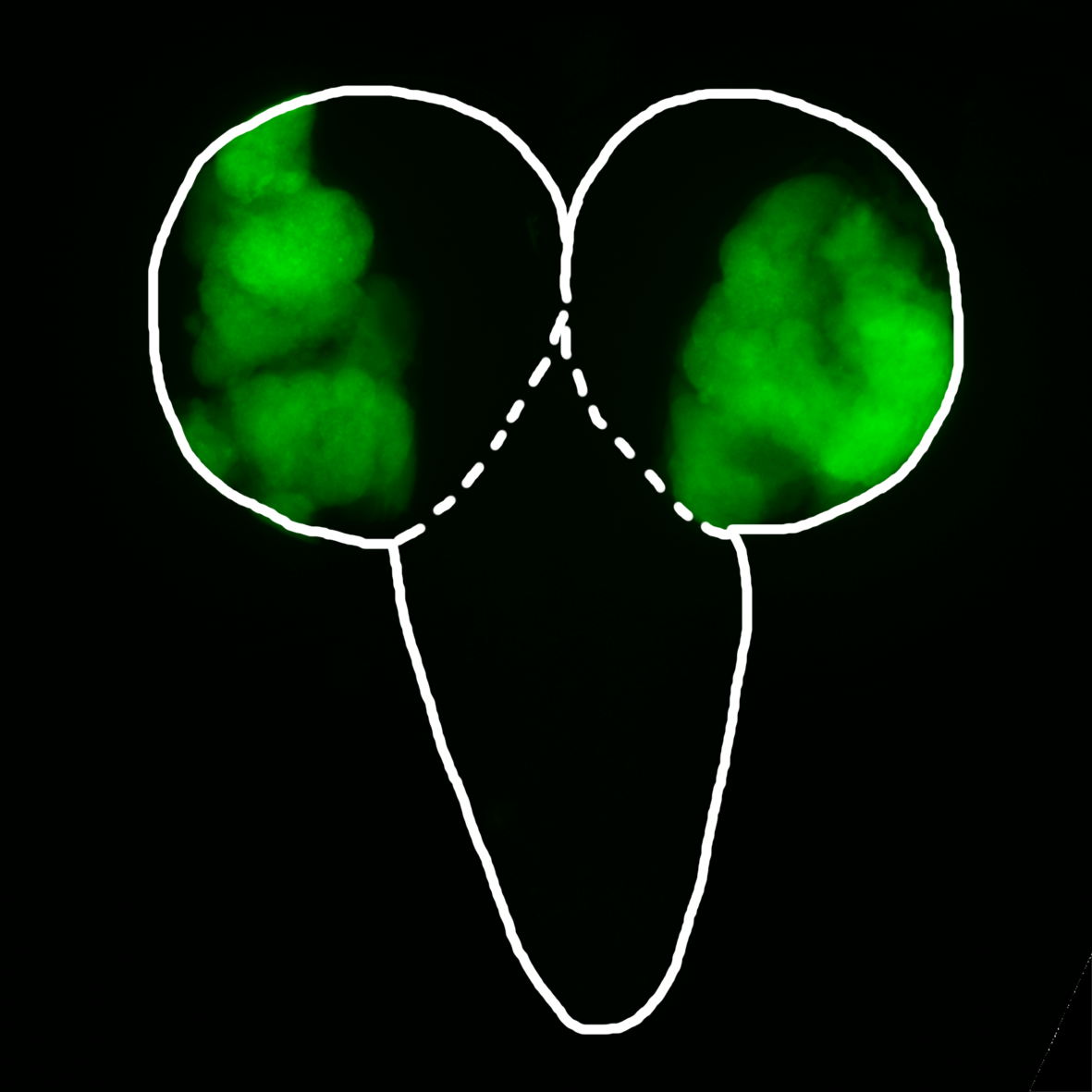

Supplement: Supplementary file 8 — Source data Fig. 4 [file 44318_2025_489_MOESM8_ESM.zip › Figure 4H/12-1 rotated and cut image with border line.tif]

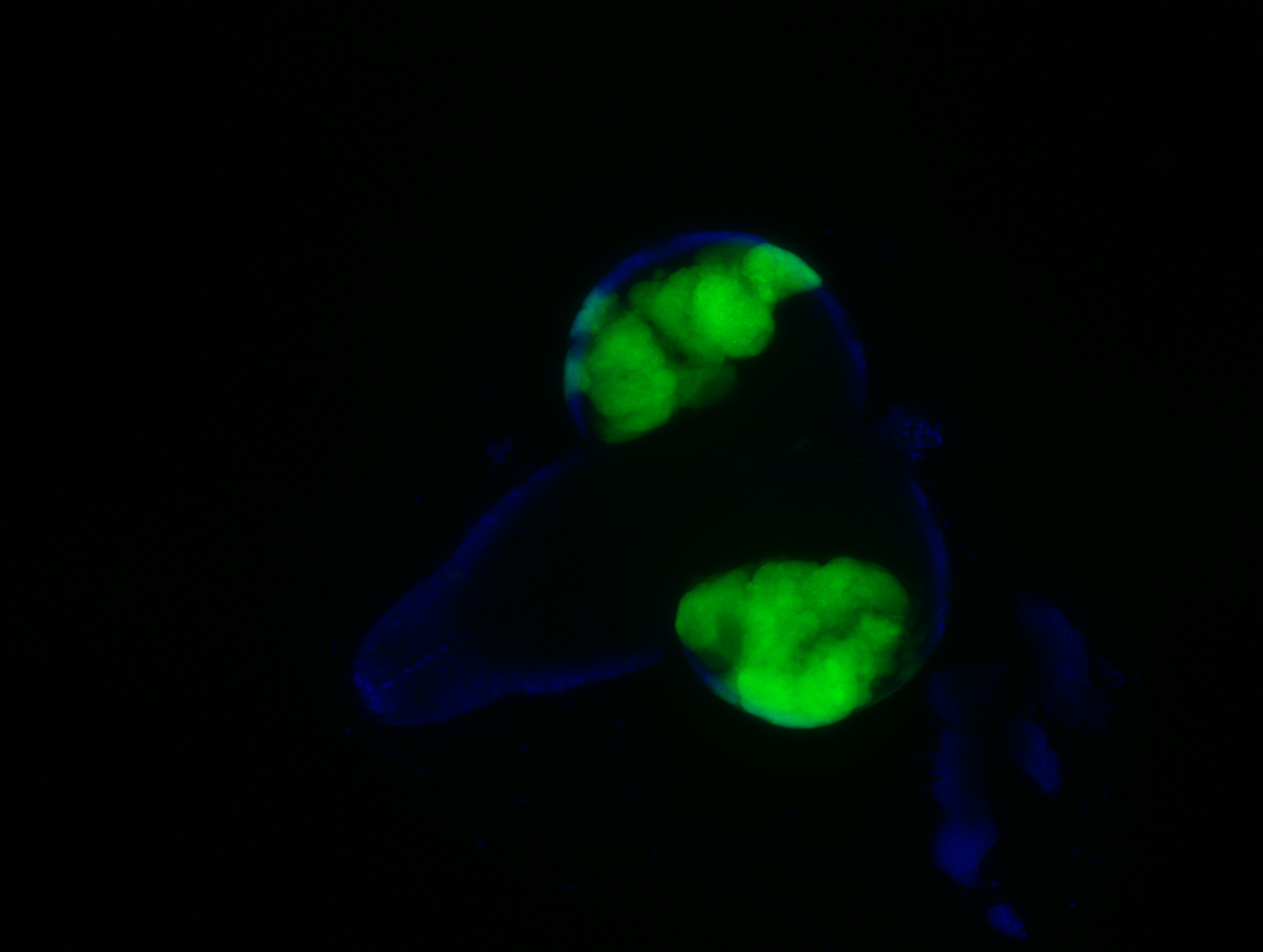

Supplement: Supplementary file 8 — Source data Fig. 4 [file 44318_2025_489_MOESM8_ESM.zip › Figure 4H/12-2 original image.tif]

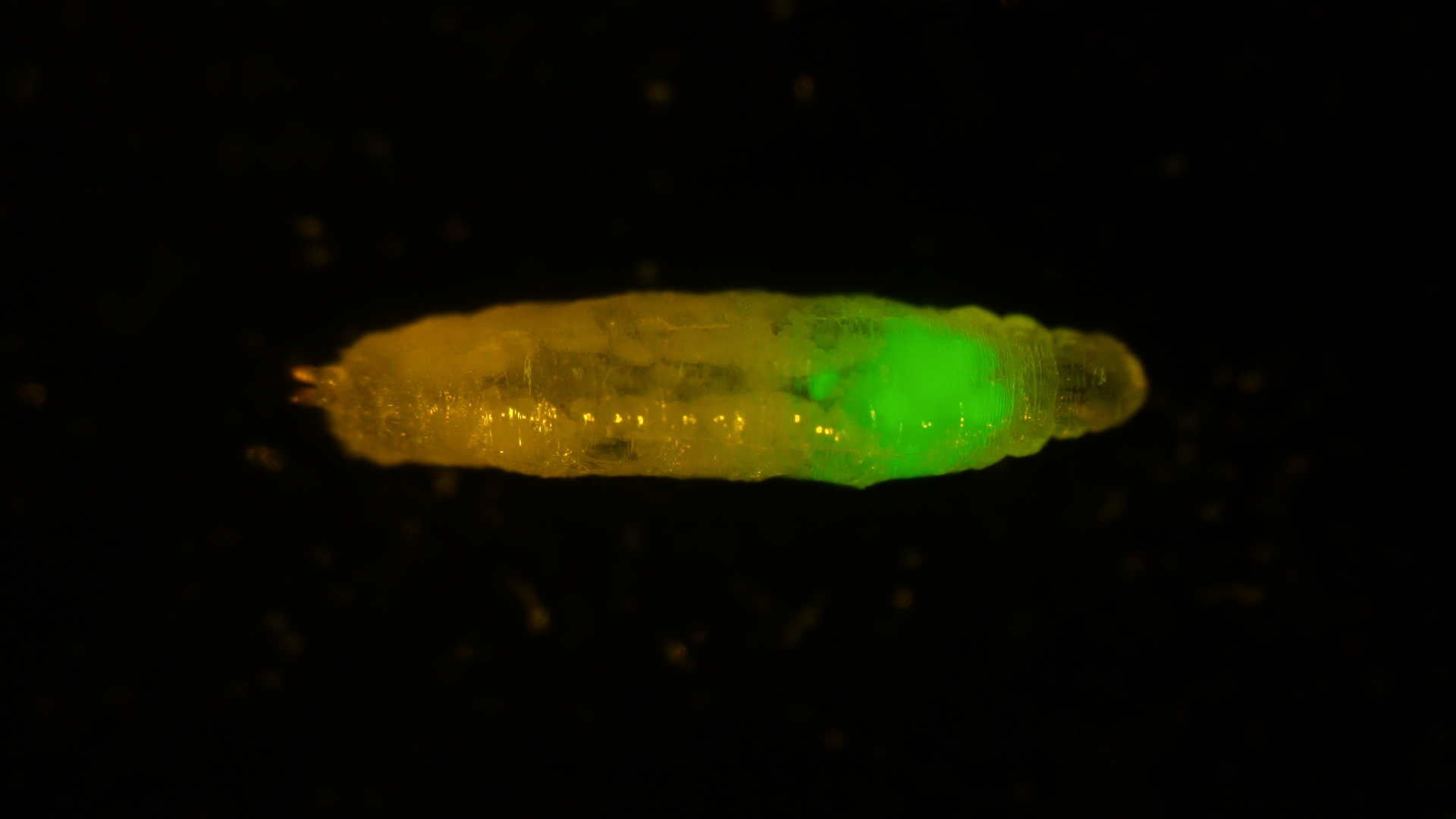

Supplement: Supplementary file 8 — Source data Fig. 4 [file 44318_2025_489_MOESM8_ESM.zip › Figure 4H/13 original image.tif]

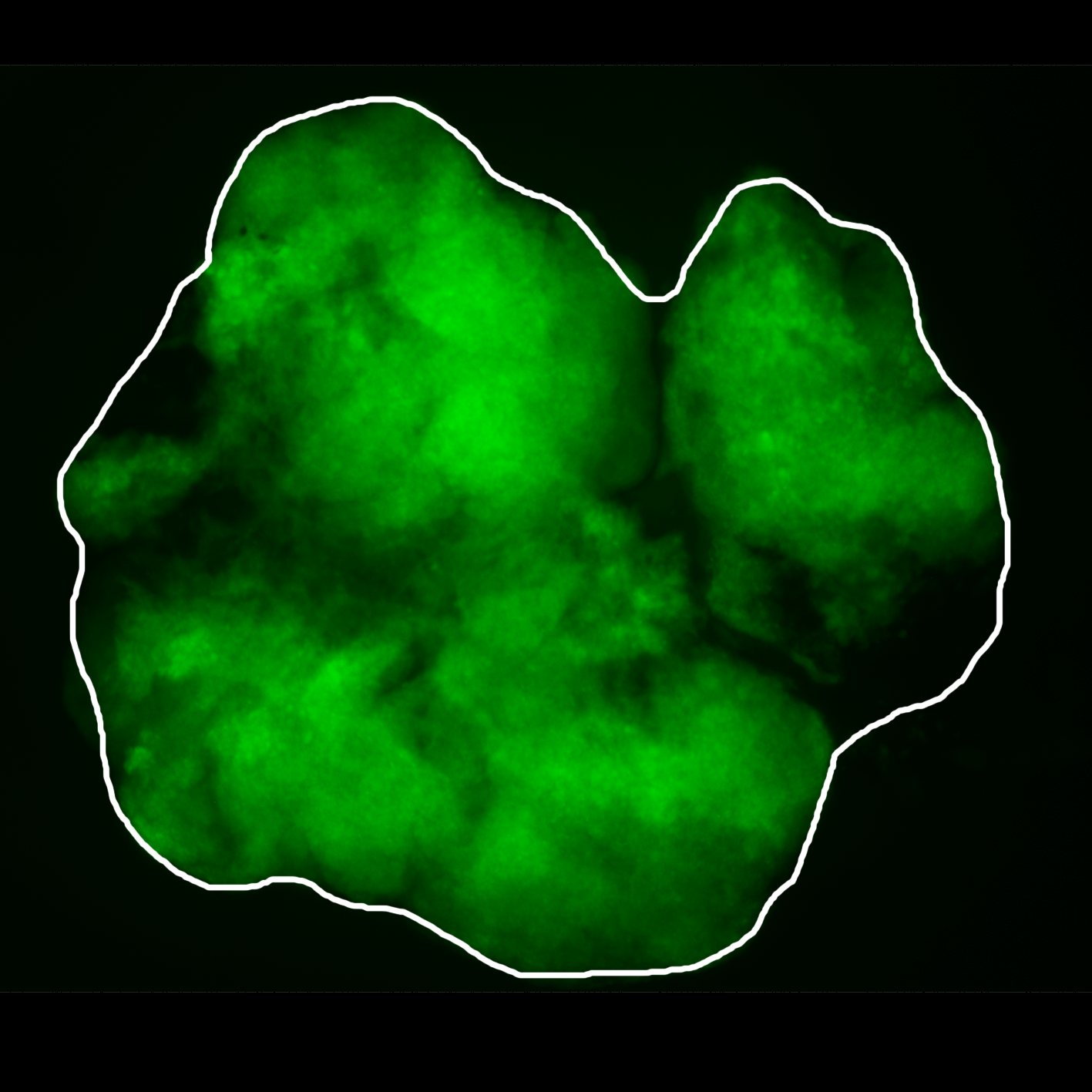

Supplement: Supplementary file 8 — Source data Fig. 4 [file 44318_2025_489_MOESM8_ESM.zip › Figure 4H/14-1 rotated and cut image with border line.tif]

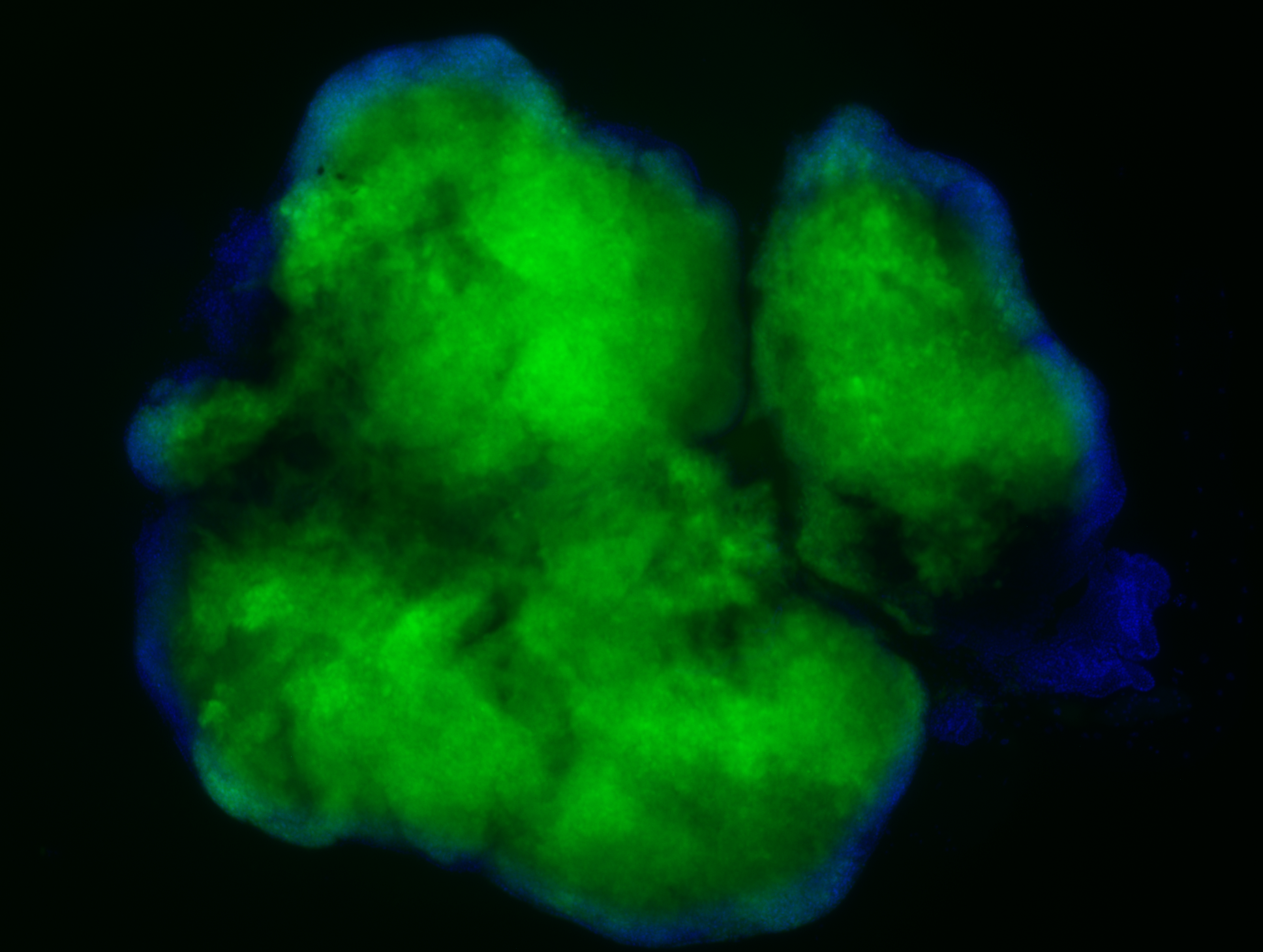

Supplement: Supplementary file 8 — Source data Fig. 4 [file 44318_2025_489_MOESM8_ESM.zip › Figure 4H/14-2 original image.tif]

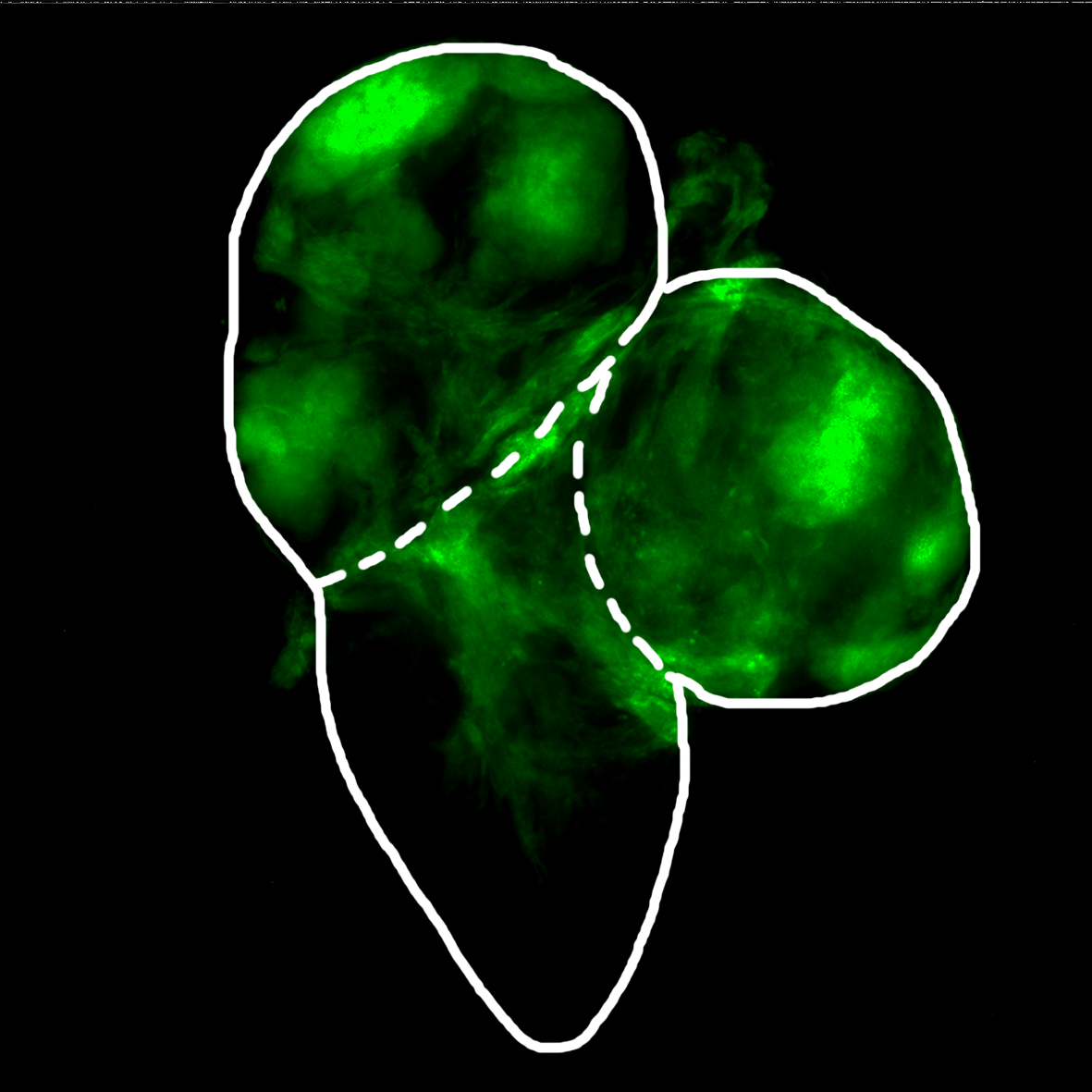

Supplement: Supplementary file 8 — Source data Fig. 4 [file 44318_2025_489_MOESM8_ESM.zip › Figure 4H/15-1 rotated and cut image with border line.tif]

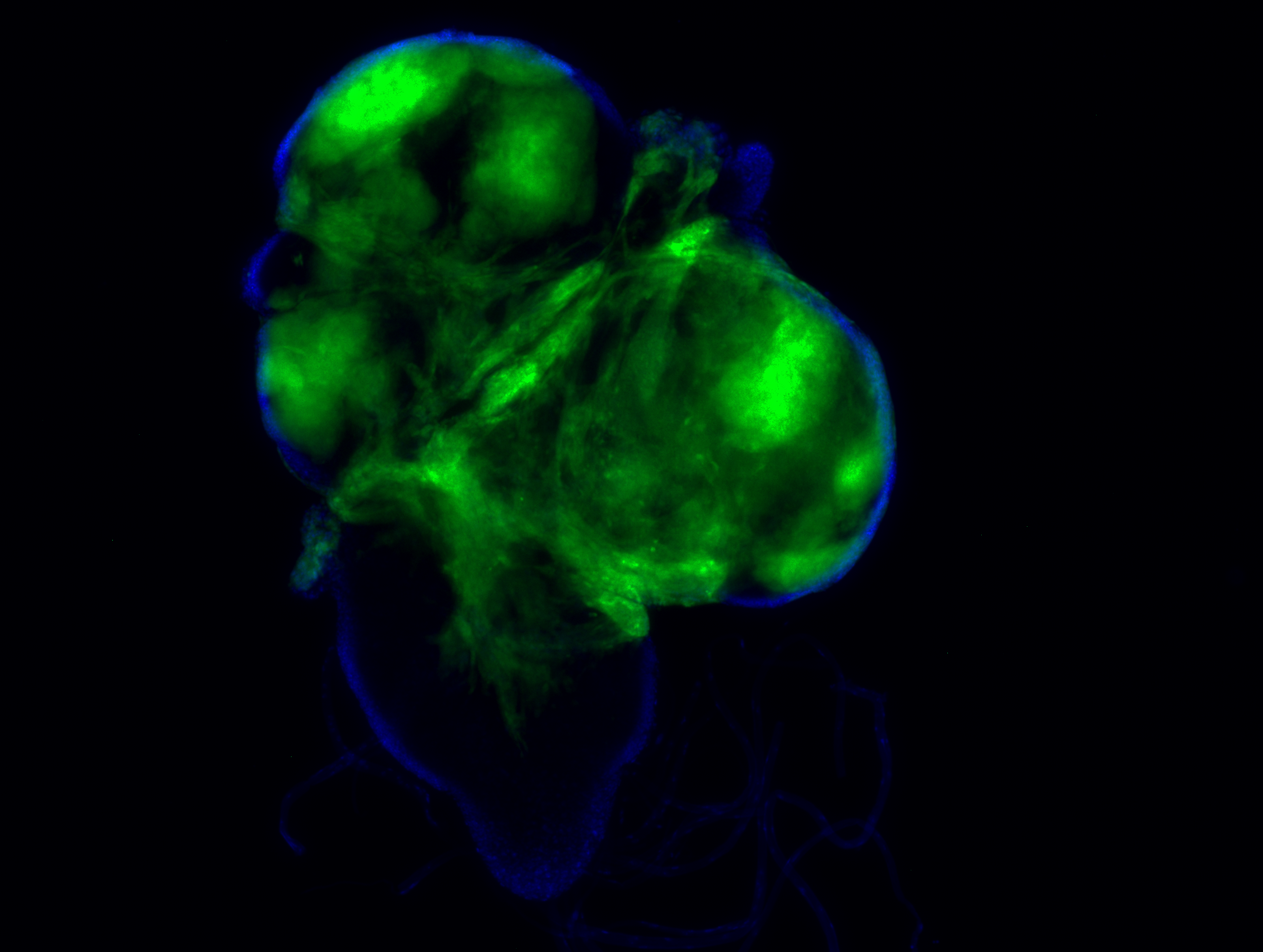

Supplement: Supplementary file 8 — Source data Fig. 4 [file 44318_2025_489_MOESM8_ESM.zip › Figure 4H/15-2 original image.tif]

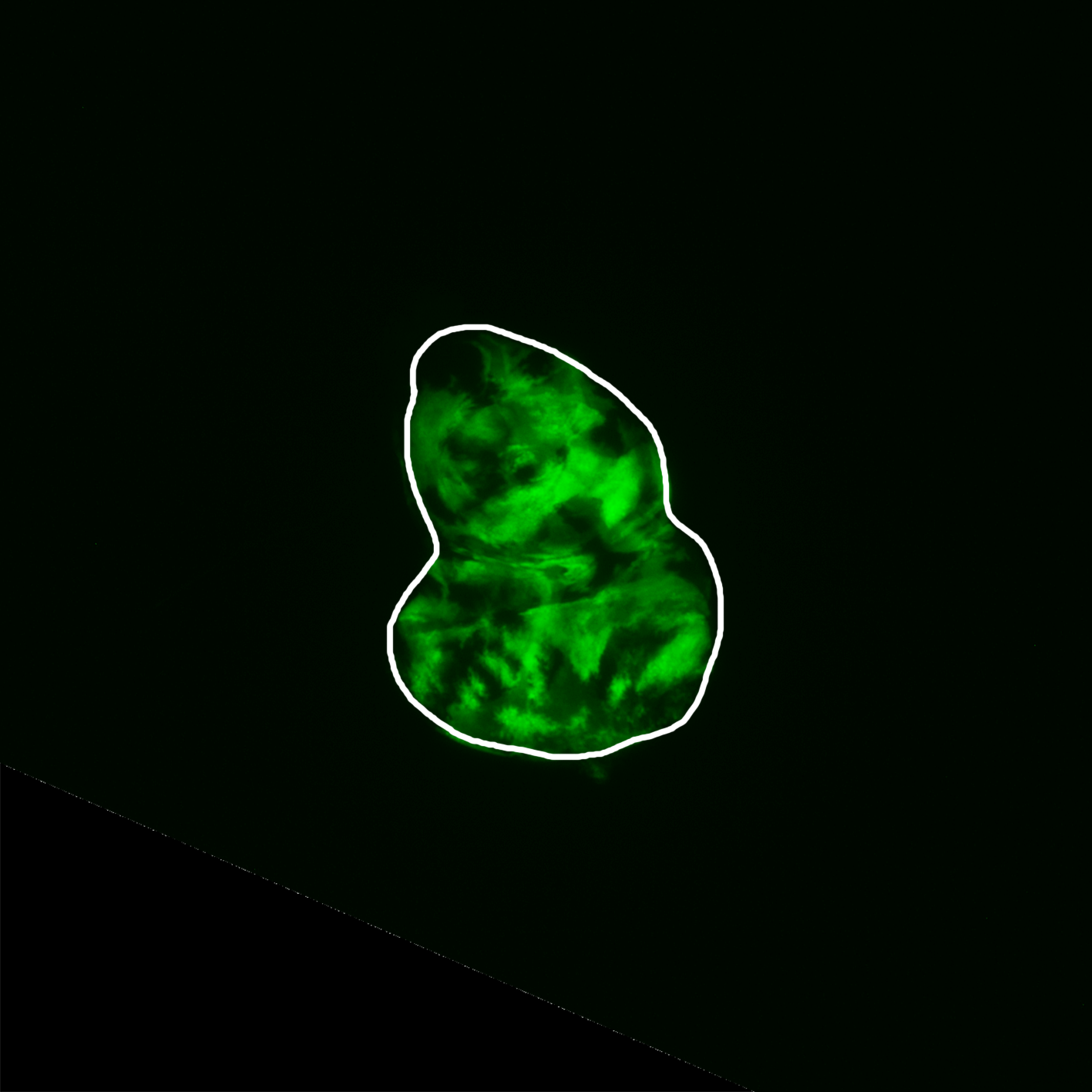

Supplement: Supplementary file 8 — Source data Fig. 4 [file 44318_2025_489_MOESM8_ESM.zip › Figure 4H/2-1 rotated and cut image with border line.tif]

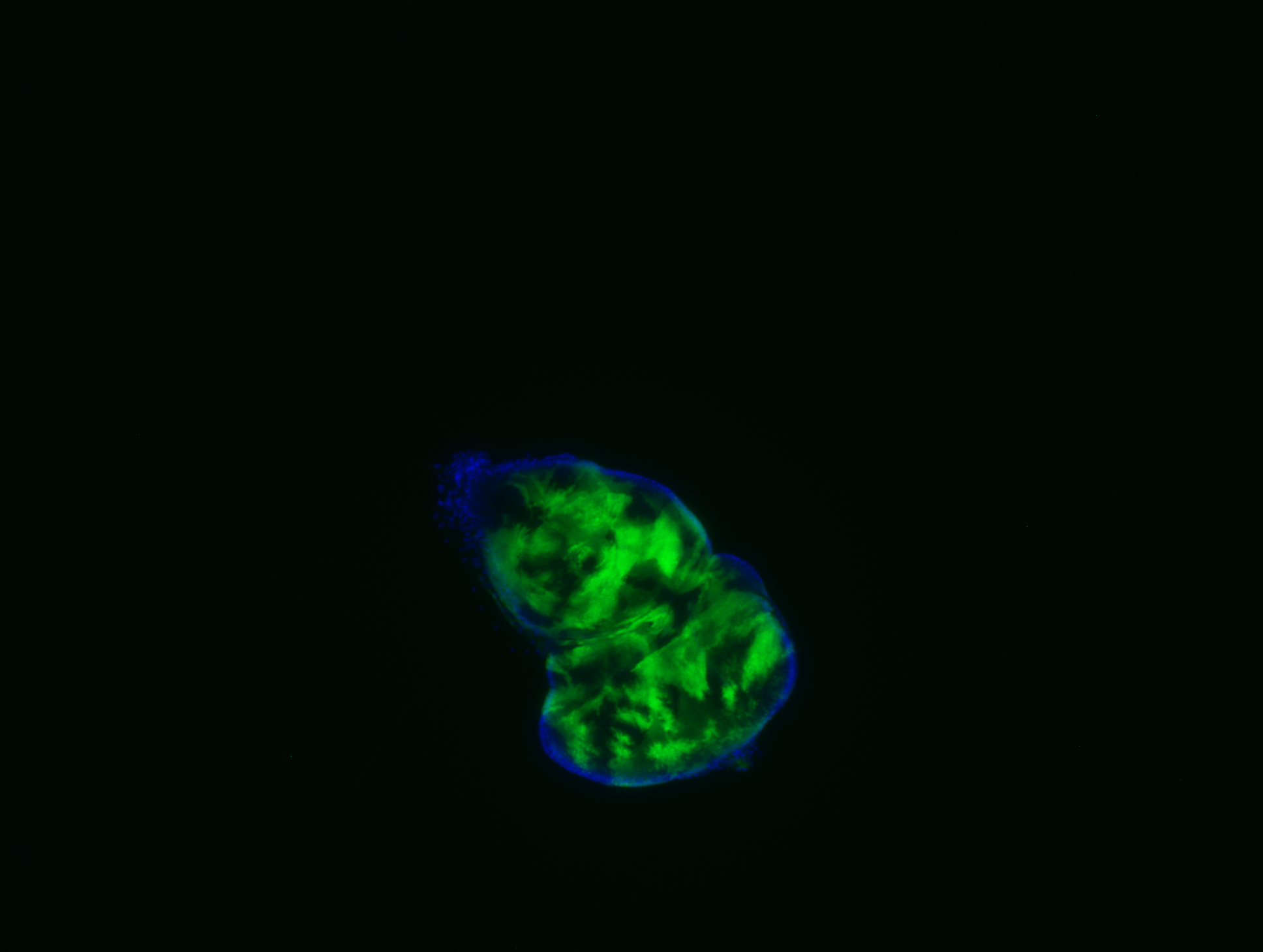

Supplement: Supplementary file 8 — Source data Fig. 4 [file 44318_2025_489_MOESM8_ESM.zip › Figure 4H/2-2 original image.tif]

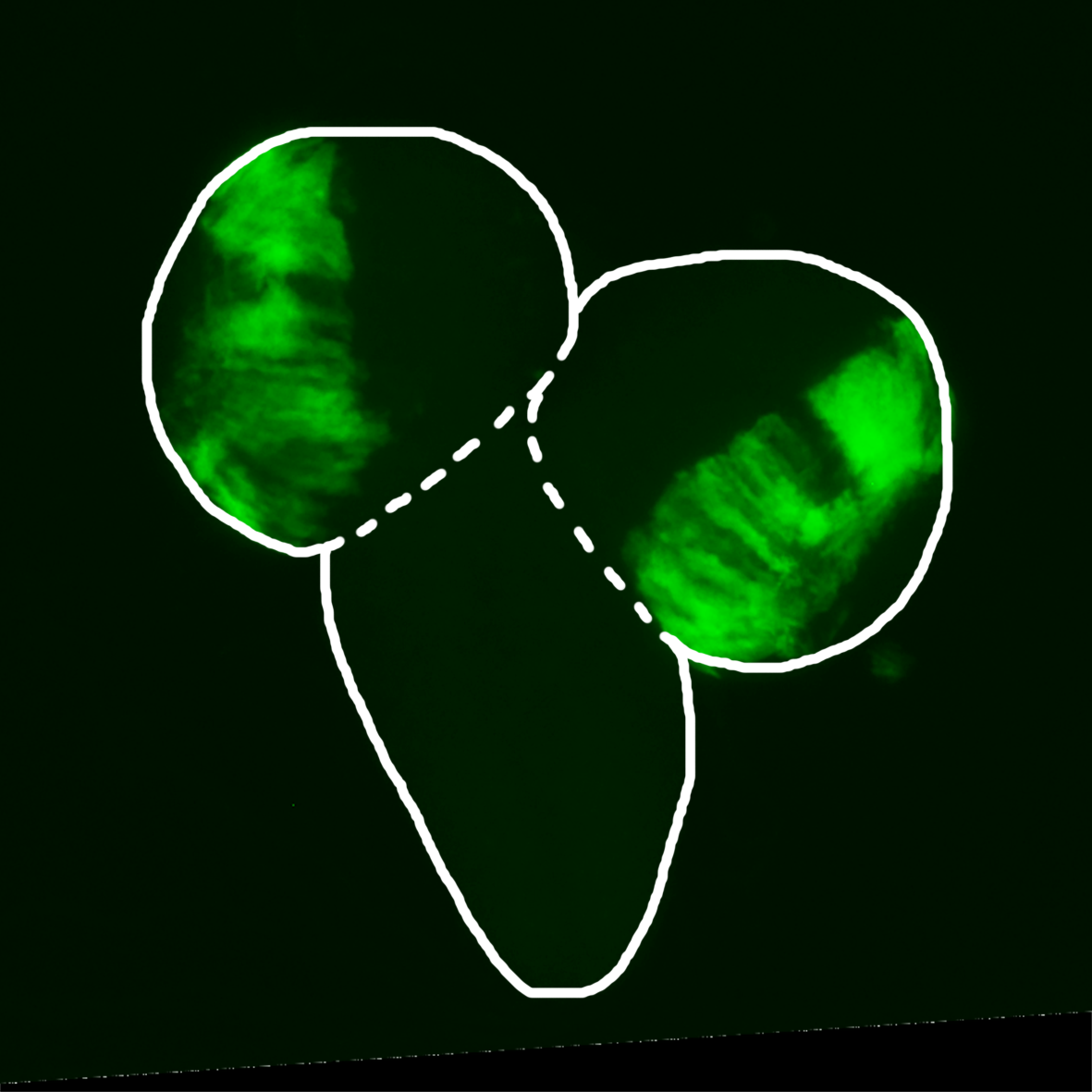

Supplement: Supplementary file 8 — Source data Fig. 4 [file 44318_2025_489_MOESM8_ESM.zip › Figure 4H/3-1 rotated and cut image with border line.tif]

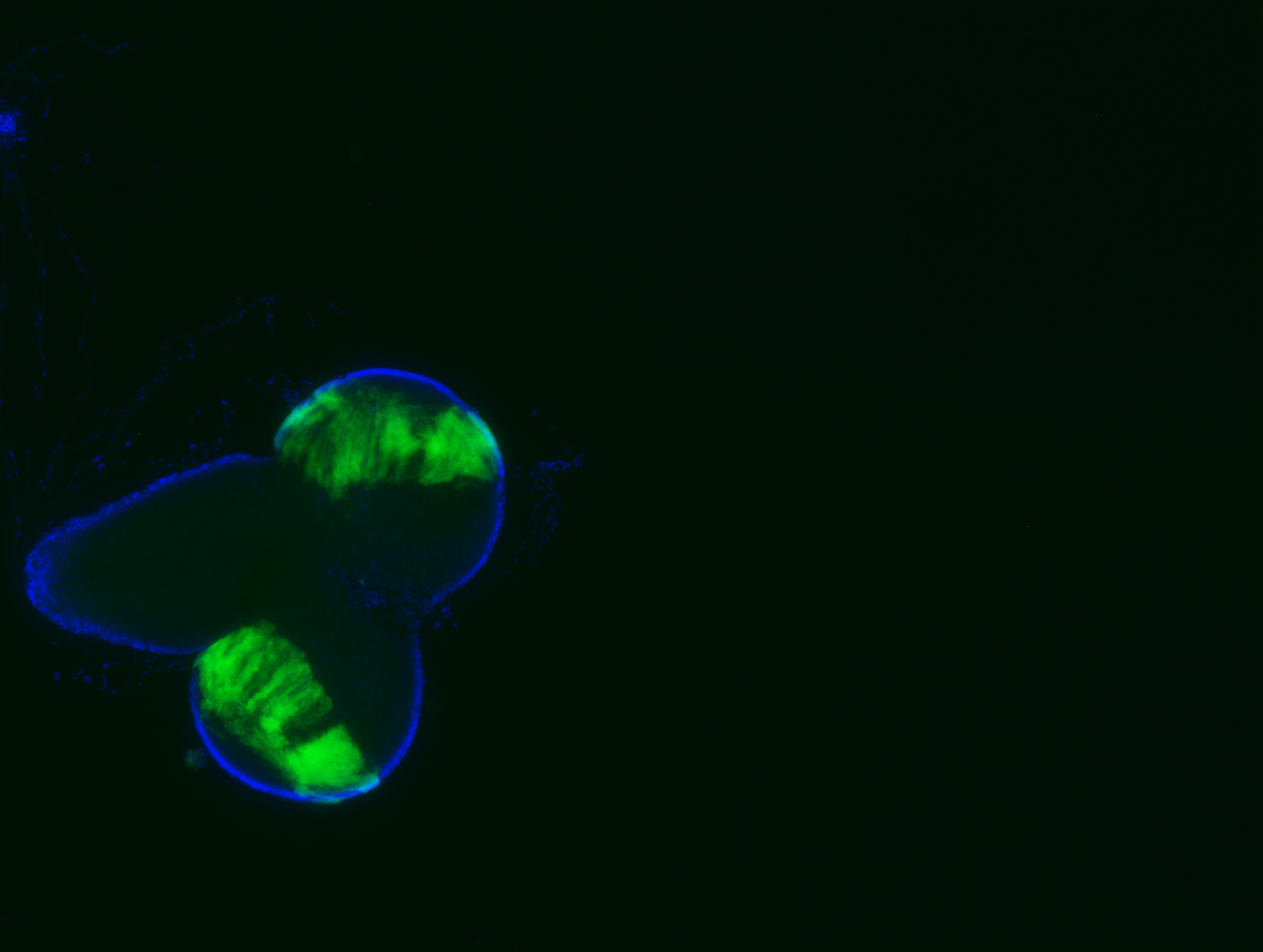

Supplement: Supplementary file 8 — Source data Fig. 4 [file 44318_2025_489_MOESM8_ESM.zip › Figure 4H/3-2 original image.tif]

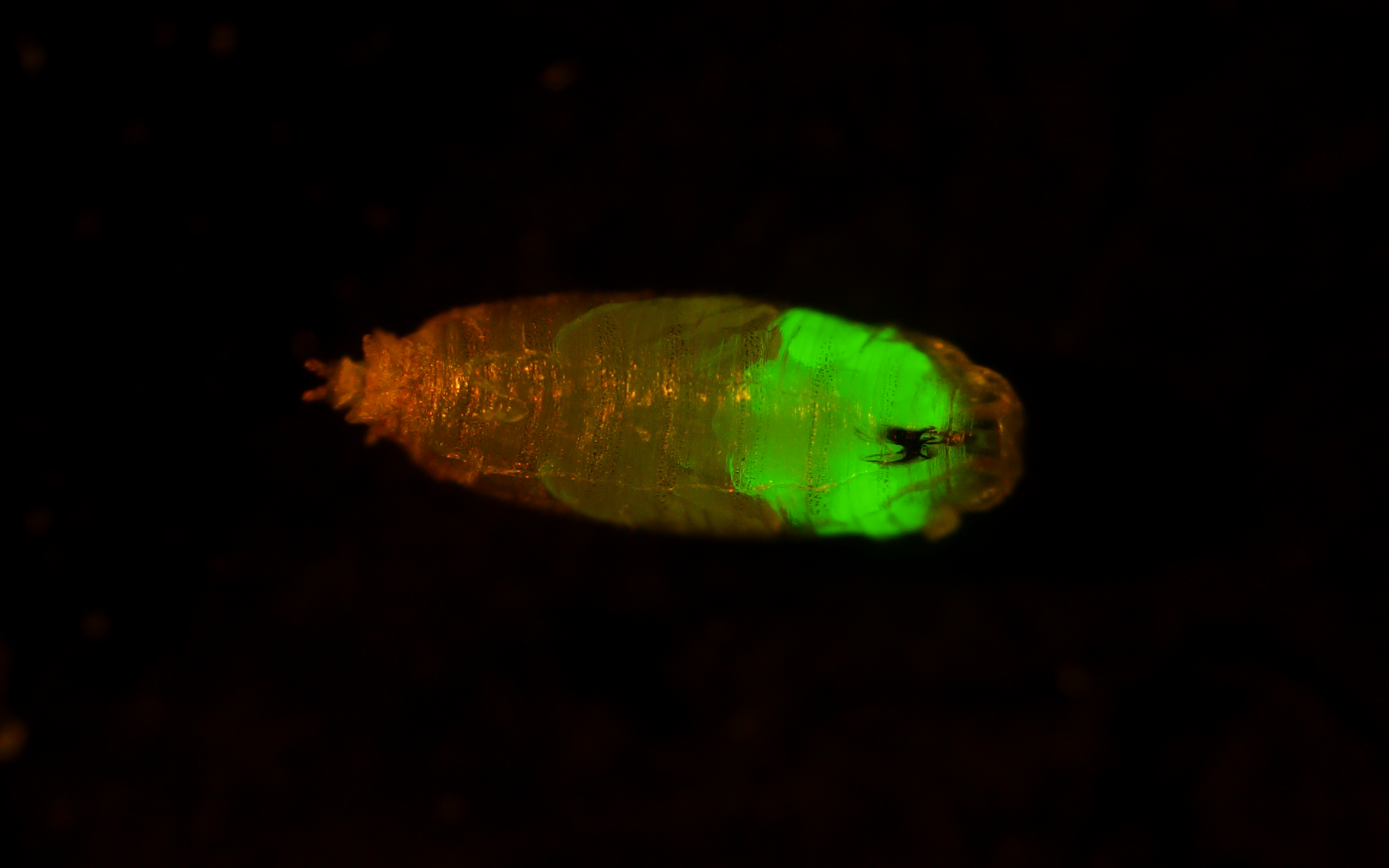

Supplement: Supplementary file 8 — Source data Fig. 4 [file 44318_2025_489_MOESM8_ESM.zip › Figure 4H/4 original image.tif]

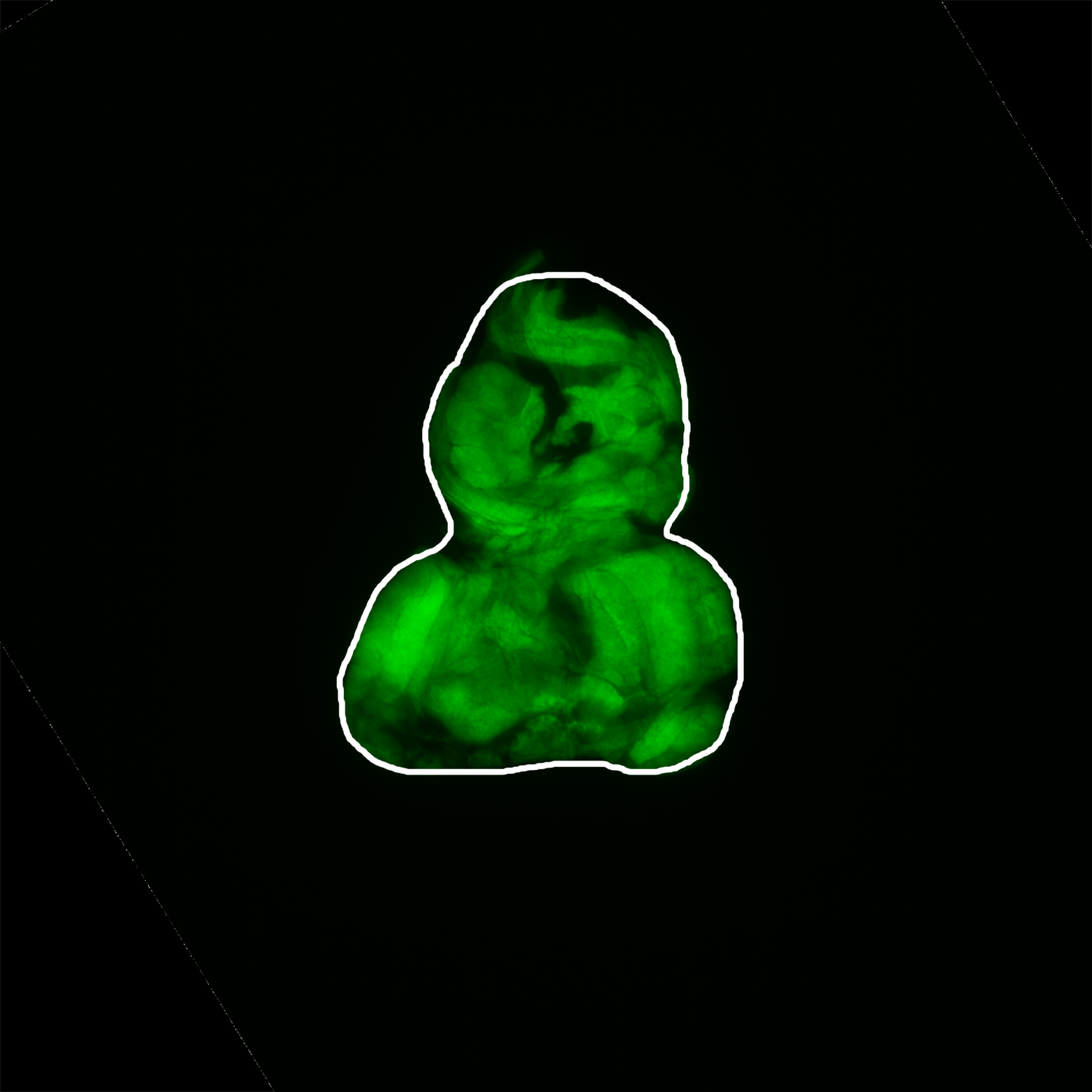

Supplement: Supplementary file 8 — Source data Fig. 4 [file 44318_2025_489_MOESM8_ESM.zip › Figure 4H/5-1 rotated and cut image with border line.tif]

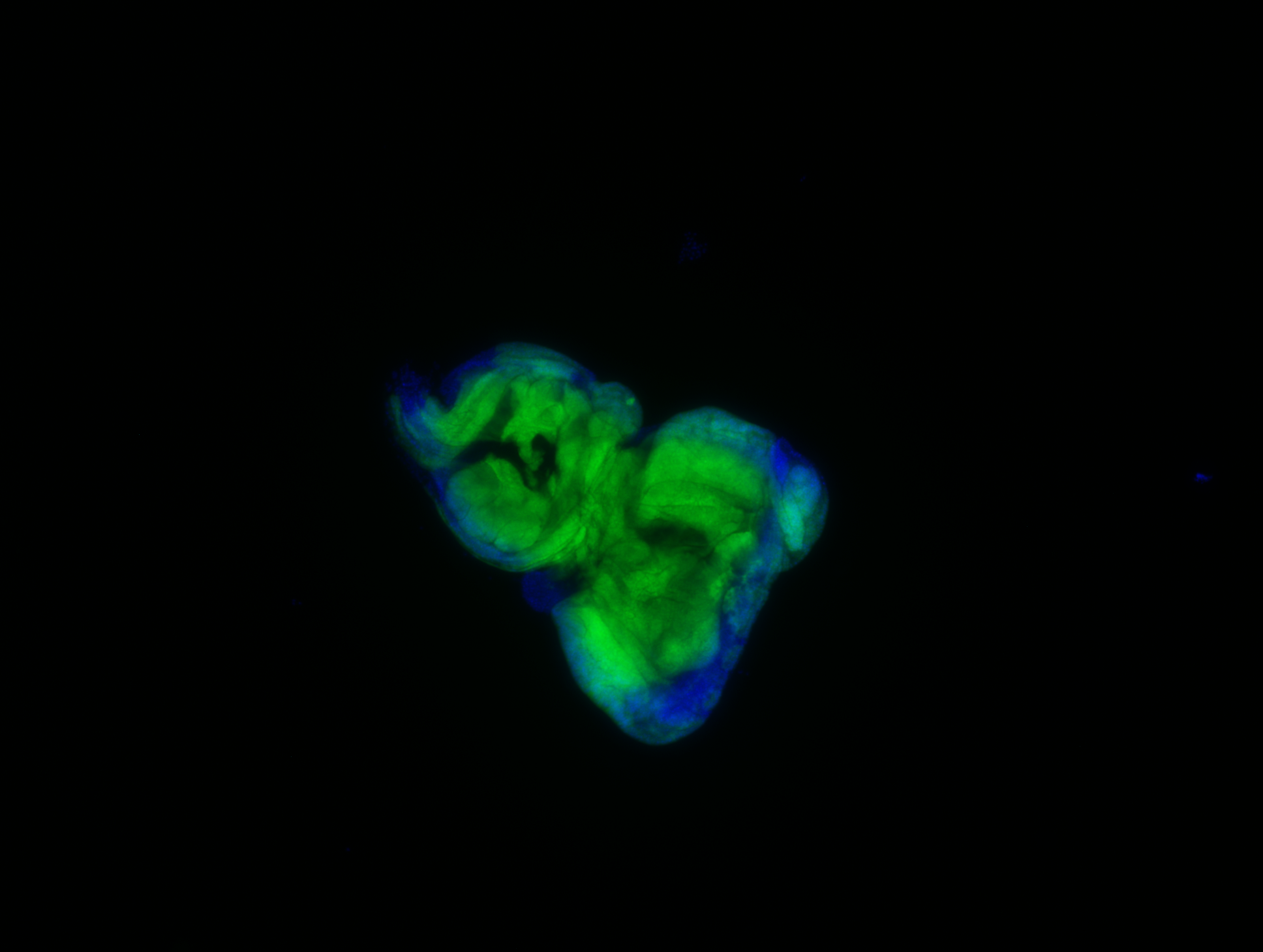

Supplement: Supplementary file 8 — Source data Fig. 4 [file 44318_2025_489_MOESM8_ESM.zip › Figure 4H/5-2 original image.tif]

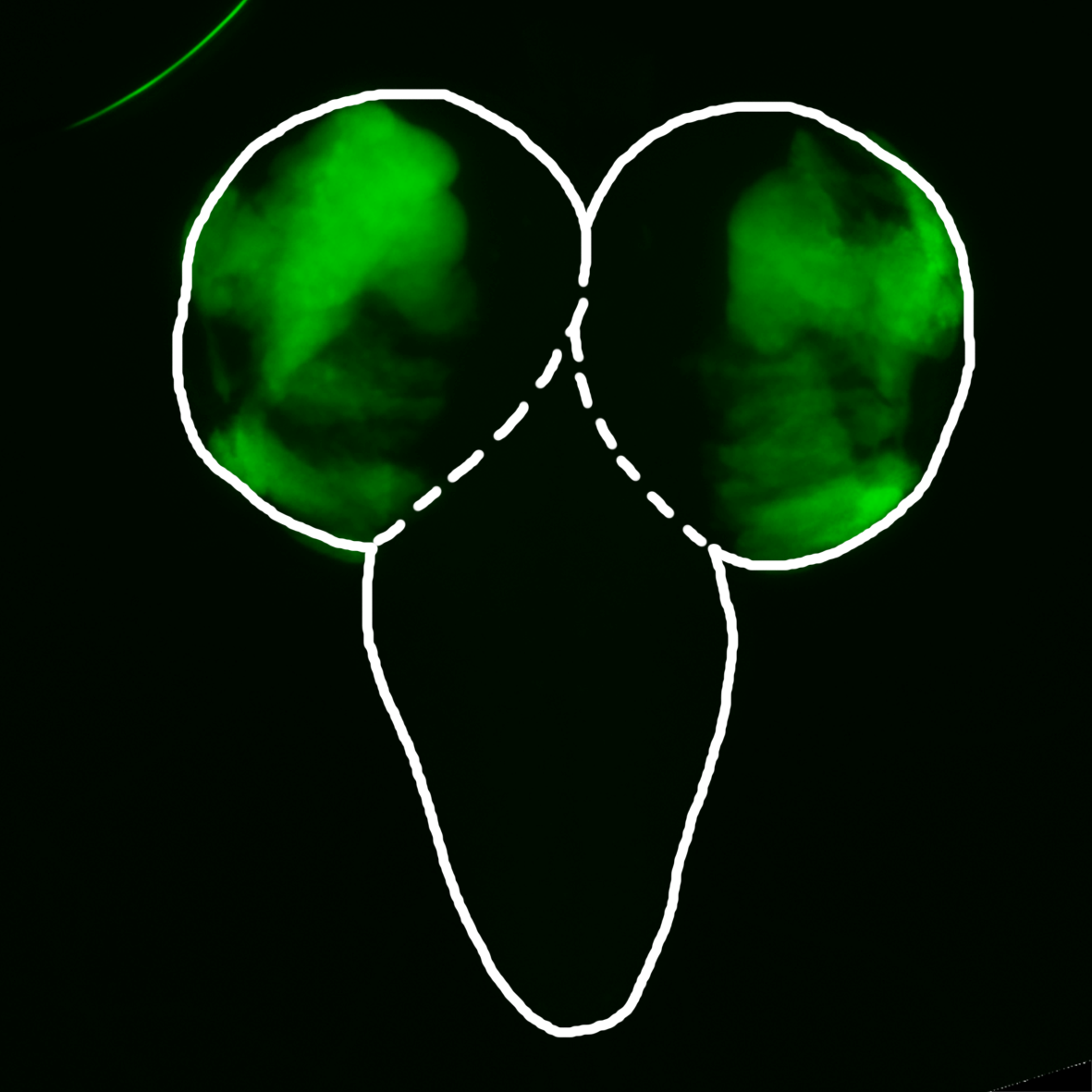

Supplement: Supplementary file 8 — Source data Fig. 4 [file 44318_2025_489_MOESM8_ESM.zip › Figure 4H/6-1 rotated and cut image with border line.tif]

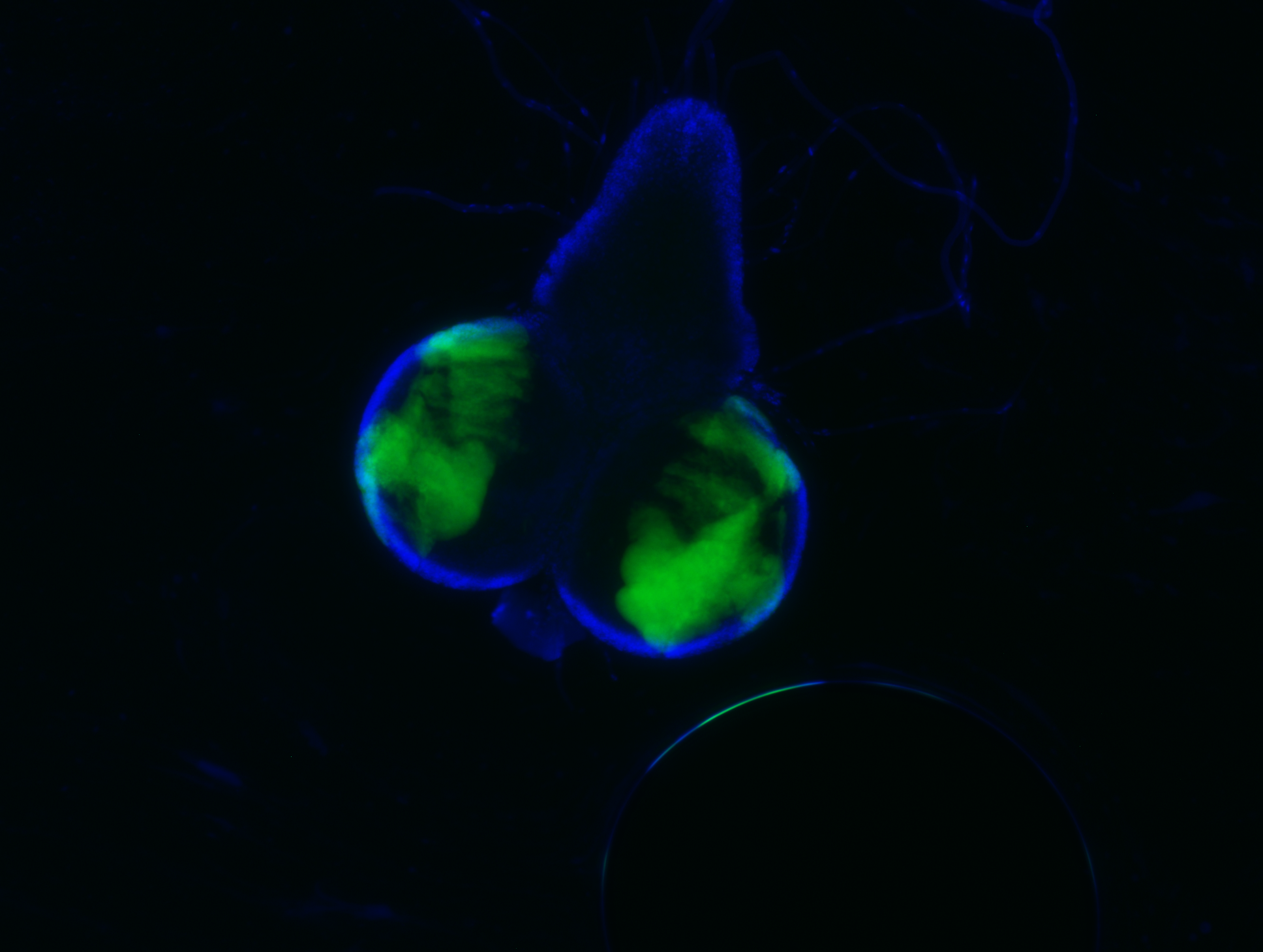

Supplement: Supplementary file 8 — Source data Fig. 4 [file 44318_2025_489_MOESM8_ESM.zip › Figure 4H/6-2 original image.tif]

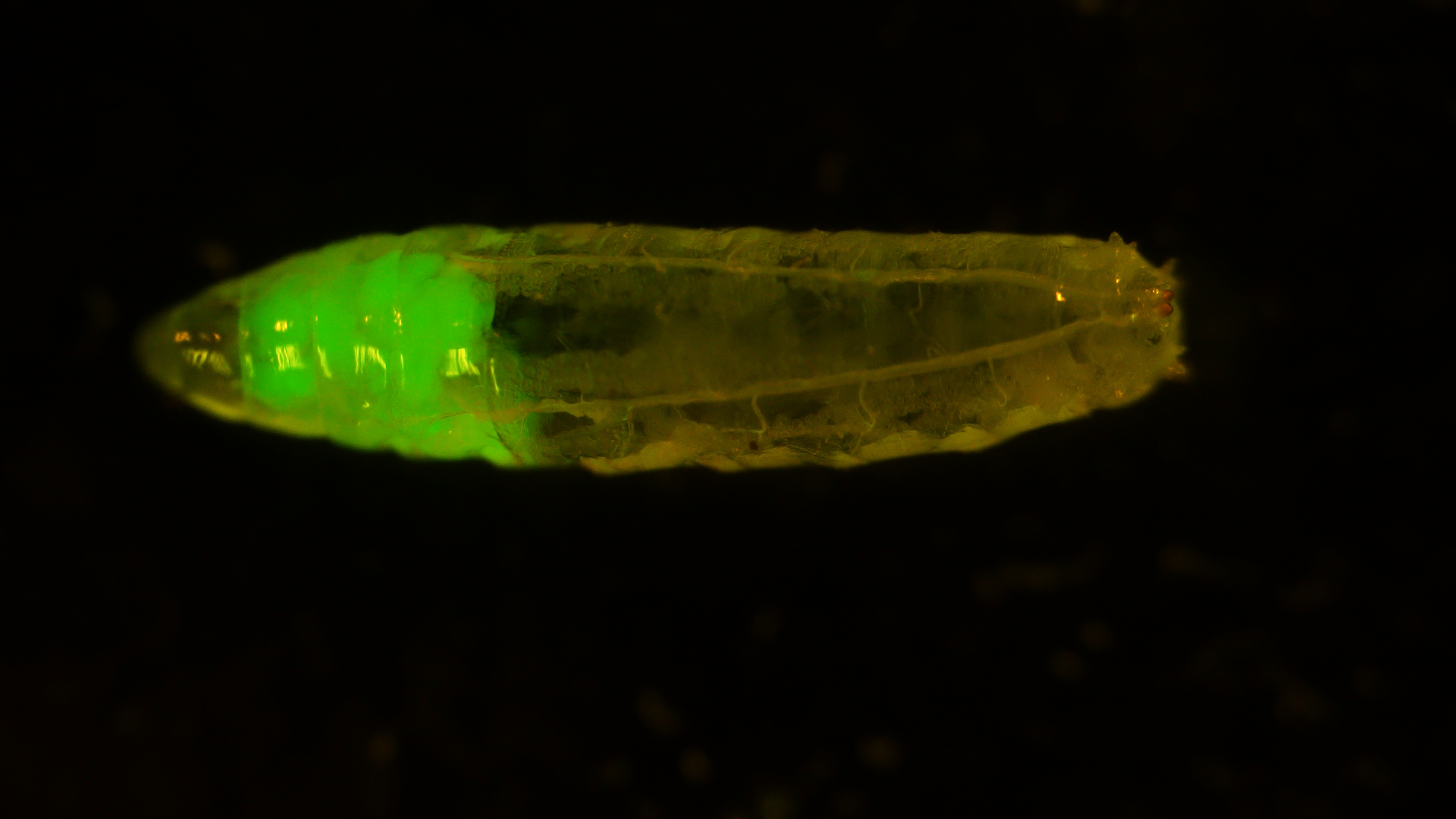

Supplement: Supplementary file 8 — Source data Fig. 4 [file 44318_2025_489_MOESM8_ESM.zip › Figure 4H/7 original image.tif]

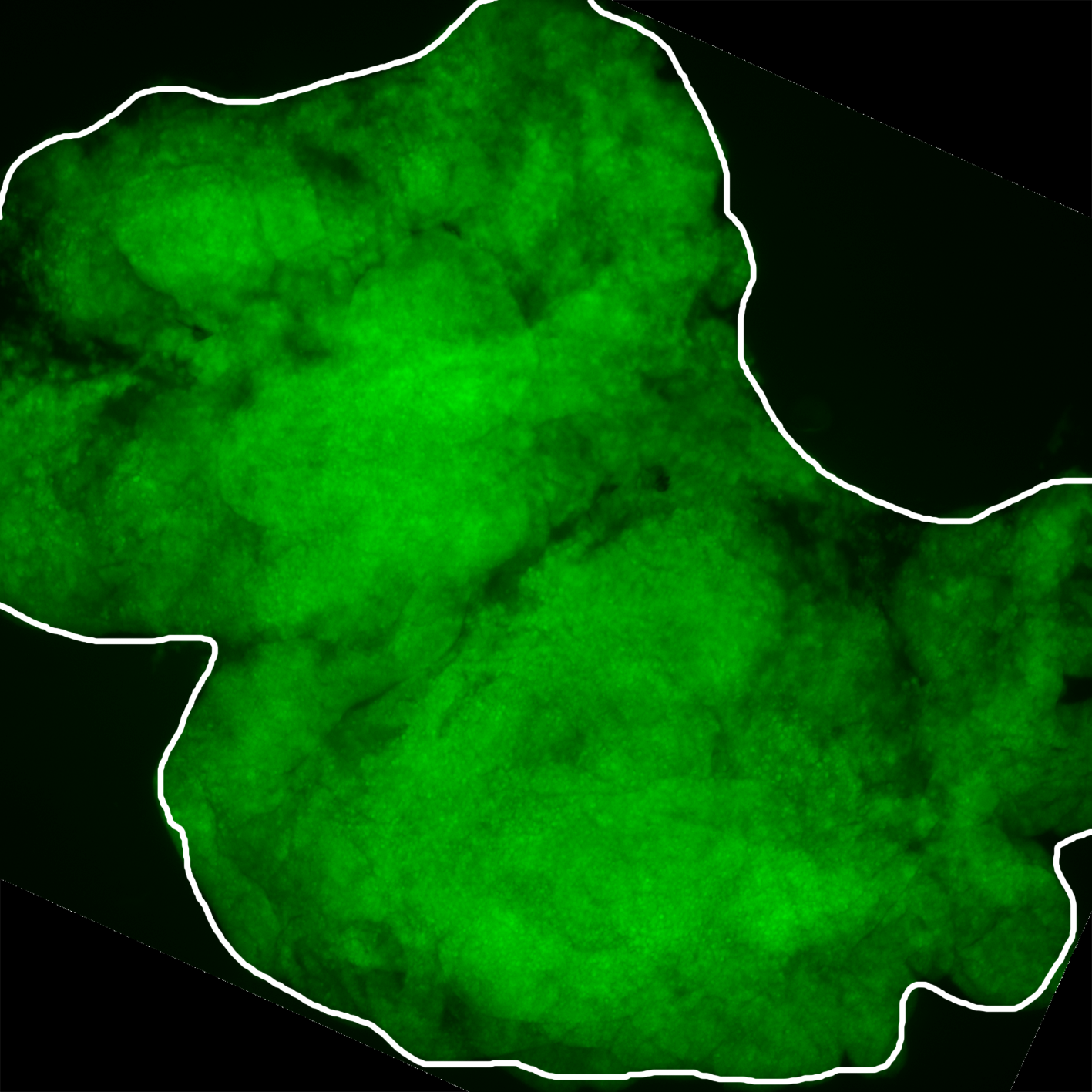

Supplement: Supplementary file 8 — Source data Fig. 4 [file 44318_2025_489_MOESM8_ESM.zip › Figure 4H/8-1 rotated and cut image with border line.tif]

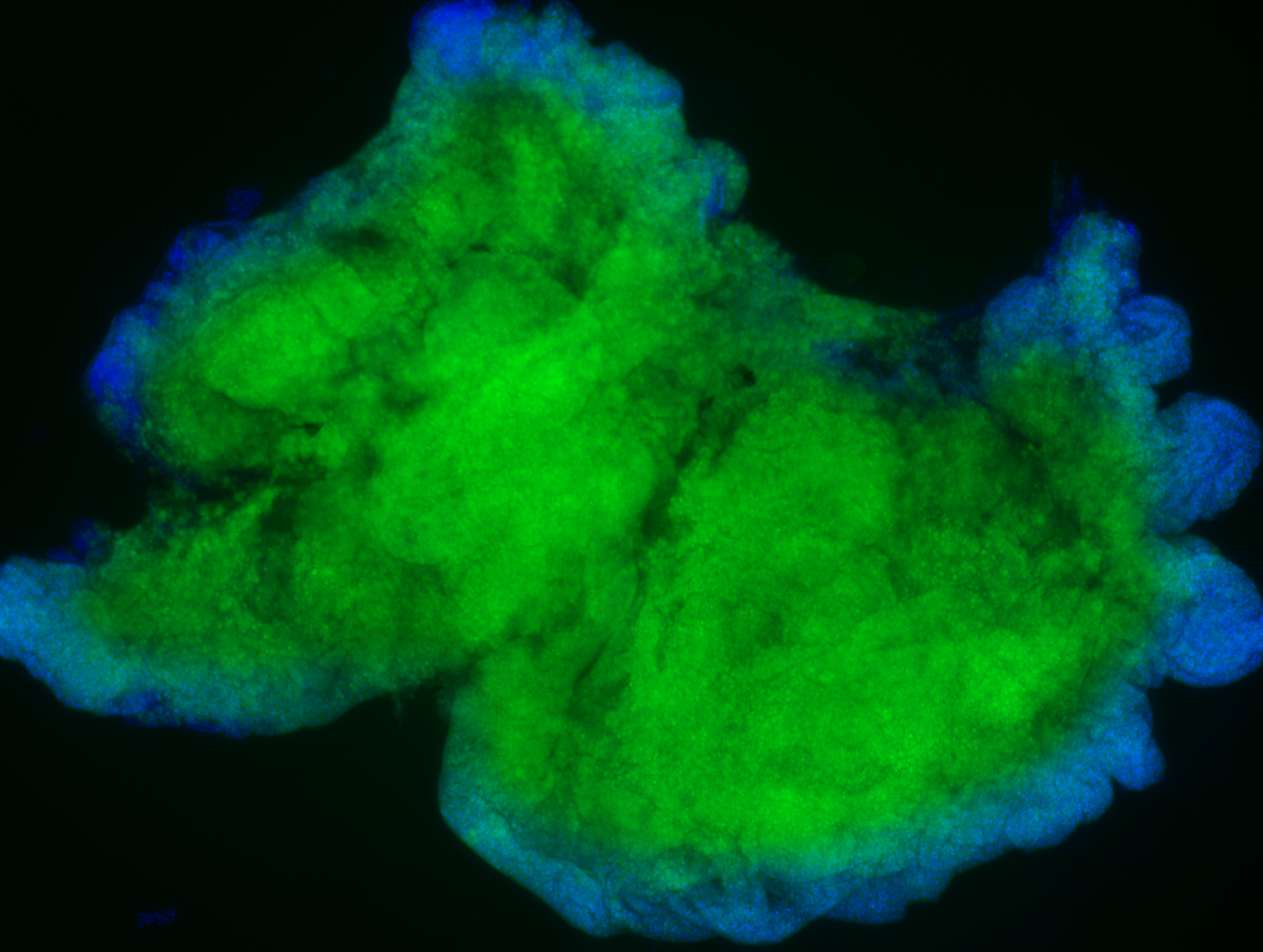

Supplement: Supplementary file 8 — Source data Fig. 4 [file 44318_2025_489_MOESM8_ESM.zip › Figure 4H/8-2 original image.tif]

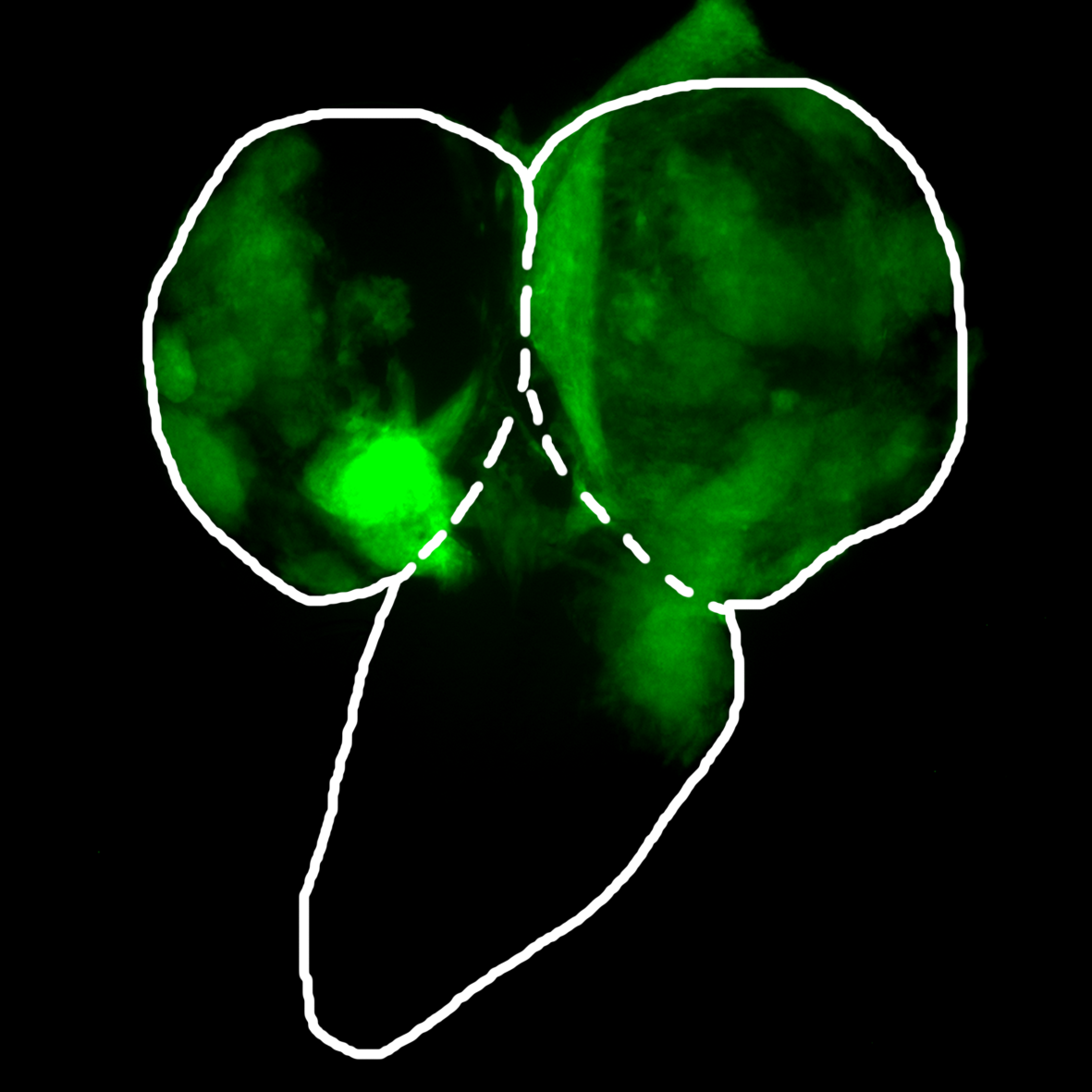

Supplement: Supplementary file 8 — Source data Fig. 4 [file 44318_2025_489_MOESM8_ESM.zip › Figure 4H/9-1 rotated and cut image with border line.tif]

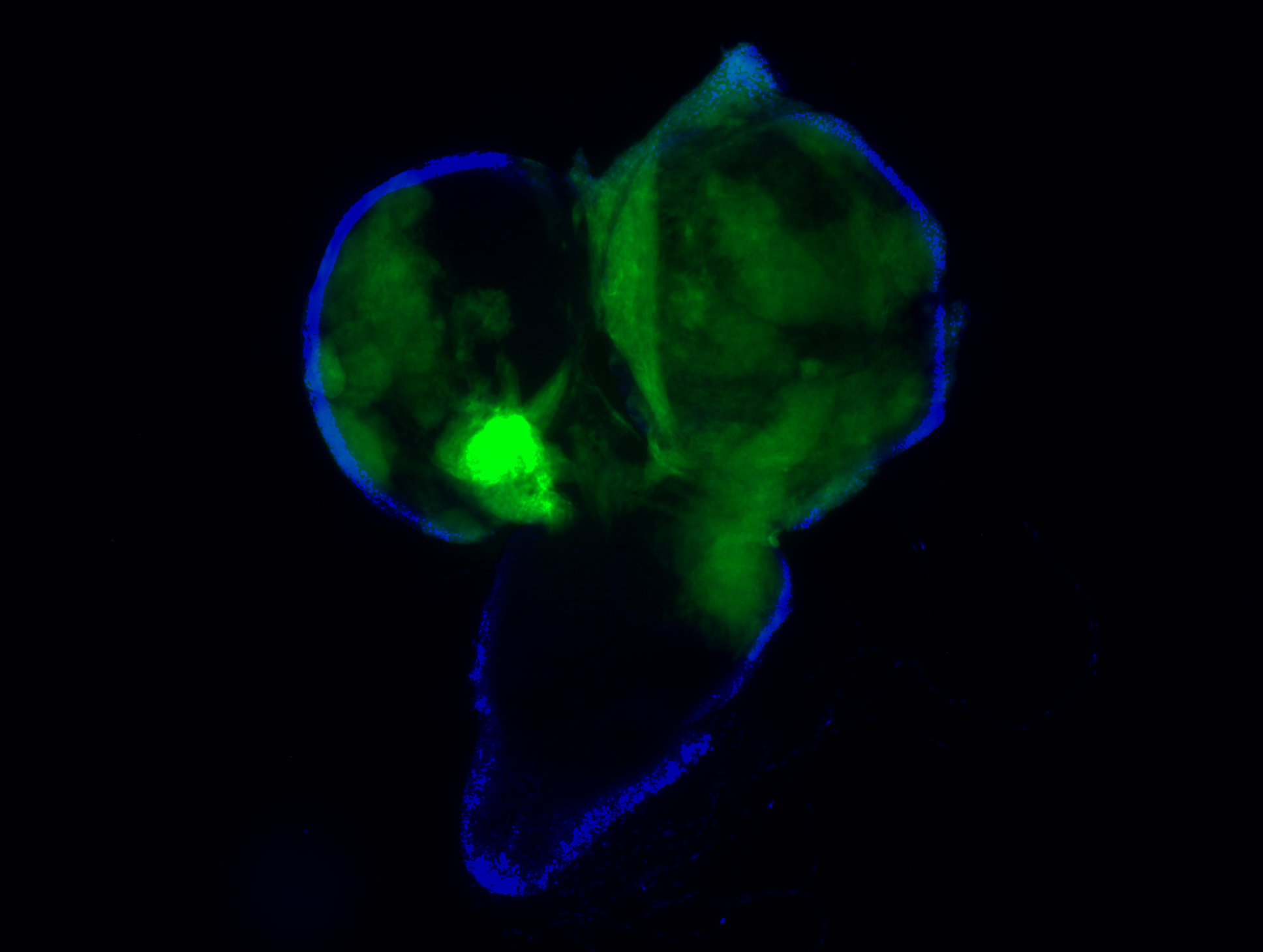

Supplement: Supplementary file 8 — Source data Fig. 4 [file 44318_2025_489_MOESM8_ESM.zip › Figure 4H/9-2 original image.tif]

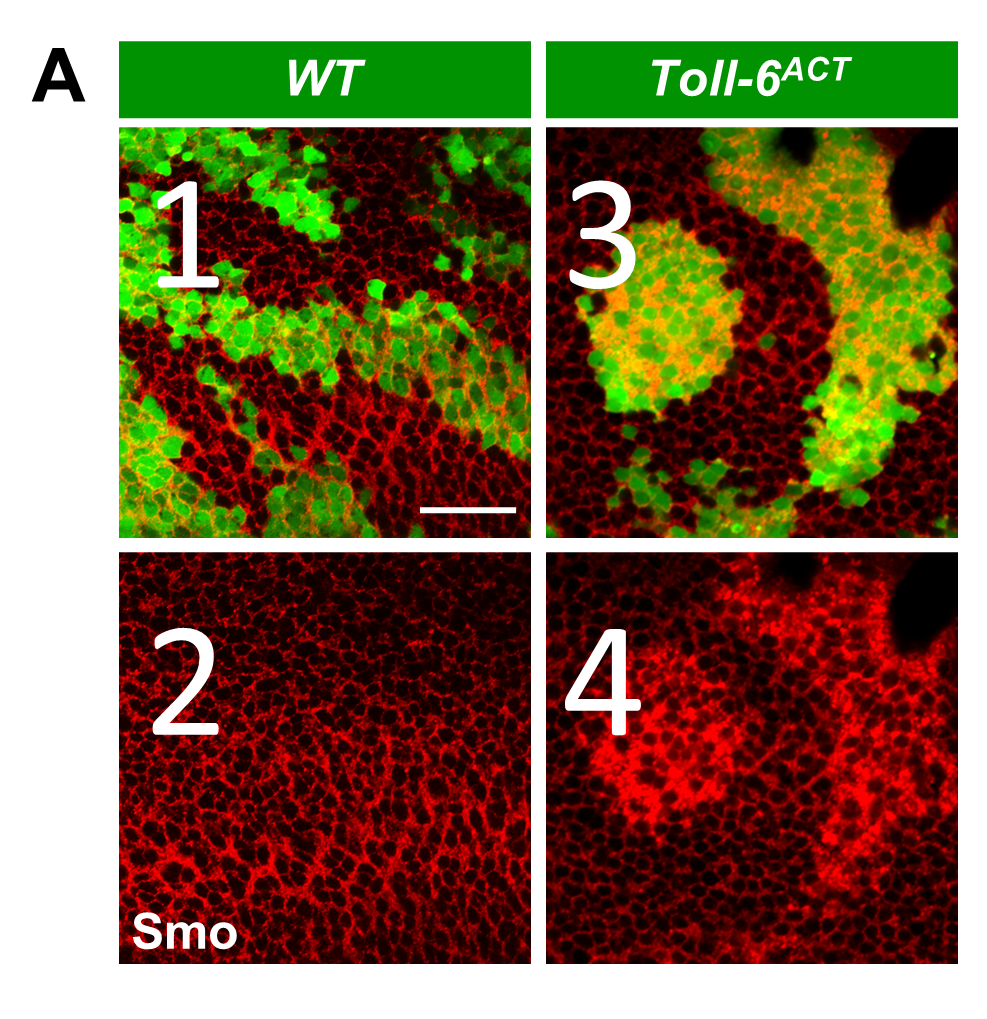

Supplement: Supplementary file 8 — Source data Fig. 4 [file 44318_2025_489_MOESM8_ESM.zip › Figure 4A/0 paper Figure 4A with provided image sequence.tif]

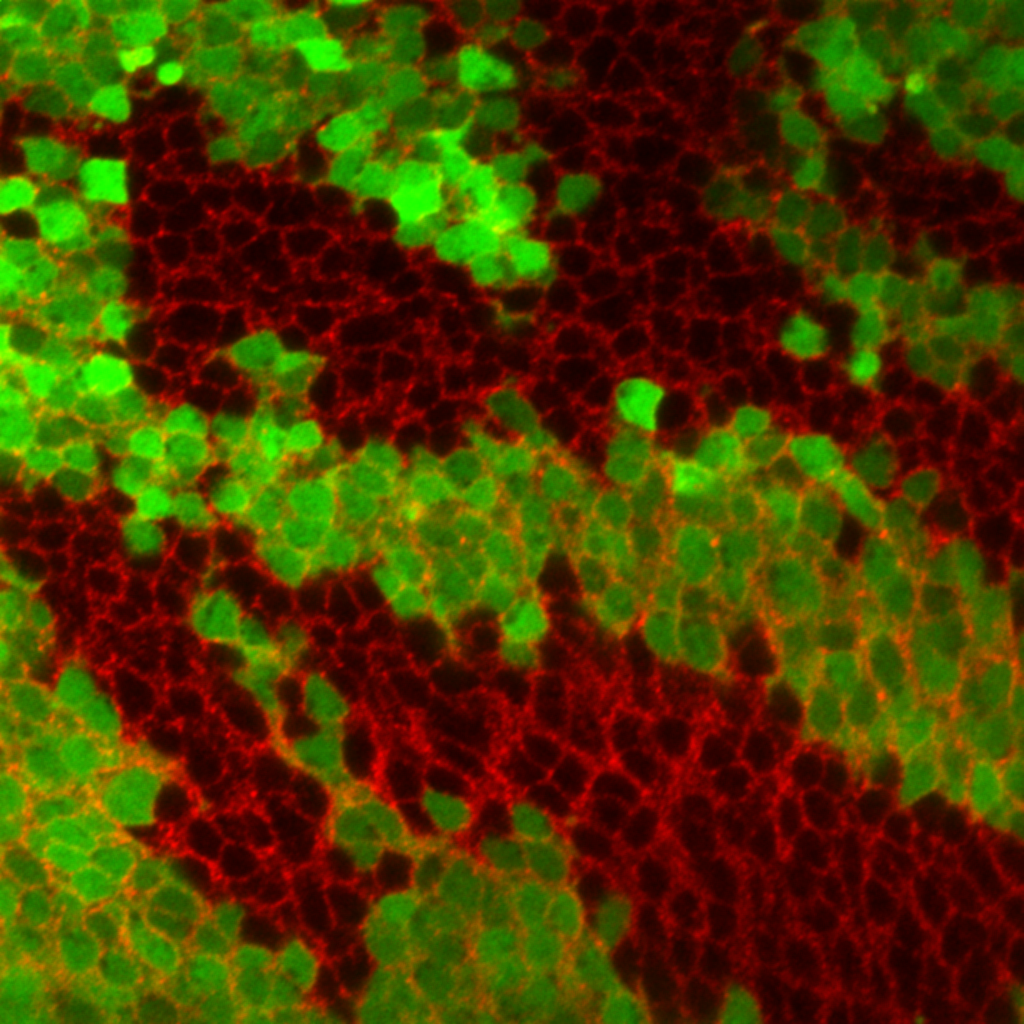

Supplement: Supplementary file 8 — Source data Fig. 4 [file 44318_2025_489_MOESM8_ESM.zip › Figure 4A/1 original image.tif]

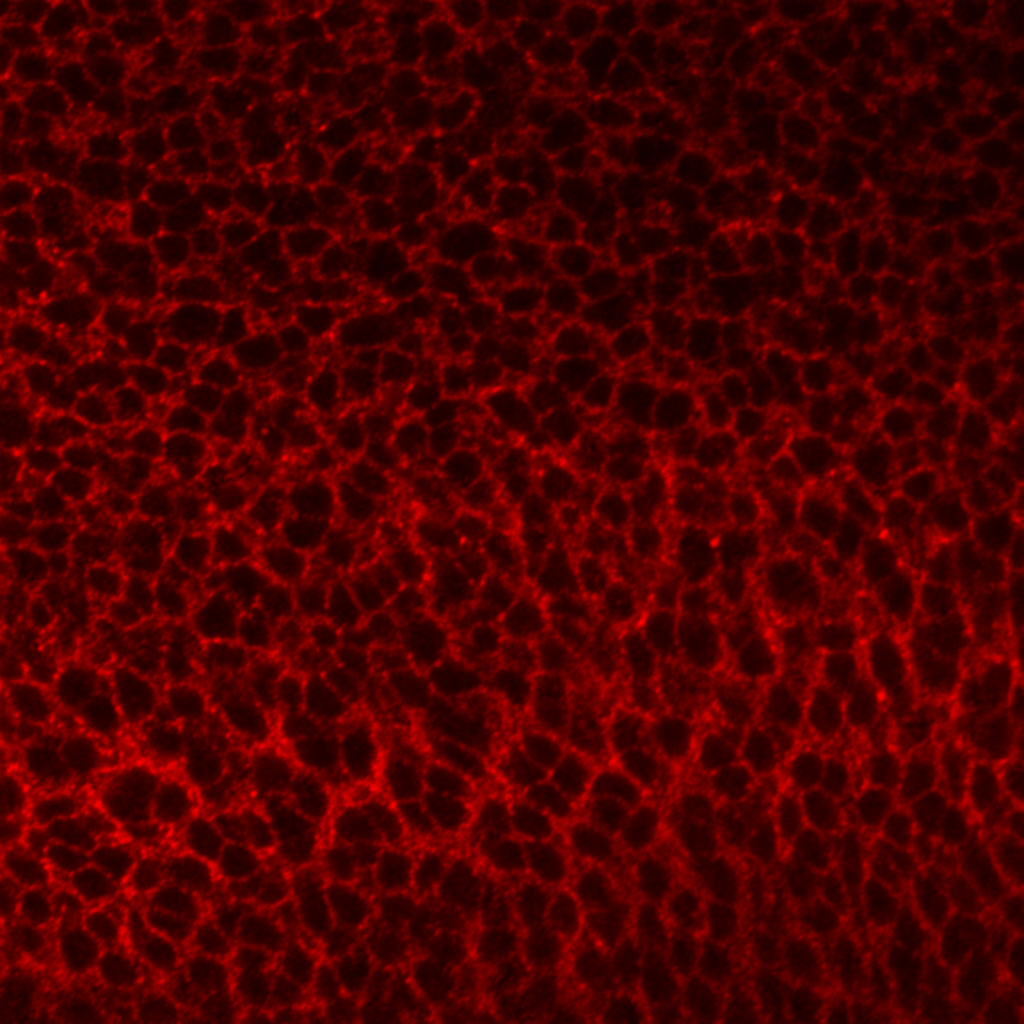

Supplement: Supplementary file 8 — Source data Fig. 4 [file 44318_2025_489_MOESM8_ESM.zip › Figure 4A/2 original image.tif]

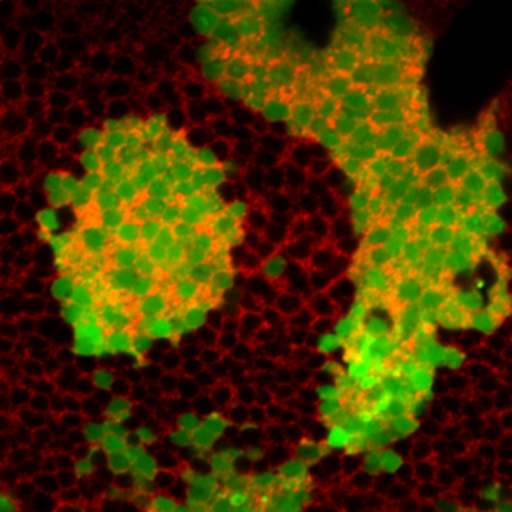

Supplement: Supplementary file 8 — Source data Fig. 4 [file 44318_2025_489_MOESM8_ESM.zip › Figure 4A/3 original image.tif]

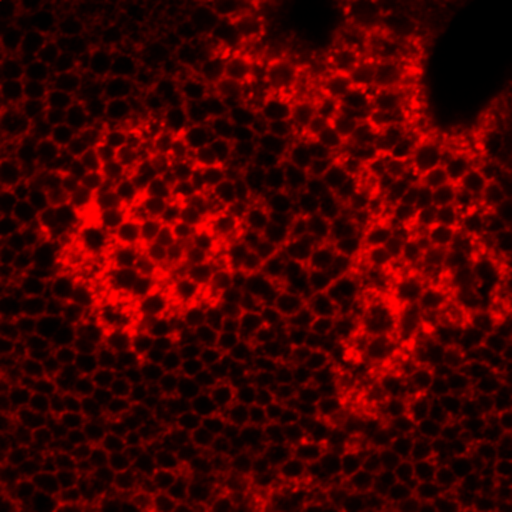

Supplement: Supplementary file 8 — Source data Fig. 4 [file 44318_2025_489_MOESM8_ESM.zip › Figure 4A/4 original image.tif]

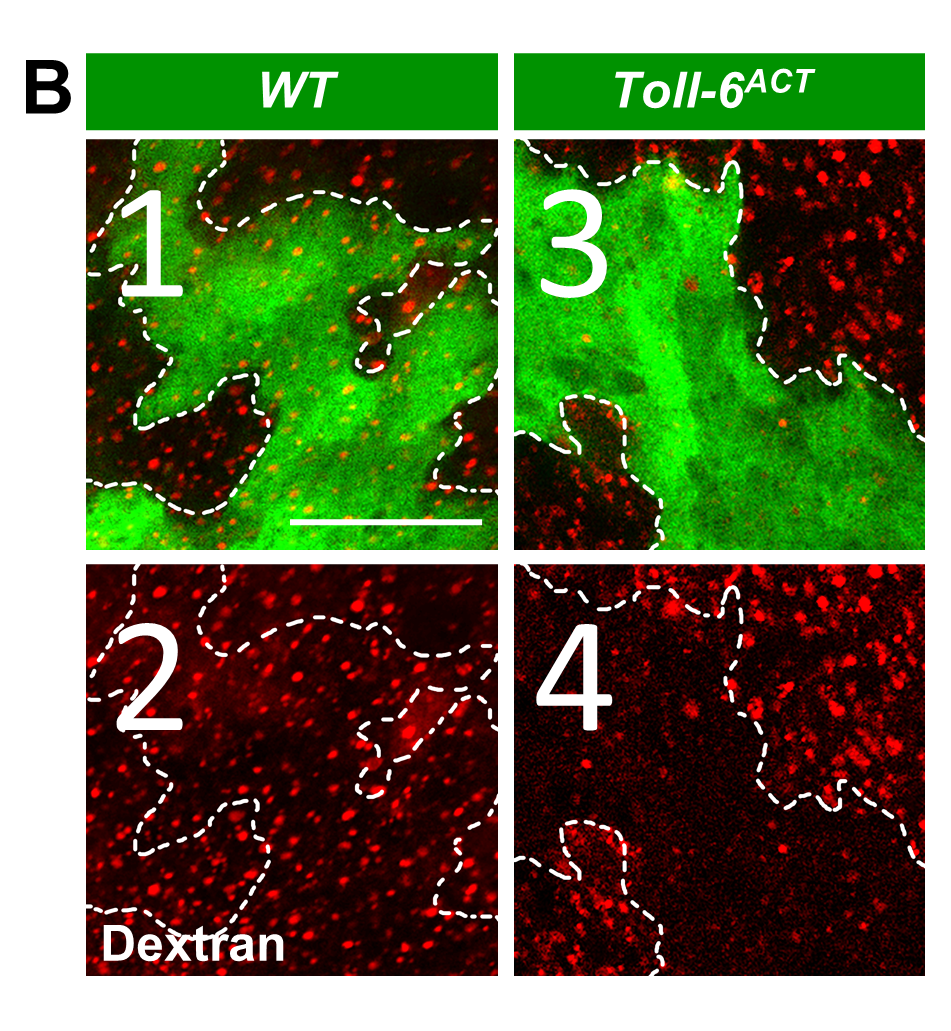

Supplement: Supplementary file 8 — Source data Fig. 4 [file 44318_2025_489_MOESM8_ESM.zip › Figure 4B/0 paper Figure 4B with provided image sequence.tif]

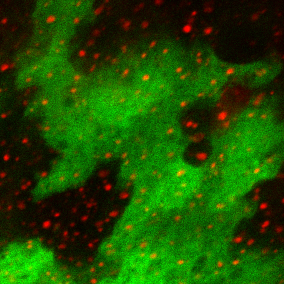

Supplement: Supplementary file 8 — Source data Fig. 4 [file 44318_2025_489_MOESM8_ESM.zip › Figure 4B/1 original image.tif]

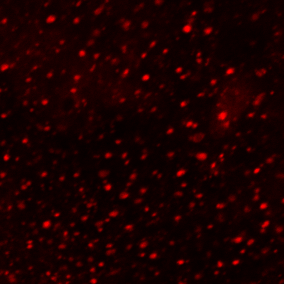

Supplement: Supplementary file 8 — Source data Fig. 4 [file 44318_2025_489_MOESM8_ESM.zip › Figure 4B/2 original image.tif]

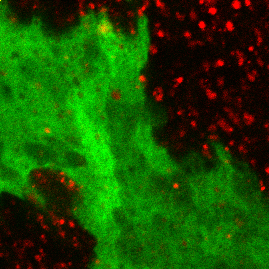

Supplement: Supplementary file 8 — Source data Fig. 4 [file 44318_2025_489_MOESM8_ESM.zip › Figure 4B/3 original image.tif]

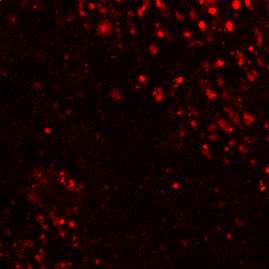

Supplement: Supplementary file 8 — Source data Fig. 4 [file 44318_2025_489_MOESM8_ESM.zip › Figure 4B/4 original image.tif]

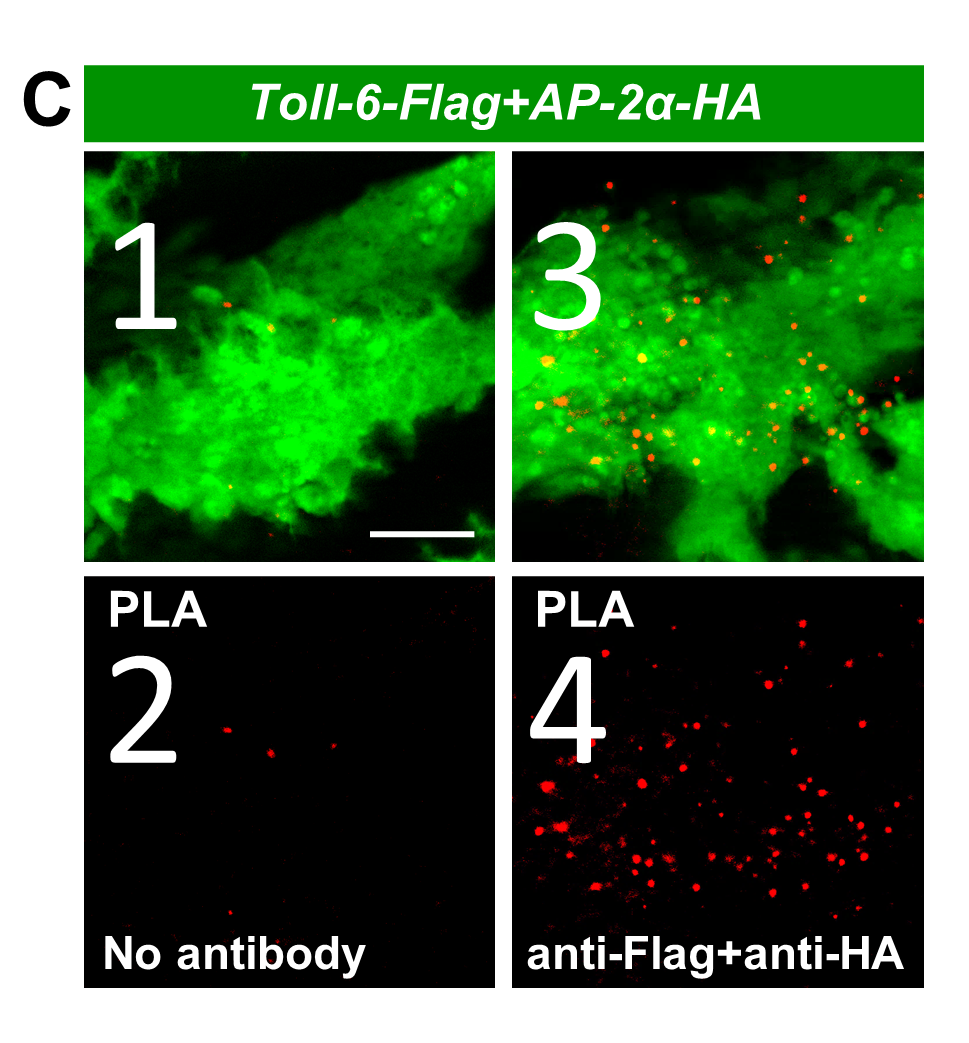

Supplement: Supplementary file 8 — Source data Fig. 4 [file 44318_2025_489_MOESM8_ESM.zip › Figure 4C/0 paper Figure 4C with provided image sequence.tif]

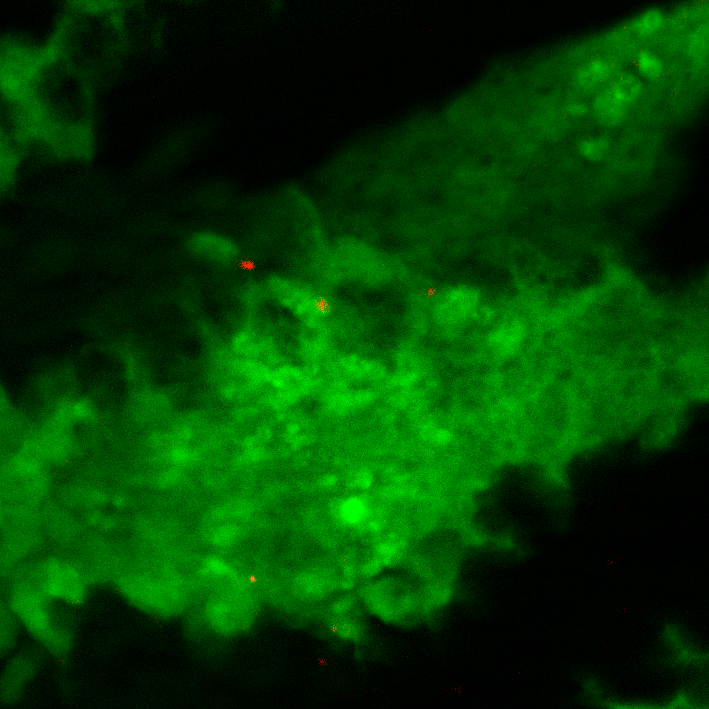

Supplement: Supplementary file 8 — Source data Fig. 4 [file 44318_2025_489_MOESM8_ESM.zip › Figure 4C/1-1 rotated and cut image.tif]

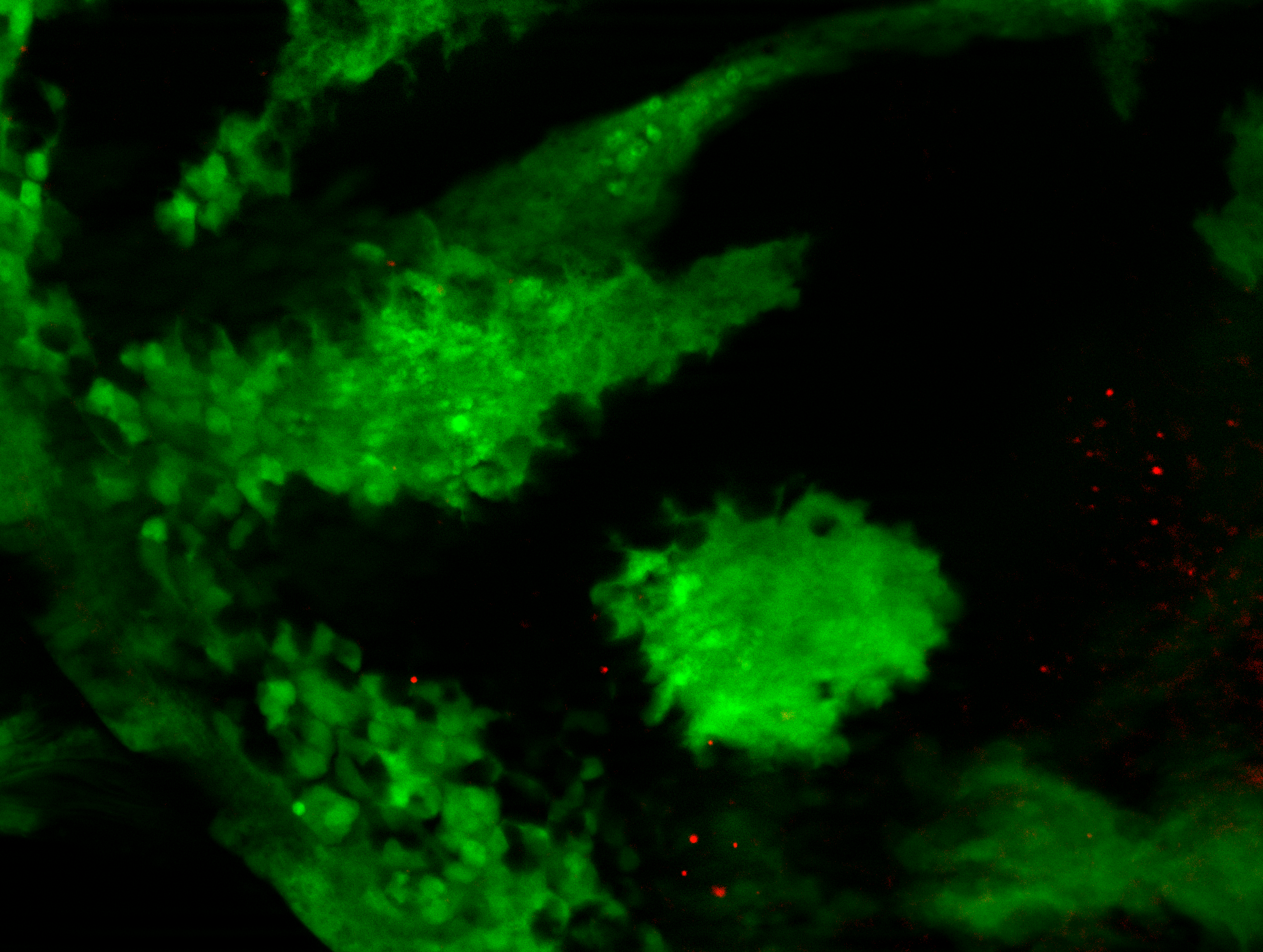

Supplement: Supplementary file 8 — Source data Fig. 4 [file 44318_2025_489_MOESM8_ESM.zip › Figure 4C/1-2 original image.tif]
