# Supplementary figures and images for: Adipose tissue-secreted Spz5 promotes distal tumor progression via Toll-6-mediated Hh pathway activation in Drosophila (part 4 of 5)
Source: EMBO J. 2025 Jun 23;44(15):4301–30. doi: 10.1038/s44318-025-00489-y (PMC12317064; doi:10.1038/s44318-025-00489-y)

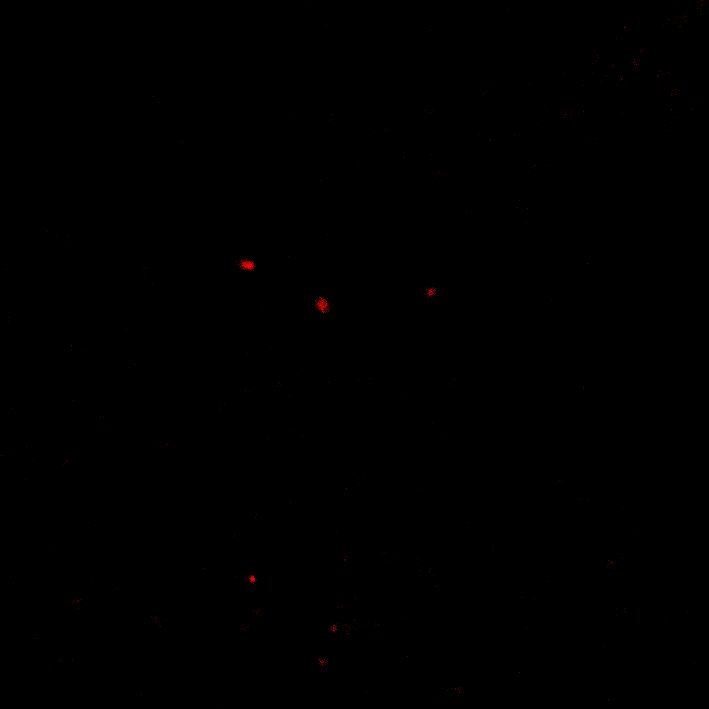

Supplement: Supplementary file 8 — Source data Fig. 4 [file 44318_2025_489_MOESM8_ESM.zip › Figure 4C/2-1 rotated and cut image.tif]

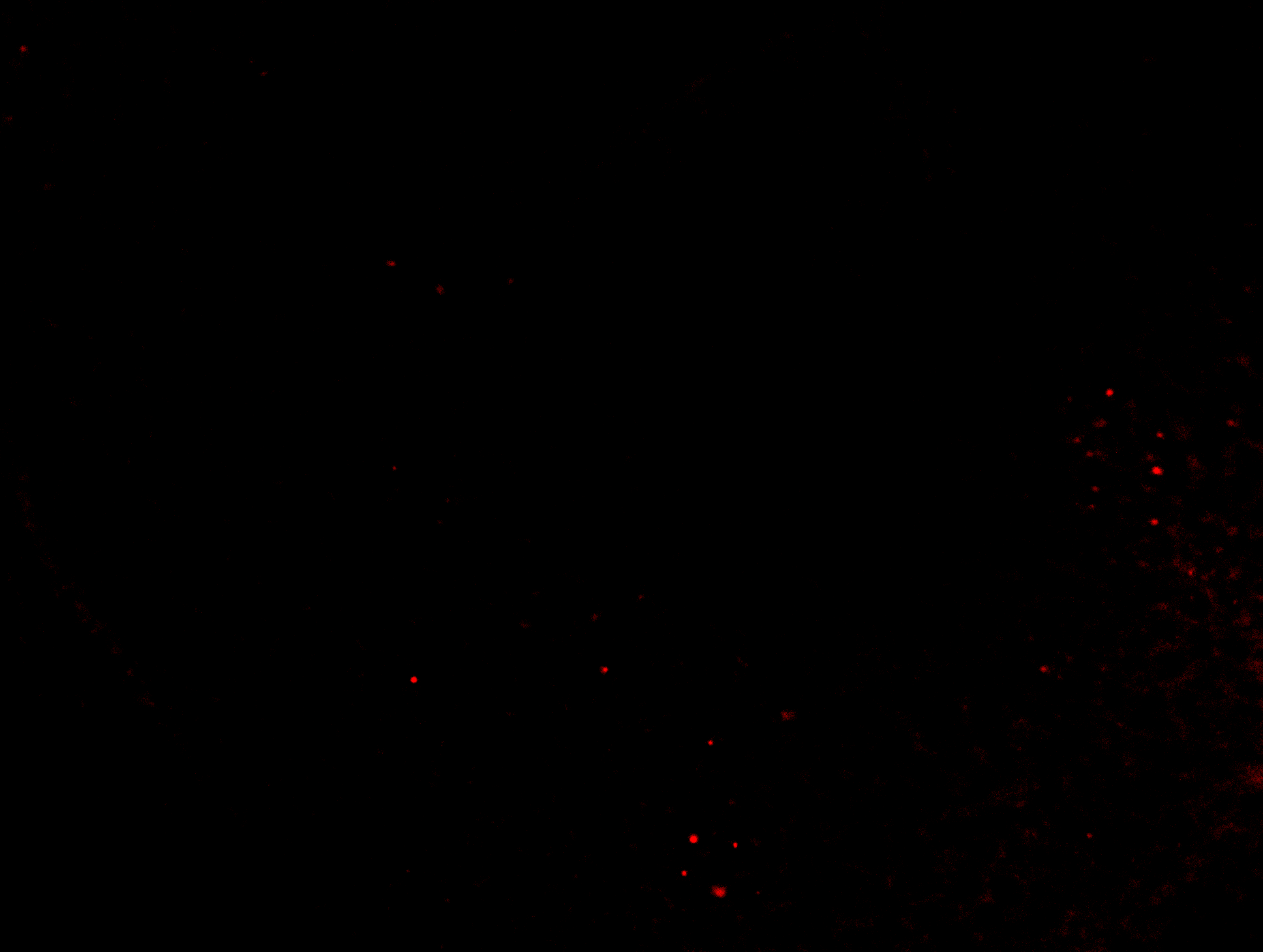

Supplement: Supplementary file 8 — Source data Fig. 4 [file 44318_2025_489_MOESM8_ESM.zip › Figure 4C/2-2 original image.tif]

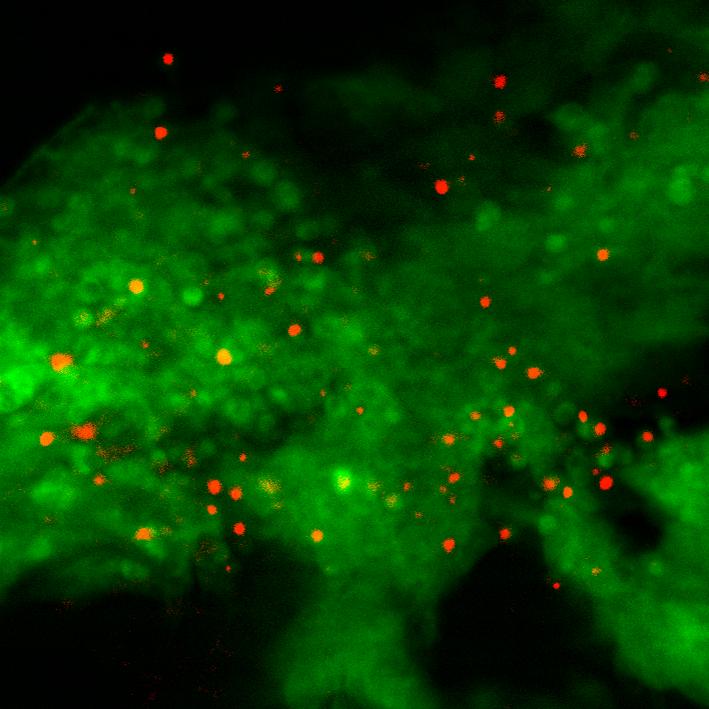

Supplement: Supplementary file 8 — Source data Fig. 4 [file 44318_2025_489_MOESM8_ESM.zip › Figure 4C/3-1 rotated and cut image.tif]

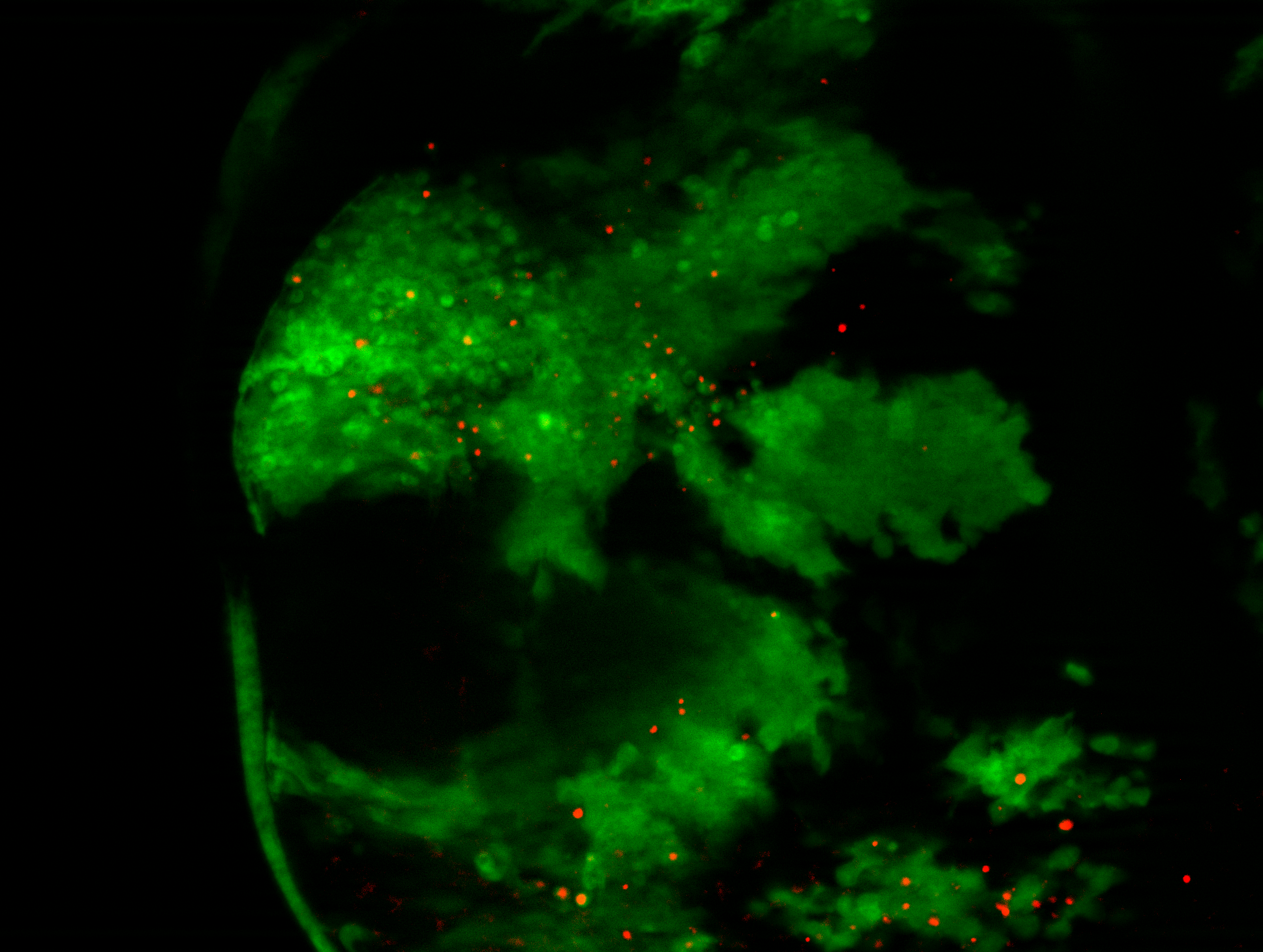

Supplement: Supplementary file 8 — Source data Fig. 4 [file 44318_2025_489_MOESM8_ESM.zip › Figure 4C/3-2 original image.tif]

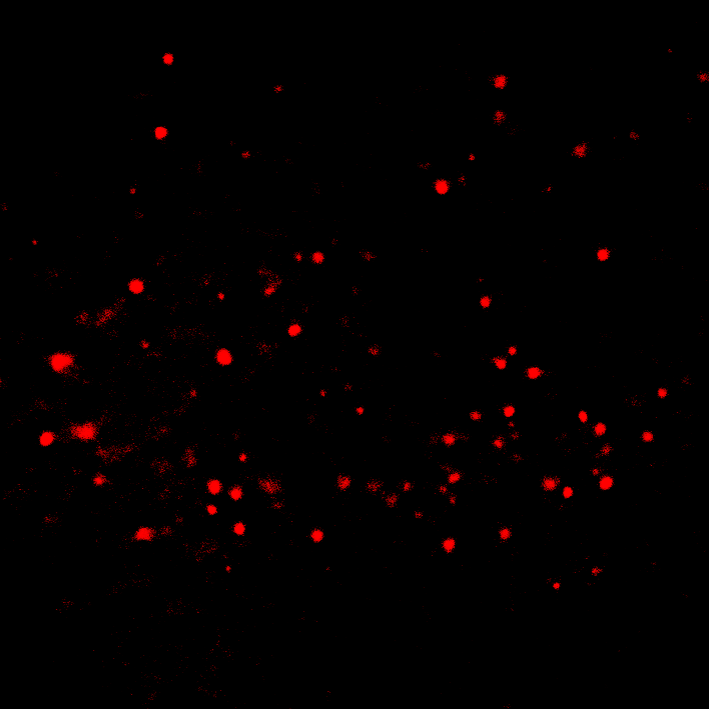

Supplement: Supplementary file 8 — Source data Fig. 4 [file 44318_2025_489_MOESM8_ESM.zip › Figure 4C/4-1 rotated and cut image.tif]

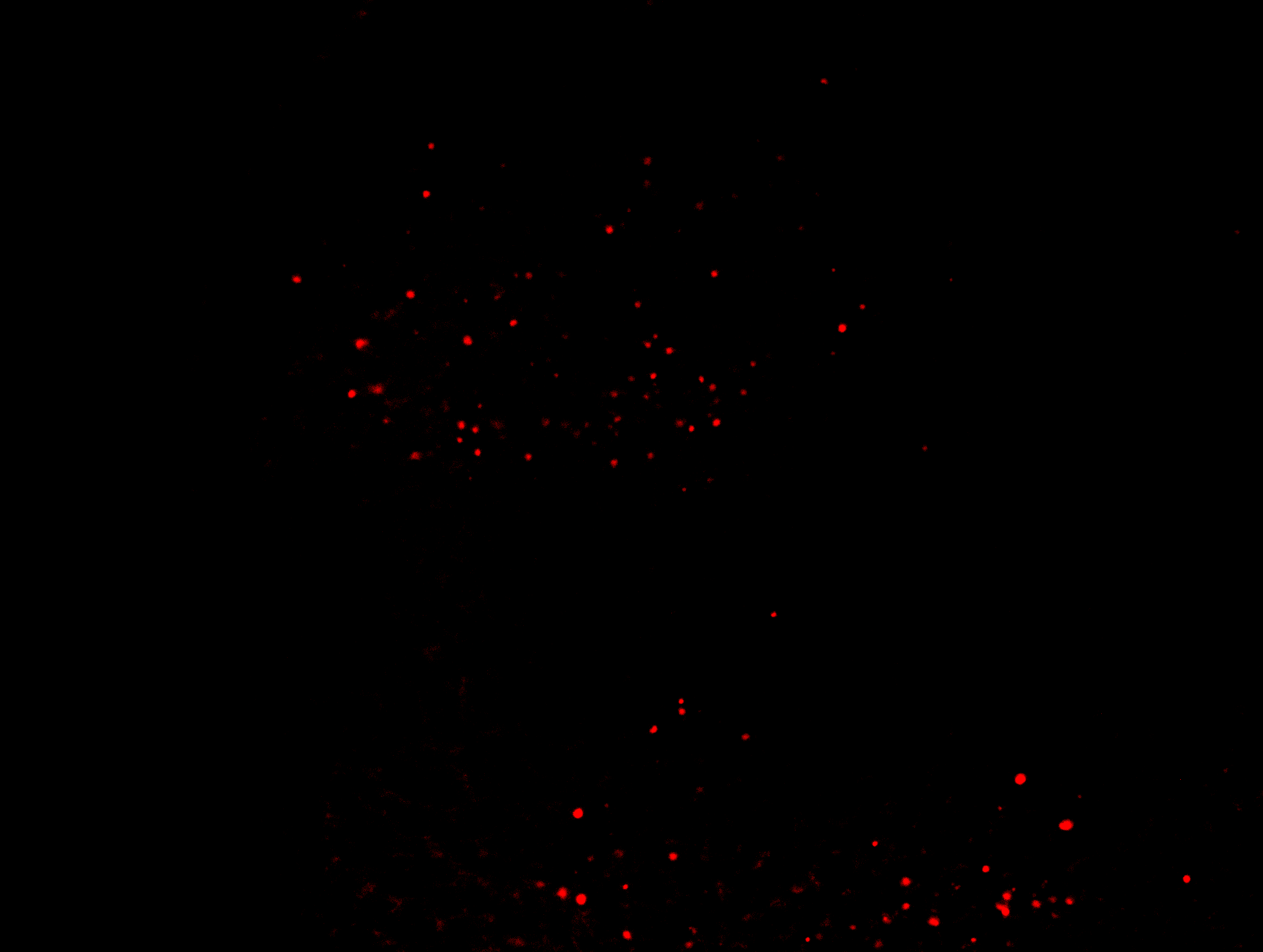

Supplement: Supplementary file 8 — Source data Fig. 4 [file 44318_2025_489_MOESM8_ESM.zip › Figure 4C/4-2 original image.tif]

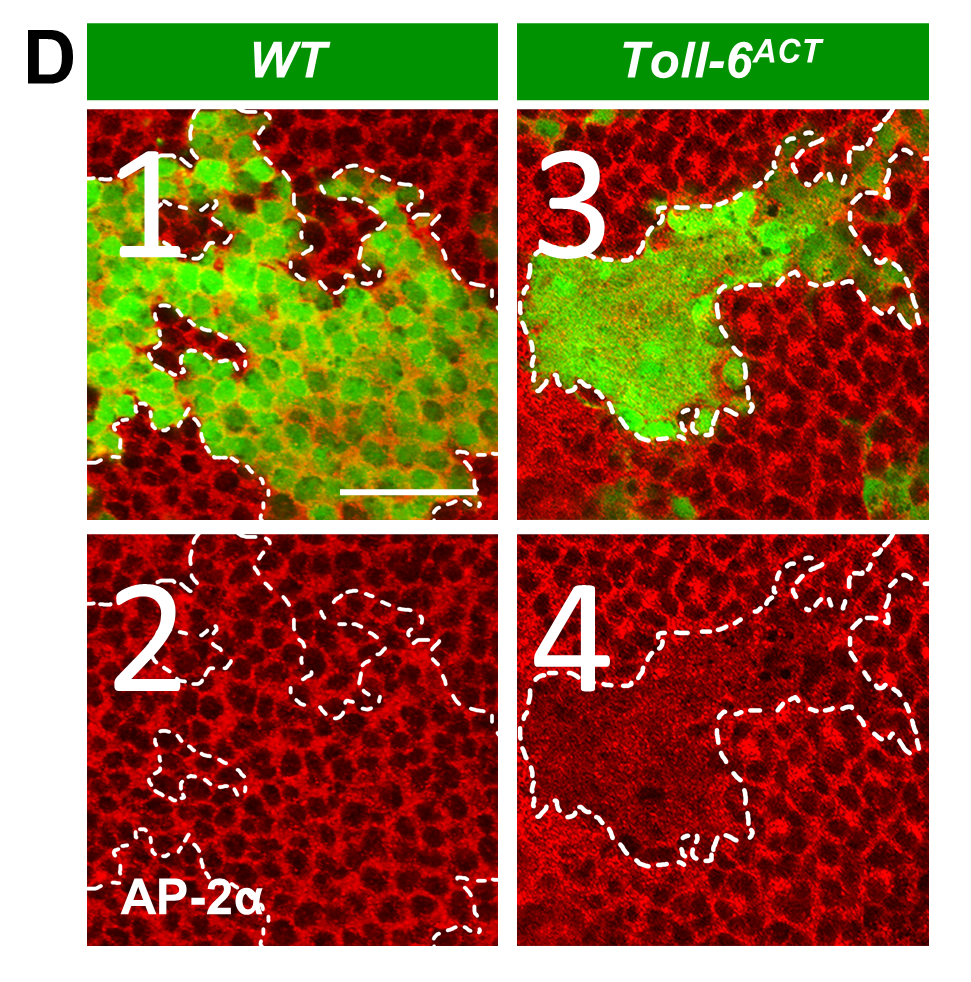

Supplement: Supplementary file 8 — Source data Fig. 4 [file 44318_2025_489_MOESM8_ESM.zip › Figure 4D/0 paper Figure 4D with provided image sequence.tif]

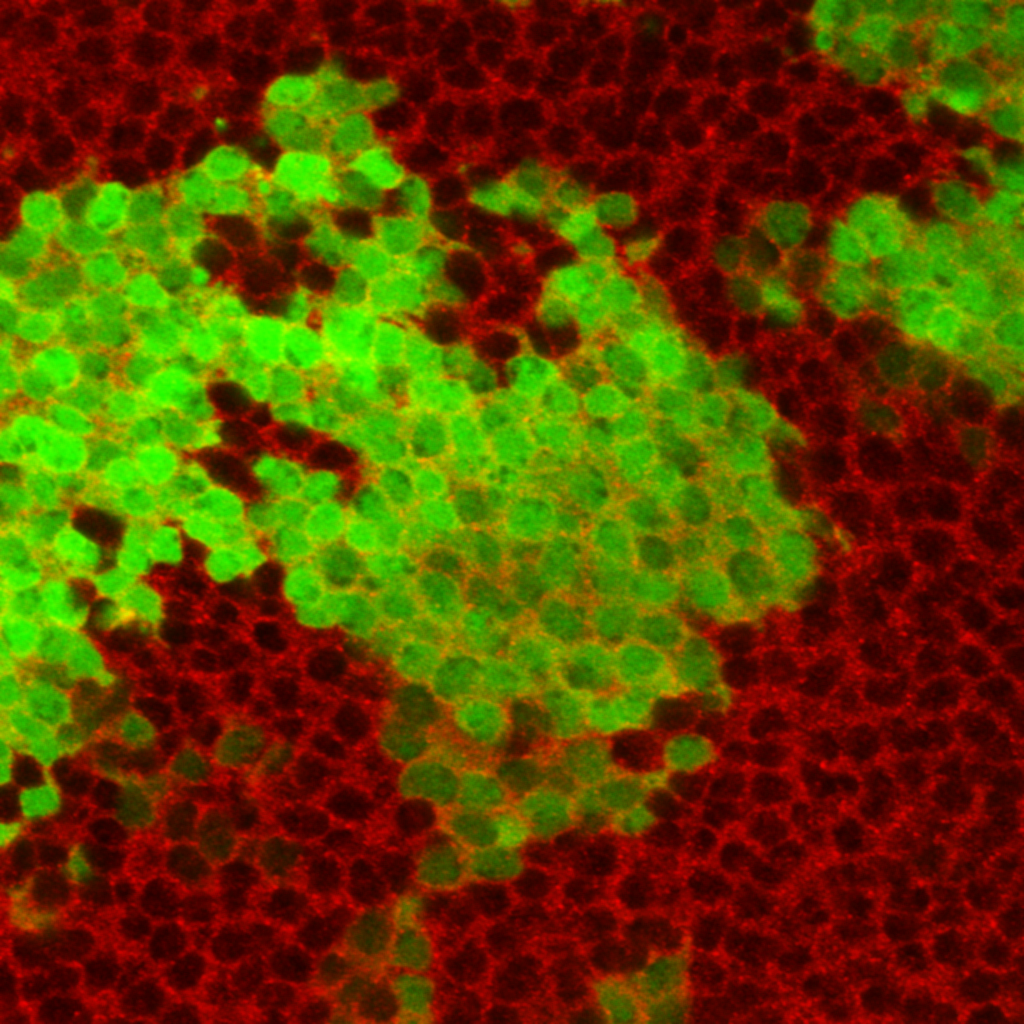

Supplement: Supplementary file 8 — Source data Fig. 4 [file 44318_2025_489_MOESM8_ESM.zip › Figure 4D/1 original image.tif]

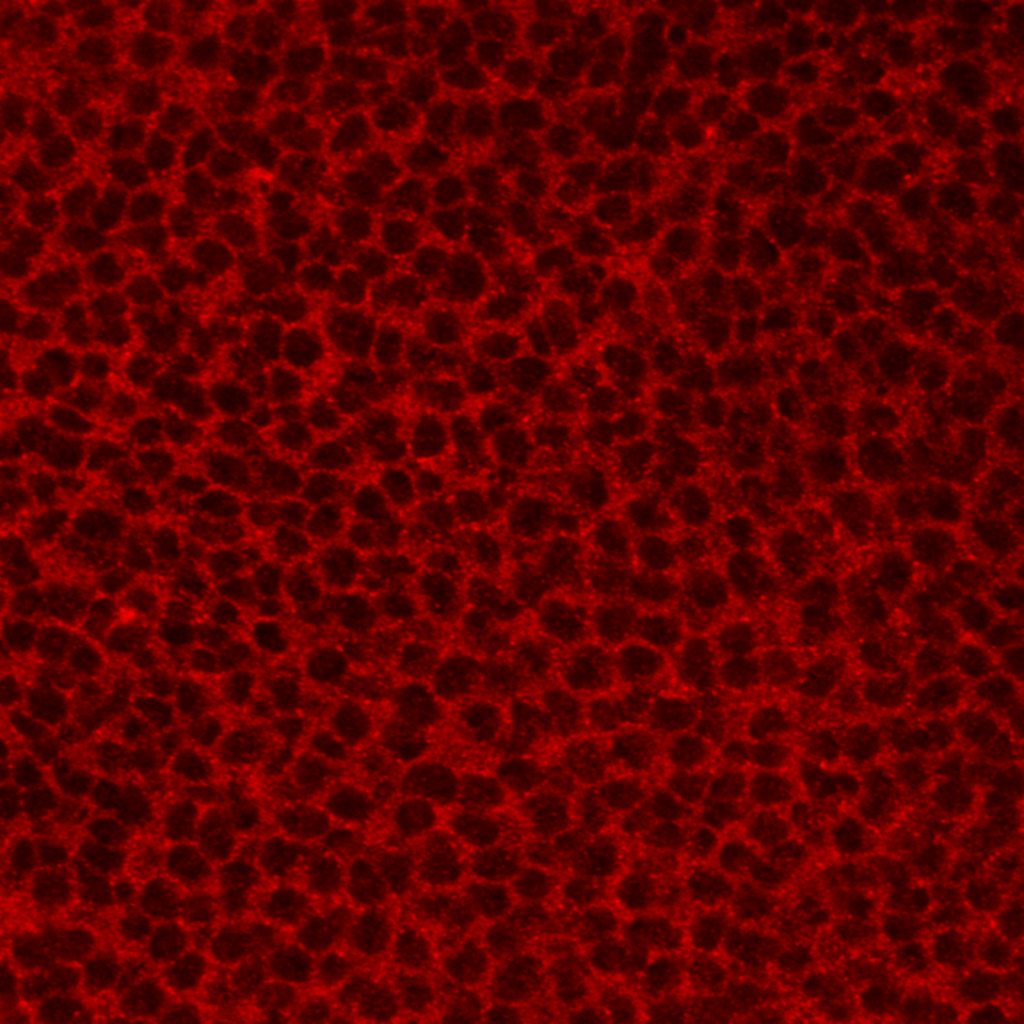

Supplement: Supplementary file 8 — Source data Fig. 4 [file 44318_2025_489_MOESM8_ESM.zip › Figure 4D/2 original image.tif]

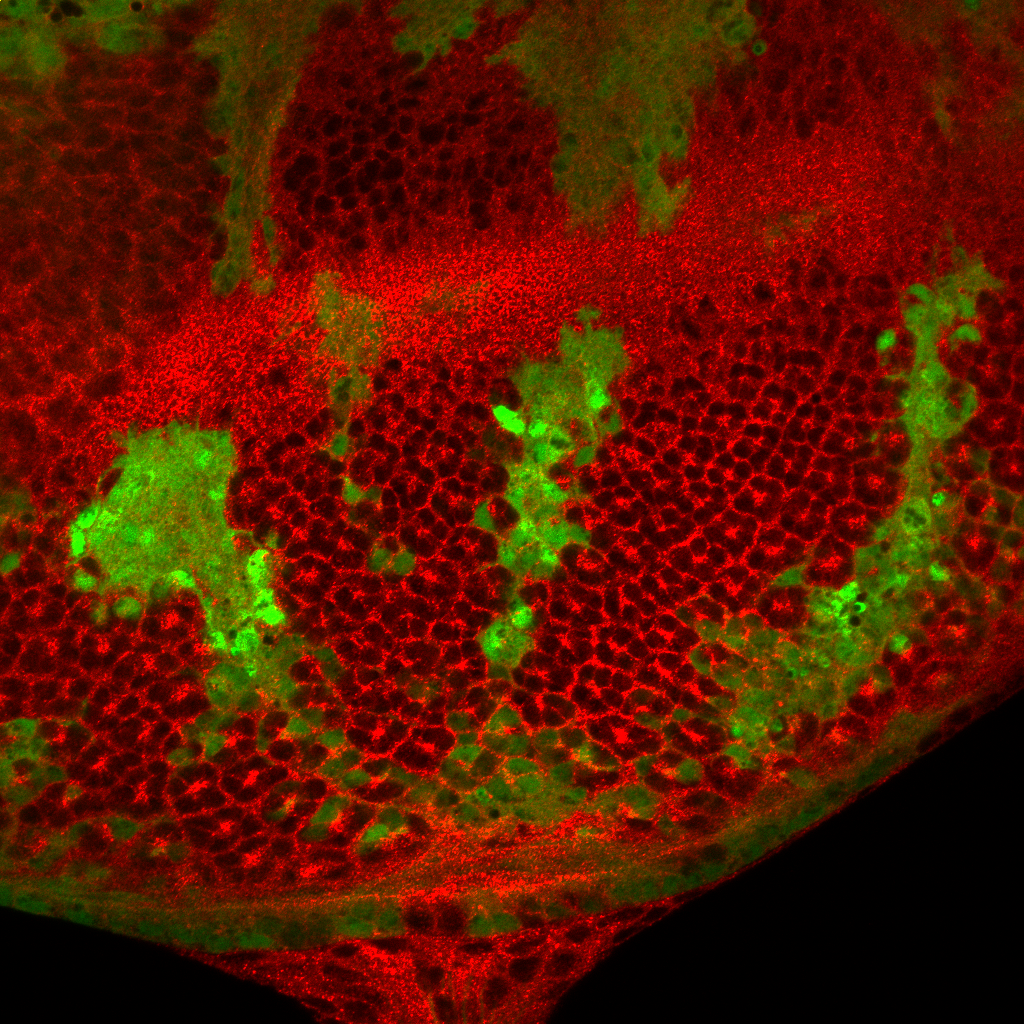

Supplement: Supplementary file 8 — Source data Fig. 4 [file 44318_2025_489_MOESM8_ESM.zip › Figure 4D/3 original image.tif]

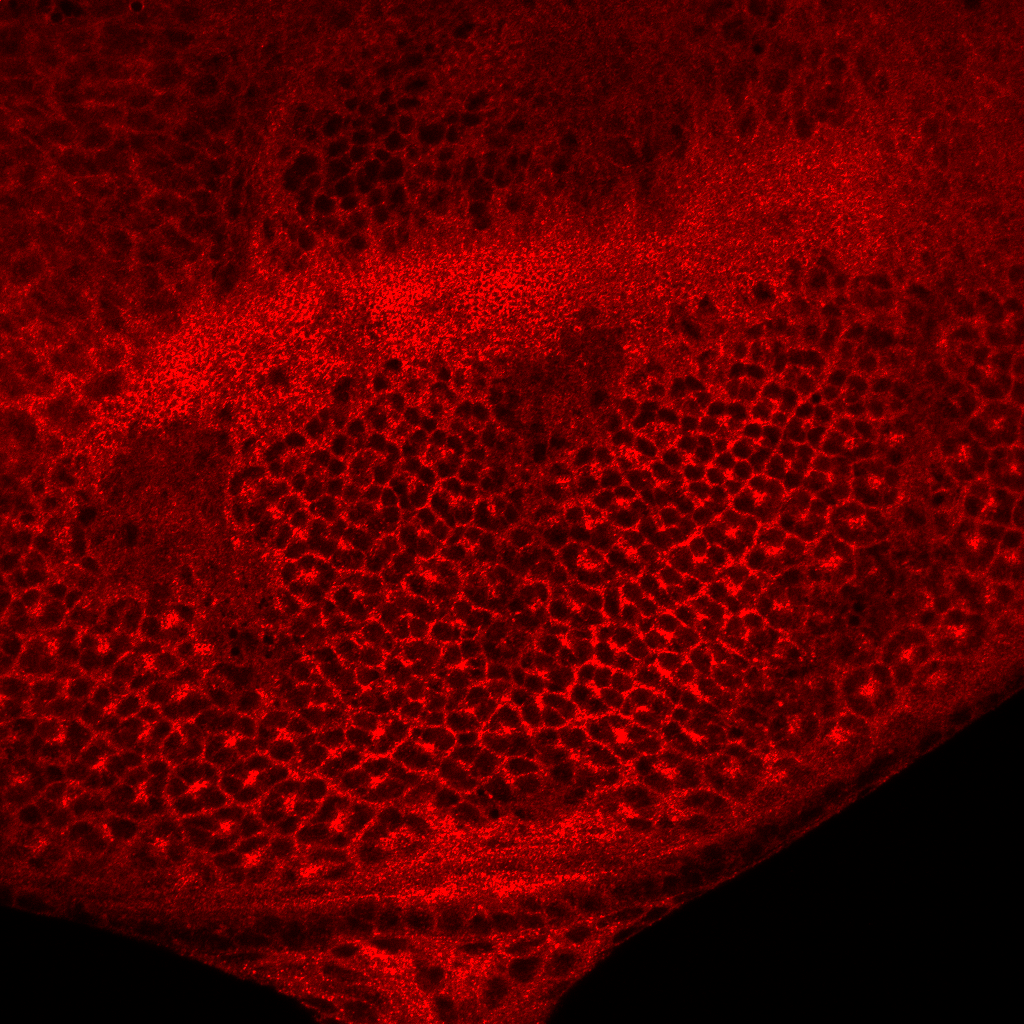

Supplement: Supplementary file 8 — Source data Fig. 4 [file 44318_2025_489_MOESM8_ESM.zip › Figure 4D/4 original image.tif]

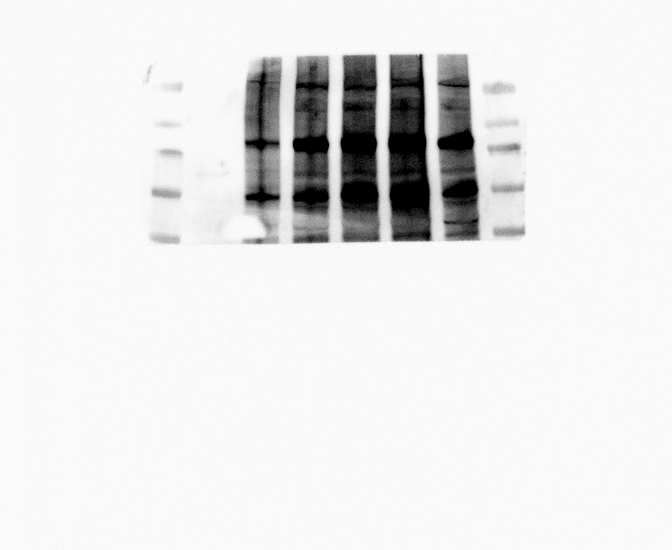

Supplement: Supplementary file 9 — Source data Fig. 5 [file 44318_2025_489_MOESM9_ESM.zip › Figure 5D/1. western myc-Ub.tif]

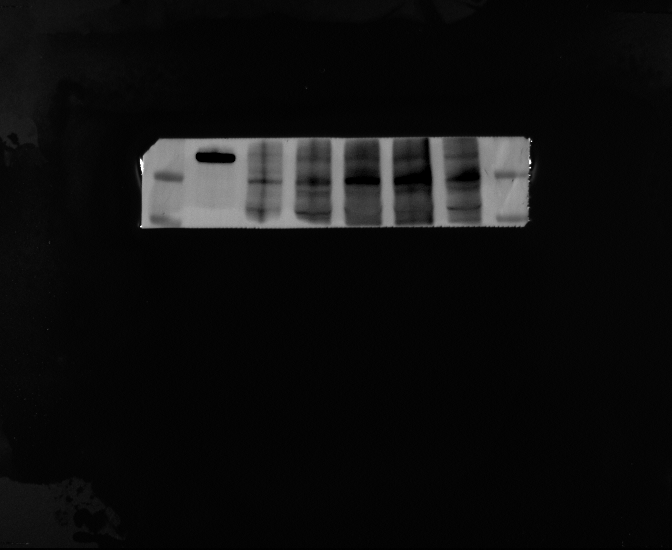

Supplement: Supplementary file 9 — Source data Fig. 5 [file 44318_2025_489_MOESM9_ESM.zip › Figure 5D/2. western Ap-2α .tif]

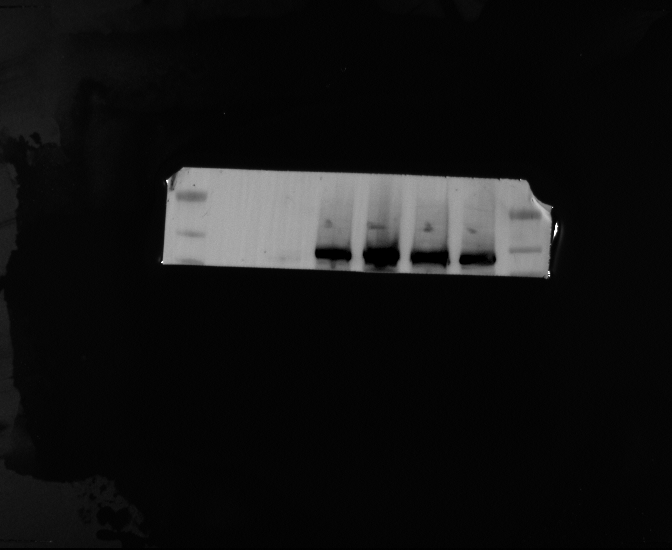

Supplement: Supplementary file 9 — Source data Fig. 5 [file 44318_2025_489_MOESM9_ESM.zip › Figure 5D/3. western mib1.tif]

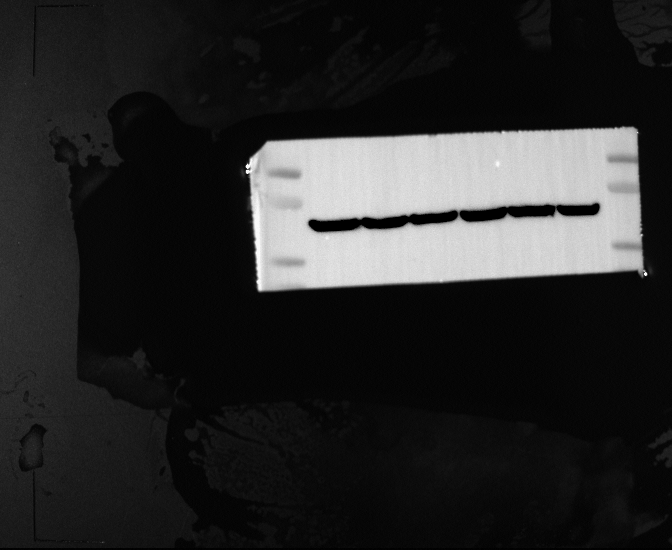

Supplement: Supplementary file 9 — Source data Fig. 5 [file 44318_2025_489_MOESM9_ESM.zip › Figure 5D/4. western actin.tif]

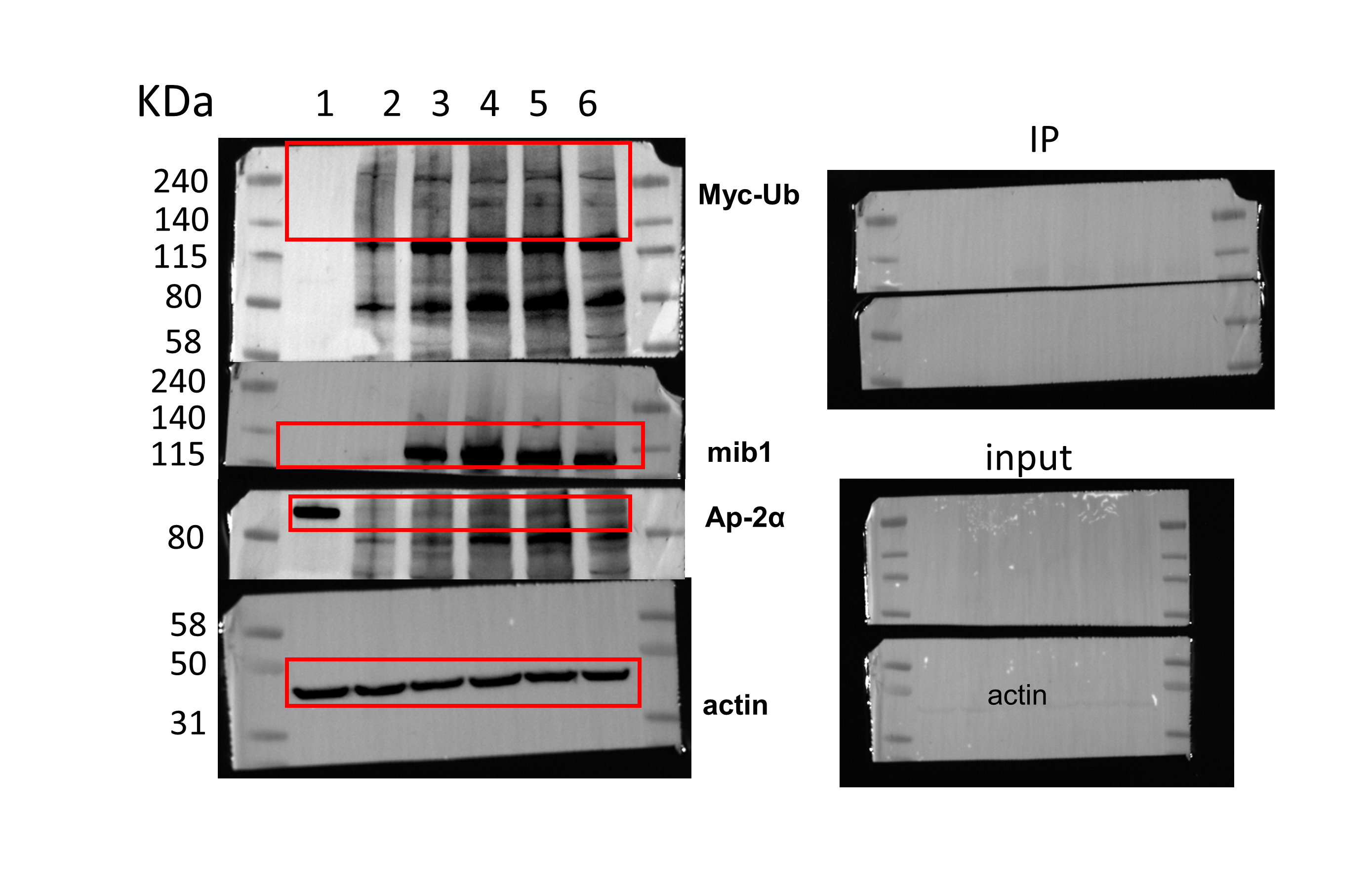

Supplement: Supplementary file 9 — Source data Fig. 5 [file 44318_2025_489_MOESM9_ESM.zip › Figure 5D/5. western whole.TIF]

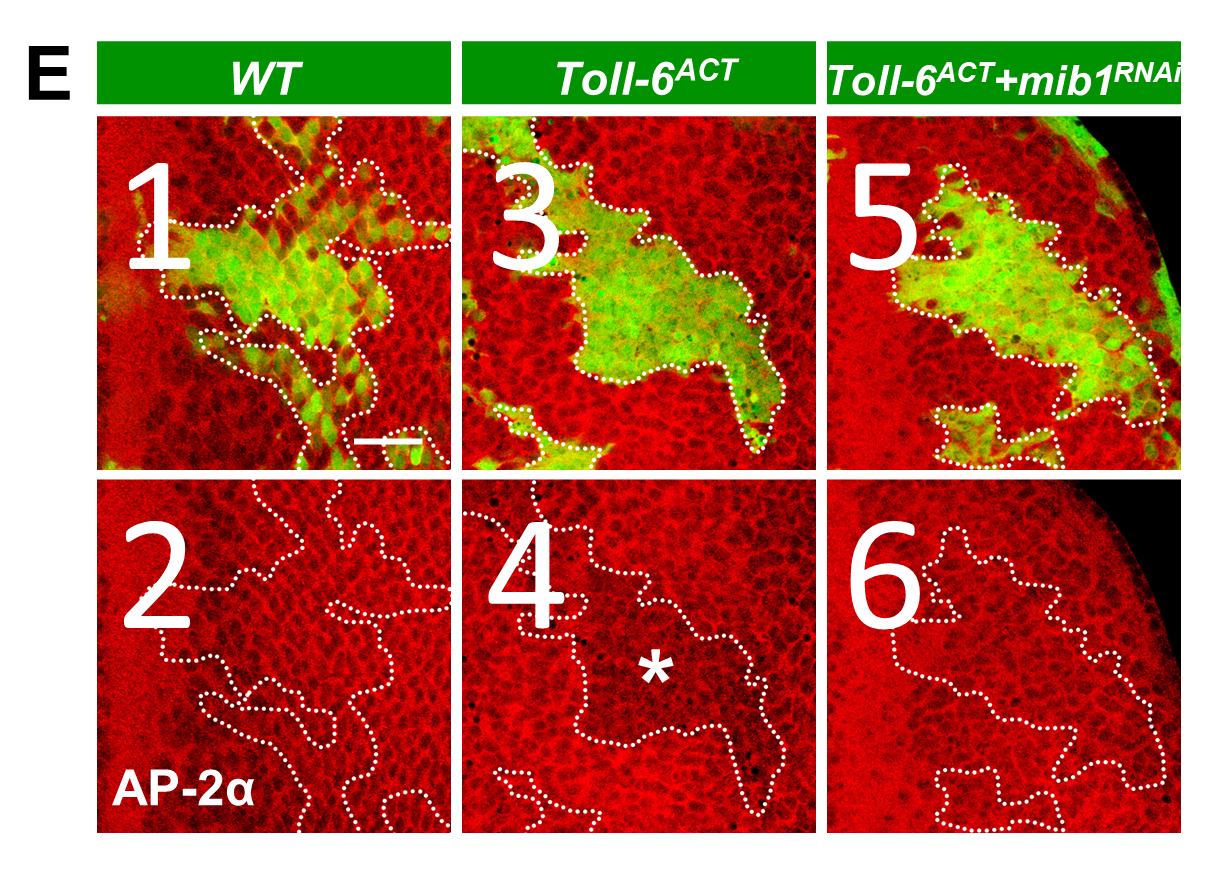

Supplement: Supplementary file 9 — Source data Fig. 5 [file 44318_2025_489_MOESM9_ESM.zip › Figure 5E/0 paper Figure 5E with provided image sequence.tif]

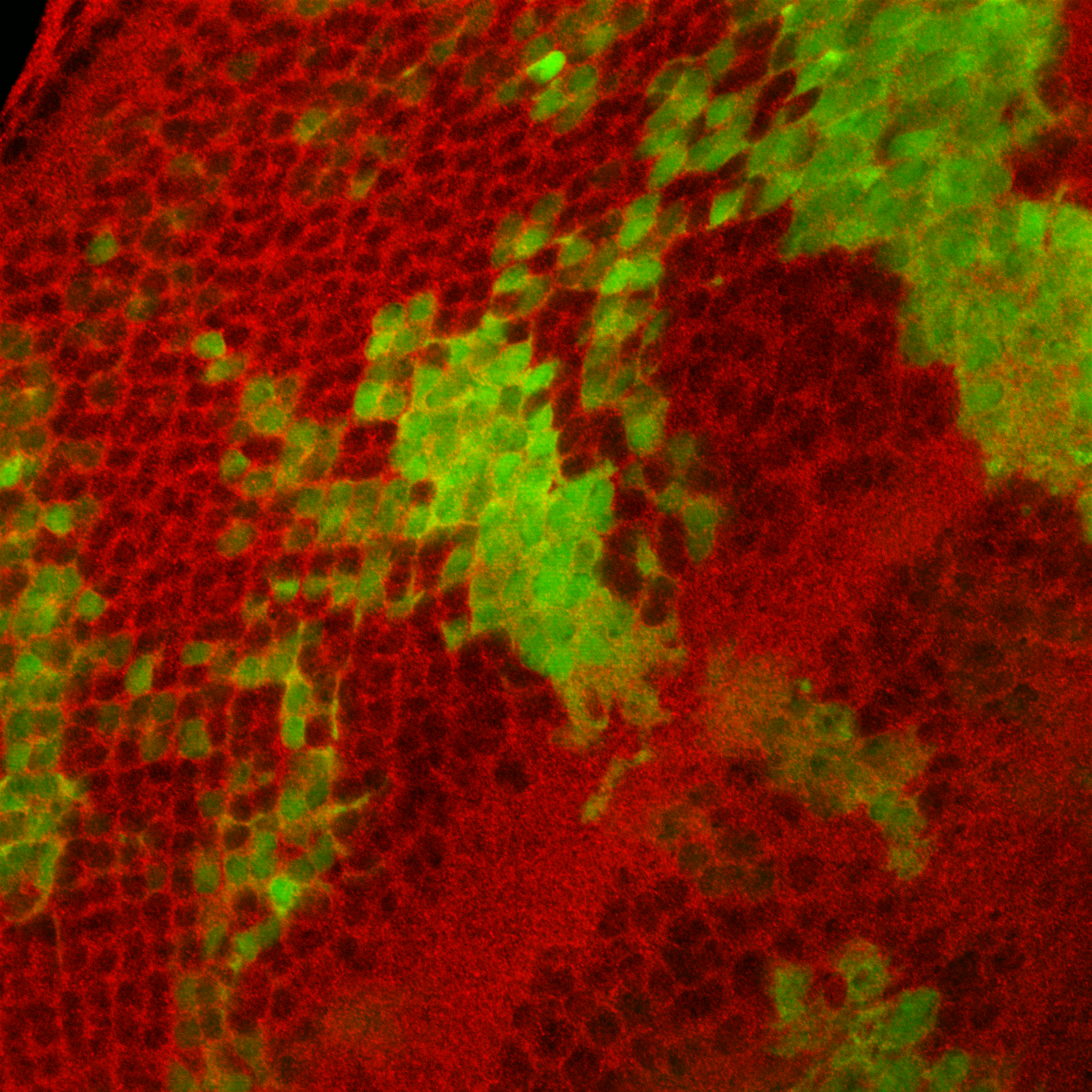

Supplement: Supplementary file 9 — Source data Fig. 5 [file 44318_2025_489_MOESM9_ESM.zip › Figure 5E/1 original image.tif]

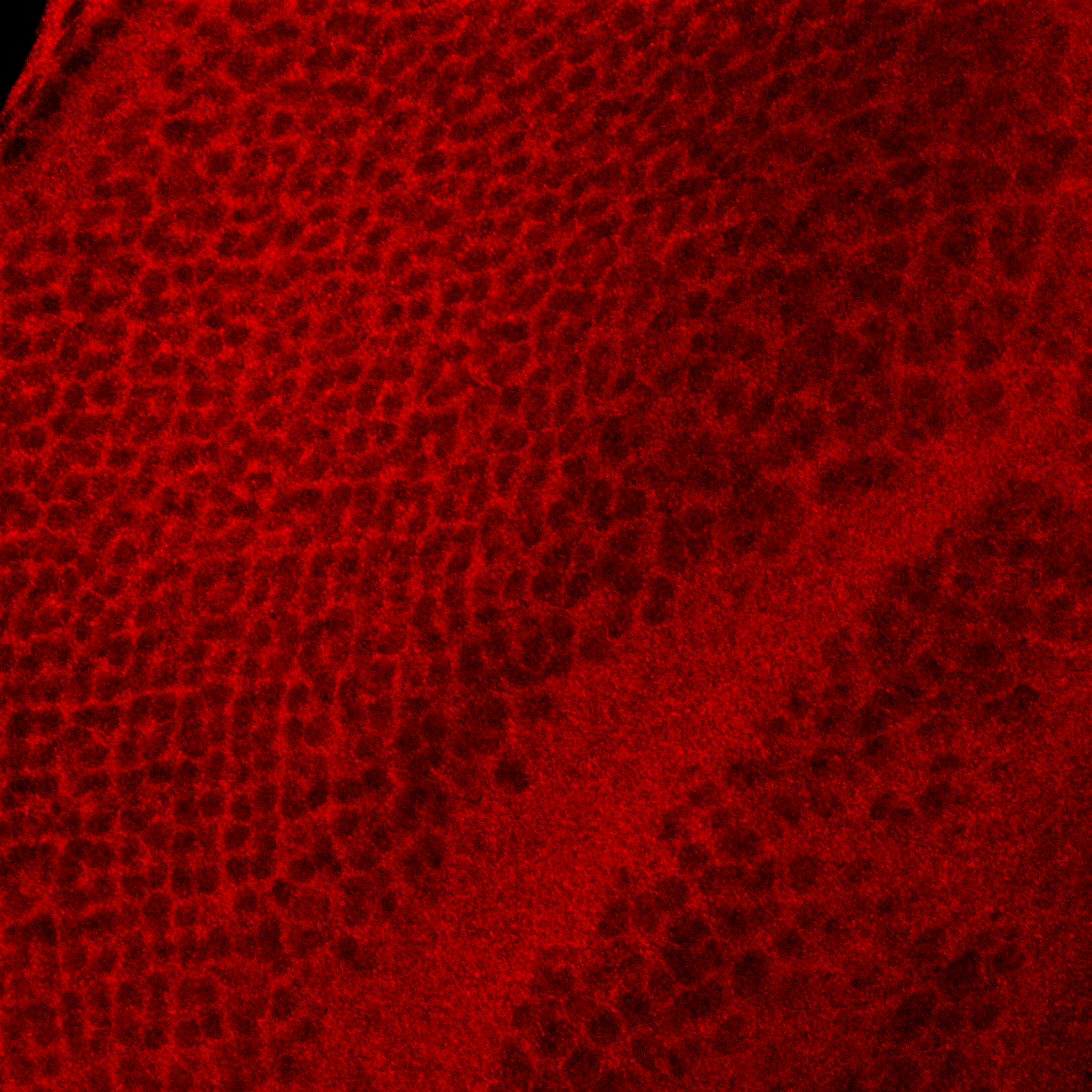

Supplement: Supplementary file 9 — Source data Fig. 5 [file 44318_2025_489_MOESM9_ESM.zip › Figure 5E/2 original image.tif]

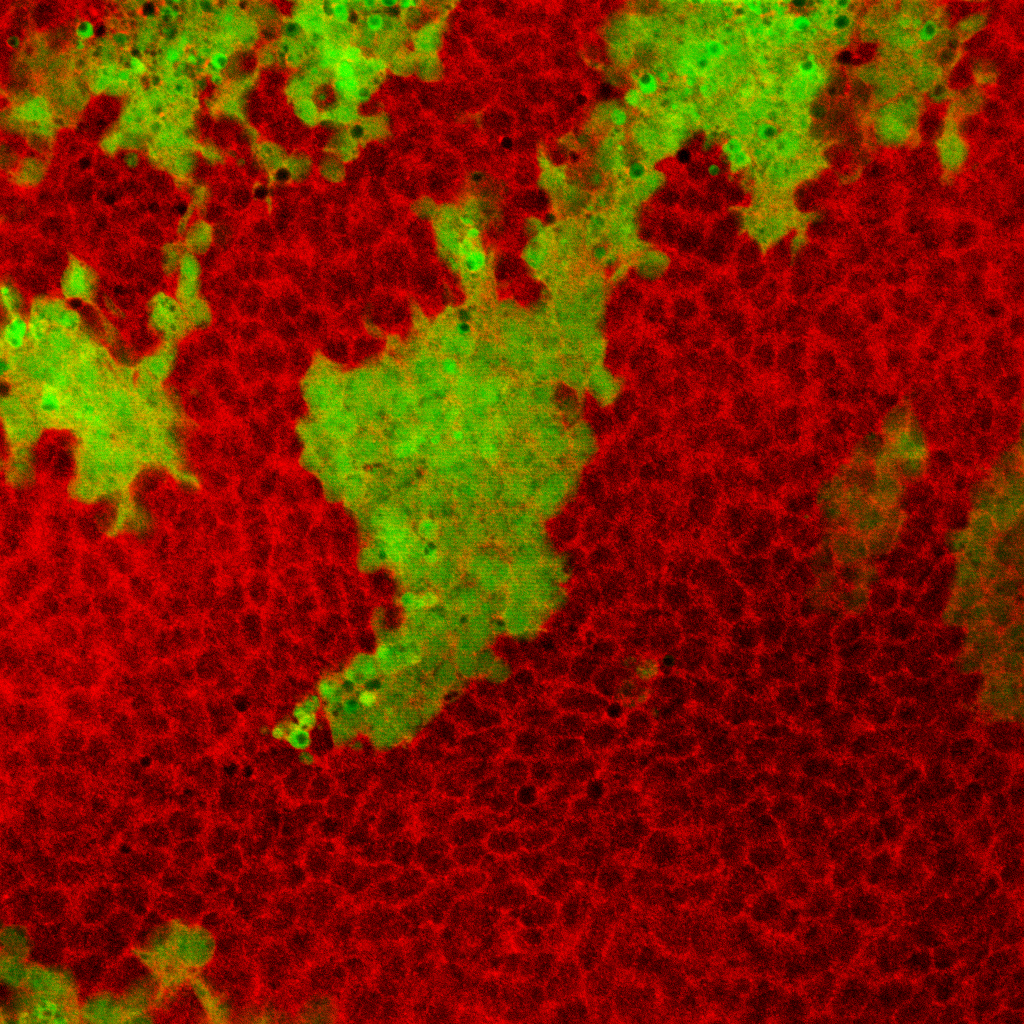

Supplement: Supplementary file 9 — Source data Fig. 5 [file 44318_2025_489_MOESM9_ESM.zip › Figure 5E/3 original image.tif]

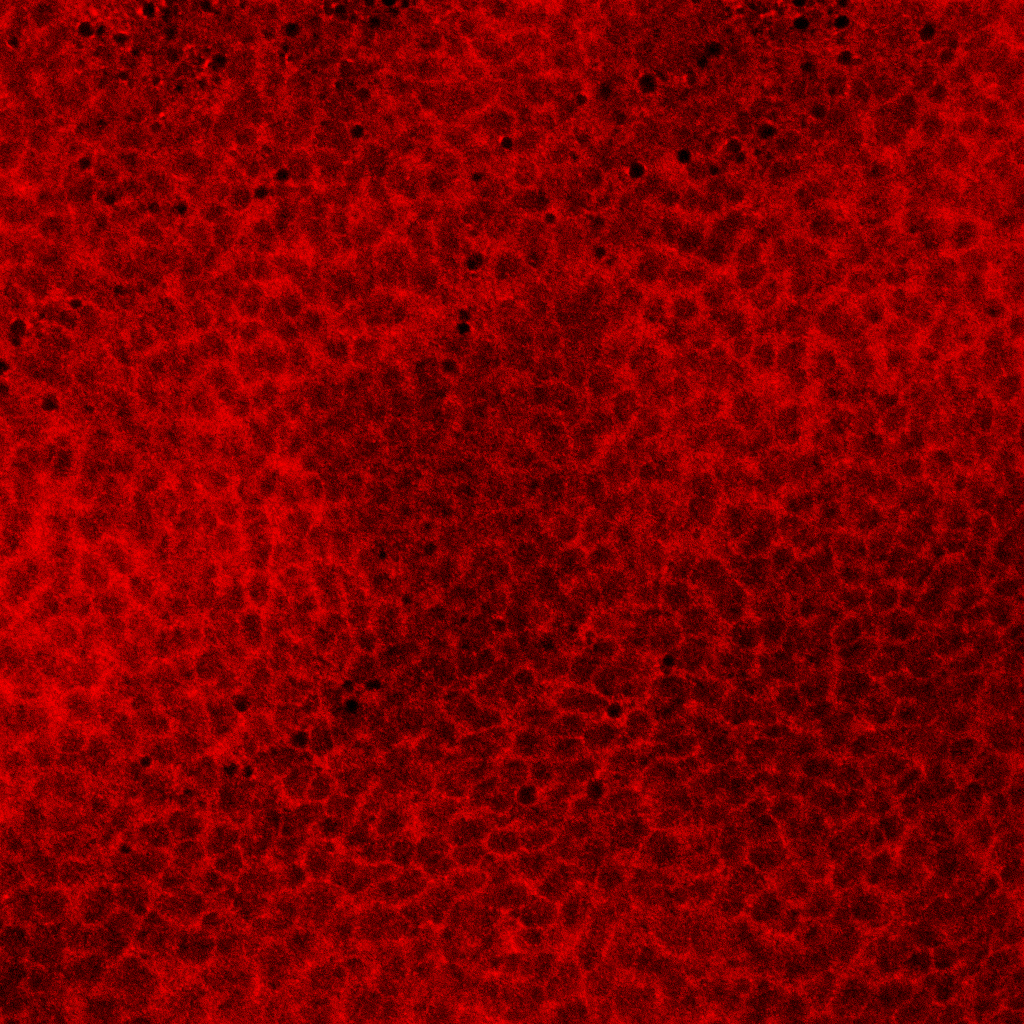

Supplement: Supplementary file 9 — Source data Fig. 5 [file 44318_2025_489_MOESM9_ESM.zip › Figure 5E/4 original image.tif]

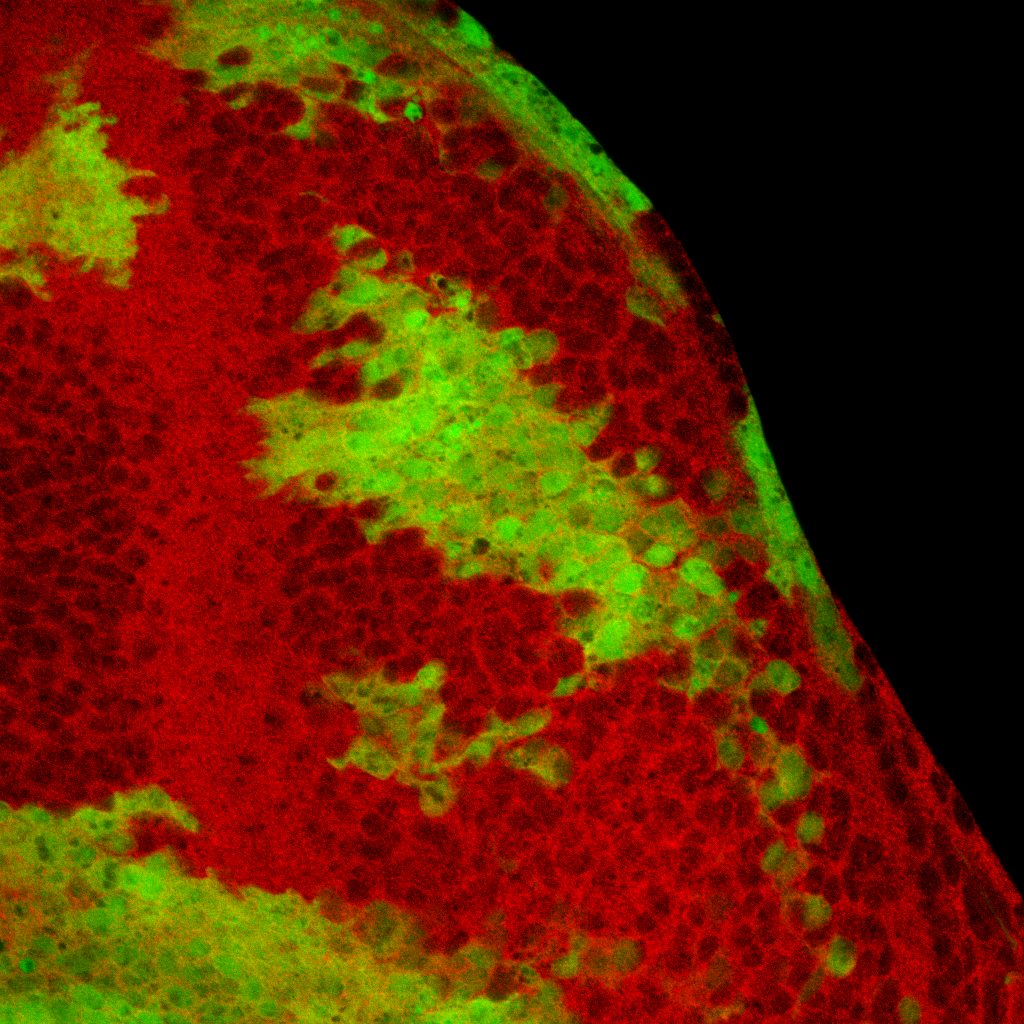

Supplement: Supplementary file 9 — Source data Fig. 5 [file 44318_2025_489_MOESM9_ESM.zip › Figure 5E/5 original image.tif]

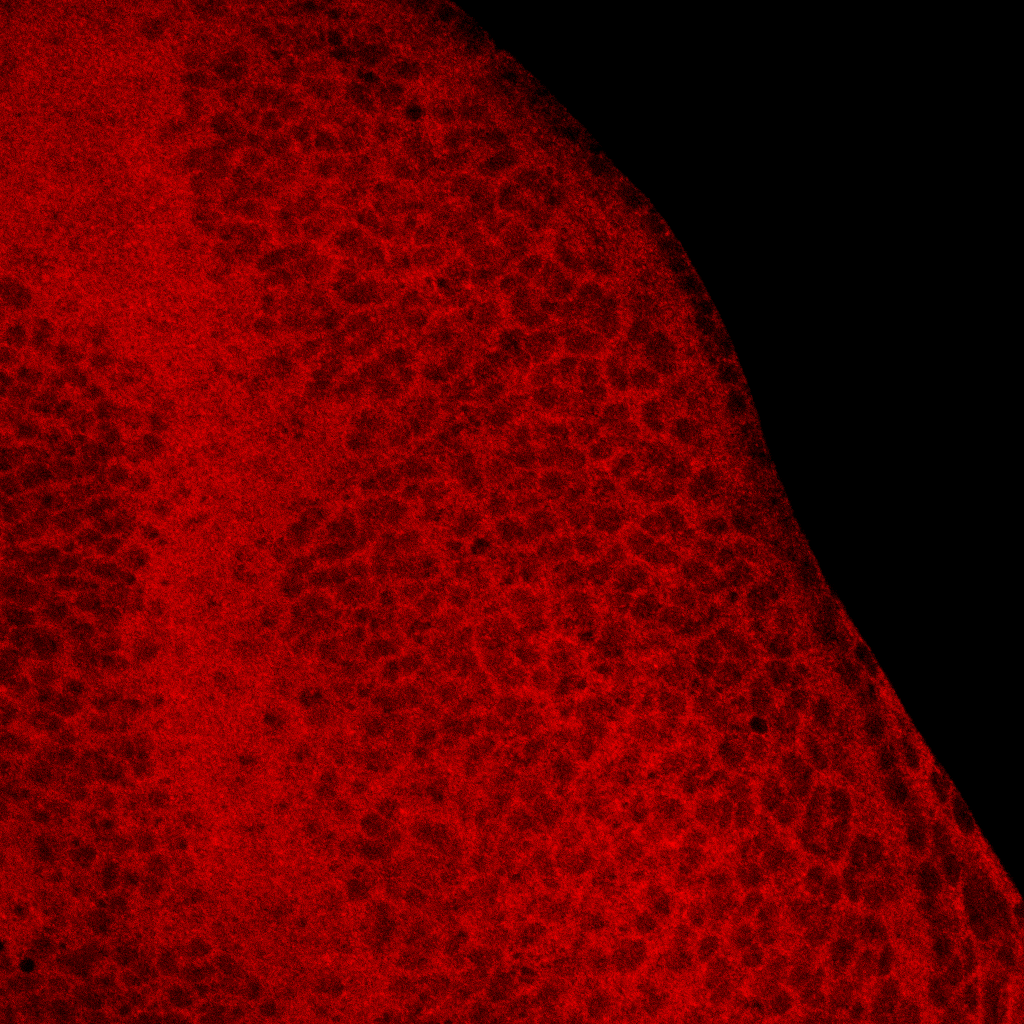

Supplement: Supplementary file 9 — Source data Fig. 5 [file 44318_2025_489_MOESM9_ESM.zip › Figure 5E/6 original image.tif]

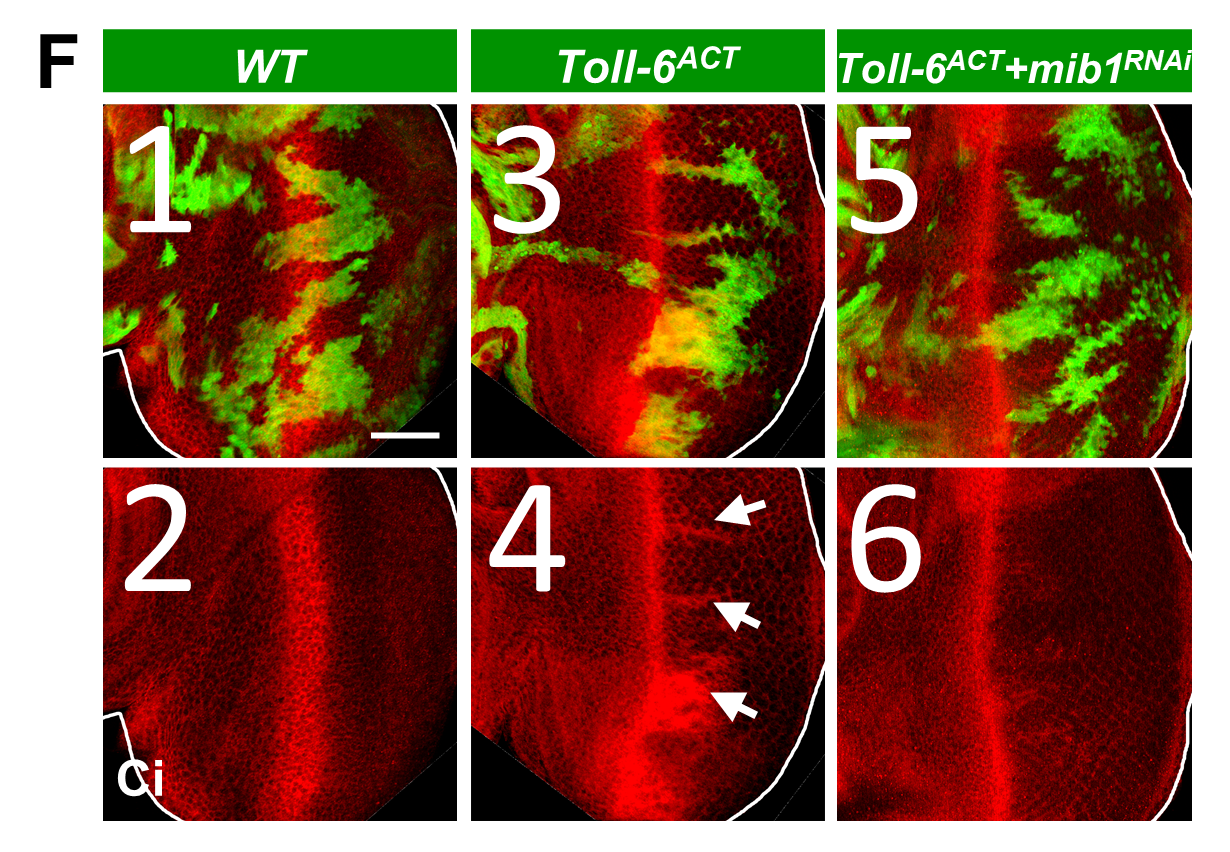

Supplement: Supplementary file 9 — Source data Fig. 5 [file 44318_2025_489_MOESM9_ESM.zip › Figure 5F/0 paper Figure 5F with provided image sequence.tif]

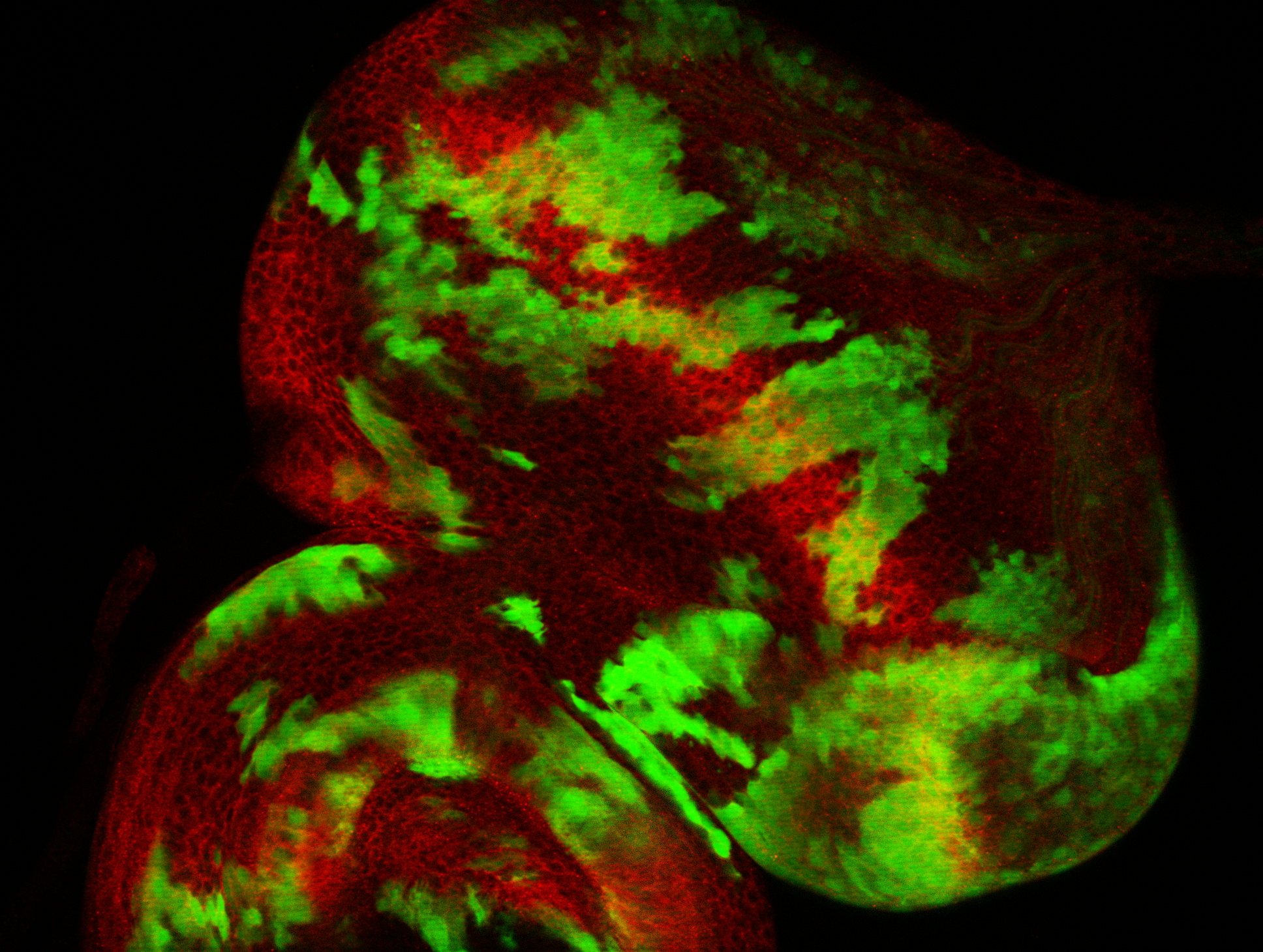

Supplement: Supplementary file 9 — Source data Fig. 5 [file 44318_2025_489_MOESM9_ESM.zip › Figure 5F/1 original image.tif]

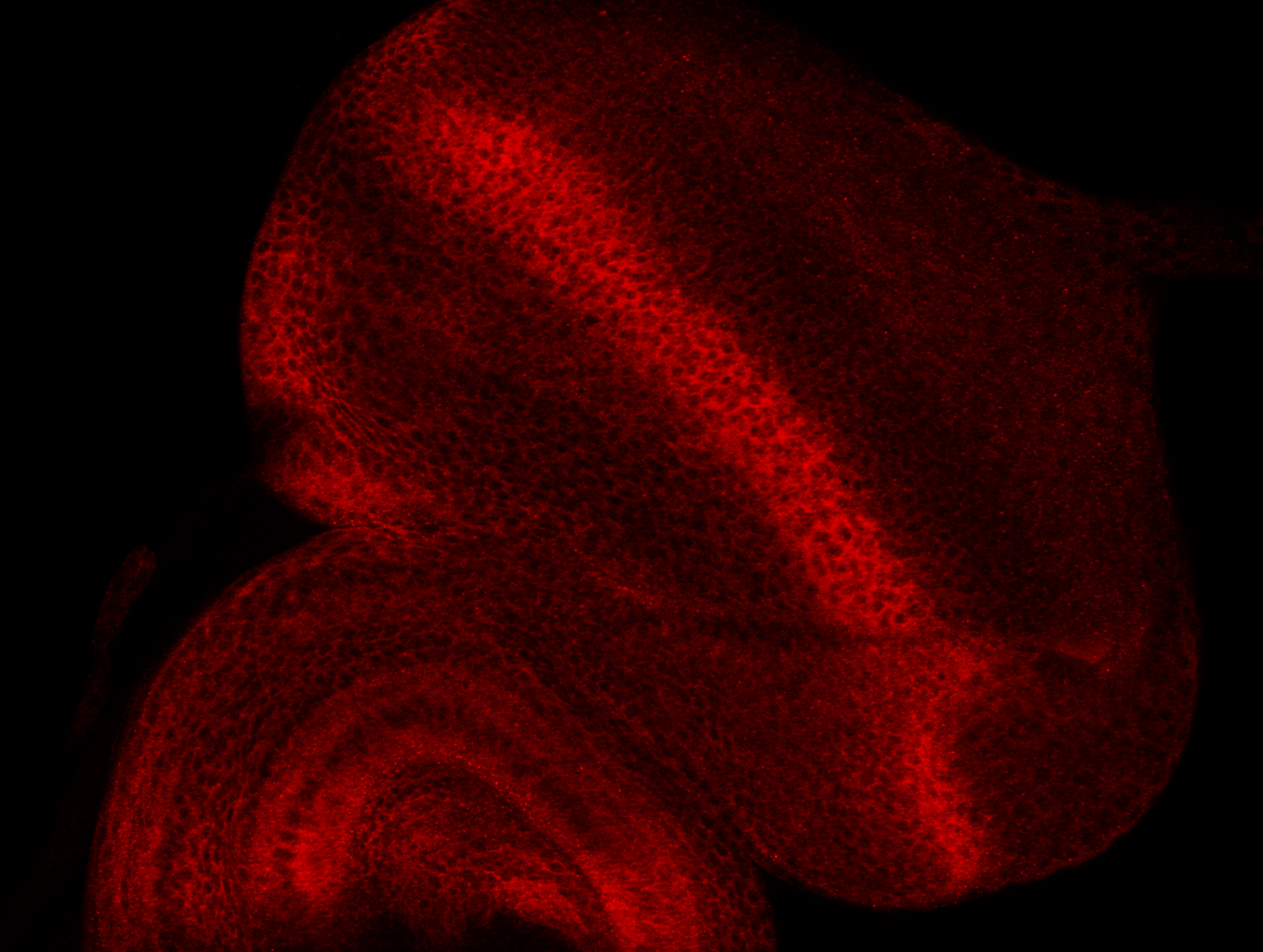

Supplement: Supplementary file 9 — Source data Fig. 5 [file 44318_2025_489_MOESM9_ESM.zip › Figure 5F/2 original image.tif]

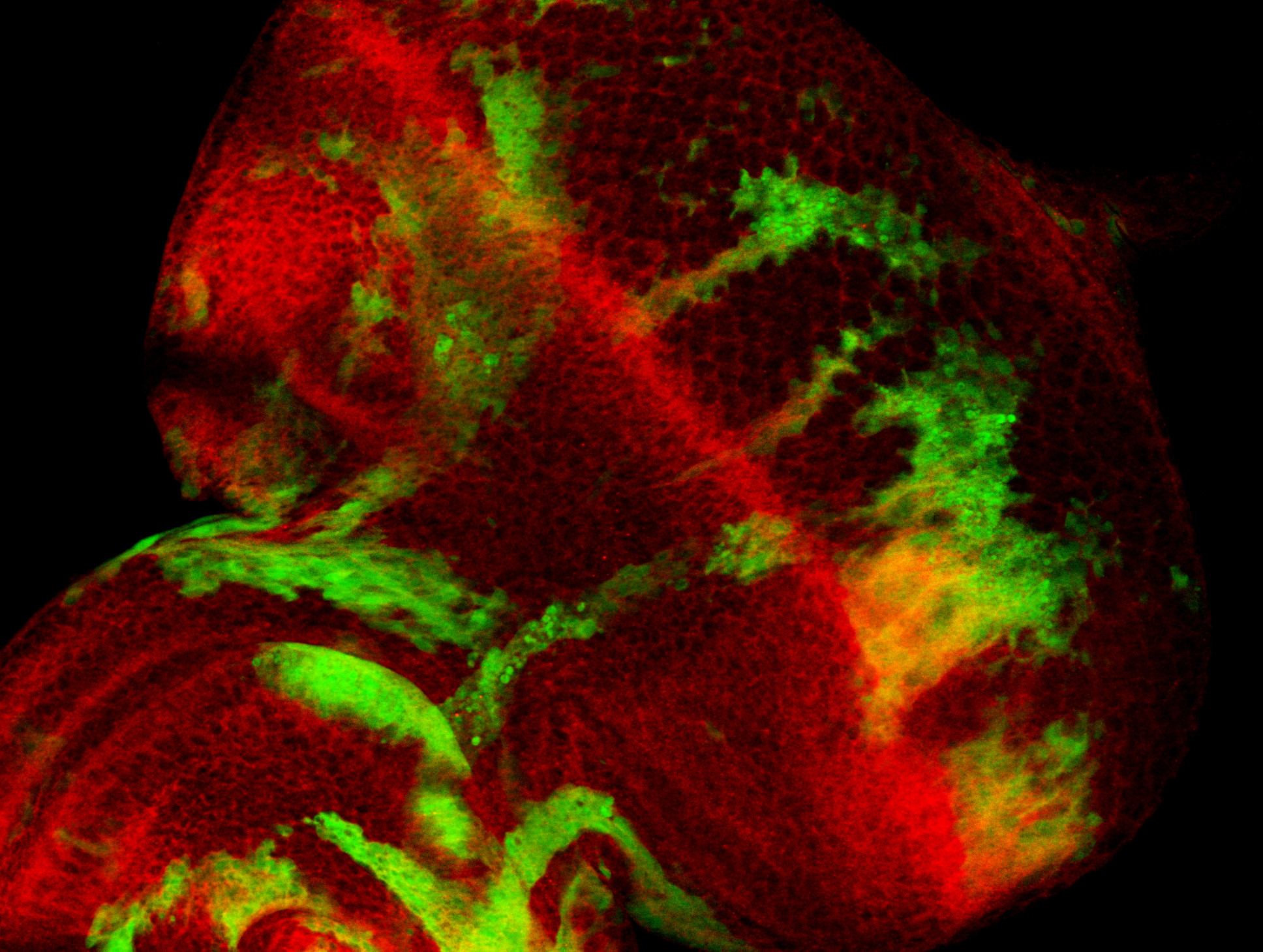

Supplement: Supplementary file 9 — Source data Fig. 5 [file 44318_2025_489_MOESM9_ESM.zip › Figure 5F/3 original image.tif]

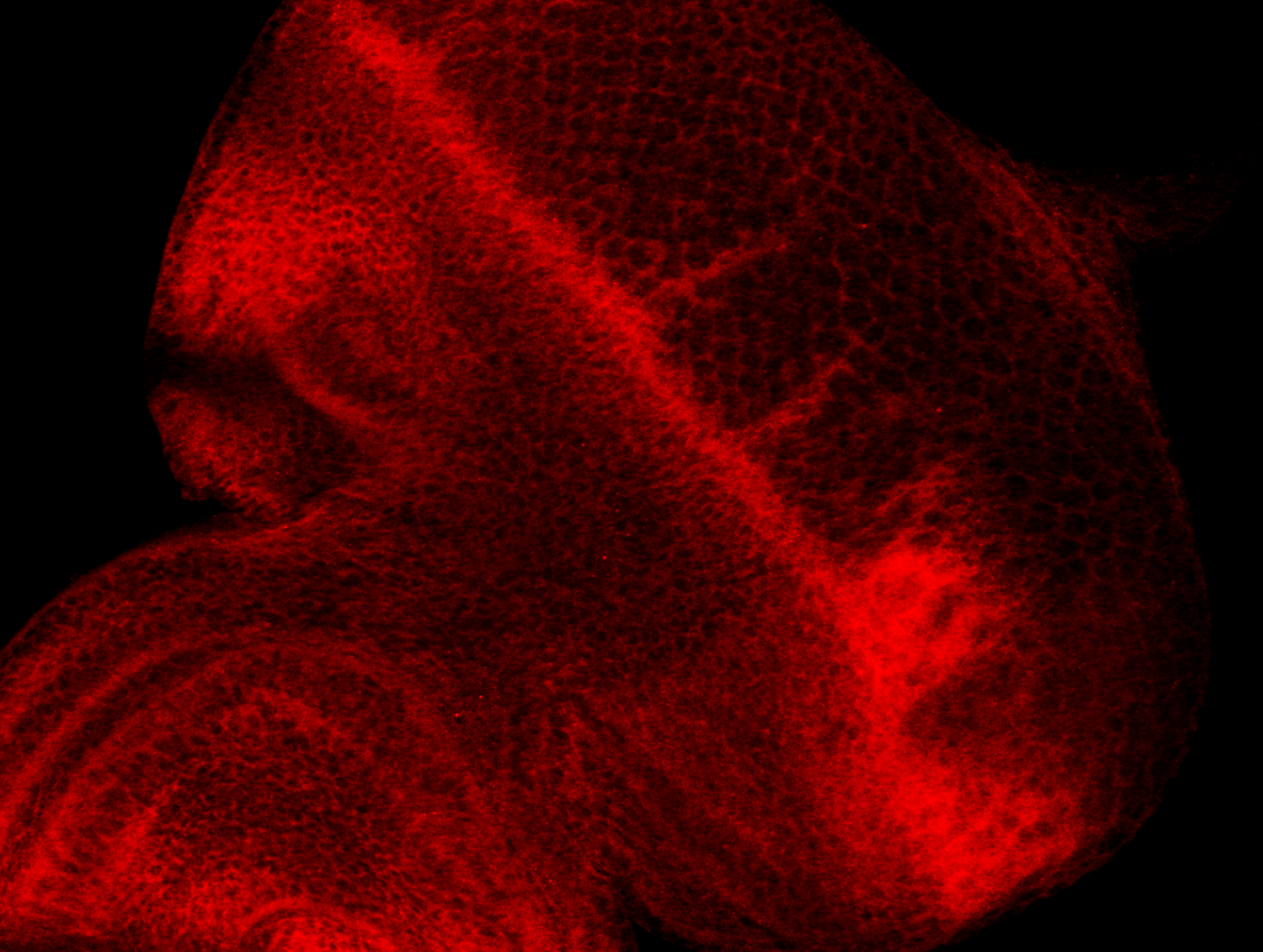

Supplement: Supplementary file 9 — Source data Fig. 5 [file 44318_2025_489_MOESM9_ESM.zip › Figure 5F/4 original image.tif]

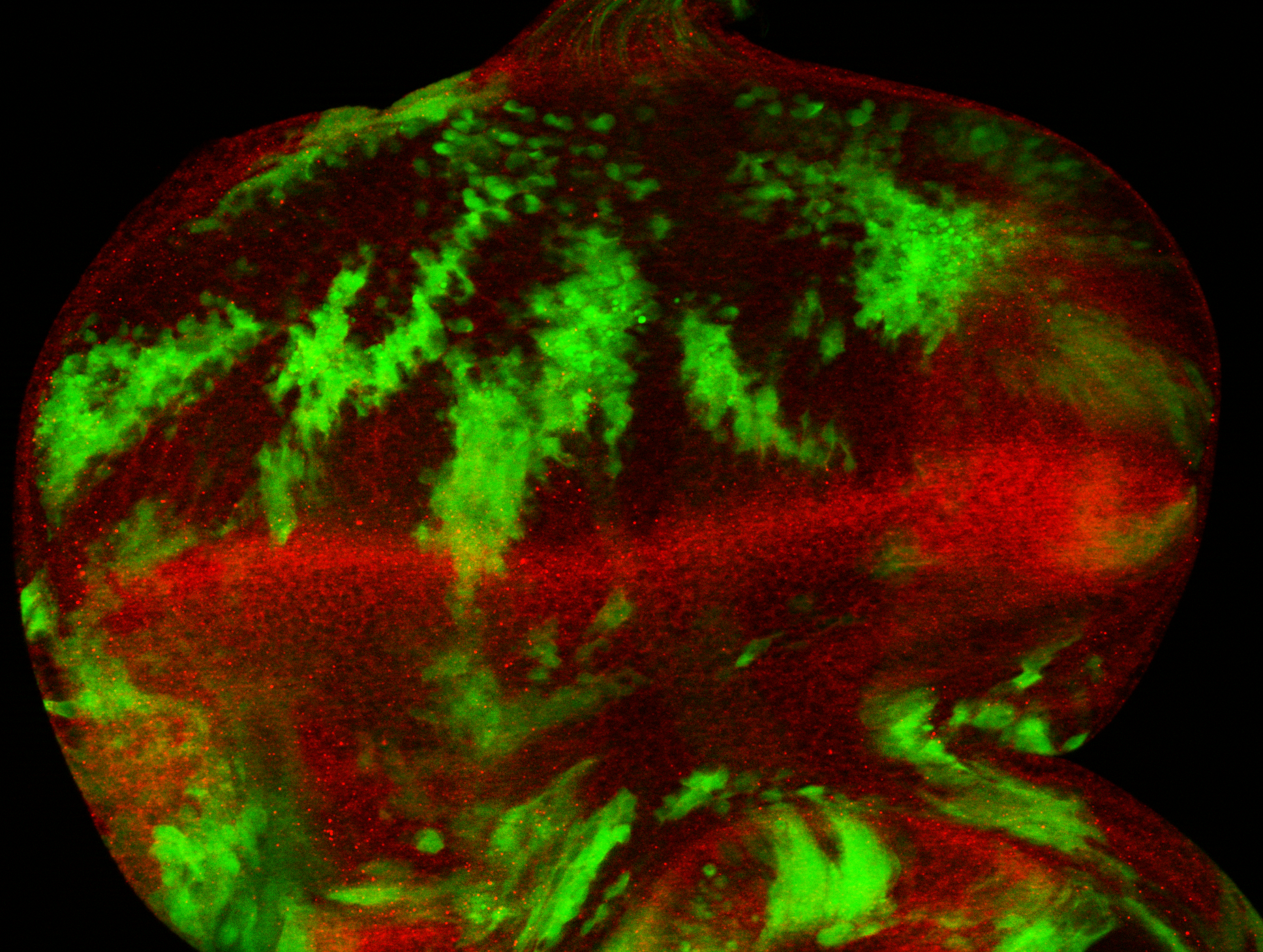

Supplement: Supplementary file 9 — Source data Fig. 5 [file 44318_2025_489_MOESM9_ESM.zip › Figure 5F/5 original image.tif]

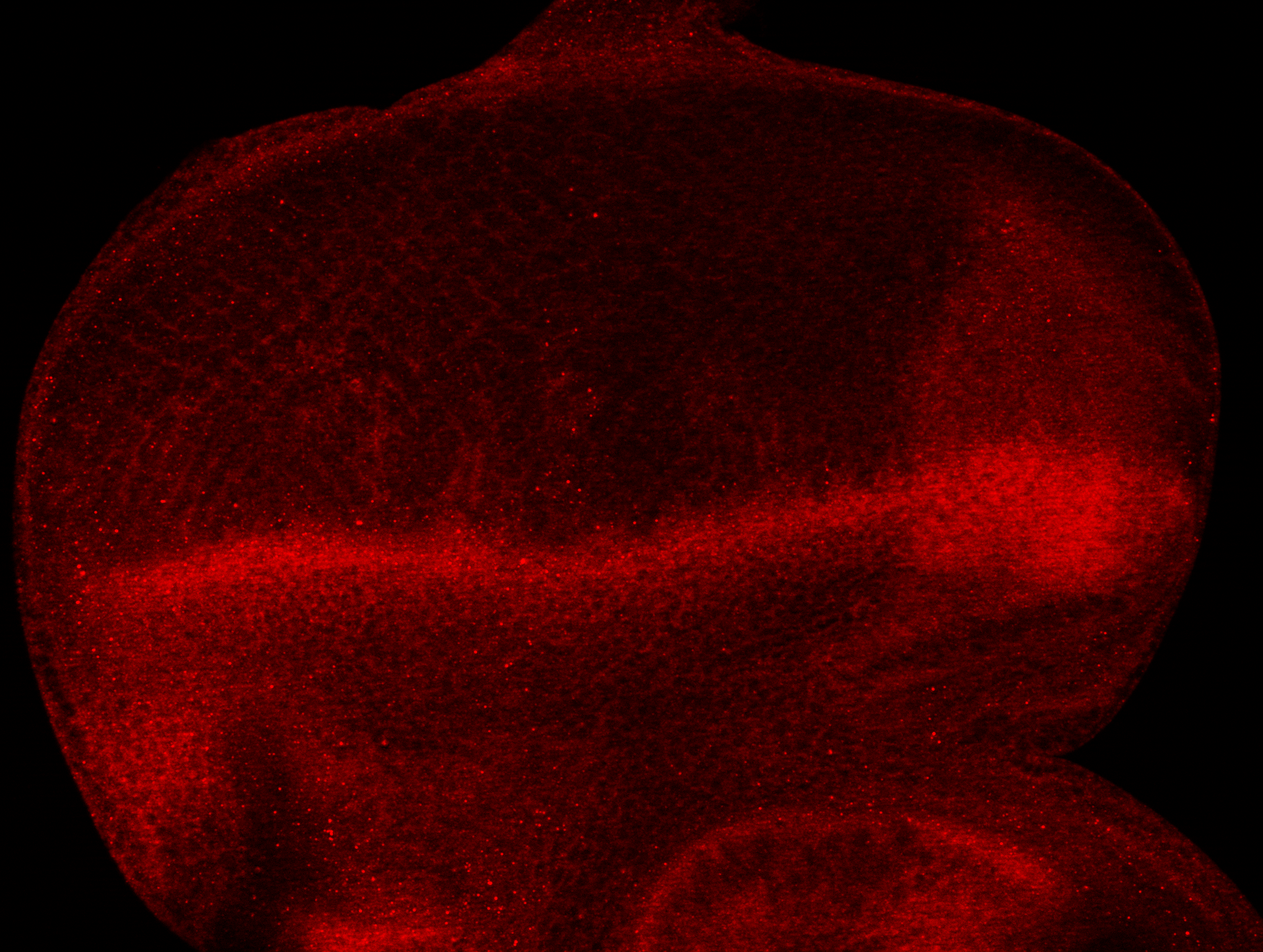

Supplement: Supplementary file 9 — Source data Fig. 5 [file 44318_2025_489_MOESM9_ESM.zip › Figure 5F/6 original image.tif]

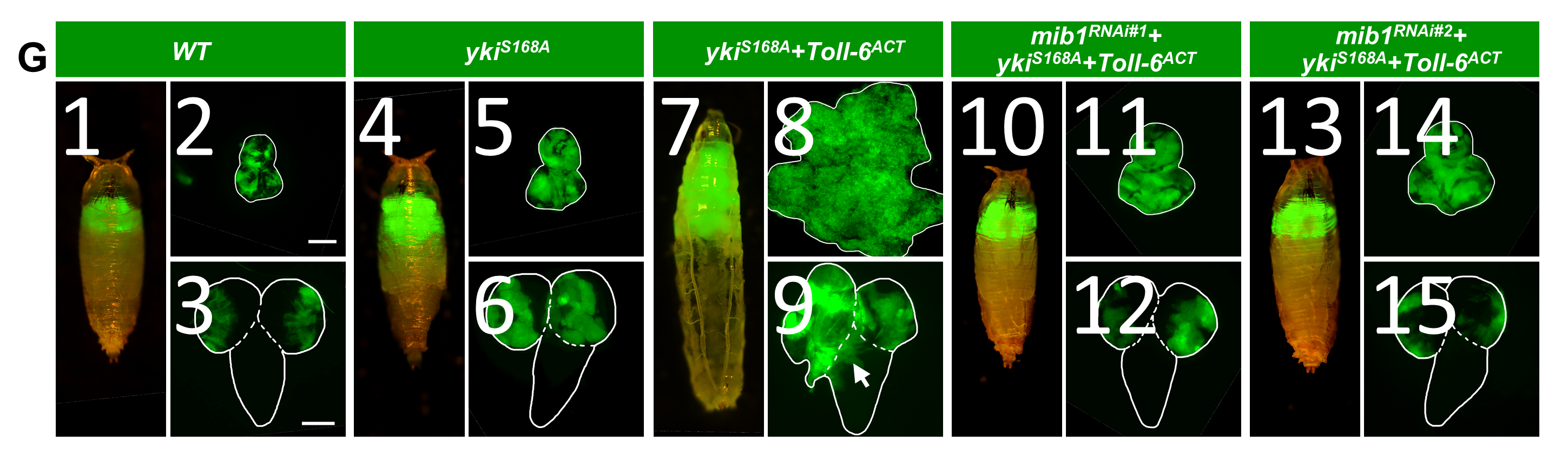

Supplement: Supplementary file 9 — Source data Fig. 5 [file 44318_2025_489_MOESM9_ESM.zip › Figure 5G/0 paper Figure 5G with provided image sequence.tif]

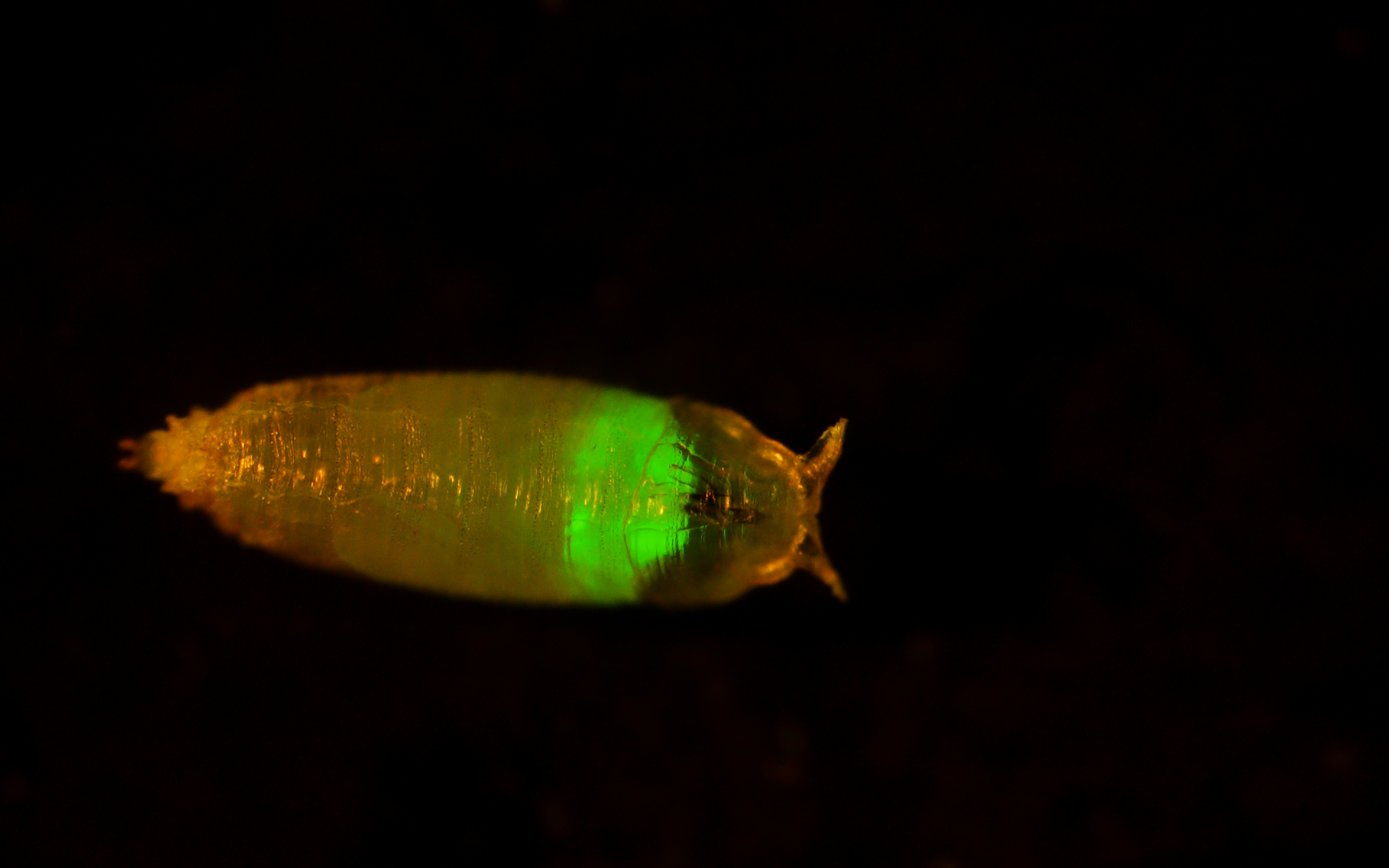

Supplement: Supplementary file 9 — Source data Fig. 5 [file 44318_2025_489_MOESM9_ESM.zip › Figure 5G/1 original image.tif]

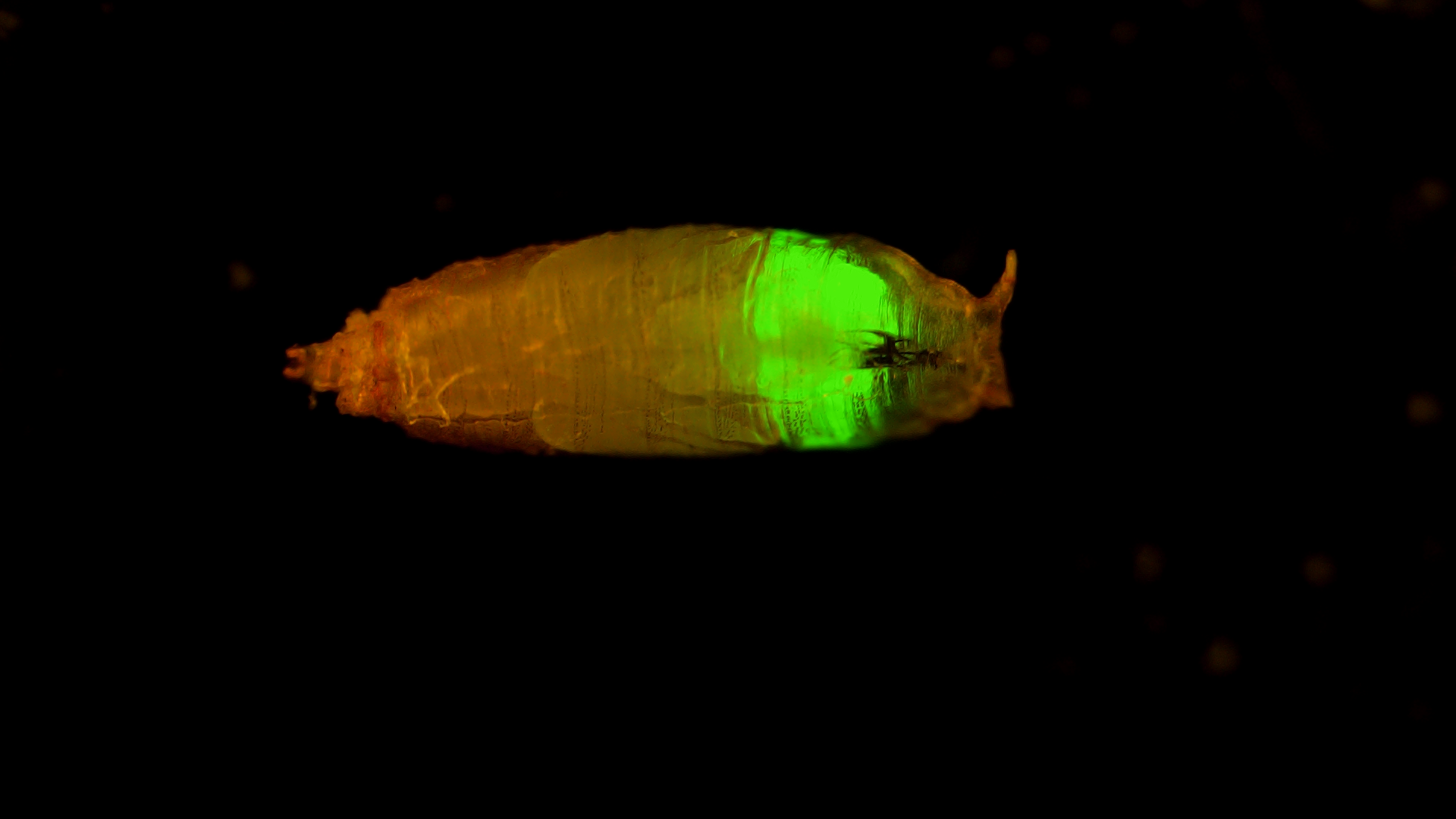

Supplement: Supplementary file 9 — Source data Fig. 5 [file 44318_2025_489_MOESM9_ESM.zip › Figure 5G/10 original image.tif]

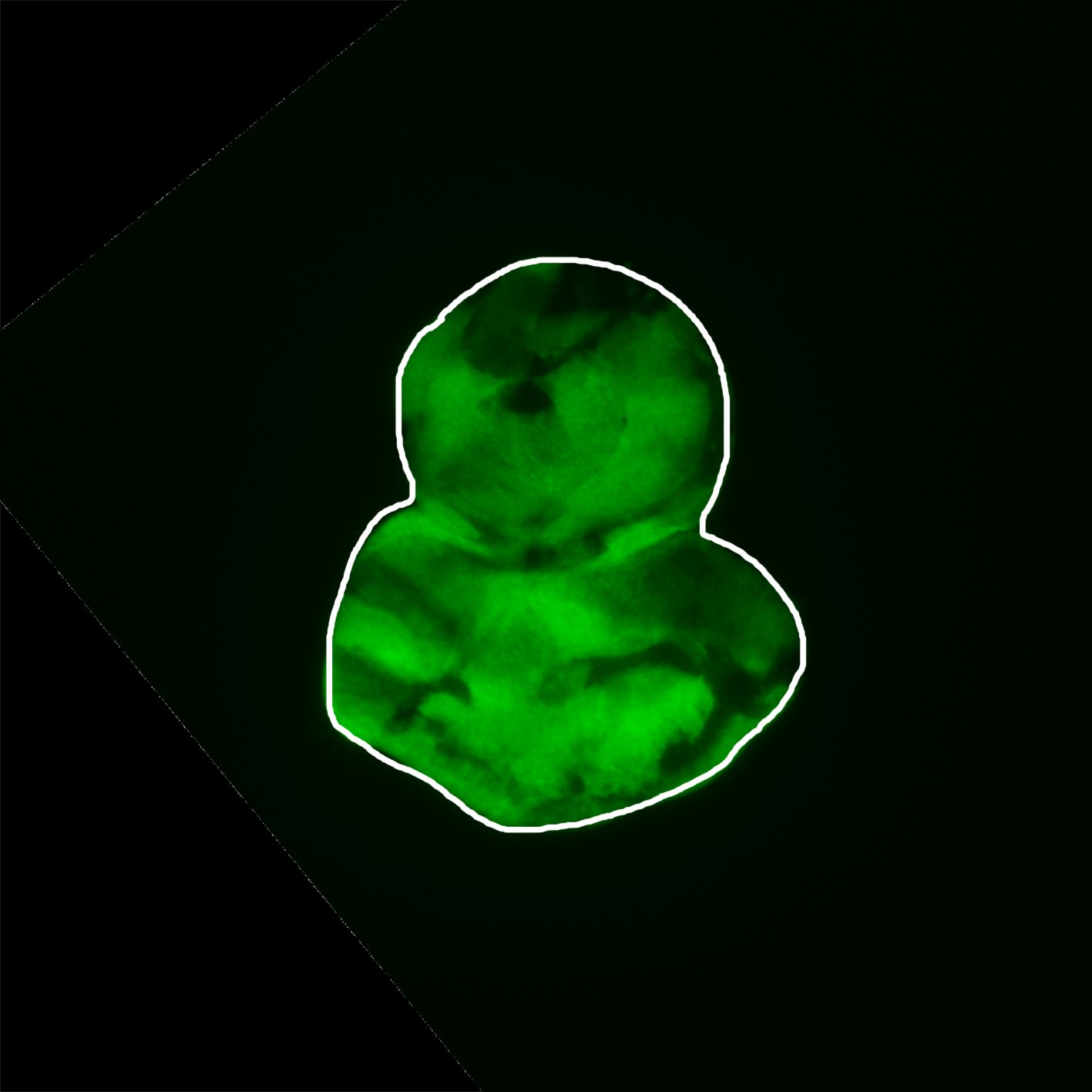

Supplement: Supplementary file 9 — Source data Fig. 5 [file 44318_2025_489_MOESM9_ESM.zip › Figure 5G/11-1 rotated and cut image with border line.tif]

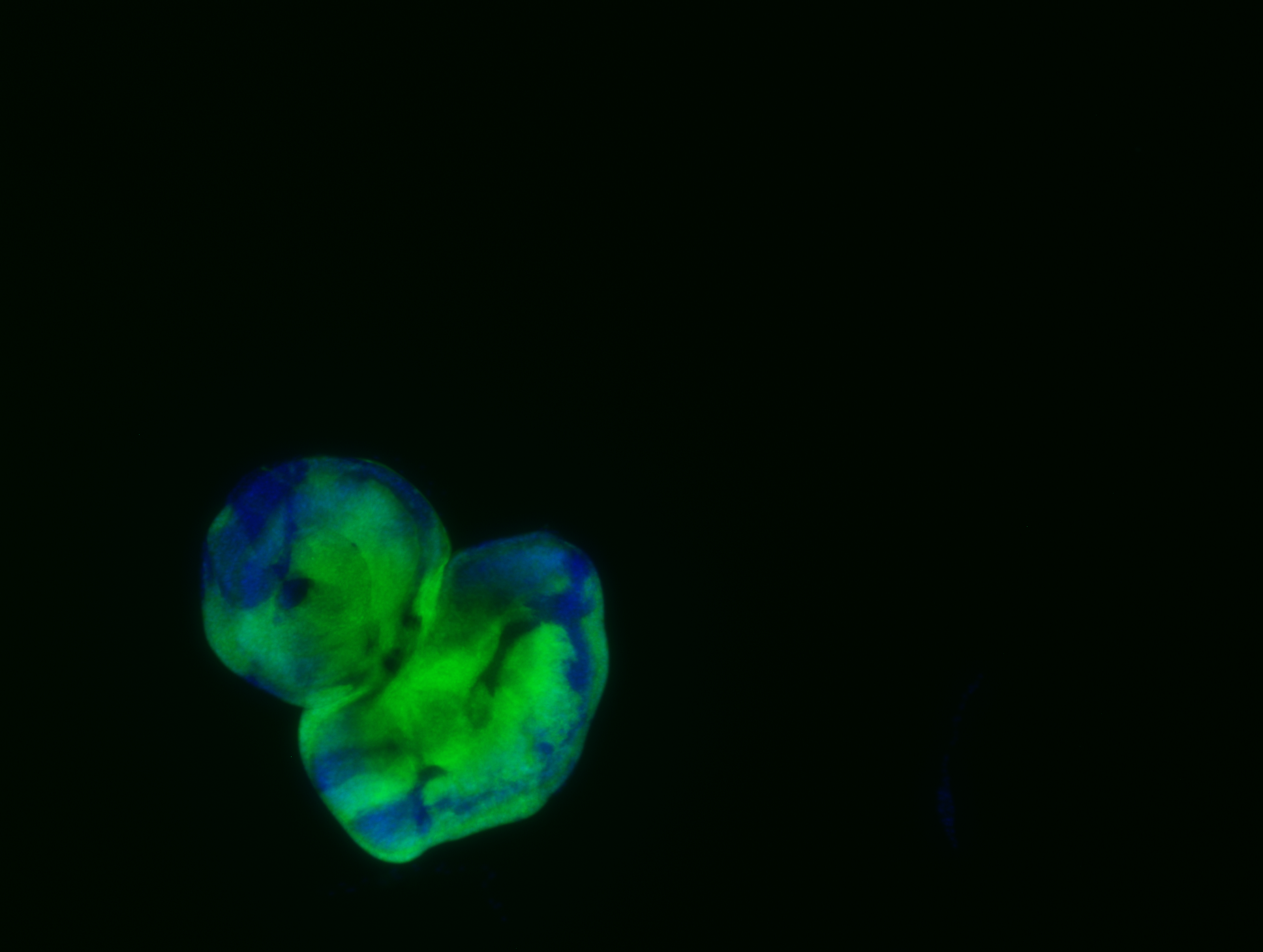

Supplement: Supplementary file 9 — Source data Fig. 5 [file 44318_2025_489_MOESM9_ESM.zip › Figure 5G/11-2 original image.tif]

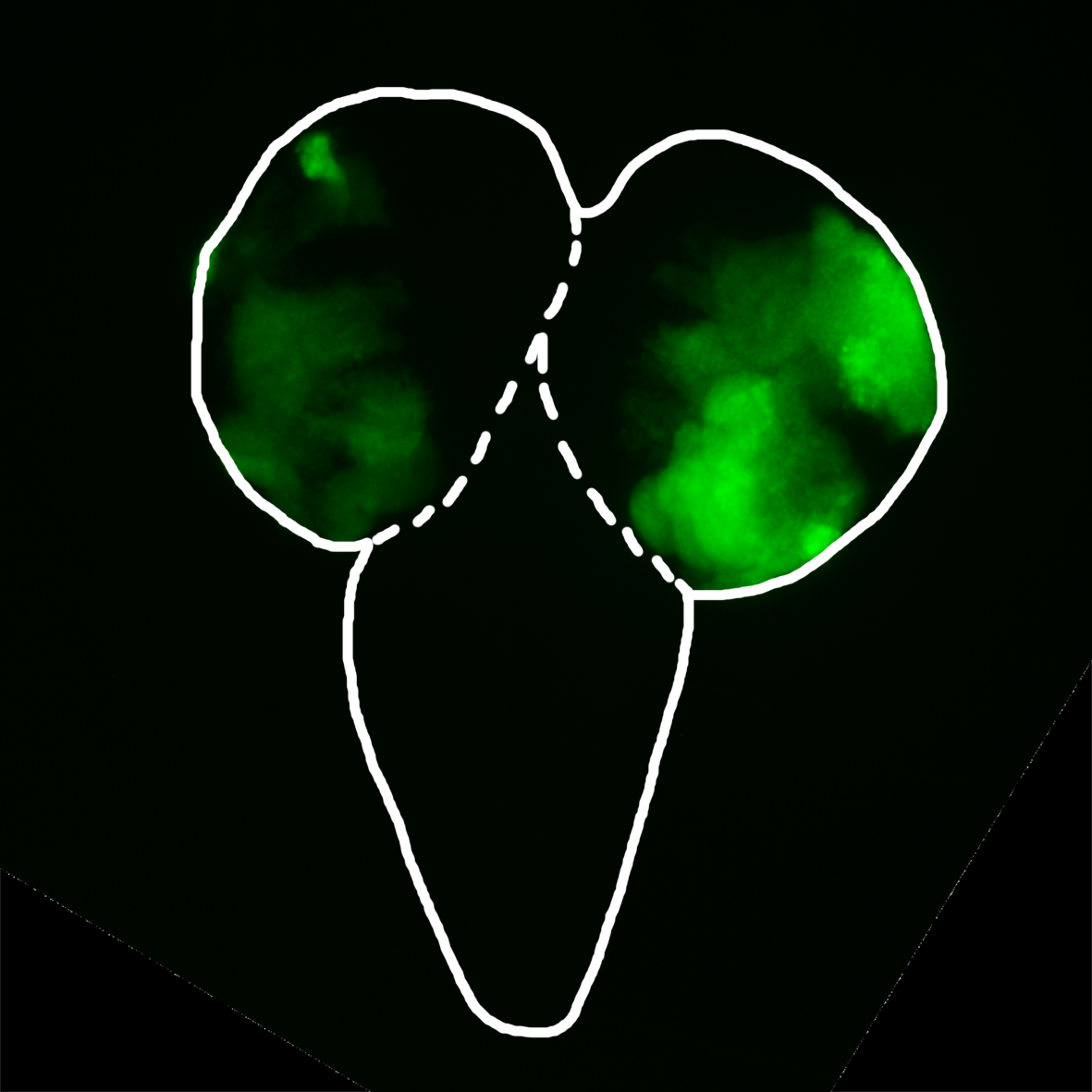

Supplement: Supplementary file 9 — Source data Fig. 5 [file 44318_2025_489_MOESM9_ESM.zip › Figure 5G/12-1 rotated and cut image with border line.tif]

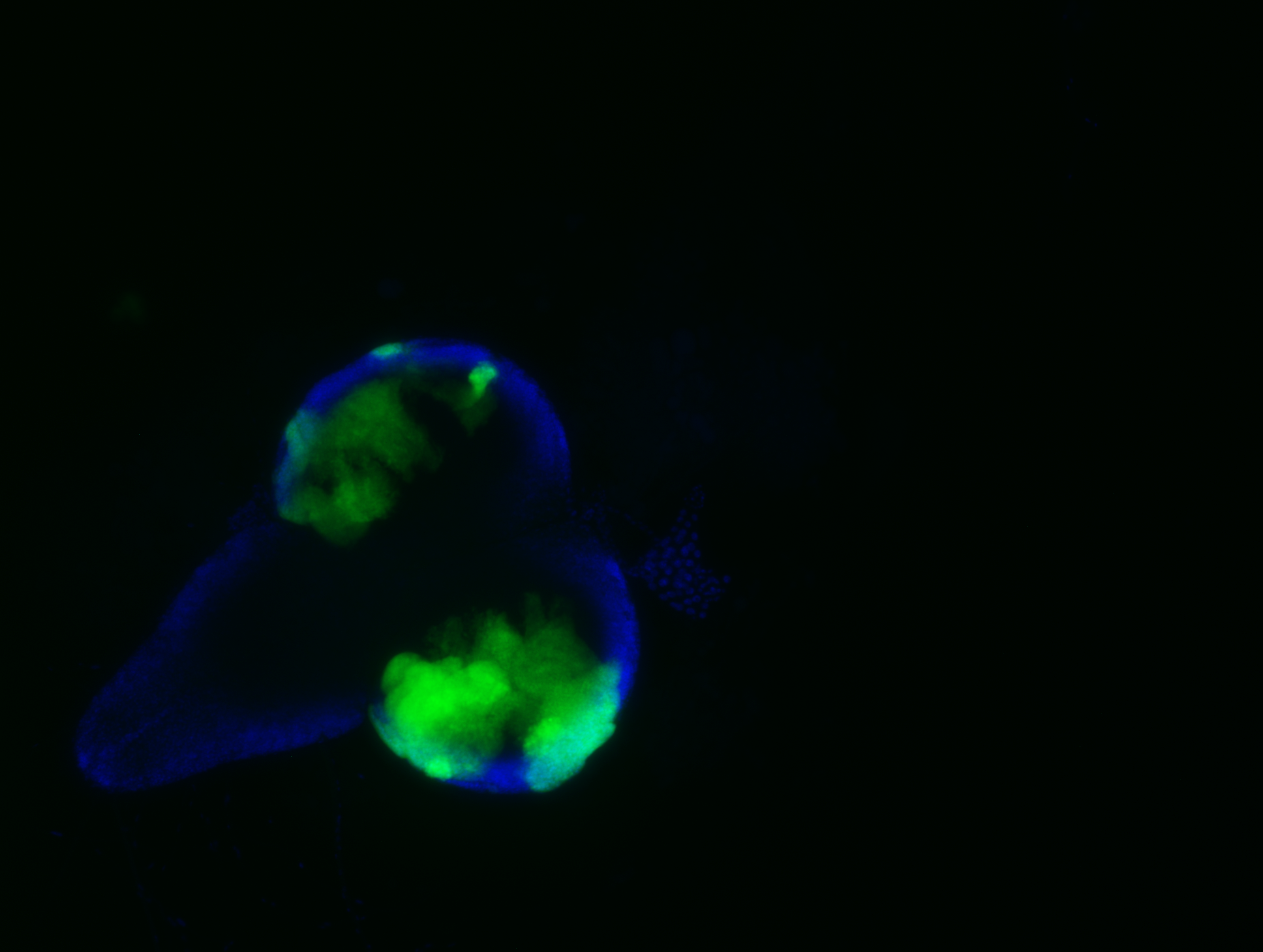

Supplement: Supplementary file 9 — Source data Fig. 5 [file 44318_2025_489_MOESM9_ESM.zip › Figure 5G/12-2 original image.tif]

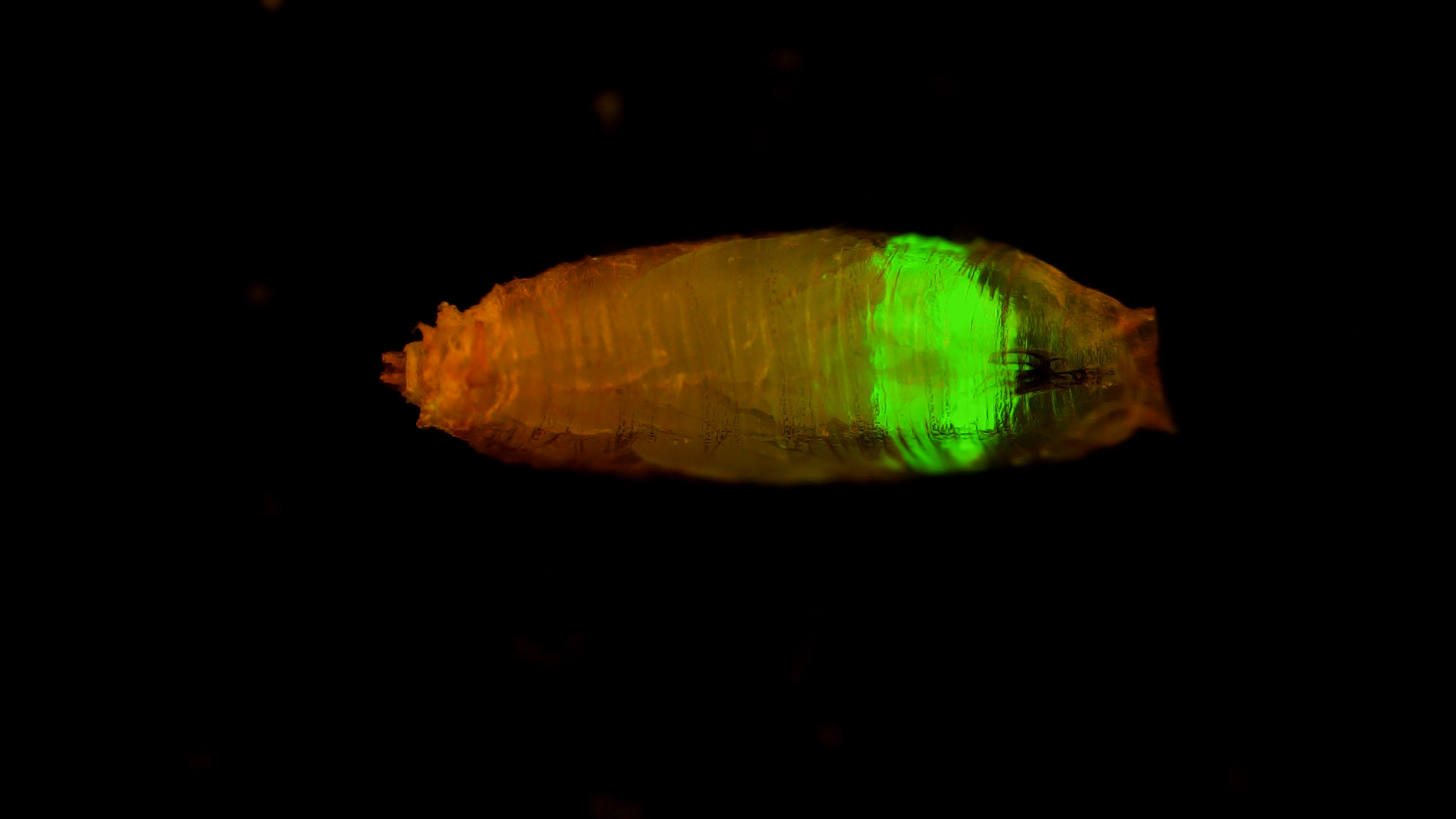

Supplement: Supplementary file 9 — Source data Fig. 5 [file 44318_2025_489_MOESM9_ESM.zip › Figure 5G/13 original image.tif]

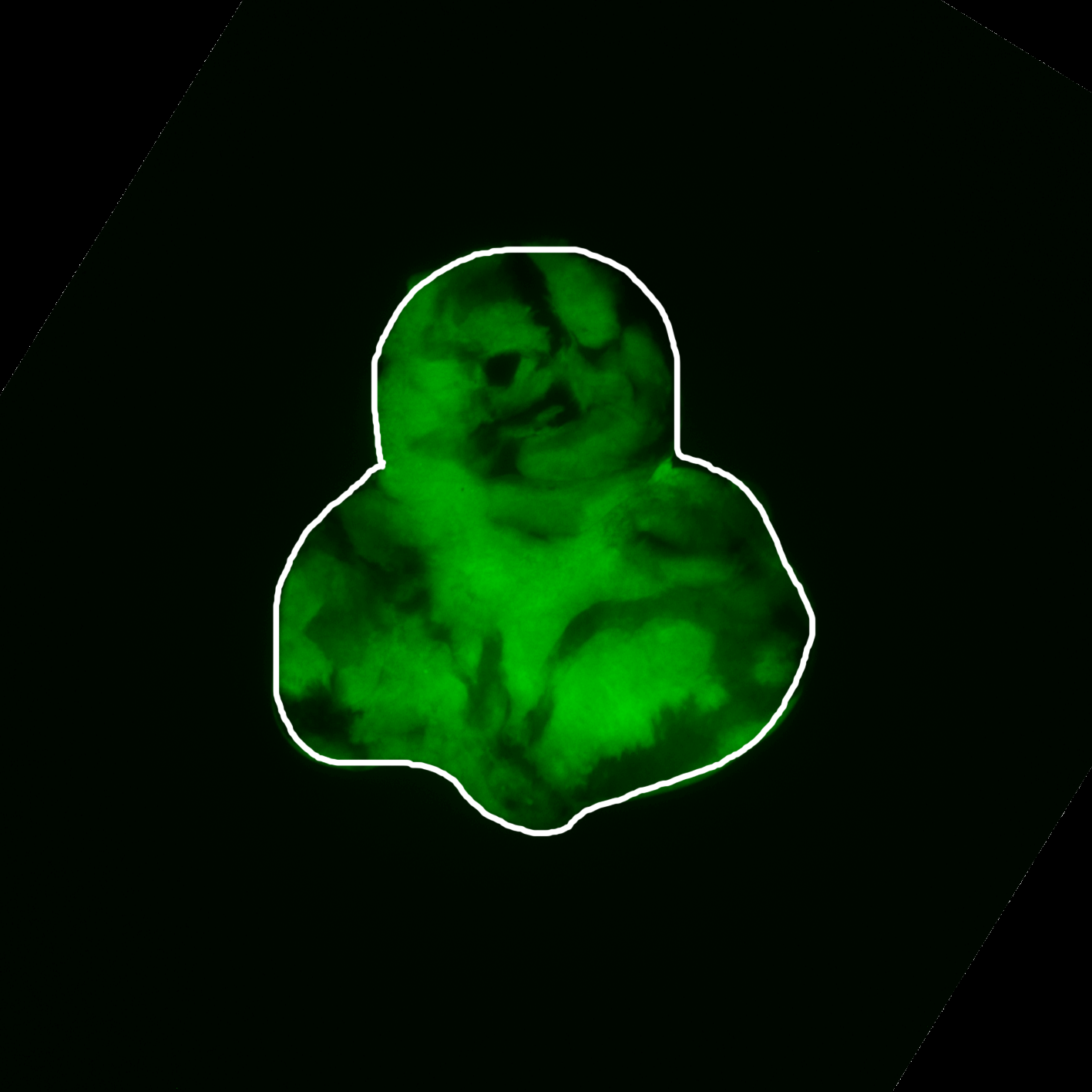

Supplement: Supplementary file 9 — Source data Fig. 5 [file 44318_2025_489_MOESM9_ESM.zip › Figure 5G/14-1 rotated and cut image with border line.tif]

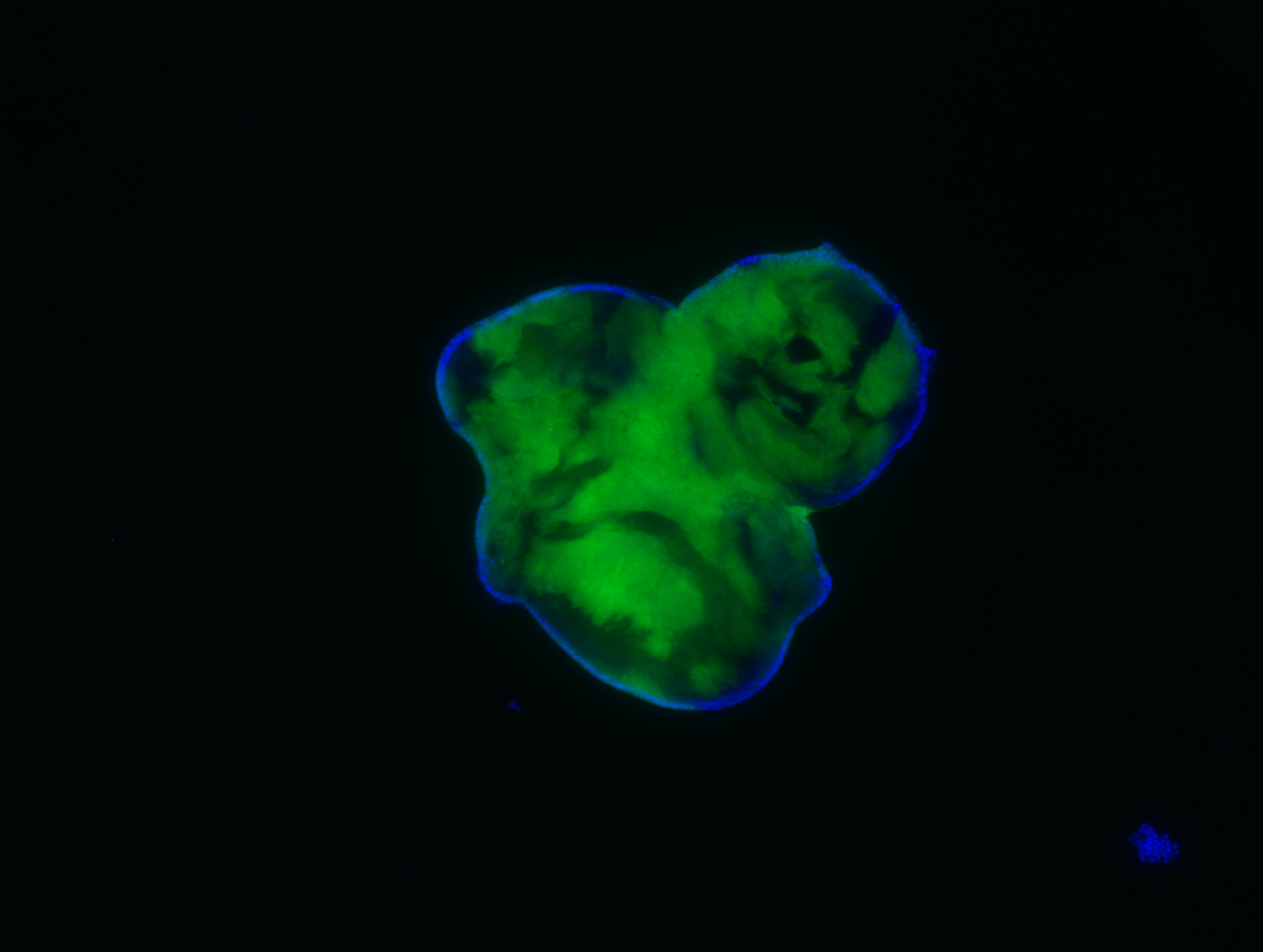

Supplement: Supplementary file 9 — Source data Fig. 5 [file 44318_2025_489_MOESM9_ESM.zip › Figure 5G/14-2 original image.tif]

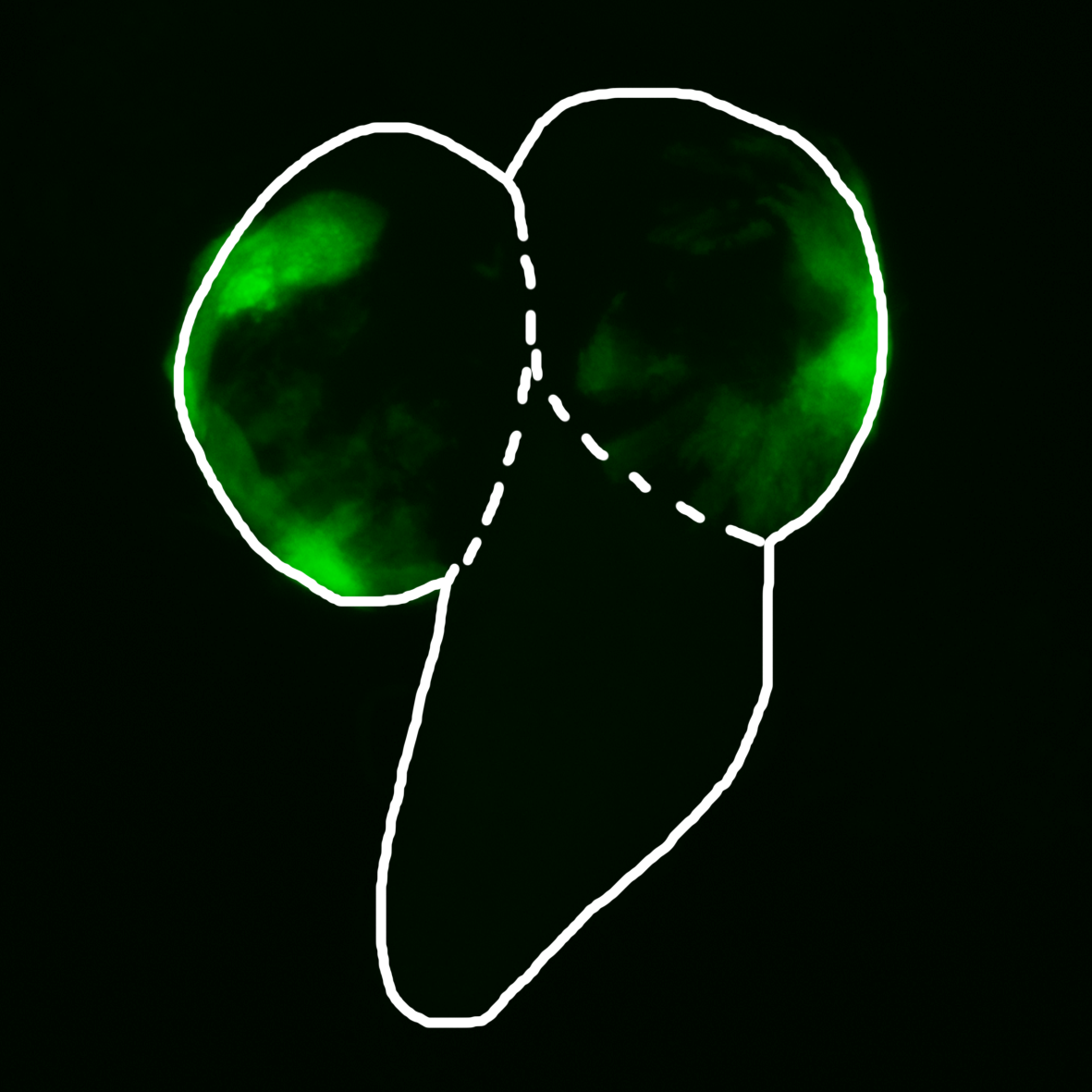

Supplement: Supplementary file 9 — Source data Fig. 5 [file 44318_2025_489_MOESM9_ESM.zip › Figure 5G/15-1 rotated and cut image with border line.tif]

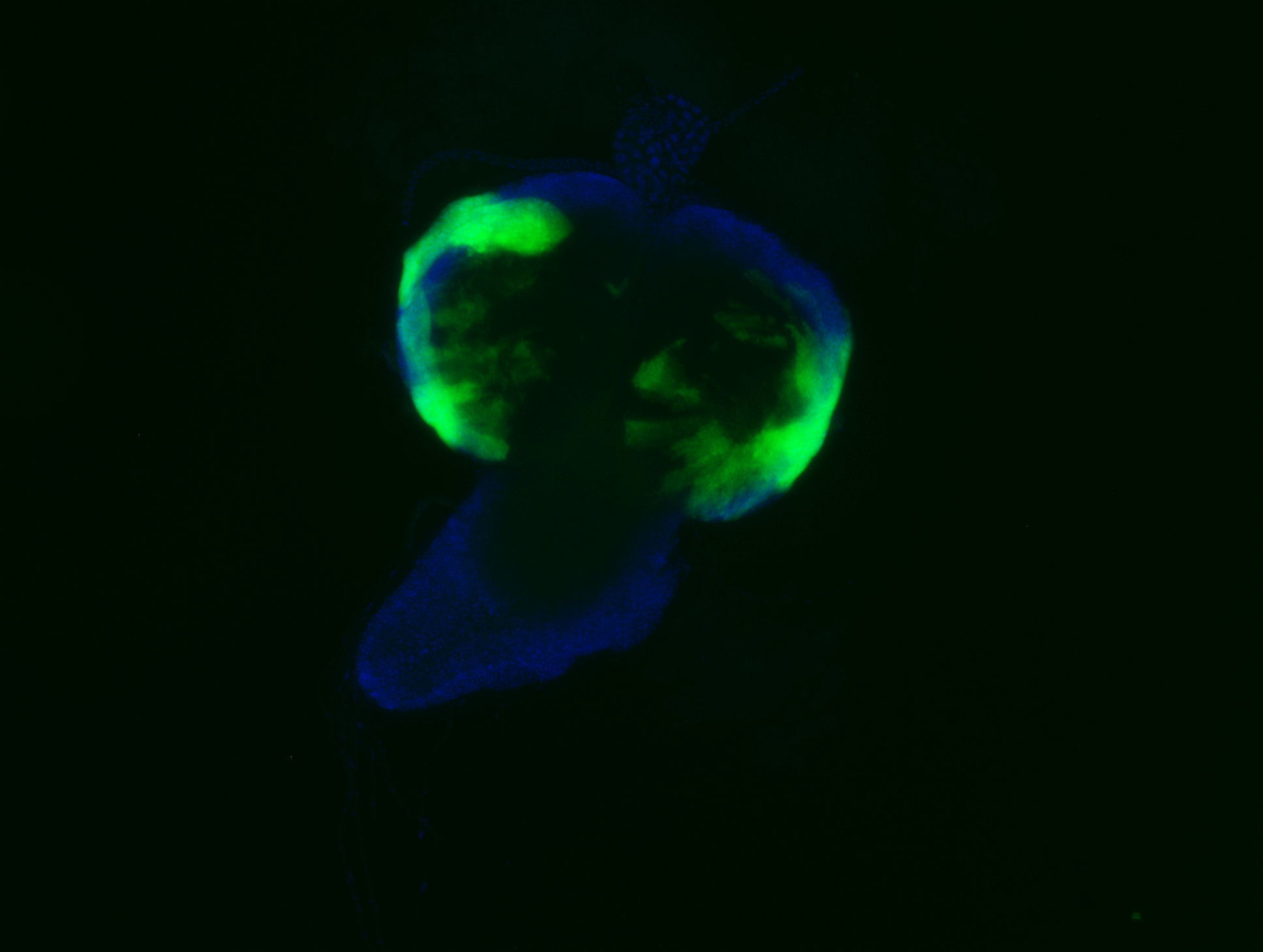

Supplement: Supplementary file 9 — Source data Fig. 5 [file 44318_2025_489_MOESM9_ESM.zip › Figure 5G/15-2 original image.tif]

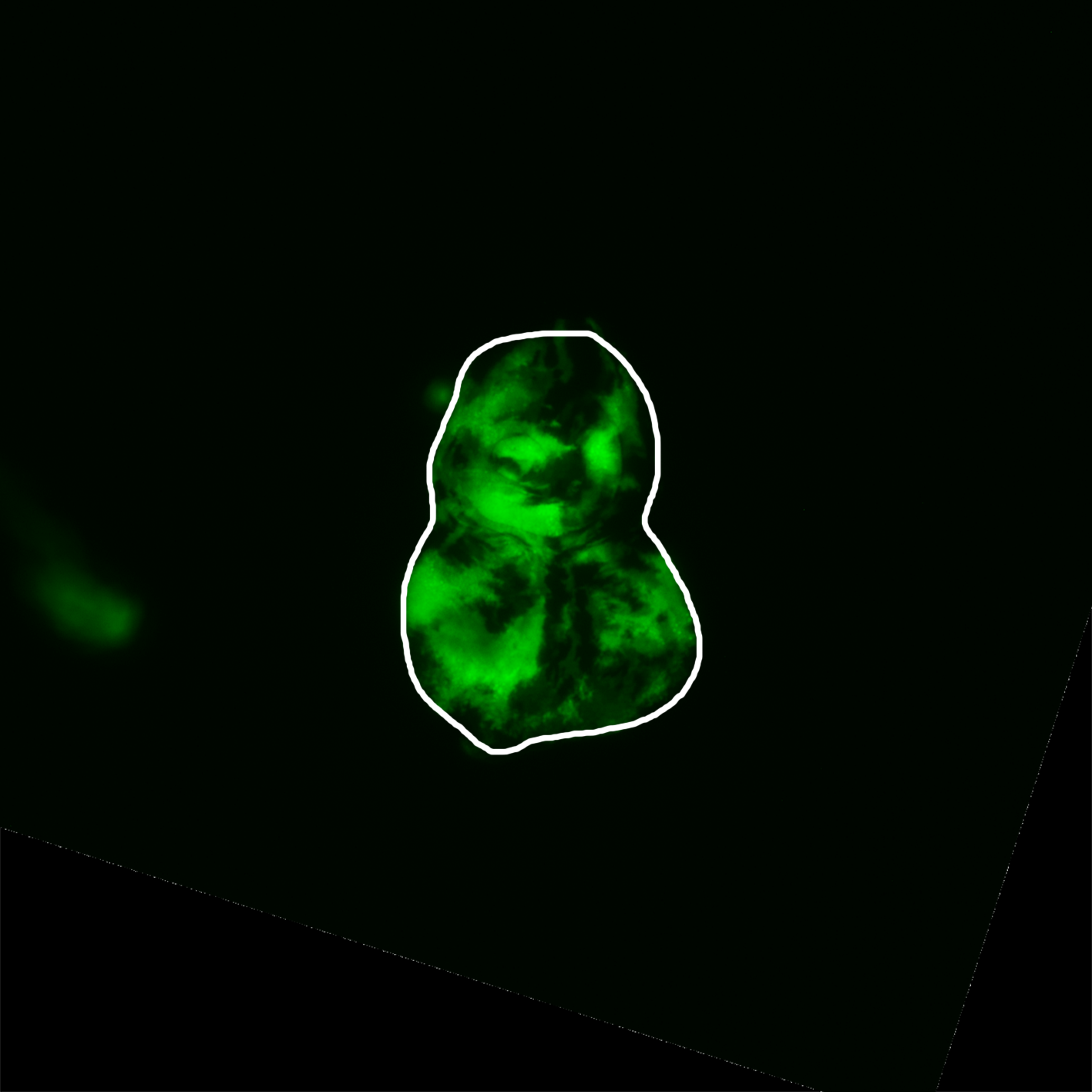

Supplement: Supplementary file 9 — Source data Fig. 5 [file 44318_2025_489_MOESM9_ESM.zip › Figure 5G/2-1 rotated and cut image with border line.tif]

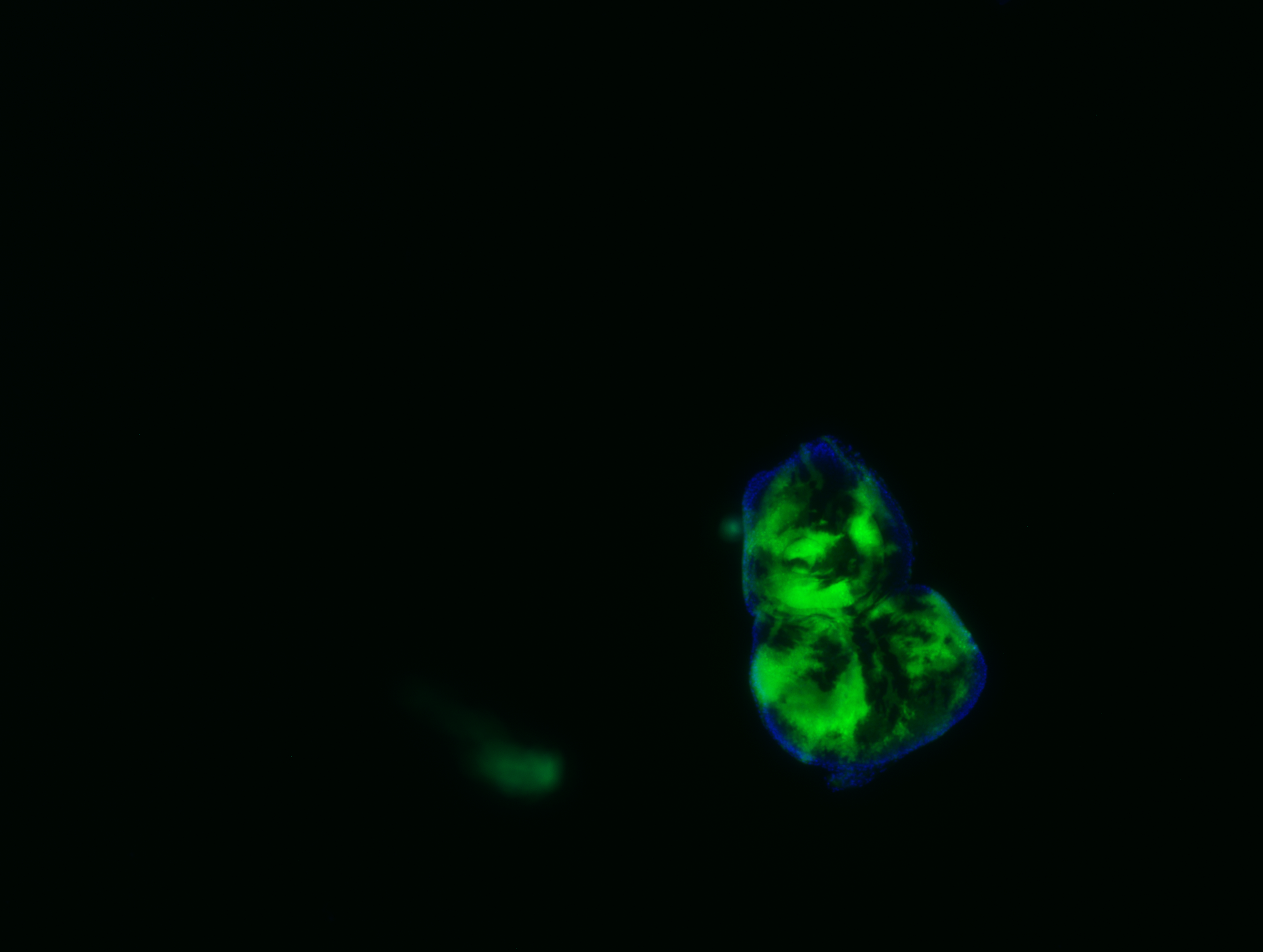

Supplement: Supplementary file 9 — Source data Fig. 5 [file 44318_2025_489_MOESM9_ESM.zip › Figure 5G/2-2 original image.tif]

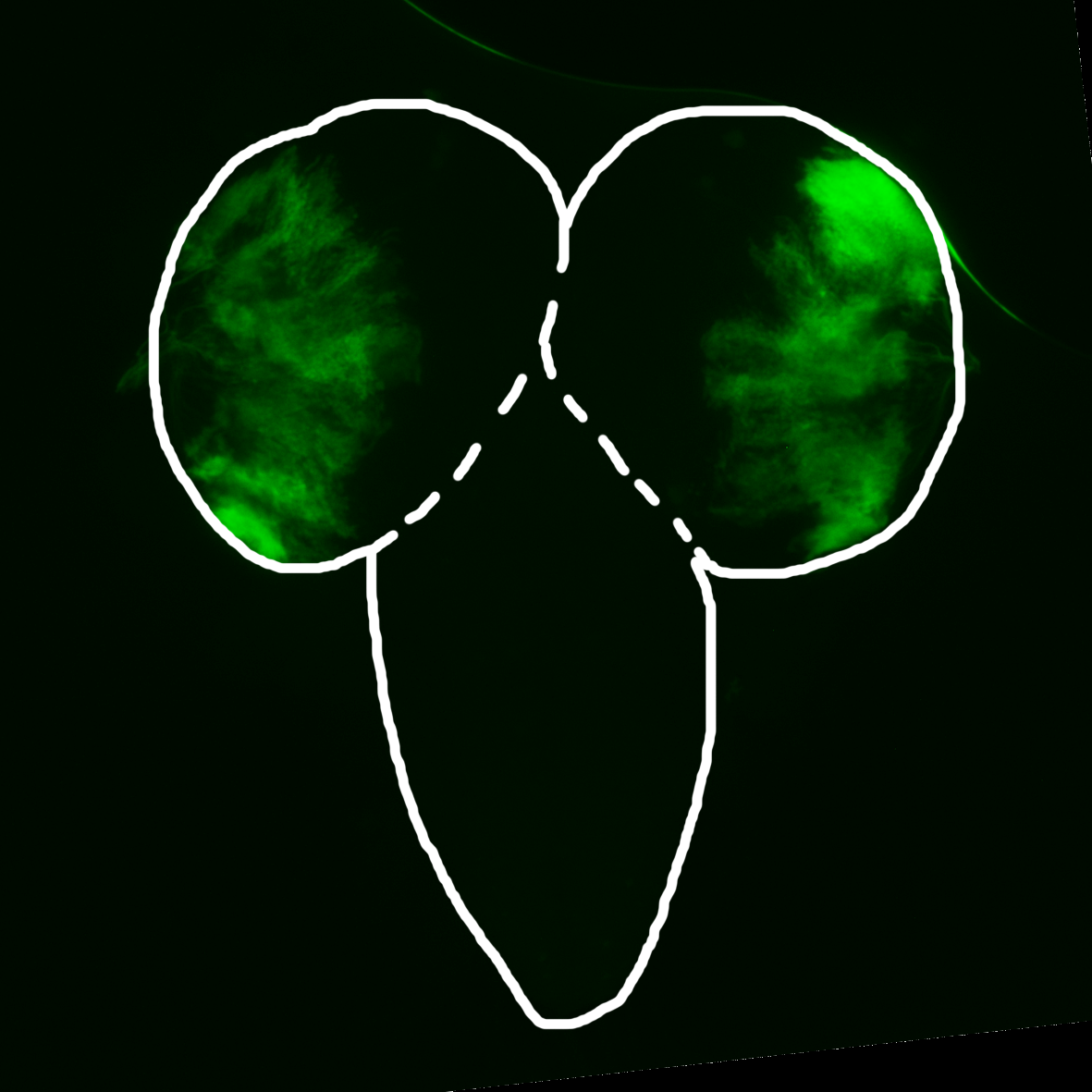

Supplement: Supplementary file 9 — Source data Fig. 5 [file 44318_2025_489_MOESM9_ESM.zip › Figure 5G/3-1 rotated and cut image with border line.tif]

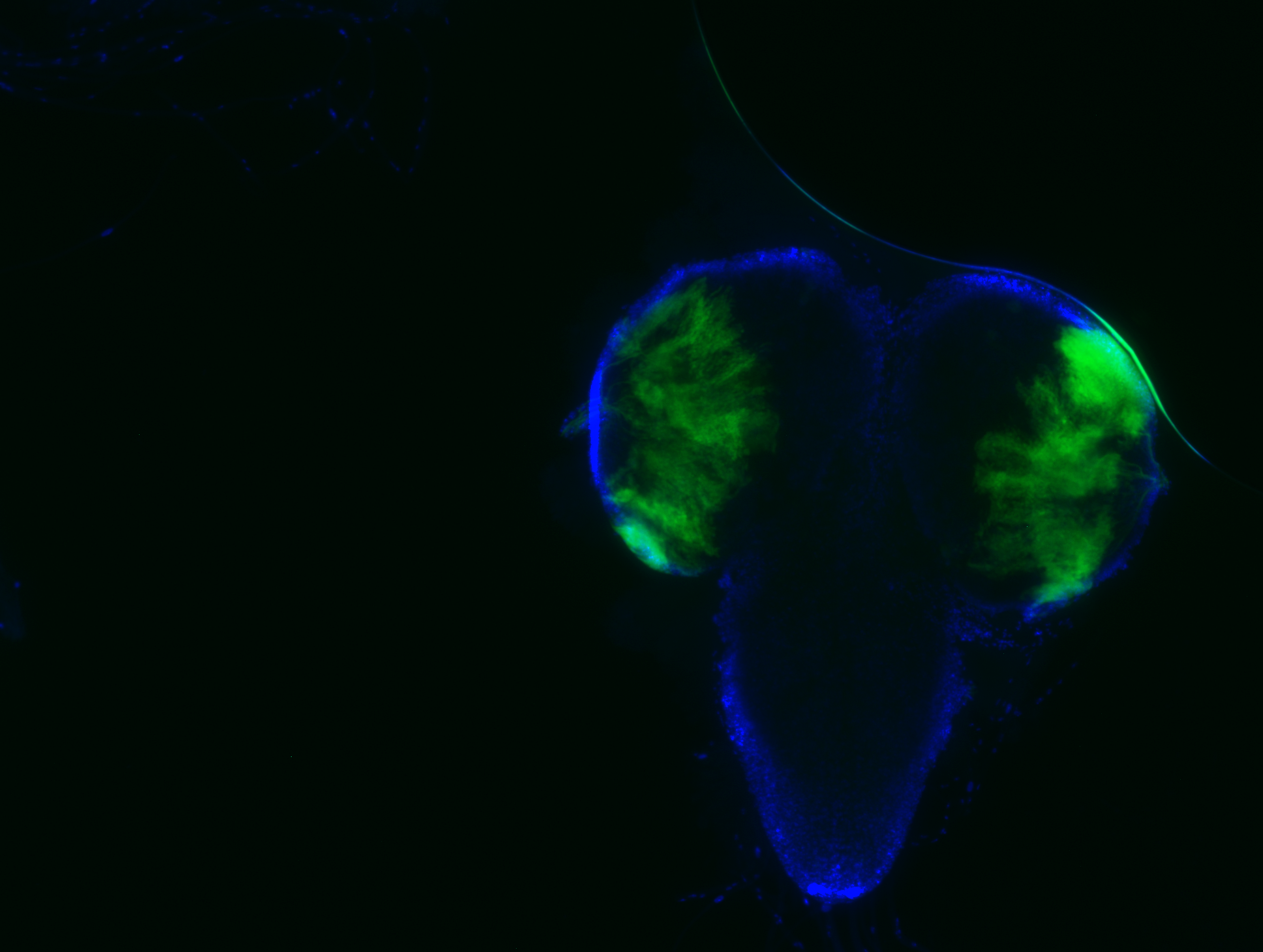

Supplement: Supplementary file 9 — Source data Fig. 5 [file 44318_2025_489_MOESM9_ESM.zip › Figure 5G/3-2 original image.tif]

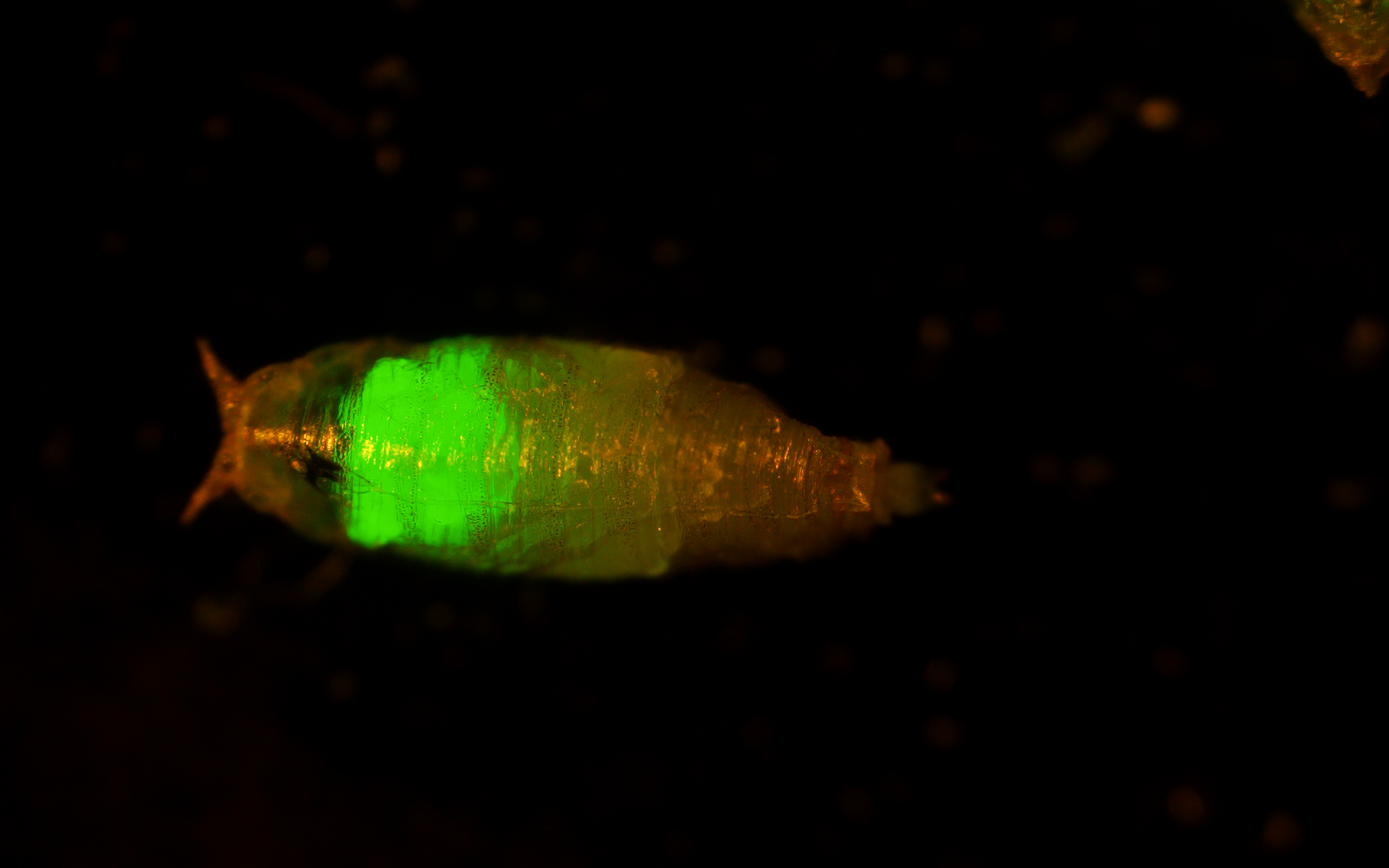

Supplement: Supplementary file 9 — Source data Fig. 5 [file 44318_2025_489_MOESM9_ESM.zip › Figure 5G/4 original image.tif]

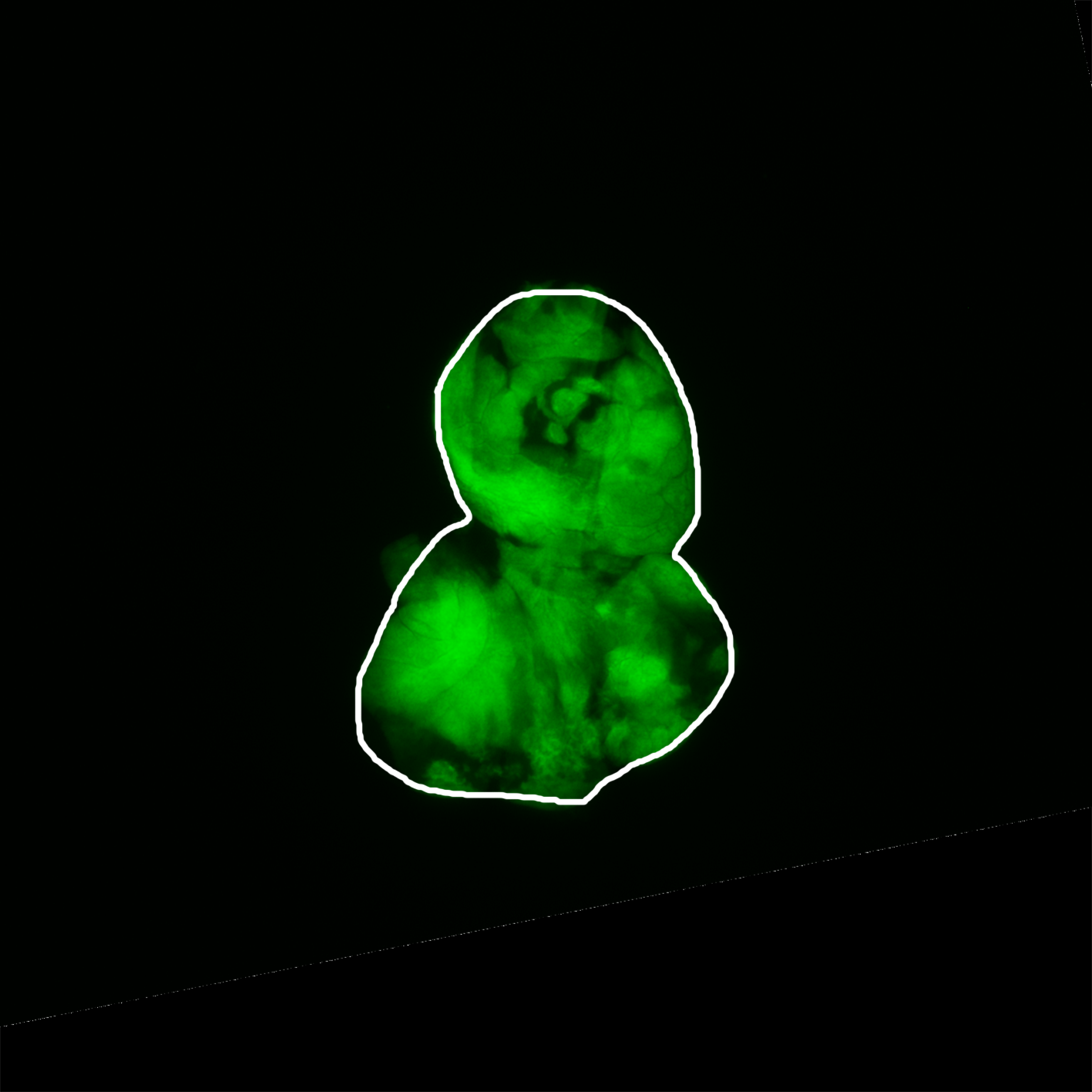

Supplement: Supplementary file 9 — Source data Fig. 5 [file 44318_2025_489_MOESM9_ESM.zip › Figure 5G/5-1 rotated and cut image with border line.tif]

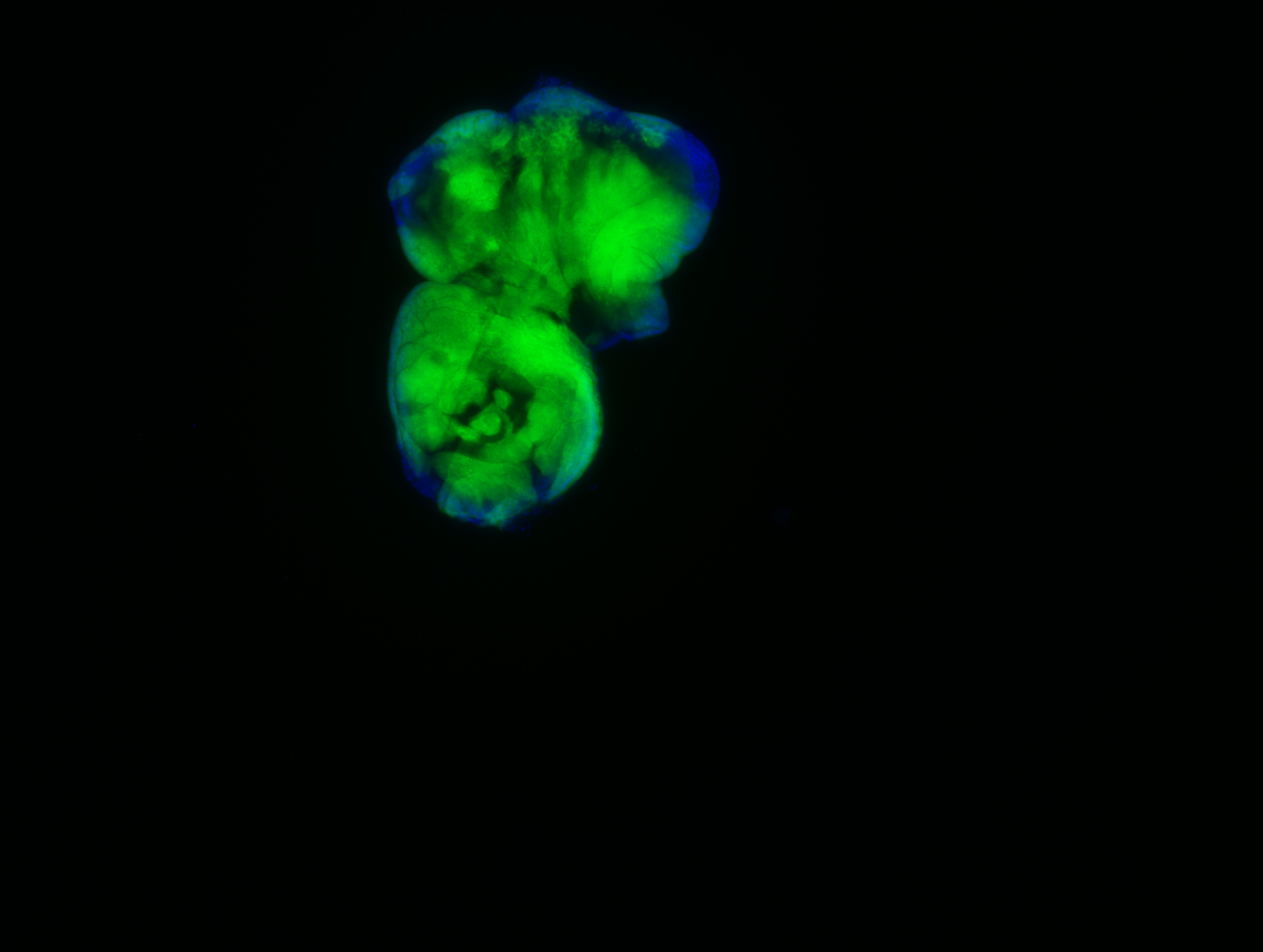

Supplement: Supplementary file 9 — Source data Fig. 5 [file 44318_2025_489_MOESM9_ESM.zip › Figure 5G/5-2 original image.tif]

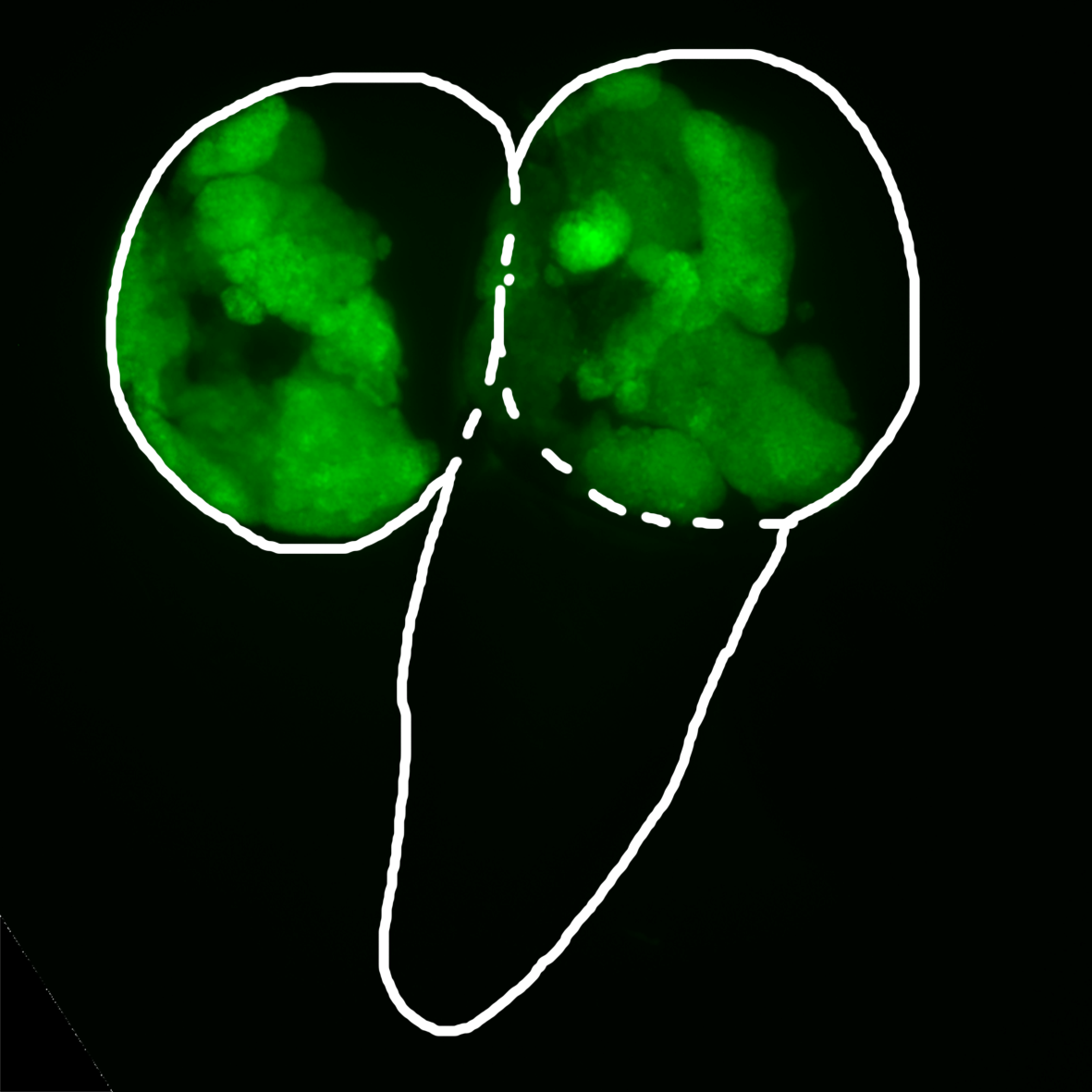

Supplement: Supplementary file 9 — Source data Fig. 5 [file 44318_2025_489_MOESM9_ESM.zip › Figure 5G/6-1 rotated and cut image with border line.tif]

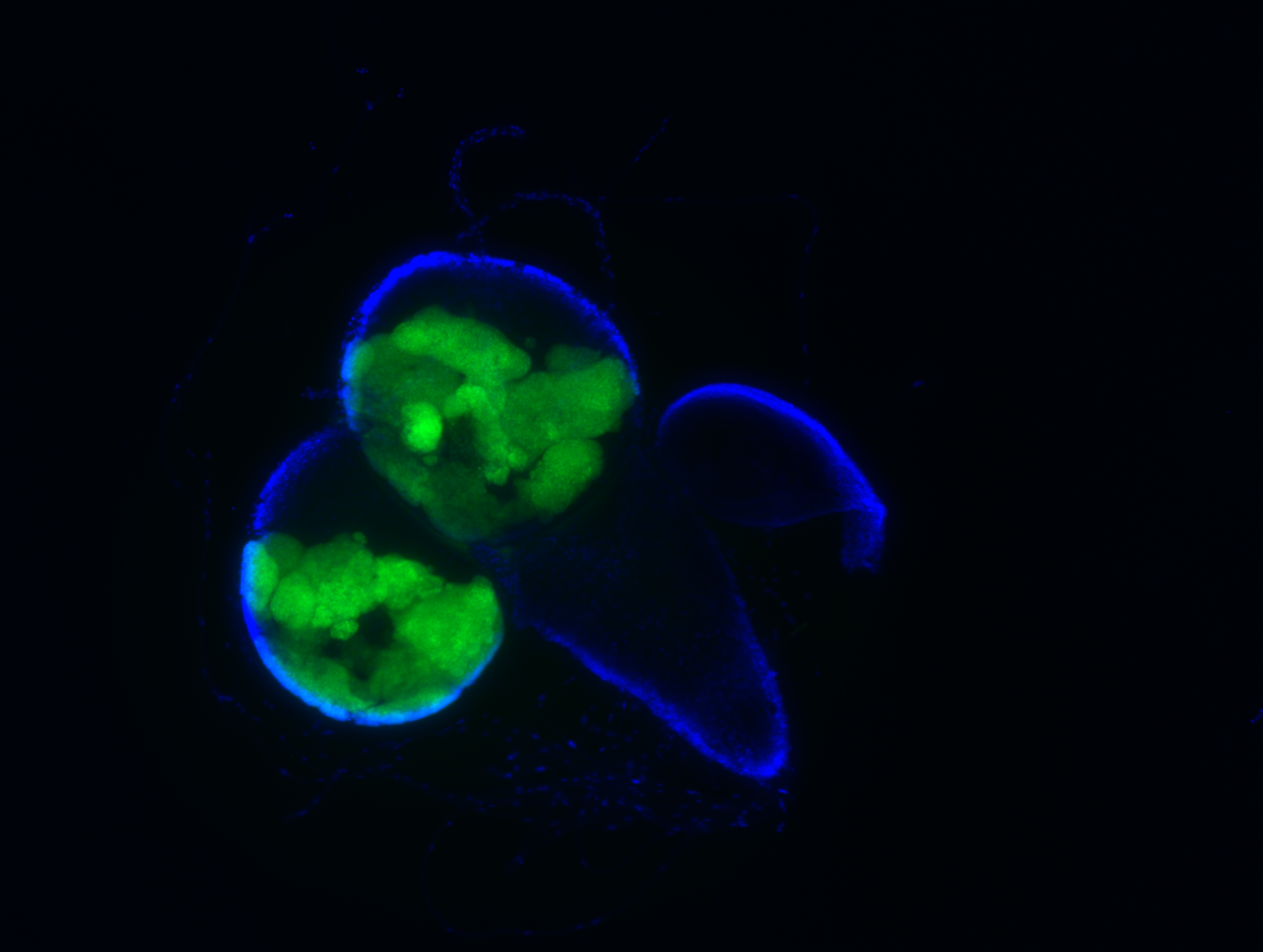

Supplement: Supplementary file 9 — Source data Fig. 5 [file 44318_2025_489_MOESM9_ESM.zip › Figure 5G/6-2 original image.tif]

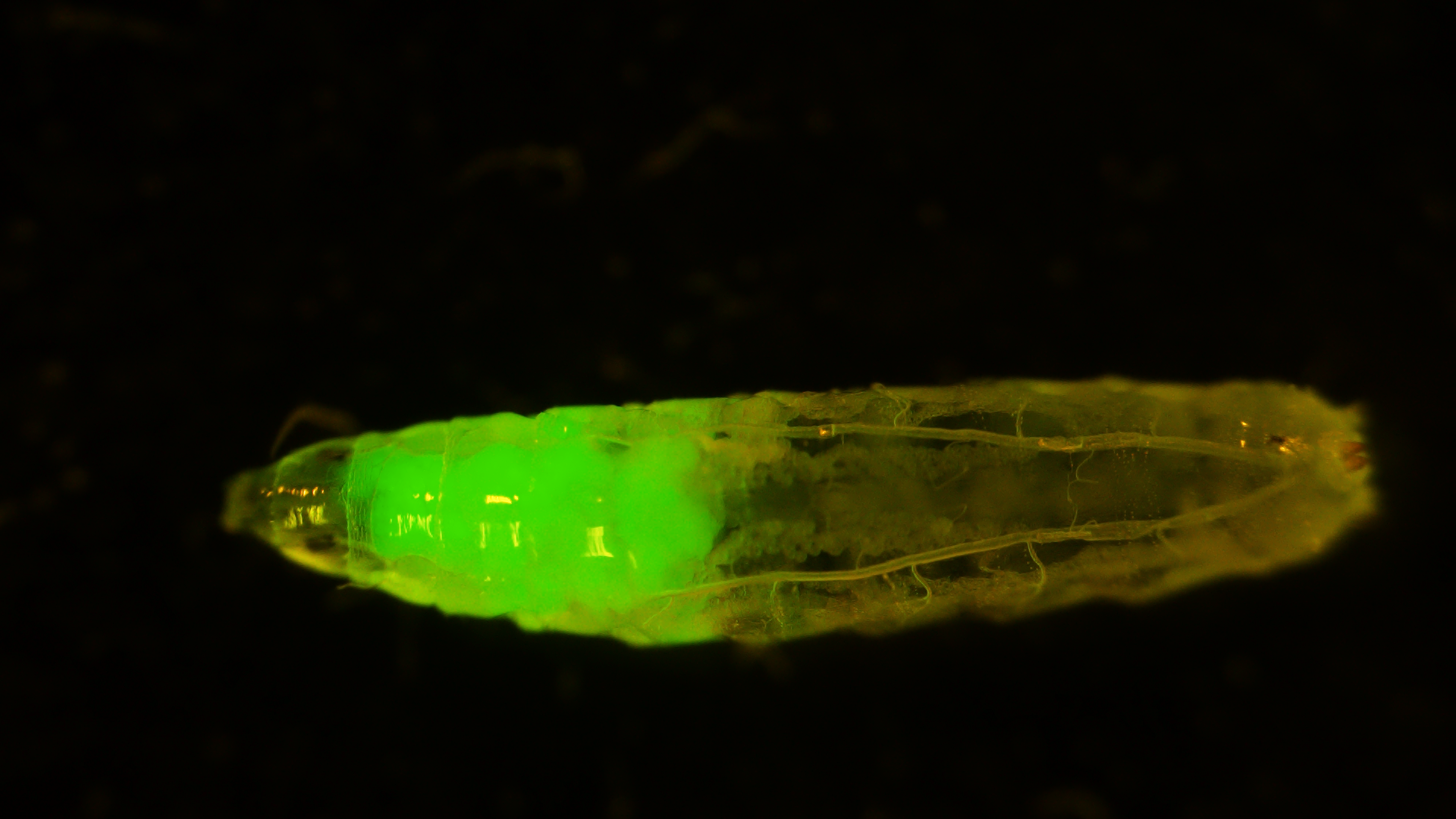

Supplement: Supplementary file 9 — Source data Fig. 5 [file 44318_2025_489_MOESM9_ESM.zip › Figure 5G/7 original image.tif]

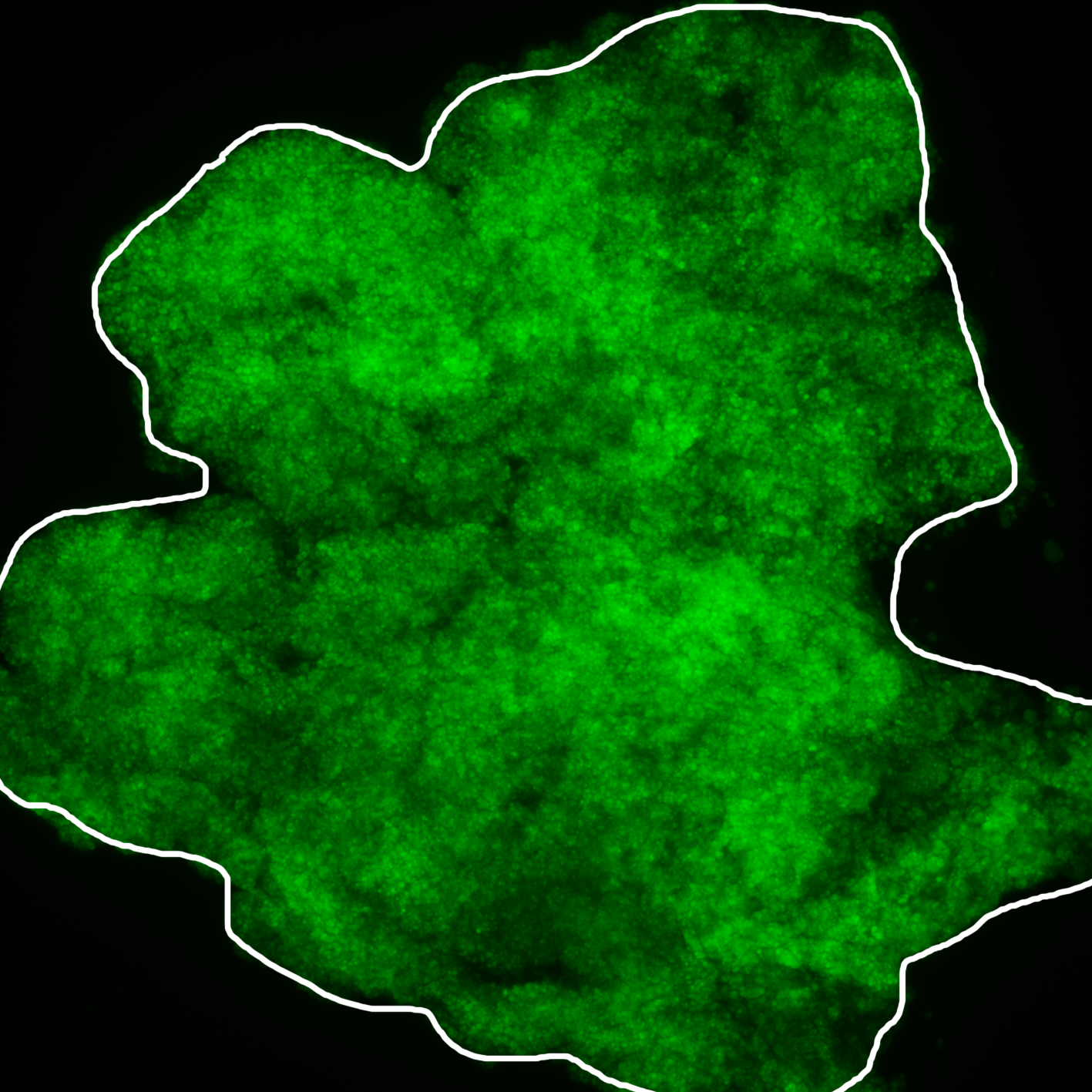

Supplement: Supplementary file 9 — Source data Fig. 5 [file 44318_2025_489_MOESM9_ESM.zip › Figure 5G/8-1 rotated and cut image with border line.tif]

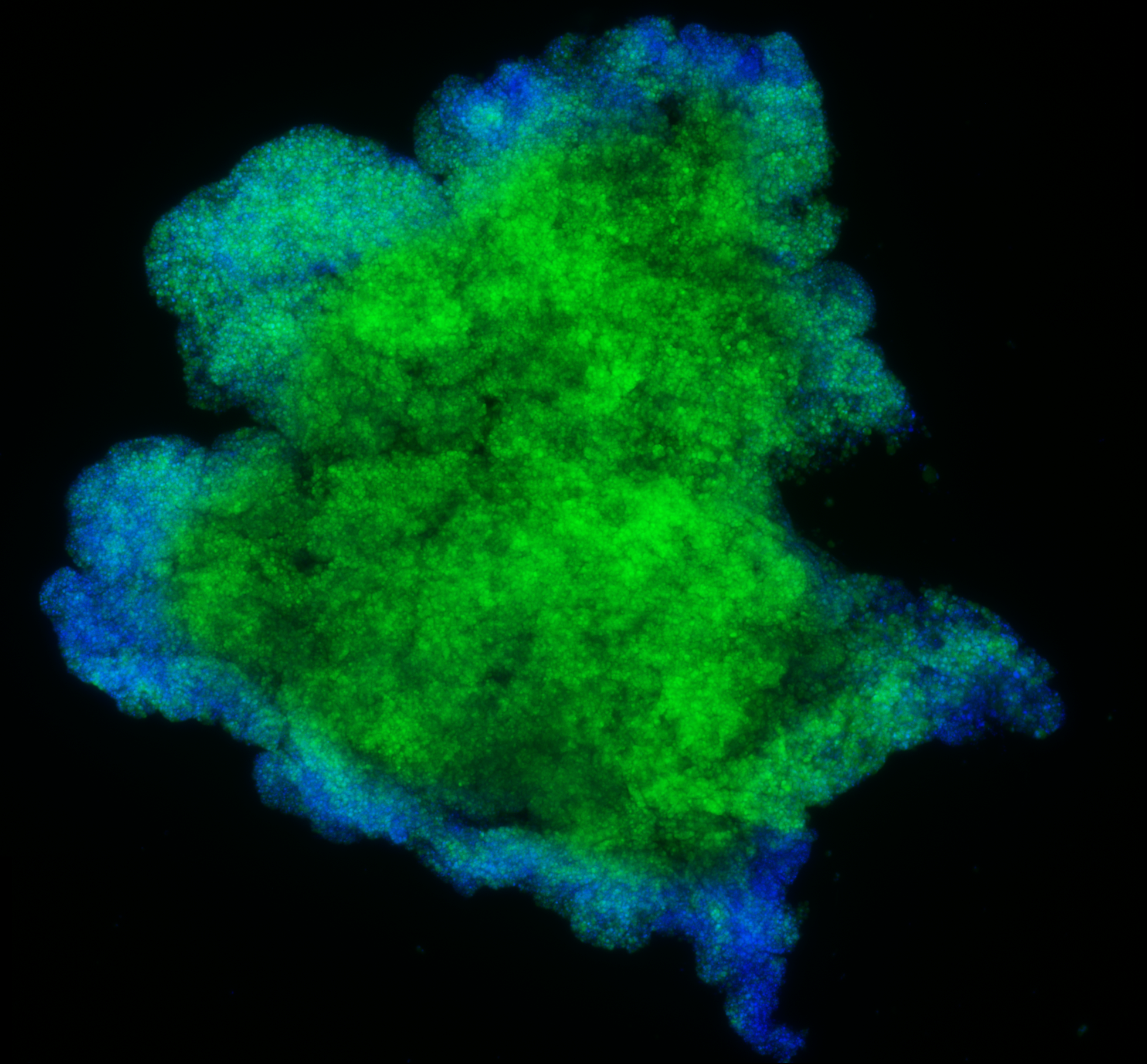

Supplement: Supplementary file 9 — Source data Fig. 5 [file 44318_2025_489_MOESM9_ESM.zip › Figure 5G/8-2 original image.tif]

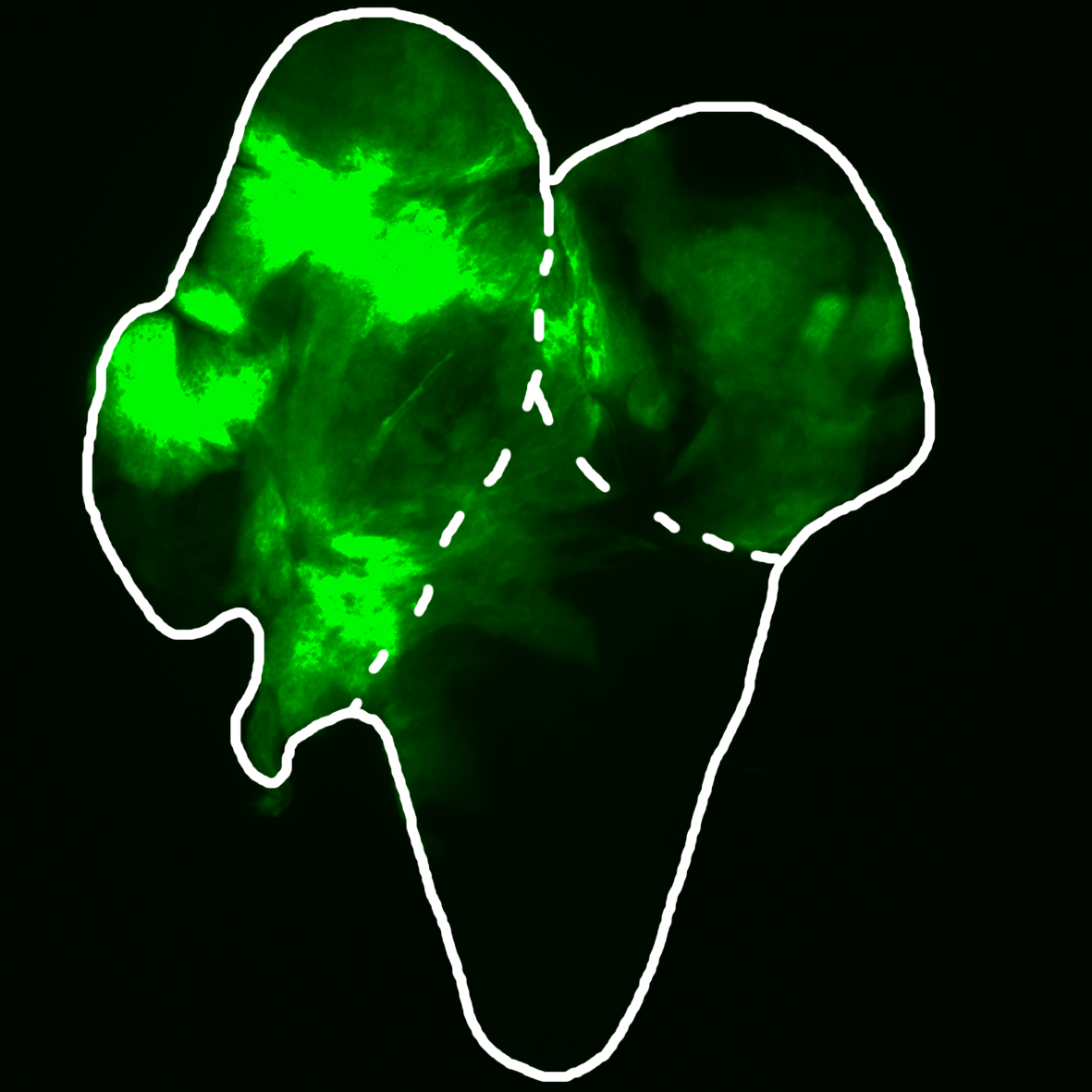

Supplement: Supplementary file 9 — Source data Fig. 5 [file 44318_2025_489_MOESM9_ESM.zip › Figure 5G/9-1 rotated and cut image with border line.tif]

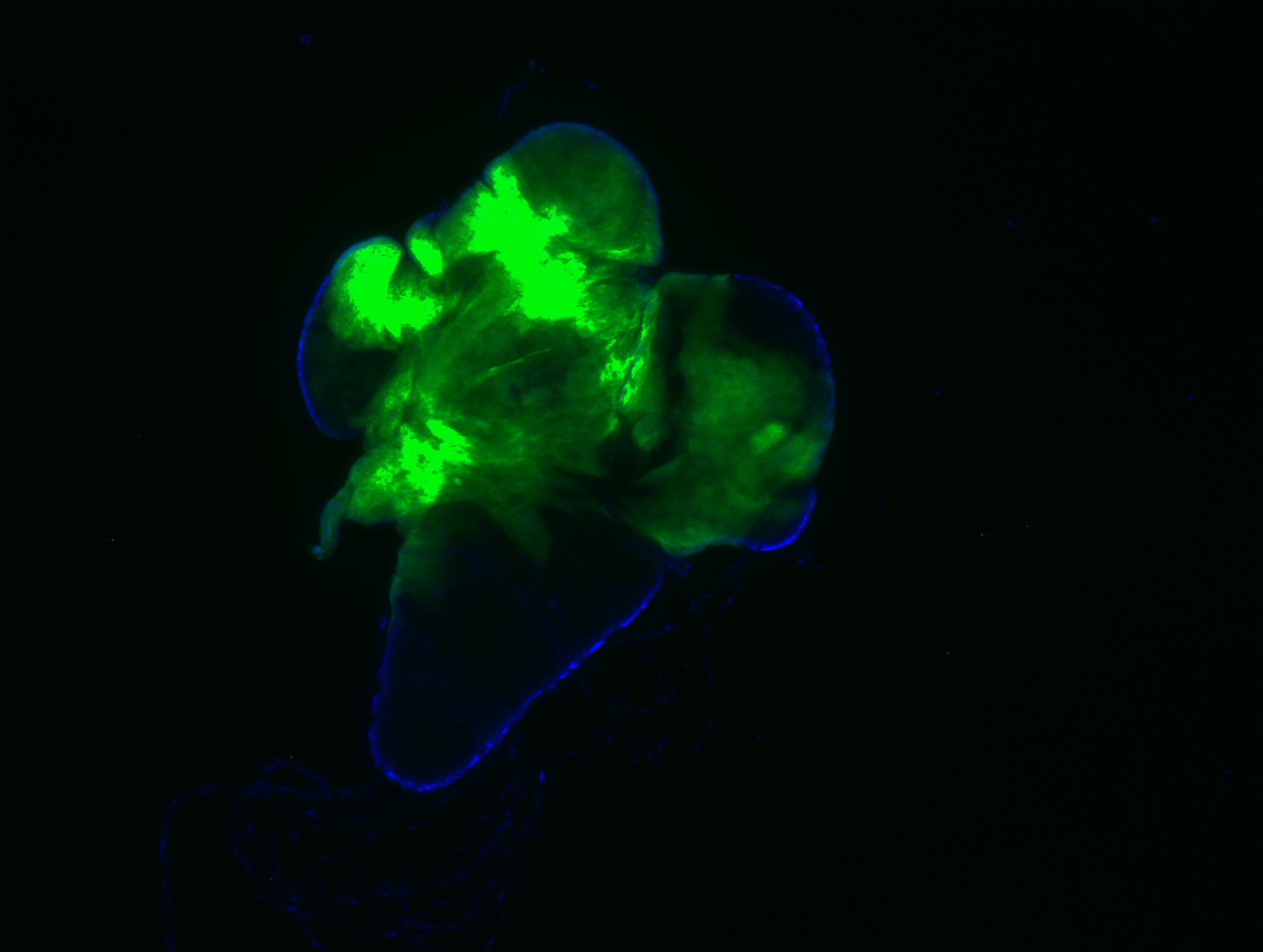

Supplement: Supplementary file 9 — Source data Fig. 5 [file 44318_2025_489_MOESM9_ESM.zip › Figure 5G/9-2 original image.tif]

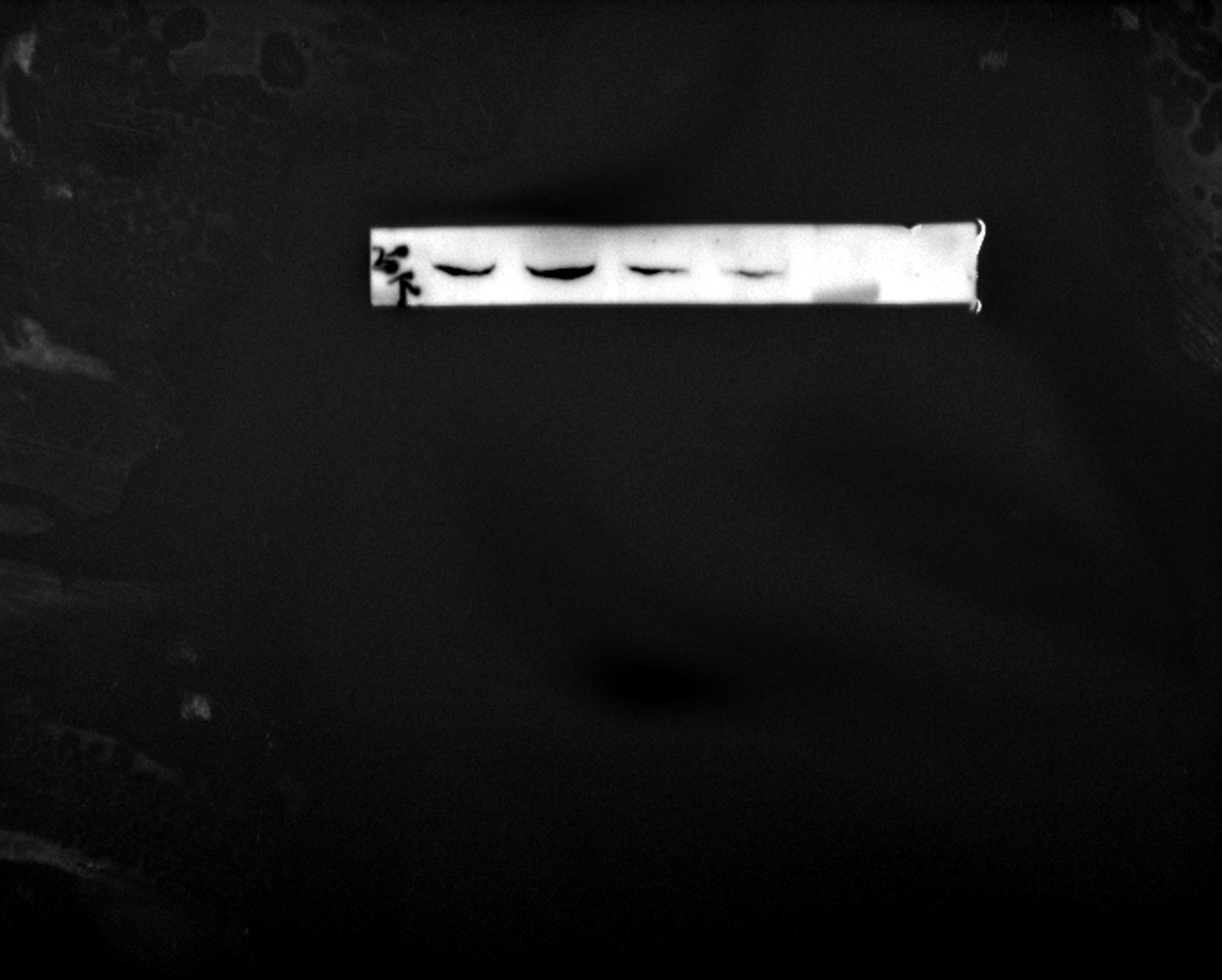

Supplement: Supplementary file 9 — Source data Fig. 5 [file 44318_2025_489_MOESM9_ESM.zip › Figure 5A/1. western Ap-2α .Tif]

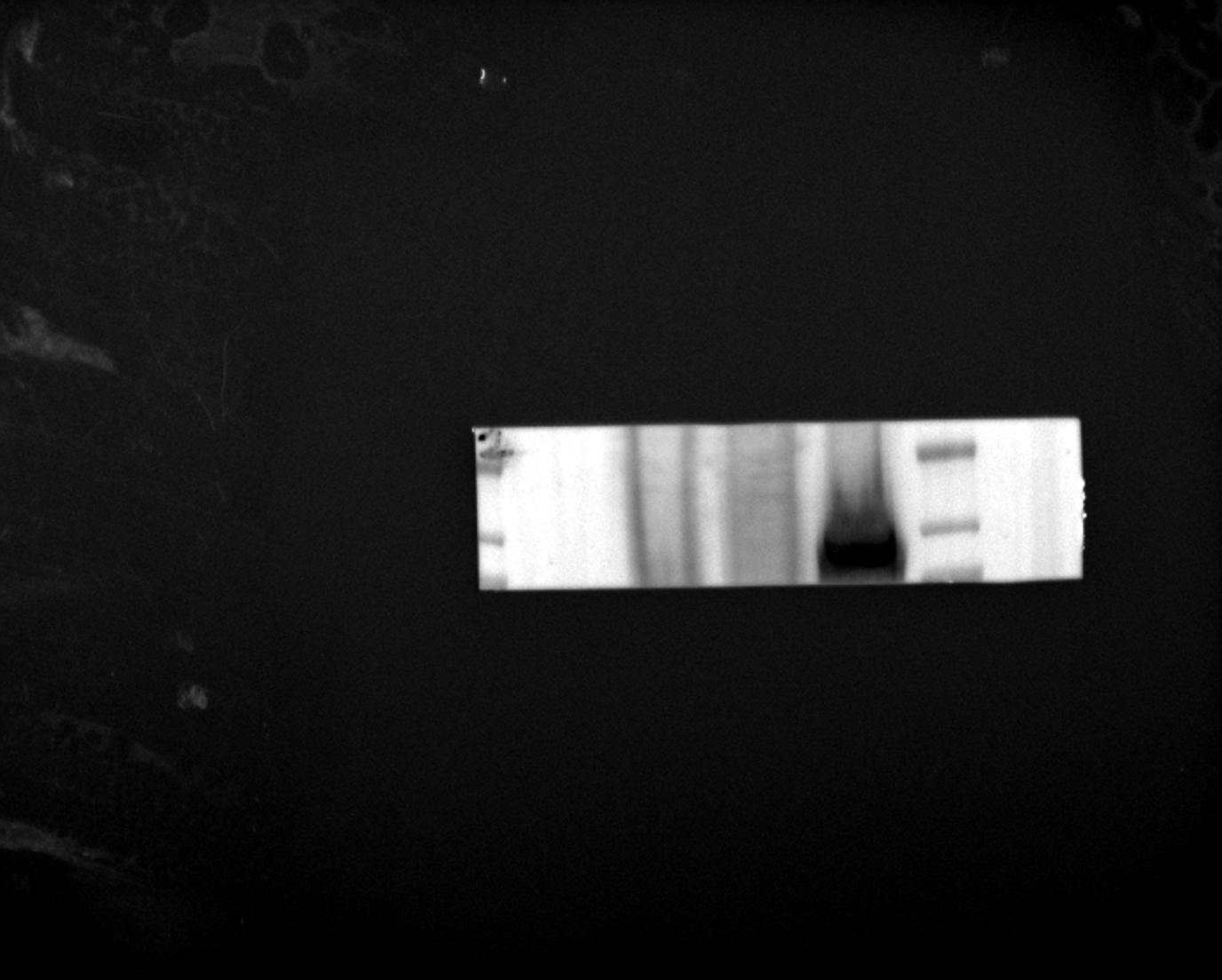

Supplement: Supplementary file 9 — Source data Fig. 5 [file 44318_2025_489_MOESM9_ESM.zip › Figure 5A/2.western mib1.Tif]

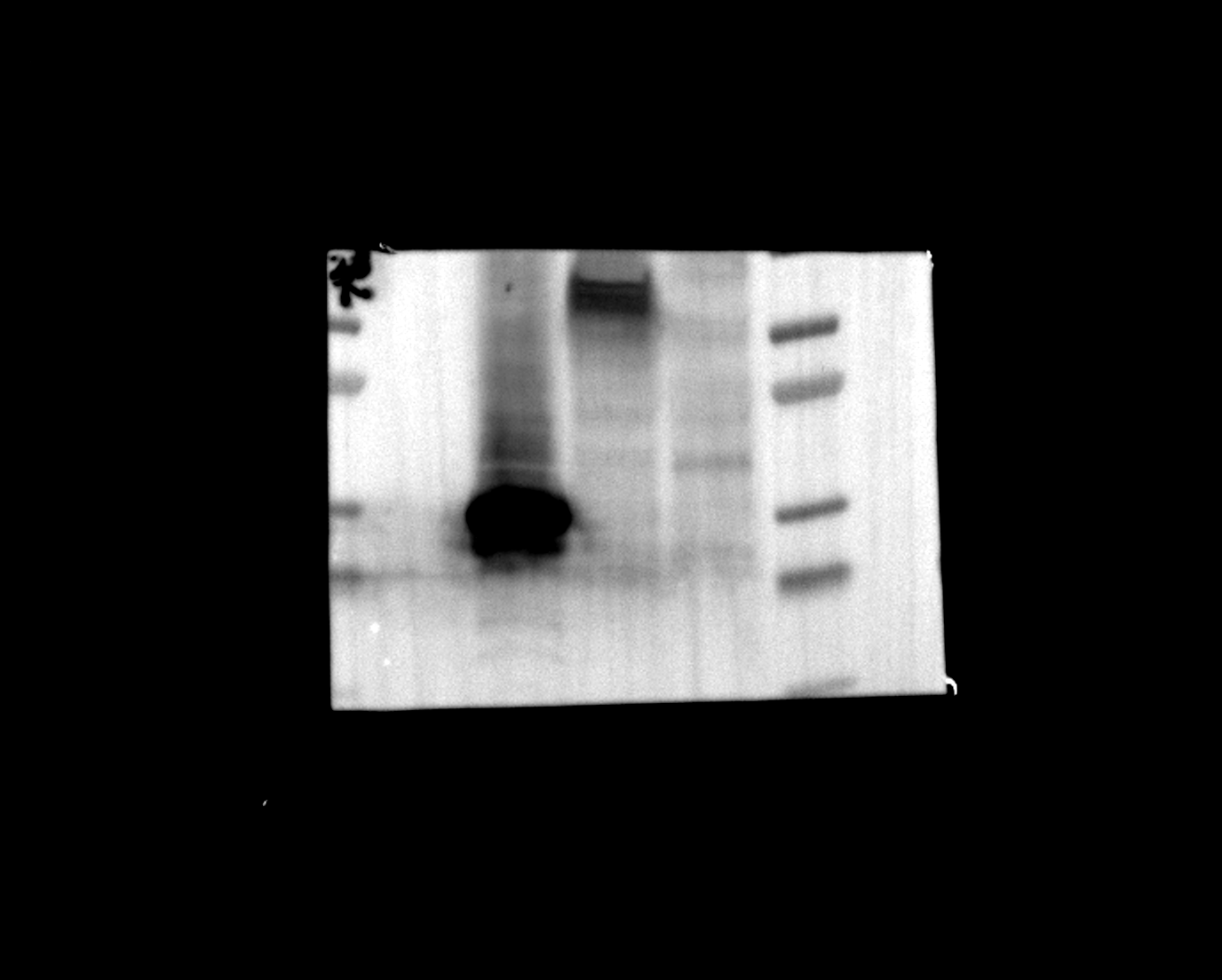

Supplement: Supplementary file 9 — Source data Fig. 5 [file 44318_2025_489_MOESM9_ESM.zip › Figure 5A/3. western Toll-6 and GFP.Tif]

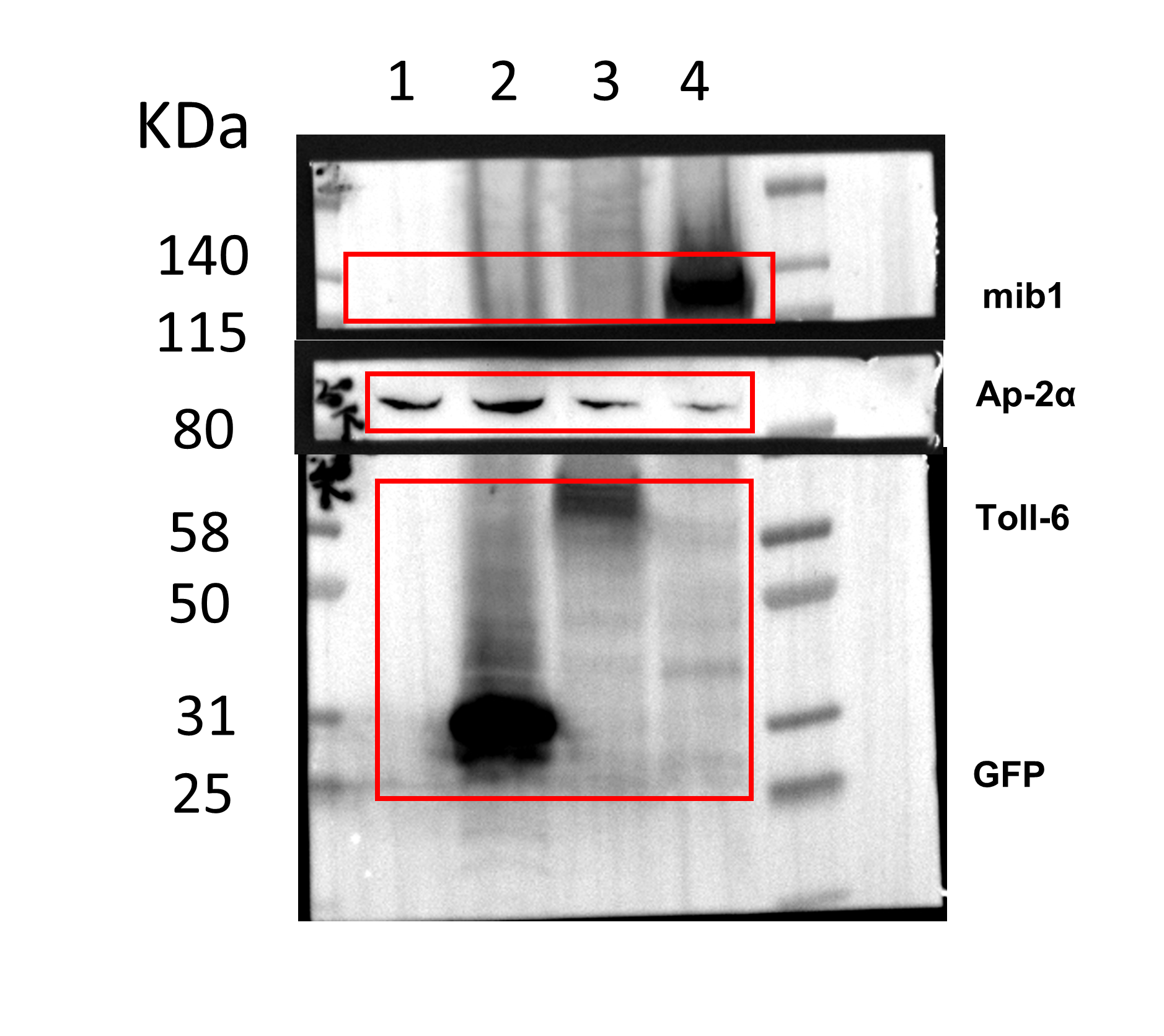

Supplement: Supplementary file 9 — Source data Fig. 5 [file 44318_2025_489_MOESM9_ESM.zip › Figure 5A/4. western whole.tif]

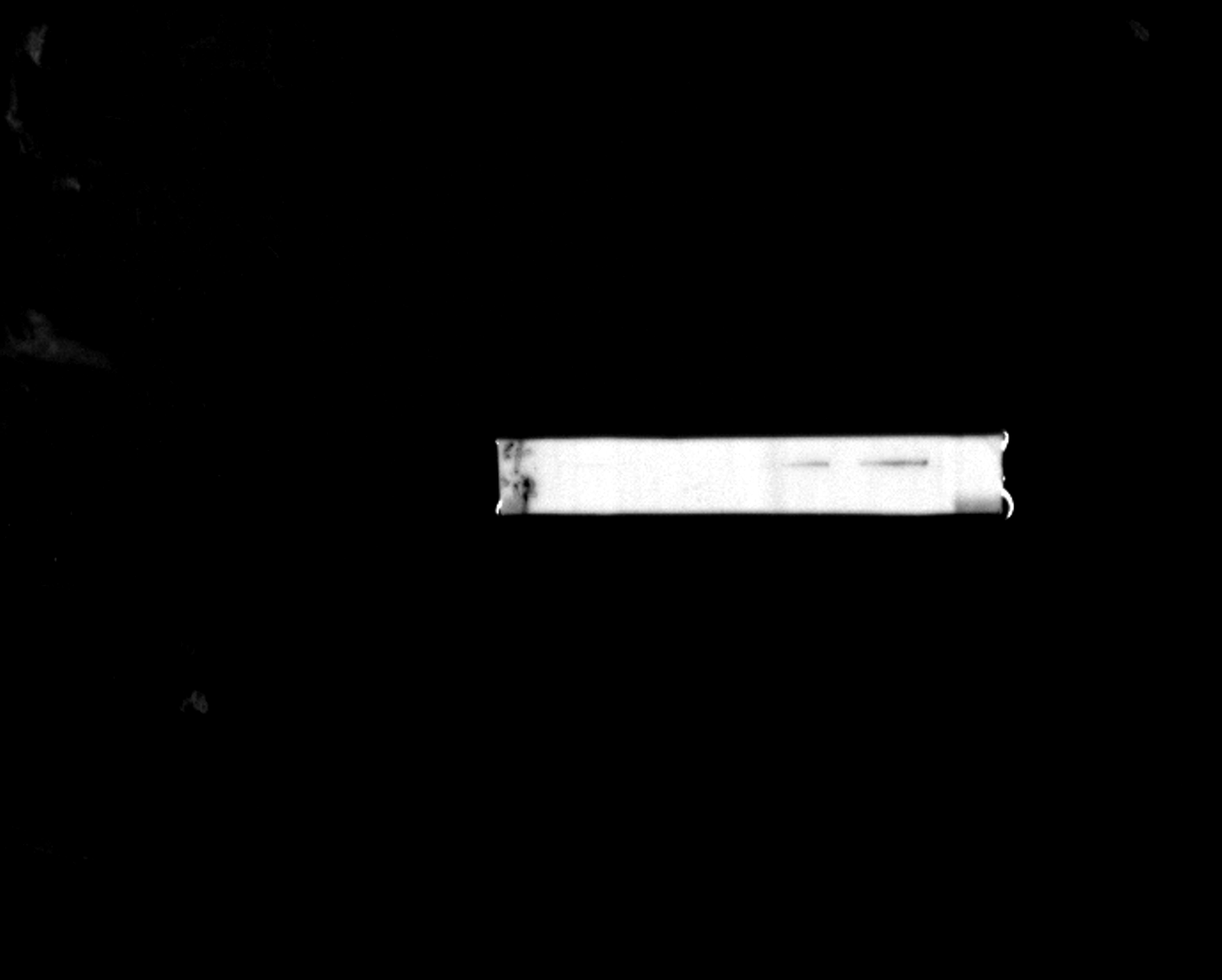

Supplement: Supplementary file 9 — Source data Fig. 5 [file 44318_2025_489_MOESM9_ESM.zip › Figure 5A/5. western Ap-2α .Tif]

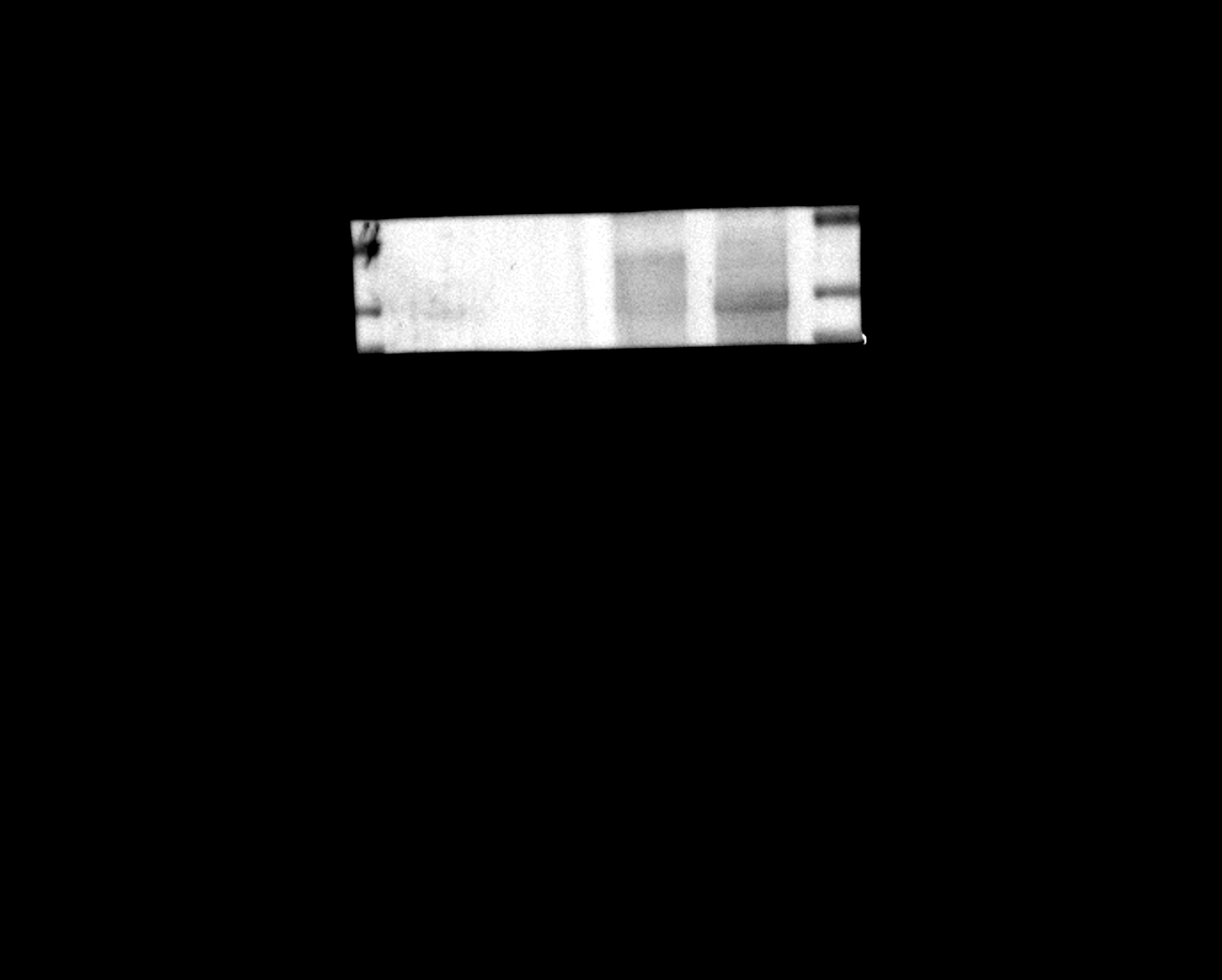

Supplement: Supplementary file 9 — Source data Fig. 5 [file 44318_2025_489_MOESM9_ESM.zip › Figure 5A/6.western mib1.Tif]

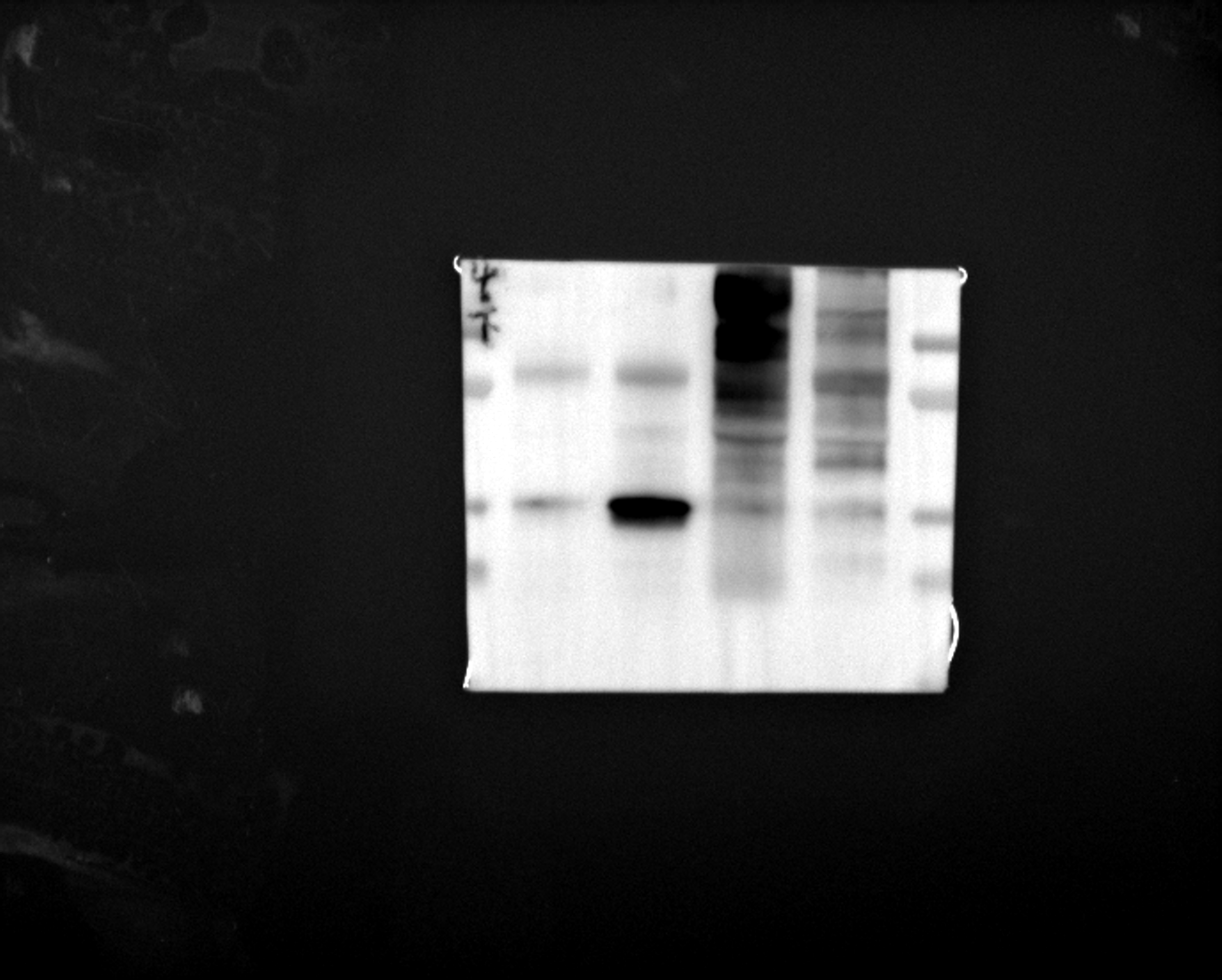

Supplement: Supplementary file 9 — Source data Fig. 5 [file 44318_2025_489_MOESM9_ESM.zip › Figure 5A/7. western Toll-6 and GFP.Tif]

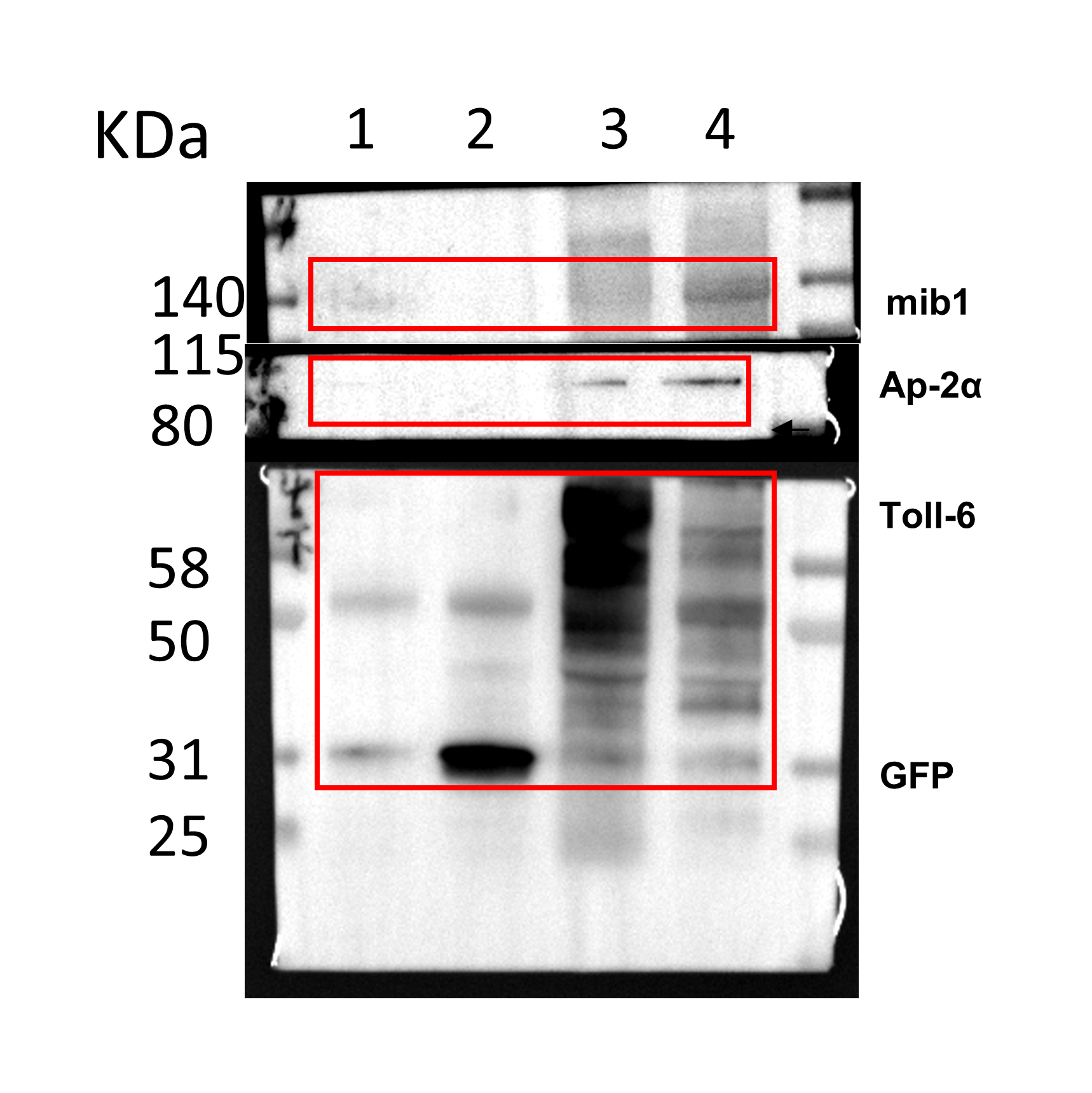

Supplement: Supplementary file 9 — Source data Fig. 5 [file 44318_2025_489_MOESM9_ESM.zip › Figure 5A/8. western whole.tif]

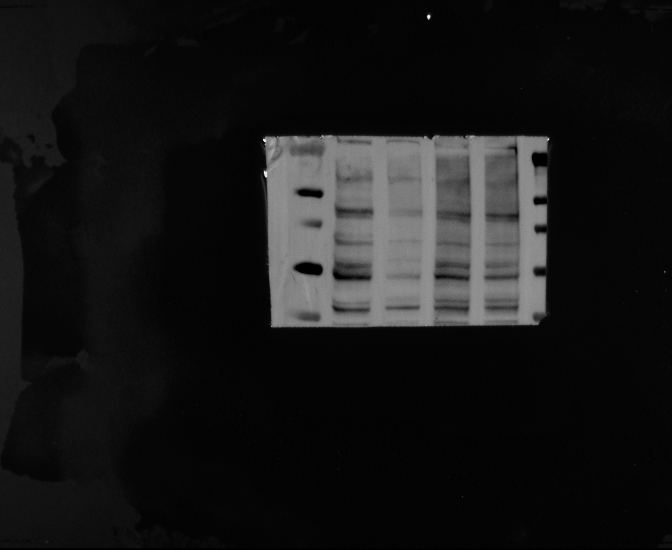

Supplement: Supplementary file 9 — Source data Fig. 5 [file 44318_2025_489_MOESM9_ESM.zip › Figure 5B/1. western myc-Ub.tif]

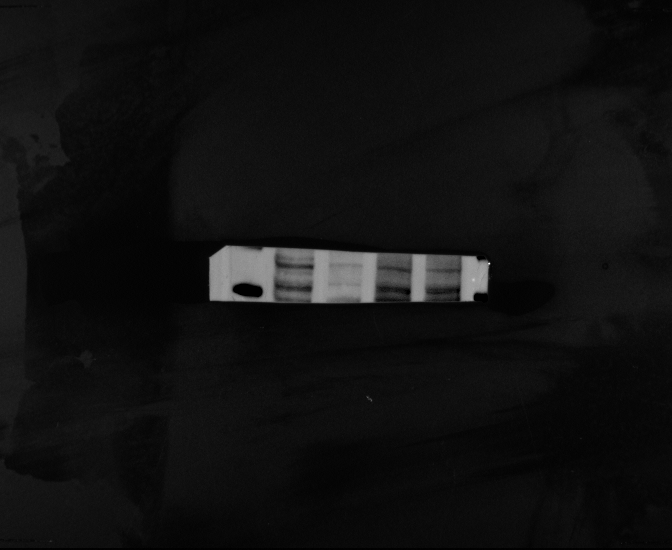

Supplement: Supplementary file 9 — Source data Fig. 5 [file 44318_2025_489_MOESM9_ESM.zip › Figure 5B/2. western Ap-2α .tif]

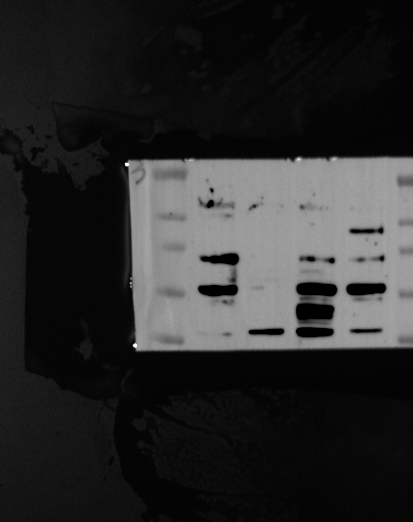

Supplement: Supplementary file 9 — Source data Fig. 5 [file 44318_2025_489_MOESM9_ESM.zip › Figure 5B/3 and 4. western mib1 and toll6.tif]

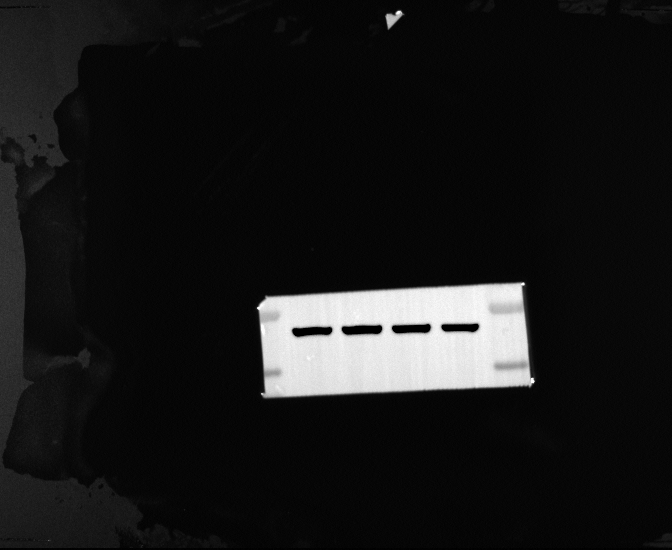

Supplement: Supplementary file 9 — Source data Fig. 5 [file 44318_2025_489_MOESM9_ESM.zip › Figure 5B/5.western actin.tif]

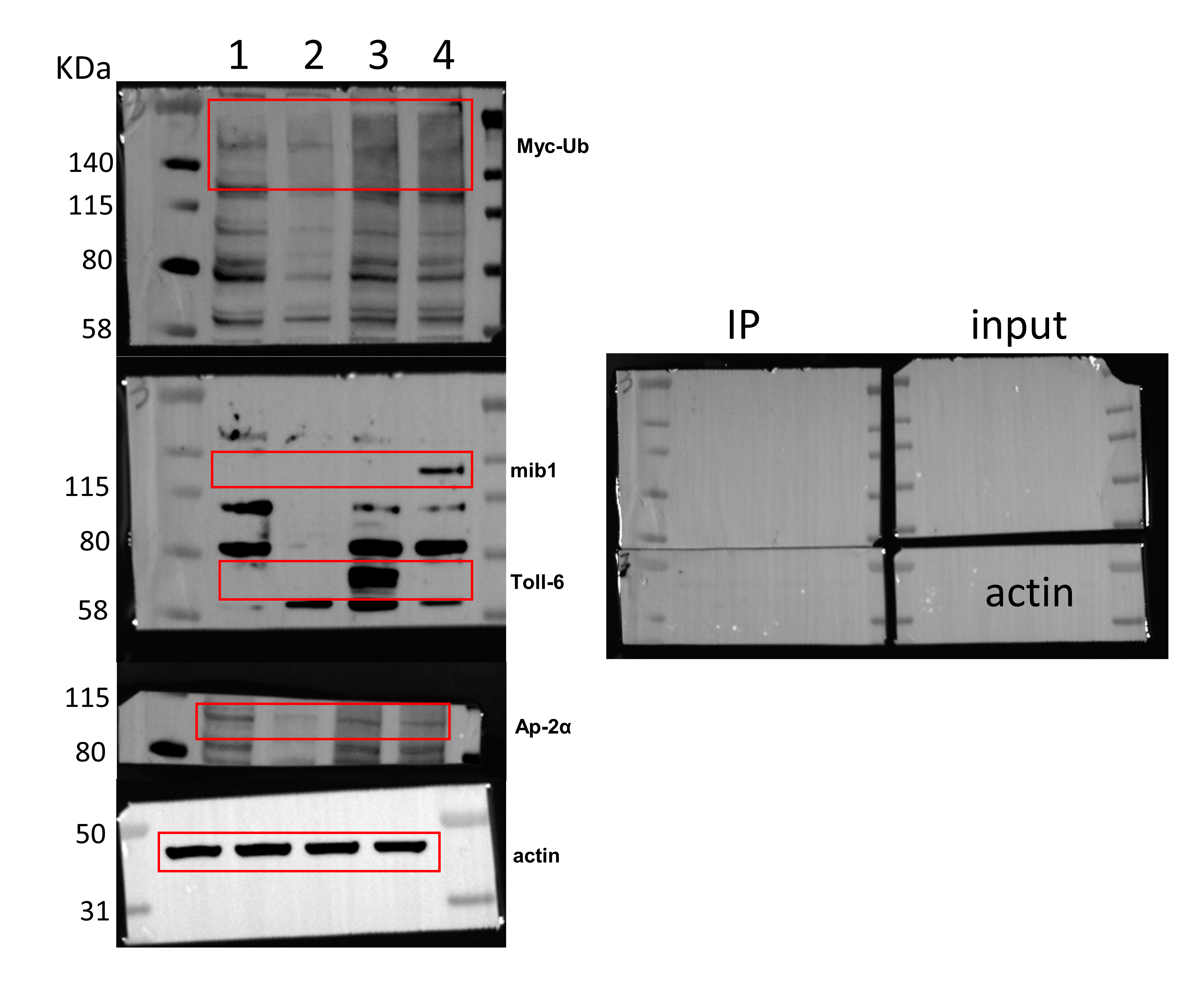

Supplement: Supplementary file 9 — Source data Fig. 5 [file 44318_2025_489_MOESM9_ESM.zip › Figure 5B/6. western whole.TIF]

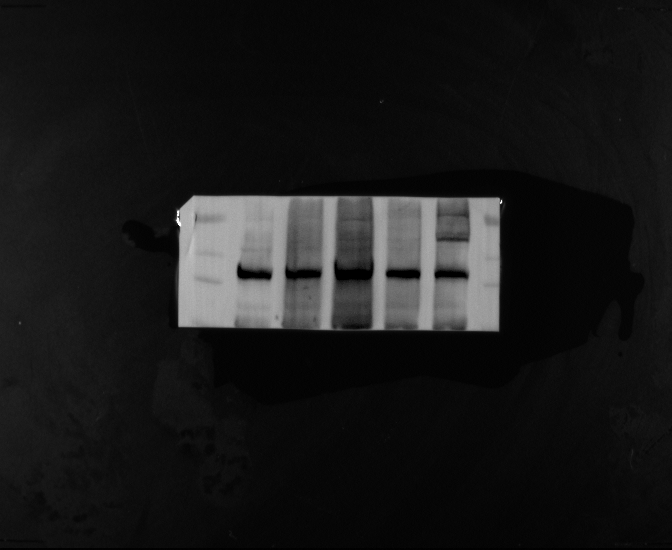

Supplement: Supplementary file 9 — Source data Fig. 5 [file 44318_2025_489_MOESM9_ESM.zip › Figure 5C/1.western myc-Ub.tif]

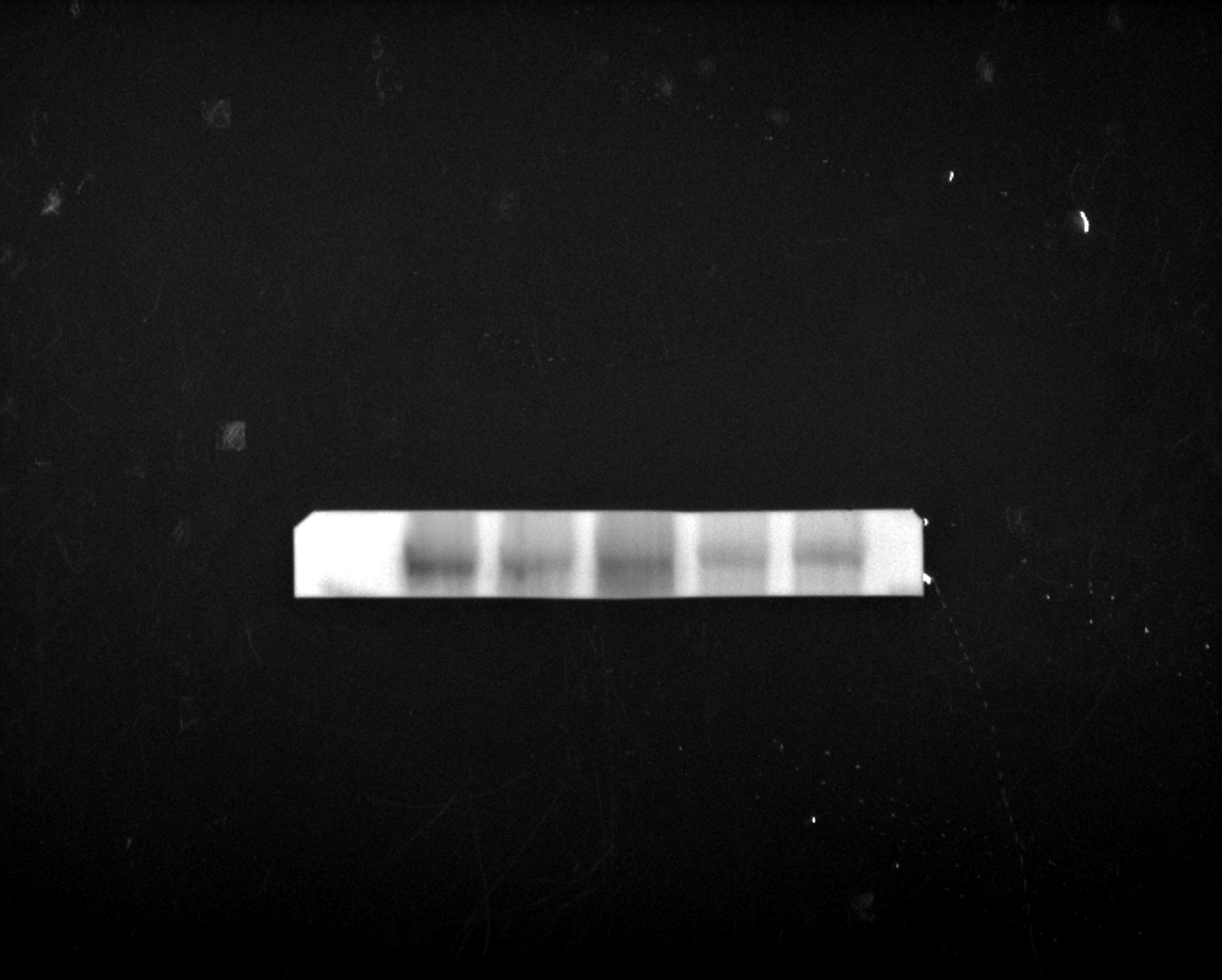

Supplement: Supplementary file 9 — Source data Fig. 5 [file 44318_2025_489_MOESM9_ESM.zip › Figure 5C/2.western Ap-2α.Tif]

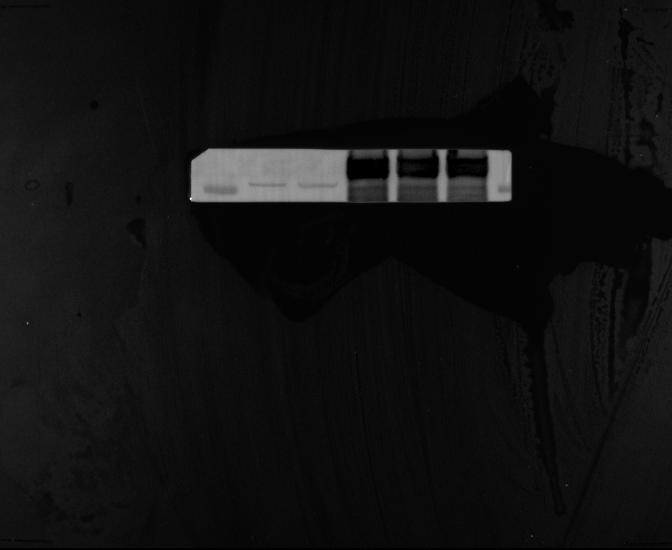

Supplement: Supplementary file 9 — Source data Fig. 5 [file 44318_2025_489_MOESM9_ESM.zip › Figure 5C/3.western Toll-6.tif]

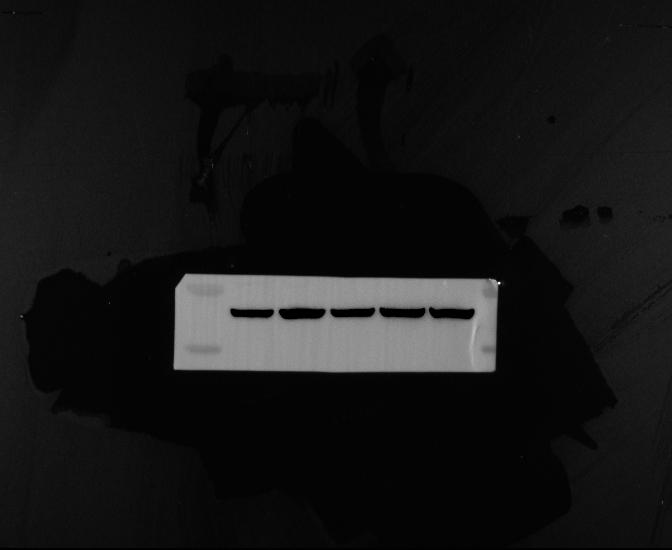

Supplement: Supplementary file 9 — Source data Fig. 5 [file 44318_2025_489_MOESM9_ESM.zip › Figure 5C/4. wesstern actin.tif]

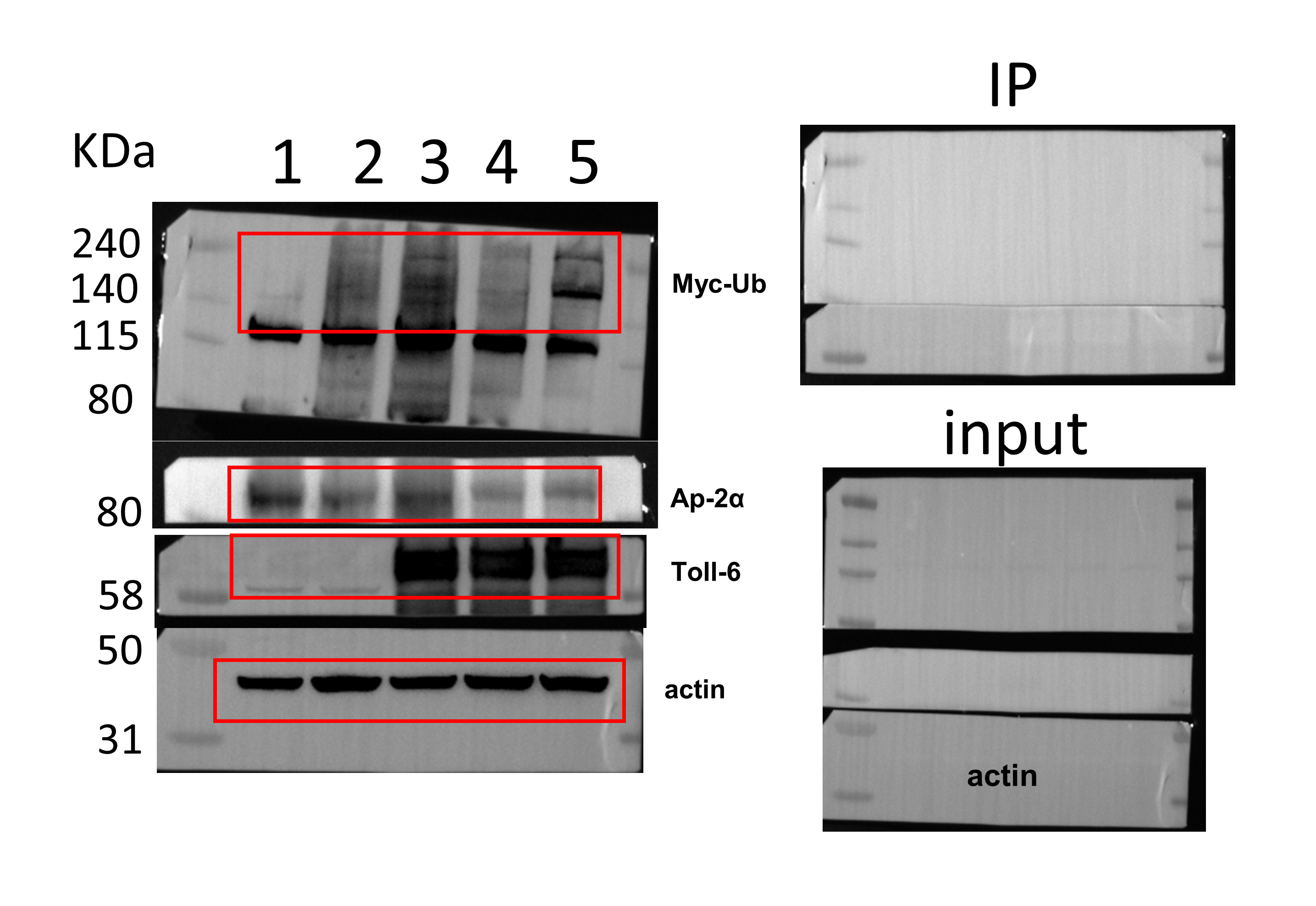

Supplement: Supplementary file 9 — Source data Fig. 5 [file 44318_2025_489_MOESM9_ESM.zip › Figure 5C/5. whole.TIF]

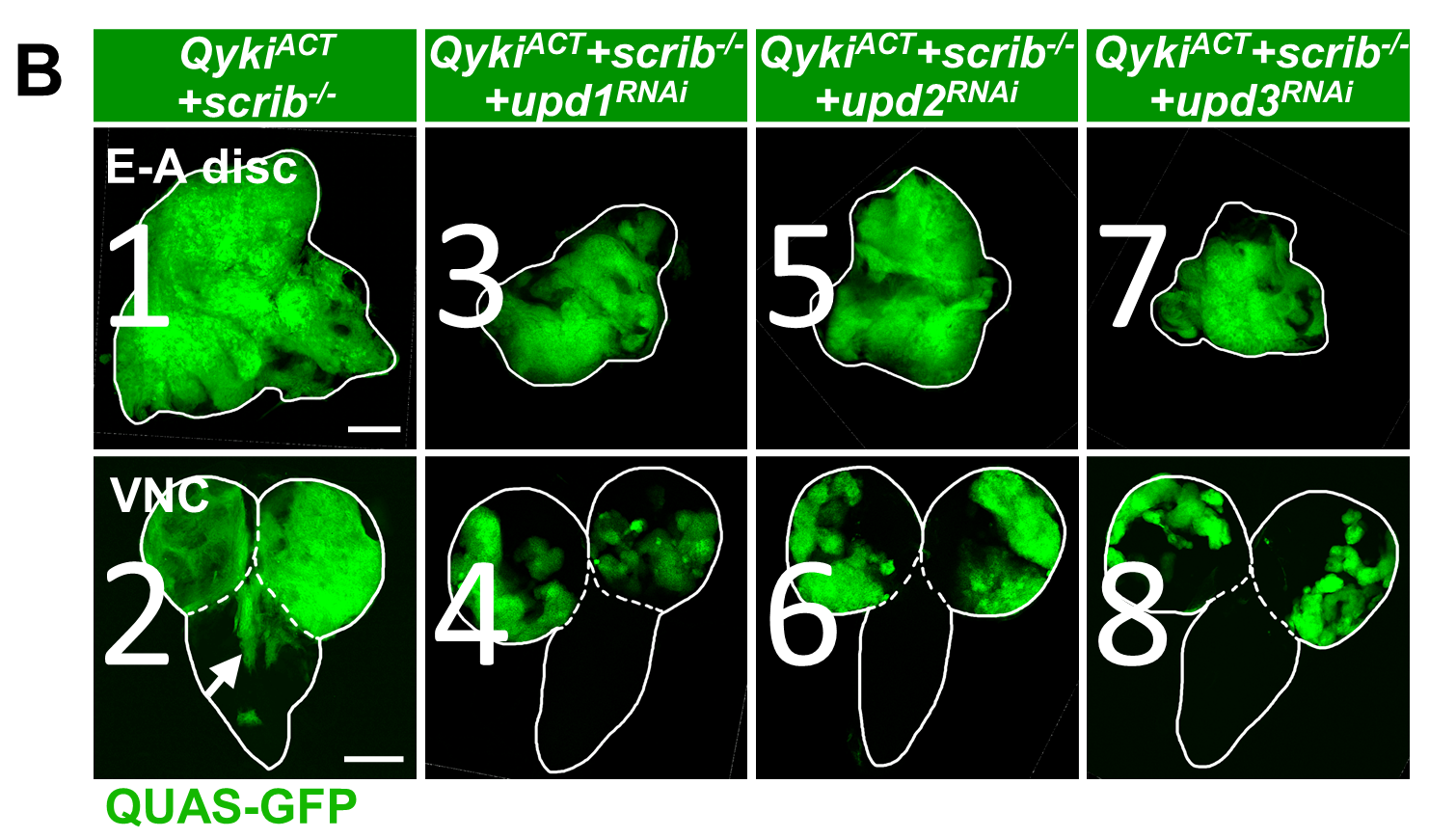

Supplement: Supplementary file 10 — Source data Fig. 6 [file 44318_2025_489_MOESM10_ESM.zip › Figure 6B/0 paper Figure 6B with provided image sequence.tif]

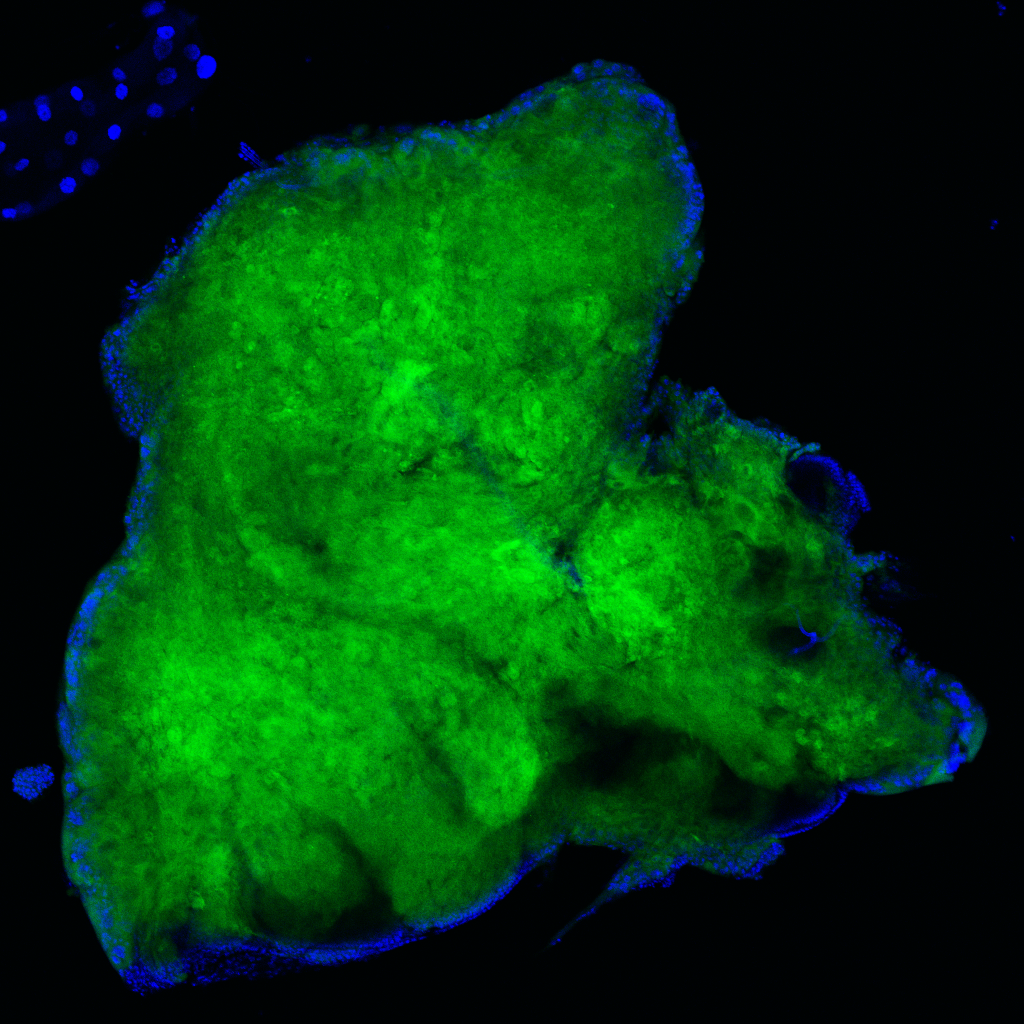

Supplement: Supplementary file 10 — Source data Fig. 6 [file 44318_2025_489_MOESM10_ESM.zip › Figure 6B/1 original image.tif]

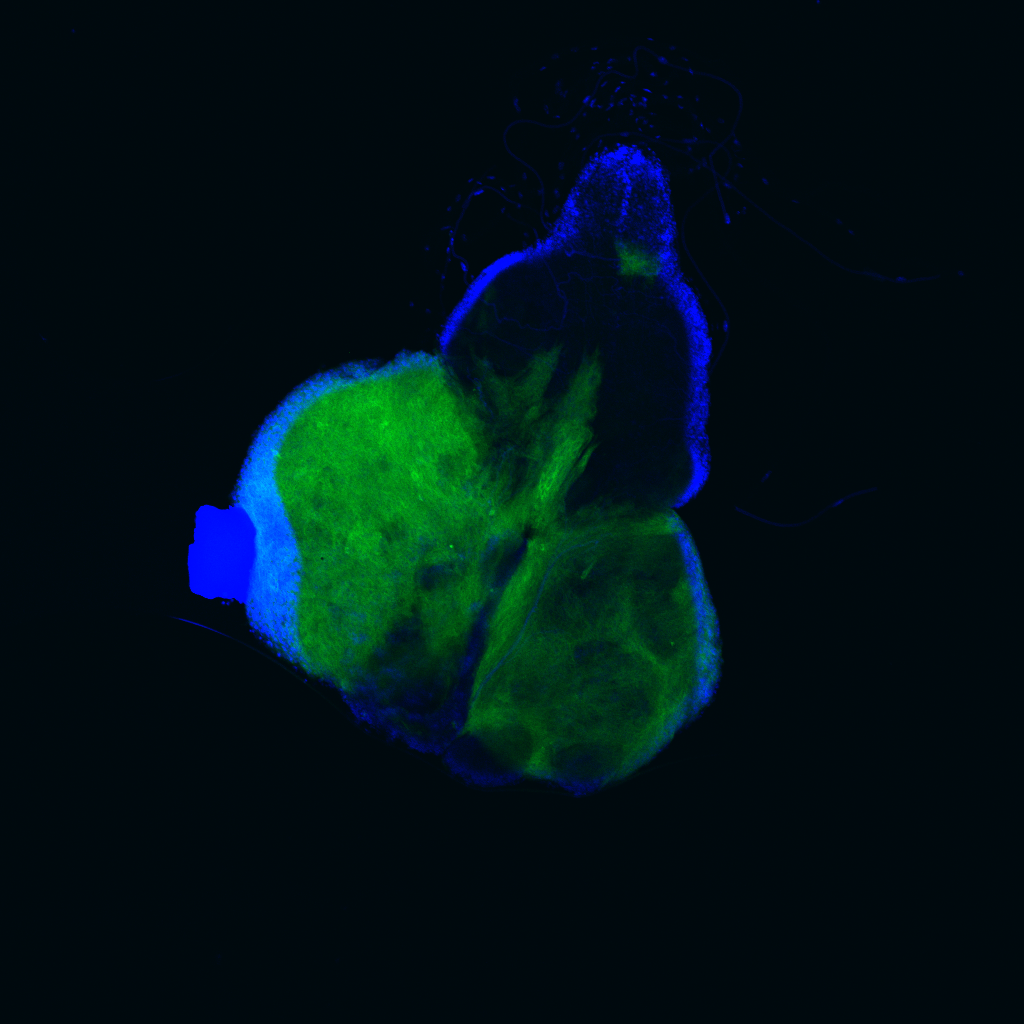

Supplement: Supplementary file 10 — Source data Fig. 6 [file 44318_2025_489_MOESM10_ESM.zip › Figure 6B/2 original image.tif]

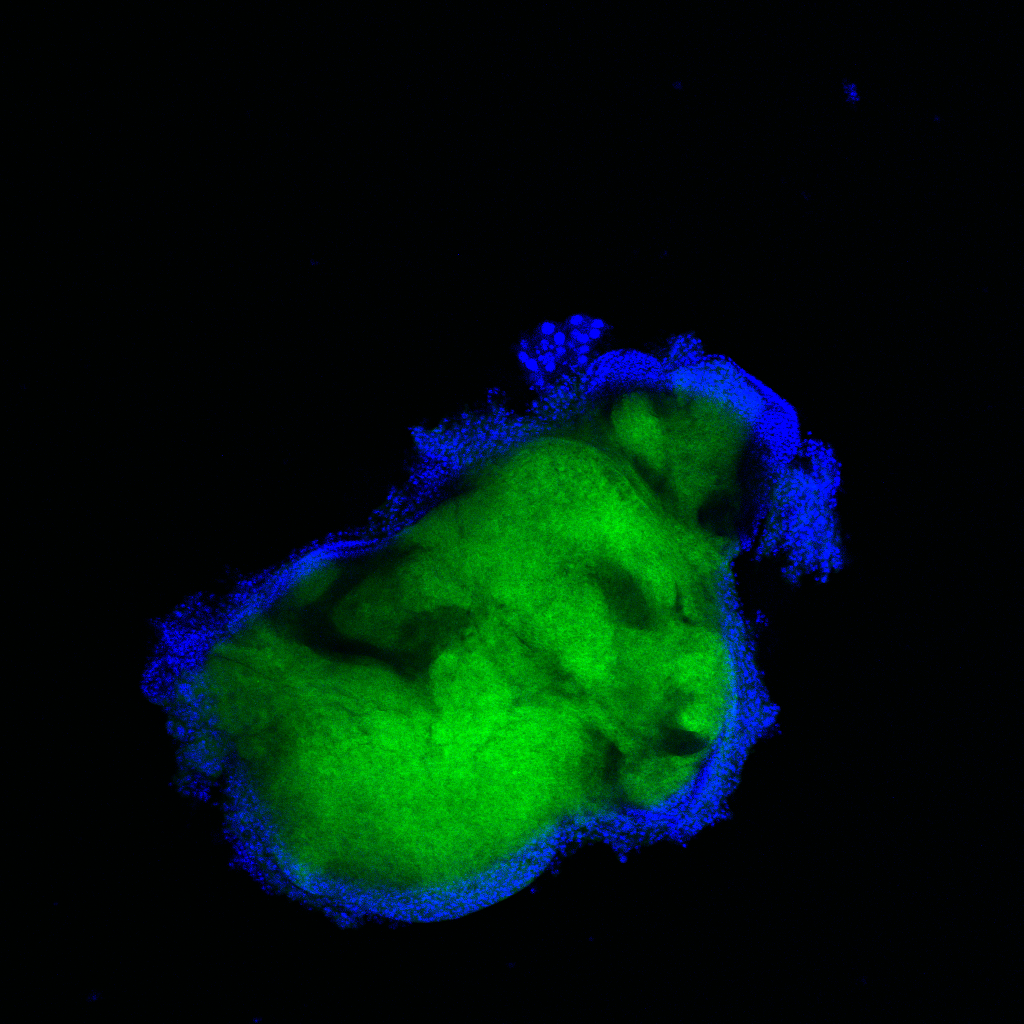

Supplement: Supplementary file 10 — Source data Fig. 6 [file 44318_2025_489_MOESM10_ESM.zip › Figure 6B/3 original image.tif]

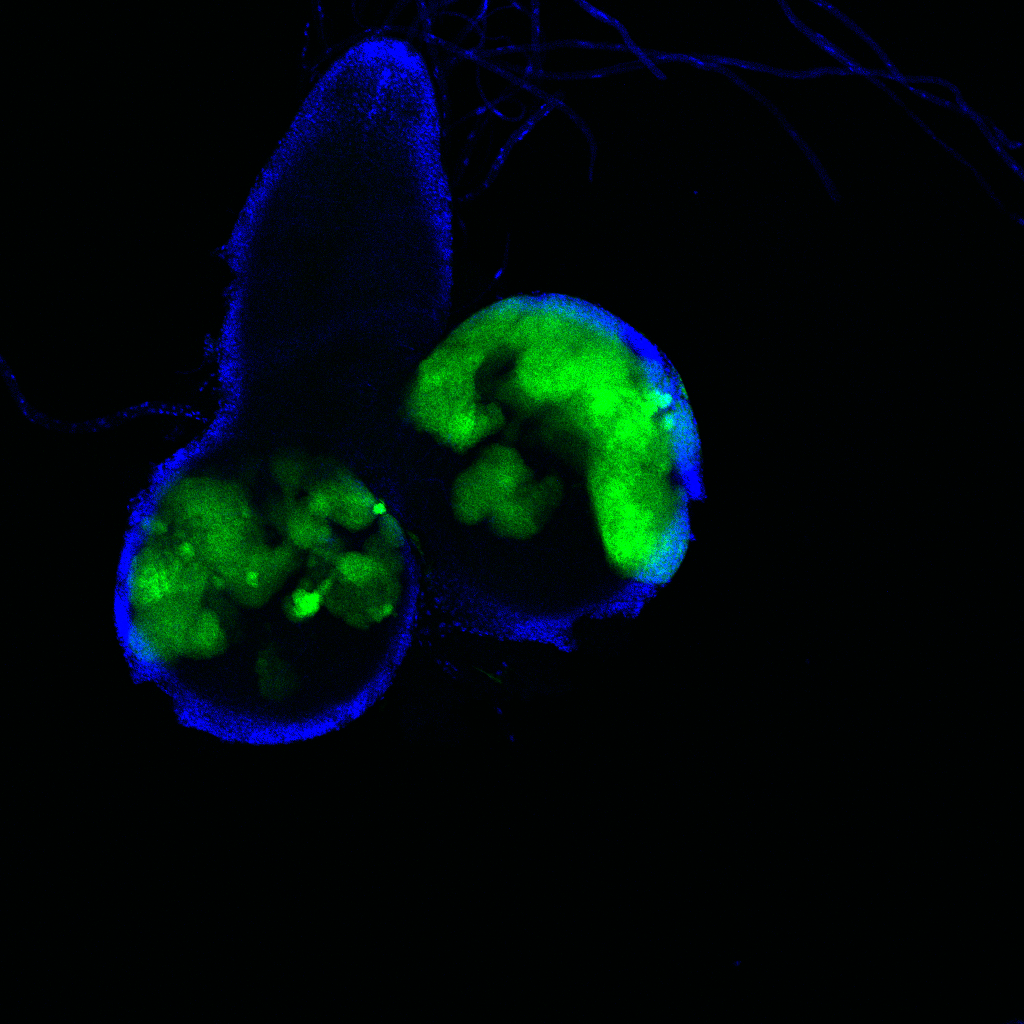

Supplement: Supplementary file 10 — Source data Fig. 6 [file 44318_2025_489_MOESM10_ESM.zip › Figure 6B/4 original image.tif]

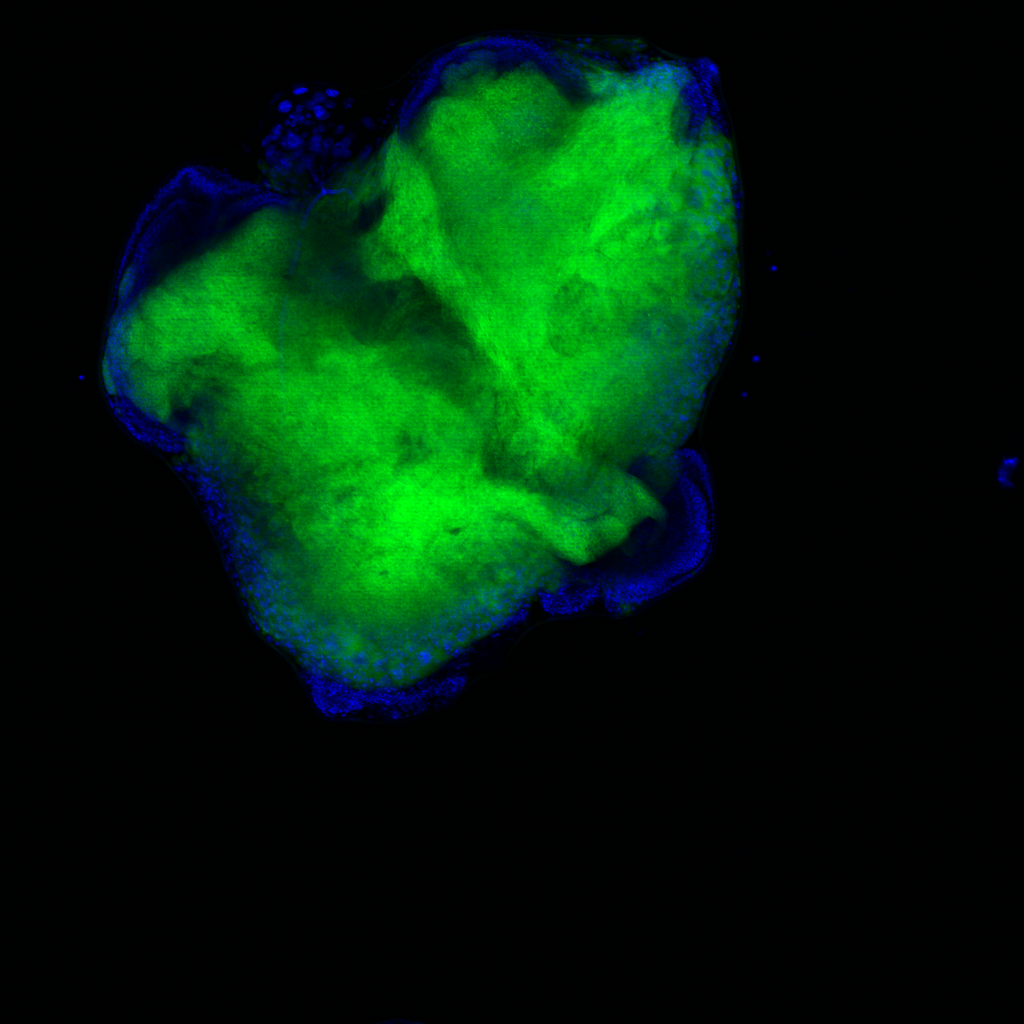

Supplement: Supplementary file 10 — Source data Fig. 6 [file 44318_2025_489_MOESM10_ESM.zip › Figure 6B/5 original image.tif]

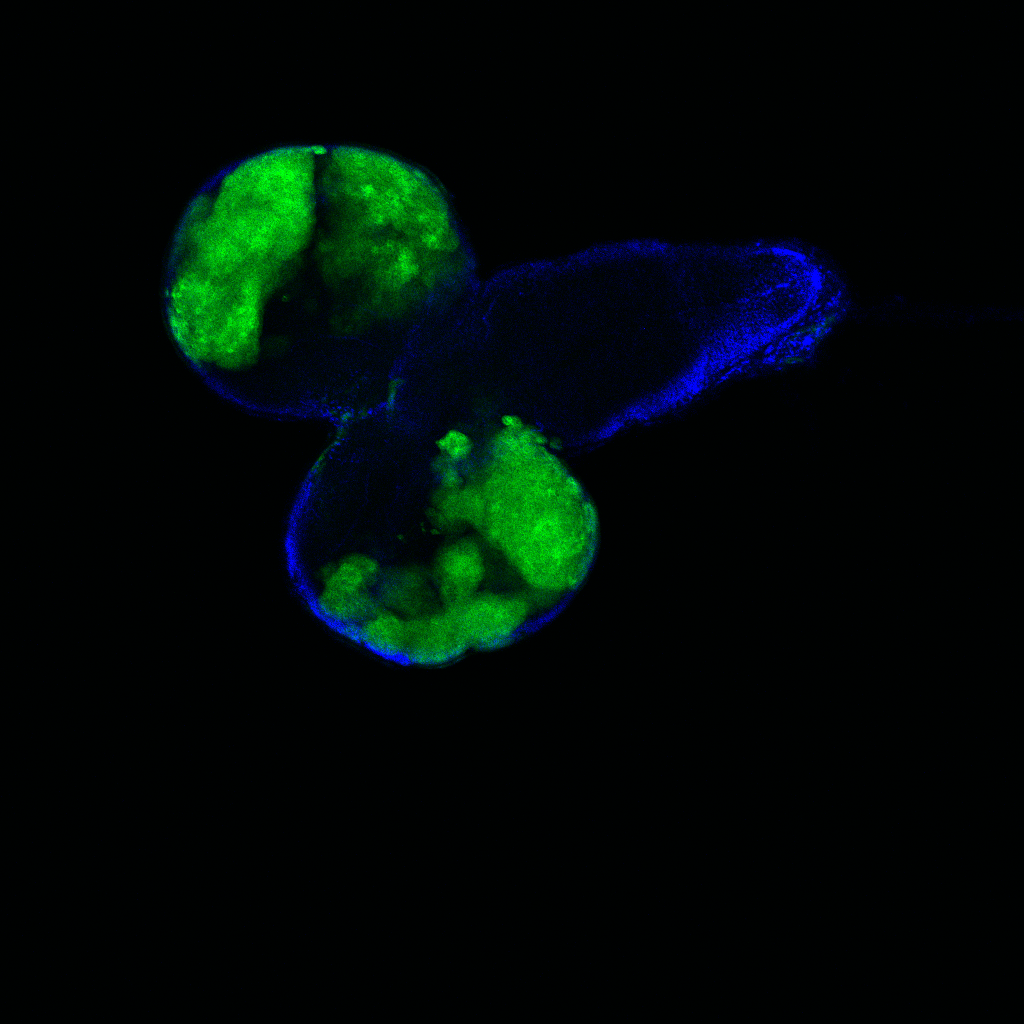

Supplement: Supplementary file 10 — Source data Fig. 6 [file 44318_2025_489_MOESM10_ESM.zip › Figure 6B/6 original image.tif]

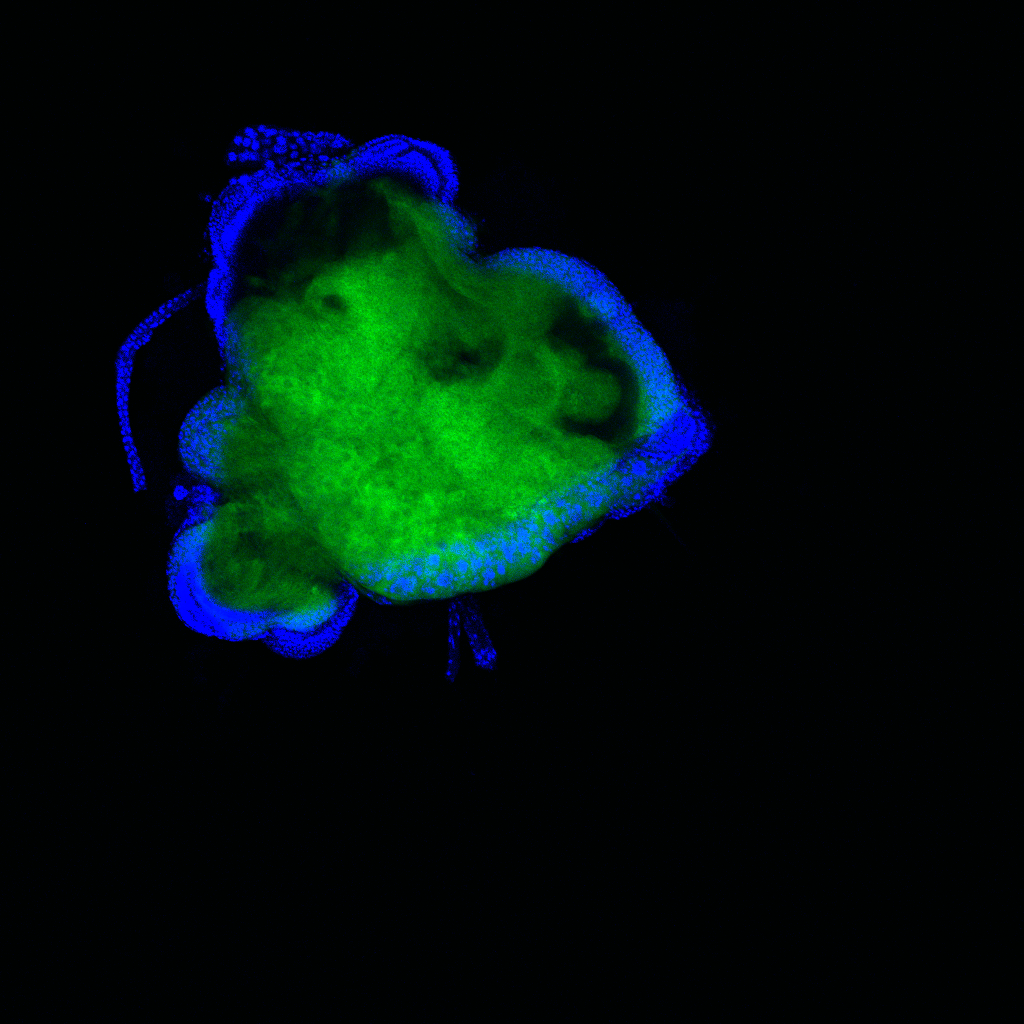

Supplement: Supplementary file 10 — Source data Fig. 6 [file 44318_2025_489_MOESM10_ESM.zip › Figure 6B/7 original image.tif]

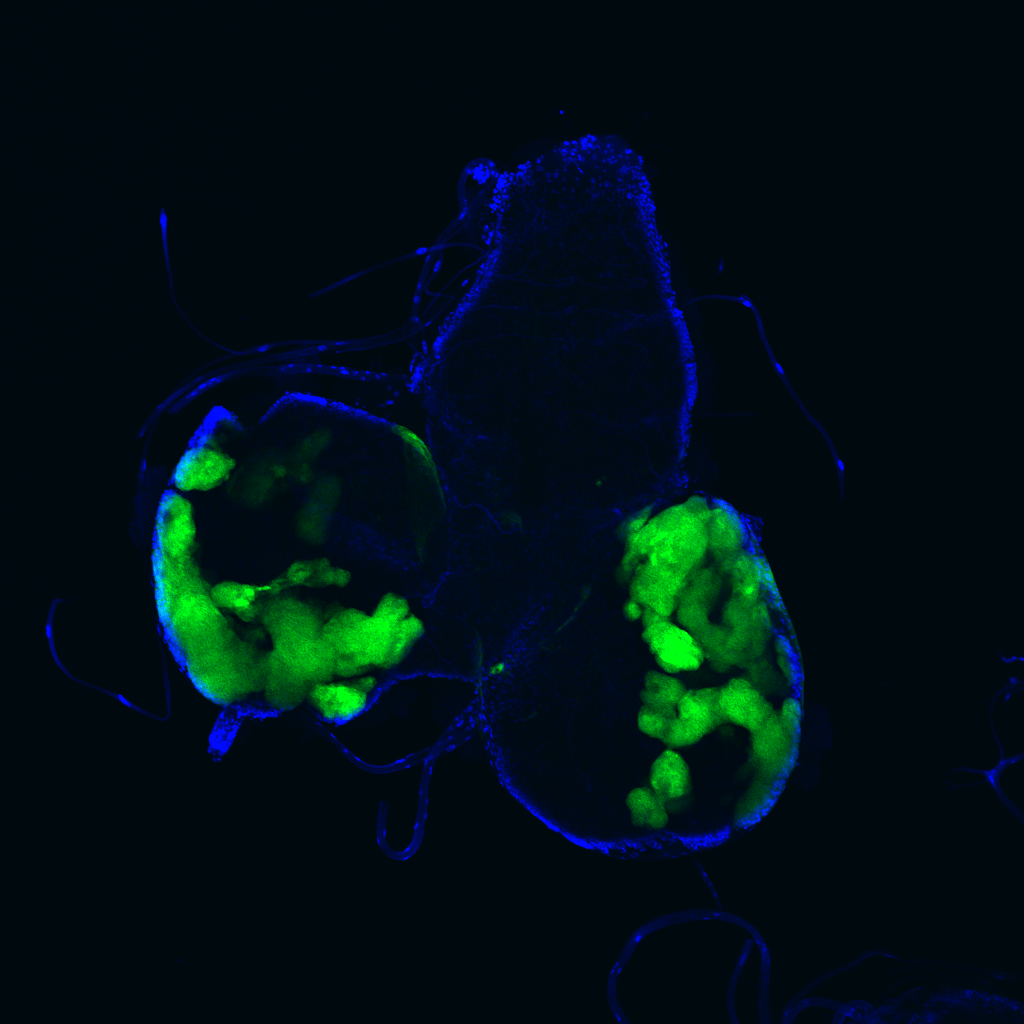

Supplement: Supplementary file 10 — Source data Fig. 6 [file 44318_2025_489_MOESM10_ESM.zip › Figure 6B/8 original image.tif]

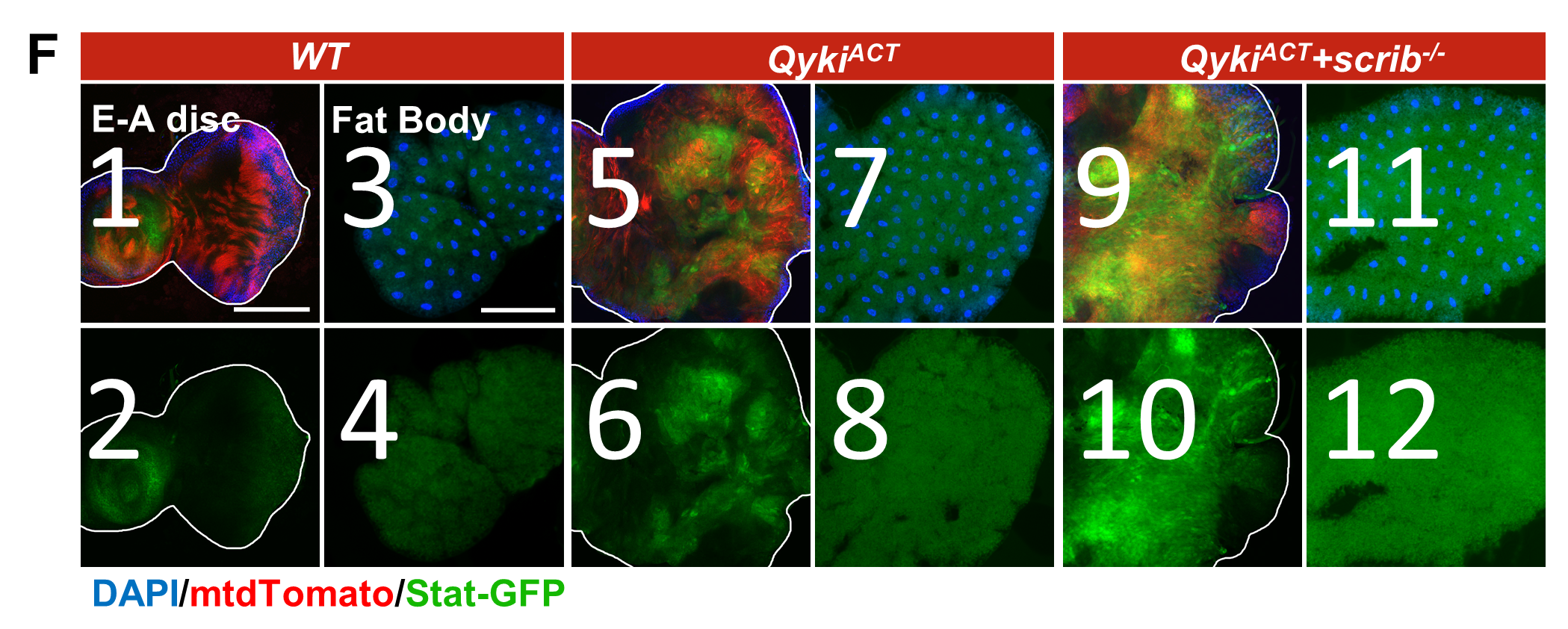

Supplement: Supplementary file 10 — Source data Fig. 6 [file 44318_2025_489_MOESM10_ESM.zip › Figure 6F/0 paper Figure 6F with provided image sequence.tif]

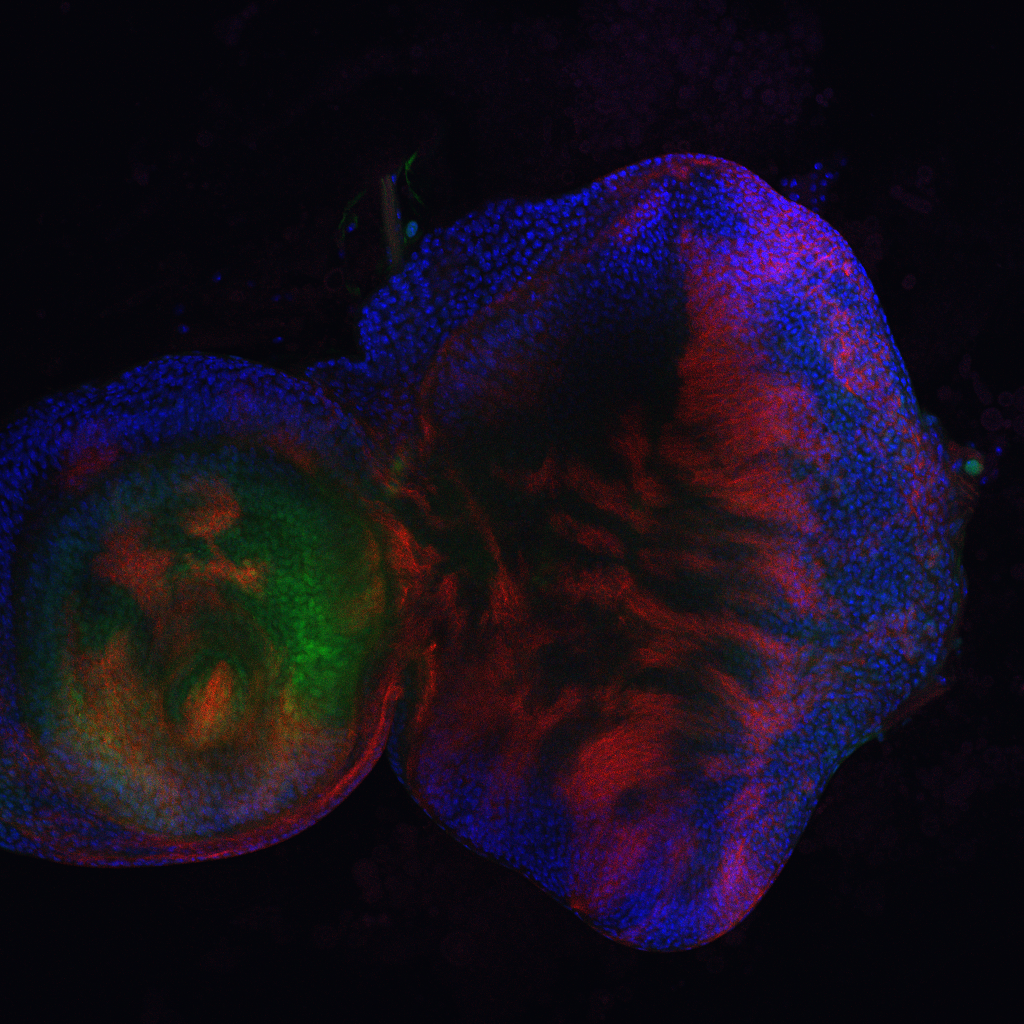

Supplement: Supplementary file 10 — Source data Fig. 6 [file 44318_2025_489_MOESM10_ESM.zip › Figure 6F/1 original image.tif]

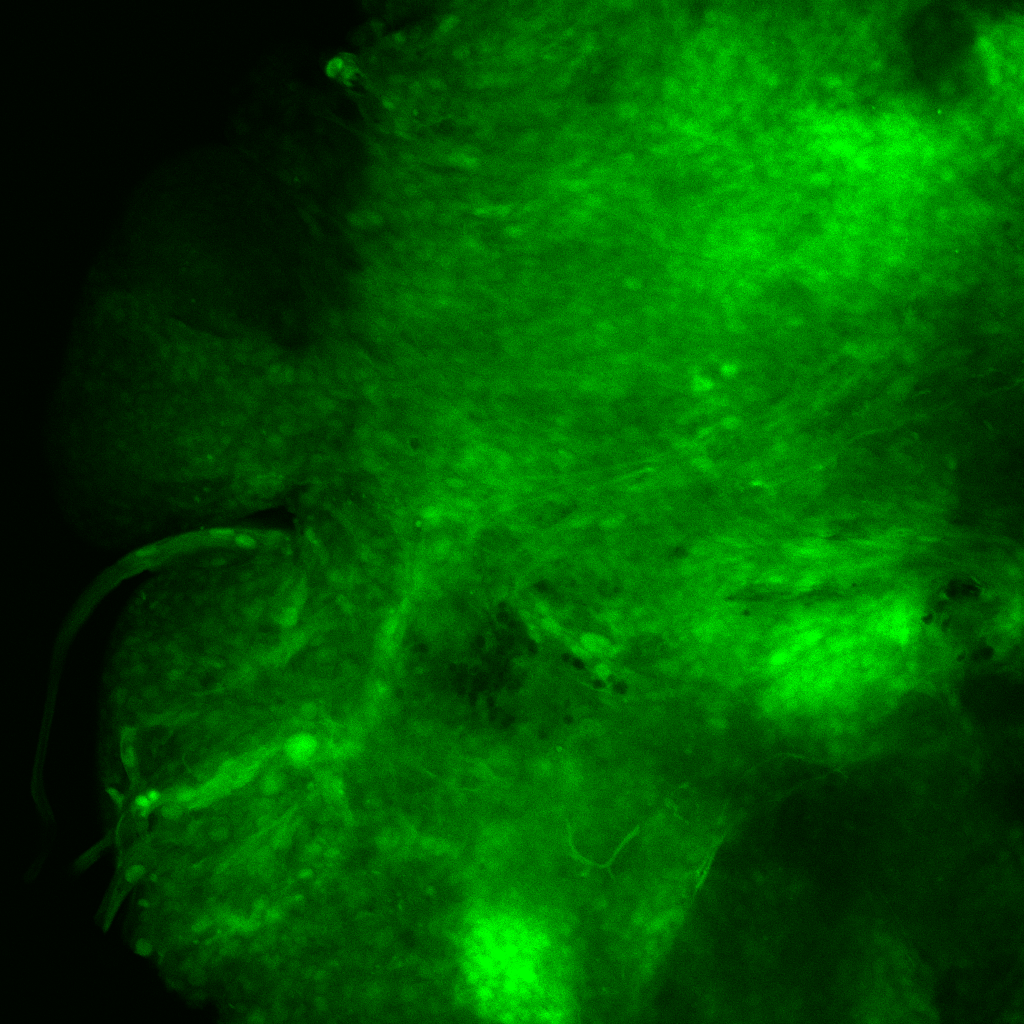

Supplement: Supplementary file 10 — Source data Fig. 6 [file 44318_2025_489_MOESM10_ESM.zip › Figure 6F/10 original image.tif]

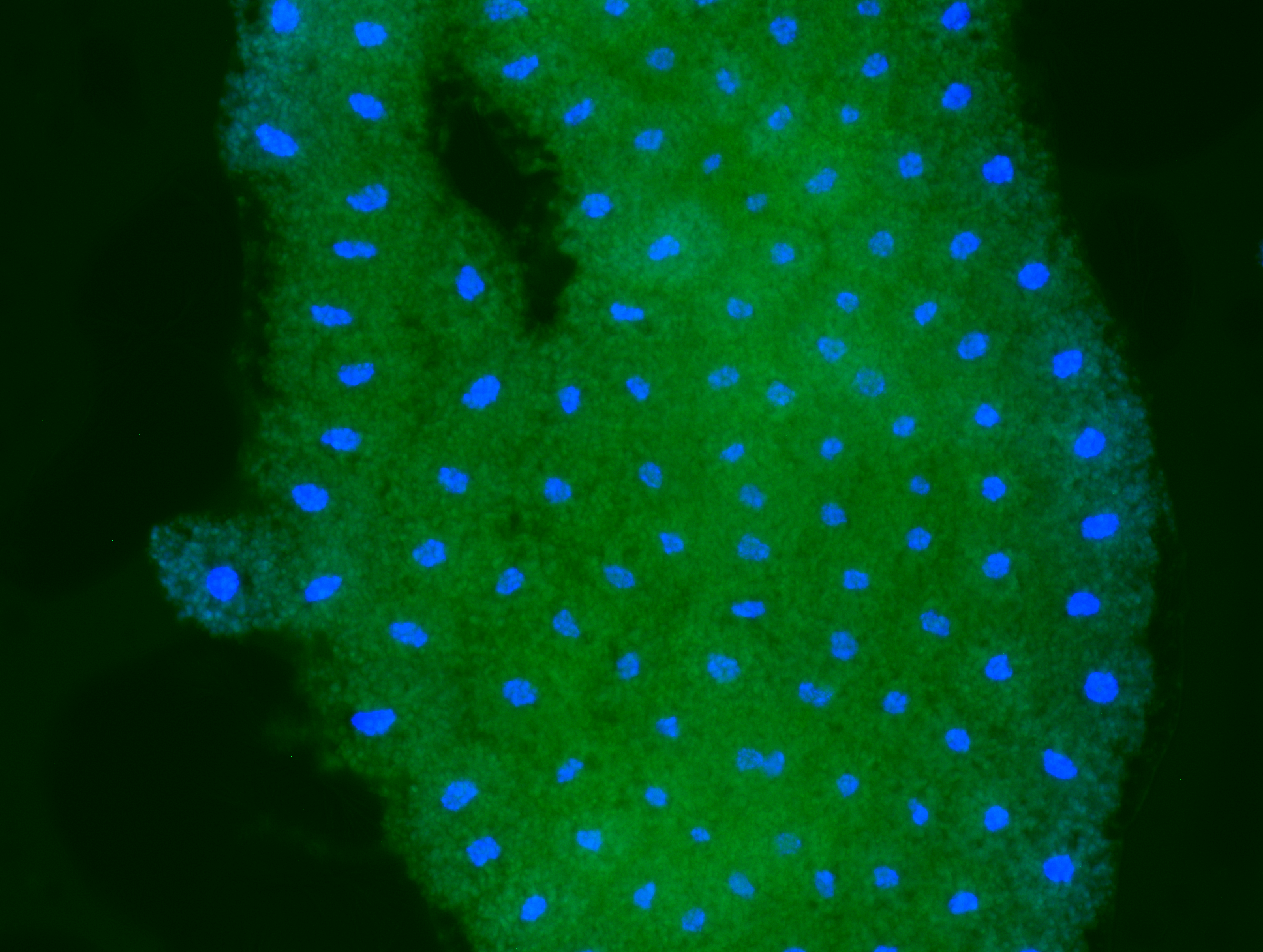

Supplement: Supplementary file 10 — Source data Fig. 6 [file 44318_2025_489_MOESM10_ESM.zip › Figure 6F/11 original image.tif]

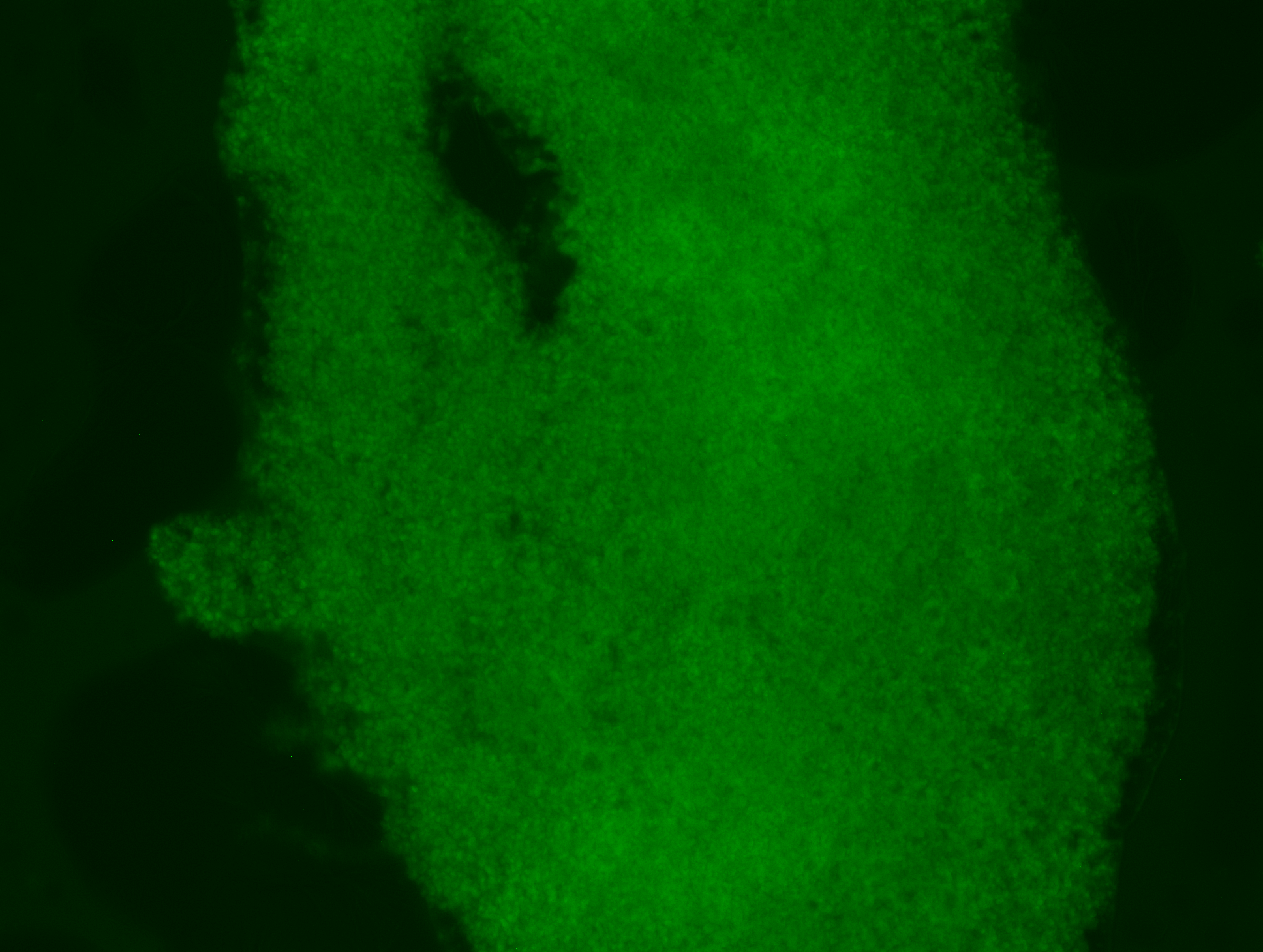

Supplement: Supplementary file 10 — Source data Fig. 6 [file 44318_2025_489_MOESM10_ESM.zip › Figure 6F/12 original image.tif]

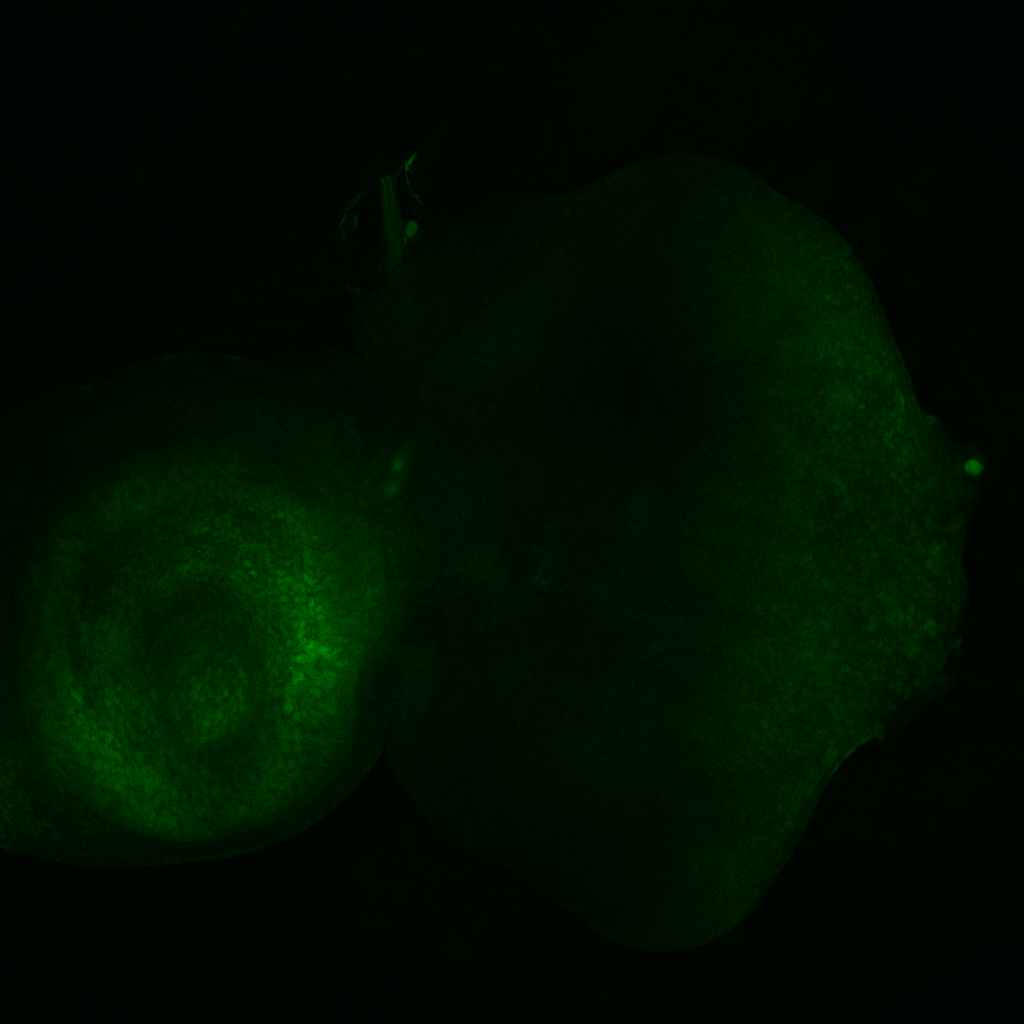

Supplement: Supplementary file 10 — Source data Fig. 6 [file 44318_2025_489_MOESM10_ESM.zip › Figure 6F/2 original image.tif]

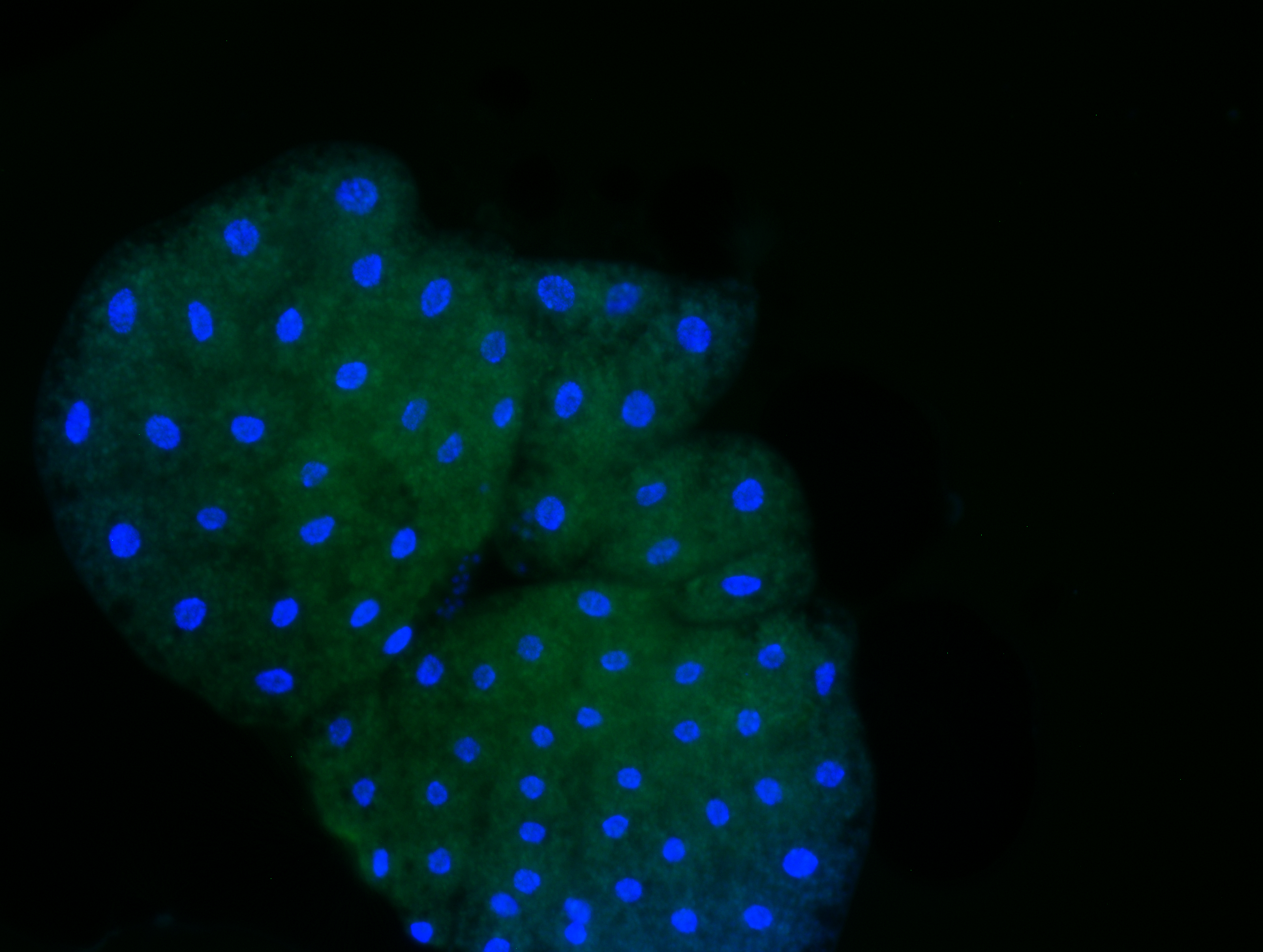

Supplement: Supplementary file 10 — Source data Fig. 6 [file 44318_2025_489_MOESM10_ESM.zip › Figure 6F/3 original image.tif]

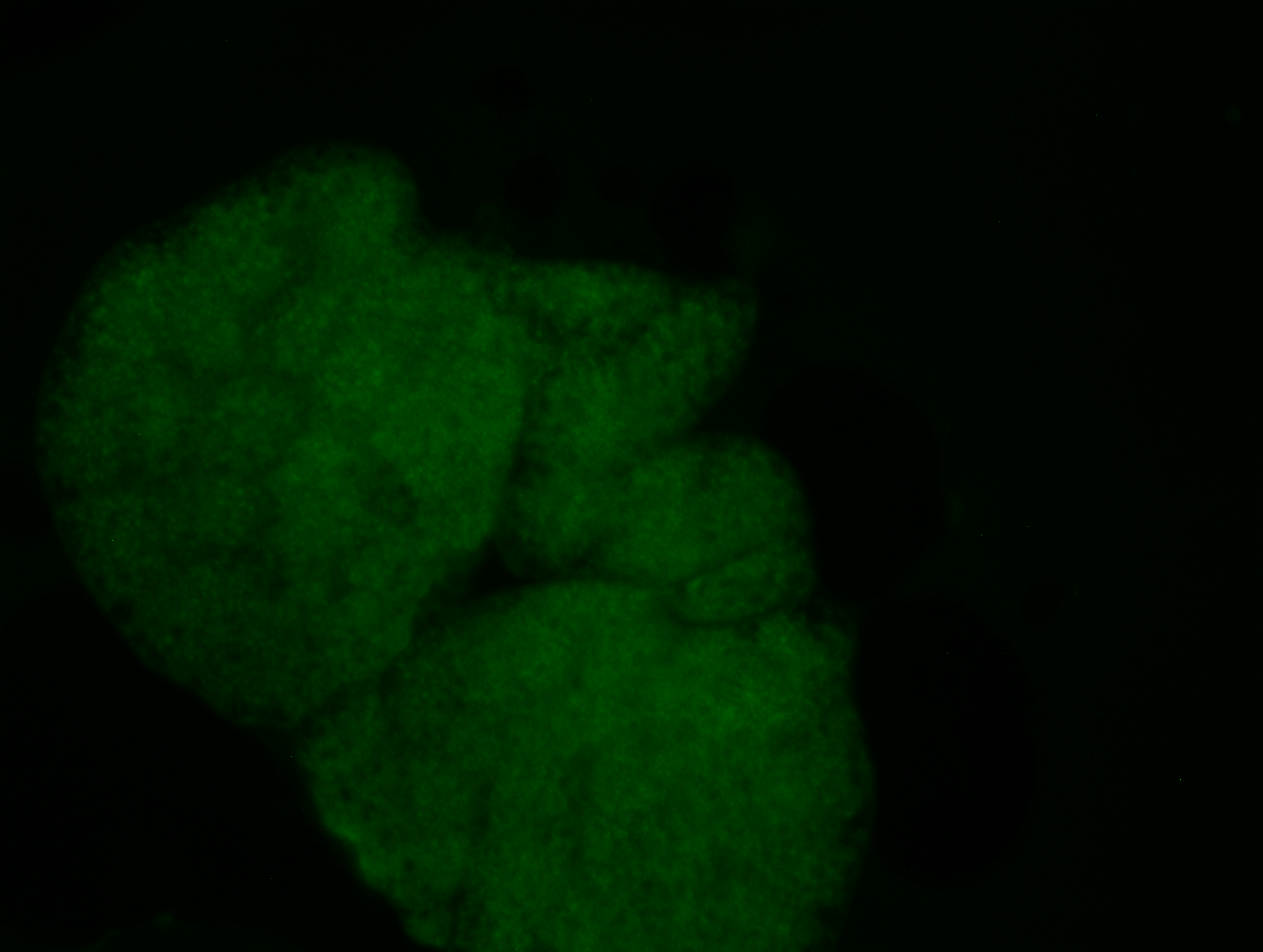

Supplement: Supplementary file 10 — Source data Fig. 6 [file 44318_2025_489_MOESM10_ESM.zip › Figure 6F/4 original image.tif]

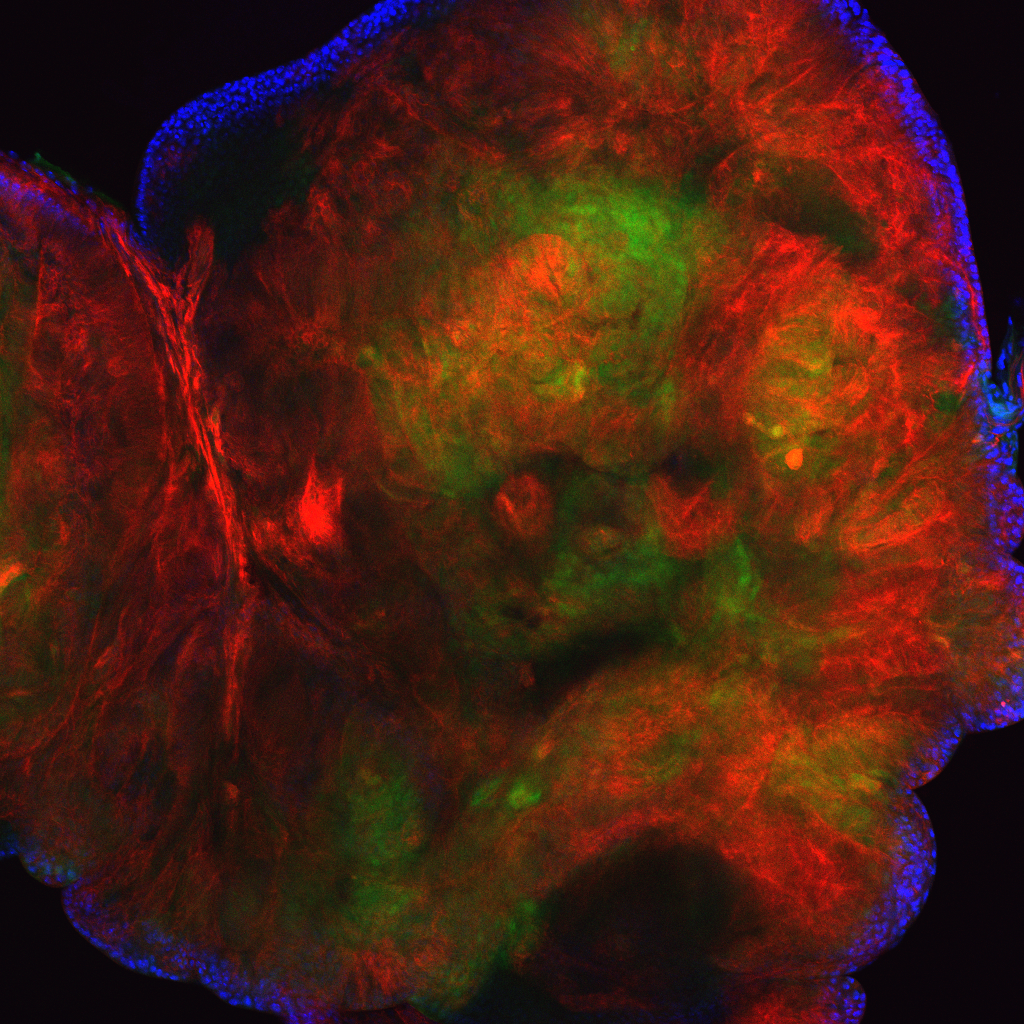

Supplement: Supplementary file 10 — Source data Fig. 6 [file 44318_2025_489_MOESM10_ESM.zip › Figure 6F/5 original image.tif]

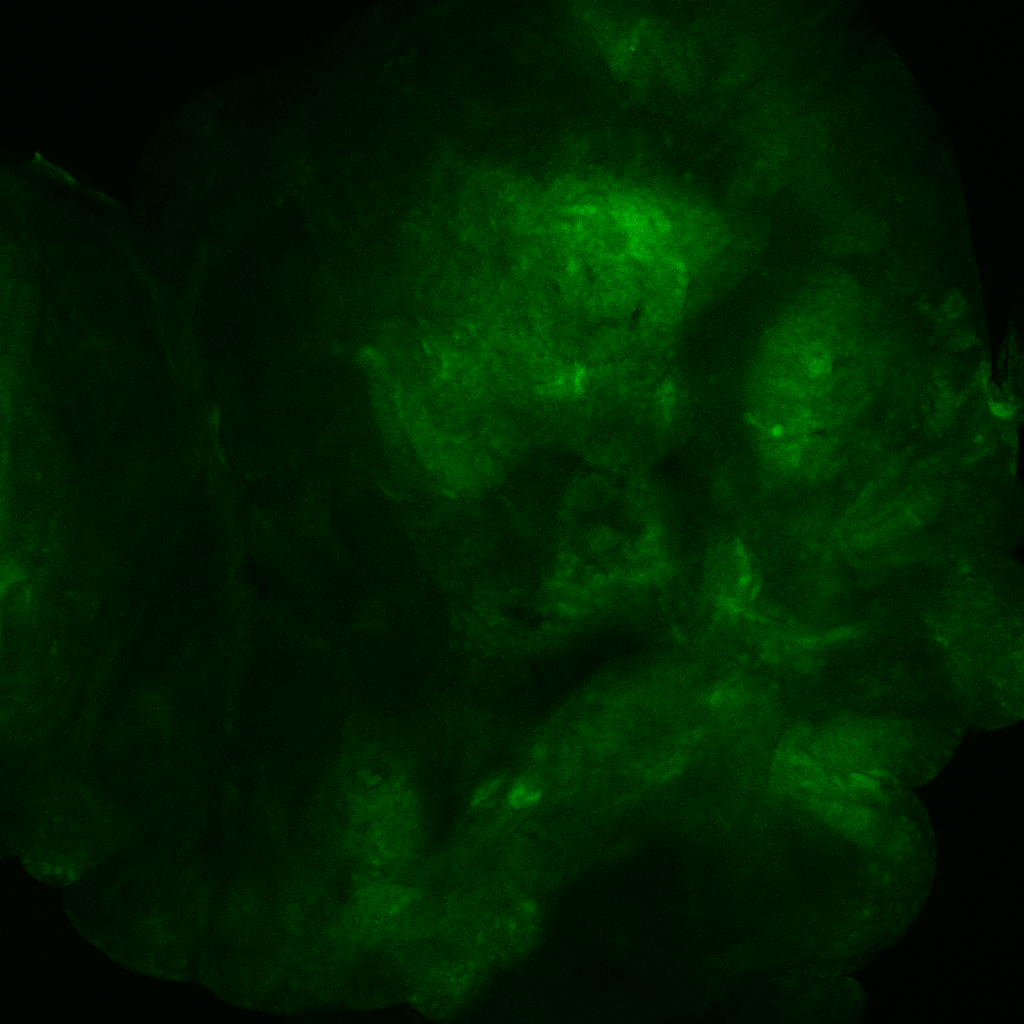

Supplement: Supplementary file 10 — Source data Fig. 6 [file 44318_2025_489_MOESM10_ESM.zip › Figure 6F/6 original image.tif]

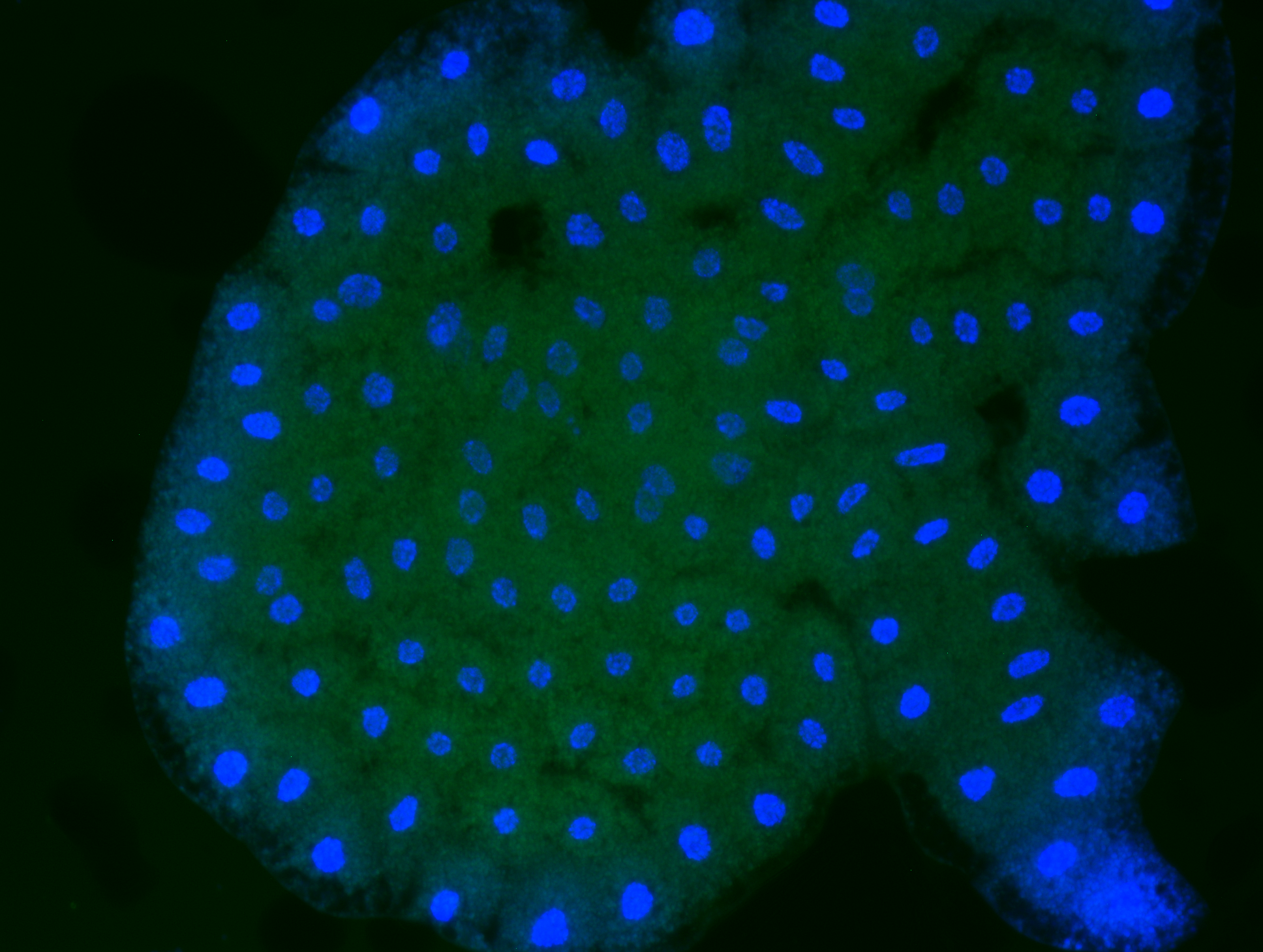

Supplement: Supplementary file 10 — Source data Fig. 6 [file 44318_2025_489_MOESM10_ESM.zip › Figure 6F/7 original image.tif]

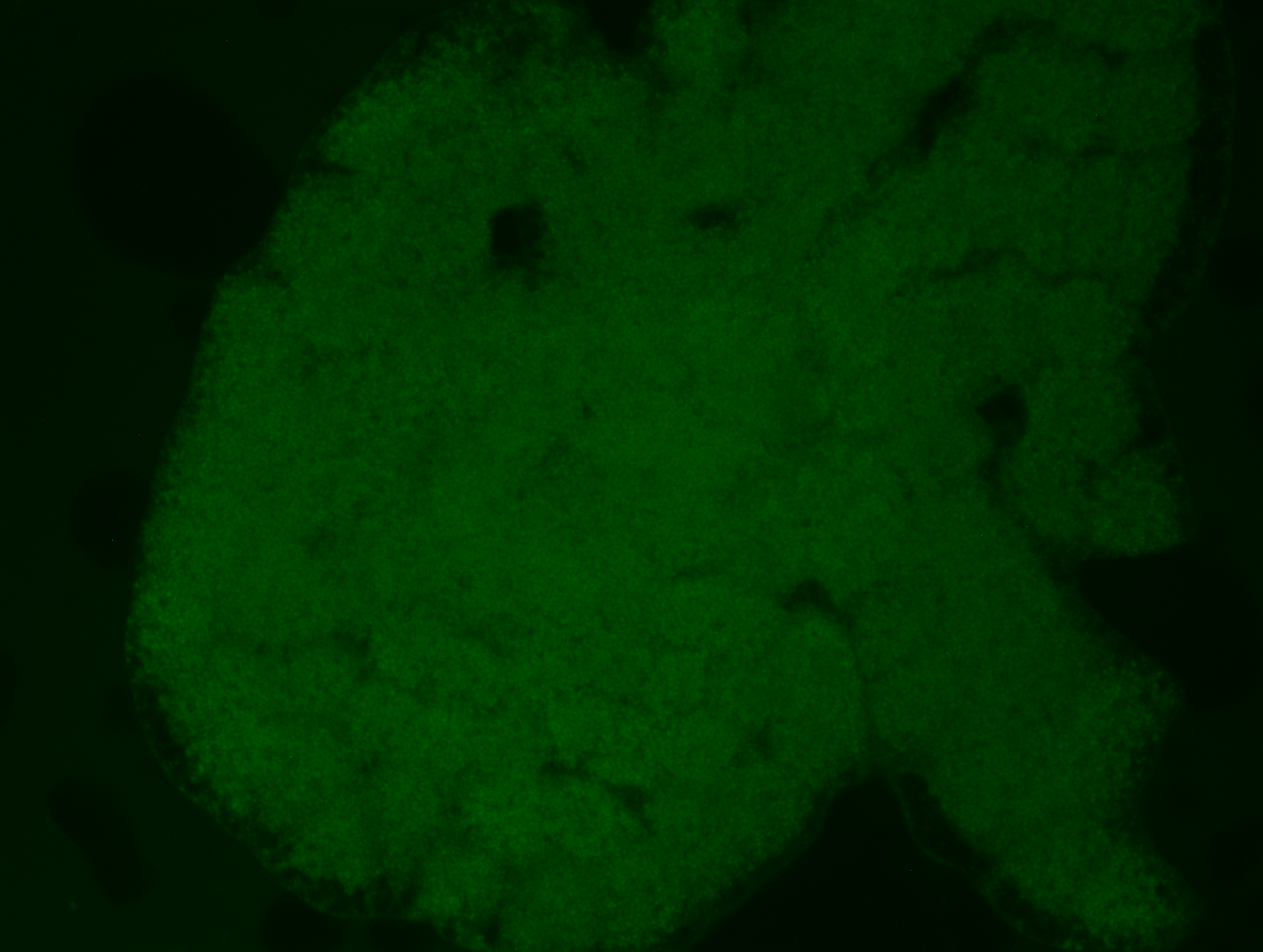

Supplement: Supplementary file 10 — Source data Fig. 6 [file 44318_2025_489_MOESM10_ESM.zip › Figure 6F/8 original image.tif]

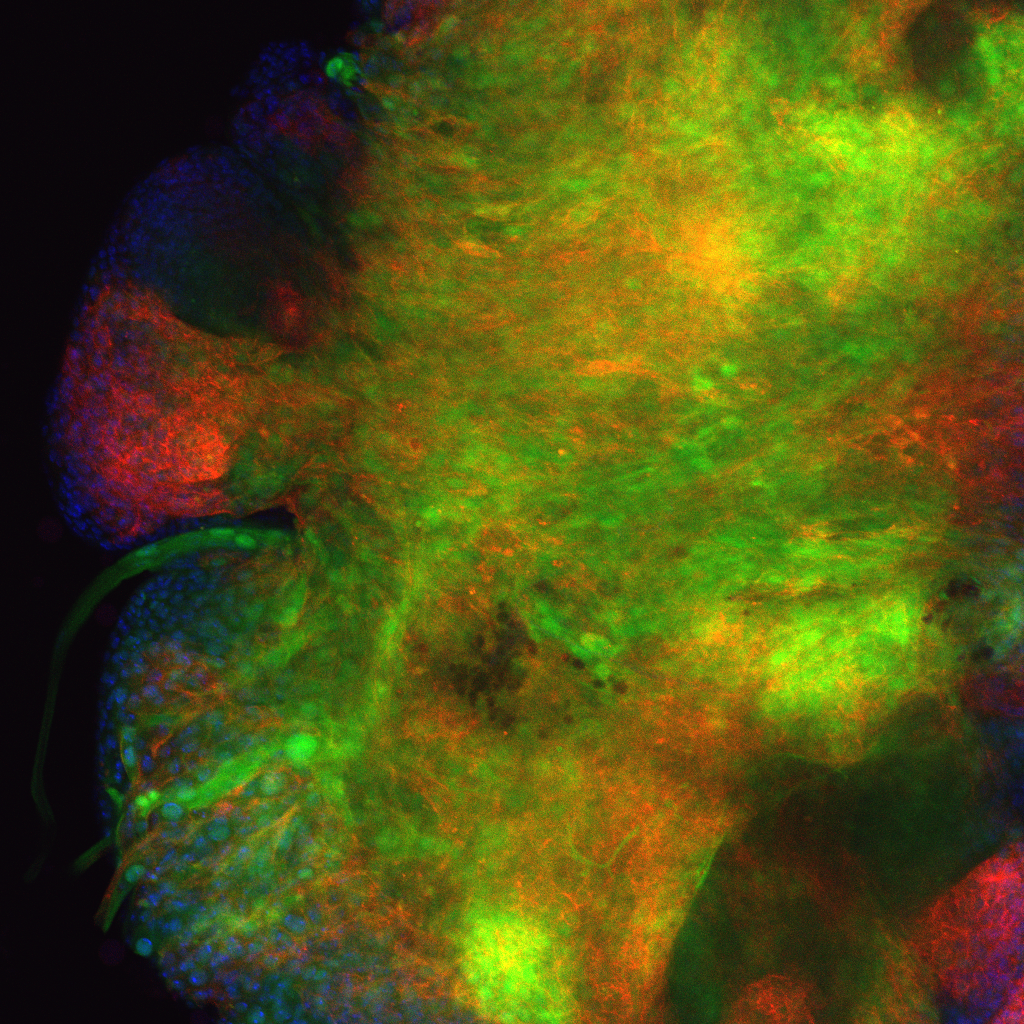

Supplement: Supplementary file 10 — Source data Fig. 6 [file 44318_2025_489_MOESM10_ESM.zip › Figure 6F/9 original image.tif]

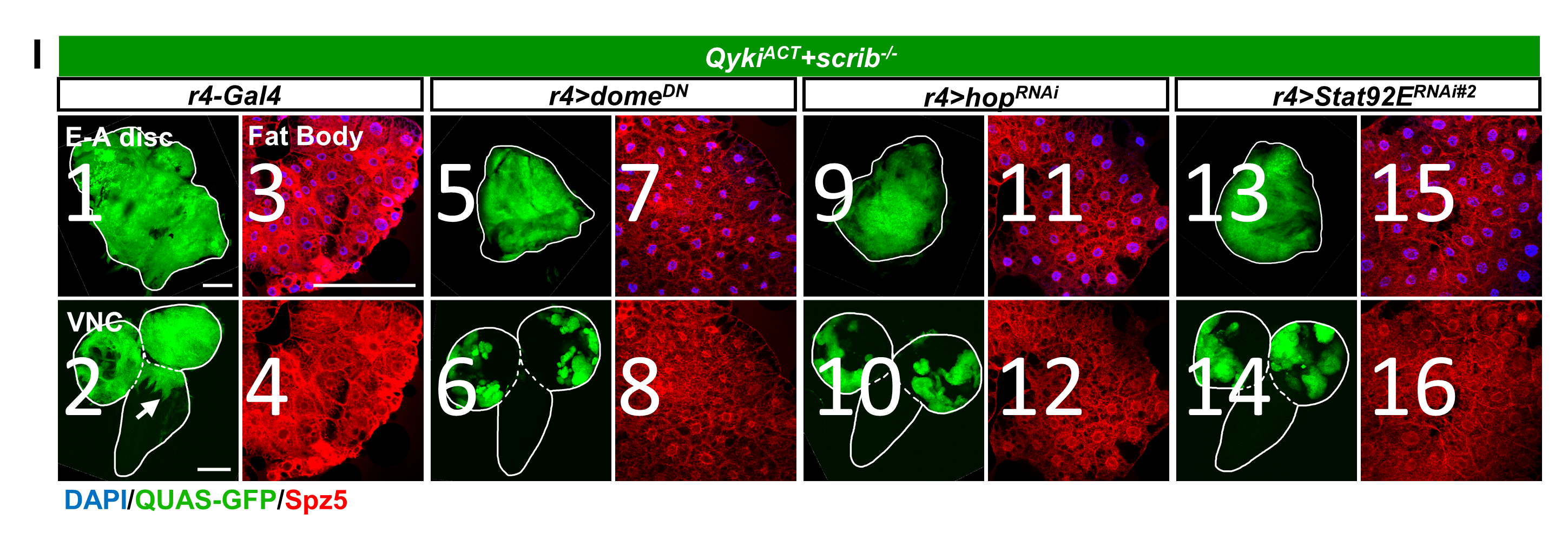

Supplement: Supplementary file 10 — Source data Fig. 6 [file 44318_2025_489_MOESM10_ESM.zip › Figure 6I/0 paper Figure 6I with provided image sequence.tif]

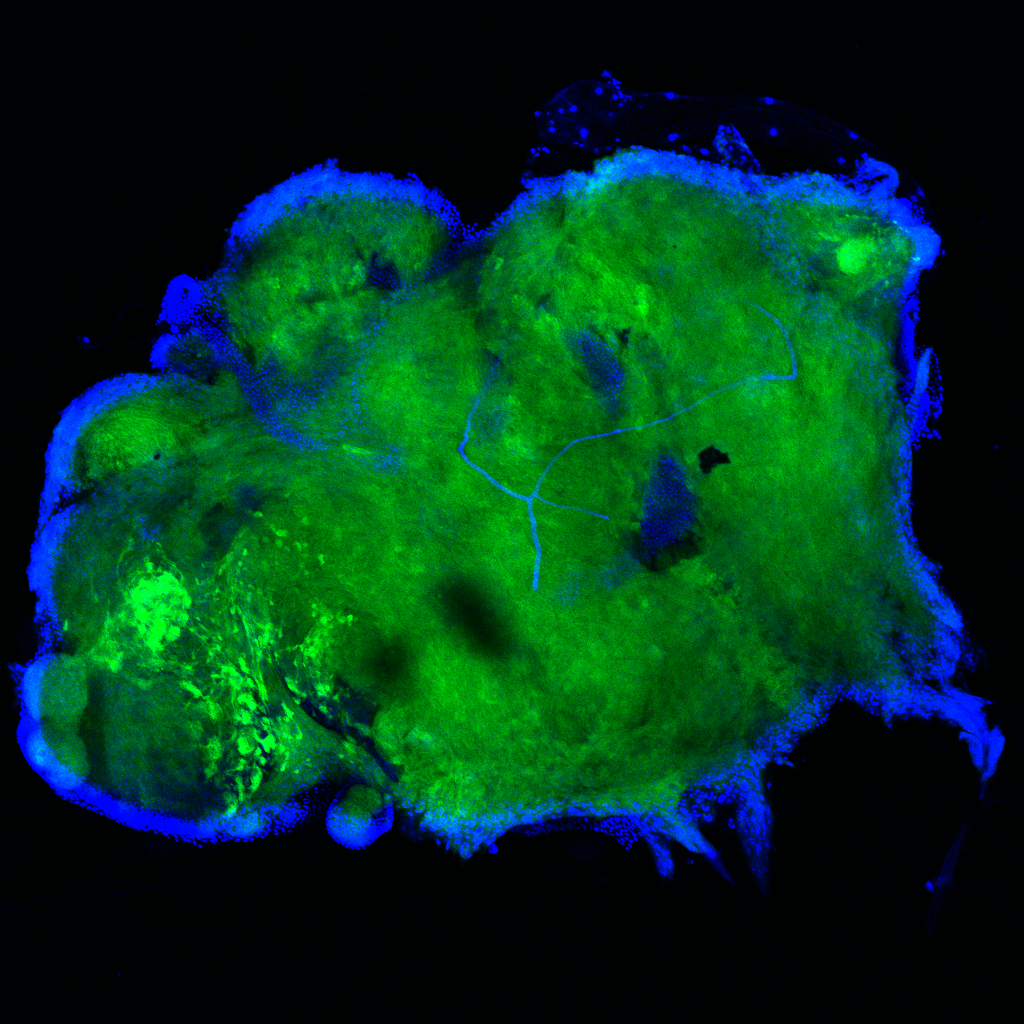

Supplement: Supplementary file 10 — Source data Fig. 6 [file 44318_2025_489_MOESM10_ESM.zip › Figure 6I/1 original image.tif]

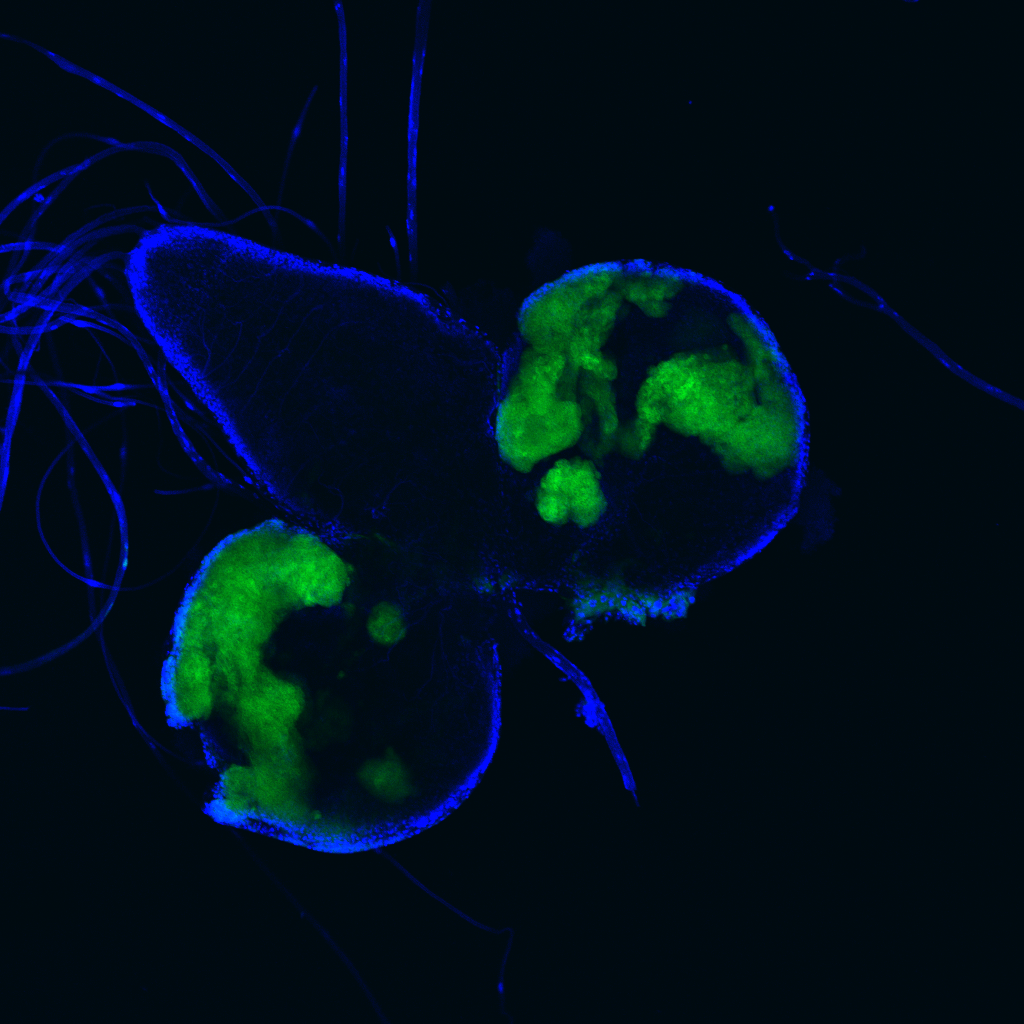

Supplement: Supplementary file 10 — Source data Fig. 6 [file 44318_2025_489_MOESM10_ESM.zip › Figure 6I/10 original image.tif]

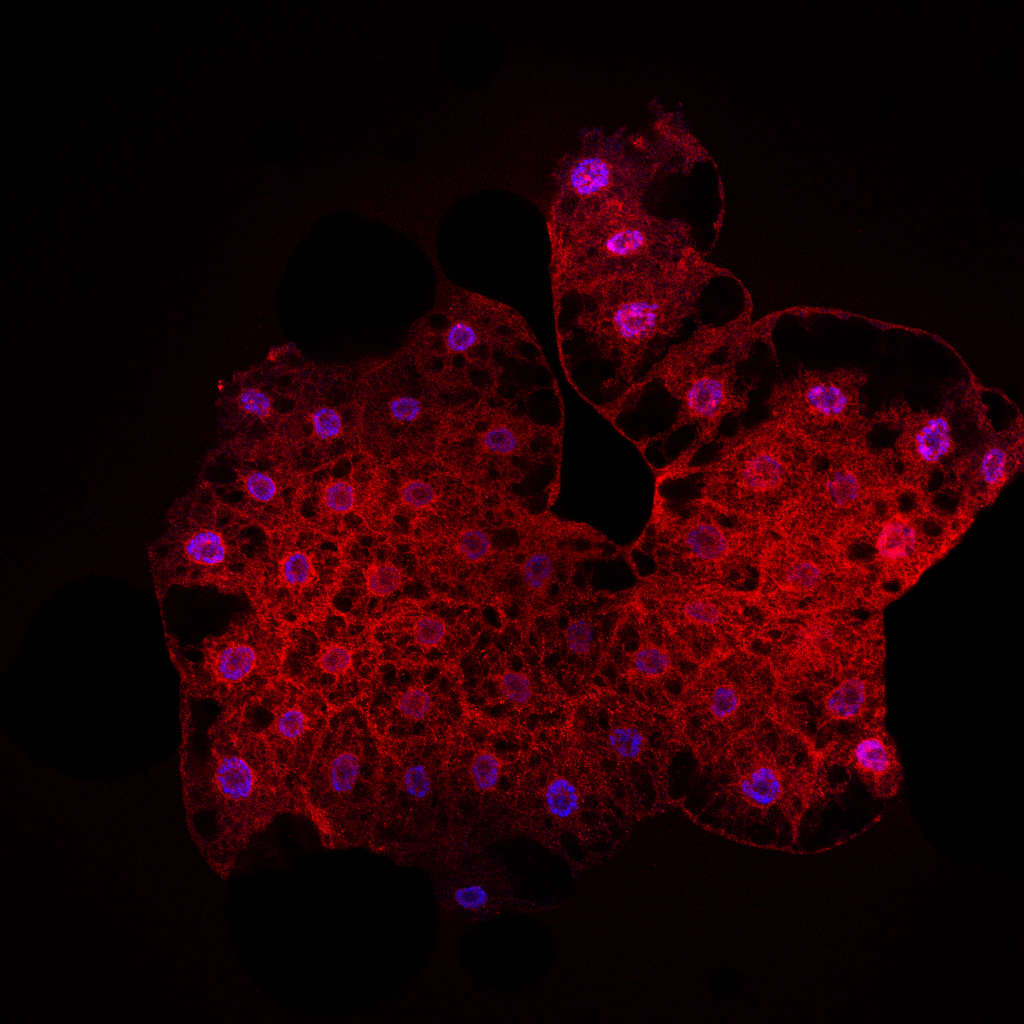

Supplement: Supplementary file 10 — Source data Fig. 6 [file 44318_2025_489_MOESM10_ESM.zip › Figure 6I/11 original image.tif]
