# Supplementary figures and images for: Adipose tissue-secreted Spz5 promotes distal tumor progression via Toll-6-mediated Hh pathway activation in Drosophila (part 5 of 5)
Source: EMBO J. 2025 Jun 23;44(15):4301–30. doi: 10.1038/s44318-025-00489-y (PMC12317064; doi:10.1038/s44318-025-00489-y)

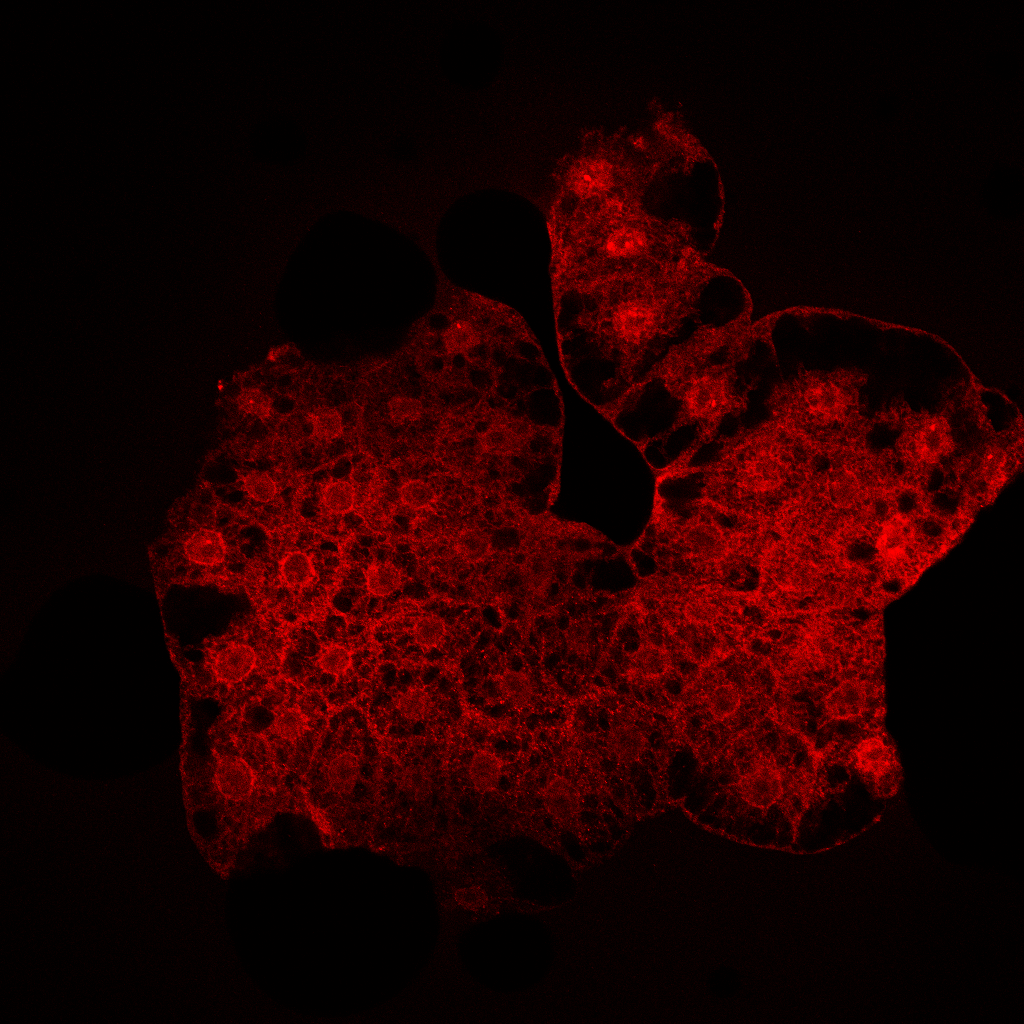

Supplement: Supplementary file 10 — Source data Fig. 6 [file 44318_2025_489_MOESM10_ESM.zip › Figure 6I/12 original image.tif]

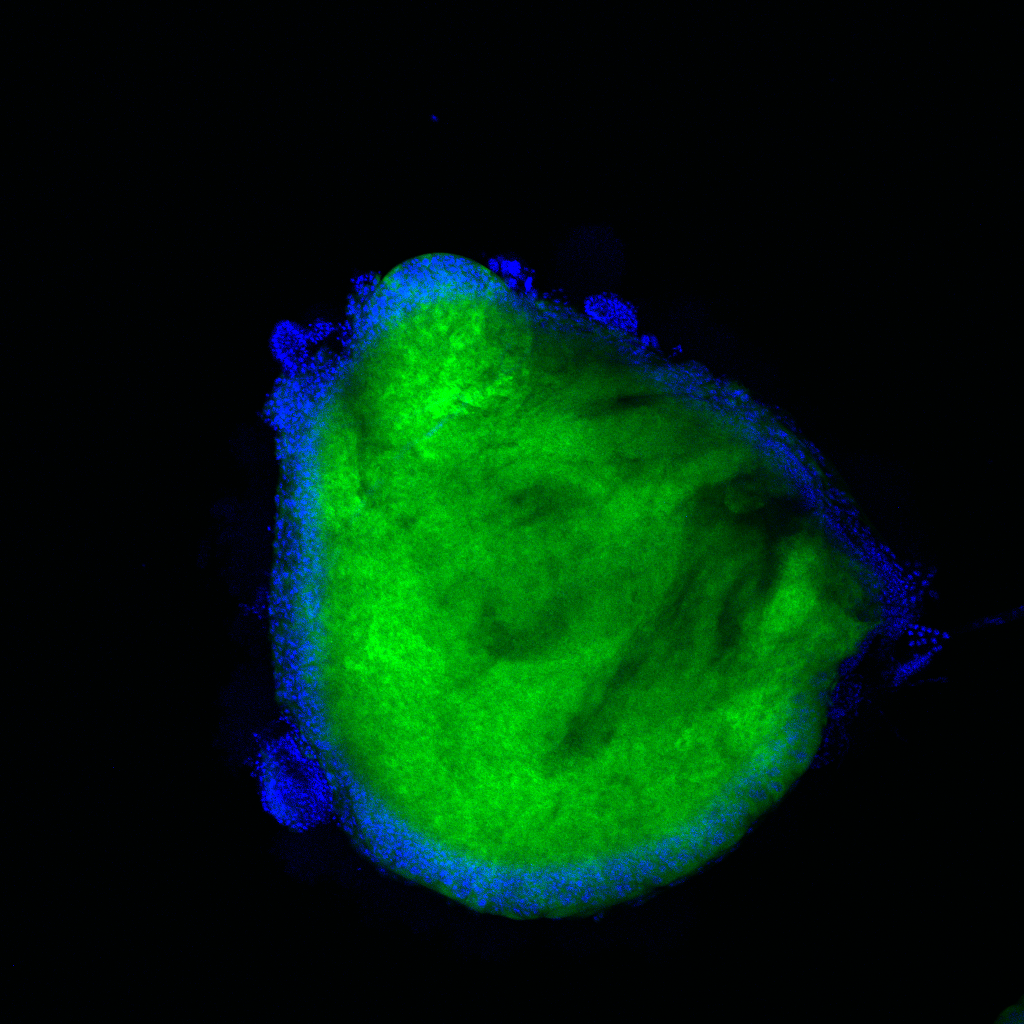

Supplement: Supplementary file 10 — Source data Fig. 6 [file 44318_2025_489_MOESM10_ESM.zip › Figure 6I/13 original image.tif]

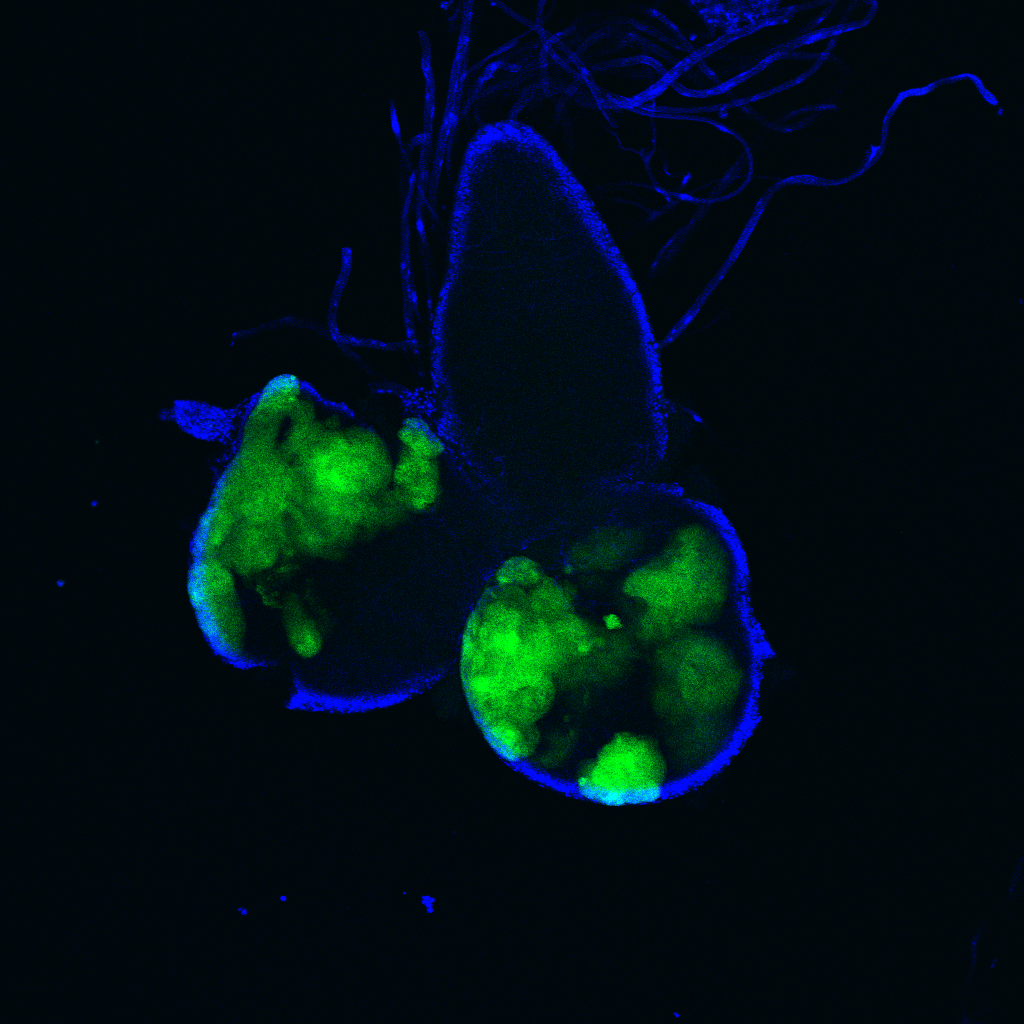

Supplement: Supplementary file 10 — Source data Fig. 6 [file 44318_2025_489_MOESM10_ESM.zip › Figure 6I/14 original image.tif]

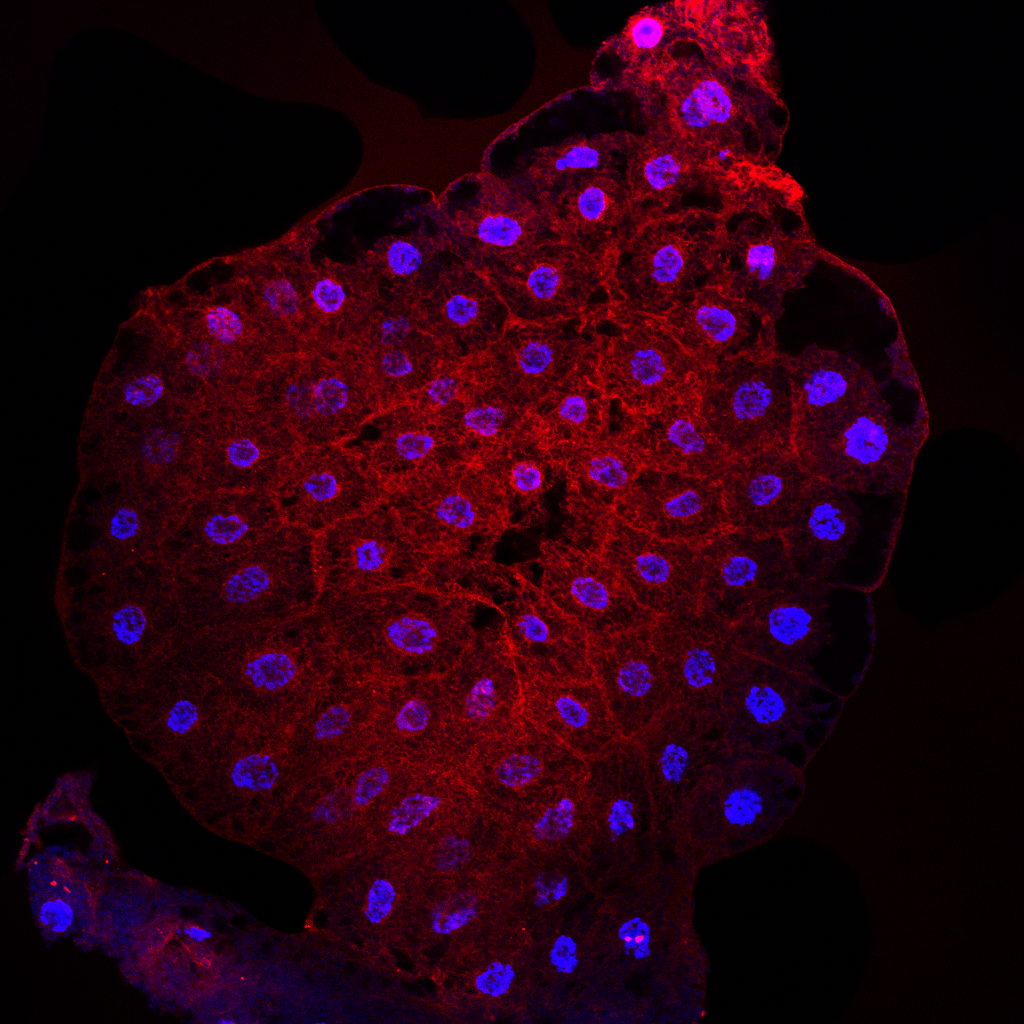

Supplement: Supplementary file 10 — Source data Fig. 6 [file 44318_2025_489_MOESM10_ESM.zip › Figure 6I/15 original image.tif]

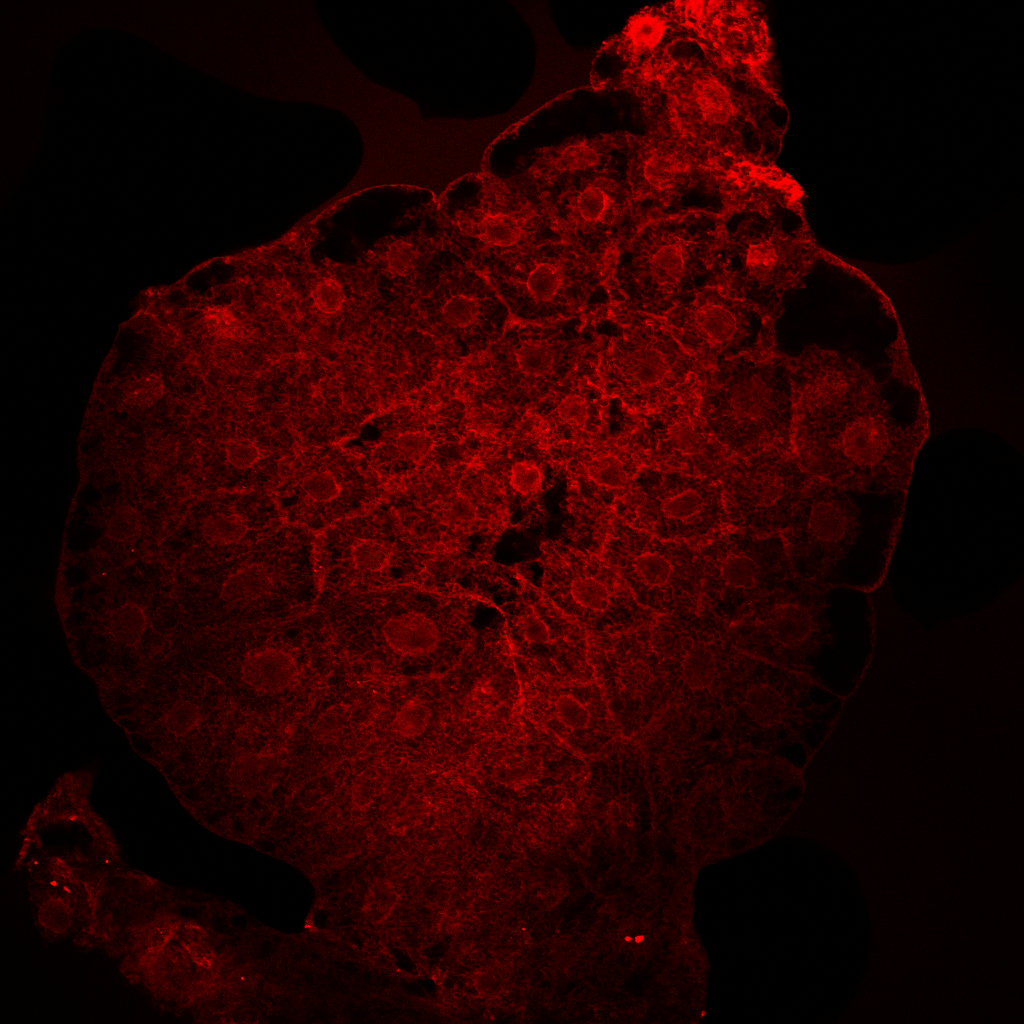

Supplement: Supplementary file 10 — Source data Fig. 6 [file 44318_2025_489_MOESM10_ESM.zip › Figure 6I/16 original image.tif]

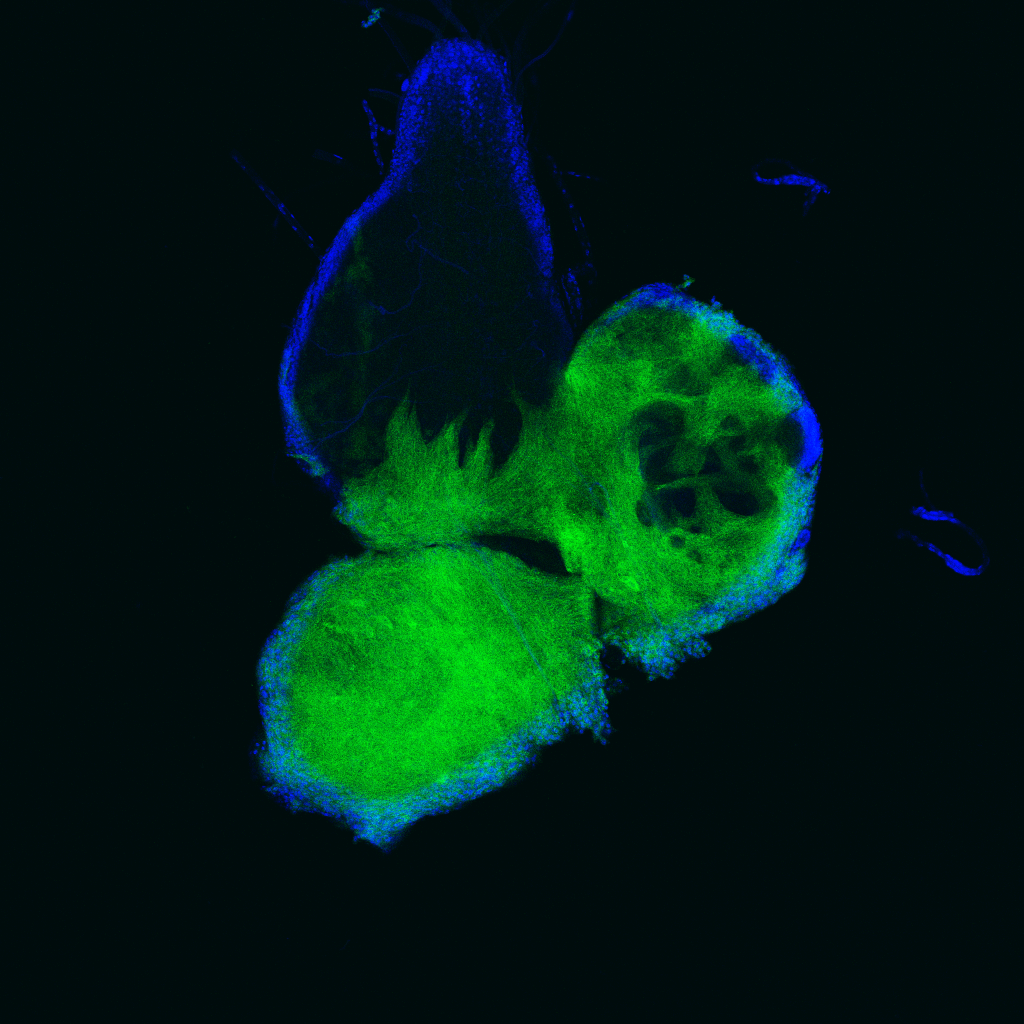

Supplement: Supplementary file 10 — Source data Fig. 6 [file 44318_2025_489_MOESM10_ESM.zip › Figure 6I/2 original image.tif]

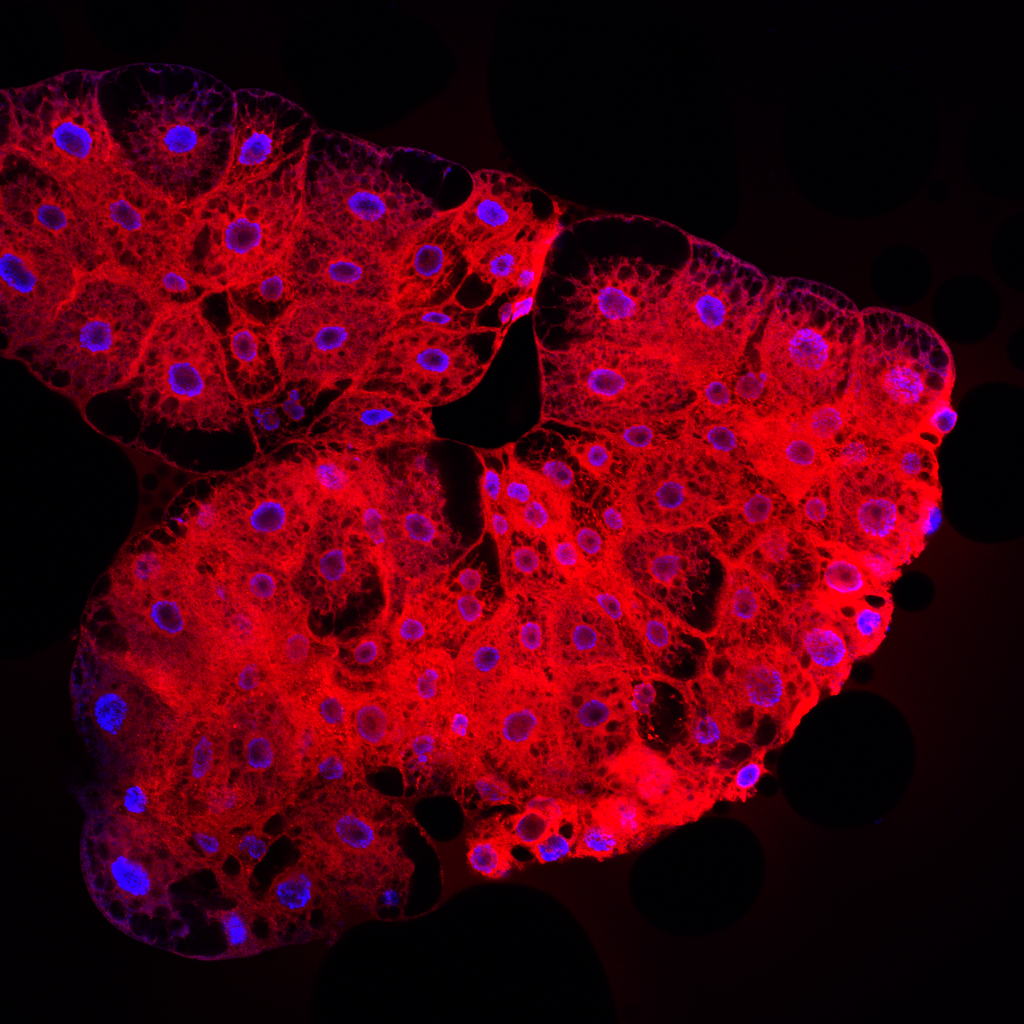

Supplement: Supplementary file 10 — Source data Fig. 6 [file 44318_2025_489_MOESM10_ESM.zip › Figure 6I/3 original image.tif]

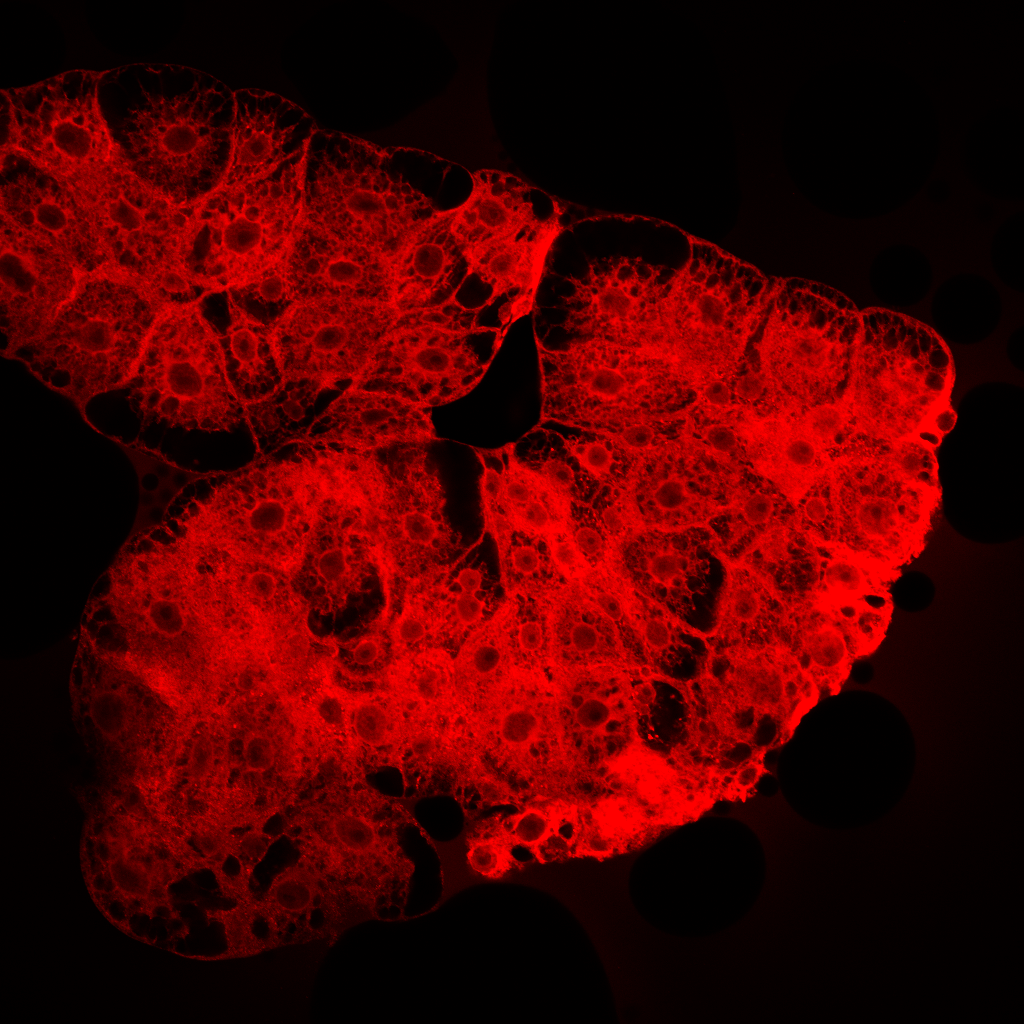

Supplement: Supplementary file 10 — Source data Fig. 6 [file 44318_2025_489_MOESM10_ESM.zip › Figure 6I/4 original image.tif]

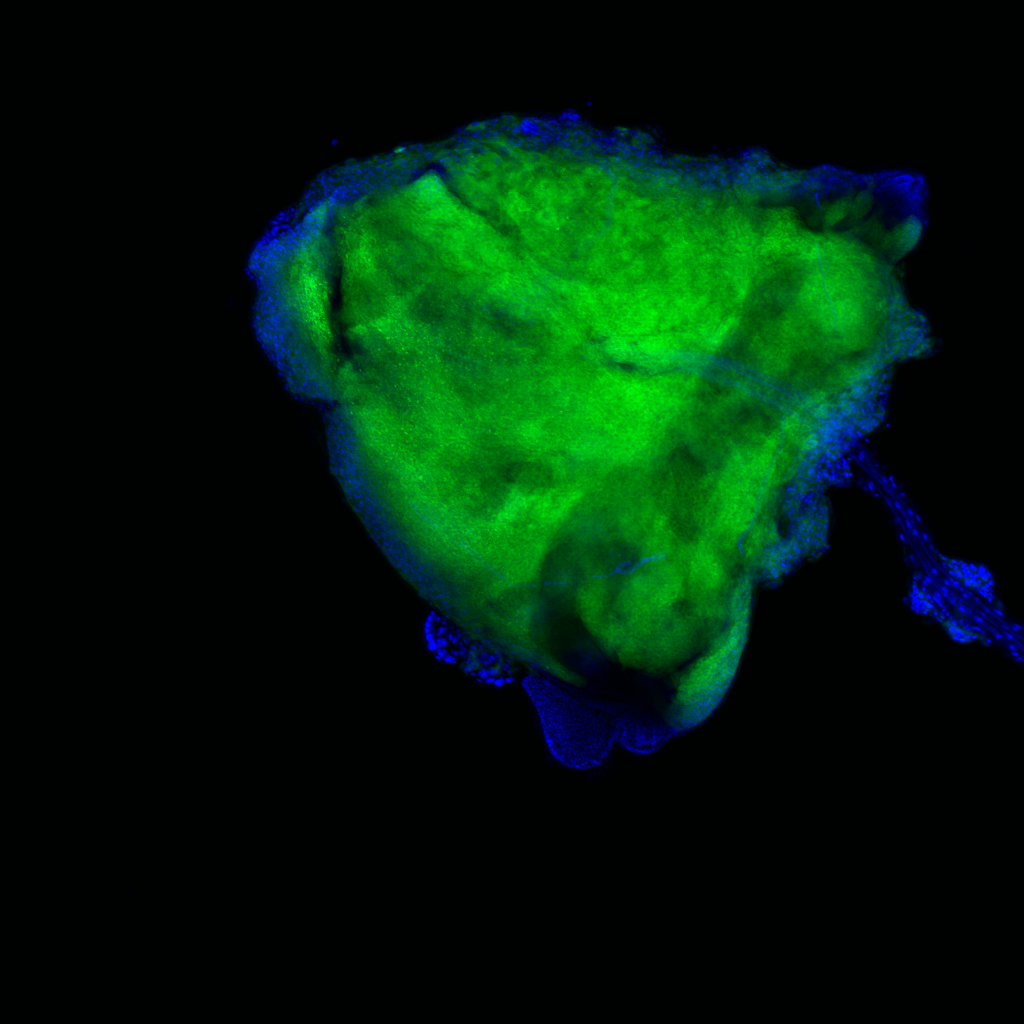

Supplement: Supplementary file 10 — Source data Fig. 6 [file 44318_2025_489_MOESM10_ESM.zip › Figure 6I/5 original image.tif]

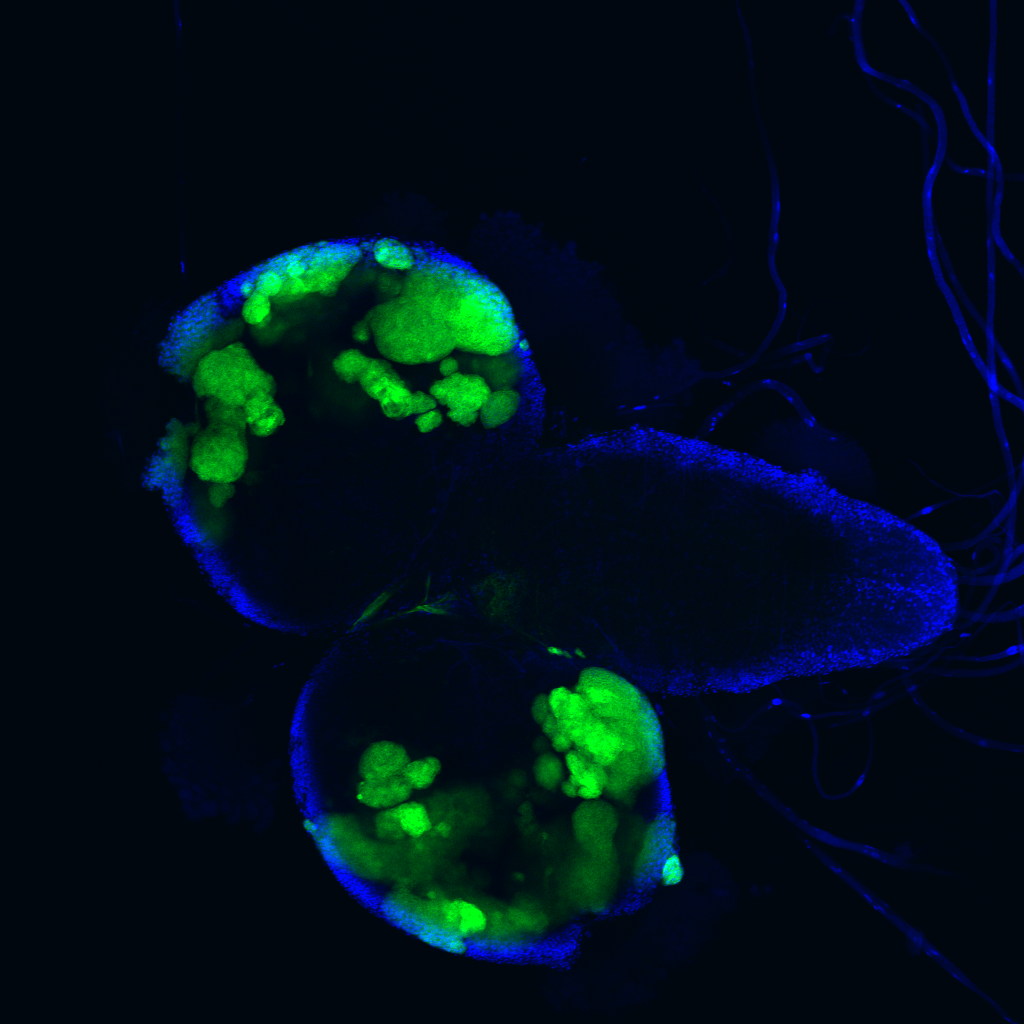

Supplement: Supplementary file 10 — Source data Fig. 6 [file 44318_2025_489_MOESM10_ESM.zip › Figure 6I/6 original image.tif]

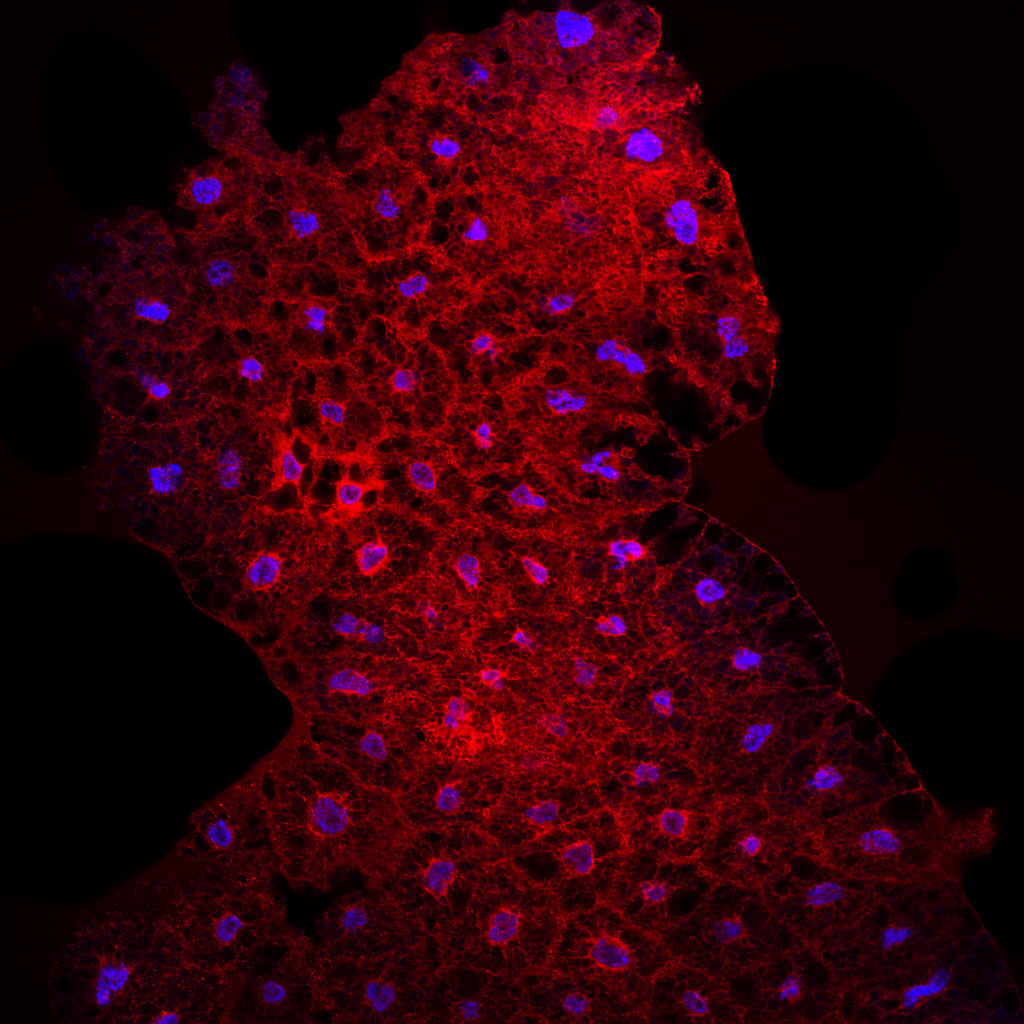

Supplement: Supplementary file 10 — Source data Fig. 6 [file 44318_2025_489_MOESM10_ESM.zip › Figure 6I/7 original image.tif]

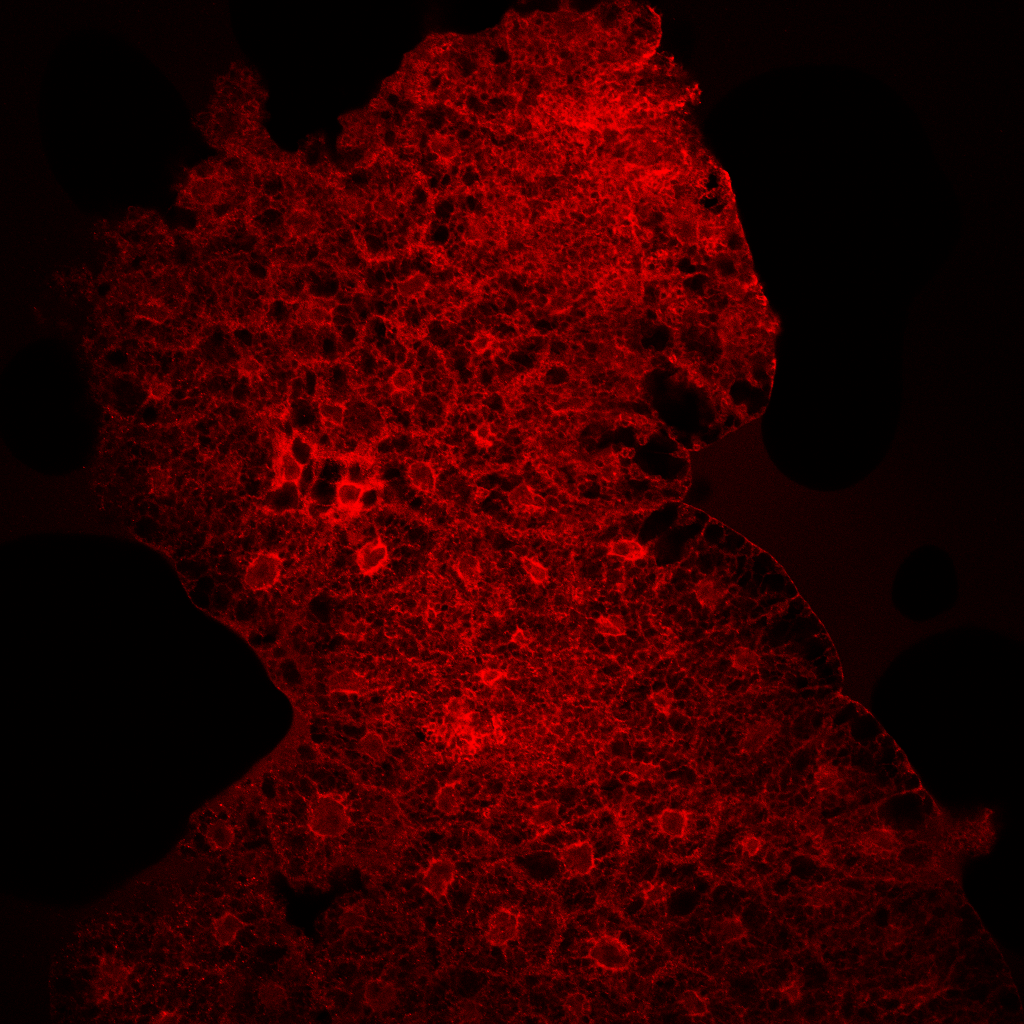

Supplement: Supplementary file 10 — Source data Fig. 6 [file 44318_2025_489_MOESM10_ESM.zip › Figure 6I/8 original image.tif]

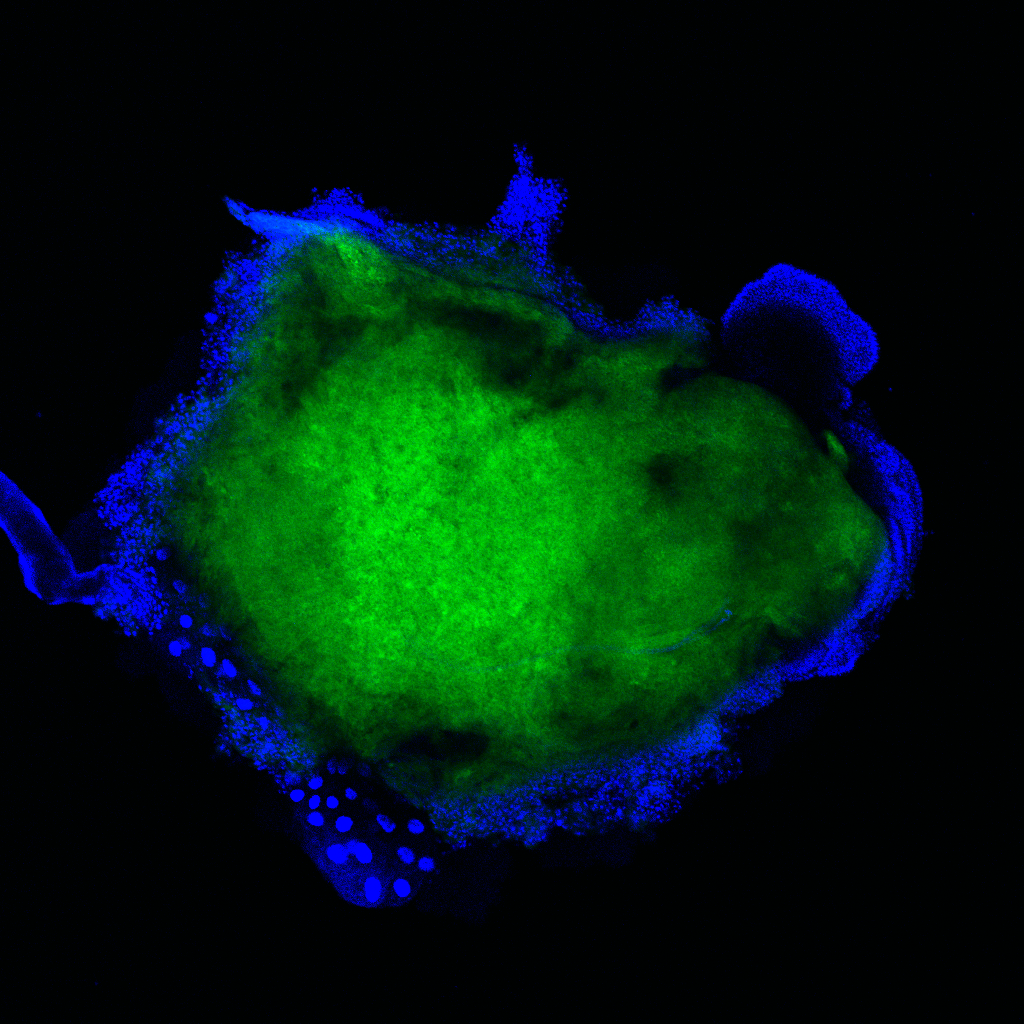

Supplement: Supplementary file 10 — Source data Fig. 6 [file 44318_2025_489_MOESM10_ESM.zip › Figure 6I/9 original image.tif]

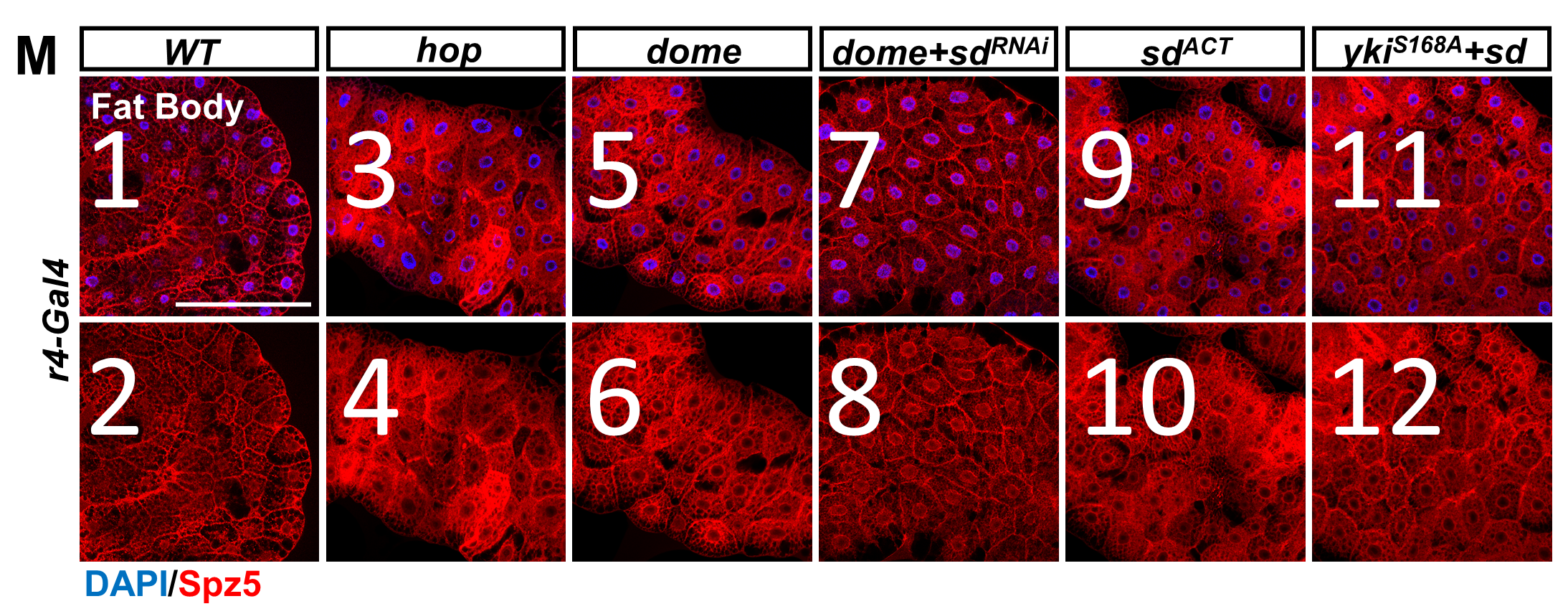

Supplement: Supplementary file 10 — Source data Fig. 6 [file 44318_2025_489_MOESM10_ESM.zip › Figure 6M/0 paper Figure 6M with provided image sequence.tif]

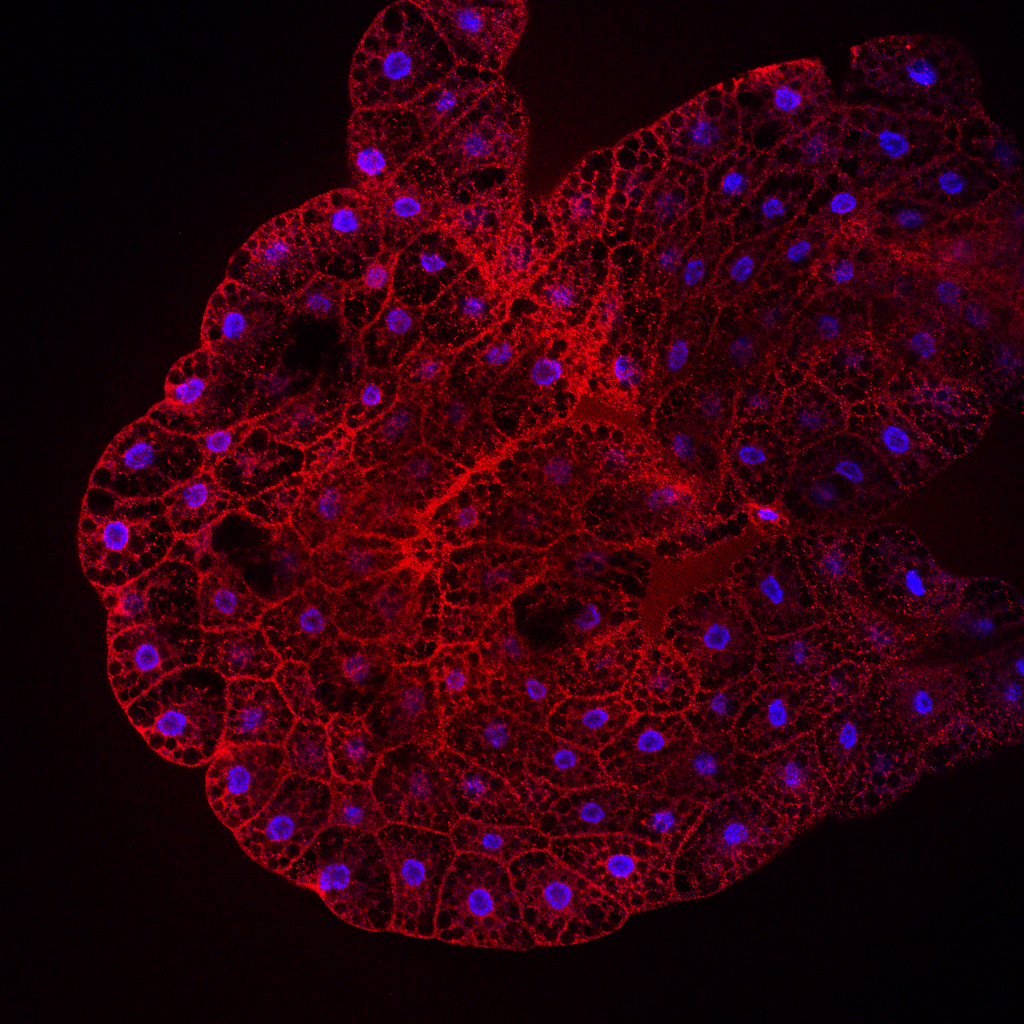

Supplement: Supplementary file 10 — Source data Fig. 6 [file 44318_2025_489_MOESM10_ESM.zip › Figure 6M/1 original image.tif]

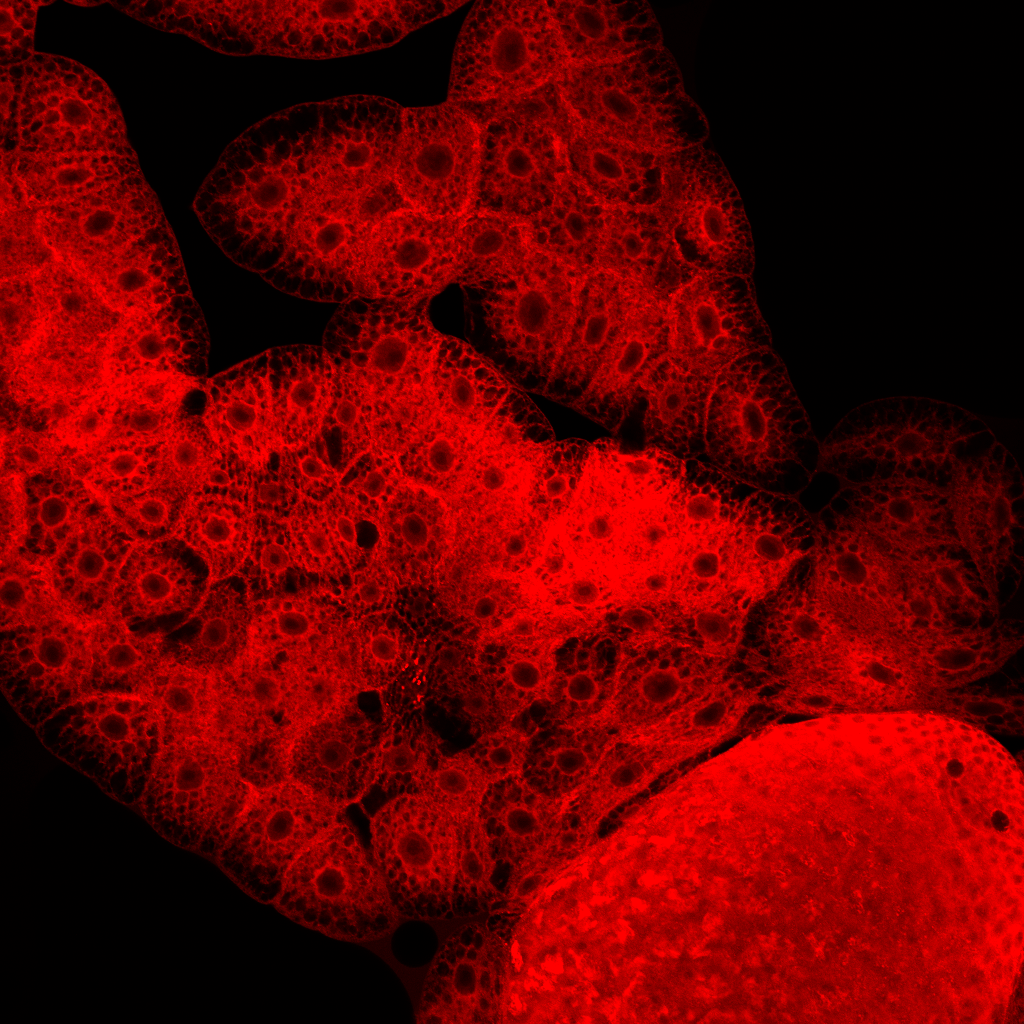

Supplement: Supplementary file 10 — Source data Fig. 6 [file 44318_2025_489_MOESM10_ESM.zip › Figure 6M/10 original image.tif]

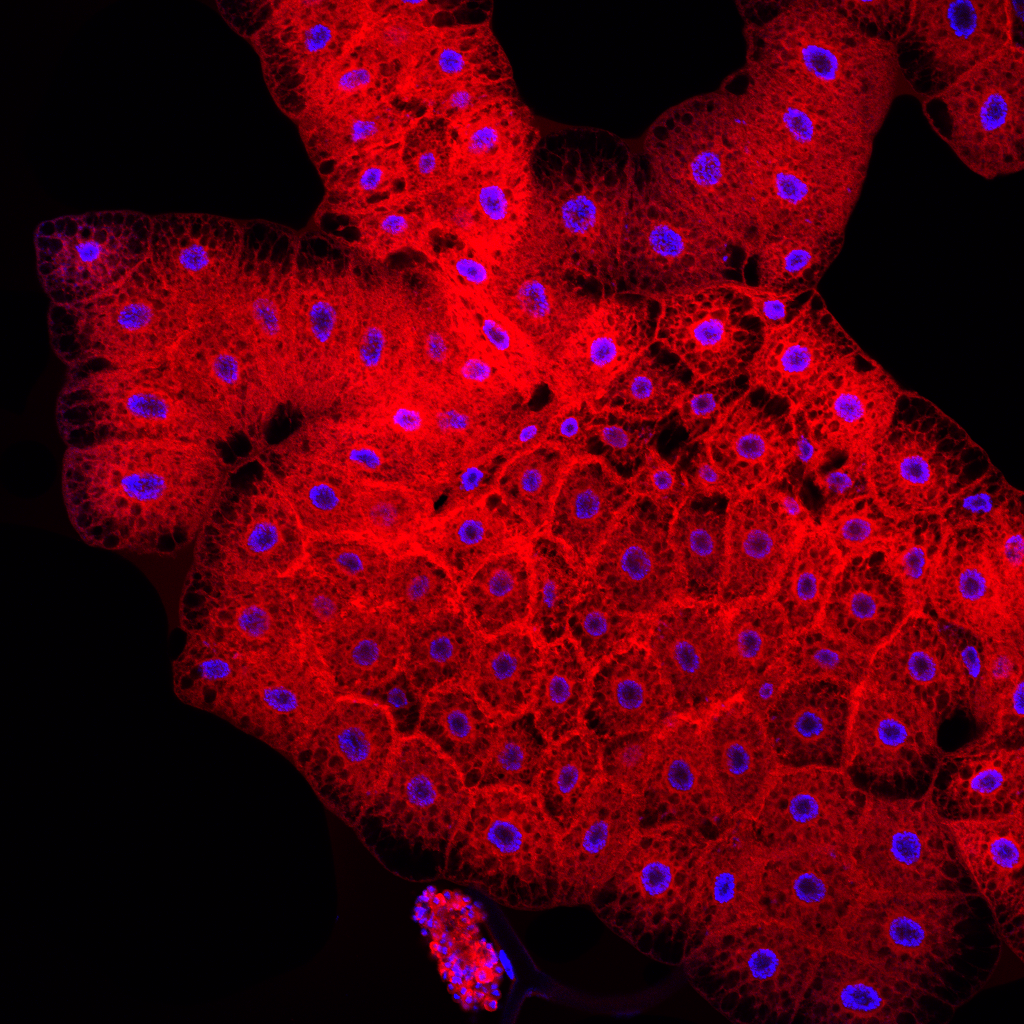

Supplement: Supplementary file 10 — Source data Fig. 6 [file 44318_2025_489_MOESM10_ESM.zip › Figure 6M/11 original image.tif]

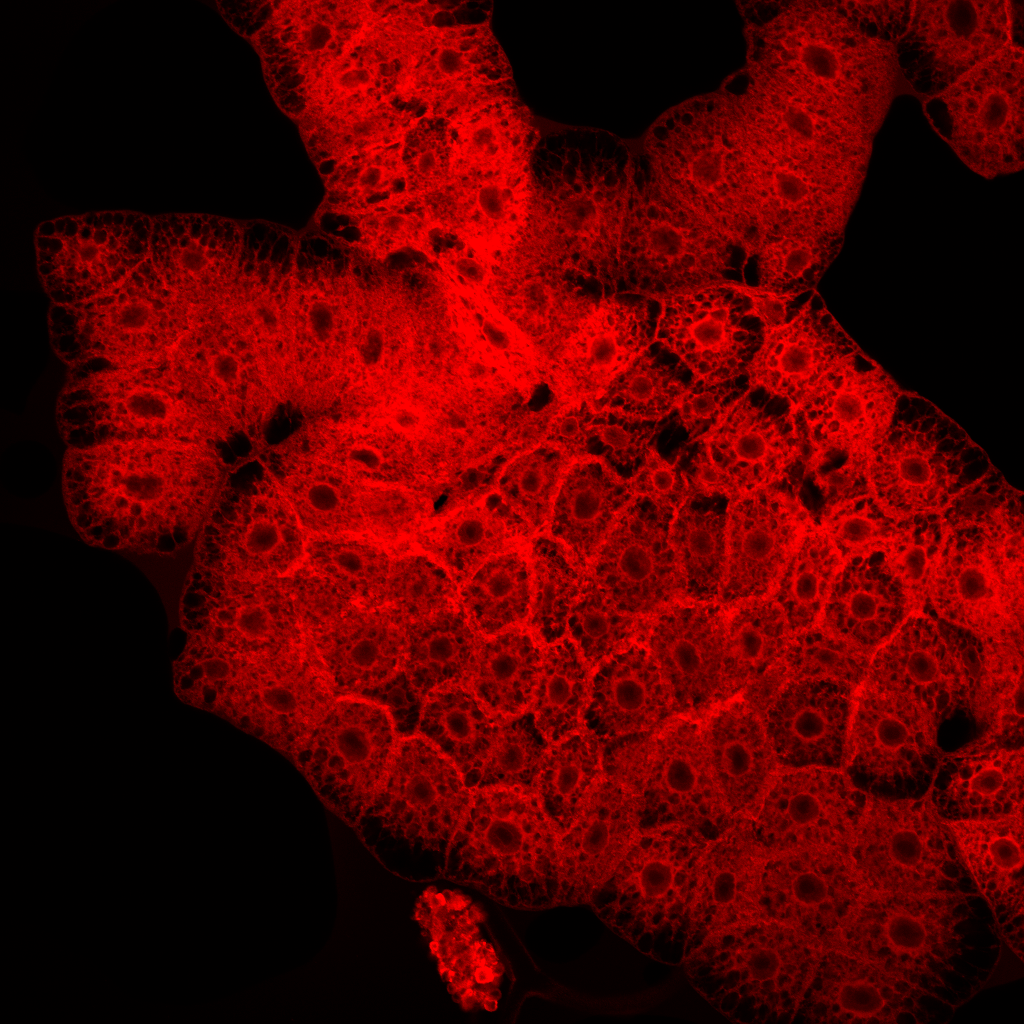

Supplement: Supplementary file 10 — Source data Fig. 6 [file 44318_2025_489_MOESM10_ESM.zip › Figure 6M/12 original image.tif]

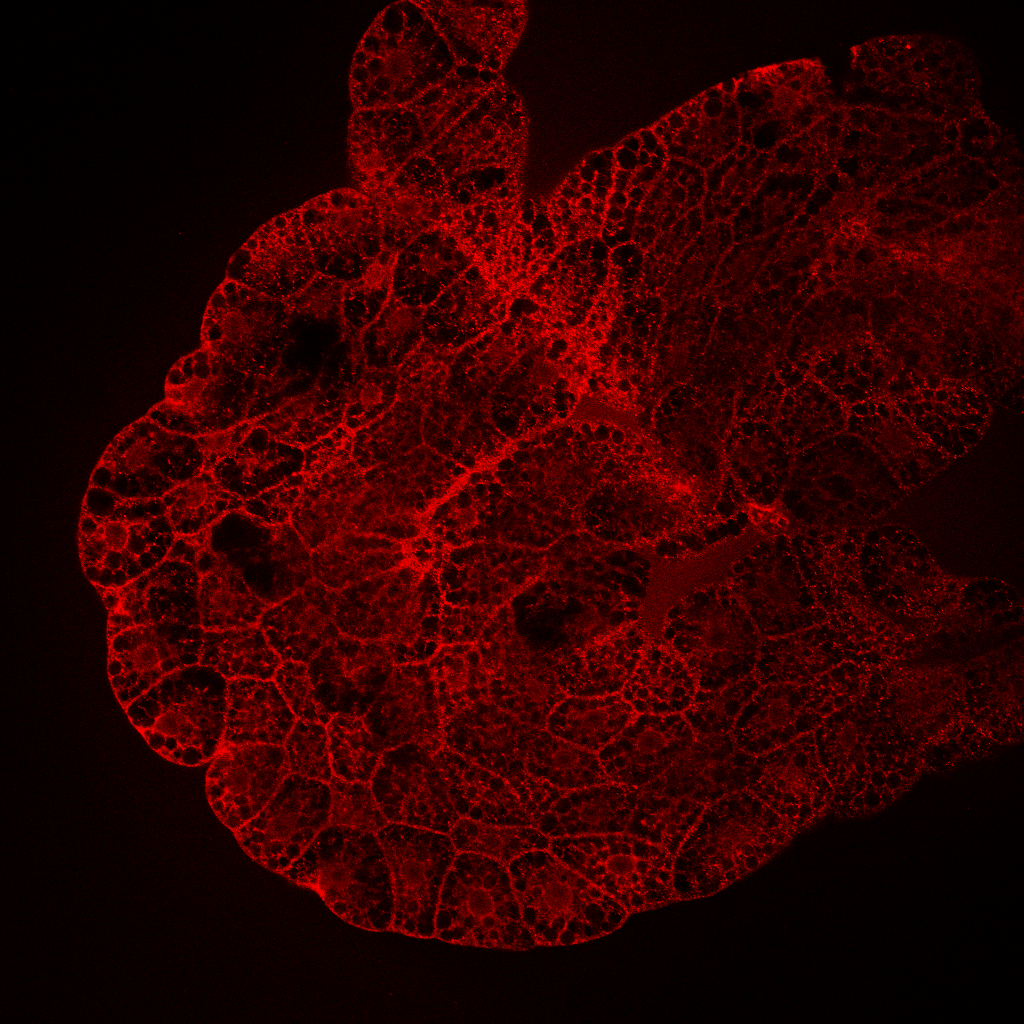

Supplement: Supplementary file 10 — Source data Fig. 6 [file 44318_2025_489_MOESM10_ESM.zip › Figure 6M/2 original image.tif]

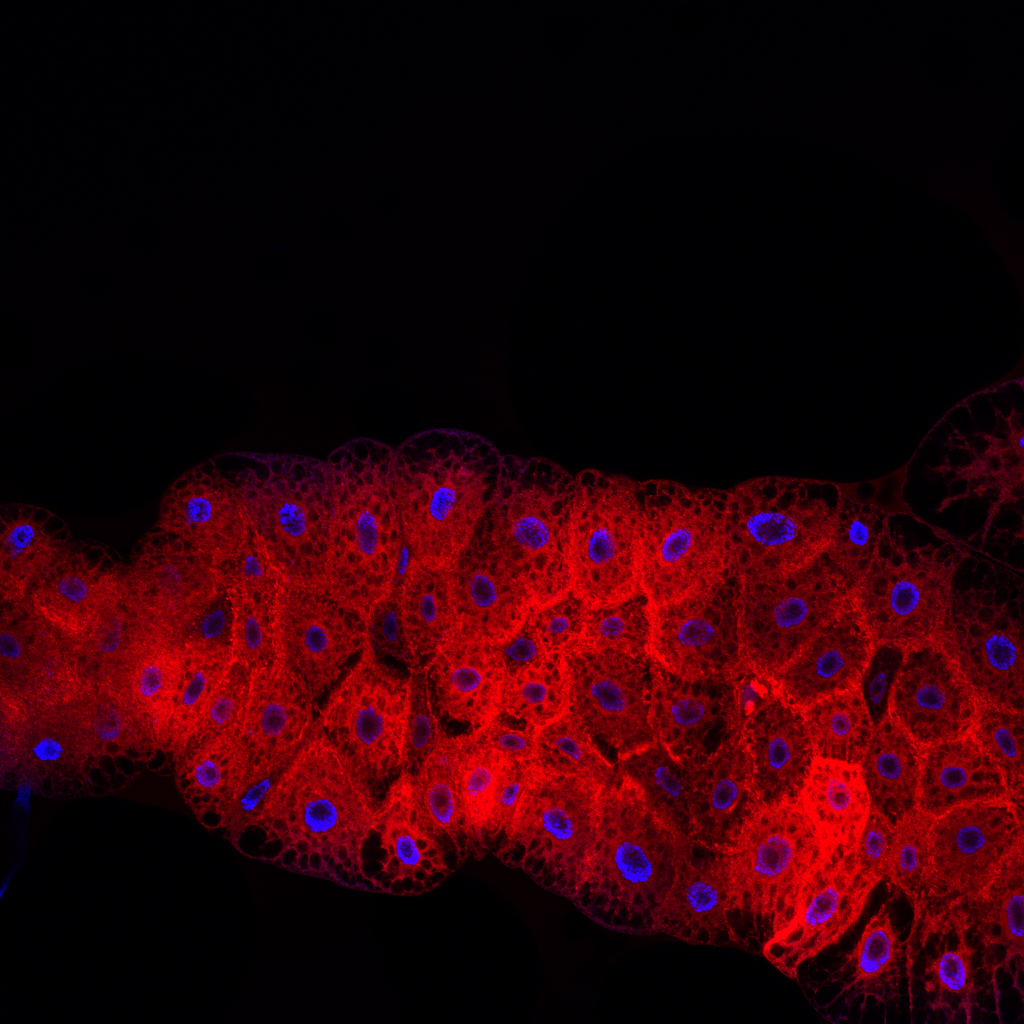

Supplement: Supplementary file 10 — Source data Fig. 6 [file 44318_2025_489_MOESM10_ESM.zip › Figure 6M/3 original image.tif]

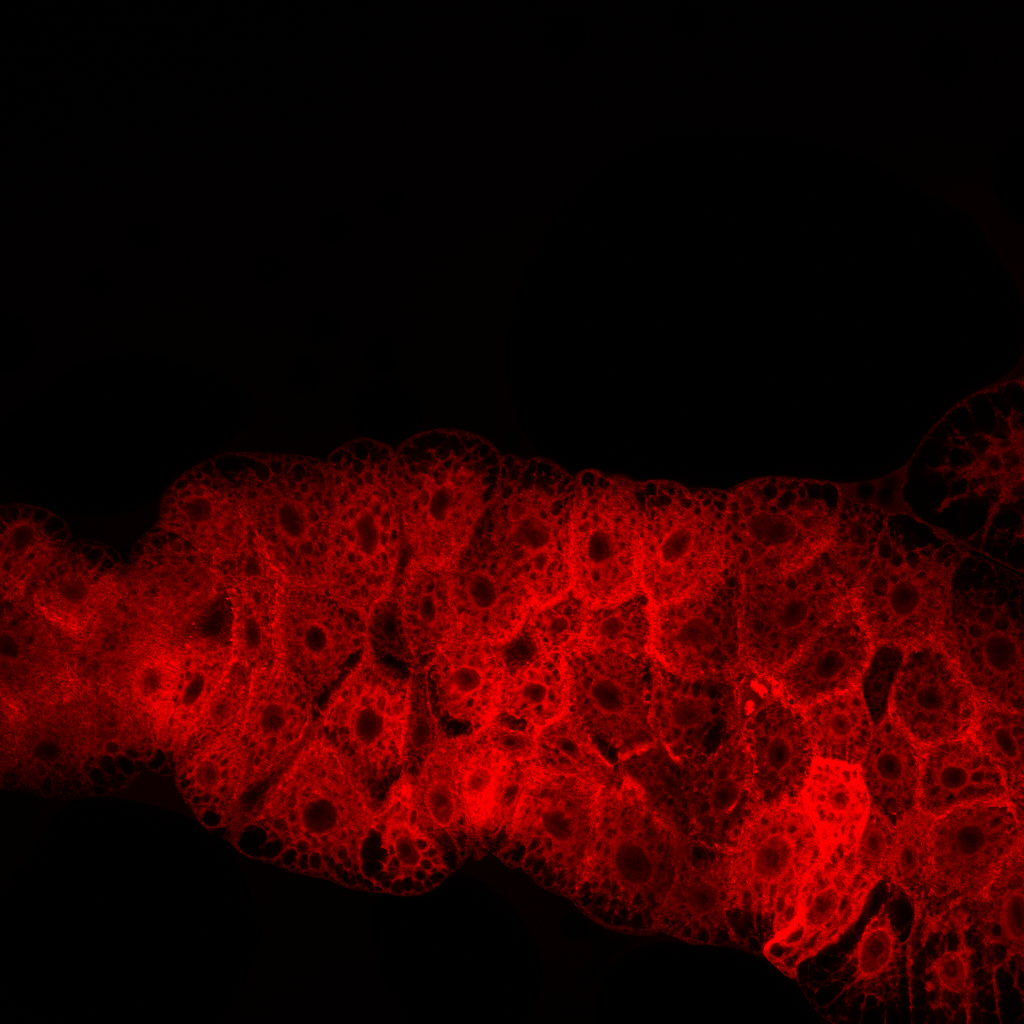

Supplement: Supplementary file 10 — Source data Fig. 6 [file 44318_2025_489_MOESM10_ESM.zip › Figure 6M/4 original image.tif]

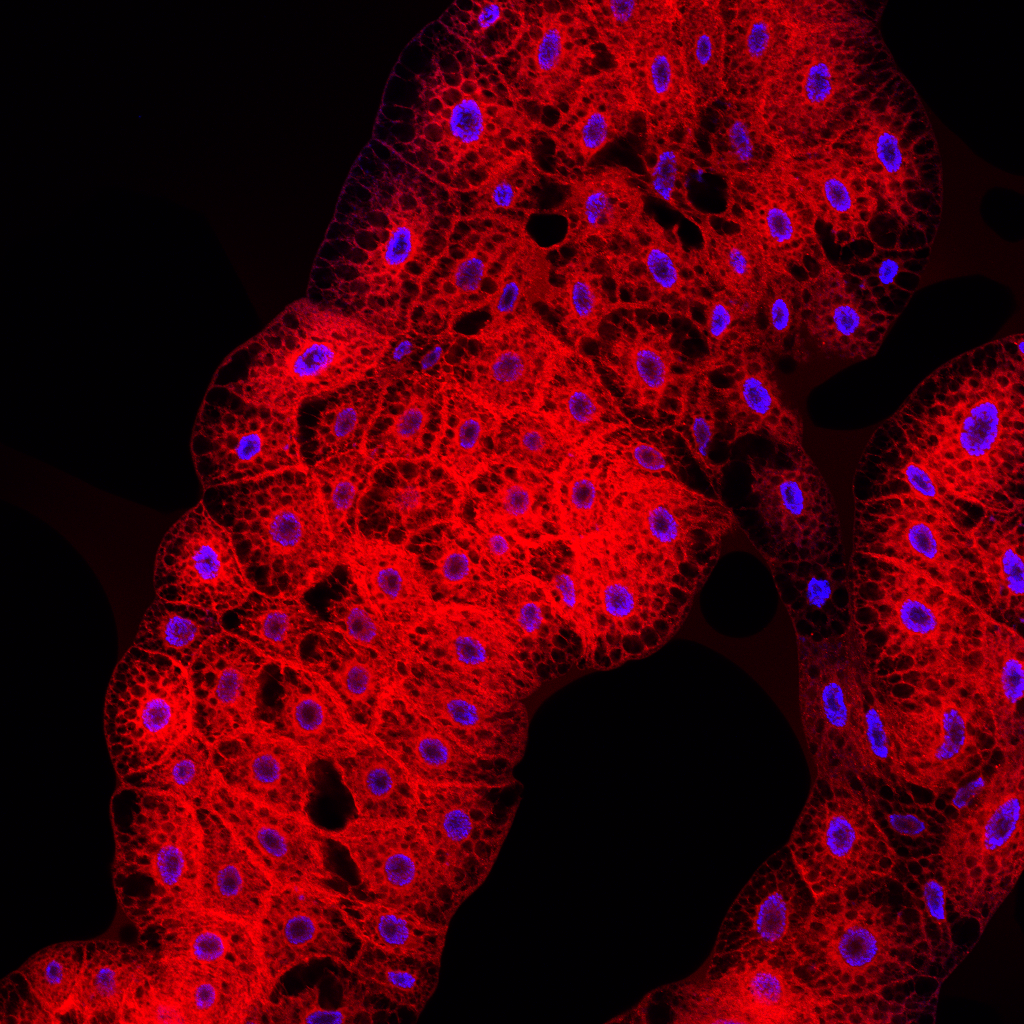

Supplement: Supplementary file 10 — Source data Fig. 6 [file 44318_2025_489_MOESM10_ESM.zip › Figure 6M/5 original image.tif]

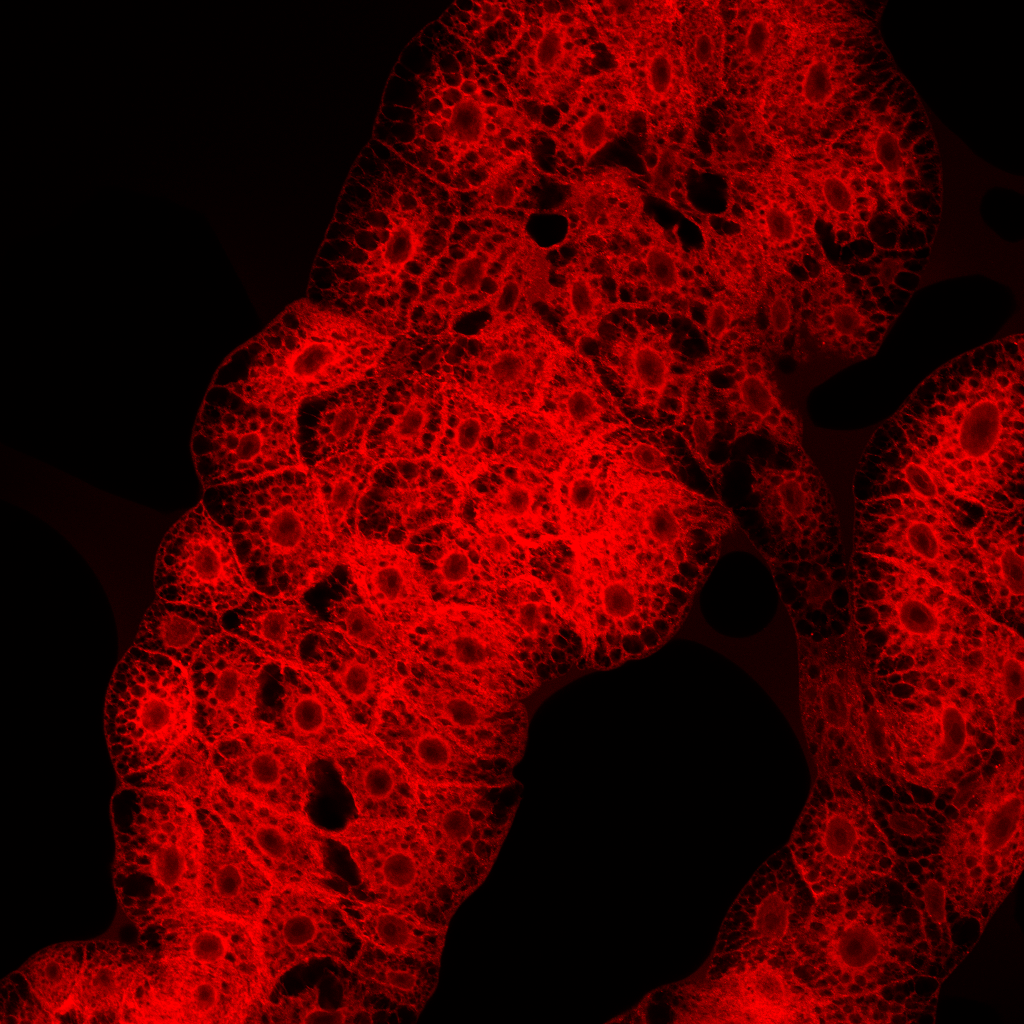

Supplement: Supplementary file 10 — Source data Fig. 6 [file 44318_2025_489_MOESM10_ESM.zip › Figure 6M/6 original image.tif]

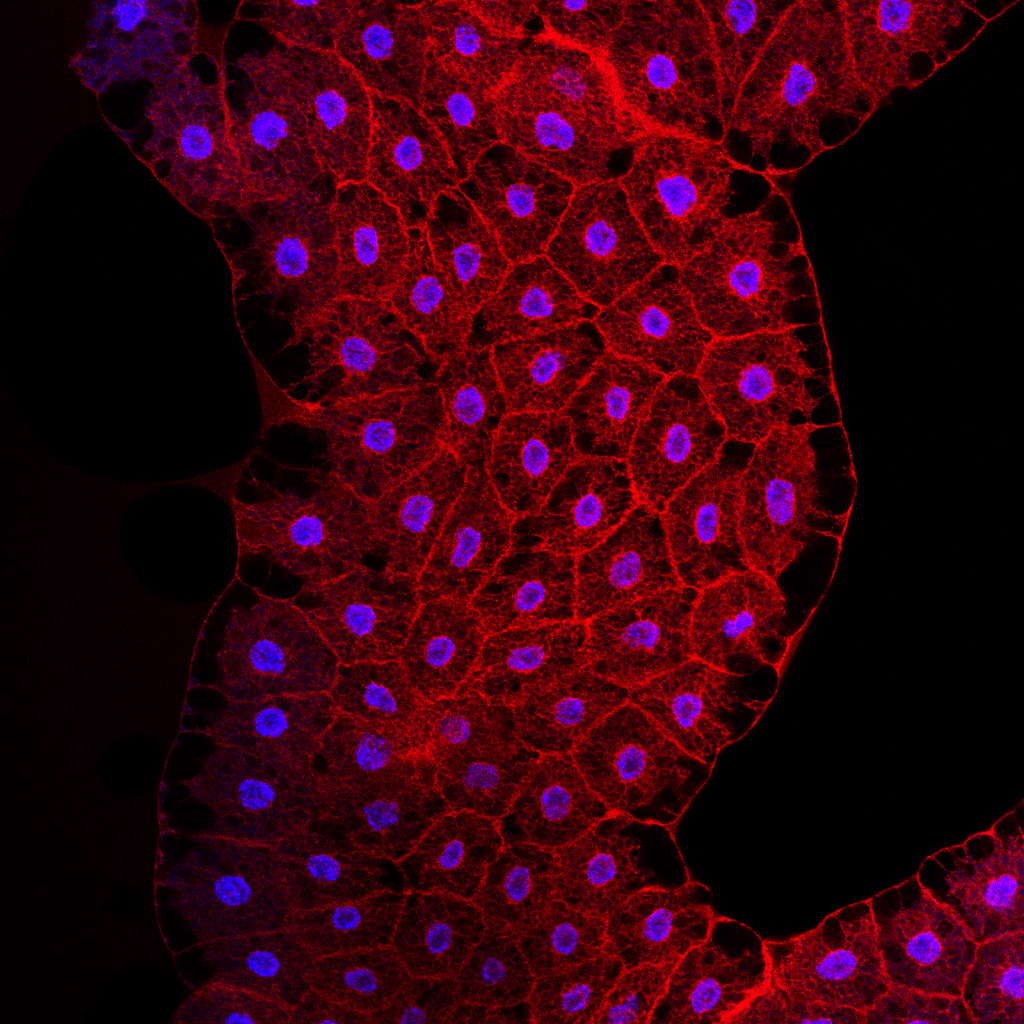

Supplement: Supplementary file 10 — Source data Fig. 6 [file 44318_2025_489_MOESM10_ESM.zip › Figure 6M/7 original image.tif]

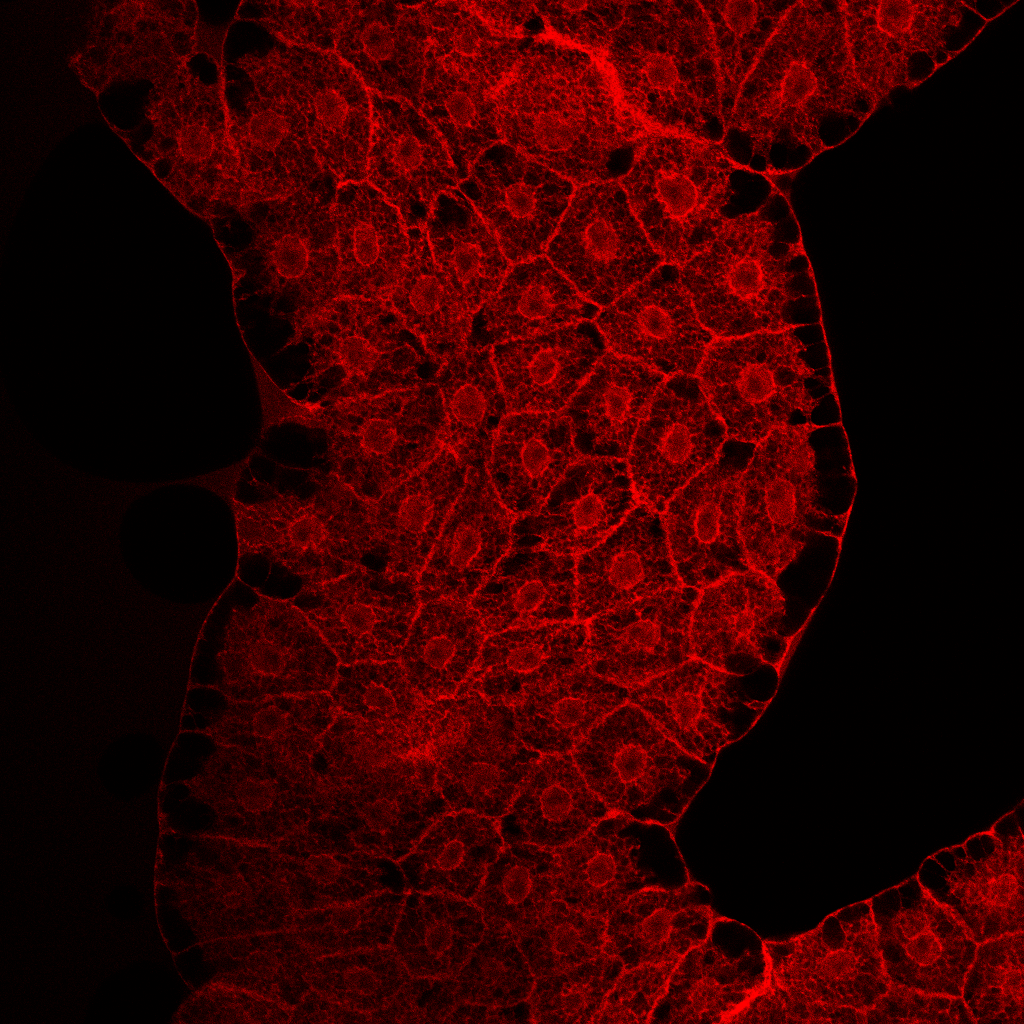

Supplement: Supplementary file 10 — Source data Fig. 6 [file 44318_2025_489_MOESM10_ESM.zip › Figure 6M/8 original image.tif]

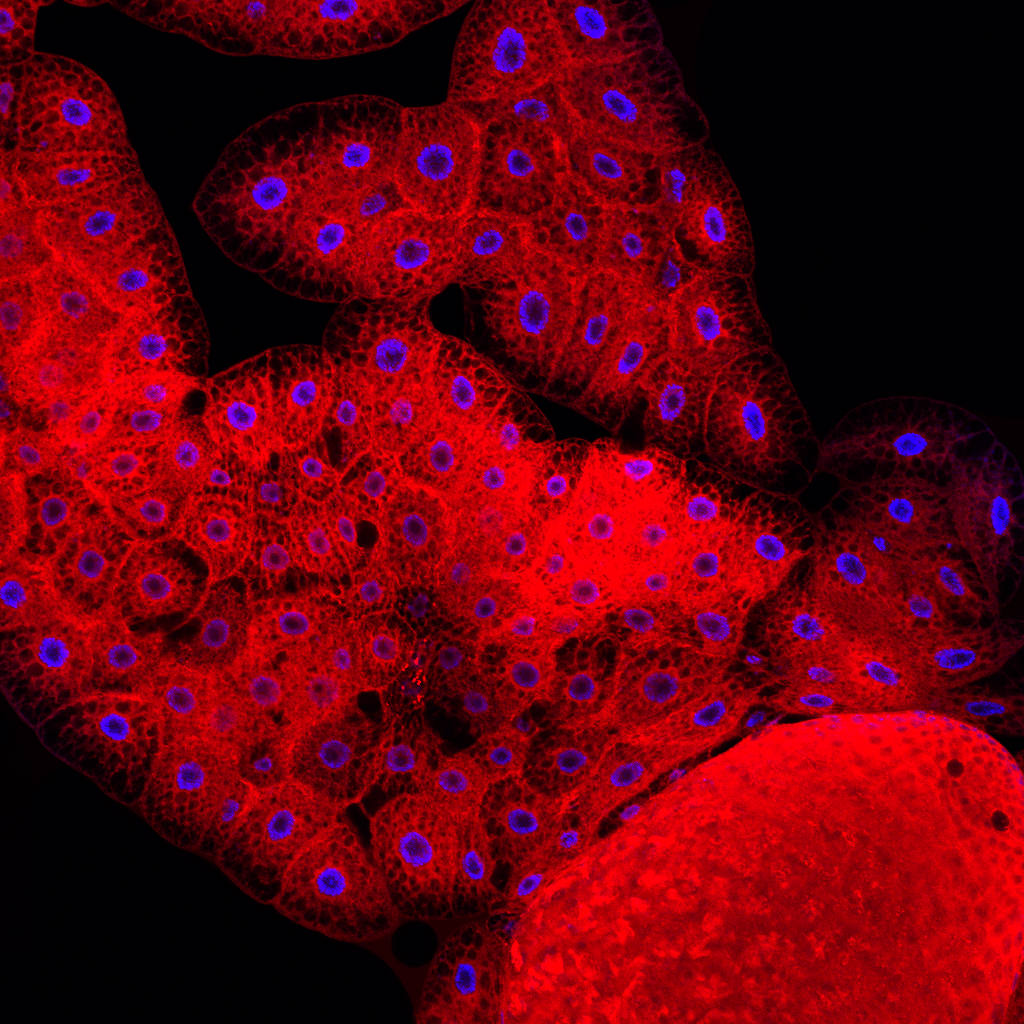

Supplement: Supplementary file 10 — Source data Fig. 6 [file 44318_2025_489_MOESM10_ESM.zip › Figure 6M/9 original image.tif]

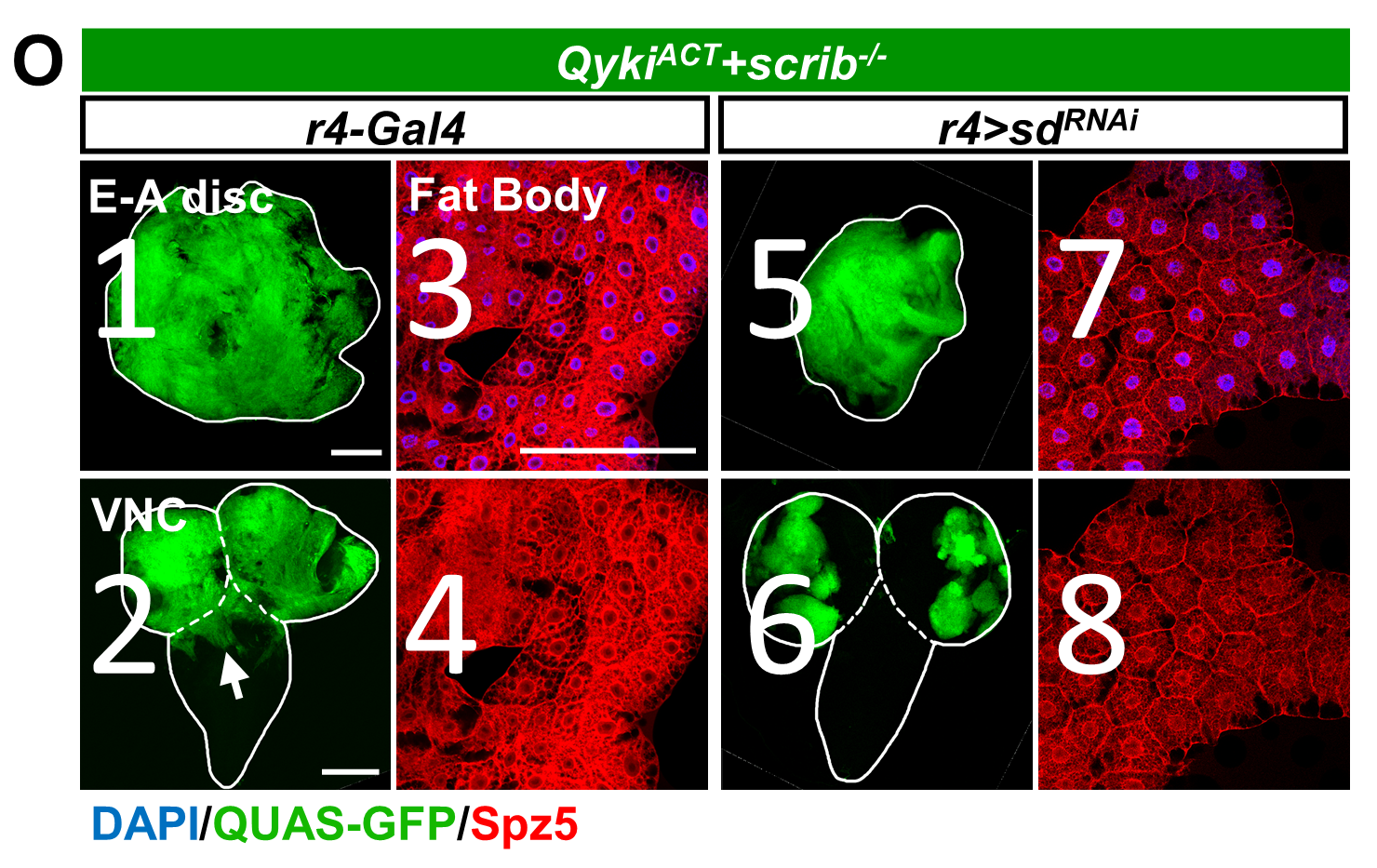

Supplement: Supplementary file 10 — Source data Fig. 6 [file 44318_2025_489_MOESM10_ESM.zip › Figure 6O/0 paper Figure 6O with provided image sequence.tif]

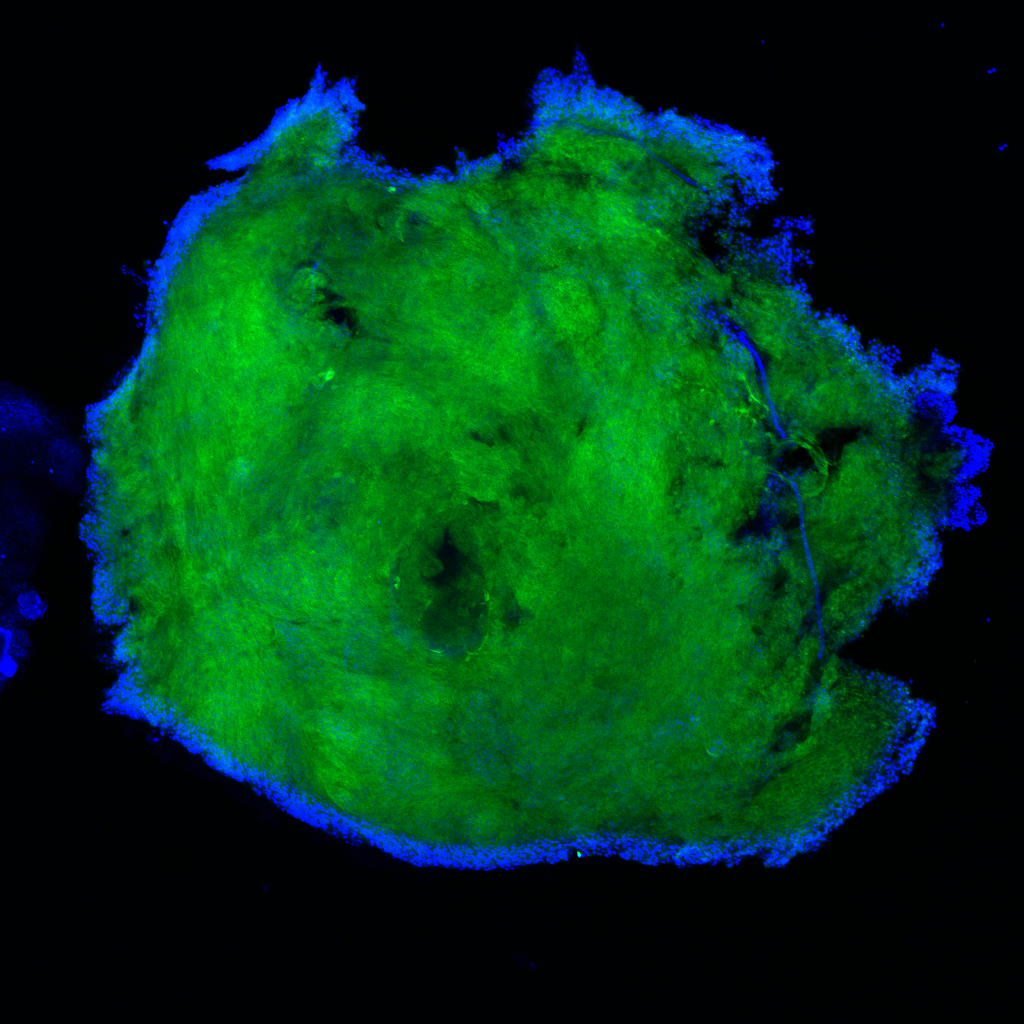

Supplement: Supplementary file 10 — Source data Fig. 6 [file 44318_2025_489_MOESM10_ESM.zip › Figure 6O/1 original image.tif]

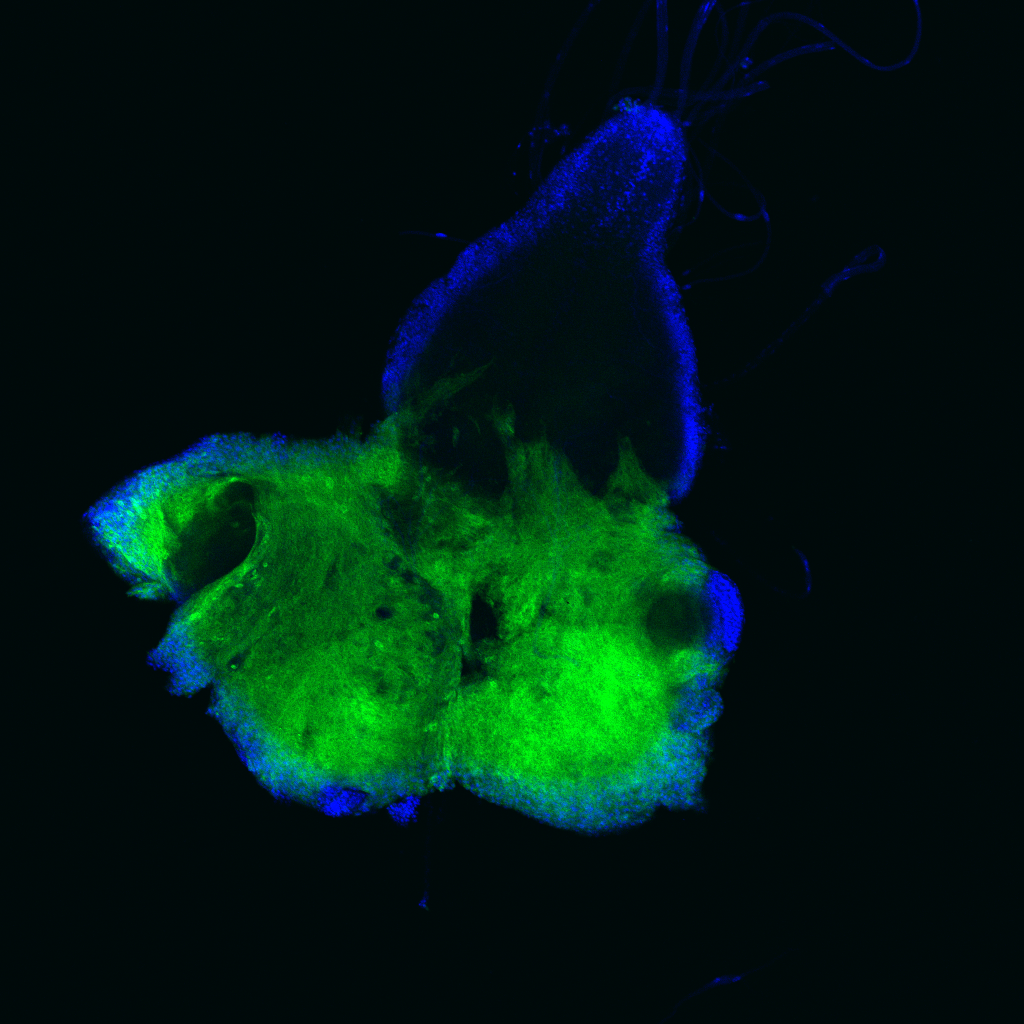

Supplement: Supplementary file 10 — Source data Fig. 6 [file 44318_2025_489_MOESM10_ESM.zip › Figure 6O/2 original image.tif]

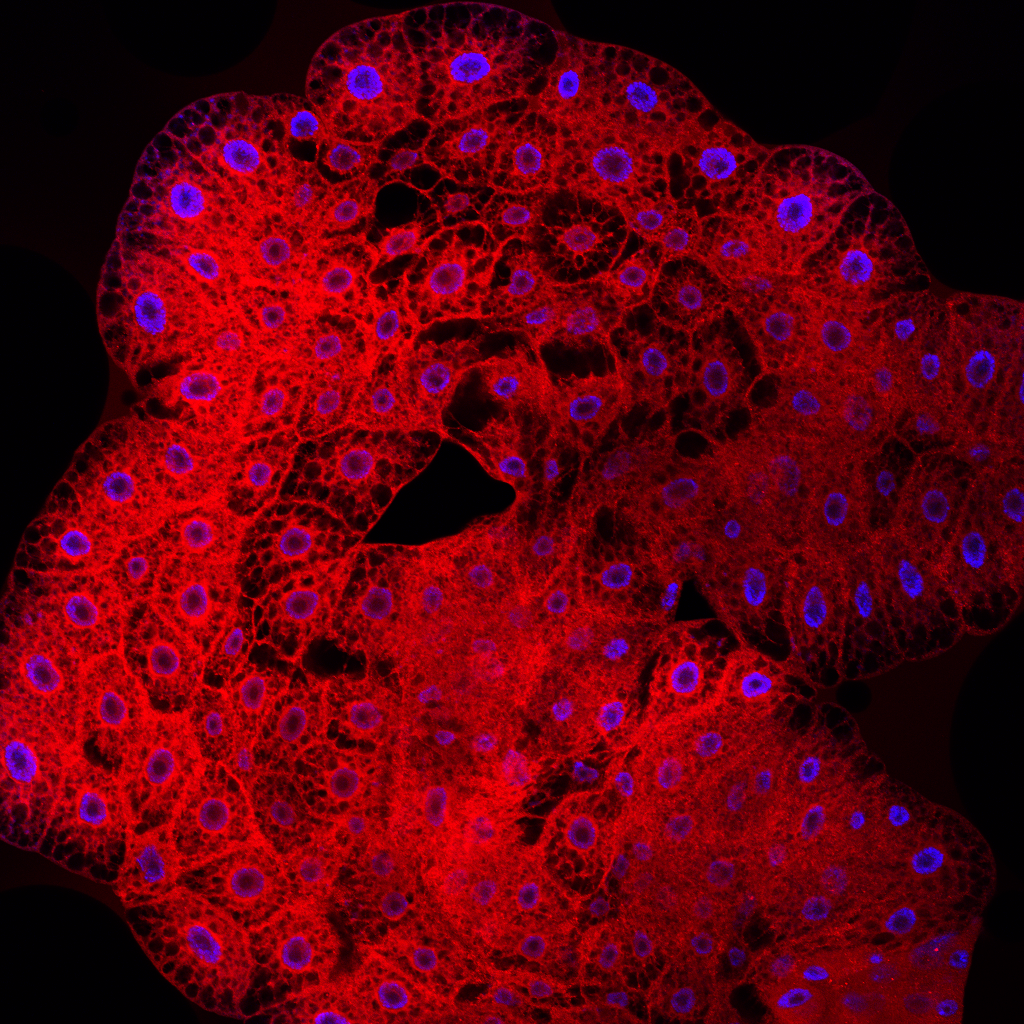

Supplement: Supplementary file 10 — Source data Fig. 6 [file 44318_2025_489_MOESM10_ESM.zip › Figure 6O/3 original image.tif]

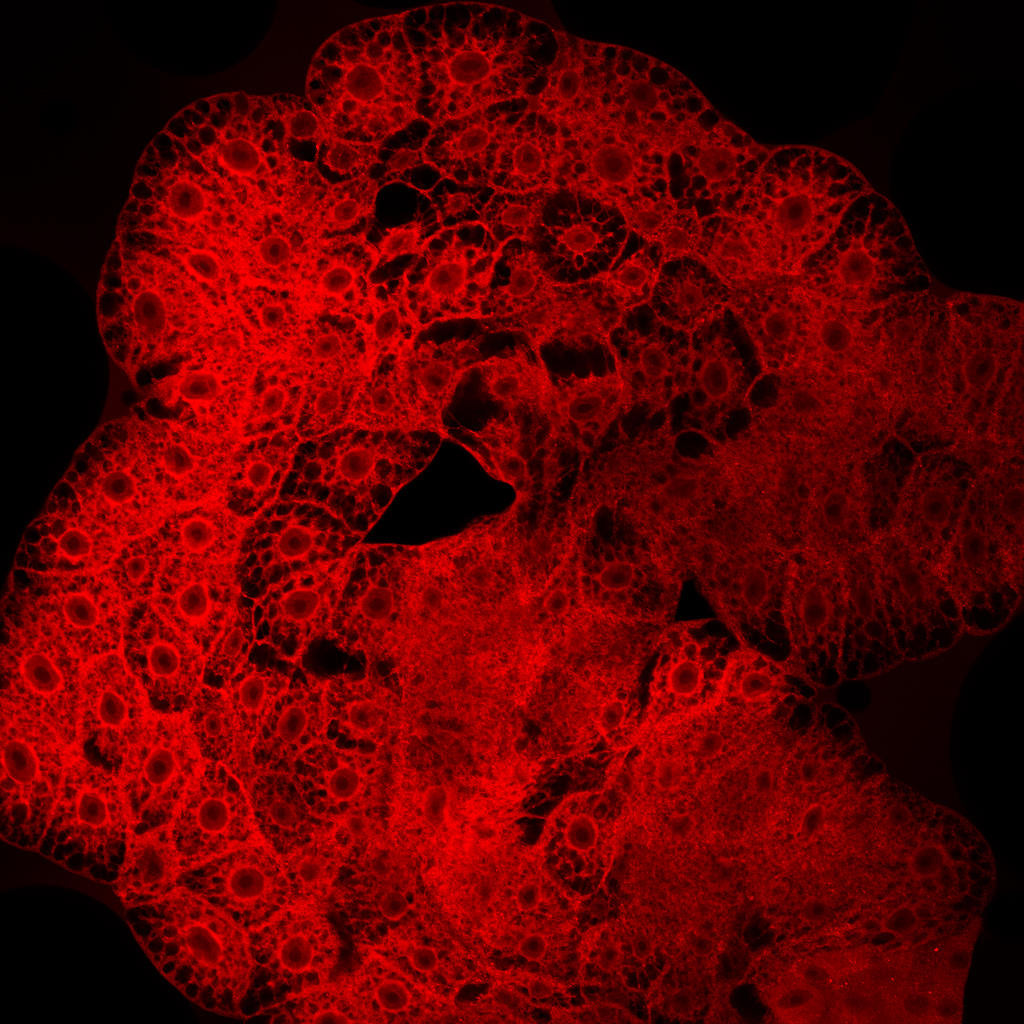

Supplement: Supplementary file 10 — Source data Fig. 6 [file 44318_2025_489_MOESM10_ESM.zip › Figure 6O/4 original image.tif]

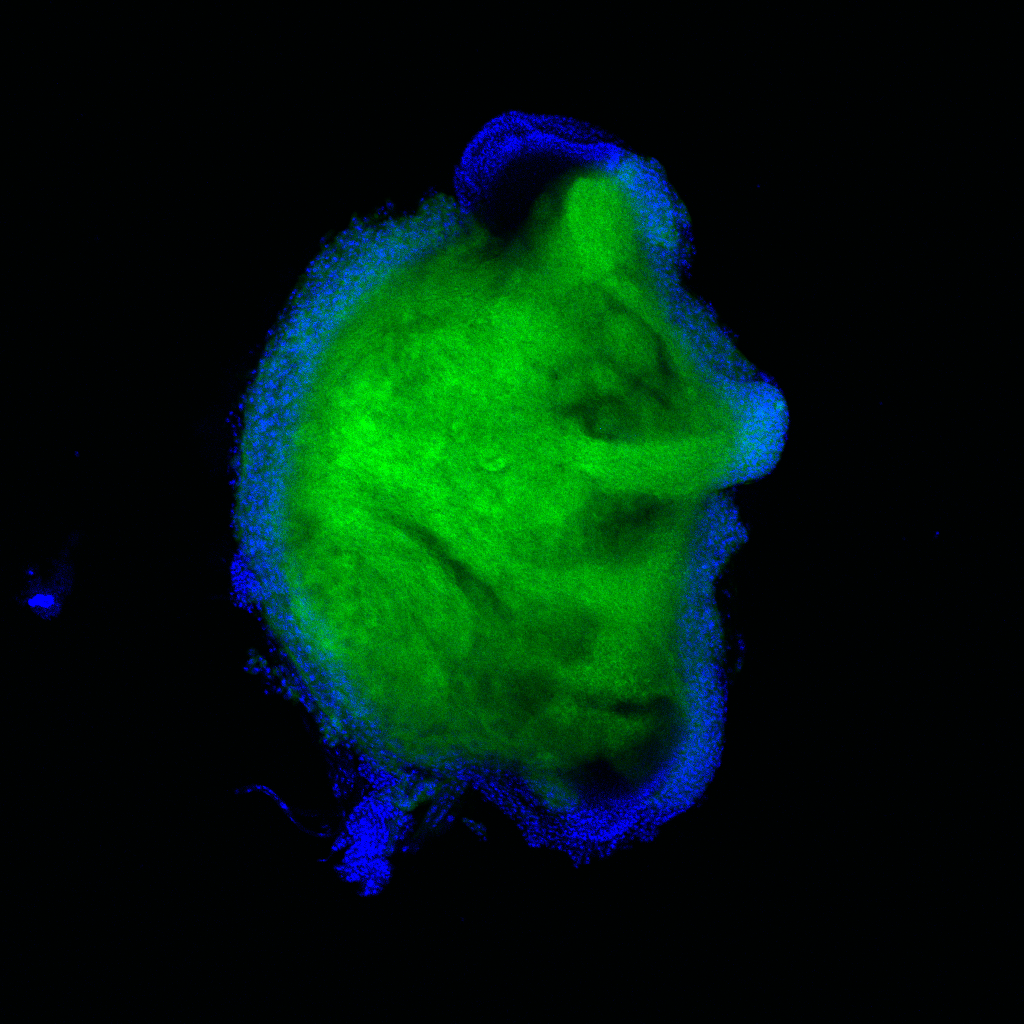

Supplement: Supplementary file 10 — Source data Fig. 6 [file 44318_2025_489_MOESM10_ESM.zip › Figure 6O/5 original image.tif]

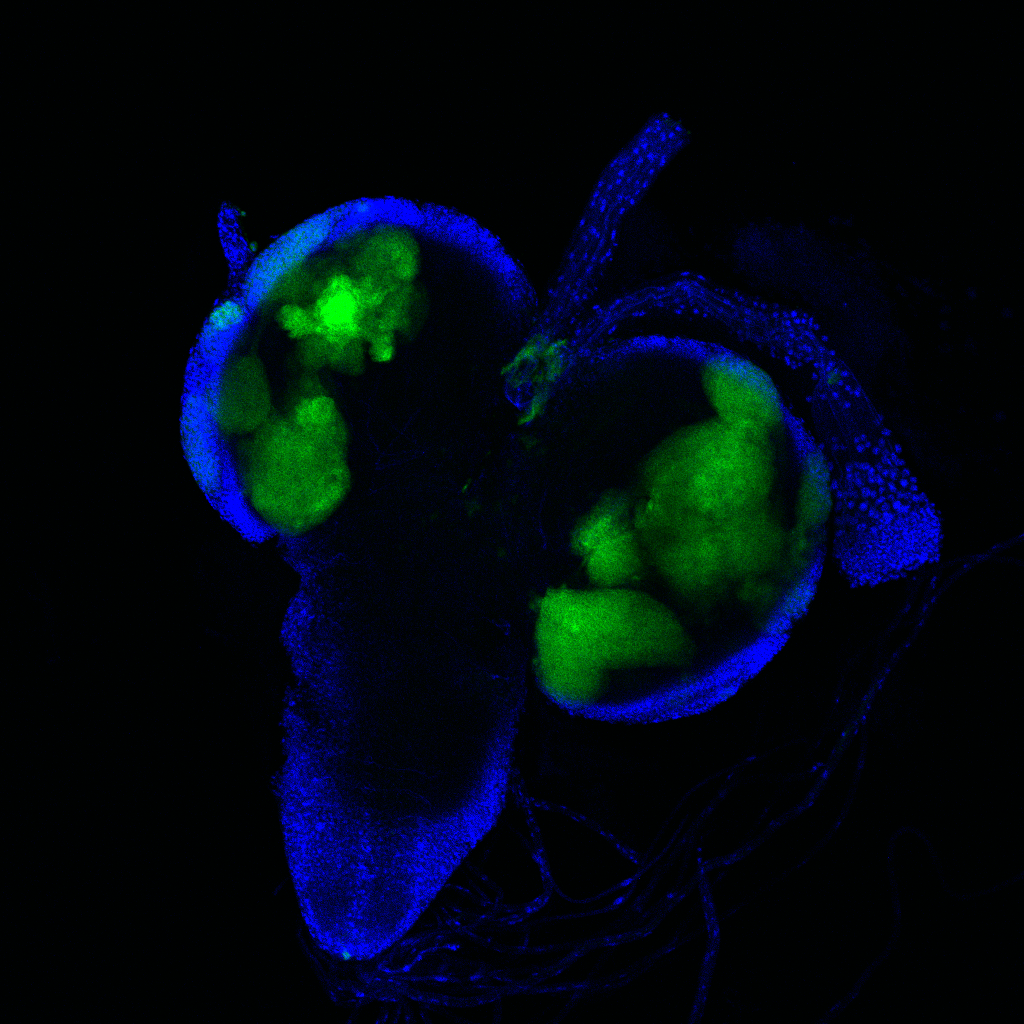

Supplement: Supplementary file 10 — Source data Fig. 6 [file 44318_2025_489_MOESM10_ESM.zip › Figure 6O/6 original image.tif]

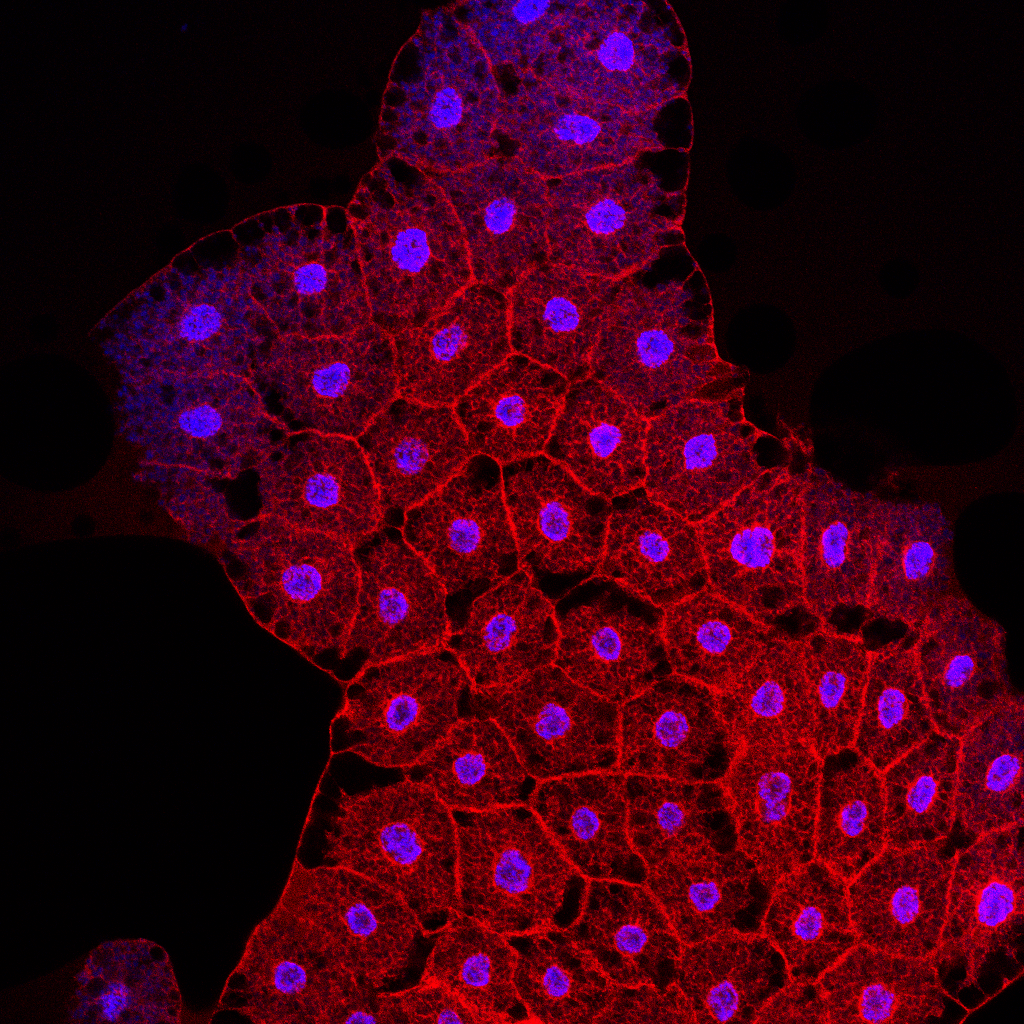

Supplement: Supplementary file 10 — Source data Fig. 6 [file 44318_2025_489_MOESM10_ESM.zip › Figure 6O/7 original image.tif]

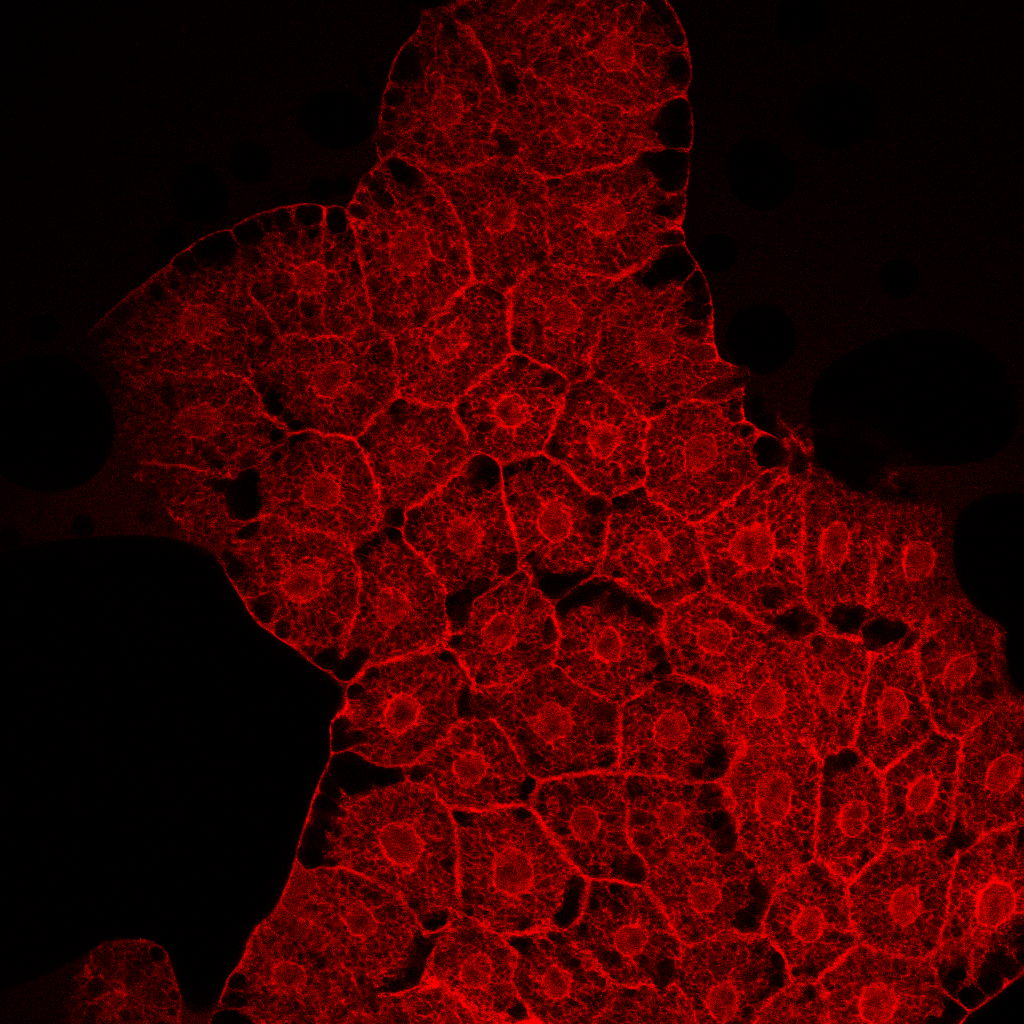

Supplement: Supplementary file 10 — Source data Fig. 6 [file 44318_2025_489_MOESM10_ESM.zip › Figure 6O/8 original image.tif]
