# Supplementary material for: Adipose tissue-secreted Spz5 promotes distal tumor progression via Toll-6-mediated Hh pathway activation in Drosophila
Source: EMBO J. 2025 Jun 23;44(15):4301–30. doi: 10.1038/s44318-025-00489-y (PMC12317064; doi:10.1038/s44318-025-00489-y)
Supplement: Supplementary file 11 — Expanded View Figures [file 44318_2025_489_MOESM11_ESM.pdf]

Expanded View Figures

Figure EV1. Fat body-derived Spz5 is essential for *Qyki*^{ACT}*/scrib*$^{-/-}$ tumor progression in eye-antennal discs.

(**A**) Ventral nerve cord (VNC) with GFP-labeled *WT*, *Qyki*^{ACT}, and *Qyki*^{ACT}*/scrib*$^{-/-}$ clones induced by ey-FLP/QMARCM, stained with anti-Mmp1 antibody. (**B**) Eye-antennal discs (E-A discs, top) and VNC (bottom) with GFP and RFP double-labeled clones, respectively, by QF/QUAS and Gal4/UAS system. (**C**) Cartoon illustrating the *Drosophila* tumor interorgan communication model between fat body or hemocytes and E-A discs. The QMARCM system generates *Qyki*^{ACT}*/scrib*$^{-/-}$ tumor in the larval E-A discs, whereas the Gal4/UAS system expresses UAS-controlled genes in either fat body or hemocytes. (**D**) E-A discs (top) and VNC (bottom) with GFP-labeled *Qyki*^{ACT}*/scrib*$^{-/-}$ clones induced by ey-FLP/QMARCM, and expression of *WT*, *spz5*$^{RNAi\#1}$, and *spz5*$^{RNAi\#2}$ in fat body under control of *r4-Gal4*, the arrow indicates VNC invasion site. (**E**, **F**) Quantification of GFP-labeled clone size in E-A discs (**E**, from left to right, $n = 19$, 25, 23), or percentage of clone invasion into VNC (**F**, total number from four independent experiments, from left to right, $n = 110$, 127, 113) for the indicated genotypes. (**G**) E-A discs (top) and VNC (bottom) with GFP-labeled *Qyki*^{ACT}*/scrib*$^{-/-}$ clones induced by ey-FLP/QMARCM, and expression of *WT*, *spz5*$^{RNAi\#1}$, and *spz5*$^{RNAi\#2}$ in hemocytes under control of *He-Gal4*, the arrows indicate VNC invasion sites. (**H**, **I**) Quantification of GFP-labeled clone size in E-A discs (**H**, from left to right, $n = 20$, 24, 22), or percentage of clone invasion into VNC (**I**, total number from four independent experiments, from left to right, $n = 119$, 113, 139) for the indicated genotypes. The *P* values of (**E**, **F**, **H**, **I**) were determined by unpaired nonparametric Mann–Whitney test. Exact *P* values are shown in the figures. The box plots of (**E**, **F**, **H**, **I**) boundaries represent the 25th (lower quartile) and 75th (upper quartile) percentiles, with the center line indicating the median, and the whiskers extend to the minimum and maximum values. Scale bars: 200 µm for (**A**, **B**, **D**, **G**). DAPI 4′,6-diamidino-2-phenylindole.

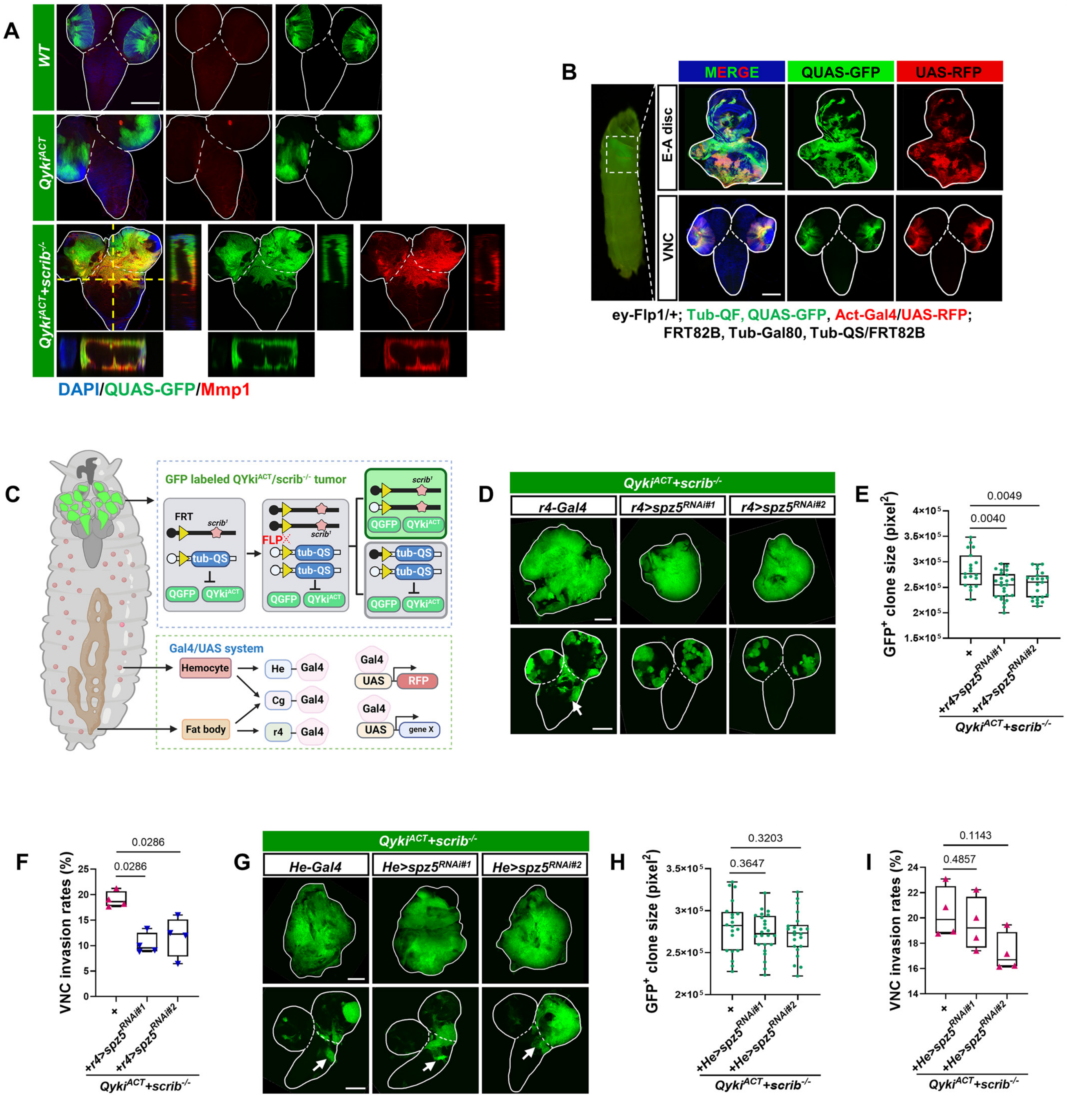
A
WT
Qyki^ACT
Qyki^ACT+scrib-/-
DAPI/QUAS-GFP/Mmp1
B
MERGE
QUAS-GFP
UAS-RFP
E-A disc
VNC
ey-Flp1/+; Tub-QF, QUAS-GFP, Act-Gal4/UAS-RFP;
FRT82B, Tub-Gal80, Tub-QS/FRT82B
C
GFP labeled QYki^ACT/scrib-/- tumor
FRT
scrib1
tub-QS
QGFP
QYki^ACT
FLP
Gal4/UAS system
Hemocyte
Fat body
He
Cg
r4
Gal4
UAS
RFP
gene X
D
Qyki^ACT+scrib-/-
r4-Gal4
r4>spz5^RNAi#1
r4>spz5^RNAi#2
E
GFP+ clone size (pixel2)
0.0049
0.0040
+
+r4>spz5^RNAi#1
+r4>spz5^RNAi#2
Qyki^ACT+scrib-/-
F
VNC invasion rates (%)
0.0286
0.0286
G
He-Gal4
He>spz5^RNAi#1
He>spz5^RNAi#2
H
0.3203
0.3647
+He>spz5^RNAi#1
+He>spz5^RNAi#2
I
0.1143
0.4857

 © The Author(s)

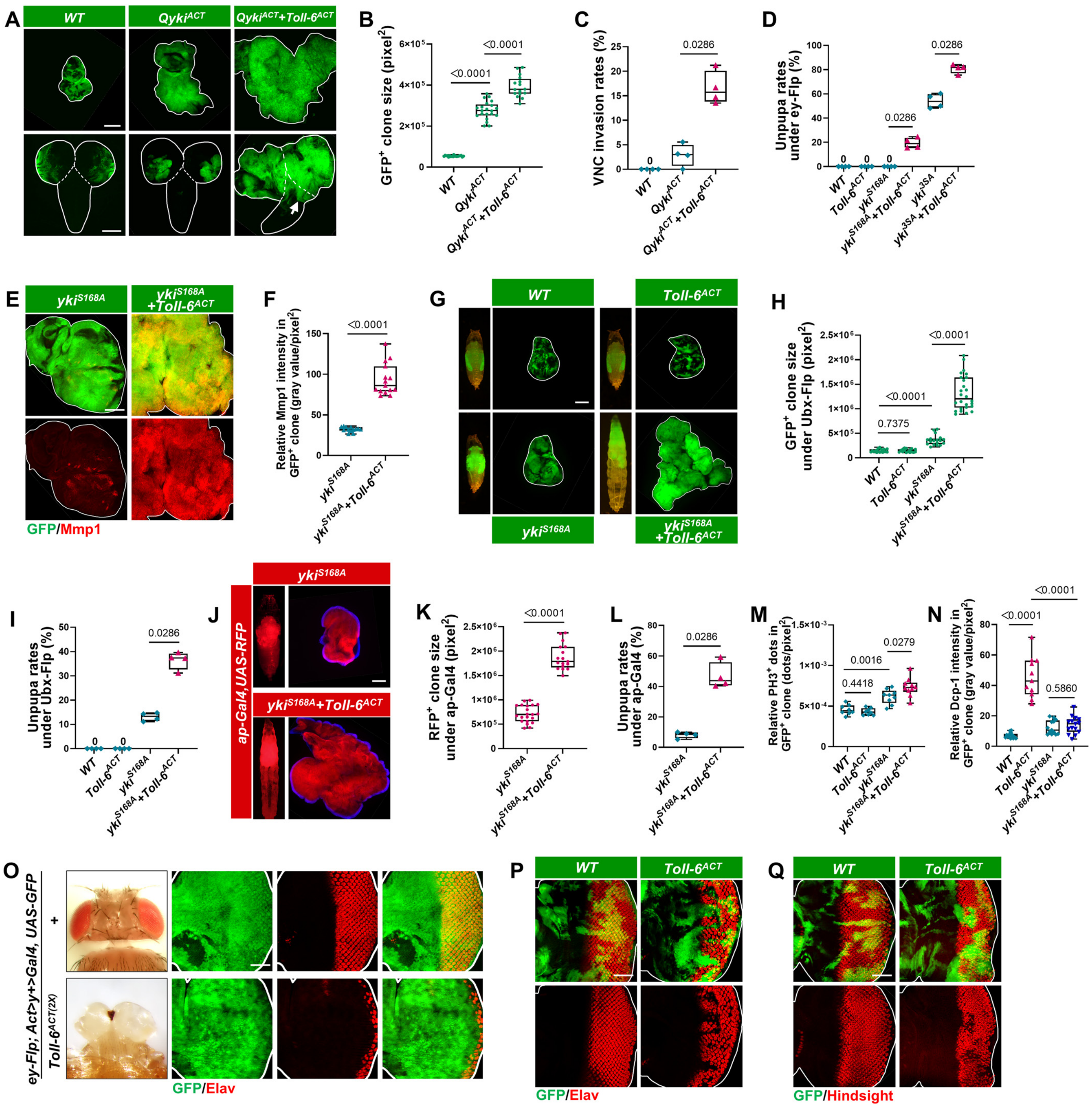

Figure EV2. Activated Toll-6 promotes Yki-driven tumor malignancy.

(**A**) E-A discs (top) and VNC (bottom) with GFP-labeled clones induced by ey-FLP/QMARCM for *WT*, *Qyki^{ACT}*, and *Qyki^{ACT}/Toll-6^{ACT}*, the arrow indicates VNC invasion site. (**B**, **C**) Quantification of GFP-labeled clone size in E-A discs (**B**, from left to right, *n* = 19, 21, 16), or percentage of clone invasion into VNC (**C**, total number from four independent experiments, from left to right, *n* = 116, 134, 140) for the indicated genotypes. (**D**) Quantification of *Drosophila* larval non-pupation rates with indicated genotypes (total number from four independent experiments, from left to right, *n* = 224, 253, 204, 205, 226, 226). (**E**) E-A discs with GFP-labeled *yki^{S168A}* and *yki^{S168A}/Toll-6^{ACT}* clones were stained with anti-Mmp1 antibody. (**F**) Quantification of the relative Mmp1 intensity in clones with indicated genotypes (from left to right, *n* = 13, 15). (**G**) Pupa/larva (left) and wing imaginal discs (right) with GFP-labeled Ubx-FLP/MARCM-induced mosaic clones in *WT*, *Toll-6^{ACT}*, *yki^{S168A}*, and *yki^{S168A}/Toll-6^{ACT}* genotypes. (**H**, **I**) Quantification of GFP-labeled clone size in wing imaginal discs (**H**, from left to right, *n* = 19, 16, 18, 26), or percentage of *Drosophila* larval un-pupation rates (**I**, total number from four independent experiments, from left to right, *n* = 186, 205, 220, 213) for the indicated genotypes. (**J**) RFP-labeled pupa/larva (left) and wing imaginal discs (right) expressing *yki^{S168A}* and *yki^{S168A}/Toll-6^{ACT}* under control of *ap-Gal4*. (**K**, **L**) Quantification of RFP-labeled wing imaginal disc size (**K**, from left to right, *n* = 21, 17), or percentage of *Drosophila* larval un-pupation rates (**L**, total number from four independent experiments, from left to right, *n* = 234, 228) for the indicated genotypes. (**M**) Quantification of PH3-positive dots in GFP-labeled clones with indicated genotypes (from left to right, *n* = 8, 8, 9, 10). (**N**) Quantification of Dcp-1 intensity in GFP-labeled clones with indicated genotypes (from left to right, *n* = 14, 11, 13, 15). (**O**) Adult head (left) and E-A discs (right) stained with anti-Elav antibody with indicated genotypes. (**P**, **Q**) E-A discs with GFP-labeled clones induced by ey-FLP/MARCM for *WT* and *Toll-6^{ACT}* were stained with anti-Elav antibody (**P**) and anti-Hindsight antibody (**Q**). The *P* values of (**B**, **C**, **D**, **F**, **H**, **I**, **K**, **L**, **M**, **N**) were determined by unpaired nonparametric Mann–Whitney test. Exact *P* values are shown in the figures. The box plots of (**B**, **C**, **D**, **F**, **H**, **I**, **K**, **L**, **M**, **N**) boundaries represent the 25th (lower quartile) and 75th (upper quartile) percentiles, with the center line indicating the median, and the whiskers extend to the minimum and maximum values. Scale bars: 200 µm for (**A**, **G**, **J**); 100 µm for (**E**); 50 µm for (**O**–**Q**).

 © The Author(s)

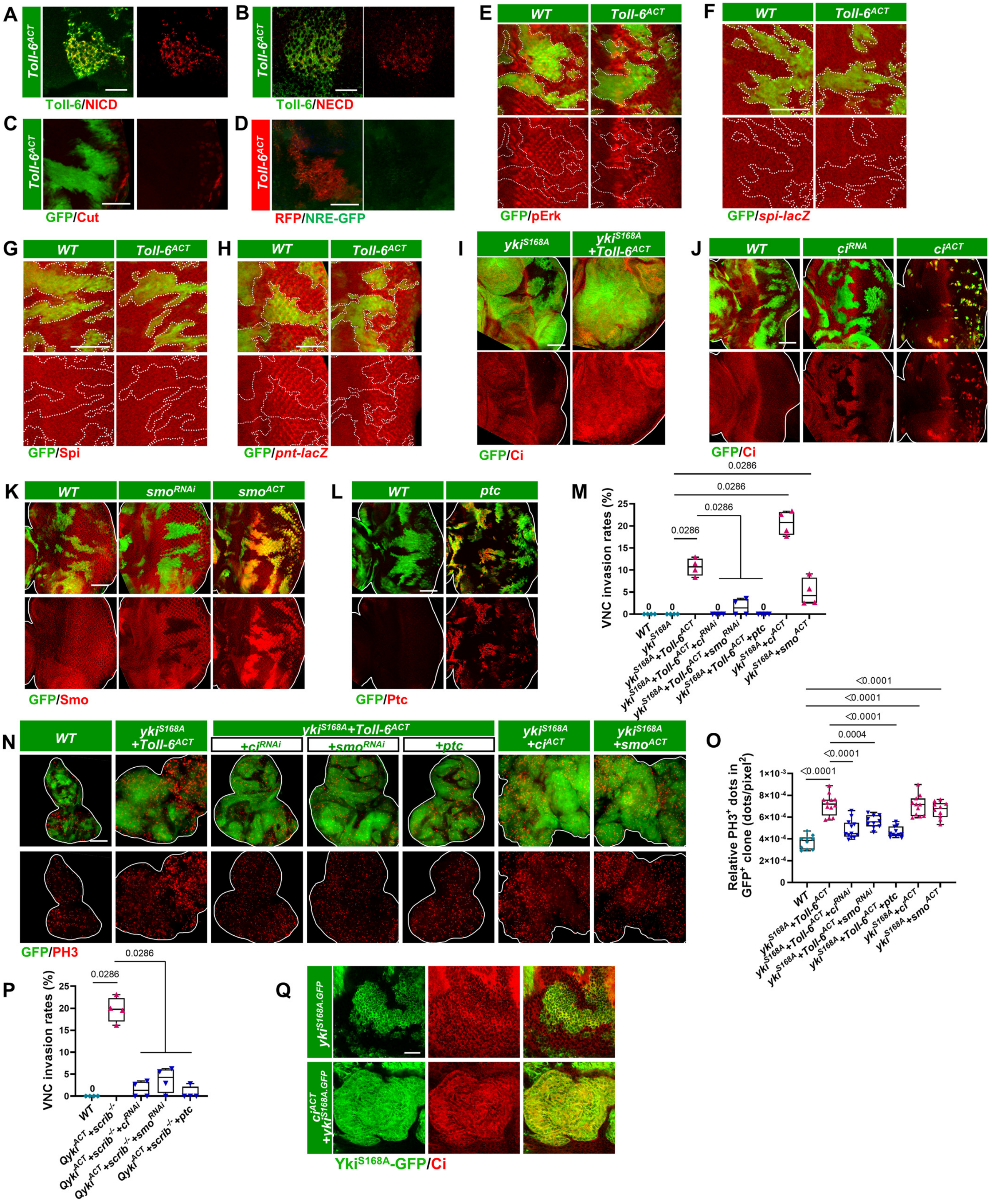
A
Toll-6ACT
Toll-6/NICD
B
Toll-6/NECD
C
GFP/Cut
D
RFP/NRE-GFP
E
WT
GFP/pErk
F
GFP/spi-lacZ
G
GFP/Spi
H
GFP/pnt-lacZ
I
ykiS168A
ykiS168A +Toll-6ACT
GFP/Ci
J
ciRNA
ciACT
GFP/Ci
K
smoRNAi
smoACT
GFP/Smo
L
ptc
GFP/Ptc
M
VNC invasion rates (%)
0.0286
N
+ciRNAi
+smoRNAi
+ptc
ykiS168A +ciACT
ykiS168A +smoACT
GFP/PH3
O
Relative PH3+ dots in GFP+ clone (dots/pixel2)
<0.0001
0.0004
P
QykiACT +scrib-/-
Q
ykiS168A.GFP
ciACT +ykiS168A.GFP
YkiS168A-GFP/Ci

Figure EV3. Cooperation between Hedgehog activation and oncogenic Yki in driving tumorigenesis.

(**A**) E-A discs with *Toll-6*^{ACT} clones, co-immunostained with anti-Toll-6 and anti-NICD antibodies. (**B**) E-A discs with *Toll-6*^{ACT} clones, co-immunostained with anti-Toll-6 and anti-NECD antibodies. (**C**) E-A discs with GFP-labeled *Toll-6*^{ACT} clones were stained with anti-Cut antibody. (**D**) E-A discs with RFP-labeled *Toll-6*^{ACT} clones were examined using the Notch signaling indicator NRE-GFP. (**E**) E-A discs with GFP-labeled *WT* and *Toll-6*^{ACT} clones were stained with anti-pErk antibody. (**F**) E-A discs with GFP-labeled *WT* and *Toll-6*^{ACT} clones were stained with anti-β-galactosidase antibody to label *spi* transcription. (**G**) E-A discs with GFP-labeled *WT* and *Toll-6*^{ACT} clones were stained with anti-Spi antibody. (**H**) E-A discs with GFP-labeled *WT* and *Toll-6*^{ACT} clones were stained with anti-β-galactosidase antibody to label *pnt* transcription. (**I**) E-A discs with GFP-labeled *yki*^{S168A} and *yki*^{S168A}*/Toll-6*^{ACT} clones were stained with anti-Ci antibody. (**J**) E-A discs with GFP-labeled *WT*, *ci*^{RNAi}, and *ci*^{ACT} clones were stained with anti-Ci antibody. (**K**) E-A discs with GFP-labeled *WT*, *smo*^{RNAi}, and *smo*^{ACT} clones were stained with anti-Smo antibody. (**L**) E-A discs with GFP-labeled *WT* and *ptc* overexpression clones were stained with anti-Ptc antibody. (**M**) Quantification of the percentage of clone invasion into VNC with indicated genotypes (total number from four independent experiments, from left to right, $n = 98$, 110, 142, 124, 132, 85, 132, 146), as related to Fig. 3D. (**N**) E-A discs with GFP-labeled *WT*, *yki*^{S168A}*/Toll-6*^{ACT}, *yki*^{S168A}*/Toll-6*^{ACT}*/ci*^{RNAi}, *yki*^{S168A}*/Toll-6*^{ACT}*/smo*^{RNAi}, *yki*^{S168A}*/Toll-6*^{ACT}*/ptc*, *yki*^{S168A}*/ci*^{ACT}, and *yki*^{S168A}*/smo*^{ACT} clones were stained with anti-phospho-histone H3 (PH3) antibody. (**O**) Quantification of the number of PH3-positive cells in GFP-labeled clones with indicated genotypes (from left to right, $n = 10$, 12, 12, 10, 11, 11, 10). (**P**) Quantification of the percentage of clone invasion into VNC with indicated genotypes (total number from four independent experiments, from left to right, $n = 93$, 146, 128, 130, 115), as related to Fig. 3G. (**Q**) E-A discs with GFP-fused *yki*$^{S168A.GFP}$ (top) and *yki*$^{S168A.GFP}$*/ci*^{ACT} (bottom) clones were stained with anti-Ci antibody. The *P* values of (**M**, **O**, **P**) were determined by unpaired nonparametric Mann–Whitney test. Exact *P* values are shown in the figures. The box plots of (**M**, **O**, **P**) boundaries represent the 25th (lower quartile) and 75th (upper quartile) percentiles, with the center line indicating the median, and the whiskers extend to the minimum and maximum values. Scale bars: 100 μm for (**N**); 50 μm for (**C**–**L**); 20 μm for (**A**, **B**, **Q**).

 © The Author(s)

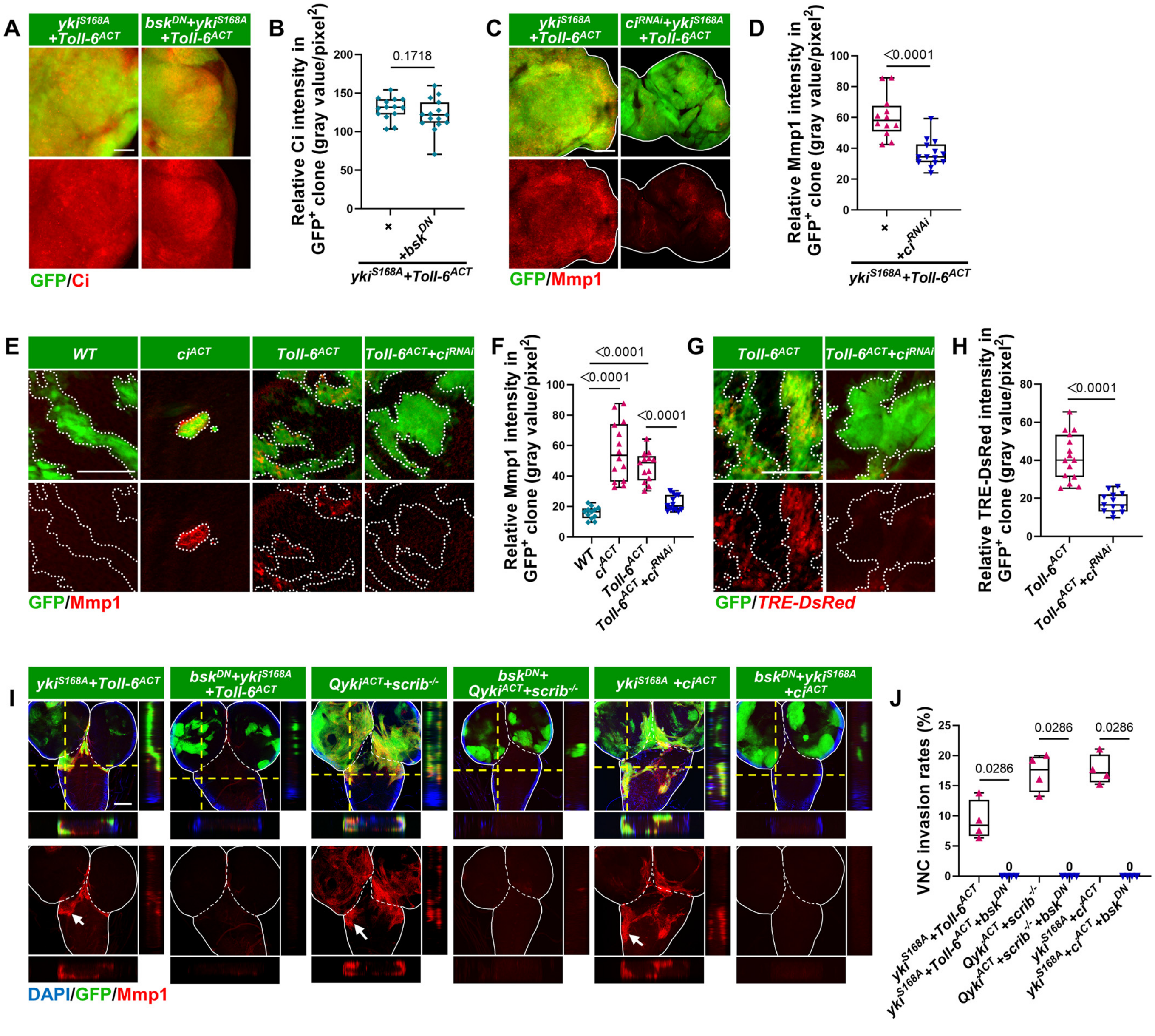

Figure EV4. Hedgehog activation promotes JNK-dependent tumor invasion.

(**A**) E-A discs with GFP-labeled *yki*^{S168A}/*Toll-6*^{ACT} and *yki*^{S168A}/*Toll-6*^{ACT}/*bsk*^{DN} clones were stained with anti-Ci antibody. (**B**) Quantification of the relative Ci intensity in GFP-labeled clones with indicated genotypes (from left to right, $n = 14$, 15). (**C**) E-A discs with GFP-labeled *yki*^{S168A}/*Toll-6*^{ACT} and *yki*^{S168A}/*Toll-6*^{ACT}/*ci*^{RNAi} clones were stained with anti-Mmp1 antibody. (**D**) Quantification of the relative Mmp1 intensity in GFP-labeled clones with indicated genotypes (from left to right, $n = 12$, 14). (**E**) E-A discs with GFP-labeled *WT*, *ci*^{ACT}, *Toll-6*^{ACT}, and *Toll-6*^{ACT}/*ci*^{RNAi} clones were stained with anti-Mmp1 antibody. (**F**) Quantification of the relative Mmp1 intensity in GFP-labeled clones with indicated genotypes (from left to right, $n = 12$, 14, 13, 13). (**G**) E-A discs with GFP-labeled *Toll-6*^{ACT} and *Toll-6*^{ACT}/*ci*^{RNAi} clones expressed the JNK reporter *TRE-DsRed*. (**H**) Quantification of the relative *TRE-DsRed* intensity in GFP-labeled clones with indicated genotypes (from left to right, $n = 15$, 13). (**I**) z-stack confocal images of GFP-labeled *yki*^{S168A}/*Toll-6*^{ACT}, *yki*^{S168A}/*Toll-6*^{ACT}/*bsk*^{DN}, *Qyki*^{ACT}/*scrib*$^{-/-}$, *Qyki*^{ACT}/*scrib*$^{-/-}$/*bsk*^{DN}, *yki*^{S168A}/*ci*^{ACT}, and *yki*^{S168A}/*ci*^{ACT}/*bsk*^{DN} clones were stained with anti-Mmp1 antibody. (**J**) Quantification of the percentage of clone invasion into VNC with indicated genotypes (total number from four independent experiments, from left to right, $n = 241$, 211, 231, 229, 214, 218). The *P* values of (**B**, **D**, **F**, **H**, **J**) were determined by unpaired nonparametric Mann–Whitney test. Exact *P* values are shown in the figures. The box plots of (**B**, **D**, **F**, **H**, **J**) boundaries represent the 25th (lower quartile) and 75th (upper quartile) percentiles, with the center line indicating the median, and the whiskers extend to the minimum and maximum values. Scale bars: 100 µm for (**C**) and I; 50 µm for (**A**, **E**, **G**).

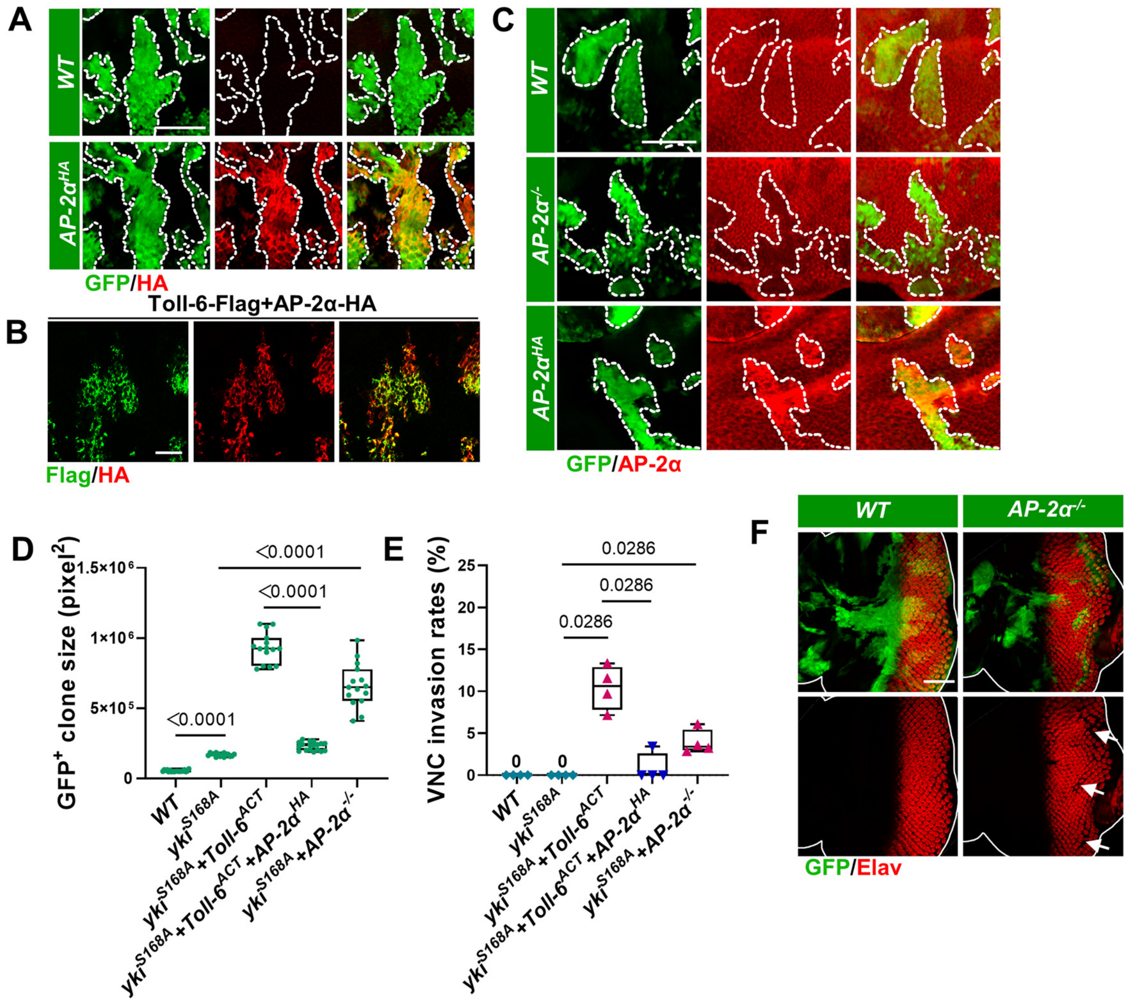

Figure EV5. Toll-6 regulates Hedgehog signaling via AP-2α-dependent endocytosis defects.

(**A**) E-A discs with GFP-labeled *WT* and *AP-2α*^{HA} clones were stained with anti-HA antibody. (**B**) E-A discs with *Toll-6*$^{ACT.Flag}$/*AP-2α*^{HA} clones, co-immunostained with anti-Flag and anti-HA antibodies. (**C**) E-A discs with GFP-labeled *WT*, *AP-2α*$^{-/-}$, and *AP-2α*^{HA} clones were stained with anti-AP-2α antibody. (**D**, **E**) Quantification of GFP-labeled clone size in E-A discs (**D**, from left to right, $n = 19, 16, 15, 18, 15$), or percentage of clone invasion into VNC (**E**, total number from four independent experiments, from left to right, $n = 92, 95, 115, 107, 127$) for the indicated genotypes, as related to Fig. 4H. (**F**) E-A discs with GFP-labeled *WT* (left) and *AP-2α*$^{-/-}$ (right) clones were stained with anti-Elav antibody, the arrows indicate decreased Elav protein level. The *P* values of (**D**, **E**) were determined by unpaired nonparametric Mann–Whitney test. Exact *P* values are shown in the figures. The box plots of (**D**, **E**) boundaries represent the 25th (lower quartile) and 75th (upper quartile) percentiles, with the center line indicating the median, and the whiskers extend to the minimum and maximum values. Scale bars: 50 μm for (**A**, **C**), and (**F**); 20 μm for (**B**).

 © The Author(s)

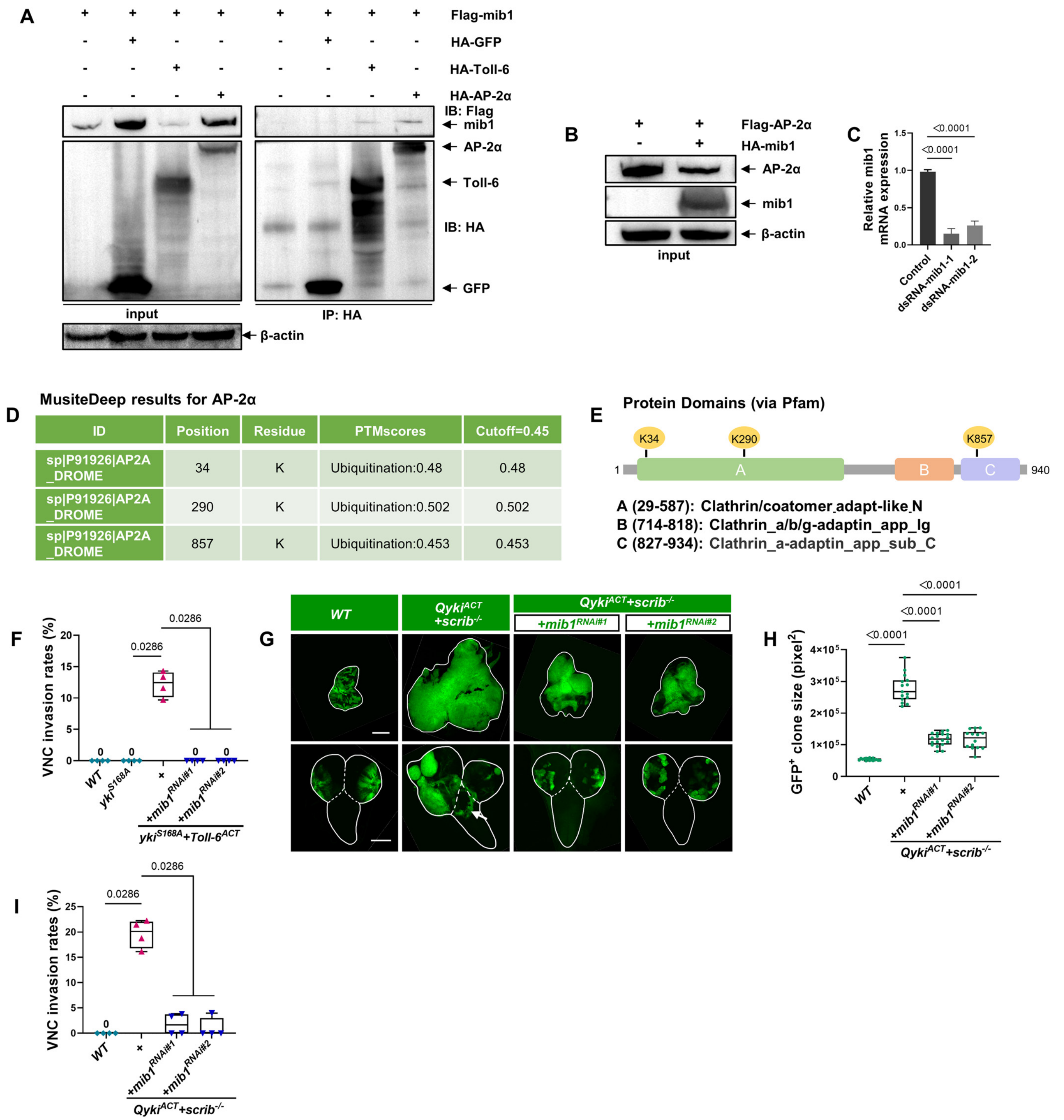
A
Flag-mib1
HA-GFP
HA-Toll-6
HA-AP-2α
IB: Flag
mib1
AP-2α
Toll-6
IB: HA
GFP
input
IP: HA
β-actin
B
Flag-AP-2α
HA-mib1
AP-2α
mib1
β-actin
input
C
Relative mib1 mRNA expression
<0.0001
<0.0001
Control
dsRNA-mib1-1
dsRNA-mib1-2
D
MusiteDeep results for AP-2α
ID | Position | Residue | PTMscores | Cutoff=0.45
sp|P91926|AP2A_DROME | 34 | K | Ubiquitination:0.48 | 0.48
sp|P91926|AP2A_DROME | 290 | K | Ubiquitination:0.502 | 0.502
sp|P91926|AP2A_DROME | 857 | K | Ubiquitination:0.453 | 0.453
E
Protein Domains (via Pfam)
K34
K290
K857
1
940
A (29-587): Clathrin/coatomer adapt-like N
B (714-818): Clathrin_a/b/g-adaptin_app_Ig
C (827-934): Clathrin_a-adaptin_app_sub_C
F
VNC invasion rates (%)
0.0286
0.0286
WT
yki S168A
+mib1 RNAi#1
+mib1 RNAi#2
yki S168A +Toll-6 ACT
G
WT
Qyki ACT +scrib -/-
+mib1 RNAi#1
+mib1 RNAi#2
H
GFP+ clone size (pixel2)
<0.0001
<0.0001
<0.0001
I
VNC invasion rates (%)
0.0286
0.0286
Qyki ACT +scrib -/-

Figure EV6. Toll-6 physically interacts with Mib1 to degrade AP-2α.

(**A**) S2 cells transfected with HA-tagged AP-2α, HA-tagged Toll-6 and Flag-tagged Mib1. Mib1 physically interacts with Toll-6 and AP-2α. (**B**) The lysates were analyzed by western blotting. Mib1 can reduce the protein expression of AP-2α. (**C**) qRT-PCR analysis showing efficiency of dsRNA-mediated depletion of Mib1. (**D**) The detailed information on ubiquitination sites identified through MusiteDeep. Three AP-2α sites (K34, K290, and K857) were identified in the framework (Cut off = 0.45). (**E**) Distribution of AP-2α sites identified on Pfam. K34 and K290 are located in the N-terminal region, whereas K857 is located in C-terminal region. (**F**) Quantification of the percentage of clone invasion into VNC (total number from four independent experiments, from left to right, $n = 74, 95, 115, 142, 113$) for the indicated genotypes. (**G**) E-A discs (top) and VNC (bottom) with ey-FLP/QMARCM-induced GFP-labeled clones of *WT*, $Qyki^{ACT}/scrib^{-/-}$, $Qyki^{ACT}/scrib^{-/-}/mib1^{RNAi\#1}$, and $Qyki^{ACT}/scrib^{-/-}/mib1^{RNAi\#2}$, the arrow indicates VNC invasion site. (**H**, **I**) Quantification of GFP-labeled clone size in E-A discs (**H**, from left to right, $n = 16, 15, 18, 14$), or percentage of clone invasion into VNC (**I**, total number from four independent experiments, from left to right, $n = 88, 118, 109, 102$) for the indicated genotypes. The *P* value of (**C**) was determined using one-way ANOVA with Tukey's multiple comparison test; the *P* values of (**F**, **H**, **I**) were determined by unpaired nonparametric Mann–Whitney test. Exact *P* values are shown in the figures. The box plots of (**F**, **H**, **I**) boundaries represent the 25th (lower quartile) and 75th (upper quartile) percentiles, with the center line indicating the median, and the whiskers extend to the minimum and maximum values. Scale bars: 200 µm for (**G**).

© The Author(s)

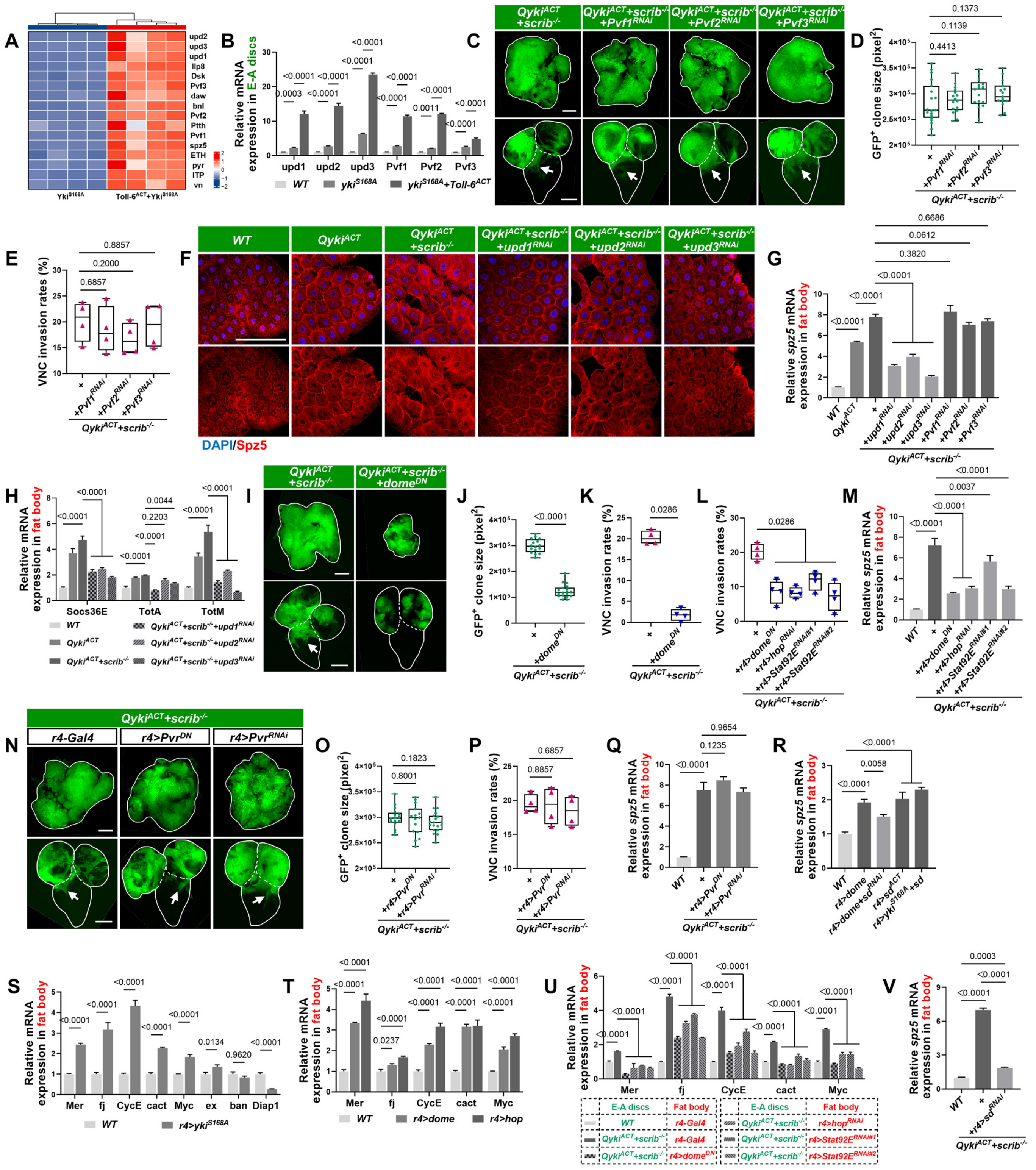
A
B
C
D
E
F
G
H
I
J
K
L
M
N
O
P
Q
R
S
T
U
V
upd1 upd2 upd3 Pvf1 Pvf2 Pvf3
WT
Relative mRNA expression in E-A discs
Relative mRNA expression in fat body
Relative spz5 mRNA expression in fat body
GFP+ clone size (pixel2)
VNC invasion rates (%)
DAPI/Spz5
Socs36E TotA TotM
Mer fj CycE cact Myc ex ban Diap1
r4-Gal4
E-A discs
Fat body

Figure EV7. Tumor-derived Upds promote Spz5 secretion from the fat body.

(**A**) Heatmap of selected top differentially expressed *Drosophila* secreted ligands between the *yki*^{S168A} and *yki*^{S168A}*/Toll-6*^{ACT} samples. Normalized gene expression values were calculated as log2(CPM + 1) and scaled by column. (**B**) Quantitative real-time PCR (qRT-PCR) to determine the mRNA levels of indicated genes in E-A discs with *WT*, *yki*^{S168A}, or *yki*^{S168A}*/Toll-6*^{ACT} genotypes ($n = 3$ independent experiments). (**C**) E-A discs (top) and VNC (bottom) with ey-FLP/QMARCM-induced GFP-labeled clones of *Qyki*^{ACT}*/scrib*$^{-/-}$, *Qyki*^{ACT}*/scrib*$^{-/-}$*/Pvf1*^{RNAi}, *Qyki*^{ACT}*/scrib*$^{-/-}$*/Pvf2*^{RNAi}, and *Qyki*^{ACT}*/scrib*$^{-/-}$*/Pvf3*^{RNAi}. Arrows indicate VNC invasion sites. (**D**, **E**) Quantification of GFP-labeled clone size in E-A discs (**D**, from left to right, $n = 19$, 16, 17, 15), or percentage of clone invasion into VNC (**E**, total number from four independent experiments, from left to right, $n = 230$, 214, 229, 236) for the indicated genotypes. (**F**) Fat bodies dissected from larvae bearing ey-FLP/QMARCM clones were stained with anti-Spz5 antibody. (**G**) qRT-PCR to determine spz5 mRNA levels in fat bodies with the indicated genotypes ($n = 3$ independent experiments). (**H**) qRT-PCR to determine the mRNA levels of Socs36E, TotA, and TotM in fat bodies with the indicated genotypes ($n = 3$ independent experiments). (**I**) E-A discs (top) and VNC (bottom) with ey-FLP/QMARCM-induced GFP-labeled clones of *Qyki*^{ACT}*/scrib*$^{-/-}$ and *Qyki*^{ACT}*/scrib*$^{-/-}$*/dome*^{DN}, the arrow indicates VNC invasion site. (**J**, **K**) Quantification of GFP-labeled clone size in E-A discs (**J**, from left to right, $n = 16$, 18), or percentage of clone invasion into VNC (**K**, total number from four independent experiments, from left to right, $n = 227$, 240) for the indicated genotypes. (**L**) Quantification of the percentage of clone invasion into VNC with indicated genotypes (total number from four independent experiments, from left to right, $n = 234$, 199, 242, 217, 196), related to Fig. 6I. (**M**) qRT-PCR to determine spz5 mRNA levels in fat bodies with the indicated genotypes ($n = 3$ independent experiments), related to Fig. 6I. (**N**) E-A discs (top) and VNC (bottom) with GFP-labeled *Qyki*^{ACT}*/scrib*$^{-/-}$ clones induced by ey-FLP/QMARCM, and expression of *WT*, *Pvr*^{DN}, and *Pvr*^{RNAi} in fat bodies under the control of *r4-Gal4*; the arrows indicate VNC invasion sites. (**O**, **P**) Quantification of GFP-labeled clone size in E-A discs (**O**, from left to right, $n = 16$, 15, 19), or percentage of clone invasion into VNC (**P**, total number from four independent experiments, from left to right, $n = 220$, 220, 222) for the indicated genotypes. (**Q**) qRT-PCR to determine spz5 mRNA levels in fat bodies with the indicated genotypes ($n = 3$ independent experiments). (**R**) qRT-PCR to determine the spz5 mRNA levels in fat bodies under the control of an *r4-Gal4* promoter to express *WT*, *dome*, *dome/sd*^{RNAi}, *sd*^{ACT}, and *yki*^{S168A}*/sd* ($n = 3$ independent experiments). (**S**) qRT-PCR to determine the mRNA levels of Hippo pathway target genes in fat bodies under the control of an *r4-Gal4* promoter to express *WT* or *yki*^{S168A} ($n = 3$ independent experiments). (**T**) qRT-PCR to determine the mRNA levels of Hippo pathway target genes in fat bodies under the control of an *r4-Gal4* promoter to express *WT*, *dome*, or *hop* ($n = 3$ independent experiments). (**U**) qRT-PCR to determine the mRNA levels of Hippo pathway target genes in fat bodies with the indicated genotypes ($n = 3$ independent experiments), with E-A disc genotypes labeled in green and fat body genotypes labeled in red. (**V**) qRT-PCR to determine spz5 mRNA levels in fat bodies with the indicated genotypes ($n = 3$ independent experiments), related to Fig. 6O. The *P* values of (**B**, **G**, **H**, **M**, **Q**, **R**, **S**, **T**, **U**, **V**) were determined using one-way ANOVA with Tukey's multiple comparison test; the *P* values of (**D**, **E**, **J**, **K**, **L**, **O**, **P**) were determined by unpaired nonparametric Mann–Whitney test. Exact *P* values are shown in the figures. The box plots of (**D**, **E**, **J**, **K**, **L**, **O**, **P**) boundaries represent the 25th (lower quartile) and 75th (upper quartile) percentiles, with the center line indicating the median, and the whiskers extend to the minimum and maximum values. Scale bars: 200 µm for (**C**, **F**, **I**, **N**). DAPI 4′,6-diamidino-2-phenylindole.

© The Author(s)
